# Assembly and analysis of a male sterile rubber tree mitochondrial genome reveals DNA rearrangement events and a novel transcript

**DOI:** 10.1186/1471-2229-14-45

**Published:** 2014-02-10

**Authors:** Jeremy R Shearman, Duangjai Sangsrakru, Panthita Ruang-areerate, Chutima Sonthirod, Pichahpuk Uthaipaisanwong, Thippawan Yoocha, Supannee Poopear, Kanikar Theerawattanasuk, Somvong Tragoonrung, Sithichoke Tangphatsornruang

**Affiliations:** 1 National Center for Genetic Engineering and Biotechnology, 113 Thailand Science Park, Paholyothin Road, Khlong Nueng, Khlong Luang, Pathumthani 12120, Thailand; 2 Rubber Research Institute of Thailand (RRIT), Department of Agriculture, Ministry of Agriculture and Cooperatives, 50 Phaholyothin Road, Chatuchack, Bangkok 10900, Thailand

**Keywords:** Rubber tree, Hevea brasiliensis, Mitochondria, Cytoplasmic male sterility, Genome sequencing

## Abstract

**Background:**

The rubber tree, *Hevea brasiliensis*, is an important plant species that is commercially grown to produce latex rubber in many countries. The rubber tree variety BPM 24 exhibits cytoplasmic male sterility, inherited from the variety GT 1.

**Results:**

We constructed the rubber tree mitochondrial genome of a cytoplasmic male sterile variety, BPM 24, using 454 sequencing, including 8 kb paired-end libraries, plus Illumina paired-end sequencing. We annotated this mitochondrial genome with the aid of Illumina RNA-seq data and performed comparative analysis. We then compared the sequence of BPM 24 to the contigs of the published rubber tree, variety RRIM 600, and identified a rearrangement that is unique to BPM 24 resulting in a novel transcript containing a portion of *atp9*.

**Conclusions:**

The novel transcript is consistent with changes that cause cytoplasmic male sterility through a slight reduction to ATP production efficiency. The exhaustive nature of the search rules out alternative causes and supports previous findings of novel transcripts causing cytoplasmic male sterility.

## Background

Mitochondria are membrane-bounded organelles that function in energy metabolism, biosynthesis of cofactors and vitamins, cellular differentiation, signalling, cell growth, and cell death [1]. They contain their own genomes which are inherited maternally in most plant species. The first flowering plant mitochondrial DNA (mtDNA) to be completely sequenced was *Arabidopsis thaliana*[2], since then there have only been 37 additional mitochondrial genomes sequenced and analyzed from flowering plants [http://www.ncbi.nlm.nih.gov/Genomes/]. These additional mitochondrial genomes have increased our understanding of genome rearrangement, DNA transfer and phylogenetic diversity. Plant mitochondrial genomes encode tRNAs, rRNAs, proteins and ribosomal proteins and range in size from 200 Kb in *Brassica hirta*[3] to 2.74 Mb in *Cucumis melo*[4]. Mitochondrial genome expansion in land plants is primarily due to large intergenic regions, repeated segments, intron expansion and incorporation of foreign DNA such as plastid and nuclear DNA [5,6]. Accumulation of repetitive sequences in plant mitochondrial genomes cause frequent recombination events and dynamic genome rearrangements within a species [7,8]. Several mutations by gene rearrangement of the mitochondrial genes were found associated with cytoplasmic male sterility (CMS) such as the *T-urf13* gene in maize [9], *pcf* gene (a fusion of *atp9* and *cox2* portions) in petunia [10], *cox1* in rice [11] and mutations in ATPase subunits in sunflower [12] and Brassica [13]. RNA processing also plays an important role in controlling CMS as evidenced in *orf355/orf77* (*atp9*) and *T-urf13* in maize [14,15].

Conventional strategies for obtaining mitochondrial genome sequencing involve isolation of mitochondrial DNA, cloning and sequencing. However, problems with this approach of mitochondrial genome sequencing include difficulty resolving sequence of the mitochondrial genome from the nuclear genome and assembly of a single circular genome due to the highly dynamic genome structure. Rivarola et al. [16] suggested that examination of the read depths of the resulting assemblies could be used to separate reads of nuclear, chloroplast and mitochondria origin. With the development of next generation sequencing (NGS) technologies, new strategies have been used to obtain plant mitochondrial genomes. A combination approach of shotgun and paired-end NGS sequencing from non-enriched whole genome DNA libraries have been successfully used to obtain the mitochondrial genomes of melon [4], carrot [17] and date palm [18].

*Hevea brasiliensis*, or rubber tree, is an important economical plant that can produce natural latex at a commercial scale. Sequencing information of its nuclear genome [19], plastid genome [20] and mitochondrial genome is important for genetic improvement and understanding of biological mechanisms of the plant species. The closest plant species to *H. brasiliensis* with a mitochondrial genome draft reported is from *Ricinus communis* which is in the same Euphorbiaceae family [16]. In this study, we obtained a draft of the rubber tree mitochondrial genome of the variety BPM 24, a cytoplasmic male sterile descendant of a GT 1 (female) × AVROS 1734 (male) cross [21]. The variety GT 1 is male sterile, its offspring BPM 24 is male sterile and the offspring of BPM 24 are also male sterile. Thus the cause for male sterility in this line is cytoplasmically inherited, which makes the mitochondrion the most probable cause. The assembled BPM 24 genome was characterized for gene annotation, transcription analysis, RNA editing events, sequence variation and recombinations within the species that cause cytoplasmic male sterility in rubber tree.

## Methods

### Plant materials

Shoot apical meristem samples of *H. brasiliensis* (varieties BPM 24, RRII 105, RRIC 110, PB 235, RRIT 251 and RRIM 600) were collected for DNA and RNA extraction from an experimental field at the Rubber Research Institute of Thailand, Ministry of Agriculture and Cooperatives, Thailand. The samples for DNA extraction were processed using the DNeasy Plant Mini Kit (Qiagen, CA, USA). The samples for RNA extraction were immediately frozen in liquid nitrogen and stored at -80°C until RNA extraction following the protocols in Triwitayakorn et al. [22].

### Sequence analysis

The DNA from variety BPM 24 was sequenced in house on a Genome Sequencer (GS) FLX platform (Roche, USA) using two libraries: shotgun sequencing and 8-kb paired-end sequencing according to Roche protocols. In addition this sample was sequenced on a Hiseq 2000 platform (Illumina, USA) using paired-end sequencing at Macrogen (Korea). The genomic sequencing reads from 454 were assembled *de novo* using gsAssembler (Newbler, version 2.7, Roche, USA). Scaffolds were produced using SSPACE_basic_V2.0 [23]. The scaffold graph was produced using bb.454contignet [17]. The assembled contigs were searched for sequence homology against the publicly available plant mitochondrial genomes and repeats were identified using Reputer. The Illumina data was mapped to the 454 assembled contigs to improve on the assembly and the sequence depth was used to differentiate between mitochondrial sequences and nuclear encoded mitochondrial copies. To identify regions of plastid origin, the assembled sequences were aligned against the rubber tree chloroplast genome [20] using BLAST. Comparison of mitochondrial genome structures of rice, tobacco, castor bean and rubber tree was performed using MAUVE [24].

The extracted RNA from the six rubber tree varieties were sequenced on an Illumina HiSeq2000 at Macrogen (Korea). RNA sequence data quality was checked using FastQC and was cleaned using TRIMMOMATIC v0.27 [25]. The reads were mapped to the assembled genome using TopHat (v2.0.9) [26] with bowtie (v1.0.0) [27] and the fusion search option.

### Sequence annotation

Open Reading Frames (ORFs) were predicted using Open Reading Frame Finder [https://www.ncbi.nlm.nih.gov/gorf/gorf.html]. The tRNA genes were searched using tRNAscan-SE [28]. The annotated genes were also checked with the plant mitochondrial genome annotation program Mitofy [29]. All predicted ORFs, tRNA genes and rRNA genes were searched against the publicly available mitochondrial nucleotide and protein sequence database. Expression of genes was checked by mapping the RNA sequencing data from each sample to the assembled genome using TopHat. RNA-editing events were identified from this mapping data using VarScan (v2.3.4) [30], in addition RNA-editing events were predicted using PREP-Mt [31]. RNA-editing events were compared to other plant species by obtaining sequences from genbank with RNA-editing information and performing an alignment. Trans-membrane domains were predicted using TMHMM (v2.0) [32].

### PCR and Sanger confirmation

The contig graph was confirmed by PCR using 50 primer pairs (see Additional file 1). PCR for rearrangement sites was performed for each of the six varieties of rubber tree in both genomic and cDNA samples. Primers for suspected rearrangement sites were designed so that they flanked the suspected rearrangement site in non repetitive genomic DNA and additional primers were designed within the regions indicated as expressed by the RNA-seq mapping data (see Additional file 2).

### Phylogenetic tree

The phylogenetic tree was constructed using seven species (*Ricinus communis*, *Hevea brasiliensis*, *Carica papaya*, *Brassica napus*, *Raphanus sativus*, *Arabidopsis thaliana* and *Cycas taitungensis* as an outgroup). Gene sequences from each species for 21 conserved genes (*nad1*, *nad2*, *nad3*, *nad4*, *nad4L*, *nad5*, *nad6*, *nad7*, *nad9*, *cob*, *cox1*, *cox2*, *cox3*, *atp1*, *atp4*, *atp6*, *atp8*, *atp9*, *rps3*, *rps4*, *rps12*) were compared and a maximum likelihood tree was constructed using MEGA 5 with 1000 bootstrap replications [33].

## Results and discussion

### Mitochondrial sequence assembly

**Figure 1:**
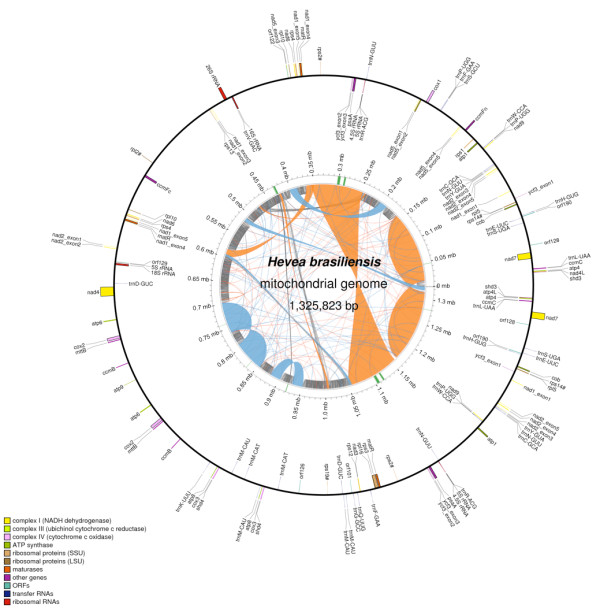
**Annotated representation of the rubber tree mitochondrial genome (outer circle).** Grey arches indicate the mapping of each pair of the Illumina paired-end sequence data (inner circle). Direct repeats are shown as blue arches and inverted repeats as orange arches (inner circle).

We assembled the mitochondrial sequence of the rubber tree variety BPM 24 from 454 sequence data into 37 contigs ranging from 101 bp to 147 kb in length with an N50 size of 51 kb (DDBJ: AP014526). Additional contigs were identified as mitochondrial sequence but, despite a similar GC content, had a sequence depth less than 10% of the other contigs. The low sequencing depth indicated that these contigs were nuclear encoded copies of mitochondrial sequence and these sequences were removed from the sequence assembly. Scaffolding the confirmed mitochondrial contigs produced a complex scaffold graph with 37 nodes and 49 edges that consisted of many small loops linked by repeat sequences. Twenty-one of these contigs had single 3’ and 5’ edges, 15 contigs had two 3’ and/or 5’ edges and one contig had three 3’ and 5’ edges resulting in thousands of possible configurations (see Additional file 1). To obtain the master circle mitochondrial sequence, the contig graph was traversed in such a way as to use all of the contigs at least once. We used Illumina paired-end data to confirm the scaffolds predicted by the 454 data and to correct homopolymer errors that are common in pyrosequencing data. We also performed PCR using a set of primers designed to the edges of each scaffold to confirm that these scaffolds did indeed join as shown by the presence of a PCR product (see Additional file 1). Several large segments of the mitochondrial genome were repeated in reverse orientation in the master circle resulting in duplication of approximately 350 kb in the master circle (Figure 1).

Several studies have attempted to identify the mechanism of plant mitochondrial DNA replication (for review see [34]) with evidence found for rolling circle replication and recombination mediated replication. However, the exact mechanism remains to be fully elucidated. In addition, studies using pulse field gel electrophoresis and electron microscopy failed to find a single circular strand of DNA, instead finding many smaller subgenomic circles and linear strands of DNA consistent with rolling circle replication products ([35] and references). Recent efforts to sequence plant mitochondrial genomes have also found evidence for subgenomic circles in the form of scaffold graphs of varying complexity [36-42], much like what we found in this study. In fact some studies have suggested that subgenomic circles are the native state of plant mitochondrial genomes [36,40], and must therefore be self replicating. With so many repeat sequences it is quite possible that an origin of replication sequence could exist multiple times in plant mitochondrial genomes allowing for independently replicating subgenomic circles to exist. Furthermore it is difficult to imagine how such diversity in mitochondrial genome size and subgenomic circle number could exist if there was but a single origin of replication with only the complete master circle able to segregate to dividing mitochondria. Such a mechanism would surely result in loss of non essential sequence and evolution towards more compact mitochondrial genomes as observed in mammalian mitochondrial genomes [43]. Thus our data adds to the growing body of evidence that plant mitochondrial genomes can consist of several independently replicating subgenomic circular DNA strands, a single circular DNA strand, or a mixture of both. Despite this, it is common practice to reconstruct a single ‘master circle’ DNA strand to represent the complete mitochondrial genome [36,39].

Plant mitochondrial genomes contain a large number of repeat sequences that enable homologous recombination to produce multiple subgenomic circles (for review see [44]). The rubber tree contig graph predicts a large number of possible subgenomic circles for the mitochondrial genome, suggesting mitochondrial mosaicism exits in rubber tree. The large number of possible subgenomic circles is facilitated by a large number of direct and inverted repeats. Over 34% of the mitochondrial genome consists of repeat and inverted repeat motifs. The most common repeat size was 20–40 bp with over 1800 instances accounting for 3.4% of the genome, followed by the 41–200 bp size range accounting for 1.16% of the genome (Table 1). Almost 30% of the genome consists of repeats larger than 200 bp, in many cases these large repeats formed individual contigs and are a contributing factor in the complexity of the contig graph (Table 1). Mapping reads from the Illumina paired-end run to the assembly gave a range of read depths that support variation in copy number of loci across the mitochondrial genome, the highest read depth was approximately three fold higher than the lowest depth (see Additional file 3). This suggests that the rubber tree subgenomic circles exist at different stoichiometries, similar to what was found in the cucumber mitochondrial genome [36]. Thus the rubber tree master circle is accurate in terms of sequence and contig orientation, but may under-represent the true copy number of each contig.

**Table 1:** Size, number and direction of repeat sequences in the rubber tree mitochondrial genome

**Repeat length (bp)**	**Number of repeats**	**% genome size**	**Direct**	**Inverted**
20-40	1837	3.44	962	875
41-60	160	0.59	88	72
61-80	42	0.21	21	21
81-100	14	0.09	9	5
101-200	27	0.26	13	14
>200	29	29.95	15	14
Total	2109	34.55	1108	1001

### Annotation of the mitochondrial genome

We identified 65 open reading frames that match known genes (Table 2). These gene annotations were supported by Illumina paired-end RNA-seq data from BPM 24 plus 5 additional clones (RRIM 600, RRIC 110, RRII 105, RRIT 251 and PB 235). These genes were primarily from the oxidative phosphorylation pathway (24 genes) and ribosome (12 genes). Fifty-four genes are encoded by a single exon and 11 genes are encoded across multiple exons. We found trans-splicing in three genes, *nad1*, *nad2* and *nad5*. Group II trans-splicing in these three nad genes is well documented and occurs in organelles of multiple plant species (for review see [45]). Each of the trans-spliced nad genes have large introns, up to several hundred kb, and at least one exon encoded on the opposite strand compared to the other exons for that gene, consistent with findings in other species [45]. In addition a gene transferred from the chloroplast, *ycf3*, would require trans-splicing to produce a functional mRNA in the rubber tree mitochondrion.

**Table 2:** Coding information of the rubber tree mitochondrial genome

**Gene function**	**Gene name**
Complex I	*(2x)nad1[5], (2x)nad2[5], nad3, nad4[4], (2x)nad4L, nad5[5], (2x)nad6, (2x)nad7[5], (2x)nad9*
Complex II	*(2x)shd3, (2x)shd4*
Complex III	*(2x)cob*
Complex IV	*cox1[2], (2x)cox2[2], cox3*
Complex V	*(2x)atp1, (2x)atp4, (2x)atp6, (2x)atp8, atp9*
Cytochrome-c biogenesis	*(2x)ccmB, (2x)ccmC, ccmFc[2], ccmFn*
SecY-independent transport	*(2x)mttB*
Ribosomal RNAs	*5S rRNA, 18S rRNA, 26S rRNA*
Ribosomal protein small subunit	*rps1, rps3[2], (2x)rps4, rps12, rps13*
Ribusomal protein large subunit	*(2x)rpl5, (2x)rpl10, rpl16*
Intron maturase	*(3x)matR*
Chloroplast transferred complete genes	*(2x)4.5S rRNA, (2x)5S rRNA, 16S rRNA, (2x)psaA, (2x)ycf3[3]*
Conserved Hypothetical genes	*orf101, orf122, orf126, (2x)orf128, orf129, (2x)orf190*
Transfer RNA	*(2x)trnC-GCA, trnD-GUC, trnD-GUC-cp, (2x)trnE-UUC, (2x)trnF-GAA, trnG-GCC, (2x)trnH-GUG-cp, trnK-UUU, (2x)trnL-UAA[2], (4x)trnM-CAU, (2x)trnM-CAU-cp, (4x)trnN-GUU-cp, trnP-UGG, (2x)trnP-UGG-cp, trnQ-UUG, (2x)trnR-ACG-cp, trnS-GCT, (2x)trnS-TGA, trnV-GAC-cp, (2x)trnW-CCA-cp, (2x)trnY-GTA*
Pseudogenes	*rpl2, (2x)rps2, (2x)rps14, rps19*
cp-derived gene fragment transfer	*16S rRNA, 23S rRNA, atpE, ndhF, (2x)psaB, (2x)psbC, (2x)rpoA, rps12_3end, (2x)ycf1, (2x)ycf15, (2x)ycf2, ycf68*
Lost gene (transferred to nucleus)	rps10[2]

*Note: Bracketed numbers indicate copy number of each gene, square brackets indicate number of exons, chloroplast derived tRNAs have -cp appended to them.

There were 19 tRNA genes identified, five of which occurred twice in the assembled mitochondrial master circle (Table 2). Seven of the tRNA genes plus 12 other genes are also found on the rubber tree chloroplast genome suggesting that they have been transferred from the chloroplast to the mitochondrial genome. It is unlikely to be chloroplast DNA contamination as these sequences differ from those in the chloroplast genome at multiple sites and there are sequencing reads extending from mitochondrial sequence to these transferred chloroplast fragments. Gene transfer from chloroplast to mitochondria is a common phenomenon in plants and the chloroplast copies that we found in the rubber tree mitochondria are largely consistent with previously identified chloroplast gene transfer events [46]. Exceptions include two genes, trnS-GGA and trnI-CAU, that have been transferred from chloroplast to mitochondria in a range of species but were not found in the rubber tree mitochondrial genome. A third gene, trnE-UUC, also known to have been transferred from chloroplast to mitochondria in other species, was found in the rubber tree mitochondria, but did not appear to be from the chloroplast DNA. *Ricinus communis* was similar to rubber tree in that it lacked the trnI-CAU gene and had a mitochondrial copy of trnE-UUC that was different to the chloroplast copy, but unlike rubber tree had a chloroplast copy of trnS-GGA. This shows that the chloroplast-derived trnS-GGA was lost to rubber tree after the split from *Ricinus* and that the either chloroplast-derived trnI-CAU and trnE-UUC genes were lost to the *Ricinus*/rubber tree clade or that the transfer occurred after this clade split from the other species.

**Figure 2:**
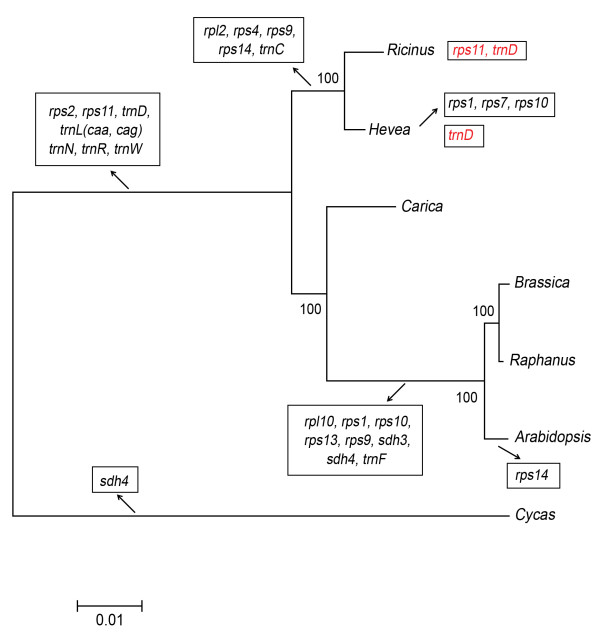
**Phylogenetic tree of seven mitochondrial genomes in plants.** Mitochondrial-like tRNA genes and protein-coding genes eliminated during evolution are presented in black boxes and genes that appear to have regained are presented in red boxes.

A phylogenetic tree constructed using seven species and 21 mitochondrial genes showed that rubber tree is most closely related to *Ricinus* (Figure 2). The number and type of mitochondrial genes can vary widely across species with gene loss and transfer to the nucleus occurring commonly [47]. Among the 7 species used we observed 26 different gene loss events (Figure 2). Interestingly, there were five events where genes that had previously been lost to a clade were regained by a species, two of these events were observed in *Ricinus communis* which regained *rps11* and t*rnD-GUC* (as previously reported [48]). Rubber tree also regained t*rnD-GUC* suggesting the event took place before the split from *Ricinus communis*. In each of these cases the regained gene was lost quite far back in the clade making it unlikely to be an error in the phylogenetic tree construction. It is interesting to note that rubber tree also gained a chloroplast copy of t*rnD-GUC* (in addition to the mitochondrial copy mentioned above) suggesting that this tRNA might play an important role in the rubber tree mitochondria.

### RNA editing

We identified cases of RNA editing by mapping the RNA-seq data to the assembled mitochondrial genome. This identified 224 cases among the six samples where the RNA-seq base was a thymine while the genomic base was a cytosine (see Additional file 4). Out of these sites 184 were also predicted to be RNA editing sites based on information from other species and 199 changed an amino acid. The most common amino acid changes were S to L with 52 events, P to L with 42 events and S to F with 29 events. RNA-editing was compared across 29 species, where data was available, and found to be highly conserved across all species (see Additional file 4). We found 8 cases where a C was edited to a U in rubber tree, in genes *atp4*, *cox1*, *cox2*, *matR*, *nad1*, *nad2* and *nad7*, but remained an unedited C in all other species (see Additional file 4). There was a single case, in *cytochrome c biogenesis factor C* (*ccmFC*), where RNA-editing was required to produce a stop codon, this RNA-editing site was highly conserved among many species. The most heavily edited gene was *cytochrome c oxidase subunit 2* (*cox2*) with 15 RNA-editing events in 783 bp of sequence.

Two cases were found where BPM 24 showed a lack of RNA-editing at a location where all the other rubber tree samples showed either RNA-editing or a variant, one was in *succinate dehydrogenase subunit 3* (*sdh3*) and the other was in *maturase-R* (*matR*). The *sdh3* RNA-editing event did not change an amino acid and was not found in any other species so is unlikely to have a significant effect. The RNA-editing event in *matR* changes a hystidine residue (positively charged) to a tyrosine residue (hydrophobic with a negative dipole) which may be required for correct protein folding, but is not in a functional motif. The RNA-editing in *matR* was observed in four other species while six other species showed a T variant. There were only two cases where a species with RNA-editing information had the same base (C) as BPM 24 at this locus, however, this could represent incomplete information rather than a lack of RNA-editing. Mitochondrial encoded maturases have been found to be required for the proper splicing of some group II introns in Arabidopsis with mutations resulting in retarded growth and developmental phenotypes [49,50]. However, the specific function of *matR* remains unknown so whether this lack of RNA-editing plays a role in the CMS phenotype or not is unclear.

### Male sterility

Since it is known that BPM 24 male sterility is mitochondrially inherited there must be a mitochondrial change that causes CMS [21]. With this in mind we compared contigs from the published rubber tree genome, variety RRIM 600 [19], that were identified as mitochondrial sequence against the BPM 24 mitochondrial contigs and scaffold assembly. This identified 11 contigs from the published genome that blast to the BPM 24 mitochondrial genome in a disjointed manner (Table 3) indicating a rearrangement in BPM 24 compared to the published rubber tree sequence. Five of these potential rearrangement sites that were within 1 kb of a gene were checked by PCR in six varieties of rubber tree. Two sites were found to exist in both the arrangement represented by the published genome and the arrangement represented by our assembly in all varieties, confirming the variation in mitochondrial subcircles identified in the scaffold graph. Three of these regions showed variation amongst the six varieties tested (see Additional file 2). One region was present as the published rubber tree (RRIM 600) arrangement in varieties BPM 24, RRII 105 and RRIM 600 but not in RRIC 110, RRIT 251 or PB 235, however all varieties had the BPM 24 arrangement for this region. Two rearrangements were unique to BPM 24 and in both cases were close to or within coding sequence making them good candidates for the cause of CMS in BPM 24.

**Table 3:** Blast result of published contigs (RRIM 600) vs BPM 24 master circle showing 11 rearranged contigs

**Query id**^**+**^	**% identity**	**Align length**	q. start^*^	q. end^*^	**24 start**^**±**^	**24 end**^**±**^	**e-value**
AJJZ011005169.1	85.93	135	549	683	*338566*	*338700*	7e-27
	96.74	276	937	1211	854221	854495	4e-130
AJJZ011005166.1	98.79	1077	241	1317	21535	20459	0
	96	200	1	198	*1017254*	*1017055*	4e-87
AJJZ010488272.1	100	144	182	325	115093	115236	3e-78
	96.76	185	51	235	159007	159191	2e-88
	100	117	1	117	221602	221486	4e-62
*AJJZ010386739.1*	98.62	217	186	401	*179706*	*179922*	4e-112
	98.3	176	1	176	*1031613*	*1031788*	5e-90
AJJZ010369193.1	99.35	308	1	308	423994	424301	4e-171
	100	203	306	508	663278	663076	3e-113
AJJZ010233339.1	94.44	162	567	728	202461	202622	1e-66
	82.95	88	415	502	*741897*	*741984*	3e-08
	86.92	107	414	520	*882085*	*882190*	1e-19
AJJZ010228768.1	92.62	149	2483	2631	15228	15376	7e-54
	84.71	327	2631	2934	15404	15728	6e-67
	82.71	451	1622	2060	357491	357055	9e-66
	87.14	770	831	1596	*358263*	*357495*	0
*AJJZ010174367.1*	91.99	1548	5871	7413	*156744*	*158290*	0
	92.52	1096	9138	10223	529298	528203	0
	89.38	885	10204	11068	894891	895774	0
	94.33	141	11065	11203	895680	895820	3e-40
AJJZ010143874.1	99.97	7965	1	7965	38753	30789	0
	100	1864	7733	9596	*127434*	*125571*	0
*AJJZ010142287.1*	99.18	612	1	612	*338444*	*339054*	0
	100	585	512	1096	854280	854864	0
AJJZ010039172.1	98.4	187	80	266	17960	17774	6e-97
	99.6	248	266	513	*32045*	*32292*	1e-137

^**+**^Italics indicates that the rearrangement or part of it has been confirmed by PCR.

^*^Start and end of query sequence alignment location.

^**±**^Start and end of master circle sequence alignment location. Bold indicates a gene is located within 1 kb.

**Figure 3:**
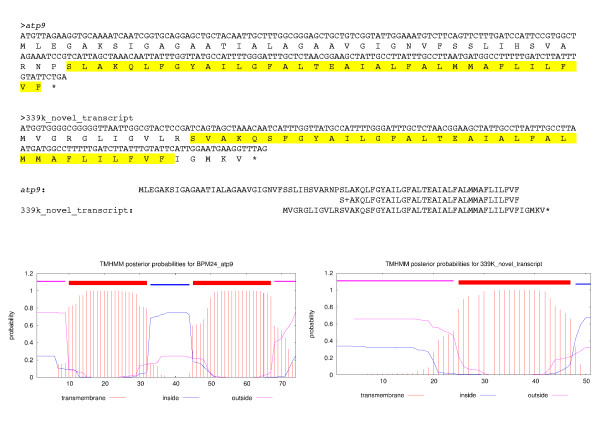
**Comparison of the RNA and amino acid sequences of rubber tree *apt9 *** **with the novel transcript and the predicted trans-membrane structures of each.**

The first rearrangement unique to BPM 24 was identified by the 1096 bp published RRIM 600 contig AJJZ010142287.1. The first 612 bp of this contig maps to 338444–339054 bp in the BPM 24 master circle and the last 585 bp of the contig maps to 854280–854864 bp in the master circle (Table 3). In the master circle the break point sections share a 101 bp repeat sequence with 1 mismatch between the two sequences which may be the footprint of a homologous recombination (see Additional file 2). Interestingly, the 338444–339054 bp region has RNA-seq data supporting expression at this region but no gene is annotated in the assembly and BPM 24 has an extra 240 bp of sequence that none of the other varieties share. This additional sequence was checked by PCR using primers flanking the break site and was confirmed to be present only in BPM 24 both in genomic DNA and cDNA (see Additional file 2). The sequence has an open reading frame encoding 51 amino acids, 33 of which are identical to the tail end of *ATPase subunit 9* (*atp9*) plus 5 additional amino acids. A full copy of *atp9* is annotated as occurring at 760961–761254 bp and the RNA-seq data at this region is the same in all six rubber tree varieties. The rubber tree *atp9* has a transmembrane region near the amino-terminus and another near the carboxyl-terminus of the protein (Figure 3). The novel transcript incorporates the entire carboxyl-terminus transmembrane domain (Figure 3) which may allow it to compete with the full *atp9* gene in the ATP synthase complex. It is likely that a recombination occurred in a subgenomic circle containing a copy of *atp9* in BPM 24 resulting in the novel transcript at this recombination site. The fact that the other rubber tree varieties also show expression at this location is likely a mapping artefact where RNA-seq reads from the normal gene are mapping to this section, this is supported by seven sequence variants between BPM 24 and the other rubber tree varieties in the RNA-seq reads that map to this section (see Additional file 2). This shows that the sequence identified at the 339 kb region is an additional transcript rather than a mutant form of *atp9* for BPM 24, similar to what has been identified in other CMS plants [51,52].

The second rearrangement was identified by the 11561 bp contig AJJZ010174367.1 from RRIM 600 that maps to four separate sections of the master circle (Table 3). The most interesting of these is Section 5871–7413 bp of the published RRIM 600 contig that matches 156744–158290 bp of the master circle, and similar to the first rearrangement, has a 29 bp repeat sequence near the break site and RNA-seq data supporting expression with 128 bp of extra sequence unique to BPM 24. Using PCR primers that flanked the rearrangement site within the expressed section we found that the published contig sequence is present in all varieties and the rearranged sequence is found and expressed only in BPM 24 (see Additional file 2). The expressed sequence at this site matches *ATPase subunit 1* (*atp1*) which is annotated at 156716–158245 bp on the master circle negative strand, placing one of the break points 45 bp before the start codon of *atp1* and the other 28 bp before the end of *atp1*. All varieties had RNA-seq data consistent with a complete and functional copy of *atp1* with the extra sequence in BPM 24 occurring after the stop codon suggesting that this variant is unlikely to affect the protein product.

A novel or fusion transcript is a common occurrence in CMS plants and often involves a portion of, or is near an ATP synthase subunit gene [53]. In total, nine cases of a novel transcript containing part of an ATP synthase gene have been found in CMS plants which are not found in control plants: *atp1* in eggplant [54]; *atp6* in maize [9], *Brassica tournefortii*[13], wheat [55] and chilli [56]; *atp8* in sunflower [57]; *atp9* in petunia [10], rapeseed [58] and sorghum [59]. In addition, disruptions to the ATP synthase complex, not featuring fusion transcripts, in plant mitochondria have been associated with CMS in chilli [60], *Oryza rufipogon*[61], *Arabidopsis thaliana*[62], wheat [63], maize [64] and tobacco [65]. Since the observed novel transcript in rubber tree is both a novel fustion transcript and includes a portion of an ATP synthase subunit, typical of a CMS causing change, it is highly likely to be the cause of CMS in rubber tree. While it may be difficult to imagine how disruption to such a fundamental function as energy production could result in male sterility but not affect any other cell type or developmental process, there is evidence that some cell types are more sensitive than others to perturbation of mitochondrial efficiency. A prime example of this is Leber’s hereditary optic neuropathy in human where a mitochondrial mutation in an oxidative phosphorylation gene only affects retinal ganglion cells [66]. The most common finding in CMS plants is an additional transcript that contains part of an ATP synthase gene and is thus a gain of function change which explains how it can be specific to anthers. Anther development has a high energy demand and mitochondria undergo rapid expansion in copy number early during anther growth, increasing by as much as 40 fold per cell [67]. Anthers of CMS maize begin to breakdown shortly after this mitochondrial expansion suggesting a link between the two processes [67]. Indeed, cell death of sunflower CMS anthers has been associated with the release of mitochondrial cytochrome c oxidase into the cytosol [68], which is an activation signal for apoptosis-like cell death [69]. This particular form of sunflower CMS is caused by a novel transcript with *atp8*-like sequence and has been shown to have reduced ATP hydrolysis function [57]. Thus novel transcripts that encode part of an ATP synthase gene, such as the one identified in BPM 24, cause CMS, at least in some cases, by slightly reducing the ATP synthase complex activity to a point where mitochondria cannot generate sufficient energy for the highly energy reliant anthers resulting in mitochondria mediated apoptosis-like cell death.

## Conclusion

We have reconstructed the mitochondrial sequence of rubber tree clone BPM 24 and identified coding sequences and repeat elements. We then used the published contigs from RRIM 600 to identify rearrangements in BPM 24 that result in fusion transcripts for *atp1* and *atp9*, with the *atp9* fusion transcript likely reducing the efficiency of ATP production and resulting in cytoplasmic male sterility. Since BPM 24 is the offspring of the variety GT 1, we have indirectly identified the cause of CMS in GT 1 also. The exhaustive nature of this search approach rules out any other cause for the observed CMS in BPM 24 rubber tree and corroborates findings by other groups, often using less exhaustive search approaches, that novel fusion transcripts of ATP synthase genes can cause CMS.

### Availability of supporting data

Rubber tree mitochondrial genome master circle: DDBJ: AP014526.

Rubber tree mitochondrial genome raw reads: DDBJ: DRA001347.

## Competing interests

The authors declare that they have no competing interests.

## Authors’ contributions

KT, STr and STa conceived the study. JRS, PA, CS and PU performed the data analysis. DS, TY and SP performed the lab work. JRS and STa drafted the manuscript. All authors read and approved the final manuscript.

## Supplementary Material

Additional file 1 Primers and products showing rearrangement events and novel transcripts in the CMS rubber tree variety BPM 24. Click here for file

Additional file 2 Scaffold graph, primers and primer products used to confirm the scaffold order in the rubber tree master circle genome. Click here for file

Additional file 3 Mapping results of the Illumina genomic data against the rubber tree master circle genome. Click here for file

Additional file 4 Table of RNA editing events in the rubber tree RNA-seq data and comparison of RNA editing events to RNA editing event information from genbank. Click here for file
