# Supplementary material for: Assembly and analysis of a male sterile rubber tree mitochondrial genome reveals DNA rearrangement events and a novel transcript
Source: BMC Plant Biol. 2014 Feb 10;14:45. doi: 10.1186/1471-2229-14-45 (PMC3925788; doi:10.1186/1471-2229-14-45)
Supplement: Additional file 1 — Primers and products showing rearrangement events and novel transcripts in the CMS rubber tree variety BPM 24. [file 1471-2229-14-45-S1.pdf]

Supplementary file 1

We designed primers to each of the contig ends (table S1.1), then performed PCR using these primers to confirm each of the contig joins that make up the master circle (figure S1.1, figure S1.2 and figure S1.3). The master circle was constructed using the contig graph (figure S1.4) such that all contigs were covered at least once.

Table S1.1: Primers used to confirm contig joins in the master circle

**Primer names**	**Primer pair number**	**Sequence**	**Tm**	**Expected amplicon size (bp)**	**Connection**
mtHv_ct1_L	1	CTTTCCCTGAGGTTGAAGTTG	55	1124	c00001-c00002
mtHv_ct2_R		TTCTATGGGAAATTGGAAACG			
mtHv_ct2_L	2	AATTGCACCAGTCTTTCATGC	55	980	c00002-c00800
mtHv_ct800_R		GTTCATTTCAATCGTGCCAGT			
mtHv_ct800_L	3	GAGGATTTACAGTCCCAGATGC	55	381	c00800-c00003
mtHv_ct3_R		ACTTCCATGAGCAGAAAGAGC			
mtHv_ct3_L	4	GTGAGGCAATGAAGAATTGGA	55	279	c00003-c00038
mtHv_ct38_R		AGGAGATGTGCCCCATAAAGA			
mtHv_ct38_L	5	AAGGGCTTCCTATTCCTCCAT	55	576	c00038-c00021
mtHv_ct21_R		GCCTTTGCTTTTCTAAGGACA			
mtHv_ct21_L	6	CCAGAAAAGAAAGGTCGACTG	55	488	c00021-c01565
mtHv_ct1565_R		ATGCCCCATTGACTTCTCTCT			
mtHv_ct1565_L	7	TAAACTAAGCCGAAGCACAGG	55	328	c01565-c00352
mtHv_ct352_R		CCGGAAGTACGCCATAAAGTA			
mtHv_ct352_L	8	TCCCTTATCCAGTTGATGCAG	55	193	c00352-c00020
mtHv_ct20_R		GTAATGCATGAAGCGGAGTCT			
mtHv_ct20_L	9	GAGCTTATCAACGACACTCTGG	55	326	c00020-c00400
mtHv_ct400_R		CATGTGTTAGGTTCTCGTTGC			
mtHv_ct400_L	10	CAAGATCCGAACAACAAGGAG	55	201	c00400-c00086
mtHv_ct86_R		AAGTCATGAGCGATAGCGAAG			
mtHv_ct86_L	11	TTCATTCGGCCCTGTAGTTAG	55	1066	c00086-c00030
mtHv_ct30_R		TCTGGATGGCTCTCATAATCC			

mtHv_ct30_L<br>mtHv_ct38_R	12	TACAGAGGACAGAGGTGAAGGA<br>AGGAGATGTGCCCCATAAAGA	55	287	c00030-c00038
mtHv_ct38_L<br>mtHv_ct37_R	13	AAGGGCTTCCTATTCCTCCAT<br>GGATACCAAGGATGGAGACAA	55	358	c00038-c00037
mtHv_ct37_L<br>mtHv_ct9453_R	14	TAGACTCCAAACCTGCCAATC<br>TTAGCAAGGTAAGCCCCATCT	55	670	c00037-c09453
mtHv_ct9453_L<br>mtHv_ct493_R	15	AAAGAAGGCAGGGAATCACAG<br>CAACCGGCCATAGTGAACTTA	55	258	c09453-c00493
mtHv_ct493_L<br>mtHv_ct19_R	16	TCTCAGTCAAATCGAGGGTTG<br>GACACATCCCATTGCACTGAT	55	252	c00493-c00019
mtHv_ct19_L<br>mtHv_ct1565_L	17	GCATCGGATAGAAAGAGATCG<br>TAAACTAAGCCGAAGCACAGG	55	574	c00019-c01565
mtHv_ct1565_R<br>mtHv_ct18_R	18	ATGCCCCATTGACTTCTCTCT<br>TCATACCTTCCCTTCCTTGCT	55	554	c01565-c00018
mtHv_ct18_L<br>mtHv_ct17_R	19	GACCGGTTTTCCTTTGCTATC<br>TCCCTTCTGAAGAGTCATTGC	55	248	c00018-c00017
mtHv_ct17_L<br>mtHv_ct11_R	20	TGACAAGCGGATGAGTTTTCT<br>TGATAAGTAGGCGCTGCAGTT	55	435	c00017-c00011
mtHv_ct11_L<br>mtHv_ct86_L	21	GGACCAAGAAGTGTTTGAAGG<br>TTCATTCGGCCCTGTAGTTAG	55	983	c00011-c00086
mtHv_ct400_R<br>mtHv_ct4_R	22	CATGTGTTAGGTTCTCGTTGC<br>TGATTAGTTCAAGACTGGACAGG	55	257	c00400-c00004
mtHv_ct4_L<br>mtHv_ct5_R	23	TCCCAACATCATTCTCAAACA<br>AGCCGTCTAAAGAAAGGCGTA	55	970	c00004-c00005
mtHv_ct5_L<br>mtHv_ct6_R	24	TCAAGGAGAGTTTGACCATCC<br>CCGATTGGTTGTGAGTGTCTT	55	924	c00005-c00006
mtHv_ct6_L<br>mtHv_ct12_R	25	CTCTCGGATTGTTGAAGAAGG<br>TGGAGATTTGTAGCATCATTCA	55	1313	c00006-c00012

mtHv_ct12_L<br>mtHv_ct25668_R	26	TTGGACTGTGAACGAATTTCA<br>GACGCTTTCTCGAATCTTCAC	55	666	c00012-c25668
mtHv_ct25668_L<br>mtHv_ct22_R	27	GGCTAGAAATCCGTCATTAGC<br>TCTTAGTCCTACCGCTTTCCA	55	247	c25668-c00022
mtHv_ct22_L<br>mtHv_ct12_R	28	GCTCAAGCCTATTCCATTTCC<br>TGGAGATTTGTAGCATCATTCA	55	876	c00022-c00012
mtHv_ct25668_L<br>mtHv_ct23_R	29	GGCTAGAAATCCGTCATTAGC<br>AAGTGAAAGAAGCCCACAGAC	55	234	c25668-c00023
mtHv_ct23_L<br>mtHv_ct29_R	30	TTGAATCTGCTGACAGTGAGG<br>GATAATGATGAGCCCCGTGTA	55	579	c00023-c00029
mtHv_ct29_L<br>mtHv_ct24_R	31	TAACTCCGGCTGCAAGTCTAA<br>TAAGCAGGAGAATCACGAAGG	55	331	c00029-c00024
mtHv_ct24_L<br>mtHv_ct29_R	32	CTGAACGTGCCAAGGAAATAG<br>GATAATGATGAGCCCCGTGTA	55	327	c00024-c00029
mtHv_ct29_L<br>mtHv_ct15_R	33	TAACTCCGGCTGCAAGTCTAA<br>CTTGCACCAATCAAATCCACT	55	238	c00029-c00015
mtHv_ct15_L<br>mtHv_ct493_L	34	AGACTGGTCTTGCGAAGATGT<br>TCTCAGTCAAATCGAGGGTTG	55	362	c00015-c00493
mtHv_ct493_R<br>mtHv_ct16_R	35	CAACCGGCCATAGTGAACTTA<br>TATTAGGGATTGGGGATCACA	55	235	c00493-c00016
mtHv_ct16_L<br>mtHv_ct400_L	36	CAGATCAAGTCTGACCGAAATC<br>CAAGATCCGAACAACAAGGAG	55	419	c00016-c00400
mtHv_ct20_R<br>mtHv_ct279_R	37	GTAATGCATGAAGCGGAGTCT<br>CCCGGCCTTAAAAGACAGAT	55	189	c00020-c00279
mtHv_ct279_L<br>mtHv_ct800_L	38	TGCCTACGATCCTTTCTGACT<br>GAGGATTTACAGTCCCAGATGC	55	354	c00279-c00800
mtHv_ct2_R<br>mtHv_ct333_R	39	TTCTATGGGAAATTGGAAACG<br>GAAAGAGAAGGTTGCCCAGTT	55	1093	c00002-c00333

mtHv_ct333_L<br>mtHv_ct17_R	40	AAGAAAGCTTCTGCCCTCTTG<br>TCCCTTCTGAAGAGTCATTGC	55	296	c00333-c00017
mtHv_ct17_L<br>mtHv_ct1_R	41	TGACAAGCGGATGAGTTTTCT<br>CATGGATTCCTACGAAAGAGG	55	439	c00017-c00001

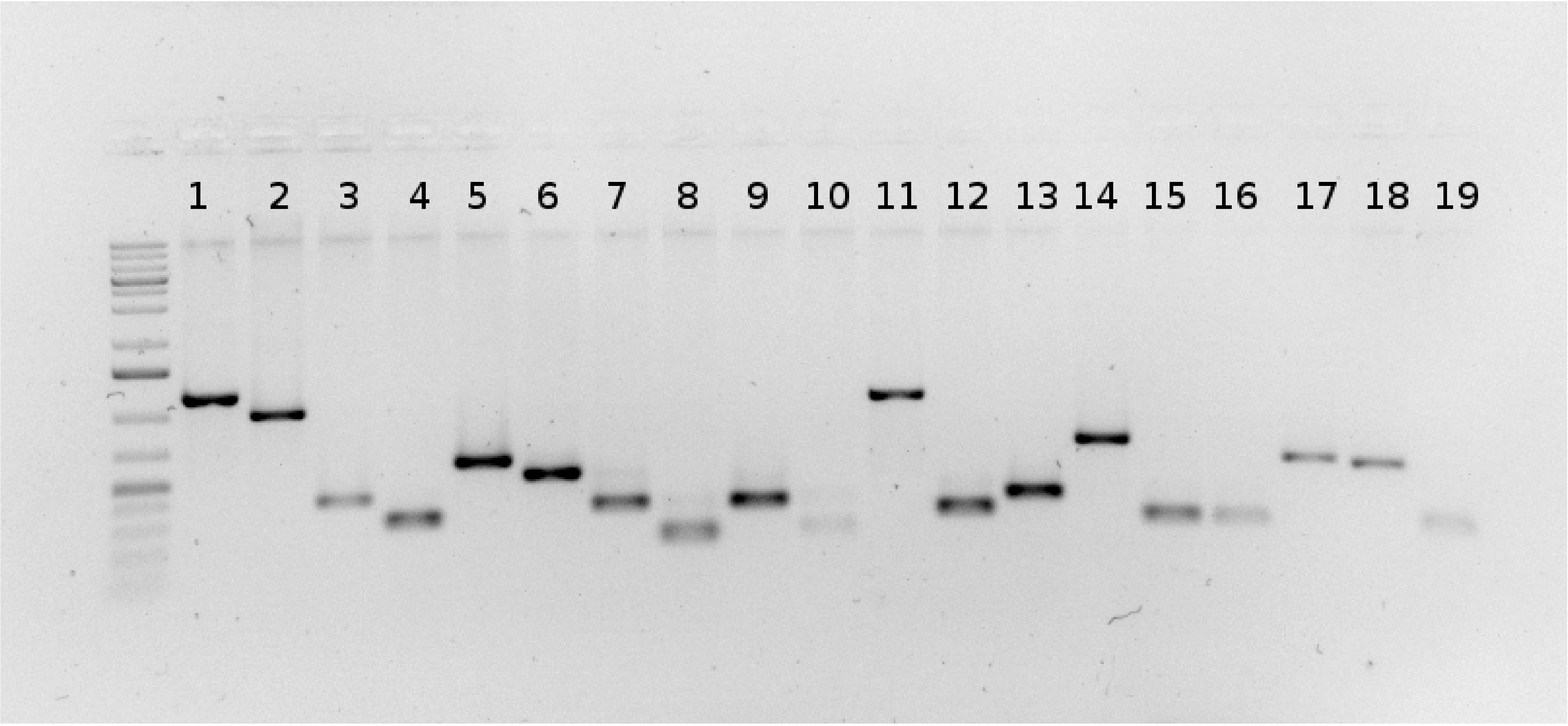

Figure S1.1: PCR products confirming master circle contig joins of primer pairs 1-19 from table S1.1. The DNA ladder is the Fermentas 1 kb plus generuler.

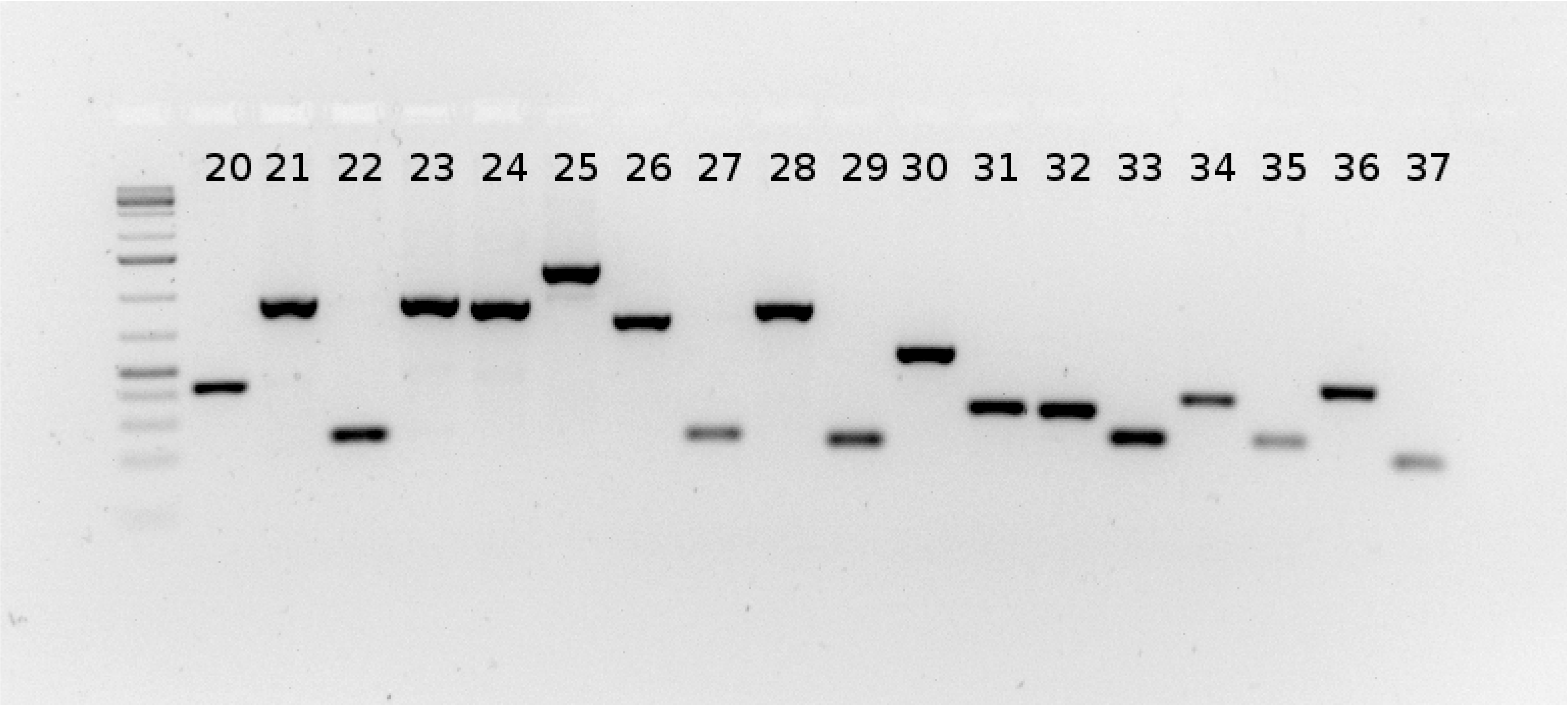

Figure S1.2: PCR products confirming master circle contig joins of primer pairs 20-37 from table S1.1. The DNA ladder is the Fermentas 1 kb plus generuler.

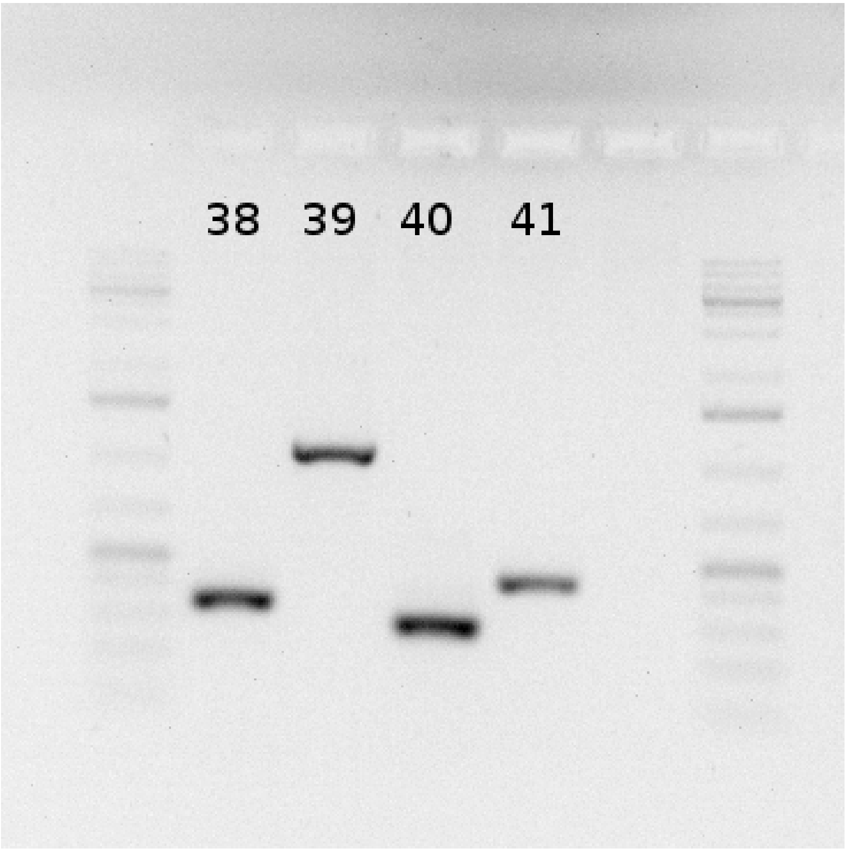

Figure S1.3: PCR products confirming master circle contig joins of primer pairs 38-41 from table S1.1. The DNA ladder is the Fermentas 1 kb plus generuler.

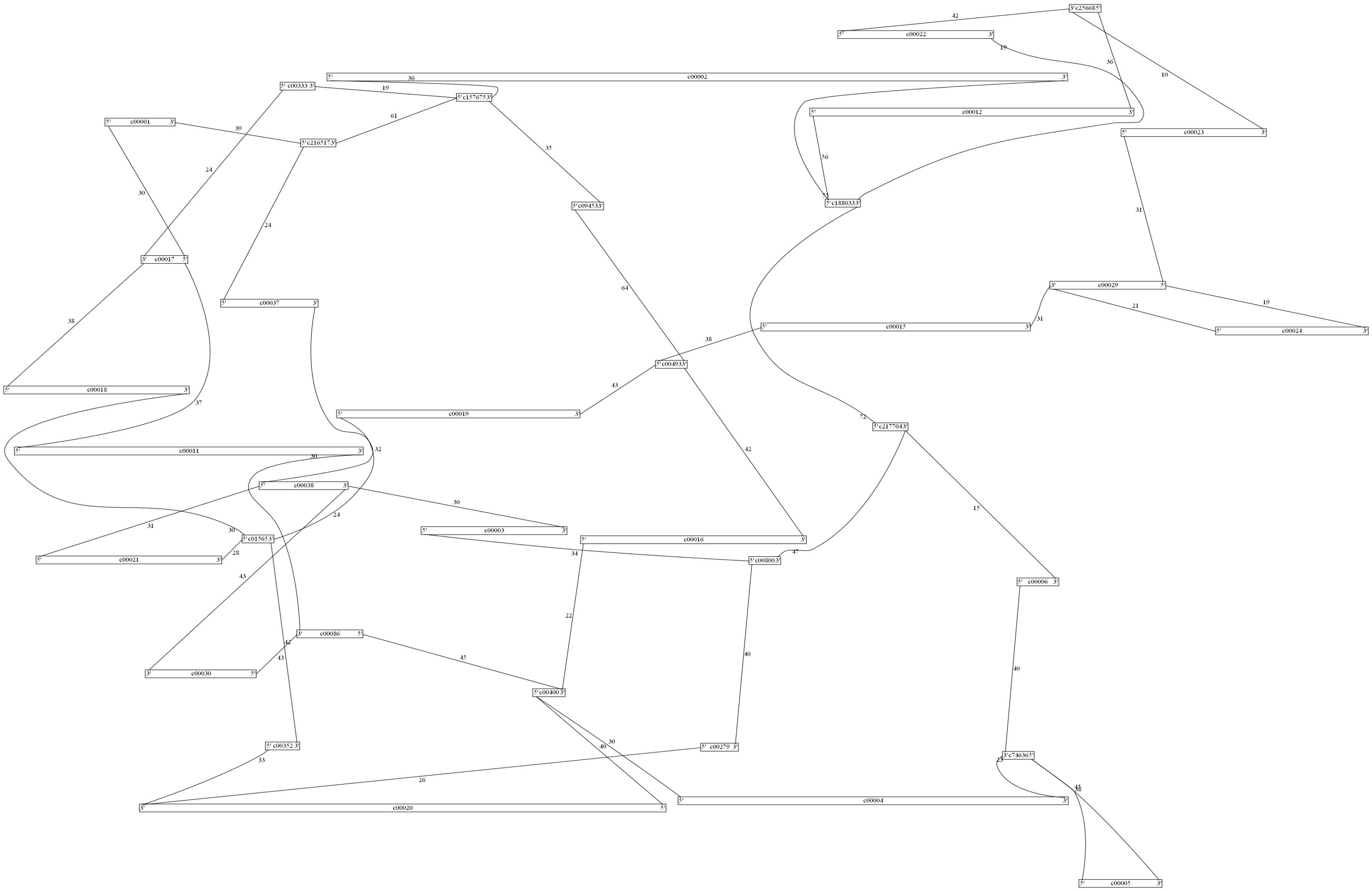

Figure S1.4: Scaffold graph showing 5` and 3` edges. The numbers indicate the number of supporting reads for each contig join.
