# Supplementary material for: Assembly and analysis of a male sterile rubber tree mitochondrial genome reveals DNA rearrangement events and a novel transcript
Source: BMC Plant Biol. 2014 Feb 10;14:45. doi: 10.1186/1471-2229-14-45 (PMC3925788; doi:10.1186/1471-2229-14-45)
Supplement: Additional file 2 — Scaffold graph, primers and primer products used to confirm the scaffold order in the rubber tree master circle genome. [file 1471-2229-14-45-S2.pdf]

Supplementary file 4

Blasting the published contigs against the rubber tree mitochondrial master circle identified 11 contigs that appeared to have been rearranged in the master circle. We developed sets of primers to check these loci at rearrangements that occurred in or within 1 kb of a gene. Sets of three or four primers were developed for each break site tested such that one primer was common to both arrangement types and one primer was unique to each of the arrangement types (figure S4.1, table S4.1). We tested one or more break sites from five different published contigs using the primer combinations in table S4.2 and in most cases found that both the master circle arrangement and the published contig arrangement existed in all samples (figure S4.2, figure S4.3, figure S4.4), most likely as different forms of subgenomic circles. In some cases the sequence surrounding a break site was too repetitive to design unique primers and these cases are likely to represent different subgenomic circles also. We found that two rearrangements were unique to BPM 24 (Figure S4.2 lane 7 and figure 4.4 lanes 13 and 14) where only BPM24 had the master circle arrangement while all other samples had only the published contig arrangement. In addition we found that one of the published contig arrangements was present only in samples BPM 24, RRIC 105 and RRIM 600 while all samples had the master circle arrangement for this locus. The two rearrangements that were unique to BPM 24 also had RNA-seq data supporting expression at these loci (figure S4.5 And figure S4.6). Additional primers (Table S4.3) were required to test the two confirmed rearrangements as one primer from each pair of the existing primers (table S4.1) were outside of the expressed sequence. These primers were checked in both genomic and cDNA for each of the six rubber tree varieties and gave a product in each case only for BPM 24 (Figure S4.7 and figure S4.8).

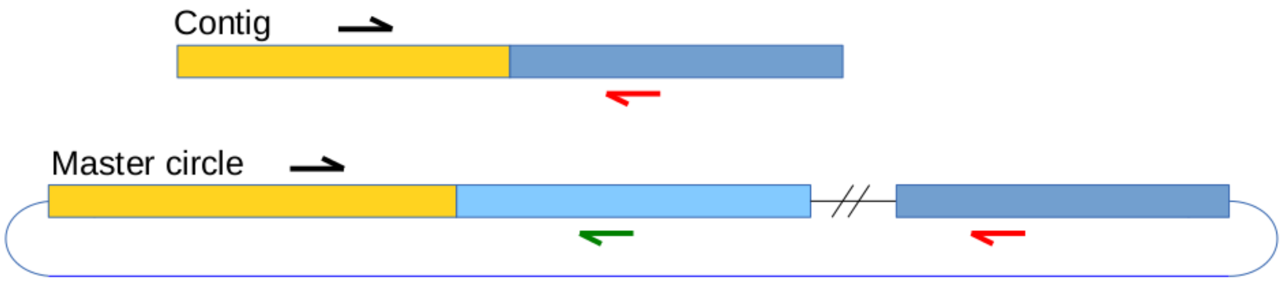

Figure S4.1: Schematic of primer design for each break point, indicated by coloured rectangles. The double cut line indicates a gap too large for a PCR product to form.

Table S4.1: Primers used to check suspect rearrangements between published contigs and the BPM 24 master circle

**Primer***	**Sequence**	**Pair**
AJJZ010039172_F	taaaagaatgaatgggaagcaggtc	AJJZ010039172_R
AJJZ010039172_R	ctgaatgaagtcctccgtttctttc	AJJZ010039172_F
Master_17960-17774_R	taatacatgatgccattcccgattc	AJJZ010039172_F
Master_32045-32292_F	gctttatatctccgacacacaagg	AJJZ010039172_R
AJJZ010143874_F	ctaagcccgttctattgcataacc	AJJZ010143874_R
AJJZ010143874_R	gatcttcgtcaaatacccgactttg	AJJZ010143874_F
Master_38753-30789_R	cgtaacgtaacaactagaaagctgg	AJJZ010143874_F
Master_127434-125571_F	cgggtttatggattggattcagtc	AJJZ010143874_R
AJJZ010174367_F	gtgaataagataagattaggtgctttgg	AJJZ010174367_R
AJJZ010174367_R	atcgaatgccactagacagaatttc	AJJZ010174367_F
Master_156744-158290_F	cagtagcattgatttgagttttaccc	AJJZ010174367_R
AJJZ010386739_F	aactattggaagcaaatggaaaagg	AJJZ010386739_R
AJJZ010386739_R	aacgatcaatagtagtttccaaccc	AJJZ010386739_F
Master_179706-179922_F	tacgtggcaagagaggtttattatc	AJJZ010386739_R
Master_1031613-1031788_R	tatcgaaataataagcatgtggggc	AJJZ010386739_F
AJJZ010142287_F	ttttgaaatgagcgcacctacg	AJJZ010142287_R
AJJZ010142287_R	atggcgtagaacttccaaatgg	AJJZ010142287_F
Master_338444-339054_F	tgattgagagcgattgaacagc	AJJZ010142287_R
Master_854280-854864_R	aggccaagcattaagaaagagg	AJJZ010142287_F

* Primers are named according to either the published contig they were designed to or the location in the master circle to which the published contig maps.

Table S4.2: Primer combinations tested

Lane Group	Left Primer	Right Primer
1	AJJZ010039172_F	AJJZ010039172_R
2	AJJZ010039172_F	Master_17960-17774_R
3	Master_32045-32292_F	AJJZ010039172_R
4	AJJZ010143874_F	AJJZ010143874_R
5	Master_127434-125571_F	AJJZ010143874_R
6	AJJZ010143874_F	Master_38753-30789_R
7	Master_156744-158290_F	AJJZ010174367_R
8	AJJZ010174367_F	AJJZ010174367_R
9	AJJZ010386739_F	AJJZ010386739_R
10	Master_179706-179922_F	AJJZ010386739_R
11	AJJZ010386739_F	Master_1031613-1031788_R
12	AJJZ010142287_F	AJJZ010142287_R
13	Master_338444-339054_F	AJJZ010142287_R
14	AJJZ010142287_F	Master_854280-854864_R

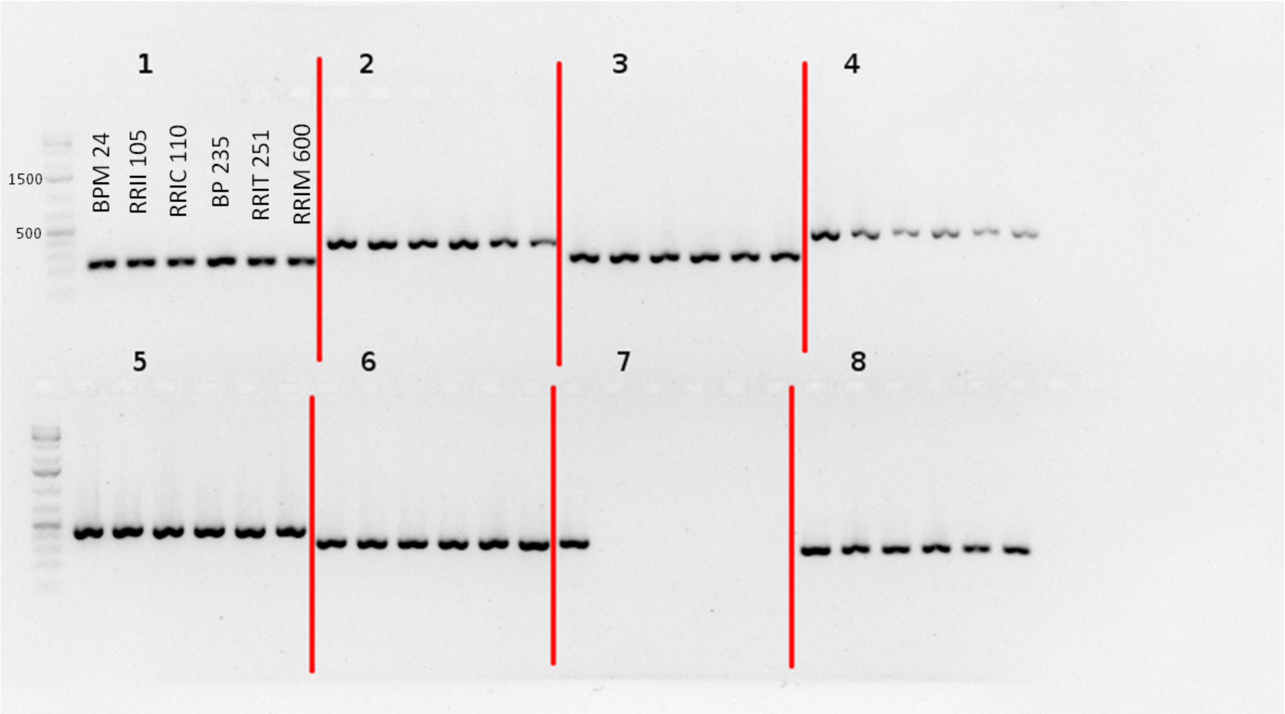

Figure S4.2: Products of primer combinations 1-8 from table S4.2 for rubber tree varieties BPM 24, RRII 105, RRIC 110, PB 235, RRIT 251 and RRIM 600 (respectively). The DNA ladder is the Fermentas 1 kb plus generuler.

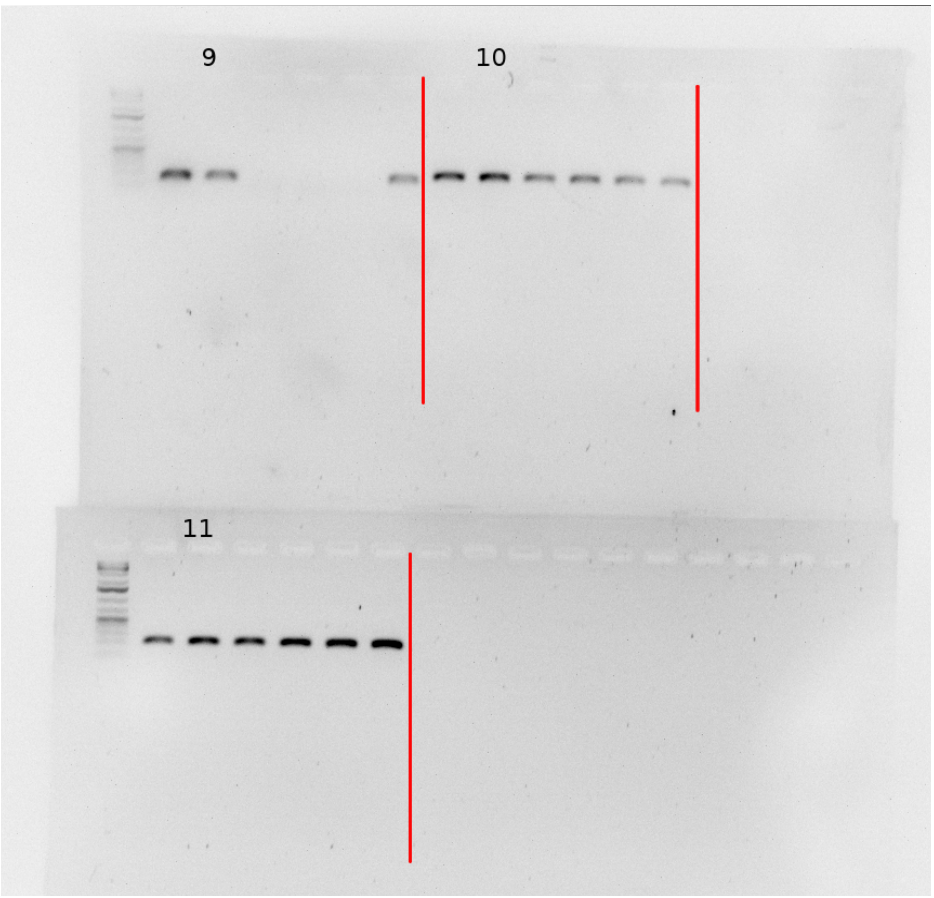

Figure S4.3: Products of primer combinations 9-11 from table S4.2 for rubber tree varieties BPM 24, RRII 105, RRIC 110, PB 235, RRIT 251 and RRIM 600 (respectively). The DNA ladder is the Fermentas 1 kb plus generuler.

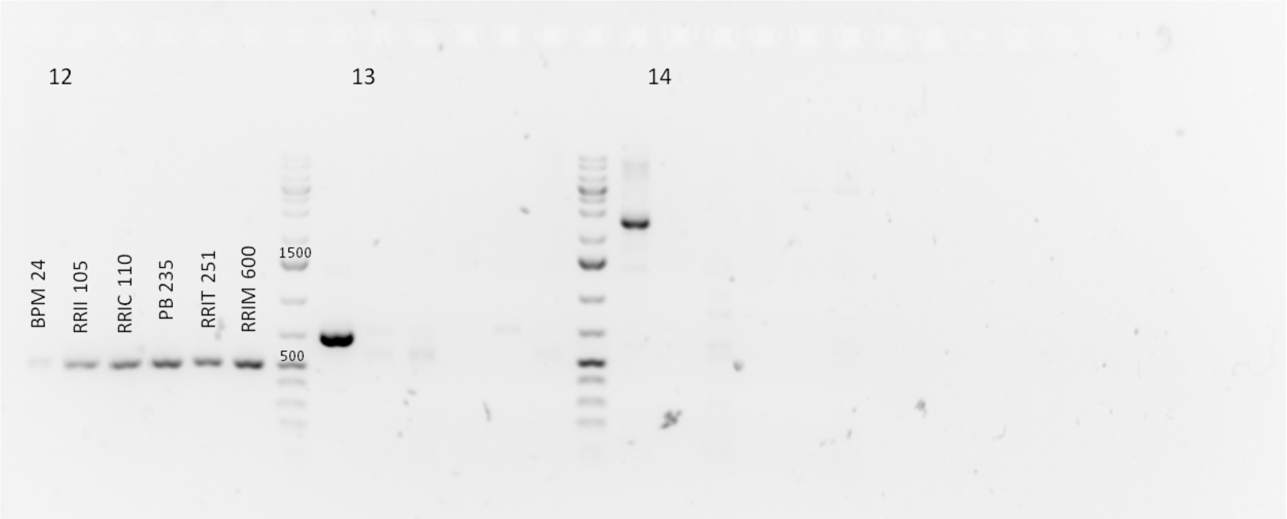

Figure S4.4: Products of primer combinations 12-14 from table S4.2 for rubber tree varieties BPM 24, RRII 105, RRIC 110, PB 235, RRIT 251 and RRIM 600 (respectively). The DNA ladder is the Fermentas 1 kb plus generuler.

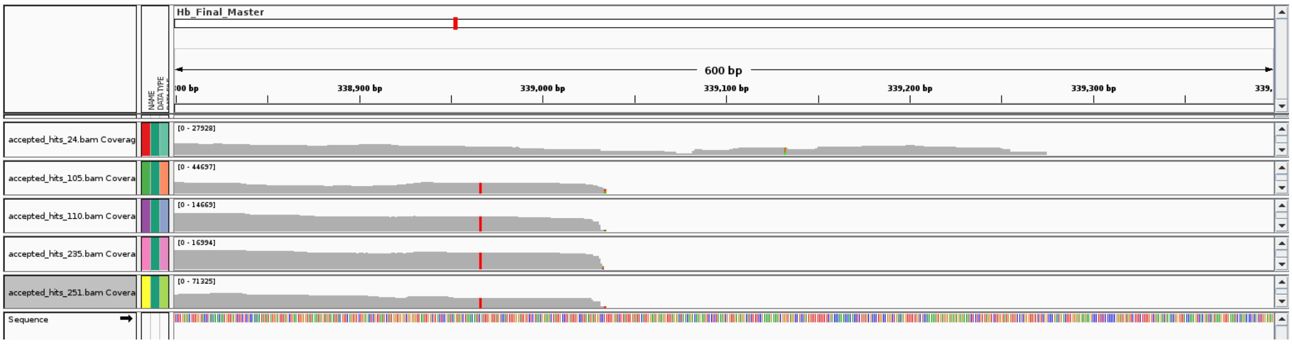

Figure S4.5: RNA-seq read depth at the break site near 338 kb showing additional sequence unique to BPM 24

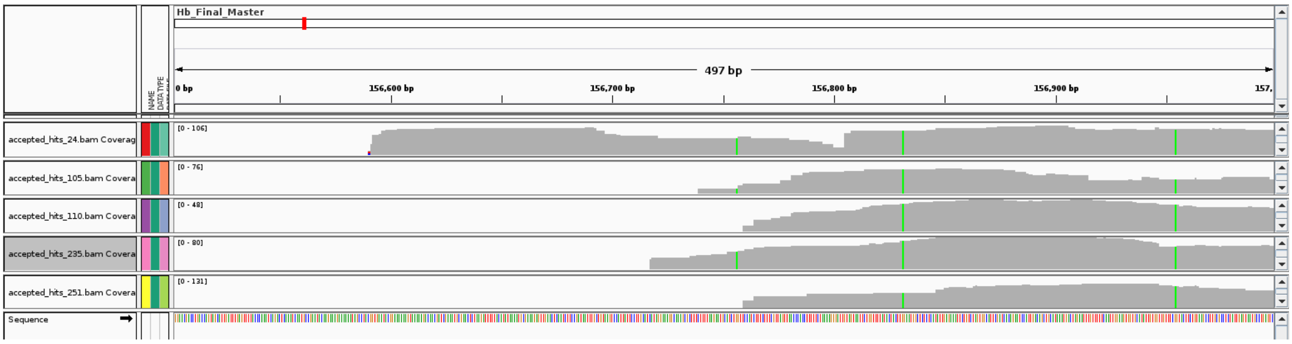

Figure S4.6: RNA-seq read depth at the break site near 157 kb showing additional sequence unique to BPM 24

Table S4.3: Primers used to check expression at confirmed rearrangements between published contigs and the BPM 24 master circle.

**Primer**	**Sequence**	**Pair**
Master_e339274_F	acttcttgtctgttagtcctaaacc	AJJZ010142287_R
Master_e156677_F	tcttccgataatttgctgtaagtcc	AJJZ010174367_R

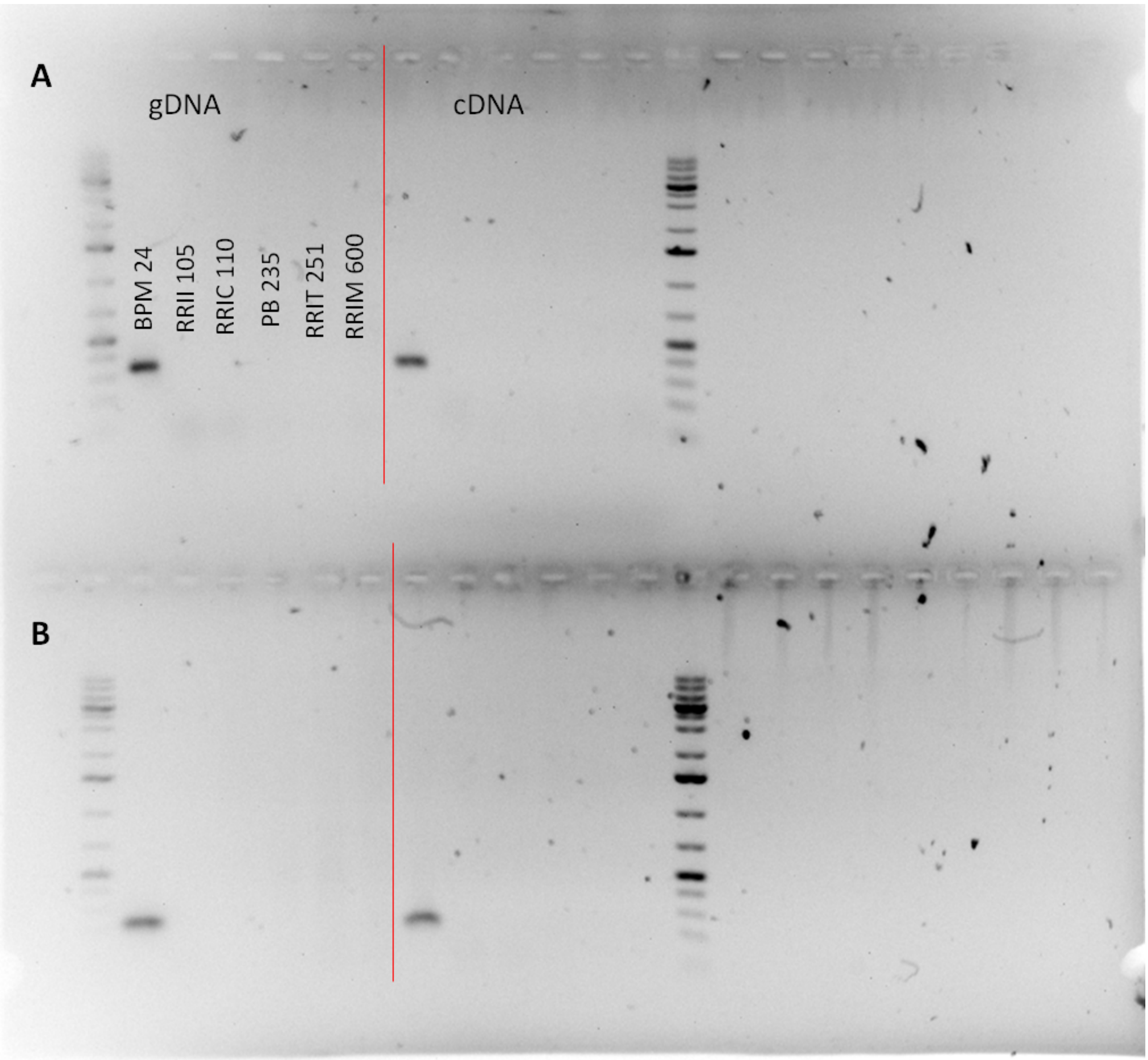

Figure S4.7: **A**. Products from cDNA and genomic DNA (respectively) for the rearrangement at 339 kb. **B**. Products from cDNA and genomic DNA (respectively) for the rearrangement at 157 kb. The DNA ladder is the Fermentas 1 kb plus generuler.
