# Supplementary material for: Assembly and analysis of a male sterile rubber tree mitochondrial genome reveals DNA rearrangement events and a novel transcript
Source: BMC Plant Biol. 2014 Feb 10;14:45. doi: 10.1186/1471-2229-14-45 (PMC3925788; doi:10.1186/1471-2229-14-45)
Supplement: Additional file 3 — Mapping results of the Illumina genomic data against the rubber tree master circle genome. [file 1471-2229-14-45-S3.pdf]

Supplementary File 2

We used paired end sequence data from an Illimina HiSeq2000 run to confirm that the sequence we identified as mitochondrial was indeed mitochondrial sequence and not a nuclear encoded copy of mitochondrial DNA or chloroplast sequence. The data was mapped to the master circle rubber tree mitochondrial genome and read depth per locus was determined using samtools mpileup. The resulting read depths were largely in the range of 80 to 300 with the median read depth being 186 (Figure S2.1A and S2.1B). The maximum read depth was 4843 and is from chloroplast sequence that has been transferred to the mitochondria, the high read depth is the result of chloroplast sequences partially mapping to these sections (Figure S2.1A). To identify regions of differing copy number we took the mean read depth of 100 bp bins of the rubber tree mitochondrial genome and used the R package changepoint (http://cran.r-project.org/web/packages/changepoint/index.html) to identify levels where this mean changed significantly from the surrounding means and graphed the data (Figure S2.1A and S2B).

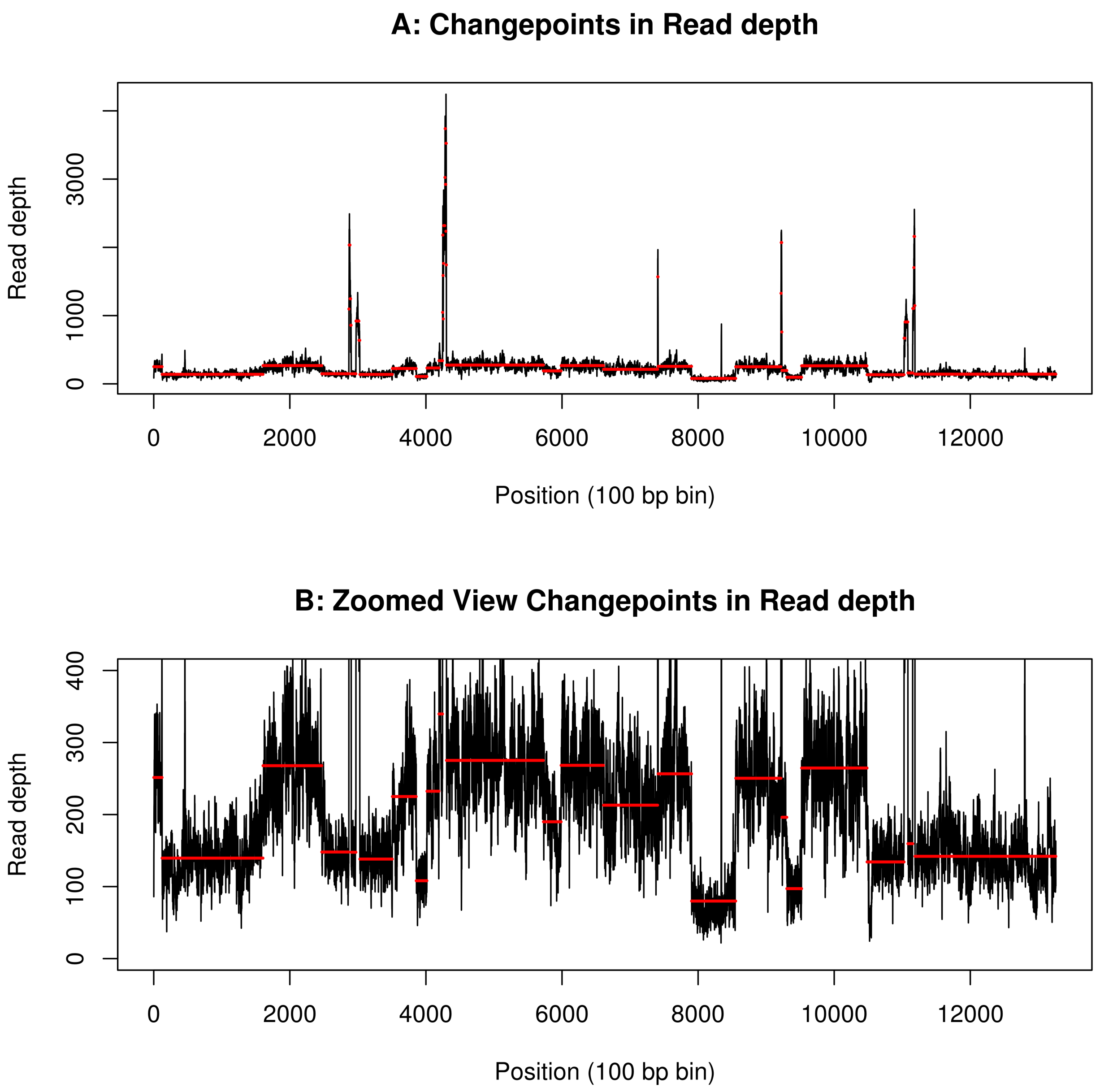

Figure S2.1: Read depth of mapped illumina paired-end reads to the rubber tree master circle where each point is the mean of a 100 bp bin of sequence. A: large spikes in read depth represent chloroplast derived miochondrial sequence. B: Zoom in at y axis of figure S2.1A to highlight the changes in read depth of mitochondrial sequence.
