# Supplementary material for: Assembly and analysis of a male sterile rubber tree mitochondrial genome reveals DNA rearrangement events and a novel transcript
Source: BMC Plant Biol. 2014 Feb 10;14:45. doi: 10.1186/1471-2229-14-45 (PMC3925788; doi:10.1186/1471-2229-14-45)
Supplement: Additional file 4 — Table of RNA editing events in the rubber tree RNA-seq data and comparison of RNA editing events to RNA editing event information from genbank. [file 1471-2229-14-45-S4.pdf]

Supplementary File 3

Cases of RNA-editing in expressed rubber tree mitochondrial genes were identified by mapping RNA-seq data from six varieties of rubber tree against the rubber tree master circle mitochondrial genome. There were 224 instances where the RNA-seq reads had a T and the genomic sequence had a C were considered as RNA-edited events. RNA-editing was also predicted using PREP-Mt which predicted 184 (82%) of the above editing sites (table S3.1).

Table S3.1: RNA-editing sites, effect and state in each of six rubber tree varieties

Position	Gene	Ref	Var	Editing site	BPM 24	RRIM 600	RRII 105	RRIC 110	RRIT 235	PB 251	predicted
12778	shd3	C	T	shd3eU67PS	C/T	T	T	NA	T	NA	yes
12785	shd3	C	T	shd3eU74SF	C/T	T	NA	T	C/T	NA	yes
12801	shd3	G	A	shd3eU90	G	NA	NA	A	A	A	no
12915	shd3	C	T	shd3eU204	C/T	NA	C/T	C/T	C/T	C/T	no
12995	shd3	C	T	shd3eU284	C	T	T	T	T	T	no
13213	nad4L	C	T	nad4LeU8PL	C/T	C	C/T	C/T	C/T	C	yes
13246	nad4L	C	T	nad4LeU41SF	T	C	C/T	NA	T	NA	yes
13402	nad4L	C	T	nad4LeU197PL	C/T	C	C/T	C/T	T	NA	yes
13756	atp4	C	T	atp4eU59SF	NA	NA	T	C/T	T	NA	no
13786	atp4	C	T	atp4eU89SL	T	T	NA	T	C/T	T	yes
13815	atp4	C	T	atp4eU118RC	T	T	NA	T	T	T	yes
13912	atp4	C	T	atp4eU215SL	T	NA	T	T	T	T	yes
14079	atp4	C	T	atp4eU382	C	C	C/T	C/T	C	C/T	no
14092	atp4	C	T	atp4eU395SL	T	C	T	T	T	T	yes
14113	atp4	C	T	atp4eU416TI	T	NA	T	T	T	C/T	yes
15940	ccmC	C	T	ccmCeU5SF	C/T	T	T	T	T	NA	no
16114	ccmC	C	T	ccmCeU179AV	T	NA	C/T	T	T	NA	yes

16119	ccmC	C	T	ccmCeU184RW	T	NA	C/T	T	T	NA	yes
16187	ccmC	C	T	ccmCeU252	C	NA	C/T	C/T	C/T	NA	no
16216	ccmC	C	T	ccmCeU281TI	NA	NA	T	T	T	T	no
26812	nad7_exon5	G	A	nad7eU1137	A	NA	G/A	G/A	G/A	G/A	no
26825	nad7_exon5	G	A	nad7eU1124PL	A	NA	A	G/A	A	G/A	yes
26892	nad7_exon5	G	A	nad7eU1057RC	A	NA	A	NA	A	A	yes
26899	nad7_exon5	G	A	nad7eU1050	A	NA	A	A	A	A	no
26986	nad7_exon5	G	A	nad7eU963	A	NA	G/A	G/A	G/A	G	no
28881	nad7_exon4	G	A	nad7eU836PL	A	G/A	A	A	A	A	yes
30187	nad7_exon3	G	A	nad7eU578SL	A	A	A	A	A	G/A	no
30300	nad7_exon3	G	A	nad7eU465	G/A	NA	G/A	G	G/A	G	no
30382	nad7_exon3	G	A	nad7eU383SL	A	NA	A	A	A	G/A	yes
30541	nad7_exon3	G	A	nad7eU224TM	NA	G/A	A	A	A	G	yes
30561	nad7_exon2	G	A	nad7eU200SF	A	A	A	G/A	G/A	A	yes
33183	nad7_exon1	G	A	nad7eU38SL	A	A	A	A	A	NA	yes
83148	cob	G	A	cobeU1124PL	A	NA	A	A	A	A	yes
83188	cob	G	A	cobeU1084PS	A	NA	A	A	A	A	yes
83290	cob	G	A	cobeU982HY	G/A	NA	A	A	G/A	A	yes
83419	cob	G	A	cobeU853HY	G/A	A	A	A	A	A	yes
83464	cob	G	A	cobeU808PS	A	A	A	A	G/A	A	yes
83704	cob	G	A	cobeU568HY	G/A	NA	A	A	A	G/A	yes
83914	cob	G	A	cobeU358RW	A	NA	A	A	A	A	yes
83947	cob	G	A	cobeU325HY	A	NA	NA	NA	A	G/A	yes
83986	cob	G	A	cobeU286LF	A	A	A	A	A	A	yes
84094	cob	G	A	cobeU178HY	A	NA	A	A	A	A	yes
84154	cob	G	A	cobeU118PS	A	NA	A	A	A	A	yes
85930	rpl5	G	A	rpl5eU518PL	G/A	NA	A	A	A	NA	yes
85933	rpl5	G	A	rpl5eU515PL	G/A	NA	A	A	A	NA	yes

86116	rpl5	G	A	rpl5eU332SL	A	NA	A	A	A	G/A	no
86181	rpl5	G	A	rpl5eU267	G/A	NA	G/A	A	G/A	G/A	no
86282	rpl5	G	A	rpl5eU166PS	G/A	NA	A	A	A	A	no
98491	nad1_exon1	G	A	nad1eU265RW	A	NA	A	G/A	A	NA	yes
98541	nad1_exon1	G	A	nad1eU215SF	A	NA	A	A	A	A	yes
98589	nad1_exon1	G	A	nad1eU167SL	A	NA	A	NA	A	A	yes
98716	nad1_exon1	G	A	nad1eU40	G/A	NA	G/A	G/A	G/A	G	yes
98754	nad1_exon1	G	A	nad1eU2TM	G/A	NA	G/A	G/A	G/A	G/A	yes
123509	nad2_exon5	G	A	nad2eU1400SL	A	A	G/A	G/A	G/A	G/A	yes
125277	nad2_exon4	G	A	nad2eU1127SL	A	NA	A	G/A	G/A	A	yes
125476	nad2_exon4	G	A	nad2eU928HY	A	NA	G/A	G/A	NA	NA	yes
125484	nad2_exon4	G	A	nad2eU920PL	A	NA	G/A	G/A	NA	NA	yes
125595	nad2_exon4	G	A	nad2eU809SF	A	NA	A	A	A	A	yes
125604	nad2_exon4	G	A	nad2eU800SL	A	NA	A	A	A	A	yes
128281	nad2_exon3	G	A	nad2eU662SF	G	NA	A	G/A	G/A	G	yes
128334	nad2_exon3	G	A	nad2eU609	G	NA	G	G/A	A	G	yes
148471	nad9	C	T	nad9eU92SF	T	NA	C	T	T	T	yes
148546	nad9	C	T	nad9eU167SL	T	NA	C/T	C/T	C/T	T	yes
148569	nad9	C	T	nad9eU190HY	T	NA	C/T	C/T	C/T	C/T	yes
148677	nad9	C	T	nad9eU298PS	T	NA	C/T	C/T	T	C/T	yes
148707	nad9	C	T	nad9eU328RW	T	NA	C/T	T	T	T	yes
162758	rps1	G	A	rps1eU781	G/A	G/A	G/A	G/A	G/A	NA	no
163327	rps1	G	A	rps1eU212PS	A	NA	A	A	A	G/A	no
174030	ccmFn	C	T	ccmFneU38PL	T	NA	T	NA	T	NA	yes
174357	ccmFn	C	T	ccmFneU365SL	T	NA	C	T	C/T	NA	yes
174944	ccmFn	C	T	ccmFneU952RC	T	T	C/T	NA	T	NA	yes
181673	nad5_exon4	G	A	nad5eU1610PL	A	NA	A	A	A	A	yes
181694	nad5_exon4	G	A	nad5eU1589SF	A	NA	G/A	NA	NA	A	yes

181733	nad5_exon4	G	A	nad5eU1550TI	G/A	G/A	G/A	G/A	G/A	A	yes
181793	nad5_exon4	G	A	nad5eU1490PL	A	NA	A	A	A	NA	yes
221616	cox1_exon1	C	T	cox1eU15	C/T	T	T	C/T	C/T	C/T	no
221843	cox1_exon1	C	T	cox1eU242SF	T	NA	T	T	C/T	T	yes
221855	cox1_exon1	C	T	cox1eU254SF	T	NA	T	T	C/T	C/T	yes
221944	cox1_exon1	C	T	cox1eU343	NA	C/T	C	NA	NA	C	no
222044	cox1_exon1	C	T	cox1eU443SL	T	NA	T	T	C/T	T	yes
222053	cox1_exon1	C	T	cox1eU452SF	T	NA	T	T	T	T	yes
222116	cox1_exon1	C	T	cox1eU515SF	T	NA	T	T	T	T	yes
222152	cox1_exon1	C	T	cox1eU551SL	T	T	T	T	T	T	yes
222191	cox1_exon1	C	T	cox1eU590PL	T	T	T	T	T	T	yes
223329	cox1_exon2	C	T	cox1eU761SF	T	NA	T	T	T	T	yes
223606	cox1_exon2	C	T	cox1eU1038	C/T	T	C/T	C/T	C/T	C/T	no
223647	cox1_exon2	C	T	cox1eU1079PL	T	T	T	T	T	T	yes
223754	cox1_exon2	C	T	cox1eU1186HY	T	T	T	T	C/T	T	yes
223842	cox1_exon2	C	T	cox1eU1274PL	T	T	T	T	T	C/T	yes
223855	cox1_exon2	C	T	cox1eU1287	C/T	NA	C/T	C/T	C/T	T	no
223957	cox1_exon2	C	T	cox1eU1389	C	C	C/T	C/T	C/T	C/T	no
223970	cox1_exon2	C	T	cox1eU1402RC	C/T	T	T	T	NA	T	yes
223973	cox1_exon2	C	T	cox1eU1405RC	C/T	T	T	NA	NA	T	yes
224001	cox1_exon2	C	T	cox1eU1433SL	T	T	T	T	T	T	yes
224057	cox1_exon2	C	T	cox1eU1489PS	T	NA	T	T	T	T	yes
229015	nad5_exon2	G	A	nad5eU1310SL	A	NA	A	A	A	A	yes
229600	nad5_exon2	G	A	nad5eU725SL	G/A	NA	A	A	A	A	yes
229612	nad5_exon2	G	A	nad5eU713SL	G/A	A	A	G/A	A	A	yes
229786	nad5_exon2	G	A	nad5eU539PL	A	NA	NA	A	A	NA	yes
229927	nad5_exon2	G	A	nad5eU398SF	A	NA	A	G/A	A	A	yes
229951	nad5_exon2	G	A	nad5eU374PL	A	NA	A	A	A	A	yes

231013	nad5_exon1	G	A	nad5eU155RW	G/A	A	G/A	A	G/A	G/A	yes
352739	MatR	C	T	matReU32SF	T	NA	T	T	T	T	yes
353033	MatR	C	T	matReU326QL	C/T	T	T	T	T	NA	yes
354252	MatR	C	T	matReU1545	C/T	NA	C/T	C/T	C/T	C/T	no
354463	MatR	C	T	matReU1756PS	C	T	C/T	C/T	T	C/T	yes
354533	MatR	C	T	matReU1826PL	C/T	T	C/T	T	C/T	C/T	yes
354551	MatR	C	T	matReU1844SL	T	NA	T	T	T	C/T	yes
355592	nad1_exon5	C	T	nad1eU823LF	T	C	C/T	C/T	C/T	C/T	yes
355667	nad1_exon5	C	T	nad1eU898RW	T	NA	A	C/T	C/T	T	yes
355697	nad1_exon5	C	T	nad1eU928RW	T	NA	C/T	C/T	C/T	T	yes
355706	nad1_exon5	C	T	nad1eU937PS	T	C	C/T	C/T	C/T	T	yes
450681	nad1_exon3	G	A	nad1eU635SL	G/A	NA	G	A	G	NA	yes
450743	nad1_exon3	G	A	nad1eU573	A	NA	G	A	G/A	NA	no
546014	ccmFc_exon2	G	A	ccmFceU1315R*	A	NA	G/A	A	A	A	yes
546061	ccmFc_exon2	G	A	ccmFceU1268SL	A	NA	G/A	A	A	A	no
546451	ccmFc_exon2	G	A	ccmFceU878SF	A	A	NA	A	A	A	yes
547881	ccmFc_exon1	G	A	ccmFceU406RC	G/A	G/A	A	G/A	G/A	G/A	yes
547977	ccmFc_exon1	G	A	ccmFceU310RC	A	A	A	A	A	A	no
584511	rpl10	G	A	rpl10eU397LF	G	NA	A	NA	G/A	A	no
584578	rpl10	G	A	rpl10eU330	G	NA	A	NA	G/A	A	no
584594	rpl10	G	A	rpl10eU314SL	G	NA	A	NA	A	A	yes
584899	rpl10	G	A	rpl10eU9	NA	NA	G/A	NA	A	A	no
589445	nad6	G	A	nad6eU463PS	G/A	NA	A	A	A	A	no
589747	nad6	G	A	nad6eU161PL	G/A	NA	A	A	G/A	A	yes
589762	nad6	G	A	nad6eU146SF	G/A	NA	A	A	A	A	yes
589882	nad6	G	A	nad6eU26PL	A	NA	G/A	A	A	NA	no
590588	rps4	G	A	rps4eU1027RW	G/A	A	NA	NA	NA	A	yes
590593	rps4	G	A	rps4eU1022PL	G/A	A	NA	NA	NA	A	yes

590689	rps4	G	A	rps4eU926SL	A	NA	NA	NA	G/A	A	yes
591121	rps4	G	A	rps4eU494SL	A	NA	NA	NA	A	A	yes
591439	rps4	G	A	rps4eU176SL	A	NA	NA	A	G/A	G	yes
591470	rps4	G	A	rps4eU145PS	G/A	NA	NA	A	A	A	yes
591554	rps4	G	A	rps4eU61RW	G/A	G	A	A	G/A	A	yes
624907	nad2_exon1	C	T	nad2eU26SF	T	NA	T	T	T	NA	yes
624937	nad2_exon1	C	T	nad2eU56SF	C/T	NA	C/T	T	C	NA	yes
624951	nad2_exon2	C	T	nad2eU70LF	T	NA	T	C/T	T	C/T	no
626482	nad2_exon2	C	T	nad2eU344PL	T	NA	T	T	T	T	no
664637	nad4_exon1	C	T	nad4eU29SF	T	NA	C/T	T	C	T	yes
664652	nad4_exon1	C	T	nad4eU44PL	T	NA	C/T	T	C	T	yes
664925	nad4_exon1	C	T	nad4eU317SL	C/T	NA	C/T	T	T	T	yes
666645	nad4_exon2	C	T	nad4eU608SL	T	NA	T	T	C/T	T	yes
666696	nad4_exon2	C	T	nad4eU659SF	T	NA	T	T	T	T	yes
666804	nad4_exon2	C	T	nad4eU767PL	T	NA	T	T	T	T	yes
670444	nad4_exon3	C	T	nad4eU1010PL	T	NA	T	C/T	T	NA	yes
670535	nad4_exon3	C	T	nad4eU1101	C	NA	C/T	C	C/T	C/T	no
670563	nad4_exon3	C	T	nad4eU1129LF	T	NA	T	T	T	C/T	yes
670606	nad4_exon3	C	T	nad4eU1172SL	T	NA	T	NA	NA	NA	yes
670645	nad4_exon3	C	T	nad4eU1211SL	C/T	NA	T	T	NA	NA	yes
670789	nad4_exon3	C	T	nad4eU1355SL	T	NA	T	T	T	T	yes
673519	nad4_exon4	C	T	nad4eU1417HY	T	C/T	C/T	T	C/T	C/T	yes
673535	nad4_exon4	C	T	nad4eU1433PL	C/T	C	C/T	C/T	C/T	C/T	yes
697141	atp6	C	T	atp6eU37PS	T	C	T	T	C/T	T	yes
697220	atp6	C	T	atp6eU116SL	C/T	NA	C/T	G/A	C/T	T	yes
697271	atp6	C	T	atp6eU167PL	C/T	NA	A	G/A	C/T	T	yes
697277	atp6	C	T	atp6eU173PL	C/T	NA	C/T	T	C/T	T	yes
697505	atp6	C	T	atp6eU401SL	C/T	T	T	T	C/T	T	yes

697564	atp6	C	T	atp6eU460PS	C/T	NA	C/T	C/T	C/T	T	yes
697567	atp6	C	T	atp6eU463HY	T	NA	C/T	C/T	C/T	NA	yes
697589	atp6	C	T	atp6eU485SL	T	NA	C/T	C/T	C/T	T	yes
697631	atp6	C	T	atp6eU527SL	T	NA	C/T	C/T	C/T	T	yes
697703	atp6	C	T	atp6eU599PL	C/T	NA	C/T	NA	T	T	yes
697768	atp6	C	T	atp6eU664HY	C/T	NA	T	T	C/T	NA	yes
697775	atp6	C	T	atp6eU671SF	C/T	NA	T	T	T	NA	yes
713794	cox2_exon1	C	T	cox2eU27	C/T	T	C/T	C/T	C/T	C/T	no
713838	cox2_exon1	C	T	cox2eU71SF	C/T	T	C/T	C/T	T	T	yes
713923	cox2_exon1	C	T	cox2eU156	C/T	T	C/T	C/T	C/T	C/T	no
713930	cox2_exon1	C	T	cox2eU163RW	T	T	C/T	T	T	T	yes
714020	cox2_exon1	C	T	cox2eU253RW	C/T	T	C/T	T	T	T	yes
714045	cox2_exon1	C	T	cox2eU278PL	C/T	T	T	T	T	T	yes
714146	cox2_exon1	C	T	cox2eU379RW	T	T	T	T	C/T	T	yes
714210	cox2_exon1	C	T	cox2eU443TM	T	C/T	C/T	T	C/T	T	yes
714243	cox2_exon1	C	T	cox2eU476SL	T	C/T	C/T	T	T	T	yes
714324	cox2_exon1	C	T	cox2eU557PL	T	T	T	T	T	T	yes
714348	cox2_exon1	C	T	cox2eU581SL	T	NA	T	T	T	G/A	yes
714399	cox2_exon1	C	T	cox2eU632SL	T	C	C/T	C/T	C/T	C/T	yes
714465	cox2_exon1	C	T	cox2eU698TM	T	T	T	T	T	T	yes
716543	cox2_exon2	C	T	cox2eU721PS	C/T	NA	C/T	C/T	C/T	C/T	yes
716564	cox2_exon2	C	T	cox2eU742RW	C/T	NA	C/T	C/T	C/T	C/T	yes
717741	mttB	C	T	mttBeU262HY	C/T	NA	NA	NA	C/T	T	yes
742834	ccmB	C	T	ccmBeU424RC	C/T	NA	C	C	C/T	NA	yes
743006	ccmB	C	T	ccmBeU596SL	C/T	NA	T	NA	C/T	NA	yes
761183	atp9	C	T	ccmBeU223RX	T	T	T	T	T	T	yes
854178	atp9	C	T	atp9eU20SL	T	C	T	C/T	C/T	T	yes
882681	atp8	C	T	atp8eU458PL	T	T	T	T	T	T	yes

883993	cox3	C	T	cox3eU245PL	T	NA	T	T	T	T	yes
884011	cox3	C	T	cox3eU263PL	T	NA	T	T	T	T	yes
884037	cox3	C	T	cox3eU289LF	NA	NA	T	NA	C/T	T	yes
884059	cox3	C	T	cox3eU311SF	NA	NA	T	NA	C/T	T	yes
884136	cox3	C	T	cox3eU388RW	T	T	T	T	C/T	T	yes
884161	cox3	C	T	cox3eU413PL	T	T	T	T	A	T	yes
884260	cox3	C	T	cox3eU512SL	C/T	T	C/T	C/T	C/T	T	yes
884502	sdh4	C	T	sdh4eU29SL	A	NA	T	C/T	T	T	no
884512	sdh4	C	T	sdh4eU39	C/T	NA	C/T	C/T	T	T	no
884826	sdh4	C	T	sdh4eU353SF	C/T	NA	C/T	T	C/T	C/T	yes
1031134	rps12	G	A	rps12eU284SF	G/A	NA	G/A	G/A	G/A	G/A	yes
1031149	rps12	G	A	rps12eU269SL	G/A	NA	G/A	G/A	A	A	yes
1031222	rps12	G	A	rps12eU196HY	A	A	A	G/A	A	G/A	yes
1031272	rps12	G	A	rps12eU146PL	A	A	A	G/A	G/A	A	yes
1031318	rps12	G	A	rps12eU100RC	G/A	G/A	A	G/A	G/A	G/A	yes
1031347	rps12	G	A	rps12eU71SL	A	A	G/A	G/A	G/A	G/A	yes
1031615	nad3	G	A	nad3eU208LF	A	A	G/A	G/A	A	A	yes
1031677	nad3	G	A	nad3eU146SF	A	NA	A	A	A	A	yes
1031743	nad3	G	A	nad3eU80PL	A	NA	A	A	NA	G/A	yes
1031751	nad3	G	A	nad3eU344SL	A	NA	G/A	G/A	A	G/A	yes
1031756	nad3	G	A	nad3eU349RW	A	NA	G/A	G/A	A	G/A	yes
1031818	nad3	G	A	nad3eU5SL	A	NA	G/A	G/A	G/A	G/A	yes
1046524	rpl16	G	A	nad3eU407SL	G/A	G	A	G/A	G/A	A	yes
1046530	rpl16	G	A	nad3eU401PL	A	G/A	A	G/A	A	A	yes
1046596	rpl16	G	A	rpl16eU335PL	G/A	G/A	A	A	G/A	G/A	yes
1046699	rpl16	G	A	rpl16eU232LF	G/A	A	G/A	G/A	G/A	G/A	yes
1046827	rpl16	G	A	rpl16eU104TI	G/A	G/A	A	G/A	G/A	G/A	yes
1046975	rps3	G	A	rps3eU1619SL	G/A	G/A	G/A	G/A	G/A	G/A	yes

1047006	rps3	G	A	rps3eU1588PS	G/A	A	G/A	G/A	G/A	G/A	no
1047039	rps3	G	A	rps3eU1555RC	G/A	A	G/A	G/A	G/A	G/A	yes
1047563	rps3	G	A	rps3eU1031PL	G/A	NA	G/A	G/A	G/A	G/A	yes
1047704	rps3	G	A	rps3eU890SL	G/A	NA	G/A	G/A	G/A	G/A	no
1047881	rps3	G	A	rps3eU713SL	G/A	G/A	G/A	G/A	G/A	G/A	yes
1048502	rps3	G	A	rps3eU92SL	A	G/A	G/A	G/A	G/A	G/A	yes
1168653	atp1	C	T	atp1eU1039PS	T	T	T	T	T	T	yes
1168792	atp1	C	T	atp1eU1178SL	T	T	T	T	C/T	T	yes
1168830	atp1	C	T	atp1eU1216LF	T	T	T	C/T	C/T	T	yes
1168906	atp1	C	T	atp1eU1292PL	C/T	NA	T	T	T	T	yes
1169029	atp1	C	T	atp1eU1415PL	T	T	T	T	T	T	yes
1169104	atp1	C	T	atp1eU1490PL	T	NA	T	C	T	T	yes

RNA-editing comparison

We obtained RNA-editing information from genbank for all plant species where RNA-editing information was available for all rubber tree genes with sufficient RNA-seq depth to identify RNA-editing events. Some species had RNA-editing information available for all of their mitochondrial genes, but many species only had information available for some genes. We converted the RNA-edited C into a U for all species including the six rubber tree varieties and then aligned the resulting sequence to identify sites of RNA-editing that were conserved or novel. Cases where the sequence depth for individual rubber tree varieties was insufficient at RNA-edited sites have an 'N' instead of a C or U call. There were many cases where rubber tree appears to lack an RNA-editing site, however this was largely due to low or no coverage in all of the varieties at that particular site, this is most noticeable in *nad4* and *nad7*.

atp1 multiple sequence alignment

```
Lotus japonicus                  ---------------------------GAAGATCAGAACGAAATTACTTA
BPM24_atp1                       --------------------------------------------------
Millettia pinnata                -------------------------------GTCTGGGCGAACTTCAAGA
RRIM600_atp1                     --------------------------------------------------
Arabidopsis thaliana             ----------------------------GAGTCCCGCCTCGAAGAAAGAA
Brassica napus                   -----------------------GGGTATAAATATACCT-AAAGTTTTAA
RRII105_atp1                     --------------------------------------------------
Beta macrocarpa                  ATTACCCCCGGAATGGACCATGCCTGACCTTGTTCGGACAGTGCTTGGGG
Beta vulgaris vulgaris           ATTACCCCCGGAATGGACCATGCCTGACCTTGTTCGGACAGTGCTTGGGG
Beta vulgaris maritima           ATTACCCCCGGAATGGACCATGCCTGACCTTGTTCGGACAGTGCTTGGGG
Silene latifolia                 ----------------------------------AGGATTGGGGATGAGG
PB235_atp1                       --------------------------------------------------
RRIT251_atp1                     --------------------------------------------------
Citrullus lanatus                -------------TGGTCTATCCAAGAATTTTCCCGTACGGTGATTGGGG
Cucurbita pepo                   ---------------------CCGGGTAGGGGCTGGC-TATTGGACGGGG
RRIC110_atp1                     --------------------------------------------------
Oryza sativa Japonica            --------------------------------TTCGCATAAATGAAAAAA
Cycas taitungensis               ---------------GGTCCAATTCAATTATGCACCACCCCCCTTTAGCG
Anomodon rugelii                 --------------------------------------------------
Anomodon attenuatus              --------------------------------------------------
Megaceros aenigmaticus           --------------------------------------------------
Phaeoceros laevis                --------------------------------------------------


Lotus japonicus                  GAGTAATCTTTTATTT-TTCATCCCGAACTAGACAGACAGAGCTTCATAC
BPM24_atp1                       --------------------------------------------------
Millettia pinnata                ATGGAAGGATTCACT----AAGGCAAAACTCCCAACATTACAAATCCATT
RRIM600_atp1                     --------------------------------------------------
Arabidopsis thaliana             GGTTTCAGTCTCATTCAACCAACTCTCTTTTTGAAGCAGATAGTTCACGA
Brassica napus                   ATCTTTTTTTTTATT--ATTGTTGATACTCCTGCAGAGAATCTCTTAGGA
RRII105_atp1                     --------------------------------------------------
Beta macrocarpa                  ATGAAGCTCTTCAGC-AG---GGCTTTCTGACGGATGCCTACTATGATGT
Beta vulgaris vulgaris           ATGAAGCTCTTCAGC-AG---GGCTTTCTGACGGATGCCTACTATGATGT
```

```
Beta vulgaris maritima          ATGAAGCTCTTCAGC-AG---GGCTTTCTGACGGATGCCTACTATGATGT
Silene latifolia                ATGACGCTCTTCAGC-AC---GTCTTTCTCAAGGATGCCTACTATGATCT
PB235_atp1                      --------------------------------------------------
RRIT251_atp1                    --------------------------------------------------
Citrullus lanatus               AAGAGGTGGTTCAAG-ACCCCTCTTATCTAAGCGACGTCTTGTCCGATCT
Cucurbita pepo                  CCCGGGTTAT-CAAG-GCTCAAAATGGCAAG--GACTTCTCATTGAGCAG
RRIC110_atp1                    --------------------------------------------------
Oryza sativa Japonica           AAAGCGAGATGTGCACGAAAGAAGATCATAGTTCAGCTTTAAAATGGTGG
Cycas taitungensis              GAGAGGGTAAGCGGA-AGCAAGACAAATGGTCATAGAAGCAATCTAATAG
Anomodon rugelii                --------------------------------------------------
Anomodon attenuatus             --------------------------------------------------
Megaceros aenigmaticus          --------------------------------------------------
Phaeoceros laevis               --------------------------------------------------


Lotus japonicus                 GGTGCTTTTCATAAGTTCGAGTCT-TCCTGTCTGGTTTGATTCTACCTGT
BPM24_atp1                      --------------------------------------------------
Millettia pinnata               TTTGGCTTCTATAAGCCCGGGGGGGTCTTTTATGAAGGGAATACTCCT-T
RRIM600_atp1                    --------------------------------------------------
Arabidopsis thaliana            GATAAT-TGAGTAAGTTAAGATAGATGTAACAATTTGAACCAAAGGCCTT
Brassica napus                  GCGAACGCGATCTATTTAGACCTGATCTATCAGG--GCACGAATAGTCGC
RRII105_atp1                    --------------------------------------------------
Beta macrocarpa                 GATGTTATGTGGCACTCATAGTTGGGGATGCGAGGAGCTCCTGAATTTGC
Beta vulgaris vulgaris          GATGTTATGTGGCACTCATAGTTGGGGATGCGAGGAGCTCCTGAATTTGC
Beta vulgaris maritima          GATGTTATGTGGCACTCATAGTTGGGGATGCGAGGAGCTCCTGAATTTGC
Silene latifolia                TATGTTATGTGGCACTCGTAGTTGGTTATGCGACGAGGTTTTTAAGTTTT
PB235_atp1                      --------------------------------------------------
RRIT251_atp1                    --------------------------------------------------
Citrullus lanatus               TTCACTTCATGGTTTGCAGAGTTGGTATTGGGAGGAAGCTGTGAAGCTTC
Cucurbita pepo                  GCTCAATGCTATCTTTCACAGTCAAGAGAACAACGAGG--GCCGCGCTCC
RRIC110_atp1                    --------------------------------------------------
Oryza sativa Japonica           TGTCCCTGTGTTAGTAAGTGGTTGAAATAGCTCATGGGAGTGTCTGCCCC
Cycas taitungensis              TATAGAGCCTAATTATGGCATTTAGTCTTTATTAGATCCGGTTCGCCCTT
Anomodon rugelii                --------------------------------------------------
Anomodon attenuatus             --------------------------------------------------
Megaceros aenigmaticus          --------------------------------------------------
Phaeoceros laevis               --------------------------------------------------


Lotus japonicus                 TCGATCAAAGGACAAG--CCTCTCTCTTTTTCAATTCTTCCAACCCCCTG
BPM24_atp1                      --------------------------------------------------
Millettia pinnata               ATGGTTATAGGACGTA--CTAGTTGCTCGATCAGGACCCTAGCTTTATTG
RRIM600_atp1                    --------------------------------------------------
Arabidopsis thaliana            TTTAGTGATTAGTCTG--CTAGTT--TCTTTGATCTTAGTCGGTGTAAAT
Brassica napus                  TACTTTGCCCAAGCT---CTGGAG--TTAGTGCTTTCGTTCAGTTCATAA
RRII105_atp1                    --------------------------------------------------
Beta macrocarpa                 TGGATCTAAT--------CAACTAT--------TCTTGATCTGGGCGGG-
Beta vulgaris vulgaris          TGGATCTAAT--------CAACTAT--------TCTTGATCTGGGCGGG-
Beta vulgaris maritima          TGGATCTAAT--------CAACTAT--------TCTTGATCTGGGCGGG-
Silene latifolia                TGGATCTAAT--------CAGCTATAACCTATGTCTTTCTTTGGAAAGAG
PB235_atp1                      --------------------------------------------------
RRIT251_atp1                    --------------------------------------------------
Citrullus lanatus               TAACTAGAATTACCAGCTCGGTAATGTGAAAGAGAACATCTTAGTCTTTA
Cucurbita pepo                  TACTTTCACTTTCACTCTCTTCTTTCTAAAGAAGCCCGCTCTTGTCTTGA
RRIC110_atp1                    --------------------------------------------------
Oryza sativa Japonica           ATTCGATAATGGCATTTATGATCTAGTGGAGTGAGTGATTGTGTGGTGTT
Cycas taitungensis              TCAAATGAATGA-----ATGAATCGAAGAAGCAGCTGTTTCATTCCATTC
Anomodon rugelii                --------------------------------------------------
Anomodon attenuatus             --------------------------------------------------
Megaceros aenigmaticus          --------------------------------------------------
Phaeoceros laevis               --------------------------------------------------
```

```
Lotus japonicus                   GGGTCTT----CTTTTGTTTTGCGGTATTCCCCC------ACGAGGTCAA
BPM24_atp1                        --------------------------------------------------
Millettia pinnata                 CGAGCCAAAAACCTTTTGTTTGCGGCATTCCCCCTGTTATAGAAGGTAAA
RRIM600_atp1                      --------------------------------------------------
Arabidopsis thaliana              TATTCCTTTTTTTTTTCTAAAGCGGTATTCCTCCTTGCCTCTTAATTAAA
Brassica napus                    GGTTGACTTTTTTTTTCTAAAGCGGTATTCCTCCTTGCCTCTGAATTCAA
RRII105_atp1                      --------------------------------------------------
Beta macrocarpa                   ----------ATCTATCAGCAGCGGCATTCTCCCTTA-ACTCT----ATT
Beta vulgaris vulgaris            ----------ATCTATCAGCAGCGGCATTCTCCCTTA-ACTCT----ATT
Beta vulgaris maritima            ----------ATCTATCAGCAGCGGCATTCTCCCTTA-ACTCT----ATT
Silene latifolia                  AACTCTTTTCATTTATCAGCAGCGGCATTCTCCTCTATACTCTTTATATT
PB235_atp1                        --------------------------------------------------
RRIT251_atp1                      --------------------------------------------------
Citrullus lanatus                 ----------------CAGCAGCGGCATTCTCCCTTG-ACTCG----AGA
Cucurbita pepo                    TCTGGGCGGGATCTATCAGCAGCGGCATTCTCCCTTG-ACTCG----AGA
RRIC110_atp1                      --------------------------------------------------
Oryza sativa Japonica             CAGTCTAAGGCTTTTTGAAAAGCGGATTTCTCCCTTCTCTCATCCATCGT
Cycas taitungensis                TATTT------TCTCGGTAAAGCGGATTTCTCCCTATCCTCCA----TTT
Anomodon rugelii                  --------------------------------------------------
Anomodon attenuatus               --------------------------------------------------
Megaceros aenigmaticus            --------------------------------------------------
Phaeoceros laevis                 --------------------------------------------------

Lotus japonicus                   ATGAATGGAATTTGA--------ATTATGGAATTCTCATTAAGAGCTGCG
BPM24_atp1                        --------------------------ATGGAATTTTCTCCCAGAGCTGCG
Millettia pinnata                 AAGAATGGAATAT----------ATTATGGACTTTTCTGTAAGAGCTGCT
RRIM600_atp1                      --------------------------ATGGAATTTTCTCCCAGAGCTGCG
Arabidopsis thaliana              A--AATGGAAT--------------TATGGAATTATCTCCTAGAGCTGCG
Brassica napus                    A--AATGGAAT--------------TATGGAATTATCTCCUAGAGCTGCG
RRII105_atp1                      --------------------------ATGGAATTTTCTCCCAGAGCTGCG
Beta macrocarpa                   CTATTTTTGAA-------------TCATGGAATTCTCTCCCAGAGCTGCG
Beta vulgaris vulgaris            CTATTTTTGAA-------------TCATGGAATTCTCTCCCAGAGCTGCG
Beta vulgaris maritima            CTATTTTTGAA-------------TCATGGAATTCTCTCCCAGAGCTGCG
Silene latifolia                  ATATATTAAAAAAAAAAAAAAATTTCATGGAATTCTCTCCCAGAGCTGCG
PB235_atp1                        --------------------------ATGGAATTTTCTCCCAGAGCTGCG
RRIT251_atp1                      --------------------------ATGGAATTTTCTCCCAGAGCTGCG
Citrullus lanatus                 CCATTTGAGAA-------------TCATGGAATTCTCTCCCGGAGCTGCG
Cucurbita pepo                    CCATTTTAGAA-------------TCATGAAATTCTCTCCCGGAGCTGCG
RRIC110_atp1                      --------------------------ATGGAATTTTCTCCCAGAGCTGCG
Oryza sativa Japonica             CTTTGTTAAAG--------------TATGGAATTCTCACCCAGAGCTGCG
Cycas taitungensis                GTTCCTTAAGT---------------AUGAAATTATATCCTGGAGCTGCG
Anomodon rugelii                  -----------AUGAACAAATTAACTGGAAATAAGTTGGCTGGAG---CA
Anomodon attenuatus               -----------AUGAACAAATTAACTGGAAATAAGTTGGCTGGAG---CA
Megaceros aenigmaticus            --------------------------AUGAACAAATTTGCTGGAA---CG
Phaeoceros laevis                 --------------------------AUGAACAAATTTGCTGGAA---CG
                                                                *    *      **    *

Lotus japonicus                   GAACTAACAACTCTATTAGAAAGTAGAATTACCAACTTTTACACAAATTT
BPM24_atp1                        GAACTAACAACTCTATTAGAAAGTCGAATTACCAACTATTACACAAATTT
Millettia pinnata                 GAACTUACTACTCTATTAGAAAGTAGAATTACCAACTTTTACACCAATTT
RRIM600_atp1                      GAACTAACAACTCTATTAGAAAGTCGAATTACCAACTATTACACAAATTT
Arabidopsis thaliana              GAACTAACGAATCTATTCGAAAGTCGAATTAGGAACTTTTACGCGAATTT
Brassica napus                    GAACTAACGAATCTATTCGAAAGTCGAATTAGGAACTTTTACGCGAATTT
RRII105_atp1                      GAACTAACAACTCTATTAGAAAGTCGAATTACCAACTATTACACAAATTT
Beta macrocarpa                   GAACTAACGAATCTATTAGAAAGTAGAATTACCAACTTTTACACGAATTT
Beta vulgaris vulgaris            GAACTAACGAATCTATTAGAAAGTAGAATTACCAACTTTTACACGAATTT
Beta vulgaris maritima            GAACTAACGAATCTATTAGAAAGTAGAATTACCAACTTTTACACGAATTT
Silene latifolia                  GAACTAACAACTCTATTAGAAAGTAGAATTAGCAACTTTTATACGAATTT
PB235_atp1                        GAACTAACAACTCTATTAGAAAGTCGAATTACCAACTATTACACAAATTT
RRIT251_atp1                      GAACTAACAACTCTATTAGAAAGTCGAATTACCAACTATTACACAAATTT
```

```
Citrullus lanatus                 GAACTAACGACTTTATTAGAAAGTCGAATTACCAACTTTTACACGAATTT
Cucurbita pepo                    GAACTAACGACTTTATTAGAAAGTAGAATTACGAACTTTTACACTCATTT
RRIC110_atp1                      GAACTAACAACTCTATTAGAAAGTCGAATTACCAACTATTACACAAATTT
Oryza sativa Japonica             GAACTUACGACTCTATTAGAAAGTAGAATGACCAACTTTTACACGAATTT
Cycas taitungensis                GAACCCACTACTATATCGGGAAGAAGAATCACCAACTATTGCACCAATTT
Anomodon rugelii                  GAACTATCTACATTATTAGAACAAAGAATTACCAATTACTACACCAAATT
Anomodon attenuatus               GAACTATCTACATTATTAGAACAAAGAATTACCAATTACTACACCAAATT
Megaceros aenigmaticus            GAGCCATCTACGTTATTAGAACAAAGAATTACCAACTATTACACCAAATT
Phaeoceros laevis                 GAACCATCTACGTTATTAGAACAAAGAATTACTAACTATTACACCAAATT
                                  ** *   * *   ***  * *    **** *  ** *  *   *  * **

Lotus japonicus                   TCAAGTGGATGAGATCGGTCGAGTGGTCTCAGTTGGAGATGGGATTGCAC
BPM24_atp1                        TCAAGTGGATGAGATCGGTCGAGTGGTCTCAGTTGGAGATGGGATTGCAC
Millettia pinnata                 TCAAGTGGATGAGATCGGTCGAGTGGTCTCAGTTGGAGATGGGATTGCAC
RRIM600_atp1                      TCAAGTGGATGAGATCGGTCGAGTGGTCTCAGTTGGAGATGGGATTGCAC
Arabidopsis thaliana              TCAAGTGGATGAGATCGGTCGAGTGGTCTCAGTTGGAGATGGGATTGCAC
Brassica napus                    TCAAGTGGATGAGATCGGTCGAGTGGTCTCAGTTGGAGATGGGATTGCAC
RRII105_atp1                      TCAAGTGGATGAGATCGGTCGAGTGGTCTCAGTTGGAGATGGGATTGCAC
Beta macrocarpa                   TCAAGTGGATGAGATCGGTCGAGTGGTCTCAGTTGGAGATGGGATTGCAC
Beta vulgaris vulgaris            TCAAGTGGATGAGATCGGTCGAGTGGTCTCAGTTGGAGATGGGATTGCAC
Beta vulgaris maritima            TCAAGTGGATGAGATCGGTCGAGTGGTCTCAGTTGGAGATGGGATTGCAC
Silene latifolia                  TCAAGTGGATGAGATCGGTCGAGTGGTCTCAGTTGGAGATGGGATTGCAC
PB235_atp1                        TCAAGTGGATGAGATCGGTCGAGTGGTCTCAGTTGGAGATGGGATTGCAC
RRIT251_atp1                      TCAAGTGGATGAGATCGGTCGAGTGGTCTCAGTTGGAGATGGGATTGCAC
Citrullus lanatus                 TCAAGTGGATGAGATCGGTCGAGTGGTCTCAGTTGGAGATGGGATTGCAC
Cucurbita pepo                    GAAAGTGGATGAGATCGGTCGAGTGGTCTCAGTTGGAGATGGTATTGCAC
RRIC110_atp1                      TCAAGTGGATGAGATCGGTCGAGTGGTCTCAGTTGGAGATGGGATTGCAC
Oryza sativa Japonica             TCAAGTGGATGAGATCGGTCGAGTGGTCTCAGTTGGAGATGGGATTGCAC
Cycas taitungensis                ACAAGTTGATGGGATCGGTCGAGTGGTCCCAGTTGGGGATGGGATTGCAC
Anomodon rugelii                  GCAAGTGGATGAGATCGGTCGAGTGGTATCAGTTGGAGATGGAATTGCAC
Anomodon attenuatus               GCAAGTGGATGAGATCGGTCGAGTGGTATCAGTTGGAGATGGAATTGCAC
Megaceros aenigmaticus            GCAAGTGGATGAGATCGGTCGAGTGGTATCAGTTGGAGATGGAATTGCAC
Phaeoceros laevis                 GCAAGTGGATGAGATTGGTCGAGTGGTATCAGTTGGAGATGGAATTGCAC
                                    **** **** *** ***********  ******* ***** *******

Lotus japonicus                   GTGTTTATGGATTGAACGAGATTCAAGCTGGGGAAATGGTTGAATTTGCC
BPM24_atp1                        GTGTTTATGGATTAAACGAGATTCAAGCTGGGGAAATGGTTGAATTTGCC
Millettia pinnata                 GTGTTTATGGATTGAACGAGATTCAAGCTGGGGAAATGGTTGAATTTGCC
RRIM600_atp1                      GTGTTTATGGATTAAACGAGATTCAAGCTGGGGAAATGGTTGAATTTGCC
Arabidopsis thaliana              AAGTTTATGGATTGAACGAGATTCAAGCTGGGGAAATGGTTCTTTTTGCC
Brassica napus                    AAGTTTATGGATTGAACGAGATTCAAGCTGGGGAAATGGTTCTTTTTGCC
RRII105_atp1                      GTGTTTATGGATTAAACGAGATTCAAGCTGGGGAAATGGTTGAATTTGCC
Beta macrocarpa                   GTGTTTATGGATTGAACGAGATTCAAGCTGGGGAAATGGTGGAATTTGCC
Beta vulgaris vulgaris            GTGTTTATGGATTGAACGAGATTCAAGCTGGGGAAATGGTGGAATTTGCC
Beta vulgaris maritima            GTGTTTATGGATTGAACGAGATTCAAGCTGGGGAAATGGTGGAATTTGCC
Silene latifolia                  GTGTTTATGGATTGAACGAGATTCAAGCTGGAGAAATGGTGGAATTTGCC
PB235_atp1                        GTGTTTATGGATTAAACGAGATTCAAGCTGGGGAAATGGTTGAATTTGCC
RRIT251_atp1                      GTGTTTATGGATTAAACGAGATTCAAGCTGGGGAAATGGTTGAATTTGCC
Citrullus lanatus                 GTGTTTATGGATTGAAGGAGATTCAAGCTGGGGAAATGGTGGAATTTGCC
Cucurbita pepo                    GTGTTTATGGATTGAAGGAGATTCAAGCTGGGGAAATGGTGGAATTTGCC
RRIC110_atp1                      GTGTTTATGGATTAAACGAGATTCAAGCTGGGGAAATGGTTGAATTTGCC
Oryza sativa Japonica             GTGTTTATGGATTGAACGAGATTCAAGCTGGAGAAATGGTGGAATTTGCC
Cycas taitungensis                GTGTTTATGGATCAAACGAGATTCAAGCTGGGGAAATGGTTGAATTCGCC
Anomodon rugelii                  GTGTTTATGGATTAAACAAGATTCAAGCTGGAGAAATGGTTGAATTTGCC
Anomodon attenuatus               GTGTTTATGGATTAAACAAGATTCAAGCTGGAGAAATGGTTGAATTTGCC
Megaceros aenigmaticus            GTGTTTATGGATCGAACAAGATTCAGGCAGGGGAAATGGTCGAATTTGCC
Phaeoceros laevis                 GTGTTTATGGATTGAACAAGATTCAGGCAGGGGAAATGGTCGAATTTGCC
                                    **********  **  *******  ** ** ********    ** ***

Lotus japonicus                   AGCGGTGTGAAAGGAATAGCGTTGAATCTTGAGAATGAGAATGTCGGAAT
BPM24_atp1                        AGCGGTGTGAAAGGAATAGCGTTGAATCTTGAGAATGAGAATGTAGGGAT
Millettia pinnata                 AGCGGTGTGAAAGGAGTAGCGTTGAATCTTGAGAATGAGAATGTCGGAAT
```

```
RRIM600_atp1                      AGCGGTGTGAAAGGAATAGCGTTGAATCTTGAGAATGAGAATGTAGGGAT
Arabidopsis thaliana              AACGGTGTGAAAGGAATGGCCTTGAATCTTGAGAATGAGAATGTCGGGAT
Brassica napus                    AACGGTGTGAAAGGAATGGCCTTGAATCTTGAGAATGAGAATGTCGGGAT
RRII105_atp1                      AGCGGTGTGAAAGGAATAGCGTTGAATCTTGAGAATGAGAATGTAGGGAT
Beta macrocarpa                   AGCGGTGTGAAAGGAATAGCCTTAAATCTTGAGAATGAGAATGTAGGGAT
Beta vulgaris vulgaris            AGCGGTGTGAAAGGAATAGCCTTAAATCTTGAGAATGAGAATGTAGGGAT
Beta vulgaris maritima            AGCGGTGTGAAAGGAATAGCCTTAAATCTTGAGAATGAGAATGTAGGGAT
Silene latifolia                  AGCGGTGTGAAAGGAATAGCCTTAAATCTTGAGAATGAAAATGTAGGGAT
PB235_atp1                        AGCGGTGTGAAAGGAATAGCGTTGAATCTTGAGAATGAGAATGTAGGGAT
RRIT251_atp1                      AGCGGTGTGAAAGGAATAGCGTTGAATCTTGAGAATGAGAATGTAGGGAT
Citrullus lanatus                 AGCGGTGTAAAAGGAATAGCCTTAAATCTTGAGAATGAGAATGTAGGGAT
Cucurbita pepo                    AGCGGTGTCAAAGGAATAGCCTTAAATCTTGAGAATGAGAATGTAGGGAT
RRIC110_atp1                      AGCGGTGTGAAAGGAATAGCGTTGAATCTTGAGAATGAGAATGTAGGGAT
Oryza sativa Japonica             AGCGGTGTGAAAGGAATAGCCTTGAATCTTGAGAATGAGAATGTAGGTAT
Cycas taitungensis                AGCGGTGTGAAAGGAATAGCGTTGAATCCTGAGAATGAGAATGTAGGTAT
Anomodon rugelii                  AGCAGTGTGAAAGGAATGGCTTTGAATCTAGAAAATGAGAATGTAGGAAT
Anomodon attenuatus               AGCAGTGTGAAAGGAATGGCTTTGAATCTAGAAAATGAGAATGTAGGAAT
Megaceros aenigmaticus            AGCGGTGTGAAAGGAATGGCTTTGAATCTAGAGAAGGAAAATGTAGGAAT
Phaeoceros laevis                 AGCGGTGTGAAAGGAATGGCTTTGAATCTAGAAAAGGAAAATGTAGGAAT
                                  * * **** ****** * ** ** ****  ** ** ** ***** ** **

Lotus japonicus                   TGTTGTCTTTGGTAGTGATACCGCTATCAAAGAAGGAGATCTTGTCAAAC
BPM24_atp1                        TGTTGTCTTTGGTAGTGATACCGCTATTAAAGAAGGAGATCTTGTCAAGC
Millettia pinnata                 TATTGTCTTTGGTAGTGATACCGCTATAAAAGAAGGAGATCTTGTCAAAC
RRIM600_atp1                      TGTTGTCTTTGGTAGTGATACCGCTATTAAAGAAGGAGATCTTGTCAAGC
Arabidopsis thaliana              TGTTGTCTTTGGTGGTGATACCGCTATAAAAGAAGGAGATCTTGTCAAGC
Brassica napus                    TGTTGTCTTTGGTGGTGATACCGCTATAAAAGAAGGAGATCTTGTCAAGC
RRII105_atp1                      TGTTGTCTTTGGTAGTGATACCGCTATTAAAGAAGGAGATCTTGTCAAGC
Beta macrocarpa                   TGTTGTCTTTGGTAGTGATACCGCTATTAAAGAGGGAGATCTTGTCAAGC
Beta vulgaris vulgaris            TGTTGTCTTTGGTAGTGATACCGCTATTAAAGAGGGAGATCTTGTCAAGC
Beta vulgaris maritima            TGTTGTCTTTGGTAGTGATACCGCTATTAAAGAGGGAGATCTTGTCAAGC
Silene latifolia                  TGTTGTCTTTGGTAGTGATACCGCTATTAAAGAGGGCGATCTTGTCAAGC
PB235_atp1                        TGTTGTCTTTGGTAGTGATACCGCTATTAAAGAAGGAGATCTTGTCAAGC
RRIT251_atp1                      TGTTGTCTTTGGTAGTGATACCGCTATTAAAGAAGGAGATCTTGTCAAGC
Citrullus lanatus                 TGTTGTCTTTGGTAGTGATACUGCTATTAAAGAAGGAGATCTTGTCAAGC
Cucurbita pepo                    TGTTGTCTTTGGTAGTGATACCGCTATTAAAGAAGGAGATCTTGTCAAGC
RRIC110_atp1                      TGTTGTCTTTGGTAGTGATACCGCTATTAAAGAAGGAGATCTTGTCAAGC
Oryza sativa Japonica             TGTTGTCTTTGGTAGTGATACCGCTATTAAAGAAGGAGATCTTGTCAAGC
Cycas taitungensis                TGTTGTATCTGGTAGTGATACCGCCATTAAAGAAGGAGATCTTGTCAAGC
Anomodon rugelii                  TGTTATATTTGGTAGTGATACTGCTATTAAAGAAGGAGACATTGTCAAGC
Anomodon attenuatus               TGTTATATTTGGTAGTGATACTGCTATTAAAGAAGGAGACATTGTCAAGC
Megaceros aenigmaticus            TGTTGTATTTGGTAGTGATACCGCTATTAAAGAAGGAGATATTGTAAGGC
Phaeoceros laevis                 TGTTGTATTTGGTAGTGATACCGCTATTAAAGAAGGAGATATTGTAAGGC
                                  * ** * * **** ******* ** ** ***** ** **  **** *  *

Lotus japonicus                   GCACTGGATCTATTGTGGATGTTCCTGCGGGAAAGGCTATGCTAGGGCGT
BPM24_atp1                        GCACTGGATCTATTGTGGATGTTCCTGCGGGAAAGGCTATGCTAGGGCGT
Millettia pinnata                 GCACTGGATCTATTGTGGATGTTCCTGCGGGAAAGGCTATGCTAGGGCGT
RRIM600_atp1                      GCACTGGATCTATTGTGGATGTTCCTGCGGGAAAGGCTATGCTAGGGCGT
Arabidopsis thaliana              GCACTGGATCTATTGTGGATGTTCCCGCGGGAAAGGCTATGCTAGGGCGT
Brassica napus                    GCACTGGATCTATTGTGGATGTTCCCGCGGGAAAGGCTATGCTAGGGCGT
RRII105_atp1                      GCACTGGATCTATTGTGGATGTTCCTGCGGGAAAGGCTATGCTAGGGCGT
Beta macrocarpa                   GCACTGGATCTATTGTGGATGTTCCTGCGGGAAAGGCTATGCTAGGGCGT
Beta vulgaris vulgaris            GCACTGGATCTATTGTGGATGTTCCTGCGGGAAAGGCTATGCTAGGGCGT
Beta vulgaris maritima            GCACTGGATCTATTGTGGATGTTCCTGCGGGAAAGGCTATGCTAGGGCGT
Silene latifolia                  GCACTGGATCTATTGTGGATGTTCCTGCGGGAAAAGCTATGCTAGGGCGT
PB235_atp1                        GCACTGGATCTATTGTGGATGTTCCTGCGGGAAAGGCTATGCTAGGGCGT
RRIT251_atp1                      GCACTGGATCTATTGTGGATGTTCCTGCGGGAAAGGCTATGCTAGGGCGT
Citrullus lanatus                 GCACTGGATCGATTGTGGATGTTCCCGCGGGAAAGGCTATGCTCGGGCGT
Cucurbita pepo                    GCACTGGATCGATTGTGGATGTTCCTGCGGGAAAGGCTATGCTCGGGCGT
RRIC110_atp1                      GCACTGGATCTATTGTGGATGTTCCTGCGGGAAAGGCTATGCTAGGGCGT
Oryza sativa Japonica             GCACTGGATCTATTGTGGATGTTCCTGCGGGAAAGGCCATGTTAGGCCGT
```

```
Cycas taitungensis                GCACTGGATCTATTGTGGATGTTCCTGTGGGAAAGGCCATGTTAGGTCGT
Anomodon rugelii                  GCACTGGATCTATTGTTGATGTTCCTGTAGGAAAGGCCTTGTTAGGTCGT
Anomodon attenuatus               GCACTGGATCTATTGTTGATGTTCCTGTAGGAAAGGCCTTGTTAGGTCGT
Megaceros aenigmaticus            GCACTGGATCTATTGTGGATGTTCCTGTAGGAAAGGCCATATTAGGTCGT
Phaeoceros laevis                 GCACTGGATCTATTGTGGATGTTCCTGTAGGAAAGGCCATATTAGGTCGT
                                  ********** ***** ******** *  ***** **  *  * ** ***

Lotus japonicus                   GTGGTCGACGCATTGGGAGTACCTATTGATGGAAGAGGGGCTCTAAGCGA
BPM24_atp1                        GTGGTCGACGCGTTGGGAGTACCTATTGATGGAAGAGGGGCTCTAAGCGA
Millettia pinnata                 GTGGTCGACGCGTTGGGAGTACCTATTGATGGAAGAGGGGCTCTAAGCGA
RRIM600_atp1                      GTGGTCGACGCGTTGGGAGTACCTATTGATGGAAGAGGGGCTCTAAGCGA
Arabidopsis thaliana              GTGGTCGACGCGATGGGAGTACCTATTGATGGAAAAGGGGCTCTAAGCGA
Brassica napus                    GTGGTCGACGCGATGGGAGTACCTATTGATGGAAGAGGGGCTCTAAGCGA
RRII105_atp1                      GTGGTCGACGCGTTGGGAGTACCTATTGATGGAAGAGGGGCTCTAAGCGA
Beta macrocarpa                   GTGGTCGACGCGTTGGGAGTACCTATTGATGGAAGAGGGGCTCTAAGCGA
Beta vulgaris vulgaris            GTGGTCGACGCGTTGGGAGTACCTATTGATGGAAGAGGGGCTCTAAGCGA
Beta vulgaris maritima            GTGGTCGACGCGTTGGGAGTACCTATTGATGGAAGAGGGGCTCTAAGCGA
Silene latifolia                  GTGGTCGACGCGTTGGGAGTACCTATTGATGGAAGAGGGGCTCTAAGCGA
PB235_atp1                        GTGGTCGACGCGTTGGGAGTACCTATTGATGGAAGAGGGGCTCTAAGCGA
RRIT251_atp1                      GTGGTCGACGCGTTGGGAGTACCTATTGATGGAAGAGGGGCTCTAAGCGA
Citrullus lanatus                 GTGGTCGACGCGTTGGGAGTACCTATTGATGGAAGAGGGGCTCTAAGCGA
Cucurbita pepo                    GTGGTGGACGCGTTGGGAGTACCTATTGATGGAAGAGGGGCTCTAAGCGA
RRIC110_atp1                      GTGGTCGACGCGTTGGGAGTACCTATTGATGGAAGAGGGGCTCTAAGCGA
Oryza sativa Japonica             GTGGTCGACGCCTTGGGAGTACCTATTGATGGAAAAGGGGCTCTAAGCGA
Cycas taitungensis                GTGGTCGATGCGTTGGGAGTACCTATTGATGGAAAAGGGGCTTTAAGCGA
Anomodon rugelii                  GTGGTTGATGCCTTAGGAGTACCTATTGATGGAAAAGGTGCTTTAAGCAC
Anomodon attenuatus               GTGGTTGATGCCTTAGGAGTACCTATTGATGGAAAAGGTGCTTTAAGCAC
Megaceros aenigmaticus            GTAGTTGATGCGTTAGGAGTACCTATTGATGGAAAAGGTGCCTTAGGCGC
Phaeoceros laevis                 GTAGTTGATGCCTTAGGAGTACCTATTGATGGAAAAGGTGCCTTAGGCGC
                                  ** ** ** **  * ******************* *** **  ** **

Lotus japonicus                   TCACGAGCGAAGACGTGTCGAAGTGAAAGCCCCTGGGATTATAGAACGTA
BPM24_atp1                        TCACGAGCGAAGACGTGTCGAAGTGAAAGCCCCTGGGATTATTGAACGTA
Millettia pinnata                 TCACGAGCGAAGACGTGTCGACGTGAAAGCCCCTGGGATTATTGAACGTA
RRIM600_atp1                      TCACGAGCGAAGACGTGTCGAAGTGAAAGCCCCTGGGATTATTGAACGTA
Arabidopsis thaliana              TCACGAGCAAAGACGTGTCGAAGTGAAAGCCCCTGGGATTCTTGAACGTA
Brassica napus                    TCACGAGCAAAGACGTGTCGAAGTGAAAGCCCCTGGGATTCTTGAACGTA
RRII105_atp1                      TCACGAGCGAAGACGTGTCGAAGTGAAAGCCCCTGGGATTATTGAACGTA
Beta macrocarpa                   TCACGAGCGTCGACGTGTCGAAGTGAAAGCCCCCGGGATTATTGAACGTA
Beta vulgaris vulgaris            TCACGAGCGTCGACGTGTCGAAGTGAAAGCCCCCGGGATTATTGAACGTA
Beta vulgaris maritima            TCACGAGCGTCGACGTGTCGAAGTGAAAGCCCCCGGGATTATTGAACGTA
Silene latifolia                  TCACGAGCGTCGACGTGTCGAAGTGAAAGCCCCTGGGATTATTGAACGTA
PB235_atp1                        TCACGAGCGAAGACGTGTCGAAGTGAAAGCCCCTGGGATTATTGAACGTA
RRIT251_atp1                      TCACGAGCGAAGACGTGTCGAAGTGAAAGCCCCTGGGATTATTGAACGTA
Citrullus lanatus                 TCACGAGCGAAGACGTGTCGAAGTGAAAGCCCCTGGGATTATTGAACGTA
Cucurbita pepo                    TCACGAGCGAAGACGTGTCGAAGTGAAAGCCCCTGGGATTATTTCCCGTA
RRIC110_atp1                      TCACGAGCGAAGACGTGTCGAAGTGAAAGCCCCTGGGATTATTGAACGTA
Oryza sativa Japonica             TCACGAACGAAGACGTGTCGAAGTGAAAGCCCCAGGGATTATTGAACGTA
Cycas taitungensis                TCACGAACGAAGACGTGTCGAAGTGAAAGCCCCTGGGATTATTGAACGTA
Anomodon rugelii                  TGCAGAACGAAAGCGTGTAGAAGTTAAAGCTCCCGGGATTATTGCACGTA
Anomodon attenuatus               TGCAGAACGAAAGCGTGTAGAAGTTAAAGCTCCCGGGATTATTGCACGTA
Megaceros aenigmaticus            TGTAGAACGAAGACGTGTAGAAGTTAAAGCCCCTGGAATTATTGCACGCA
Phaeoceros laevis                 TGTAGAACGAAGACGTGTAGAAGTTAAAGCCCCTGGAATTATTGCACGCA
                                  *   ** *     ***** ** ** ***** ** ** *** *    ** *

Lotus japonicus                   AATCTGTGCACGAGCCTATGCAAACAGGGTTAAAAGCGGTAGATAGCCTG
BPM24_atp1                        AATCTGTGCACGAGCCTATGCAAACAGGGTTAAAAGCGGTAGATAGCTTG
Millettia pinnata                 AATCTGTGCACGAGCCTATGCAAACAGGGTTAAAAGCGGTAGATAGCCTG
RRIM600_atp1                      AATCTGTGCACGAGCCTATGCAAACAGGGTTAAAAGCGGTAGATAGCTTG
Arabidopsis thaliana              AATCAGTGCACGAGCCTATGCAAACAGGGTTAAAAGCGGTAGATAGCCTG
Brassica napus                    AATCAGTGCACGAGCCTATGCAAACAGGGTTAAAAGCGGTAGATAGCCTG
RRII105_atp1                      AATCTGTGCACGAGCCTATGCAAACAGGGTTAAAAGCGGTAGATAGCTTG
```

```
Beta macrocarpa                  AATCTGTGCACGAGCCTATGCAAACCGGGTTAAAGGCGGTAGATAGCCTG
Beta vulgaris vulgaris           AATCTGTGCACGAGCCTATGCAAACCGGGTTAAAGGCGGTAGATAGCCTG
Beta vulgaris maritima           AATCTGTGCACGAGCCTATGCAAACCGGGTTAAAGGCGGTAGATAGCCTG
Silene latifolia                 AATCAGTGCACGAGCCTATGCAAACCGGCTTAAAGGCGGTAGATAGCCTG
PB235_atp1                       AATCTGTGCACGAGCCTATGCAAACAGGGTTAAAAGCGGTAGATAGCTTG
RRIT251_atp1                     AATCTGTGCACGAGCCTATGCAAACAGGGTTAAAAGCGGTAGATAGCTTG
Citrullus lanatus                AATCAGTGCACGAGCCTATGCAAACAGGGTTAAAAGCGGTAGATAGCCTG
Cucurbita pepo                   AATCTGTGCACGAACCTATGCAAACTGGGTTAAAAGCGGTAGATAGCCTG
RRIC110_atp1                     AATCTGTGCACGAGCCTATGCAAACAGGGTTAAAAGCGGTAGATAGCTTG
Oryza sativa Japonica            AATCTGTGCACGAACCCATGCAAACAGGCTTAAAAGCAGTGGATAGCCTG
Cycas taitungensis               AATCTGTGCACGAACCTATGCAAACAGGGTTGAAAGCAGTGGATAGCCCG
Anomodon rugelii                 AATCTGTGCACGAACCCATGCAAACAGGATTAAAAGCAGTAGATAGCCTG
Anomodon attenuatus              AATCTGTGCACGAACCCATGCAAACAGGATTAAAAGCAGTAGATAGCCTG
Megaceros aenigmaticus           AATCCGTGCACGAACCAATGCAAACAGGATTAAAAGCGGTGGACAGCCTG
Phaeoceros laevis                AATCCGTGCACGAACTCATGCAAACAGGATTAAAAGCGGTAGACAGCCTG
                                 **** ******** *  ******** ** ** ** ** ** ** ***  *

Lotus japonicus                  GTTCCTATAGGCCGTGGTCAACGAGAACTTATAATCGGGGACCGACAAAC
BPM24_atp1                       GTTCCTATAGGCCGTGGTCAACGAGAACTTATAATCGGGGACCGACAAAC
Millettia pinnata                GTTCCTATAGGCCGTGGTCAACGAGAACTTATAATCGGGGACCGACAAAC
RRIM600_atp1                     GTTCCTATAGGCCGTGGTCAACGAGAACTTATAATCGGGGACCGACAAAC
Arabidopsis thaliana             GTTCCTATAGGCCGTGGTCAACGAGAACTTCTAATCGGTGGCCGACAAAC
Brassica napus                   GTTCCTATAGGCCGTGGTCAACGAGAACTTCTAATCGGGGACCGACAAAC
RRII105_atp1                     GTTCCTATAGGCCGTGGTCAACGAGAACTTATAATCGGGGACCGACAAAC
Beta macrocarpa                  GTTCCTATAGGCCGTGGTCAACGAGAACTTATAATCGGGGACCGACAAAC
Beta vulgaris vulgaris           GTTCCTATAGGCCGTGGTCAACGAGAACTTATAATCGGGGACCGACAAAC
Beta vulgaris maritima           GTTCCTATAGGCCGTGGTCAACGAGAACTTATAATCGGGGACCGACAAAC
Silene latifolia                 GTTCCTATAGGCCGTGGTCAACGAGAACTTATAATCGGGGACCGACAAAC
PB235_atp1                       GTTCCTATAGGCCGTGGTCAACGAGAACTTATAATCGGGGACCGACAAAC
RRIT251_atp1                     GTTCCTATAGGCCGTGGTCAACGAGAACTTATAATCGGGGACCGACAAAC
Citrullus lanatus                GTTCCTATAGGCCGTGGCCAACGAGAACTTATAATUGGAGACCGACAAAC
Cucurbita pepo                   GTTCCTATAGGCCGTGGCCAACGAGAACTTATAATCGGGGACCGACAAAC
RRIC110_atp1                     GTTCCTATAGGCCGTGGTCAACGAGAACTTATAATCGGGGACCGACAAAC
Oryza sativa Japonica            GTTCCTATAGGCCGTGGTCAACGAGAACTTATAATCGGGGACAGACAAAC
Cycas taitungensis               GTTCCTATAGGCCGTGGTCAACGAGAACCGATAATCGGGGACCGACAGAC
Anomodon rugelii                 GTTCCTATAGGTCGTGGTCAACGAGAACTTATAATAGGAGACAGACAAAC
Anomodon attenuatus              GTTCCTATAGGTCGTGGTCAACGAGAACTTATAATAGGAGACAGACAAAC
Megaceros aenigmaticus           GTTCCTATAGGTCGTGGTCAACGAGAACCTATAATAGGAGACAGACAAAC
Phaeoceros laevis                GTTCCTACAGGTCGTGGTCAACGAGAATTTATAATAGGAGACAGACAAAC
                                 ******* *** ***** *********    **** ** * * **** **

Lotus japonicus                  TGGAAAAACAGCTATAGCTATCGATACCATATTAAACCAAAAGCAAATGA
BPM24_atp1                       TGGAAAAACAGCTATTGCTATCGATACCATATTAAACCAAAAGCAAATGA
Millettia pinnata                TGGAAAAACAGCTATTGCTATCGATACCATATTAAACCAAAAGCAAATGA
RRIM600_atp1                     TGGAAAAACAGCTATTGCTATCGATACCATATTAAACCAAAAGCAAATGA
Arabidopsis thaliana             TGGAAAAACGACTATTGCTATCGATACCATATTAAACCAAAAGCAAATAA
Brassica napus                   TGGAAAAACGACTATTGCTATCGATACCATATTAAACCAAAAGCAAATAA
RRII105_atp1                     TGGAAAAACAGCTATTGCTATCGATACCATATTAAACCAAAAGCAAATGA
Beta macrocarpa                  GGGAAAAACAGCTATTGCTATCGATACCATATTAAACCAAAAGCAACTGA
Beta vulgaris vulgaris           GGGAAAAACAGCTATTGCTATCGATACCATATTAAACCAAAAGCAACTGA
Beta vulgaris maritima           GGGAAAAACAGCTATTGCTATCGATACCATATTAAACCAAAAGCAACTGA
Silene latifolia                 GGGAAAAACAGCTATTGCTATCGATACCATATTAAACCAAAAGCAACTGA
PB235_atp1                       TGGAAAAACAGCTATTGCTATCGATACCATATTAAACCAAAAGCAAATGA
RRIT251_atp1                     TGGAAAAACAGCTATTGCTATCGATACCATATTAAACCAAAAGCAAATGA
Citrullus lanatus                TGGAAAAACAGCTATAGCTATCGATACCATATTAAACCAAAAGCAAATGA
Cucurbita pepo                   TGGAAAAACTGCTATAGCTATCGATACCATATTAAACCAAAAGCAAATGA
RRIC110_atp1                     TGGAAAAACAGCTATTGCTATCGATACCATATTAAACCAAAAGCAAATGA
Oryza sativa Japonica            TGGAAAAACAGCAATAGCTATCGATACTATATTAAACCAAAAGCAAATGA
Cycas taitungensis               TGGAAAAACTGCTATAGCTATTGATACCATATCGAACCAGAAGCGAATGA
Anomodon rugelii                 TGGAAAAACTGCTATCGCTATTGATACCATATTGAACCAGAAGCAAATCA
Anomodon attenuatus              TGGAAAAACTGCTATCGCTATTGATACCATATTGAACCAGAAGCAAATCA
Megaceros aenigmaticus           TGGAAAGACTGCTGTAGCTATTGATACCATATCGAACCAGAAAGAAATCA
```

```
Phaeoceros laevis                 TGGAAAGACTGCTGTAGCTATTGATACCATATCGAACCAAAAAGAAATAA
                                   ***** **  *  * ***** ***** ****  ***** **   * * *

Lotus japonicus                   ACTCAAGGGCCACCTCTGAGAGTGAGACATTGTATTGTGTCTATGTAGCG
BPM24_atp1                        ACTCAAGGGCCACCTCTGAGAGTGAGACATTGTATTGTGTCTATGTAGCG
Millettia pinnata                 ACTCAAGGGCCACCTCTGAGAGTGAGACATTGTATTGTGTCTATGTAGCG
RRIM600_atp1                      ACTCAAGGGCCACCTCTGAGAGTGAGACATTGTATTGTGTCTATGTAGCG
Arabidopsis thaliana              ACTCAAGGGCCACCTCTGAGAGTGAGACAATGTATTGTGTCTATGTAGCG
Brassica napus                    ACTCAAGGGCCACCTCTGAGAGTGAGACAATGTATTGTGTCTATGTAGCG
RRII105_atp1                      ACTCAAGGGCCACCTCTGAGAGTGAGACATTGTATTGTGTCTATGTAGCG
Beta macrocarpa                   ACTCAAAGGCCACCTCTGAGAGTGAGACATTGTATTGTGTCTATGTAGCG
Beta vulgaris vulgaris            ACTCAAAGGCCACCTCTGAGAGTGAGACATTGTATTGTGTCTATGTAGCG
Beta vulgaris maritima            ACTCAAAGGCCACCTCTGAGAGTGAGACATTGTATTGTGTCTATGTAGCG
Silene latifolia                  ACTCAAAGGCCACTTCTGAGAGTGAGACATTGTATTGTGTTTATGTAGCG
PB235_atp1                        ACTCAAGGGCCACCTCTGAGAGTGAGACATTGTATTGTGTCTATGTAGCG
RRIT251_atp1                      ACTCAAGGGCCACCTCTGAGAGTGAGACATTGTATTGTGTCTATGTAGCG
Citrullus lanatus                 ACTCAAGGGCCACCTCTGAGAGTGAGACATTGTATTGTGTCTATGTAGCG
Cucurbita pepo                    ACTCAAGGGCCACCTCTGAGAGTGAGACATTGTATTGTGTCTATGTAGCG
RRIC110_atp1                      ACTCAAGGGCCACCTCTGAGAGTGAGACATTGTATTGTGTCTATGTAGCG
Oryza sativa Japonica             ACTCAAGGGGCACAAATGAGAGTGAGACATTGTATTGTGTCTATGTTGCG
Cycas taitungensis                ACTCAAAGGGCACCTCTGATAGTGAAAAATTGTATCGTGTCTATGTAGCG
Anomodon rugelii                  ACACACAGGGCACCTCGGATAGTGAAAAATTGTATTGTGTGTATGTAGCG
Anomodon attenuatus               ACACACAGGGCACCTCGGATAGTGAAAAATTGTATTGTGTGTATGTAGCG
Megaceros aenigmaticus            ACGCACAGGGCACCTCTAATAGTGAGAAATTGTATTGTGTGTATGTAGCA
Phaeoceros laevis                 ACGCACAAGGCACCTCTAATAGTGATAAATTGTATTGTGTGTATGTAGCA
                                  ** **   * ***     * ***** * * ***** **** ***** **

Lotus japonicus                   ATTGGACAGAAACGCTCAACTGTGGCACAATTAGTTCAAATTCTTTCAGA
BPM24_atp1                        ATTGGACAGAAACGCTCAACTGTGGCACAATTAGTTCAAATTCTTTCAGA
Millettia pinnata                 ATTGGACAGAAACGCTCAACTGTGGCACAATTAGTTCAAATTCTTTCAGA
RRIM600_atp1                      ATTGGACAGAAACGCTCAACTGTGGCACAATTAGTTCAAATTCTTTCAGA
Arabidopsis thaliana              ATTGGACAGAAACGCTCGACTGTGGGACAATTAATTCAAACTCTTGAAGA
Brassica napus                    ATTGGACAGAAACGCTCGACTGTGGGACAATTAATTCAAACTCTTGAAGA
RRII105_atp1                      ATTGGACAGAAACGCTCAACTGTGGCACAATTAGTTCAAATTCTTTCAGA
Beta macrocarpa                   GTTGGACAGAAACGTTCAACTGTGGCACAATTAGTTCAAATTCTTTCAGA
Beta vulgaris vulgaris            GTTGGACAGAAACGTTCAACTGTGGCACAATTAGTTCAAATTCTTTCAGA
Beta vulgaris maritima            GTTGGACAGAAACGTTCAACTGTGGCACAATTAGTTCAAATTCTTTCAGA
Silene latifolia                  ATTGGACAGAAACGTTCAACTGTGGCACAATTAGTTCAAATTCTTTCAGA
PB235_atp1                        ATTGGACAGAAACGCTCAACTGTGGCACAATTAGTTCAAATTCTTTCAGA
RRIT251_atp1                      ATTGGACAGAAACGCTCAACTGTGGCACAATTAGTTCAAATTCTTTCAGA
Citrullus lanatus                 ATTGGACAGAAACGCTCAACTGTGGCACAATTAGTTCAAATTCTTTCAGA
Cucurbita pepo                    ATTGGACAGAAACGCTCAACTGTGGCACAATTAGTTCAAATTCTTTCTTC
RRIC110_atp1                      ATTGGACAGAAACGCTCAACTGTGGCACAATTAGTTCAAATTCTTTCAGA
Oryza sativa Japonica             ATTGGACAAAAACGCTCGACTGTGGCACAATTAGTTCAAATTCTTTCAGA
Cycas taitungensis                ATTGGACAGAAACGCTCAACCGCGGCACAATTAGTTCAAATTCTTTCAGG
Anomodon rugelii                  ATTGGACAGAAACGTTCAACCGTGGCACAATTAGTTAAGATTCTTTCAGA
Anomodon attenuatus               ATTGGACAGAAACGTTCAACCGTGGCACAATTAGTTAAGATTCTTTCAGA
Megaceros aenigmaticus            ATTGGACAGAAACGTTCAACCGTAGCACAATTGGTTAAGACCCTTTCAGA
Phaeoceros laevis                 ATTGGACAGAAACGTTCAACCGTAGCACAATTGGTTAAGATTCTTTCAGA
                                   ******* ***** ** ** *  * ******  ** * *  ***

Lotus japonicus                   AGCGAATGCTTTAGAATATTCCATTCTTGTAGCAGCCACCGCTTCGGATC
BPM24_atp1                        AGCGAATGCTTTAGAATATTCCATTCTTGTAGCAGCCACCGCCTCGGATC
Millettia pinnata                 AGCGAATGCTTTAGAATATTCCATTCTTGTAGCAGCCACCGCTTCGGATC
RRIM600_atp1                      AGCGAATGCTTTAGAATATTCCATTCTTGTAGCAGCCACCGCCTCGGATC
Arabidopsis thaliana              AGCGAATGCTTTGGAATATTCCATTCTTGTAGCAGCCACCGCTTCGGATC
Brassica napus                    AGCGAATGCTTTGGAATATTCCATTCTTGTAGCAGCCACCGCTTCGGATC
RRII105_atp1                      AGCGAATGCTTTAGAATATTCCATTCTTGTAGCAGCCACCGCCTCGGATC
Beta macrocarpa                   AGCGAATGCTTTGGAATATTCCATTCTTGTAGCAGCCACCGCTTCGGATC
Beta vulgaris vulgaris            AGCGAATGCTTTGGAATATTCCATTCTTGTAGCAGCCACCGCTTCGGATC
Beta vulgaris maritima            AGCGAATGCTTTGGAATATTCCATTCTTGTAGCAGCCACCGCTTCGGATC
Silene latifolia                  AGCGAATGCTTTGGAATATTCCATTCTTGTAGCAGCCACTGCTTCGGATC
```

```
PB235_atp1                        AGCGAATGCTTTAGAATATTCCATTCTTGTAGCAGCCACCGCCTCGGATC
RRIT251_atp1                      AGCGAATGCTTTAGAATATTCCATTCTTGTAGCAGCCACCGCCTCGGATC
Citrullus lanatus                 AGGGAATGCTTTGGAATATTCCATTCTTGTAGCAGCCACCGCTTCGGATC
Cucurbita pepo                    AGGGAATGCTTTGGAATATTCCATTCTTGTAGCAGCCACCGCTTCGGATC
RRIC110_atp1                      AGCGAATGCTTTAGAATATTCCATTCTTGTAGCAGCCACCGCCTCGGATC
Oryza sativa Japonica             AGCGAATGCTTTGGAATATTCCATTCTTGTAGCAGCCACCGCTTCGGATC
Cycas taitungensis                AGCGGATGCTTCGGAATATCCAATTATCGTAGCAGCCACCGCTTCGGATC
Anomodon rugelii                  AGCGGGTGCTTTAGAATATTGCATTATTGTAGCAGCTACTGCTTCGGATC
Anomodon attenuatus               AGCGGGTGCTTTAGAATATTGCATTATTGTAGCAGCTACTGCTTCGGATC
Megaceros aenigmaticus            AGCGGGTGCTTTGGAATATTGCATTATTCTAGCAGCCAGTGCTTCAGATC
Phaeoceros laevis                 AGCGGGTGCTTTAGAATATTGCATTATTCTAGCAGCCAGTGTTTTAGATC
                                  ** *  *****  ******   *** *  ******* *  *  *  ****

Lotus japonicus                   CTGCACCTCTGCAATTTCTGGCCCCATATTCTGGGTGTGCCATGGGGGAA
BPM24_atp1                        CTGCTCCTCTGCAATTTCTGGCCCCATATTCTGGGTGTGCCATGGGGGAA
Millettia pinnata                 CTGCACCTCTGCAATATCTGGCCCCATATTCTGGGTGTGCCATGGGGGAA
RRIM600_atp1                      CTGCTCCTCTGCAATTTCTGGCCCCATATTCTGGGTGTGCCATGGGGGAA
Arabidopsis thaliana              CTGCTCCTCTGCAATTTTTGGCCCCATATTCCGGGTGTGCCATGGGGGAA
Brassica napus                    CTGCTCCTCTGCAATTTTTGGCCCCATATTCCGGGTGTGCCATGGGGGAA
RRII105_atp1                      CTGCTCCTCTGCAATTTCTGGCCCCATATTCTGGGTGTGCCATGGGGGAA
Beta macrocarpa                   CTGCTCCTCTTCAATTTCTGGCCCCATATTCTGGGTGTGCTATGGGAGAA
Beta vulgaris vulgaris            CTGCTCCTCTTCAATTTCTGGCCCCATATTCTGGGTGTGCTATGGGAGAA
Beta vulgaris maritima            CTGCTCCTCTTCAATTTCTGGCCCCATATTCTGGGTGTGCTATGGGAGAA
Silene latifolia                  CTGCTCCTCTTCAATTTCTGGCCCCATATTCTGGATGTGCTATGGGAGAA
PB235_atp1                        CTGCTCCTCTGCAATTTCTGGCCCCATATTCTGGGTGTGCCATGGGGGAA
RRIT251_atp1                      CTGCTCCTCTGCAATTTCTGGCCCCATATTCTGGGTGTGCCATGGGGGAA
Citrullus lanatus                 CTGCTCCTCTTCAATTTCTGGCCCCATATTCTGGGTGTGCCATGGGGGAA
Cucurbita pepo                    CTGCTCCTCTTCAATTTCTGGCCCCATATTCTGGGTGTGCCATGGGGGAA
RRIC110_atp1                      CTGCTCCTCTGCAATTTCTGGCCCCATATTCTGGGTGTGCCATGGGGGAA
Oryza sativa Japonica             CTGCTCCTCTGCAATTTCTGGCCCCATATTCAGGGTGTGCCATGGGGGAA
Cycas taitungensis                CTGCTCCTCTGCAATTTCTGGCCCCATATCCTGGGTGTGCTGCGGGAGAA
Anomodon rugelii                  CTGCTCCTCTGCAATTCCTGGCACCATATTCAGGTTGCGCTATGGGAGAG
Anomodon attenuatus               CTGCTCCTCTGCAATTCCTGGCACCATATTCAGGTTGCGCTATGGGAGAG
Megaceros aenigmaticus            ATGCTCCTTTACAATTTCTTGCACCCTATTCAGGTTGCGCGATGGGAGAA
Phaeoceros laevis                 ATGTTCCTTTACAATTCCTTGCACCCTATTCAGGTTGTGCGATGGGAGAA
                                   **  *** * ****   * ** ** *** * ** ** **   *** **

Lotus japonicus                   TATTTCCGCGATAATGGAATGCACGCATTAATAATCTATGATGATCTTAG
BPM24_atp1                        TATTTCCGCGATAATGGAATGCACGCATTAATAATCTATGATGATCTTAG
Millettia pinnata                 TATTTCCGCGATAATGGAATGCACGCATTAATAATCTATGATGATCTTAG
RRIM600_atp1                      TATTTCCGCGATAATGGAATGCACGCATTAATAATCTATGATGATCTTAG
Arabidopsis thaliana              TATTTCCGCGATAATGGAATGCACGCATTAATAATCTATGATGATCTTAG
Brassica napus                    TATTTCCGCGATAATGGAATGCACGCATTAATAATCTATGATGATCTTAG
RRII105_atp1                      TATTTCCGCGATAATGGAATGCACGCATTAATAATCTATGATGATCTTAG
Beta macrocarpa                   TATTTCCGCGATAATGGAATGCACGCATTAATAATCTATGATGATCTTAG
Beta vulgaris vulgaris            TATTTCCGCGATAATGGAATGCACGCATTAATAATCTATGATGATCTTAG
Beta vulgaris maritima            TATTTCCGCGATAATGGAATGCACGCATTAATAATCTATGATGATCTTAG
Silene latifolia                  TATTTCCGCGATAATGGAATGCACGCATTAATAATCTATGATGATCTTAG
PB235_atp1                        TATTTCCGCGATAATGGAATGCACGCATTAATAATCTATGATGATCTTAG
RRIT251_atp1                      TATTTCCGCGATAATGGAATGCACGCATTAATAATCTATGATGATCTTAG
Citrullus lanatus                 TATTTCCGCGATAATGGAATGCACGCATTAATAATCTATGATGATCTTAG
Cucurbita pepo                    TATTTCCGGGATAATGGAATGCACGCATTAATAATCTATGATGATCTTAG
RRIC110_atp1                      TATTTCCGCGATAATGGAATGCACGCATTAATAATCTATGATGATCTTAG
Oryza sativa Japonica             TATTTCCGCGATAATGGAATGCACGCATTAATTATATATGATGATCTAAG
Cycas taitungensis                TATTTCCGCGATAATGGAATGCACGCATCAATAATCTATGATGATCCTAG
Anomodon rugelii                  TATTTTCGAGATAATGGAATGCACGCATTAATCATTTATGATGACCTTTC
Anomodon attenuatus               TATTTTCGAGATAATGGAATGCACGCATTAATCATTTATGATGACCTTTC
Megaceros aenigmaticus            TATTTTCGAGATAATGGGATGCACGCATTAATCATCTATGATGATCTGAG
Phaeoceros laevis                 TATTTTCGAGATAATGGAATGCACGCATTAATCATCTATGATGATCTGAG
                                  ***** ** ******** ********** *** ** ******** *

Lotus japonicus                   TAAACAGGCCGTGGCATATCGACAAATGTCATTATTGTTACGCCGACCAC
```

```
BPM24_atp1                        TAAACAGGCCGTGGCATATCGACAAATGTCATTATTGTTACGCCGACCAC
Millettia pinnata                 TAAACAGGCCGTGGCATATCGACAAATGTCATTATTGTTACGCCGACCAC
RRIM600_atp1                      TAAACAGGCCGTGGCATATCGACAAATGTCATTATTGTTACGCCGACCAC
Arabidopsis thaliana              TAAACAGGCGGTGGCATATCGACAAATGTCATTATTGTTACGCCGACCAC
Brassica napus                    TAAACAGGCGGTGGCATATCGACAAATGTCATTATTGTTACGCCGACCAC
RRII105_atp1                      TAAACAGGCCGTGGCATATCGACAAATGTCATTATTGTTACGCCGACCAC
Beta macrocarpa                   TAAACAGGCGGTGGCATATCGACAAATGTCATTATTGTTACGCCGACCAC
Beta vulgaris vulgaris            TAAACAGGCGGTGGCATATCGACAAATGTCATTATTGTTACGCCGACCAC
Beta vulgaris maritima            TAAACAGGCGGTGGCATATCGACAAATGTCATTATTGTTACGCCGACCAC
Silene latifolia                  TAAACAGGCGGTGGCATATCGACAAATGTCATTATTGTTACGCCGACCAC
PB235_atp1                        TAAACAGGCCGTGGCATATCGACAAATGTCATTATTGTTACGCCGACCAC
RRIT251_atp1                      TAAACAGGCCGTGGCATATCGACAAATGTCATTATTGTTACGCCGACCAC
Citrullus lanatus                 TAAACAGGCGGTGGCATATCGACAAATGTCATTATTGTTACGCCGACCAC
Cucurbita pepo                    TAAACAGGCGGTGGCATATCGACAAATGTCATTATTGTTACGCCGACCAC
RRIC110_atp1                      TAAACAGGCCGTGGCATATCGACAAATGTCATTATTGTTACGCCGACCAC
Oryza sativa Japonica             TAAACAGGCGGTGGCATATCGACAAATGTCATTATTGTTACGCCGACCAC
Cycas taitungensis                TAAACAGGCGGTGGCATATCGACAAATGTCATTATTGCTACGCCGACCAC
Anomodon rugelii                  AAAGCAATCAGTGGCATATCGACAAATGTCATTATTATTACGTCGACCAC
Anomodon attenuatus               AAAGCAATCAGTGGCATATCGACAAATGTCATTATTATTACGTCGACCAC
Megaceros aenigmaticus            TAAGCAAGCAGTGGCATATCGGCAGATGTCATTATTATTACGTCGACCAC
Phaeoceros laevis                 TAAGCAAGTAGTGGTATATCGGCAGATGTCATTATTATTACGTCGACCAC
                                   ** **    **** ****** ** ***********  **** *******

Lotus japonicus                   CAGGCCGTGAGGCTTTCCCAGGCGATGTTTTCTATTTACATTCCCGTCTC
BPM24_atp1                        CAGGCCGTGAGGCTTTCCCAGGCGATGTTTTCTATTTACATTCCCGTCTC
Millettia pinnata                 CAGGCCGTGAGGCTTTCCCAGGCGATGTTTTCTATTTACATTCCCGTCTC
RRIM600_atp1                      CAGGCCGTGAGGCTTTCCCAGGCGATGTTTTCTATTTACATTCCCGTCTC
Arabidopsis thaliana              CAGGCCGTGAGGCTTTCCCAGGTGATGTTTTCTATTTACATTCCCGTCTC
Brassica napus                    CAGGCCGTGAGGCTTTCCCAGGTGATGTTTTCTATTTACATTCCCGTCTC
RRII105_atp1                      CAGGCCGTGAGGCTTTCCCAGGCGATGTTTTCTATTTACATTCCCGTCTC
Beta macrocarpa                   CAGGCCGTGAGGCTTTCCCAGGCGACGTTTTCTATTTACATTCCCGTCTC
Beta vulgaris vulgaris            CAGGCCGTGAGGCTTTCCCAGGCGACGTTTTCTATTTACATTCCCGTCTC
Beta vulgaris maritima            CAGGCCGTGAGGCTTTCCCAGGCGACGTTTTCTATTTACATTCCCGTCTC
Silene latifolia                  CAGGCCGTGAGGCTTTCCCAGGCGACGTTTTCTATTTACATTCTCGTCTC
PB235_atp1                        CAGGCCGTGAGGCTTTCCCAGGCGATGTTTTCTATTTACATTCCCGTCTC
RRIT251_atp1                      CAGGCCGTGAGGCTTTCCCAGGCGATGTTTTCTATTTACATTCCCGTCTC
Citrullus lanatus                 CAGGCCGTGAGGCTTTCCCAGGCGATGTTTTCTATTTACATTCCCGTCTC
Cucurbita pepo                    CAGGCCGTGAGGCTTTCCCAGGCGATGTTTTCTATTTACATTCCCGTCTC
RRIC110_atp1                      CAGGCCGTGAGGCTTTCCCAGGCGATGTTTTCTATTTACATTCCCGTCTC
Oryza sativa Japonica             CAGGCCGTGAGGCTTTCCCAGGGGATGTTTTCTATTTACATTCCCGTCTC
Cycas taitungensis                CAGGCCGTGAGGCTTTCCCAGGGGATGTTCTCTATTTACATTCCCGTCCA
Anomodon rugelii                  CAGGTCGTGAGGCGTTCCCTGGAGATGTTTTTTATTTACATTCTCGTCTA
Anomodon attenuatus               CAGGTCGTGAGGCGTTCCCTGGAGATGTTTTTTATTTACATTCTCGTCTA
Megaceros aenigmaticus            CTGGCCGTGAGGCGTTCCCAGGGGATGTCTTCTATCCACATTCTCGTTTG
Phaeoceros laevis                 CTGGCTGTGAGGCGTTCCCAGGGGATGTGTTCTATTTACATTCCCGTTTG
                                  * **  ******* ***** ** ** **  * ***  ****** ***

Lotus japonicus                   TTAGAAAGAGCCGCTAAACGATCGGACCAGACAGGTGCAGGTAGCTTGAC
BPM24_atp1                        TTAGAAAGAGCCGCTAAACGATCGGACCAGACAGGTGCAGGTAGCTTGAC
Millettia pinnata                 TTAGAAAGAGCCGCTAAACGATCGGACCAGACAGGTGCAGGTAGCTTGAC
RRIM600_atp1                      TTAGAAAGAGCCGCTAAACGATCGGACCAGACAGGTGCAGGTAGCTTGAC
Arabidopsis thaliana              TTAGAAAGAGCGGCTAAACGATCGGACCAGACAGGTGCAGGTAGCTTGAC
Brassica napus                    TTAGAAAGAGCGGCTAAACGATCGGACCAGACAGGTGCAGGTAGCTTGAC
RRII105_atp1                      TTAGAAAGAGCCGCTAAACGATCGGACCAGACAGGTGCAGGTAGCTTGAC
Beta macrocarpa                   TTAGAAAGAGCCGCTAAACGATCGGACCAGACAGGCGCAGGTAGCTTGAC
Beta vulgaris vulgaris            TTAGAAAGAGCCGCTAAACGATCGGACCAGACAGGCGCAGGTAGCTTGAC
Beta vulgaris maritima            TTAGAAAGAGCCGCTAAACGATCGGACCAGACAGGCGCAGGTAGCTTGAC
Silene latifolia                  TTAGAAAGAGCCGCTAAACGATCGGACCAGACAGGTGCAGGTAGCTTGAC
PB235_atp1                        TTAGAAAGAGCCGCTAAACGATCGGACCAGACAGGTGCAGGTAGCTTGAC
RRIT251_atp1                      TTAGAAAGAGCCGCTAAACGATCGGACCAGACAGGTGCAGGTAGCTTGAC
Citrullus lanatus                 TTAGAAAGAGCCGCTAAACGATCGGACCAGACAGGTGCAGGTAGCTTGAC
Cucurbita pepo                    TTAGAAAGAGCCGCTAAACGATCGGACCAGACAGGTGCAGGTAGCTTGAC
```

```
RRIC110_atp1                       TTAGAAAGAGCCGCTAAACGATCGGACCAGACAGGTGCAGGTAGCTTGAC
Oryza sativa Japonica              TTAGAAAGAGCCGCTAAACGATCGGACCAGACAGGTGCAGGTAGCTTGAC
Cycas taitungensis                 TCAGAAAGAGCCGCTAAACGATCGGACCAGACAGGTGCGGGTAGCTTGAC
Anomodon rugelii                   TCAGAAAGAGCCGCTAAAATGTCAGACCAAACTGGTGCAGGTAGCTTGAC
Anomodon attenuatus                TCAGAAAGAGCCGCTAAAATGTCAGACCAAACTGGTGCAGGTAGCTTGAC
Megaceros aenigmaticus             TCAGAAAGAGCCGCTAAGATGTCAGACCAGACTGGCGCAGGTAGCTTGAC
Phaeoceros laevis                  TTAGAAAGAGCCGCTAAAATGTCAGACCAGACTGGTGCAGGTAGCTTGAC
                                   * ********* *****    ** ***** ** ** ** ***********

Lotus japonicus                    CGCCTTACCCGTCATTGAAACACAAGCGGGAGACGTATCGGCCTATATTC
BPM24_atp1                         CGCCTTACCCGTCATTGAAACACAAGCTGGAGACGTATCGGCCTATATTC
Millettia pinnata                  CGCCTTACCCGTCATTGAAACACAAGCGGGAGACGTATCGGCCTATATTC
RRIM600_atp1                       CGCCTTACCCGTCATTGAAACACAAGCTGGAGACGTATCGGCCTATATTC
Arabidopsis thaliana               CGCCTTACCCGTCATTGAAACACAAGCTGGAGACGTATCGGCCTATATTC
Brassica napus                     CGCCTTACCCGTCATTGAAACACAAGCTGGAGACGTATCGGCCTATATTC
RRII105_atp1                       CGCCTTACCCGTCATTGAAACACAAGCTGGAGACGTATCGGCCTATATTC
Beta macrocarpa                    CGCCCTACCCGTCATTGAAACACAAGCTGGAGACGTATCGGCCTATATTC
Beta vulgaris vulgaris             CGCCCTACCCGTCATTGAAACACAAGCTGGAGACGTATCGGCCTATATTC
Beta vulgaris maritima             CGCCCTACCCGTCATTGAAACACAAGCTGGAGACGTATCGGCCTATATTC
Silene latifolia                   CGCCTTACCCGTCATTGAAACACAAGCTGGAGACGTATCAGCCTATATTC
PB235_atp1                         CGCCTTACCCGTCATTGAAACACAAGCTGGAGACGTATCGGCCTATATTC
RRIT251_atp1                       CGCCTTACCCGTCATTGAAACACAAGCTGGAGACGTATCGGCCTATATTC
Citrullus lanatus                  CGCCTTACCCGTCATTGAAACACAAGCTGGAGACGTATCGGCCTATATTC
Cucurbita pepo                     CGCCTTACCCGTCATTGAAACACAAGCTGGAGACGTATCGGCCTATATTC
RRIC110_atp1                       CGCCTTACCCGTCATTGAAACACAAGCTGGAGACGTATCGGCCTATATTC
Oryza sativa Japonica              TGCGTTACCCGTGATTGAAACACAAGCTGGAGACGTATCGGCCTATATCC
Cycas taitungensis                 CGCGTTACCCGTCATTGAAACACAAGCTGGAGACGTGTCGGCCTATATCC
Anomodon rugelii                   TGCATTACCTGTAATTGAAACACAAGCTGGAGATGTATCTGCTTATATTC
Anomodon attenuatus                TGCATTACCTGTAATTGAAACACAAGCTGGAGATGTATCTGCTTATATTC
Megaceros aenigmaticus             TGCATTACCTGTGATTGAAACACAAGCTGGAGACGTATCTGCTTATATTC
Phaeoceros laevis                  TGCATTACCTGTGATTGAAACACAAGCTGGAGACGTATCTGCTTATATTC
                                    **  **** ** ************** ***** ** ** ** ***** *

Lotus japonicus                    CAACCAATGTGATCTCCATTACTGATGGACAAATATGTTTGGAAACAGAG
BPM24_atp1                         CTACCAATGTGATCUCCATTACTGATGGACAAATCTGTTTGGAAACAGAG
Millettia pinnata                  CCACCAATGTGATCTCAATTACTGATGGACAAATCTGTTTGGAAACAGAG
RRIM600_atp1                       CTACCAATGTGATCUCCATTACTGATGGACAAATCTGTTTGGAAACAGAG
Arabidopsis thaliana               CCACCAATGTGATCTCCATTACTGATGGACAAATCTGTTTGGAAACAGAG
Brassica napus                     CCACCAATGTGATCTCCATTACTGATGGACAAATCTGTTTGGAAACAGAG
RRII105_atp1                       CTACCAATGTGATCUCCATTACTGATGGACAAATCTGTTTGGAAACAGAG
Beta macrocarpa                    CCACCAATGTGATCTCCATTACTGATGGACAAATCTGTTTGGAAACAGAG
Beta vulgaris vulgaris             CCACCAATGTGATCTCCATTACTGATGGACAAATCTGTTTGGAAACAGAG
Beta vulgaris maritima             CCACCAATGTGATCTCCATTACTGATGGACAAATCTGTTTGGAAACAGAG
Silene latifolia                   CCACCAATGTGATCTCCATTACTGATGGACAAATCTGTTTGGAAACAGAG
PB235_atp1                         CTACCAATGTGATCUCCATTACTGATGGACAAATCTGTTTGGAAACAGAG
RRIT251_atp1                       CTACCAATGTGATCUCCATTACTGATGGACAAATCTGTTTGGAAACAGAG
Citrullus lanatus                  CCACCAATGTGATCUCTATTACTGATGGACAAATCTGTTTGGAAACAGAG
Cucurbita pepo                     CCACCAATGTGATCUCTATTACTGATGGACAAATCTGTTTGGAAACAGAG
RRIC110_atp1                       CTACCAATGTGATCUCCATTACTGATGGACAAATCTGTTTGGAAACAGAG
Oryza sativa Japonica              CCACCAATGTGATCTCCATTACAGATGGACAAATCTGTTTGGAAACAGAG
Cycas taitungensis                 CTACCAATGTGATCCCCATTACAGATGGACAAATCCGTTCGGAAACAGAG
Anomodon rugelii                   CGACCAATGTTATTCCCATTACAGATGGACAAATCTTTTTGGAAACAGAA
Anomodon attenuatus                CGACCAATGTTATTCCCATTACAGATGGACAAATCTTTTTGGAAACAGAA
Megaceros aenigmaticus             CCACCAATGTGATTCCCATTACAGATGGACAAATCTTTTTGGAAACAGAA
Phaeoceros laevis                  CCACCAATGTGATTTCCATTACAGATGGACAAATCTTTTTGGAAACAGAA
                                   * ******** **  * ***** ***********   ** *********

Lotus japonicus                    CTCTTTTATCGCGGAATTAGACCTGCTATTAACGTCGGCTTATCTGTCAG
BPM24_atp1                         CTCTTTTATCGCGGAATTAGACCTGCTATTAACGTCGGCTTATCTGTCAG
Millettia pinnata                  CTCTTTTATCGCGGAATTAGACCTGCTATTAACGTCGGCTTATCTGTCAG
RRIM600_atp1                       CTCTTTTATCGCGGAATTAGACCTGCTATTAACGTCGGCTTATCTGTCAG
Arabidopsis thaliana               CTCTTTTATCGCGGAATTAGACCTGCTATTAACGTUGGCTTATCTGTCAG
```

```
Brassica napus                      CTCTTTTATCGCGGAATTAGACCTGCTATTAACGTCGGCTTATCTGTCAG
RRII105_atp1                        CTCTTTTATCGCGGAATTAGACCTGCTATTAACGTCGGCTTATCTGTCAG
Beta macrocarpa                     CTCTTTTATCGCGGAATTAGACCTGCTATTAACGTCGGCTTATCTGTCAG
Beta vulgaris vulgaris              CTCTTTTATCGCGGAATTAGACCTGCTATTAACGTCGGCTTATCTGTCAG
Beta vulgaris maritima              CTCTTTTATCGCGGAATTAGACCTGCTATTAACGTCGGCTTATCTGTCAG
Silene latifolia                    CTCTTTTATCGCGGAATTAGACCTGCTATTAACGTCGGCTTATCTGTCAG
PB235_atp1                          CTCTTTTATCGCGGAATTAGACCTGCTATTAACGTCGGCTTATCTGTCAG
RRIT251_atp1                        CTCTTTTATCGCGGAATTAGACCTGCTATTAACGTCGGCTTATCTGTCAG
Citrullus lanatus                   CTCTTTTATCGCGGAATTAGACCTGCTATTAACGTCGGCTTATCTGTCAG
Cucurbita pepo                      CTCTTTTATCGCGGAATTAGACCCGCTATTAACGTCGGCTTATCCGTCAG
RRIC110_atp1                        CTCTTTTATCGCGGAATTAGACCTGCTATTAACGTCGGCTTATCTGTCAG
Oryza sativa Japonica               CTCTTTTATCGCGGAATTAGACCTGCTATTAACGTTGGCTTATCCGTCAG
Cycas taitungensis                  CTCTTTTATCGCGGAATCAGACCTGCTATTAACGTCGGCTTATCCGTCAG
Anomodon rugelii                    CTTTTTTATCGTGGAATTCGACCTGCTATTAATGTAGGATTATCTGTAAG
Anomodon attenuatus                 CTTTTTTATCGTGGAATTCGACCTGCTATTAATGTAGGATTATCTGTAAG
Megaceros aenigmaticus              CTCTTTTATCGTGGAATTCGACCTGCTATTAACGCGGGATTATCTGTGAG
Phaeoceros laevis                   CTCTTTTATCGTGGAATTCGACTTGCTATTAACGTGGGATTATCTGTGAG
                                    ** ******** *****  ***  ******** *  ** ***** ** **

Lotus japonicus                     TCGCGTCGGGTCTGCCGCTCAGTTGAAAGCTATGAAACAAGTCTGCGGGA
BPM24_atp1                          TCGCGTCGGGTCTGCCGCTCAGTTGAAAGCTATGAAACAAGTCTGCGGTA
Millettia pinnata                   TCGCGTCGGGTCTGCCGCTCAGTTGAAAGCTATGAAACAAGTCTGCGGTA
RRIM600_atp1                        TCGCGTCGGGTCTGCCGCTCAGTTGAAAGCTATGAAACAAGTCTGCGGTA
Arabidopsis thaliana                TCGCGTCGGGTCTGCCGCTCAGTTGAAAGCTATGAAACAAGTATGCGGTA
Brassica napus                      TCGCGTCGGGTCTGCCGCTCAGTTGAAAGCTATGAAACAAGTATGCGGTA
RRII105_atp1                        TCGCGTCGGGTCTGCCGCTCAGTTGAAAGCTATGAAACAAGTCTGCGGTA
Beta macrocarpa                     TCGCGTCGGGTCTGCCGCTCAGTTGAAAGCTATGAAACAAGTCTGCGGTA
Beta vulgaris vulgaris              TCGCGTCGGGTCTGCCGCTCAGTTGAAAGCTATGAAACAAGTCTGCGGTA
Beta vulgaris maritima              TCGCGTCGGGTCTGCCGCTCAGTTGAAAGCTATGAAACAAGTCTGCGGTA
Silene latifolia                    TCGCGTCGGGTCTGCCGCTCAGTTGAAAGCTATGAAACAAGTCTGCGGGA
PB235_atp1                          TCGCGTCGGGTCTGCCGCTCAGTTGAAAGCTATGAAACAAGTCTGCGGTA
RRIT251_atp1                        TCGCGTCGGGTCTGCCGCTCAGTTGAAAGCTATGAAACAAGTCTGCGGTA
Citrullus lanatus                   TCGCGTCGGGTCTGCCGCTCAGTTGAAAGCTATGAAACAAGTCTGCGGTA
Cucurbita pepo                      TCGCGTCGGGTCTGCCGCTCAGTTGAAAGCTATGAAACAAGTCTGCGGTA
RRIC110_atp1                        TCGCGTCGGGTCTGCCGCTCAGTTGAAAGCTATGAAACAAGTCTGCGGTA
Oryza sativa Japonica               TCGCGTCGGGTCTGCCGCTCAGTTGAAAGCTATGAAACAAGTCTGCGGTA
Cycas taitungensis                  TCGCGTCGGGTCTGCCGCTCAGTTGAAAGCTATGAGACAAGTACGTGGTA
Anomodon rugelii                    TCGTGTGAGTTCTGCGGCTCAGTCAAAAGCTATGAAACAGGTATGCGGTA
Anomodon attenuatus                 TCGTGTGAGTTCTGCGGCTCAGTCAAAAGCTATGAAACAGGTATGCGGTA
Megaceros aenigmaticus              TCGCGTCGGTTCTGCGGCTCAGTTGAAAGCTATGAAACAAGTATGTGGTA
Phaeoceros laevis                   TTGTGTTGGTTTTGCGGCTCAATTGAAAGCTATGAAACAAGTATGTGGTA
                                    * * **  * * *** ***** *  ********** *** **  * ** *

Lotus japonicus                     GTTTAAAACTTGAATTGGCACAATATCGCGAAGTCGCCGCCTTTGCTCAA
BPM24_atp1                          GTTUAAAACTGGAATTGGCACAATATCGCGAAGTGGCCGCCUTTGCTCAA
Millettia pinnata                   GTTTAAAACTGGAATTGGCACAATATCGCGAAGTCGCCGCCTTTGCTCAA
RRIM600_atp1                        GTTUAAAACTGGAATTGGCACAATATCGCGAAGTGGCCGCCUTTGCTCAA
Arabidopsis thaliana                GTTUAAAACTGGAATTGGCACAATATCGCGAAGTGGCCGCCTTTGCTCAA
Brassica napus                      GTTUAAAACTGGAATTGGCACAATATCGCGAAGTGGCCGCCTTTGCTCAA
RRII105_atp1                        GTTUAAAACTGGAATTGGCACAATATCGCGAAGTGGCCGCCUTTGCTCAA
Beta macrocarpa                     GTCUAAAACTGGAATTGGCACAATATCGCGAAGTGGCCGCCTTTGCTCAA
Beta vulgaris vulgaris              GTCUAAAACTGGAATTGGCACAATATCGCGAAGTGGCCGCCTTTGCTCAA
Beta vulgaris maritima              GTCUAAAACTGGAATTGGCACAATATCGCGAAGTGGCCGCCTTTGCTCAA
Silene latifolia                    GTCUAAAACTTGAATTGGCACAATATCGCGAAGTGGCCGCCTTTGCTCAA
PB235_atp1                          GTTUAAAACTGGAATTGGCACAATATCGCGAAGTGGCCGCCUTTGCTCAA
RRIT251_atp1                        GTTUAAAACTGGAATTGGCACAATATCGCGAAGTGGCCGCCUTTGCTCAA
Citrullus lanatus                   GTTUAAAACTGGAATTGGCACAATATCGCGAAGTGGCCGCCTTTGCTCAA
Cucurbita pepo                      GTTUAAAACTGGAATTGGCACAATATCGCGAAGTGGCCGCCTTTGCTCAA
RRIC110_atp1                        GTTUAAAACTGGAATTGGCACAATATCGCGAAGTGGCCGCCUTTGCTCAA
Oryza sativa Japonica               GTTUAAAACTGGAATTGGCACAATATCGCGAAGTGGCCGCCTTCGCTCAA
Cycas taitungensis                  GTTCAAAACCAGAATTGGCACAATATCGTGAAGTGGCCGCCTCTGCTCAA
Anomodon rugelii                    GCTTGAAACTAGAATTAGCGCAATATCGTGAAGTAGCCGCTTTTGCTCAA
```

```
Anomodon attenuatus               GCTTGAAACTAGAATTAGCGCAATATCGTGAAGTAGCCGCTTTTGCTCAA
Megaceros aenigmaticus            GCTTAAAACTAGAATTGGCACAGTATCGTGAGGTAGCCGCTTCTGCTCAA
Phaeoceros laevis                 GCTTAAAACTAGAATTGGCACAGTATCGTGAGGTAGCTGCTTCTGCTCAA
                                  *    ****  ***** ** ** ***** ** ** ** **    ******

Lotus japonicus                   TTTGGCTCAGACCTTGATGCTGCGACTCAGGCATTACTCAATAGAGGGGC
BPM24_atp1                        TTTGGCTCAGACCTTGATGCTGCGACTCAGGCATTACTCAATAGAGGCGC
Millettia pinnata                 TTTGGCTCAGACCTTGATGCTGCGACTCAGGCATTACTCAATAGAGGGGC
RRIM600_atp1                      TTTGGCTCAGACCTTGATGCTGCGACTCAGGCATTACTCAATAGAGGCGC
Arabidopsis thaliana              TTTGGCTCAGACCTTGATGCTGCGACTCAGGCATTACTCAATAGAGGTGC
Brassica napus                    TTTGGCTCAGACCTTGATGCTGCGACTCAGGCATTACTCAATAGAGGTGC
RRII105_atp1                      TTTGGCTCAGACCTTGATGCTGCGACTCAGGCATTACTCAATAGAGGCGC
Beta macrocarpa                   TTTGGGTCAGACCTTGATGCTGCGACTCAGGCATTACTCAATAGAGGTGC
Beta vulgaris vulgaris            TTTGGGTCAGACCTTGATGCTGCGACTCAGGCATTACTCAATAGAGGTGC
Beta vulgaris maritima            TTTGGGTCAGACCTTGATGCTGCGACTCAGGCATTACTCAATAGAGGTGC
Silene latifolia                  TTTGGCTCAGACCTTGATGCTGCGACTCAGGCATTACTCAATAGAGGTGC
PB235_atp1                        TTTGGCTCAGACCTTGATGCTGCGACTCAGGCATTACTCAATAGAGGCGC
RRIT251_atp1                      TTTGGCTCAGACCTTGATGCTGCGACTCAGGCATTACTCAATAGAGGCGC
Citrullus lanatus                 TTTGGGTCAGACCTTGATGCTGCGACTCAGGCATTACTCAATAGAGGTGC
Cucurbita pepo                    TTTGGGTCAGACCTTGATGCAGCGACTCAGGCATTACTCAATAGAGGTGC
RRIC110_atp1                      TTTGGCTCAGACCTTGATGCTGCGACTCAGGCATTACTCAATAGAGGCGC
Oryza sativa Japonica             TTTGGGTCAGACCTTGATGCTGCGACTCAGGCATTACTCAATAGAGGTGC
Cycas taitungensis                TCCGGGTCAGACCTTGATGCTGCTACTCAGGCACCATCAAATAGAGGTGC
Anomodon rugelii                  TCTGGTTCAGACCTTGATGCGGCTACTCAGTATCTATTAAATCGTGGGGC
Anomodon attenuatus               TCTGGTTCAGACCTTGATGCGGCTACTCAGTATCTATTAAATCGTGGGGC
Megaceros aenigmaticus            TTCGGTTCAGACCCTGATGCTGCTACTAAGTATTTATCAAATCGTGGAGC
Phaeoceros laevis                 TTTGGTTCAGACCTTGATGCTGCTATTAAGTATTTATTAAATCGTGGAGC
                                  *  ** ******* ****** ** * * **     *   *** * ** **

Lotus japonicus                   AAGGCTGACAGAAGTACTGAAACAACCACAATATGCACCACTTCCAATTG
BPM24_atp1                        AAGGCTTACAGAAGTACUGAAACAACCACAATATGCACCACTTCCAATTG
Millettia pinnata                 AAGGCTGACAGAAGTACTGAAACAACCACAATATGCACCACTTCCAATTG
RRIM600_atp1                      AAGGCTTACAGAAGTACNGAAACAACCACAATATGCACCACTTCCAATTG
Arabidopsis thaliana              AAGGCTGACAGAAGTACUGAAACAACCACAATATGCACCACTTCCAATTG
Brassica napus                    AAGGCTGACAGAAGTACUGAAACAACCACAATATGCACCACTTCCAATTG
RRII105_atp1                      AAGGCTTACAGAAGTACUGAAACAACCACAATATGCACCACTTCCAATTG
Beta macrocarpa                   AAGGCTTACAGAAGTACUGAAACAACCACAATATGCACCACTTCCAATTG
Beta vulgaris vulgaris            AAGGCTTACAGAAGTACUGAAACAACCACAATATGCACCACTTCCAATTG
Beta vulgaris maritima            AAGGCTTACAGAAGTACUGAAACAACCACAATATGCACCACTTCCAATTG
Silene latifolia                  AAGGCTTACAGAAGTACUGAAACAACAACAATATGCACCACTTCCAATTG
PB235_atp1                        AAGGCTTACAGAAGTACUGAAACAACCACAATATGCACCACTTCCAATTG
RRIT251_atp1                      AAGGCTTACAGAAGTACUGAAACAACCACAATATGCACCACTTCCAATTG
Citrullus lanatus                 AAGGCTTACAGAAGTACUGAAACAACCACAATATGCACCACTTCCAATTG
Cucurbita pepo                    AAGGCTTACAGAAGTACUGAAACAACCACAATATGCACCACTTCCAATTG
RRIC110_atp1                      AAGGCTTACAGAAGTACUGAAACAACCACAATATGCACCACTTCCAATTG
Oryza sativa Japonica             AAGGCTTACAGAAGTGUCCAAACAACCACAATATGAACCACTTCCAATTG
Cycas taitungensis                TAGGCTTACTGAAGTTCCGAAACAACCACAATATCCACCACTTCCTATTG
Anomodon rugelii                  GAGGCTAACAGAGGTTCTTAAACAACCACAATATAGCCCAATTCCTATTG
Anomodon attenuatus               GAGGCTAACAGAGGTTCTTAAACAACCACAATATAGCCCAATTCCTATTG
Megaceros aenigmaticus            TAGGCTAACGGAAGTTCCTAAACAACCACAGTATAGCCCAATTCCAATTG
Phaeoceros laevis                 TAGGCTCACGGAAGTTTCTAAACAACTACAGTATAGCCTAATTCCAATTG
                                   ***** ** ** **    ******* *** ***   * * **** ****

Lotus japonicus                   AAAAACAAATTCTAGTCATTTATGCAGCTGTCAATGGATTCTGTGATCGA
BPM24_atp1                        AAAAACAAATTCTAGTCATTTATGCAGCTGTCAATGGATTCTGTGATCGA
Millettia pinnata                 AAAAACAAATTCTAGTCATTTATGCAGCTGTCAATGGATTCTGTGATCGA
RRIM600_atp1                      AAAAACAAATTCTAGTCATTTATGCAGCTGTCAATGGATTCTGTGATCGA
Arabidopsis thaliana              AAAAACAAATACTAGTCATTTATGCAGCTGTCAATGGATTCTGTGATCGA
Brassica napus                    AAAAACAAATACTAGTCATTTATGCAGCTGTCAATGGATTCTGTGATCGA
RRII105_atp1                      AAAAACAAATTCTAGTCATTTATGCAGCTGTCAATGGATTCTGTGATCGA
Beta macrocarpa                   AAAAACAAATTCTAGTCATTTACGCAGCTGTCAATGGATTCTGTGATCGA
Beta vulgaris vulgaris            AAAAACAAATTCTAGTCATTTACGCAGCTGTCAATGGATTCTGTGATCGA
```

```
Beta vulgaris maritima            AAAAACAAATTCTAGTCATTTACGCAGCTGTCAATGGATTCTGTGATCGA
Silene latifolia                  AAAAACAAATTATAGTCATTTACGCAGCTGTCAATGGATTCTGTGATCGA
PB235_atp1                        AAAAACAAATTCTAGTCATTTATGCAGCTGTCAATGGATTCTGTGATCGA
RRIT251_atp1                      AAAAACAAATTCTAGTCATTTATGCAGCTGTCAATGGATTCTGTGATCGA
Citrullus lanatus                 AAAAACAAATTCTAGTCATTTATGCTGCTGTCAATGGATTCTGTGATCGA
Cucurbita pepo                    AAAAACAAATTCTAGTCATTTATGCTGCTGTCAATGGATTCTGTGATCGA
RRIC110_atp1                      AAAAACAAATTCTAGTCATTTATGCAGCTGTCAATGGATTCTGTGATCGA
Oryza sativa Japonica             AAAAACAAATTGTTGTGATTTATGCTGCTGTCAACGGCTTCTGTGATCGA
Cycas taitungensis                AAAAACAAATTCCAGTTATTCACGCAGCTGTGAAAGGGTTTCGTGATCGA
Anomodon rugelii                  AAAAACAAATAGTTGTTATTTATGCAGCTGTCAAAGGTTATTTAGATCAA
Anomodon attenuatus               AAAAACAAATAGTTGTTATTTATGCAGCTGTCAAAGGTTATTTAGATCAA
Megaceros aenigmaticus            AGAAACAAATTGTGGTTATTCATGCAGCTGTCAAAGGTTATTTAGATGAA
Phaeoceros laevis                 AGAAACAAATTGTGGTTATTCATGCAGCTGTCAAAGGTTATTTAGATGAA
                                  * ********    ** *** * ** ***** ** ** *     ***  *

Lotus japonicus                   ATGCCATTAGACAAAATTGCTCAATATGAGAGAGACATTCTAAGTACTAT
BPM24_atp1                        ATGCCACTAGACAGAATTTCTCAATATGAGAGAGCCATTCUAAGTAGTAT
Millettia pinnata                 ATGCCATTAGAAAAAAATTCCTCAATATGAAAGAGACATTCTAACTACTAT
RRIM600_atp1                      ATGCCACTAGACAGAATTTCTCAATATGAGAGAGCCATTCUAAGTAGTAT
Arabidopsis thaliana              ATGCCACTAGACAGAATCTCTCAATATGAGAAAGCCATTCUAAATAGTGT
Brassica napus                    ATGCCACTAGACAGAATCTCTCAATATGAGAAAGCCATTCUAAATAGTGT
RRII105_atp1                      ATGCCACTAGACAGAATTTCTCAATATGAGAGAGCCATTCUAAGTAGTAT
Beta macrocarpa                   ATGCCACTAGATAAAATTTCTCAATATGAGCGAACCATTCUAAATAGTGT
Beta vulgaris vulgaris            ATGCCACTAGATAAAATTTCTCAATATGAGCGAACCATTCUAAATAGTGT
Beta vulgaris maritima            ATGCCACTAGATAAAATTTCTCAATATGAGCGAACCATTCUAAATAGTGT
Silene latifolia                  ATGCCACTAGATAAAATTTCTCAATATGAGAGAACCATTCUAAATAGTGT
PB235_atp1                        ATGCCACTAGACAGAATTTCTCAATATGAGAGAGCCATTCUAAGTAGTAT
RRIT251_atp1                      ATGCCACTAGACAGAATTTCTCAATATGAGAGAGCCATTCUAAGTAGTAT
Citrullus lanatus                 ATGCCACTAGACAGAATTTCTCAATATGAGAGAGCCATTCUAAGTCGTAT
Cucurbita pepo                    ATGCCACTAGACAGAATTTCTCAATATGAGAGAGCCATTCCAAGTCGTAT
RRIC110_atp1                      ATGCCACTAGACAGAATTTCTCAATATGAGAGAGCCATTCUAAGTAGTAT
Oryza sativa Japonica             ATGCCACTAGACAGAATTTCTCAATATGAAAAAGCCATTCTAAGTACTAT
Cycas taitungensis                ATGCCACTAGACAGAATTCCTCGGTATGAGAGAGCCATTTCAAGTAGTAT
Anomodon rugelii                  ATTCCTATTTCGAGCATCAATCAATATGAACATGAGCTTTTGAAGTCTAT
Anomodon attenuatus               ATTCCTATTTCGAGCATCAATCAATATGAACATGAGCTTTTGAAGTCTAT
Megaceros aenigmaticus            ATTCCTATTTCAATTATTAATGAATATGAACATGAGCCATTGAAGTCTAT
Phaeoceros laevis                 ATTCCTATTTCAATTATTAATGAATATGAACATGAGCCATTGAAGCCTAT
                                  ** **  *    *  **   *   *****             *    * *

Lotus japonicus                   AAAACCAGAATTACTACAATCACT------AAAAGGTATACTAACTAGCG
BPM24_atp1                        AAAACCAGAATTACTACAATCCCTTTTAGAAAAAGGTGGGTTAACTAACG
Millettia pinnata                 TAAACCAGAATTACTACAATCACT------AAAAGGTGTACTAAATAGCG
RRIM600_atp1                      AAAACCAGAATTACTACAATCCCTTTTAGAAAAAGGTGGGTTAACTAACG
Arabidopsis thaliana              CAAACCTGAATTACTACAAGCCCTT------AAAGGTGGATTAACTAACG
Brassica napus                    CAAACCTGAATTACTACAAGCCCTT------AAAGGTGGATTAACTAACG
RRII105_atp1                      AAAACCAGAATTACTACAATCCCTTTTAGAAAAAGGTGGGTTAACTAACG
Beta macrocarpa                   AAAACCAGAATTATTACAATCCCT------TAAGGGGGGGTTAACTAACG
Beta vulgaris vulgaris            AAAACCAGAATTATTACAATCCCT------TAAGGGGGGGTTAACTAACG
Beta vulgaris maritima            AAAACCAGAATTATTACAATCCCT------TAAGGGGGGGTTAACTAACG
Silene latifolia                  AAACCCAGAATTCTTACAATCCCT------AAAGGGCGGCTTAACTAACG
PB235_atp1                        AAAACCAGAATTACTACAATCCCTTTTAGAAAAAGGTGGGTTAACTAACG
RRIT251_atp1                      AAAACCAGAATTACTACAATCCCTTTTAGAAAAAGGTGGGTTAACTAACG
Citrullus lanatus                 AAAACCAGAATTACTACAATCCCTTTTAGAAAAAGGTGGGTTAACTAACG
Cucurbita pepo                    AAAACCAGAATTACTACAATCCCTTTTCTCAAAAGGTGGGTTAACTAACG
RRIC110_atp1                      AAAACCAGAATTACTACAATCCCTTTTAGAAAAAGGTGGGTTAACTAACG
Oryza sativa Japonica             TAATCCAGAATTACTAAAATCCTTCAACGAAAAAGGGGGATTAACTAACG
Cycas taitungensis                AGATCCCGAATTACTACAATCCGTTCCGGAAAAAGGTGAGTTAACTAACG
Anomodon rugelii                  AGACTCAGATATACTTTCTGCTATCGTACAACAAAAAAACATTACTGAGC
Anomodon attenuatus               AGACTCAGATATACTTTCTGCTATCGTACAACAAAAAAACATTACTGAGC
Megaceros aenigmaticus            TGATCCAAGTATACTTTCTGCTATTGTTAAACAAAAAAGCATCTCTGAGG
Phaeoceros laevis                 TGATCCAAGTATACTTTTTGTTATTGTGAAACAAAAAAGCATCTCTGAGG
                                    *  *     *  *        *        *        *   *
```

```
Lotus japonicus                   AAAGAAAGATAGAACUAGATGCATTCTTAAAAGAAAAAGCAAATTC--TT
BPM24_atp1                        AAAGAAAGATGGAACUAGATGCATTCTTAAAAGAAAGCGCTTTGCC--TT
Millettia pinnata                 AAAGAAAGATAGAACUAGATGCATTCTTAAAAGAAAAAGCAAAAAC--TT
RRIM600_atp1                      AAAGAAAGATGGAACNAGATGCATTCTTAAAAGAAAGCGCTTTGCC--TT
Arabidopsis thaliana              AAAGAAAAATGGAACUAGATGCTTTCTTAAAAGAAAGAGCTTTAGC--TT
Brassica napus                    AAAGAAAAATGGAACUAGATGCTTTCTTAAAAGAAAGAGCTTTAGC--TT
RRII105_atp1                      AAAGAAAGATGGAACUAGATGCATTCTTAAAAGAAAGCGCTTTGCC--TT
Beta macrocarpa                   AAAAAAAGATGGAACTAGATTCCTTCTTAAAAGAATGCGCTTTGAA--TT
Beta vulgaris vulgaris            AAAAAAAGATGGAACTAGATTCCTTCTTAAAAGAATGCGCTTTGAA--TT
Beta vulgaris maritima            AAAAAAAGATGGAACTAGATTCCTTCTTAAAAGAATGCGCTTTGAA--TT
Silene latifolia                  AAAAAAAGATGGAACTAGATTCATTCTTAAAAGAATGCGCTTTGAA--TT
PB235_atp1                        AAAGAAAGATGGAACUAGATGCATTCTTAAAAGAAAGCGCTTTGCC--TT
RRIT251_atp1                      AAAGAAAGATGGAACUAGATGCATTCTTAAAAGAAAGCGCTTTGCC--TT
Citrullus lanatus                 AAAGAAAGATGGAACUAGATGCATTCTTAAAAGAAAGCGCTTTGCC--TT
Cucurbita pepo                    AAAGAAAGATGGAACCAGATGCATTCTTAAAAGAAAGCGCTTTGCC--TT
RRIC110_atp1                      AAAGAAAGATGGAACCAGATGCATTCTTAAAAGAAAGCGCTTTGCC--TT
Oryza sativa Japonica             AAAGAAAGATTGAACUTGATGCTTUTTTAAAACAAACTGCGAAGGAGATT
Cycas taitungensis                AAATAAGGATGAAACCAGATGCTCCTTCAAAAGAAAGCGTTAACCT--TT
Anomodon rugelii                  AGATTGATAGTCAACTGGCTACCTTTTGTCAAAAATTTACACAGAG--CT
Anomodon attenuatus               AGATTGATAGTCAACTGGCTACCTTTTGTCAAAAATTTACACAGAG--CT
Megaceros aenigmaticus            AAATTAACAGTCAACTTGCGACCTTTCGCAAAAAATTGACACAGAG--CT
Phaeoceros laevis                 AAATTAACAGTCAACTTGCGACCTTTTGCAAAAAATTTACACAGAG--CT
                                  * *     *   ***  *   *        ** **              *

Lotus japonicus                   A-----------AATCTGATGAAAAGGGTTCCT-----CCAGCTGTGGA-
BPM24_atp1                        T-----------CCTATGA-------------------------------
Millettia pinnata                 ACTT--------AATATGATGATGAAAAAAATG-----AAAAACATAGGT
RRIM600_atp1                      TCCT--------ATGA----------------------------------
Arabidopsis thaliana              T-----------AATTTAGCTTTCATCACATTA-----GGAATCCTCAAT
Brassica napus                    T-----------AATTTAGCTTTCATCACATTA-----GGAATCCTCAAT
RRII105_atp1                      T-----------CCTATGA-------------------------------
Beta macrocarpa                   ACTAATTCTTCTAACTTTCTATTTAT--TGAGA----TTAGATGTAATTA
Beta vulgaris vulgaris            ACTAATTCTTCTAACTTTCTATTTATATTGAGA----TTAGATGTAATTA
Beta vulgaris maritima            ACTAATTCTTCTAACTTTAGATTTAT--TGAGA----TTAGATGTAATTA
Silene latifolia                  ACCTAAAAAACAAAAGTGAAGTCTAAAATTTCC----TAAAAAGATATAA
PB235_atp1                        TCCTATGA------------------------------------------
RRIT251_atp1                      TCCTATGA------------------------------------------
Citrullus lanatus                 ACCTATGATGCAAAATCAAACTGGATGATGCAAGC---TGGAGGGGGTCC
Cucurbita pepo                    ACCTATGATGCAAAATCAAACTGGATGATGCAAGCAGCTGGAGGGGGTCC
RRIC110_atp1                      TCCTATGA------------------------------------------
Oryza sativa Japonica             AATT--------AGATAGACCTTTTTATTTTTCGTCATTCGATCACGAAA
Cycas taitungensis                GCTGAGCGTGGCGAAGGTGAATCGATTCCTCCAAATAAAAAGATACACAA
Anomodon rugelii                  TCTTAGC-----AACTCATTCAGTTUAA----------------------
Anomodon attenuatus               TCTTAGC-----AACTCATTCAGTTUAA----------------------
Megaceros aenigmaticus            TCTTAGC-----AACTCATTCGGTTTGA----------------------
Phaeoceros laevis                 TCTTAGC-----AACTCATTCGGTTTAA----------------------

Lotus japonicus                   --AGCTCTTCGAGCTCCCCGACGTCAGAAGCCAGCGTGAACCA-ACA---
BPM24_atp1                        --------------------------------------------------
Millettia pinnata                 CTGAGGATCCTGCCTCTTTTTATTTTGTATCTTGCTTATTTCTTATT---
RRIM600_atp1                      --------------------------------------------------
Arabidopsis thaliana              CAGTAAGAAAGAAAAGTTGTTAAGCAAAGACTGGAGAGGCCCCCGGTCGA
Brassica napus                    CAGTAAGAAAGAAAAGTTCTTAAGCAAAGACTGGAGAGGCCCCCGGTCGA
RRII105_atp1                      --------------------------------------------------
Beta macrocarpa                   AGAATA--AAGGCTA-ATATCTTGCCGAAAGGCGGG---AG--GGTGCCG
Beta vulgaris vulgaris            AGAATA--AAGGCTA-ATATCTTGCCGAAAGGCGGG---AG--GGTGCCG
Beta vulgaris maritima            AGAATA--AAGGCTA-ATATCTTGCCGAAAGGCGGG---AG--GGTGCCG
Silene latifolia                  AAAGTGTGAAAGAAA-GCACGAAAAAGAAATCCAATTTTGG--TAAGACT
PB235_atp1                        --------------------------------------------------
RRIT251_atp1                      --------------------------------------------------
```

```
Citrullus lanatus                 CCAGTGAATGTGAAATGTAGGTTTCAGAAAAAGGAACCGAGGTTATGAGA
Cucurbita pepo                    CCAGTTCATGTGAAATGTAGGTTTCAGAAAAAGGAACCGAGGTTATGAGA
RRIC110_atp1                      --------------------------------------------------
Oryza sativa Japonica             ACAGGGATTCTGGAA---CGGCCAAGAATCCCAGCGGTTGTTCGGGTCGA
Cycas taitungensis                TCGACGATCCTTGATTGGTTTGAATAGGGGCTCTGGTTTCTTGGGAGCGG
Anomodon rugelii                  --------------------------------------------------
Anomodon attenuatus               --------------------------------------------------
Megaceros aenigmaticus            --------------------------------------------------
Phaeoceros laevis                 --------------------------------------------------


Lotus japonicus                   A--ACAAGCCCCAAACCCCGAGCCCCCAGAGCCTGTTGCCCCT--TACCT
BPM24_atp1                        --------------------------------------------------
Millettia pinnata                 GTTATATAAACTAAAGCATTGTTTCTTGGAAACAATTGCTCTTGTTGGCT
RRIM600_atp1                      --------------------------------------------------
Arabidopsis thaliana              GATGTAGTAAGTAGGTCTCCAATACTGGGAGACTGAGGAGAATGGGAGTT
Brassica napus                    GATGTAGTAAGTAGGTCTCCAATACTGGGAGACTGAGGAGAATGGGAGTT
RRII105_atp1                      --------------------------------------------------
Beta macrocarpa                   AGACCCGGGGCAACGGGGTCTTCCCATCATG---ATAGACTAAAGGGAAA
Beta vulgaris vulgaris            AGACCCGGGGCAACGGGGTCTTCCCATCATG---ATAGACTAAAGGGAAA
Beta vulgaris maritima            AGACCCGGGGCAACGGGGTCTTCCCATCATG---ATAGACTAAAGGGAAA
Silene latifolia                  GGACTCTGAACTACGAAAAAGAGCAACTAGGTTCATCGTTCTTAAAAAGA
PB235_atp1                        --------------------------------------------------
RRIT251_atp1                      --------------------------------------------------
Citrullus lanatus                 GTAAGTGGAGTTGGGCGGGCGACTCTCCAGGG-CCTCATTCATATCTCGA
Cucurbita pepo                    GTAAGTGGAGTTGGGCGGGCTACTCTCCAGGG-CCTCATTCATATCTCGA
RRIC110_atp1                      --------------------------------------------------
Oryza sativa Japonica             AAAACCGAGAACAAGAC----ATGCCACAAAGTGGCAGATGAAGGCAGGG
Cycas taitungensis                AGATGATTAATCCCGCCAC---GCCAGAGCGGCAAGTAGGATCGAGGGTT
Anomodon rugelii                  --------------------------------------------------
Anomodon attenuatus               --------------------------------------------------
Megaceros aenigmaticus            --------------------------------------------------
Phaeoceros laevis                 --------------------------------------------------


Lotus japonicus                   TTCCCATCCTTTACTGGATGACAAC-ACTCGTAGCCAAGAACTAAATGAG
BPM24_atp1                        --------------------------------------------------
Millettia pinnata                 TGACACCCCCCTTATTTATGATGGA-GGCCTTTCCTCCCAGCTCCAGCTC
RRIM600_atp1                      --------------------------------------------------
Arabidopsis thaliana              TGTGGGTTGAGGGTGGCATGCTCCCCAGGGCCCTCTTTTTTTTAGATAGG
Brassica napus                    TGTGGGTTGAGGGTGGCATGCTCCCCAGGGCCCTCTTTTTTTTAGATAGG
RRII105_atp1                      --------------------------------------------------
Beta macrocarpa                   GTCGCTCGGAGATATTGGTTCGAATCCAATTCATCATTCCAGTTCATAAG
Beta vulgaris vulgaris            GTCGCTCGGAGATATTGGTTCGAATCCAATTCATCATTCCAGTTCATAAG
Beta vulgaris maritima            GTCGCTCGGAGATATTGGTTCGAATCCAATTCATCATTCCAGTTCATAAG
Silene latifolia                  TTTTTTCTTACTTTCTTGTCGTTATTATGATTCCCTTGTCCTCTGGTAGA
PB235_atp1                        --------------------------------------------------
RRIT251_atp1                      --------------------------------------------------
Citrullus lanatus                 GGAGTGCGTTAGTGTTTCGACTGAGGGACAAGTCAGTGTACTTTAGGAAG
Cucurbita pepo                    GGAGTGCGTTAGTCTTTCGGCTGAGGGACAAGTCAGTGTACTTTAGGAAG
RRIC110_atp1                      --------------------------------------------------
Oryza sativa Japonica             GGGAGAGCCTAGTCCTCAACCTCTTCTTCCCCAAAAGGTAGTTATGAACG
Cycas taitungensis                AGAGAACGCGACCACGTTTCTTTCGGACTGGCTGATCTTAACCTCGAGCC
Anomodon rugelii                  --------------------------------------------------
Anomodon attenuatus               --------------------------------------------------
Megaceros aenigmaticus            --------------------------------------------------
Phaeoceros laevis                 --------------------------------------------------


Lotus japonicus                   CGCGGGGGTCTTCATTGGCGGGGGATATCTGACTCTGACAAGGAAGATTT
BPM24_atp1                        --------------------------------------------------
Millettia pinnata                 TTCGAGCATACCTAACC-CAGAAGCCAGCGTGAACCAAGAAGCCCCAAAC
```

```
RRIM600_atp1                      --------------------------------------------------
Arabidopsis thaliana              TAGGGTAATGCATTTCAGTATGAATTTGATCTAGCAGTGTAAACCTGAGA
Brassica napus                    TAGGGTAATGCATTTCAGTATGAATTTGATCTAGCAGTGTAAACCTGAGA
RRII105_atp1                      --------------------------------------------------
Beta macrocarpa                   GCCTTCATTCAATAGAAGTTTAGGATGGAGCTATCACTAATAGAC--GGA
Beta vulgaris vulgaris            GCCTTCATTCAATAGAAGTTTAGGATGGAGCTATCACTAATAGAC--GGA
Beta vulgaris maritima            GCCTTCATTCAATAGAAGTTTAGGATGGAGCTATCACTAATAGAC--GGA
Silene latifolia                  GAGTCTCCTTCTTATATGCGAGGGCTTGGGCCCAGGATCGGAGCCCAGGA
PB235_atp1                        --------------------------------------------------
RRIT251_atp1                      --------------------------------------------------
Citrullus lanatus                 CTGTTGAGTACCCGATGAGATAGAATTGAAATGTGTCCATTC--------
Cucurbita pepo                    CTGGTGAGTACCCGATCAGATAGAATTGAAATGTCTA-------------
RRIC110_atp1                      --------------------------------------------------
Oryza sativa Japonica             TGCCAAACTTATTG--GATTTATTCTTGGAATGCTCATAACCACCTTTAC
Cycas taitungensis                TATGAAAAAACACTACTTTCCCGGCCCTATGAAATG--------------
Anomodon rugelii                  --------------------------------------------------
Anomodon attenuatus               --------------------------------------------------
Megaceros aenigmaticus            --------------------------------------------------
Phaeoceros laevis                 --------------------------------------------------


Lotus japonicus                   TTTTAACTT------
BPM24_atp1                        ---------------
Millettia pinnata                 CCCGAGCCC------
RRIM600_atp1                      ---------------
Arabidopsis thaliana              TTT------------
Brassica napus                    TTT------------
RRII105_atp1                      ---------------
Beta macrocarpa                   TGG------------
Beta vulgaris vulgaris            T--------------
Beta vulgaris maritima            TGG------------
Silene latifolia                  TTCCCGAGAAAGAAA
PB235_atp1                        ---------------
RRIT251_atp1                      ---------------
Citrullus lanatus                 ---------------
Cucurbita pepo                    ---------------
RRIC110_atp1                      ---------------
Oryza sativa Japonica             T--------------
Cycas taitungensis                ---------------
Anomodon rugelii                  ---------------
Anomodon attenuatus               ---------------
Megaceros aenigmaticus            ---------------
Phaeoceros laevis                 ---------------
```

###################

atp4 multiple sequence alignment

```
Citrullus lanatus                 ----TTCTCGCTGGGCGAGCGCATCCGATT----CGAAAGTCCTTTCCTA
Cucurbita pepo                    -----TCTCGCTGGGCGAGCGCATCCGATT----CTCAAGTCCTTTCCTA
Silene latifolia                  GCCTTTCTCGCTGGGCGAGCGCACCCGATT----CGAA----CTTTCCTA
Lotus japonicus                   ---------GCTGGGCGAGCGCATCCGATTGATTCTAAAGTCCTTTCCTA
Millettia pinnata                 -----------TGGGCGAGCGCATCCGATTGATTCTAAAGTCCTTTCCTA
PB235_atp4                        --------------------------------------------------
RRIC110_atp4                      --------------------------------------------------
RRII105_atp4                      --------------------------------------------------
RRIT251_atp4                      --------------------------------------------------
BPM24_atp4                        --------------------------------------------------
Spirodela polyrhiza               -------------------CGCATCCGATT----CTATAATCCTGTCCTA
RRIM600_atp4                      --------------------------------------------------
Zea mays mays                     -----TCTATTTGTTCGAATGT-TCTTTTTTTGGAAAAACCAACCACAAA
Cycas taitungensis                --------CCCTAAGCTTATCTTTACGACCATAATATAGAATAGATCTTC
Megaceros aenigmaticus            -----------------------------------------------CTG
```

```
Phaeoceros laevis                 --------------------------------------------GCCCTG

Citrullus lanatus                 AACCACTTCCCGTTCTGTTGCTGAAATCTCGAGAAAAGCTTGATAA----
Cucurbita pepo                    AACCACTTCCCGTTCAGTTGCTGAAAGATAGAGAGAAGCTTGATAA----
Silene latifolia                  AGAAACATCCCATTCAGTTGCTGAAAGATAAAGATAAGCTTTCTAA----
Lotus japonicus                   AACCACTTCCCGTTCAGTTGCTGAAAGATAGATAGAACCCTTCTAA----
Millettia pinnata                 AACCACTTCCCGTTCAGTTGCTGAAAGATAGATAGAACCTTTCTAACT--
PB235_atp4                        --------------------------------------------------
RRIC110_atp4                      --------------------------------------------------
RRII105_atp4                      --------------------------------------------------
RRIT251_atp4                      --------------------------------------------------
BPM24_atp4                        --------------------------------------------------
Spirodela polyrhiza               CACAACTTCCCGTTGAGTTGCTGAAAAATAGATACTGATAAAGTAAGTTT
RRIM600_atp4                      --------------------------------------------------
Zea mays mays                     AAAAGTCTCCCTTTCTCTTGGAGCAGAGCTTCATCATAAAAGTGGAGAGT
Cycas taitungensis                TACC-TCTTCCGTTCAGTTGCTAAAAAAGTTATGATAACGTTGATA----
Megaceros aenigmaticus            CGCAGACATGTTTTACGCGGTCAGAAGTTGTTCACTCTGATGAATA---T
Phaeoceros laevis                 CGCAGACATGTTTTACGCGGTCAGAAGTTA---ACTCTGATGAATA---T

Citrullus lanatus                 ----ATGAGATGGAGTTCCACGAATATGCACGCTAGAAAGAT-GCTATTT
Cucurbita pepo                    ----ATGAGATGGAGTTCCACGAATATGCGGGCTATAAAGAT-GCTATTT
Silene latifolia                  ----ATGAGAAAGAGTTTCACGAAGATGCAGGCTAGAAAGAT-GCTATTT
Lotus japonicus                   ----ATGAGATTGAGTTCCACGAATATGCAAGCTAGAAAGAT-GUTATTT
Millettia pinnata                 --AAATGAGATTGAGTTCCACGAATATGCAAGCTAGAAAGAT-GUTATTT
PB235_atp4                        ----ATGAGATTGAGTTCCACGAATATGCAGGCTAGAAAGAT-GTTATTT
RRIC110_atp4                      ----ATGAGATTGAGTTCCACGAATATGCAGGCTAGAAAGAT-GTTATTT
RRII105_atp4                      ----ATGAGATTGAGTTCCACGAATATGCAGGCTAGAAAGAT-GTTATTT
RRIT251_atp4                      ----ATGAGATTGAGTTCCACGAATATGCAGGCTAGAAAGAT-GTTATTT
BPM24_atp4                        ----ATGAGATTGAGTTCCACGAATATGCAGGCTAGAAAGAT-GTTATTT
Spirodela polyrhiza               CTAAATGAAATTTAGTTCAACGGATATAAAAGATAGAAAGAT-GCTATTT
RRIM600_atp4                      ----ATGAGATTGAGTTCCACGAATATGCAGGCTAGAAAGAT-GTTATTT
Zea mays mays                     CACAATGAGATTTAGTGGAATGGATATGAAGGGTATAAATAT-GUTATTT
Cycas taitungensis                ---AATGATATTTAGTTCCACGAATAGGAAGGAGAGAAATAT-GCTATTT
Megaceros aenigmaticus            CGGAAACGGACCAAGAAGAAAGAAAAATACGGATGCGTGAATTGATAATA
Phaeoceros laevis                 CGAAAACAGACCAAGAAGAAAGAAAAATACGGATGCGTGAATTGATAATA
                                      *    *   **    * * * *     *        ** * ** *

Citrullus lanatus                 GCTGCTATTCTATUTATTTGTGCATTAAGTTCGAAGAAGATCTUAATCTA
Cucurbita pepo                    GCTGCTATTCTATUTATTGGTGCATTAAGTTCGAAGAAGATCTUAATCTA
Silene latifolia                  GCTGCTATTCTATCTATTTGTGCATUAAGTTCGAAGAAGATCTUAATCTA
Lotus japonicus                   GCTGCTATTCUATCTATTTGTGCATTAAGTTCGACGAAGATATUAATCTA
Millettia pinnata                 GCTGCTATTCUATCTATTTGTGCATTAAGTTCGAAGAAGATCTUAATCTA
PB235_atp4                        GCTGCTATTCTATUTATTTGTGCATTAAGTTCGAAGAAGATCTUAATCTA
RRIC110_atp4                      GCTGCTATTCTATUTATTTGTGCATTAAGTTCGAAGAAGATCTUAATCTA
RRII105_atp4                      GCTGCTATTCTATUTATTTGTGCATTAAGTTCGAAGAAGATCTNAATCTA
RRIT251_atp4                      GCTGCTATTCTATNTATTTGTGCATTAAGTTCGAAGAAGATCTUAATCTA
BPM24_atp4                        GCTGCTATTCTATNTATTTGTGCATTAAGTTCGAAGAAGATCTUAATCTA
Spirodela polyrhiza               GCTGCTATTCCATCTATTTGTGCATCAAGTCCGAAGAAGATCTCAATCTA
RRIM600_atp4                      GCTGCTATTCTATNTATTTGTGCATTAAGTTCGAAGAAGATCTUAATCTA
Zea mays mays                     GCTGCTATTCUATUTATTTGTGCATUAAGTUCGAAGAAGATCTUAATCTA
Cycas taitungensis                GCTGCTATTCTATTTATTCGTGTATTAAGCTCGAAGAAGATCTCAATATA
Megaceros aenigmaticus            TTTGCTATTCCAATTTCTAGTGTTTCGAGTCCTAAACAAATCTTAATTTA
Phaeoceros laevis                 TTTGCTATTCTAATTTCTAGTGTTTTGAGTTCTAAACAAATCTTAATTTA
                                    ******** *  *  * ***  *  **  * *   * ** * *** **

Citrullus lanatus                 TAATGAAGAAATGATAGTAGCTUGTTGTTTTATAGGCTTTATTATATTCA
Cucurbita pepo                    TAATGAAGAAATGATAGTAGCTTGTTGTTTTATAGGCTTTATTATATTCA
Silene latifolia                  TAATGAAGAAATGATAGTAGCTUGTTGTTTTATAGGCTTTATUATATTCA
Lotus japonicus                   TAATGAAGAAATGATAGTAGCTUGTTGTTTTATAGGCTTCATCATATTCA
Millettia pinnata                 TAATGAAGAAATGATAGTAGCTUGTTGTTTTATAGGCTTCATCATATTCA
```

```
PB235_atp4                TAATGAAGAAATGATAGTAGCTUGTTGTTTTATAGGCTTTATCATATTCA
RRIC110_atp4              TAATGAAGAAATGATAGTAGCTUGTTGTTTTATAGGCTTTATCATATTCA
RRII105_atp4              TAATGAAGAAATGATAGTAGCTNGTTGTTTTATAGGCTTTATCATATTCA
RRIT251_atp4              TAATGAAGAAATGATAGTAGCTUGTTGTTTTATAGGCTTTATCATATTCA
BPM24_atp4                TAATGAAGAAATGATAGTAGCTUGTTGTTTTATAGGCTTTATCATATTCA
Spirodela polyrhiza       TAATGAAGAAATGATAGTAGCTCGTTGTTTTATAGGCTTTTTCATATTCA
RRIM600_atp4              TAATGAAGAAATGATAGTAGCTUGTTGTTTTATAGGCTTTATCATATTCA
Zea mays mays             TAATGAAGAAATGATAGTAGCTUGTTGTTTTATAGGCTTTCTCATATTAA
Cycas taitungensis        TAATGAAGAAATGATAGTAGCTTGTCGTTTTATAGGCTTTATCATATCCA
Megaceros aenigmaticus    TAATGAAGAAGTTCTTGTAGCTCGTAGTTTTATTGGTTTTGTTATATTTA
Phaeoceros laevis         TAATGAAGAAGTTTTTGTAGCTTGTAGTTTTATTGGTTTTGTTATATTTA
                          ********** *  * ****** ** ******* ** **  * ****  *

Citrullus lanatus         GTCGGAAGAGTTTAGGTAAGACTTTCAAAGCGACTUTCGACGGGAGAATC
Cucurbita pepo            GTCGTCAGAGTTTAGGTAAGACTTTCAAAGCGACTUTCGACGGGAGAATC
Silene latifolia          GTCGGAAGAGTTTAGGTAAGACTTTCAAAGTGACTCTCGACGAGAGAATC
Lotus japonicus           GTCGGAAGAGTTTAGGTAAGACTTTCAAAGTGACTCTCGACGGGAGAATC
Millettia pinnata         GTCGGAAGAGTTTAGGTAATACTTTCAAAGTGACTCTCGACGGGAGAATC
PB235_atp4                GTCGGAAGAGTTTAGGTAAGACTTTCAAAGTGACTCTCGACGGGAGAATC
RRIC110_atp4              GTCGGAAGAGTTTAGGTAAGACTTTCAAAGTGACTCTCGACGGGAGAATC
RRII105_atp4              GTCGGAAGAGTTTAGGTAAGACTTTCAAAGTGACTCTCGACGGGAGAATC
RRIT251_atp4              GTCGGAAGAGTTTAGGTAAGACTTTCAAAGTGACTCTCGACGGGAGAATC
BPM24_atp4                GTCGGAAGAGTTTAGGTAAGACTTTCAAAGTGACTCTCGACGGGAGAATC
Spirodela polyrhiza       GTCGGAAGAGTTTAGGTAAGACTTTTAAAGTCACTCTCGACGAGAGAATG
RRIM600_atp4              GTCGGAAGAGTTTAGGTAAGACTTTCAAAGTGACTCTCGACGGGAGAATC
Zea mays mays             GTCGGAAGAGTTTAGGTAAGACTTTCAAAGAAACTCTCGACGGGAGAATC
Cycas taitungensis        GTCAGAAGAGTTCGGGTAATACTCTCAAAGCGATTTTCGACGGGAGAATC
Megaceros aenigmaticus    GTGAAAAAACTTTTGGTGAAACTTTTGAAGCTACTTCTAATGCGAGAAGC
Phaeoceros laevis         GTGAAAAAACTTTTGGTGAAACTTTTGAAGCTACTTCTAATGCGAGAAGC
                          **    * * **  *** * *** *  ***  * *    * * *****

Citrullus lanatus         CAGGCTATTCAGGAAGAATUGCAGCAATTTCUCAATCCTAACGAAGTAGT
Cucurbita pepo            CAGGCTATTCAGGACGAATUGCAGCAATTTCUCAATCCTAACGAAGTAGT
Silene latifolia          CAGGCTATTCAGGAAGAATUGCAGCAATTCCUCAATCCTAACGAAGTAGT
Lotus japonicus           CAGGCTATTCAGGAAGAATUGCAGCAATTCCUCAATCCTAACGAAGTAGT
Millettia pinnata         CAGGCTATTCAGGAAGAATUGCAGCAATTCCUCAATCCTAACGAAGTAGT
PB235_atp4                CAGGCTATTCAGGAAGAATUGCAGCAATTCCCCAATCCTAACGAAGTAGT
RRIC110_atp4              CAGGCTATTCAGGAAGAATUGCAGCAATTCCCCAATCCTAACGAAGTAGT
RRII105_atp4              CAGGCTATTCAGGAAGAATUGCAGCAATTCCCCAATCCTAACGAAGTAGT
RRIT251_atp4              CAGGCTATTCAGGAAGAATUGCAGCAATTCCCCAATCCTAACGAAGTAGT
BPM24_atp4                CAGGCTATTCAGGAAGAATUGCAGCAATTCCCCAATCCTAACGAAGTAGT
Spirodela polyrhiza       GAGTCTATTCAGGAAGAATCGCAGCAATTCTCCAATCCTAACGAAGTCGT
RRIM600_atp4              CAGGCTATTCAGGAAGAATNGCAGCAATTCCCCAATCCTAACGAAGTAGT
Zea mays mays             GAGTCTATTCAGGAATCATTGCAGCAATTCTTCAATCCTAACGAAGTCAT
Cycas taitungensis        GGAGCTATTCAGAAAGAATTGCAGCAATTCCCCAATCCCAACGAAGTTGT
Megaceros aenigmaticus    GAAGCAATTTTTTCAGAGTTACAGCAATTGATAAGTTCTAAAGAGGCTCT
Phaeoceros laevis         GAAGCAATTTTTTCAGAGTTACAGCAATTGATAAGTTCTAAAGAGGCTCT
                              * ***         *  ********    * * * ** ** *   *

Citrullus lanatus         TCUTUUGGAATCCAATGAACAACAACGATTACTTA------GGATCAGTT
Cucurbita pepo            TCUTUUGGAATCCAATGAACAACAACGATTACTTA------GGATCAGTT
Silene latifolia          TCUTUUGGAATCCAATGAACAACAACGATTACTTA------GGATCAGCT
Lotus japonicus           TCUTUUGGAATCTAATGAACAACAACGATTACTTA------GGATCAGTT
Millettia pinnata         TCUTUUGGAATCTAATGAACAACAACGATTACTTA------GGATCAGTT
PB235_atp4                TCCTCCGGAATCCAATGAACAACAACGATTACTTA------GGATCAGCT
RRIC110_atp4              TCCTCCGGAATCCAATGAACAACAACGATTACTTA------GGATCAGCT
RRII105_atp4              TCCTCCGGAATCCAATGAACAACAACGATTACTTA------GGATCAGCT
RRIT251_atp4              TCCTCCGGAATCCAATGAACAACAACGATTACTTA------GGATCAGCT
BPM24_atp4                TCCTCCGGAATCCAATGAACAACAACGATTACTTA------GGATCAGCT
Spirodela polyrhiza       TCCTCCGGAATCCAATGAACAACAACGATTACTTA------GGATCAGCT
RRIM600_atp4              TCCTCCGGAATCCAATGAACAACAACGATTACTTA------GGATCAGCT
Zea mays mays             TCTGGAGGAATCCAATGAACAACAACGATTACTTAATCTACGGATCAGCT
```

```
Cycas taitungensis                TTCTCCGGAATCCAATGAACAACAACAATTACTTA------GGATCAGCT
Megaceros aenigmaticus            GTTGTCCGAGTTAAAGAAACAGCATGAATTATGCA------GTATAAGCT
Phaeoceros laevis                 GTTGTCCGAGTTAAAGAAACAGCATAAATTATGTA------GTATAAGTT
                                         ** *  **  **** **   ****   *      * ** ** *

Citrullus lanatus                 TGCGAATTTGTGGCACUGTA------GTCGAATCATTACCAA------TG
Cucurbita pepo                    TGCGAATTTGTGGCACUGTA------ATCGAATCATTACCAA------TG
Silene latifolia                  TGCGAATTTGTGGCACCGTA------GTAGAATCATTACCAA------TG
Lotus japonicus                   TGCGAATTTGTGGCACCGTA------GTAGAATCATTACCAA------TG
Millettia pinnata                 TGCGAATTTGTGGCACCGTA------GTAGAATCATTACCAA------TG
PB235_atp4                        TGCGAATTTGTGGCACCGTA------GTAGAATCATTACCAA------TG
RRIC110_atp4                      TGCGAATTTGTGGCACCGTA------GTAGAATCATTACCAA------TG
RRII105_atp4                      TGCGAATTTGTGGCACCGTA------GTAGAATCATTACCAA------TG
RRIT251_atp4                      TGCGAATTTGTGGCACCGTA------GTAGAATCATTACCAA------TG
BPM24_atp4                        TGCGAATTTGTGGCACCGTA------GTAGAATCATTACCAA------TG
Spirodela polyrhiza               TGCGAATTTGTGGCACCGTA------GTAGAATCATTACCAA------CG
RRIM600_atp4                      TGCGAATTTGTGGCACCGTA------GTAGAATCATTACCAA------TG
Zea mays mays                     TGCGAATTTGCAGCACCGTAAAAGTAGTAGAATUATTACCAG------CG
Cycas taitungensis                TGCGT-TCAATTACGCCAGAGA--TTGTAGAATCATTACCAAATGAGACG
Megaceros aenigmaticus            TGCG---TTCAGGTACGCAAATGATTGGAGAATCATGCATAAATGATATG
Phaeoceros laevis                 TGCG---TTCAGGTACGCAAATGATTGGAGAATCATGCATAAATGATATG
                                  ****           *   *         **** **    *        *

Citrullus lanatus                 G---CGCGCTATGCGCCTAAGTGCGAAAAGACAGTGCAAGCTTTGTTATG
Cucurbita pepo                    G---CGCGCTATGCGCCTAAGTGCGAAAAGACAGTGCAAGCTTTGGTATG
Silene latifolia                  G---CACGCTGTGCGCCTAAGTGCGAAAAGACAGTGCAAGCTTTGTTATG
Lotus japonicus                   G---CACGCTGTGCGCCTAAGTGCGAAAAGACAGTGCAAGCTTTGTTATG
Millettia pinnata                 G---CACGCTGTGCGCCTAAGTGCGAAAAGACAGTGCAAGCTTTGTTATG
PB235_atp4                        G---CACGCTGTGCGCCTAAGTGCGAAAAGACAGTGCAAGCTTTGTTATG
RRIC110_atp4                      G---CACGCTGTGCGCCTAAGTGCGAAAAGACAGTGCAAGCTTTGTTATG
RRII105_atp4                      G---CACGCTGTGCGCCTAAGTGCGAAAAGACAGTGCAAGCTTTGTTATG
RRIT251_atp4                      G---CACGCTGTGCGCCTAAGTGCGAAAAGACAGTGCAAGCTTTGTTATG
BPM24_atp4                        G---CACGCTGTGCGCCTAAGTGCGAAAAGACAGTGCAAGCTTTGTTATG
Spirodela polyrhiza               G---CACGCTGTACGCCTAAGTGCGAAAAGACAGTGCAAGCTTTGTTATG
RRIM600_atp4                      G---CACGCTGTGCGCCTAAGTGCGAAAAGACAGTGCAAGCTTTGTTATG
Zea mays mays                     G---CACGCTGTGCGCCTAAGTGUGAAAAGACAGTGCAAGCTTTGTTATG
Cycas taitungensis                G---CACGCTGTGCGCCTAAGTGCGAAAAGACAGTGCAAGCTGTGTTATG
Megaceros aenigmaticus            GTTACGCGCTGTGCGCCTAAGTGCAAACAGACAGTGCAAGCTATATTATG
Phaeoceros laevis                 GTTACGCGCTGCGCGCCTAAGTGCAAACAGACAGTGCAAGCTGTATTGTG
                                  *   * ****   **********  ** ************** *  * **

Citrullus lanatus                 CCGAAACCTAAATGTTAAGTUAGCAACACTTCUAAATGCCAUTTCTTCCC
Cucurbita pepo                    CCGAAACCTAAATGTTAAGTUAGCAACACTTCUAAATGCCAUTGATTCCC
Silene latifolia                  TCGAAACCTAAATGTTAAGTUAGCAACACTTCUAAATGCCACTTCTTCCC
Lotus japonicus                   CCGAAACCTAAATGTTAAGTUAGCAACACTTCUAAATGCCAUTTCTTCCC
Millettia pinnata                 CCGAAACCTAAATGTTAAGTUAGCAACACTTCUAAATGCCAUTTCTTCCC
PB235_atp4                        CCGAAACCTAAATGTTAAGTUAGCAACACTTTCAAATGCCAUTTCTTCCC
RRIC110_atp4                      CCGAAACUTAAATGTTAAGTUAGCAACACTTTCAAATGCCAUTTCTTCCC
RRII105_atp4                      CCGAAACUTAAATGTTAAGTUAGCAACACTTTCAAATGCCAUTTCTTCCC
RRIT251_atp4                      CCGAAACUTAAATGTTAAGTUAGCAACACTTTCAAATGCCAUTTCTTCCC
BPM24_atp4                        CCGAAACCTAAATGTTAAGTUAGCAACACTTTCAAATGCCAUTTCTTCCC
Spirodela polyrhiza               CCGAAACCTAAATGTTAAATCAGCAACACTTCTAAATGCCACTTCTTCCC
RRIM600_atp4                      CCGAAACCTAAATGTTAAGTCAGCAACACTTTCAAATGCCANTTCTTCCC
Zea mays mays                     CCGAAACCTAAATGTCAAGTUAGCAACACTTCTAAATGCCAUTTCTTCCC
Cycas taitungensis                CCGAAACCTAAATGTAAAGTTAGCAACACTTCCAAATGCCATTTATTCCC
Megaceros aenigmaticus            CCAACAAATGGAACAGAAGTTAAAAACACTGTTAGCTATTCAAGAGCATT
Phaeoceros laevis                 CCAACAAATGGAACAGAAGTTAAAAACACTGTTAGCTATTCAAGAGCATT
                                   * * *  *  *    ** * *  ******   *  *

Citrullus lanatus                 GTCGCATCCGTCTTCAGGACGATCTAGTCACAGGTTTTCACTTCTCAGTG
Cucurbita pepo                    GTCGCATCCGTCTTCAGGACGATCTAGTCACAGGGTTTCACTTCTCAGTG
Silene latifolia                  GTCGCACCCGTCTTCAGGACGATCTAGTCACAGGGTTTCACTTCTCAGTG
```

```
Lotus japonicus                   GTCGCATCCGTCTTCAGGACGATCTAGGCACAAAGTTTAACTTATTAGTT
Millettia pinnata                 GTCGCATCCGTCTTCAGGACGATCTAGGCACAAAGTTTAACTTATTAGTT
PB235_atp4                        GTCGCATCCGTCTTCAGGACGATCTAGTCACAGGTTTTCACTTCTCAGTG
RRIC110_atp4                      GTCGCATCCGTCTTCAGGACGATCTAGTCACAGGTTTTCACTTCTCAGTG
RRII105_atp4                      GTCGCATCCGTCTTCAGGACGATCTAGTCACAGGTTTTCACTTCTCAGTG
RRIT251_atp4                      GTCGCATCCGTCTTCAGGACGATCTAGTCACAGGTTTTCACTTCTCAGTG
BPM24_atp4                        GTCGCATCCGTCTTCAGGACGATCTAGTCACAGGTTTTCACTTCTCAGTG
Spirodela polyrhiza               GTCGCATCCGTCTTCAGGACGATATAGTCACAGGTTTTCACTTCTCAGTG
RRIM600_atp4                      GTCGCATCCGTCTTCAGGACGATCTAGTCACAGGTTTTCACTTCTCAGTG
Zea mays mays                     GTCGCATCCGTCTTCAGGACGATATAGTCACAGGTTTTCACTTTTCAGTG
Cycas taitungensis                GTCGCATCCGTCTTCGGGACGATATAGTCACAGGTTTTCACTTCTCAGTG
Megaceros aenigmaticus            CTCGCATGAGTTTACAGGAGAAGATAGTCACTTGTTTTCGCGAAACAGTT
Phaeoceros laevis                 CTCGCATGAGTTTACAGGAGAAGATAGTAACTTGTTTTCGCGAAACAGTT
                                   *****   ** * * ***  *  ***  **    ***  *     ***

Citrullus lanatus                 AGTGAAAGATTTGTCCCCGGGTGTAC------GTTGAAAGCTTCTATAGT
Cucurbita pepo                    AGTGAAAGATTTGTCCCCGGGTGTAC------GTTGAAAGCTTCTATAGT
Silene latifolia                  AGTGAAAGATTTGTCCCCGGGTCTAC------GTTGAAAGCTTCTATAGT
Lotus japonicus                   AGGAGGAGATTTTGCCCCCAGTGTAT------CTCGAAAGCAGAAAAAAT
Millettia pinnata                 AGGAGGAGATTTTGCCCCCAGTCTAT------CTCGAAAGCAGAAAAAAT
PB235_atp4                        AGTGAAAGATTTGTCCCCGGGTGTAC------GTTGAAAGCTTCTATAGT
RRIC110_atp4                      AGTGAAAGATTTGTCCCCGGGTGTAC------GTTGAAAGCTTCTATAGT
RRII105_atp4                      AGTGAAAGATTTGTCCCCGGGTGTAC------GTTGAAAGCTTCTATAGT
RRIT251_atp4                      AGTGAAAGATTTGTCCCCGGGTGTAC------GTTGAAAGCTTCTATAGT
BPM24_atp4                        AGTGAAAGATTTGTCCCCGGGTGTAC------GTTGAAAGCTTCTATAGT
Spirodela polyrhiza               AGTGAAAGATTTGTCCCCGGGTCTAC------GTTGAAAGCTTCTATCGT
RRIM600_atp4                      AGTGAAAGATTTGTCCCCGGGTGTAC------GTTGAAAGCTTCTATAGT
Zea mays mays                     AGTGAAAGATTAGTATCCGGGTCTACAACTTTGGTAGAAGCTTUTACCGT
Cycas taitungensis                AGTGAAAAATTTGTCCCCTGCACCACCGATACGAAAAAAGCTTTTATCGC
Megaceros aenigmaticus            TGTGACGAATTTCGCTTT---TCCAAA----TTGCGAAAACATCAGT--C
Phaeoceros laevis                 TGTGACGAATTTCGCTTT---TCCAAA----TTGCGAAAACATCAGT--C
                                   *      ***             *            ** *

Citrullus lanatus                 CGAACTCATTCGAGAGGGCTTGGTGGT----CTTAAGAA---TGGTTCGG
Cucurbita pepo                    CGAACTCATTCGAGAGGGCTTGGTGGT----CTTAAGAA---AGGTTCGG
Silene latifolia                  TGAACTCATTCGAGAAGGCTTGGCGGT----CTTAAGAA---TGGTTCGG
Lotus japonicus                   AGAACTCATTCGAGAGAGCTTGGTGGT----CTTAAGAA---TGGTTCGG
Millettia pinnata                 AGAACTCATTCGAGAGAGCTTGGTGGT----CTTAAGAA---TGGTTCGG
PB235_atp4                        AGAACTCATTCGAGAGGGCTTGGTGGT----CTTAAGAA---TGGTTCGG
RRIC110_atp4                      AGAACTCATTCGAGAGGGCTTGGTGGT----CTTAAGAA---TGGTTCGG
RRII105_atp4                      AGAACTCATTCGAGAGGGCTTGGTGGT----CTTAAGAA---TGGTTCGG
RRIT251_atp4                      AGAACTCATTCGAGAGGGCTTGGTGGT----CTTAAGAA---TGGTTCGG
BPM24_atp4                        AGAACTCATTCGAGAGGGCTTGGTGGT----CTTAAGAA---TGGTTCGG
Spirodela polyrhiza               AGAACTCATTUGAGAGGGCTTGGTAGT----CTTAAGAA---GGGTTCGG
RRIM600_atp4                      AGAACTCATTCGAGAGGGCTTGGTGGT----CTTAAGAA---TGGTTCGG
Zea mays mays                     AGAACAAATTCGAGAGGCCTTCTTATTAGAACCCAGAGACC-TAATTCGA
Cycas taitungensis                AGAACCGATCCGAGAAGGCTTGGTGGT----ATCAATAACGGTTTCTCGA
Megaceros aenigmaticus            TAAACTAGTTCAACAAAGCATGGTATT---ATTGAAAGATGGGGTTCTGA
Phaeoceros laevis                 TAAACTAGTTCAACAAAGCATGGTATT---ATTGAAAGATGGGGTTCTGA
                                    ***   *   * *   * *     *       *   *         *

Citrullus lanatus                 GTGGGGGGTTCTCTT----AAGAAT-----AAAGAAGACAAATAGAATCT
Cucurbita pepo                    G--GGGTTCTCTCTT----AAGAATCTCAAAAAGAAGAAAAATAGAATCT
Silene latifolia                  GTAGGAGGTTCTCTT----AAGAAT-----AAAGAAGACAAATAGAATCT
Lotus japonicus                   GTGGGGGGTTCTCTT----AAGAAT-----AAATAAGCCGAATAGAATCT
Millettia pinnata                 GTGGGGGGTTCTTTT----AAGAAT-----AAATAAGACGAATAGAATCT
PB235_atp4                        GTGGGGGGTTCTCTT----AAGAAT-----AAAGAAGATGAATAG-----
RRIC110_atp4                      GTGGGGGGTTCTCTT----AAGAAT-----AAAGAAGATGAATAG-----
RRII105_atp4                      GTGGGGGGTTCTCTT----AAGAAT-----AAAGAAGATGAATAG-----
RRIT251_atp4                      GTGGGGGGTTCTCTT----AAGAAT-----AAAGAAGATGAATAG-----
BPM24_atp4                        GTGGGGGGTTCTCTT----AAGAAT-----AAAGAAGATGAATAG-----
Spirodela polyrhiza               GTGGGGGGTTCTCTTTTTTAAGAAT-----AAGGAAGAGGAATAGAATCT
```

```
RRIM600_atp4                      GTGGGGGGTTCTCTT----AAGAAT-----AAAGAAGATGAATAG-----
Zea mays mays                     G-AAGGCTTTATAGTCCTCAGAAAGGT---GAGGGTGGGGGGTATCCCCG
Cycas taitungensis                AATGGAGGGCATUGACCT-AAGAACGG-GAGAATTGAAGGGGAATGATGA
Megaceros aenigmaticus            AATGAAAAATTTTGCAC--AAAGATGGCTTTTTTCCACAAACCACAAGTG
Phaeoceros laevis                 AATGAAAAATTTTGCAC--AAAGATGGCTTTTTTCCACAAACCACAAGTG
                                             *       *   *                   *

Citrullus lanatus                 AATTCATGTTCATGCTAACAGAAGAGCGAATCCAATACCAAGACGACTTC
Cucurbita pepo                    AATTCATGTTCATGCTAACAGAAGAGCGAATCCAATACCAAGACGACTTC
Silene latifolia                  AATTCATGTTCATGCTAACAGAAGAGCGGATCCAATACCAAGACGACTTC
Lotus japonicus                   AATTCATGTTCATGCTAACAGAAGAGCGGATCCAATACACAGACAACTTC
Millettia pinnata                 AATTCATGTTCATGCTAACAGAAGAGCGGATCCAATACACAGACAACTTC
PB235_atp4                        --------------------------------------------------
RRIC110_atp4                      --------------------------------------------------
RRII105_atp4                      --------------------------------------------------
RRIT251_atp4                      --------------------------------------------------
BPM24_atp4                        --------------------------------------------------
Spirodela polyrhiza               CATT----------------------------------------------
RRIM600_atp4                      --------------------------------------------------
Zea mays mays                     GGACCTGTGGAGACGGGGTGGGCCTGTAGCTCTGAGGATTAGAGCACGTG
Cycas taitungensis                GAATAATTATAGTTATAGCATCTCTTC--TTCCAAAGCTCGATCGCCGCC
Megaceros aenigmaticus            CGACGCTCTGCATGCTATAGTCGCTGGGTCTCGAGCAACCC---------
Phaeoceros laevis                 CGACGCTCTGCATGCTATAGTCCCTGGGTCTCGAGCCACCC---------


Citrullus lanatus                 TTTCTCAGGAAGTGCAGCAAGTACTTGAGTCATTT---------------
Cucurbita pepo                    TTTCTC--------------------------------------------
Silene latifolia                  TTTCTCAGAAA----AGCAAGTACTTGAAGAATTTTTTG-----------
Lotus japonicus                   TTTCTCAAAAAGTACTTGCTGCACT-------------------------
Millettia pinnata                 TTTCTCAAAAAGTACTTGCTGCA---------------------------
PB235_atp4                        --------------------------------------------------
RRIC110_atp4                      --------------------------------------------------
RRII105_atp4                      --------------------------------------------------
RRIT251_atp4                      --------------------------------------------------
BPM24_atp4                        --------------------------------------------------
Spirodela polyrhiza               --------------------------------------------------
RRIM600_atp4                      --------------------------------------------------
Zea mays mays                     GCTACGAACCACGGTGTCGGGGGTTCGAATCCACTTCTGAGCGGGCTGGC
Cycas taitungensis                AAACCCAAT-----------------------------------------
Megaceros aenigmaticus            --------------------------------------------------
Phaeoceros laevis                 --------------------------------------------------


Citrullus lanatus                 -----------------------------
Cucurbita pepo                    -----------------------------
Silene latifolia                  -----------------------------
Lotus japonicus                   -----------------------------
Millettia pinnata                 -----------------------------
PB235_atp4                        -----------------------------
RRIC110_atp4                      -----------------------------
RRII105_atp4                      -----------------------------
RRIT251_atp4                      -----------------------------
BPM24_atp4                        -----------------------------
Spirodela polyrhiza               -----------------------------
RRIM600_atp4                      -----------------------------
Zea mays mays                     GGCTCCACCGGGAGCGAGCCTACTGAAGG
Cycas taitungensis                -----------------------------
Megaceros aenigmaticus            -----------------------------
Phaeoceros laevis                 -----------------------------


###################
```

atp6 multiple sequence alignment

```
Megaceros aenigmaticus             --------------------------------------------------
Phaeoceros laevis                  --------------------------------------------------
Cycas taitungensis                 --------------------------------------------------
Beta macrocarpa                    --------------------------CAAAGACTCCCATGCCTTTCTTGG
Beta vulgaris maritima             --------------------------CAAAGACTCCCATGCCTTTCTTGG
Beta vulgaris vulgaris             --------------------------CAAAGACTCCCATGCCTTTCTTGG
Citrullus lanatus                  ---------------------------------------GCCTTTCTTGG
Cucurbita pepo                     ------------------------CCTCTCCCATCCCATGCCTTTCTTGG
Arabidopsis thaliana               GCATTAACGCAAGATCCC--------------GAAATGGGCTTGATTTGG
Brassica napus                     -----------------C-------------GACCTCG-CAAGGTTTT--
Silene latifolia                   GAATTGACGGGGGATCCTCATAATCATAAATAAAAGGGCTCAGATTATGG
RRII105_atp6                       --------------------------------------------------
PB235_atp6                         --------------------------------------------------
BPM24_atp6                         --------------------------------------------------
RRIC110_atp6                       --------------------------------------------------
RRIT251_atp6                       --------------------------------------------------
RRIM600_atp6                       --------------------------------------------------
Millettia pinnata                  --------------------------------------------------
Oryza sativa Japonica              GATTATAACGTGAACTCGTTGAAGAGAAGGAGACAAGCAGAAATAGACGC


Megaceros aenigmaticus             --------------------------------------------------
Phaeoceros laevis                  --------------------------------------------------
Cycas taitungensis                 --------TTTCCTGGTCAAACCAACCGGCGATTCTCGCTTGCTCGGAGG
Beta macrocarpa                    TCGGACCAACCCAACCGGTGATT--------TCCGACAAGTCTTTCTTC-
Beta vulgaris maritima             TCGGACCAACCCAACCGGTGATT--------TCCGACAAGTCTTTCTTC-
Beta vulgaris vulgaris             TCGGACCAACCCAACCGGTGATT--------TCCGACAAGTCTTTCTTC-
Citrullus lanatus                  TTGGACCAACTCAACCGGCGATTGATTGATTTCCTACAAGTCAGTCTTCC
Cucurbita pepo                     TTGGACCAACCCAACCGGCGATT--------TCCGACAAGTCTTTCTTC-
Arabidopsis thaliana               GCGAAGCTTTACATGCGGAAAGCGACAATGTCCCTTTTTTGCAGTCCATT
Brassica napus                     -TGAAGCGGCCGAAGCGGGAAGTGACAATACCGCTTTTCTTCAGCACATT
Silene latifolia                   CCGAGGCTTCGCATGCAGAAAGTCAGAATATTGAGTTTATGGATTACATT
RRII105_atp6                       --------------------------------------------------
PB235_atp6                         --------------------------------------------------
BPM24_atp6                         --------------------------------------------------
RRIC110_atp6                       --------------------------------------------------
RRIT251_atp6                       --------------------------------------------------
RRIM600_atp6                       --------------------------------------------------
Millettia pinnata                  --------------------------------------------------
Oryza sativa Japonica              TTTTTTTGAACCATTTGAGAGGGCGCAGCGTATCCGTTTCAATAACTGGC


Megaceros aenigmaticus             --------------------------------------------------
Phaeoceros laevis                  --------------------------------------------------
Cycas taitungensis                 GGCAAGCAAAAACGAGT--CTTTCAATTTTGGGAGCAAGAAGTACGGGAA
Beta macrocarpa                    ----ATTTTTGAGCAAG--AAGCGGAACTACAGGGATGAAATGAAAAGTG
Beta vulgaris maritima             ----ATTTTTGAGCAAG--AAGCGGAACTACAGGGATGAAATGAAAAGTG
Beta vulgaris vulgaris             ----ATTTTTGAGCAAG--AAGCGGAACTACAGGGATGAAATGAAAAGTG
Citrullus lanatus                  TGAATTGGTCGAGCAAG--AAGCGGAACGACAGGGATGAAATGCTAAGTG
Cucurbita pepo                     ---ATTTTGCGAGCAAG--AAGCGGAACTACAGGGATGAAATGAAAAGTG
Arabidopsis thaliana               GCTGATGATTTAACCCA--AAACGGAGTATCCGGGGAAGCCTTTCAAGAA
Brassica napus                     TTGGATGATTTGAGCGA--AAACGGAGTACAAAGTTCAGCCTTTAAGGAG
Silene latifolia                   GCGACGGATTTGGACAC--AAACGGAGTAGCCAGTGAGTCCTATAGGGAA
RRII105_atp6                       --------------------------------------------------
PB235_atp6                         --------------------------------------------------
BPM24_atp6                         --------------------------------------------------
RRIC110_atp6                       --------------------------------------------------
RRIT251_atp6                       --------------------------------------------------
RRIM600_atp6                       --------------------------------------------------
Millettia pinnata                  -----TGTTATTTACCA--ATATGGATCA-CAGTTGAATCCTACCGGGAG
Oryza sativa Japonica              AGAACGGAATAGAGTTGTTAGATGGGGCTGAATGGAGGAACGGCGATATA
```

```
Megaceros aenigmaticus      ------------------------AUGGCTTGCAGTCCACCAGAACAATT
Phaeoceros laevis           ------------------------AUGGCTTGCAGTCCACCAGAACAATT
Cycas taitungensis          AGGGATGAAATAAAAGTCTCTATTATGCCAAACAGCCCACTTGAGCAATT
Beta macrocarpa             TTTAT------------TAUGATTACGCCCAACAGCCCACTTGAGCAATT
Beta vulgaris maritima      TTTAT------------TAUGATTACGCCCAACAGCCCACTTGAGCAATT
Beta vulgaris vulgaris      TTTAT------------TAUGATTACGCCCAACAGCCCACTTGAGCAATT
Citrullus lanatus           TTTCT------------GACGATTACGCCCAACAGCCCACTTGAGCAATT
Cucurbita pepo              TTTCT------------TACGATTACGCCCAACAGCCCACTTGAGCAATT
Arabidopsis thaliana        GCTCTGAATATAGTGGGACAGGCGGCGGCCTCC---CCACTGGACCAATT
Brassica napus              GCTATGAATCAAATAGGGCTGGTGGCGCAGTCC---CCACTTGACCAATT
Silene latifolia            GCTCTGGCGCTAATAGGGGAGGTGGTGCCCAACAGCCCACTTGACCAATT
RRII105_atp6                --------------------------------------------------
PB235_atp6                  --------------------------------------------------
BPM24_atp6                  --------------------------------------------------
RRIC110_atp6                --------------------------------------------------
RRIT251_atp6                --------------------------------------------------
RRIM600_atp6                --------------------------------------------------
Millettia pinnata           CCCCT--------CTGCTTCGGACTCCATTAACAGCCCGCTTGATCAATT
Oryza sativa Japonica       GTTATCCCTGGAGGCGGCGGACCAGTAATTTCAAGCCCCTTGGATCAATT


Megaceros aenigmaticus      TGCAATTATTCCATTGATTCCTATTCATATAGGAAACTTGTATTCTCCAT
Phaeoceros laevis           TGCAATTATTCCATTGATTCCTATTCATATAGGAAACTTGTATTCTCTAT
Cycas taitungensis          TGCCATTATCCCATTGATTCCTATGGATATAGGAAACTTGTATTTCTCAT
Beta macrocarpa             TTCCATTCTCCCATTGATTCCTATGAAAATAGGAAACTTGTATTTCTCAT
Beta vulgaris maritima      TTCCATTCTCCCATTGATTCCTATGAAAATAGGAAACTTGTATTTCTCAT
Beta vulgaris vulgaris      TTCCATTCTCCCATTGATTCCTATGAAAATAGGAAACTTGTATTTCTCAT
Citrullus lanatus           TGCCATTCTCCCATTGATTCCTATGAATATAGGAAACTTTTATTTCTCAT
Cucurbita pepo              TGCCATTCTCCCATTGATTCCTATGAATATAGGAAACTTTTATTTCTCAT
Arabidopsis thaliana        TGAGATTGTCCCATTGATTCCTATGCATATCGGAAACTTCTATTTCTCAT
Brassica napus              TGAGATTGTCCCATTGATTCCTATGAATATCGGAAACTTCTATTTCTCAT
Silene latifolia            TGCCATTCTCCCATTGATTCCTATGAAACTAGGAAACTTGTATTTCTCAT
RRII105_atp6                ----------------------ATGAAGATAGGAGACTTGTATTTCTCAT
PB235_atp6                  ----------------------ATGAAGATAGGAGACTTGTATTTCTCAT
BPM24_atp6                  ----------------------ATGAAGATAGGAGACTTGTATTTCTCAT
RRIC110_atp6                ----------------------ATGAAGATAGGAGACTTGTATTTCTCAT
RRIT251_atp6                ----------------------ATGAAGATAGGAGACTTGTATTTCTCAT
RRIM600_atp6                ----------------------ATGAAGATAGGAGACTTGTATTTCTCAT
Millettia pinnata           TGAGATAATCCCATTGATTGATATGAAGATAGGTGACTTGTATTTCTCAT
Oryza sativa Japonica       TTTCATTGATCCATTATTTGGTCTTGATATGGGTAACTTTTATTTATCAT
                                                   *  *  * **  **** ****    **

Megaceros aenigmaticus      CTACCAATTCATCTTTGTTCATGCCACCAACTCTCAGTCTAGTATTGCTT
Phaeoceros laevis           CTATCAATTCATTTTTGTTCATGCCACTAACTATCAGTCTAGTATTGCTT
Cycas taitungensis          TCACAAATCCATCTTTGTCCATGCCGCTAACTATCAGTTCAGTCCTGCTT
Beta macrocarpa             TCACAAATUCATCTTTGTTTATGCTGCTAACTCTCAGTTTGGTCCTACTT
Beta vulgaris maritima      TCACAAATUCATCTTTGTTTATGCTGCTAACTCTCAGTTTGGTCCTACTT
Beta vulgaris vulgaris      TCACAAATUCATCTTTGTTTATGCTGCTAACTCTCAGTTTGGTCCTACTT
Citrullus lanatus           TCACAAATUCATCTTTGTTTATGCTGCTAACTCTCAGTTTGGTCCTACTT
Cucurbita pepo              TCACAAATUCUTCTTTCTTTATGCTGCTAACTCTCAGTTTGGTCCTACTT
Arabidopsis thaliana        TCACAAATUCATCTTTGTTCATGCTGCTAACTCTGAGTTTTTTCCTACTT
Brassica napus              TCACAAATUCATCTTTGTTCATGCTGCTAACTCTGAGTTTTTTCCTACTT
Silene latifolia            TCACAAATUCATCTTTGTTTATGCTGCTAACTCTCAGTTTGGTCCTACTT
RRII105_atp6                TCACAAATUCATCTTTGTTTATGCTTCTAACTCTCAGTTTGGTCCTACTT
PB235_atp6                  TCACAAATUCATCTTTGTTTATGCTTCTAACTCTCAGTTTGGTCCTACTT
BPM24_atp6                  TCACAAATUCATCTTTGTTTATGCTTCTAACTCTCAGTTTGGTCCTACTT
RRIC110_atp6                TCACAAATUCATCTTTGTTTATGCTTCTAACTCTCAGTTTGGTCCTACTT
RRIT251_atp6                TCACAAATUCATCTTTGTTTATGCTTCTAACTCTCAGTTTGGTCCTACTT
RRIM600_atp6                TCACAAATCCATCTTTGTTTATGCTTCTAACTCTCAGTTTGGTCCTACTT
Millettia pinnata           TCACAAATUCUTCTTTGTTTATGCTGCTCACTCTCAGTTTGGTCTTACTG
```

```
Oryza sativa Japonica             TCACAAATGAATCCTTGTUTATGGCGGTAACTGTCGTTTTGGTGCUATCT
                                    *  ***   *  ** *  ***      *** *   *    *

Megaceros aenigmaticus            CTAGTTTATTTCGTCACCCCGAATGGAGG---ACACTTAGTACTAAATGC
Phaeoceros laevis                 CTAGTTTATTTTGTCACCCCGAATGGAGG---ACACTTAGTATTAAATGC
Cycas taitungensis                TTAGTCCATCTTGTTACTAAGAACGGAGGGGGAAACTCAGTACCAAATGC
Beta macrocarpa                   CTGCTTCATTTTGTTACTAAAAAGGGAGGAGGAAACTUAGTACCAAATGT
Beta vulgaris maritima            CTGCTTCATTTTGTTACTAAAAAGGGAGGAGGAAACTUAGTACCAAATGT
Beta vulgaris vulgaris            CTGCTTCATTTTGTTACTAAAAAGGGAGGAGGAAACTUAGTACCAAATGT
Citrullus lanatus                 CTGGTTCATTTTGTTACTAAAAACGGAGGAGGAAACTUAGTACCAAATGC
Cucurbita pepo                    CTGGTTTATTTTGTTACTAAAAACGGAGGAGGAAACTUAGTACCAAATGC
Arabidopsis thaliana              CTGATTCATTTTGTTACTAAAAAGGGAGGAGGAAACTTAGTCCCAAATGC
Brassica napus                    CTGATTCATTTTATTACTAAAAAGGGAGGAGGAAACTTAGTCCCAAATGC
Silene latifolia                  UTGCTTCATTTTGTGACTAAAAAGGGAGGAGGAAACTUAGTACCAAATGT
RRII105_atp6                      CTGTTTTATTTTGTTACTAAAAAGGGAGGAGGAAAGTUAGTACCAAATGC
PB235_atp6                        CTGTTTTATTTTGTTACTAAAAAGGGAGGAGGAAAGTUAGTACCAAATGC
BPM24_atp6                        CTGTTTTATTTTGTTACTAAAAAGGGAGGAGGAAAGTUAGTACCAAATGC
RRIC110_atp6                      CTGTTTTATTTTGTTACTAAAAAGGGAGGAGGAAAGTUAGTACCAAATGC
RRIT251_atp6                      CTGTTTTATTTTGTTACTAAAAAGGGAGGAGGAAAGTUAGTACCAAATGC
RRIM600_atp6                      CTGTTTTATTTTGTTACTAAAAAGGGAGGAGGAAAGTNAGTACCAAATGC
Millettia pinnata                 CTGGTTCATTTTGTTACTAAAAATGGAGGAGGAAAGTUAGTACCCAATGC
Oryza sativa Japonica             TTATTTGGAGTTGTTACGAAAAAGGGCGGGGGAAAGTUAGTGCCAAATGC
                                   *  *     *  * **    ** ** **   * * * ***    ****

Megaceros aenigmaticus            TTGGCAACTCTTCGTGGAAATGATTTATGATTTTGTGTTTAACTTGGTGA
Phaeoceros laevis                 TTGGCAATTCTTCGTGGAAATGATTTATGATTTTGTGTTTAACTTGGTGA
Cycas taitungensis                TTGGCAATCCCTGGTAGAGCTTATTTATGATTTCGTGCCGAACCCGGTGA
Beta macrocarpa                   TTGGCAATCCTTGGTAGAGCTTATTTATGATTTCGTGCTGAACCTGGTAA
Beta vulgaris maritima            TTGGCAATCCTTGGTAGAGCTTATTTATGATTTCGTGCTGAACCTGGTAA
Beta vulgaris vulgaris            TTGGCAATCCTTGGTAGAGCTTATTTATGATTTCGTGCTGAACCTGGTAA
Citrullus lanatus                 TTGGCAATCCTTGGTAGAGCTTATTTATGATTTCGTGCUGAACCTGGTAA
Cucurbita pepo                    TTGGCAATCCTTGGTAGAGCTTATTTATGATTTCGTGTUGAACCTGGTAA
Arabidopsis thaliana              TTGGCAATCCTTGGTAGAGCTTCTTTATGATTTCGTGCTGAACCTGGTAA
Brassica napus                    TTGGCAATCCTTGGTAGAGCTTCTTTATGATTTCGTGCTGAACCTGGTAA
Silene latifolia                  TTGGCAATCCTTGGTAGAGCTTATTTATGATTTCGTGCTGAACCTGGTAA
RRII105_atp6                      TTGGCAATCCTTGGTAGAGCTTCTTTATGATTTCGTGCUGAACCUGGTAA
PB235_atp6                        TTGGCAATCCTTGGTAGAGCTTCTTTATGATTTCGTGCUGAACCUGGTAA
BPM24_atp6                        TTGGCAATCCTTGGTAGAGCTTCTTTATGATTTCGTGCUGAACCUGGTAA
RRIC110_atp6                      TTGGCAATCCTTGGTAGAGCTTCTTTATGATTTCGTGCUGAACCUGGTAA
RRIT251_atp6                      TTGGCAATCCTTGGTAGAGCTTCTTTATGATTTCGTGCUGAACCUGGTAA
RRIM600_atp6                      TTGGCAATCCTTGGTAGAGCTTCTTTATGATTTCGTGCNGAACCNGGTAA
Millettia pinnata                 TTGGCAATCCTTGGTAGAGCTTATTTATGATTTCGTGCUGAACCTGGTAA
Oryza sativa Japonica             ATGGCAATCCTTGGTAGAGCTTATTTATGATTTCGTGCTGAACCTGGTAA
                                   ******  * * ** **  *  ********** ***   ***  *** *

Megaceros aenigmaticus            ACGAACAAATAAGTGGTCCTTCTTTA---------GTGAAACAACGGTTT
Phaeoceros laevis                 ACGAACAAATAAGTGGTCCTTCTTTA---------GTGAAACAACGGTTC
Cycas taitungensis                ACGAACAAATAGGTGGTCTTTCCGGAA------ATGTTAAACAAAAGTTT
Beta macrocarpa                   ACGAACAAATAGGTGGTCTTTCCGGA------AATGTTAAACAAAAGTTT
Beta vulgaris maritima            ACGAACAAATAGGTGGTCTTTCCGGA------AATGTTAAACAAAAGTTT
Beta vulgaris vulgaris            ACGAACAAATAGGTGGTCTTTCCGGA------AATGTTAAACAAAAGTTT
Citrullus lanatus                 ACGAACAAATAGGTGGTCTTTCCGGA------AATGTTAAACAAAAGTTT
Cucurbita pepo                    ACGAACAAATAGGCGGTCTTTCCGGATCCGGAAATGTGAAACAAAAGTTT
Arabidopsis thaliana              AGGAACAAATAGGTGGTCTTTCCGGG------AATGTGAAACAAATGTTT
Brassica napus                    AGGAACAAATAGGTGGTCTTTCCGGA------AATGTGAAACAAATGTTT
Silene latifolia                  ACGAACAAATAGGGGGTCTTTCCGGA------AATGTTAAACAAAAGTTT
RRII105_atp6                      ACGAACAAATAGGTGGTCTTTCCGGA------AATGTTAAACAAAAGTTT
PB235_atp6                        ACGAACAAATAGGTGGTCTTTCCGGA------AATGTTAAACAAAAGTTT
BPM24_atp6                        ACGAACAAATAGGTGGTCTTTCCGGA------AATGTTAAACAAAAGTTT
RRIC110_atp6                      ACGAACAAATAGGTGGTCTTTCCGGA------AATGTTAAACAAAAGTTT
RRIT251_atp6                      ACGAACAAATAGGTGGTCTTTCCGGA------AATGTTAAACAAAAGTTT
RRIM600_atp6                      ACGAACAAATAGGTGGTCTTTCCGGA------AATGTTAAACAAAAGTTT
```

```
Millettia pinnata                 ACGAACAAATAGGTGGTC---------------ATGTTAAACAAAAGTTT
Oryza sativa Japonica             ACGAACAAATAGGTGGA---------------AATGTTAAACAAAAGTTT
                                  * ********* * **                   ** ******  ***

Megaceros aenigmaticus            TTTTTGTCGATATTTGTCACTTTCACTTGTCTACCATTTCGTAATTTTAT
Phaeoceros laevis                 TTTTCGTTGATATTTGTCACTTTCACTTGTCTATTATTTCGTAATTTTAT
Cycas taitungensis                CCCCCTCGCATCTCGGTCACTTTTACTTCTTCGTTATTTCGTAATCCCCA
Beta macrocarpa                   TTCCCTTGCATCTTGGTCACTTTTACTTTTTTGTTATTTUGTAATCTCCA
Beta vulgaris maritima            TTCCCTTGCATCTTGGTCACTTTTACTTTTTTGTTATTTUGTAATCTCCA
Beta vulgaris vulgaris            TTCCCTTGCATCTTGGTCACTTTTACTTTTTTGTTATTTUGTAATCTCCA
Citrullus lanatus                 TTCCCTUGCATCTUGGTCACTTTTACTTTTTUGTTATTTUGTAATCUUCA
Cucurbita pepo                    TTCCCTUGCATCTUGGTCACTTTGACTTTTTUGTTATTTUGTAATCUUCA
Arabidopsis thaliana              TTCCCTTGCATCTTGGTCACTTTTCTTTTTTTGTTATTTTGTAATCTTCA
Brassica napus                    TTCCCTTGCATCTTGGTCACTTTTCTTTTTTTGTTATTTTGTAATCTTCA
Silene latifolia                  TTCCCTTGCATCTTGGTCACTTTTACTTTTTTGTTATTTUGTAATCTCCA
RRII105_atp6                      TTTCCTCGCATCTCGGTCACTTTGACTTTTTCGTTATTTCGTAATCCCCA
PB235_atp6                        TTTCCTCGCATCTCGGTCACTTTGACTTTTTCGTTATTTCGTAATCCCCA
BPM24_atp6                        TTTCCTCGCATCTCGGTCACTTTGACTTTTTCGTTATTTCGTAATCCCCA
RRIC110_atp6                      TTTCCTCGCATCTCGGTCACTTTGACTTTTTCGTTATTTCGTAATCCCCA
RRIT251_atp6                      TTTCCTCGCATCTCGGTCACTTTGACTTTTTCGTTATTTCGTAATCCCCA
RRIM600_atp6                      TTTCCTCGCATCTCGGTCACTTTGACTTTTTCGTTATTTCGTAATCCCCA
Millettia pinnata                 TTCCCTTGCATCTUGGTCACTTTTACTTTTTUGTTATTTTGTAATCUUCA
Oryza sativa Japonica             TTCCCTUGCATCTUGGTCACTTTTACTTTTTCUTTATTTUGTAATCUCCA
                                           ** *  ********   ** *      **** *****

Megaceros aenigmaticus            CGGTATGATACCTTATAGTTTTACAGTAACAAGTCATTTTTCATTTACCT
Phaeoceros laevis                 CGGTATGATATTTTATAGTTTTACAGTAATAAGTTATTTTTTATTTACCT
Cycas taitungensis                GGGTATGATACCATATAGCTCCACAGTTACAAGTCATTTCATCATTACTT
Beta macrocarpa                   GGGTATGATACCCTATAGCTTTACAGTTACAAGTCATTTTCTCATTACTT
Beta vulgaris maritima            GGGTATGATACCCTATAGCTTTACAGTTACAAGTCATTTTCTCATTACTT
Beta vulgaris vulgaris            GGGTATGATACCCTATAGCTTTACAGTTACAAGTCATTTTCTCATTACTT
Citrullus lanatus                 GGGTATGATACCTTATAGCTTCACAGTTACAAGTCATTTTCTCATTACTT
Cucurbita pepo                    GGGTATGATACCTTATAGCTTCACAGTTACAAGTCATTTTCTCATTACTT
Arabidopsis thaliana              GGGTATGATACCTTATAGCTTCACAGTGACAAGTCATTTTCTCATTACTT
Brassica napus                    GGGTATGATACCTTATAGCTTCACAGTGACAAGTCATTTTCTCATTACTT
Silene latifolia                  GGGTATGATACCTTATAGCTTTACAGTTACAAGTCATTTTCTCATTACTT
RRII105_atp6                      GGGTATGATACCTTATAGCTTCACAGTTACAAGTCATTTTCTCATTACTT
PB235_atp6                        GGGTATGATACCTTATAGCTTCACAGTTACAAGTCATTTTCTCATTACTT
BPM24_atp6                        GGGTATGATACCTTATAGCTTCACAGTTACAAGTCATTTTCTCATTACTT
RRIC110_atp6                      GGGTATGATACCTTATAGCTTCACAGTTACAAGTCATTTTCTCATTACTT
RRIT251_atp6                      GGGTATGATACCTTATAGCTTCACAGTTACAAGTCATTTTCTCATTACTT
RRIM600_atp6                      GGGTATGATACCTTATAGCTTCACAGTTACAAGTCATTTTCTCATTACTT
Millettia pinnata                 GGGTATGATACCTTATAGCTTCACAGTTACAAGTCATTTTCTCATTACTT
Oryza sativa Japonica             GGGTATGATACCCTTTAGCTTCACAGTGACAAGTCATTTTCTCATTACTT
                                   *********   * *** *  ***** * **** ****     **** *

Megaceros aenigmaticus            TGGGTCTTCCATTCTCTCCTTCTTCTGGAACGACTTTACCTGGATTTCAA
Phaeoceros laevis                 TGGGTCTTCTATTCTCTCCTTCTCCTGGAATGACTTTACCTGGATTCAAA
Cycas taitungensis                TGGGTCCCCCATTTCCGATTTCTATTGGAATTACTATAGTGGGATCTCAA
Beta macrocarpa                   TGGGTCTTTCATTTTCCATTTTTATTGGCATTACTATAGTGGGATTTCAA
Beta vulgaris maritima            TGGGTCTTTCATTTTCCATTTTTATTGGCATTACTATAGTGGGATTTCAA
Beta vulgaris vulgaris            TGGGTCTTTCATTTTCCATTTTTATTGGCATTACTATAGTGGGATTTCAA
Citrullus lanatus                 TGGGTCTCTCATTTTCTCTTTTTATTGGTATTACTATAGTGGGATTTCAA
Cucurbita pepo                    TGGGTCTCTCATTTTCTCTTTTTATTGGTATTACTATAGTGGGATTTCAA
Arabidopsis thaliana              TGGCTCTCTCATTTTCGATTTTTATTGGCATTACTATAGTGGGATTTCAA
Brassica napus                    TGGCTCTCTCATTTTCTATTTTTATTGGCATTACTATAGTGGGATTTCAA
Silene latifolia                  TGGGCCTTTCATTTTCAATTTTTATTGGCATTACTATAGTGGGATTTCAA
RRII105_atp6                      TGGGTCTCTCATTTTCTCTTTTTATTGGCATTACTATAGTGGGATTTCAA
PB235_atp6                        TGGGTCTCTCATTTTCTCTTTTTATTGGCATTACTATAGTGGGATTTCAA
BPM24_atp6                        TGGGTCTCTCATTTTCTCTTTTTATTGGCATTACTATAGTGGGATTTCAA
RRIC110_atp6                      TGGGTCTCTCATTTTCTCTTTTTATTGGCATTACTATAGTGGGATTTCAA
RRIT251_atp6                      TGGGTCTCTCATTTTCTCTTTTTATTGGCATTACTATAGTGGGATTTCAA
```

```
RRIM600_atp6                   TGGGTCTCTCATTTTCTCTTTTTATTGGCATTACTATAGTGGGATTTCAA
Millettia pinnata              TGGGTCTCTCATTTTCTATTTTTATTGGCATTACTATAGTGGGATTTCAA
Oryza sativa Japonica          TGGCTCTTTCATTTTCCATTTTTATAGGCATTACGATCGTTGGATTTCAA
                               ***  *    ***  *   ** *   ** *  **  *  ****     **

Megaceros aenigmaticus         ATACATGGGCTTCATTTTTTCAGTTTCTTCTTACCTCGAGGAGTAGCATT
Phaeoceros laevis              ATACATGGGCTTCATTTTTTCAGTTTCTTTTTACCTCGAGGAGTAGCATT
Cycas taitungensis             AGAAATGGGCTTCATTTCTCCAGCTCCTCATCACCCGCAGGAGTACCACT
Beta macrocarpa                AGAAATGGGCTTCATTTTTTAAGCTTCTUATTACCTGCAGGAGTCCCGCT
Beta vulgaris maritima         AGAAATGGGCTTCATTTTTTAAGCTTCTUATTACCTGCAGGAGTCCCGCT
Beta vulgaris vulgaris         AGAAATGGGCTTCATTTTTTAAGCTTCTUATTACCTGCAGGAGTCCCGCT
Citrullus lanatus              AGAAATGGGCTTCATTTTTTAAGTTTCTUATTACCTGCAGGAGTCCCCCT
Cucurbita pepo                 AGAAATGGGCTTCATTTTTTAAGTTTCTUATTACCCGCTGGAGTCCCACT
Arabidopsis thaliana           AGACATGGGCTTCATTTTTTCAGCTTTTTATTACCCGCAGGAGTCCCACT
Brassica napus                 AGACATGGGCTTCATTTTTTCAGCTTTTTATTACCCGCAGGAGTCCCACT
Silene latifolia               AGAAATGGGCTTCATTTTTTAAGCTTCTUATTACCTGCAGGAGTCCCACT
RRII105_atp6                   AAAAATGGGCTTCATTTTTTAAGCTTCTUATTACCCGCAGGAGTCCCGCT
PB235_atp6                     AAAAATGGGCTTCATTTTTTAAGCTTCTUATTACCCGCAGGAGTCCCGCT
BPM24_atp6                     AAAAATGGGCTTCATTTTTTAAGCTTCTUATTACCCGCAGGAGTCCCGCT
RRIC110_atp6                   AAAAATGGGCTTCATTTTTTAAGCTTCTUATTACCCGCAGGAGTCCCGCT
RRIT251_atp6                   AAAAATGGGCTTCATTTTTTAAGCTTCTUATTACCCGCAGGAGTCCCGCT
RRIM600_atp6                   AAAAATGGGCTTCATTTTTTAAGCTTCTUATTACCCGCAGGAGTCCCGCT
Millettia pinnata              AGAAATGGGCTTCATTTTTTAAGCTTCTUATTACCCGCAGGAGTCCCACT
Oryza sativa Japonica          AGACATGGGCTTCATTTTTTTAGCTTCTTATTACCAGCGGGAGTCCCACT
                               * * ************* *  ** *  *  * ***    *****  *  *

Megaceros aenigmaticus         GCCGTTAGCTCCTTTCTTAGTAATGCTCGAGCCGTTTCCTCATAGTCTTC
Phaeoceros laevis              GCCGTTAGCTCCTTTCTTAGTAATGCTCGAGCCGTTTCCTCATAGTCTTC
Cycas taitungensis             GCCGTTAGCACCTTTCTCAGTACTCCTTGAGCTAATCCCTCATTGTTCTC
Beta macrocarpa                GCCGTTAGCACCTTTTTTAGTACTCCTTGAGCTAATCUCTUATTGTTTTC
Beta vulgaris maritima         GCCGTTAGCACCTTTTTTAGTACTCCTTGAGCTAATCUCTUATTGTTTTC
Beta vulgaris vulgaris         GCCGTTAGCACCTTTTTTAGTACTCCTTGAGCTAATCUCTUATTGTTTTC
Citrullus lanatus              GCCATTAGCACCTTTTTTAGTACTCCTTGAGCTAATCUCTUATTGTTTTC
Cucurbita pepo                 GCCATTAGCACCTTTTTTAGTACTCCTTGAGCTAATCUCTUATTGTTTTC
Arabidopsis thaliana           GCCGTTAGCACCTTTTTTAGTACTCCTTGAGCTAATTTCTTATTGTTTTC
Brassica napus                 GCCGTTAGCACCTTTTTTAGTACTCCTTGAGCTAATTTCTTATTGTTTTC
Silene latifolia               GCCGTTAGCACCTTTTTTAGTACTCCTTGAGCTAATCUCTUATTGTTTTC
RRII105_atp6                   GCCGTTAGCACCCTTTTTAGTACTCCTTGAGCTAATCUCTUATTGTTTTC
PB235_atp6                     GCCGTTAGCACCCTTTTTAGTACTCCTTGAGCTAATCUCTUATTGTTTTC
BPM24_atp6                     GCCGTTAGCACCCTTTTTAGTACTCCTTGAGCTAATCUCTUATTGTTTTC
RRIC110_atp6                   GCCGTTAGCACCCTTTTTAGTACTCCTTGAGCTAATCUCTUATTGTTTTC
RRIT251_atp6                   GCCGTTAGCACCCTTTTTAGTACTCCTTGAGCTAATCUCTNATTGTTTTC
RRIM600_atp6                   GCCGTTAGCACCCTTTTTAGTACTCCTTGAGCTAATCNCTNATTGTTTTC
Millettia pinnata              GCCGTTAGCACCTTTTTTAGTACTCCTTGAGCTAATCUCTUATTGTTTTC
Oryza sativa Japonica          GCCATTAGCACCTTTTTTAGTACTCCTTGAGCTAATCTCTUATTGTTTTC
                               *** ***** ** ** * **** * ** ****   *  ** ** **  **

Megaceros aenigmaticus         GCGCATTAAGCTTAGGTATACGTTTATCCGCCAATATGATGGCTGGTCAT
Phaeoceros laevis              GCGTATTAAGCTTAGGAATATGTTTATTCGCCAATATGATGGCTGGTCAT
Cycas taitungensis             GCGCATCAAGCTCAGGAATACGTTTATTTGCTAATATGATGGCTGGTCAT
Beta macrocarpa                GCGCATTAAGCTUAGGAATACGTTTATTTGCTAATATGATGGCCGGTCAT
Beta vulgaris maritima         GCGCATTAAGCTUAGGAATACGTTTATTTGCTAATATGATGGCCGGTCAT
Beta vulgaris vulgaris         GCGCATTAAGCTUAGGAATACGTTTATTTGCTAATATGATGGCCGGTCAT
Citrullus lanatus              GCGCATTAAGCTUAGGAATACGTTTATTTGCTAATATGATGGCCGGTCAT
Cucurbita pepo                 GCGCATTAAGCTUAGGAATACGGTTATTTGCTAATATGATGGCCGGTCAT
Arabidopsis thaliana           GCGCATTAAGCTTAGGAATACGTTTATTTGCTAATATGATGGCCGGTCAT
Brassica napus                 GCGCATTAAGCTTAGGAATACGTTTATTTGCTAATATGATGGCCGGTCAT
Silene latifolia               GCGCATTAAGCTTAGGGATACGGTTATTTGCTAATATGATGGCCGGTCAT
RRII105_atp6                   GCGCATTAAGCTUAGGAATACGTTTATTTGCTAATATGATGGCCGGTCAT
PB235_atp6                     GCGCATTAAGCTUAGGAATACGTTTATTTGCTAATATGATGGCCGGTCAT
BPM24_atp6                     GCGCATTAAGCTUAGGAATACGTTTATTTGCTAATATGATGGCCGGTCAT
RRIC110_atp6                   GCGCATTAAGCTUAGGAATACGTTTATTTGCTAATATGATGGCCGGTCAT
```

```
RRIT251_atp6                  GCGCATTAAGCTUAGGAATACGTTTATTTGCTAATATGATGGCCGGTCAT
RRIM600_atp6                  GCGCATTAAGCTNAGGAATACGTTTATTTGCTAATATGATGGCCGGTCAT
Millettia pinnata             GCGCATTAAGCTUAGGAATACGTTTATTTGCTAATATGATGGCCGGTCAT
Oryza sativa Japonica         GTGCATTAAGCTUAGGAATACGTTTATTTGCTAATATGATGGCCGGTCAT
                              * * ** ***** *** *** * ****  ** *********** ******

Megaceros aenigmaticus        AGTTCAGTGAAGATTTCAAGTGGGTTTGCTCGGACTATGCTATCTATGGG
Phaeoceros laevis             AGTTCAGTGAAGATTTCAAGTGGGTTTGCTCGGACTATGCTATCTATGGG
Cycas taitungensis            AGTTCAGTAAAGATCTCAAGTGGGTCCGCTCGGACTATGCTATGTATGAA
Beta macrocarpa               AGTTUAGTAAAGATTTTAAGTGGGTTCGCTTGGACTATGCTATGTATGAA
Beta vulgaris maritima        AGTTUAGTAAAGATTTTAAGTGGGTTCGCTTGGACTATGCTATGTATGAA
Beta vulgaris vulgaris        AGTTUAGTAAAGATTTTAAGTGGGTTCGCTTGGACTATGCTATGTATGAA
Citrullus lanatus             AGTTUAGTAAAGATTTTAAGTGGGTUCGCTTGGACTATGCTATGTATGAA
Cucurbita pepo                AGTTUAGTAAAGATTTTAAGTGGGTUCGCTTGGACTATGCTATGTATGAA
Arabidopsis thaliana          AGTTTAGTAAAGATTTTAAGTGGGTTCGCTTGGACTATGCTATGTATGAA
Brassica napus                AGTTTAGTAAAGATTTTAAGTGGGTTCGCTTGGACTATGCTATGTATGAA
Silene latifolia              AGTTUAGTAAAGATTTTAAGTGGGTTCGCTTGGACTATGCTATGTATGAA
RRII105_atp6                  AGTTUAGTAAAGATTTTAAGTGGGTTCGCTTGGACTATGCTATGTATGAA
PB235_atp6                    AGTTUAGTAAAGATTTTAAGTGGGTTCGCTTGGACTATGCTATGTATGAA
BPM24_atp6                    AGTTUAGTAAAGATTTTAAGTGGGTTCGCTTGGACTATGCTATGTATGAA
RRIC110_atp6                  AGTTUAGTAAAGATTTTAAGTGGGTTCGCTTGGACTATGCTATGTATGAA
RRIT251_atp6                  AGTTUAGTAAAGATTTTAAGTGGGTTCGCTTGGACTATGCTATGTATGAA
RRIM600_atp6                  AGTTNAGTAAAGATTTTAAGTGGGTTCGCTTGGACTATGCTATGTATGAA
Millettia pinnata             AGTTUAGTAAAGATTTTAAGTGGGTCCGCTTGGACTATGCTATGTATGAA
Oryza sativa Japonica         AGTTUAGTAAAGATTTTAAGTGGGTTCGCTTGGACTATGCTATTTCTGAA
                              **** *** ***** * ********  *** ************ * **

Megaceros aenigmaticus        GGGTATTATGTATTT---AGCACATCTTGCCCCTCTTTTGGTAGTATTCG
Phaeoceros laevis             GGGTATTATGTATTT---AGCACATCTTGCTTTTTTTCTGGTAGTATTCG
Cycas taitungensis            TAATATAATGTATTTCATAGGAGATCTTGGTCCTTCATCAATAGTTTTTG
Beta macrocarpa               TGATCTTTTATATTTCATAGGAGATCTTGGTCCTTTATTTATAGTTCTTG
Beta vulgaris maritima        TGATCTTTTATATTTCATAGGAGATCTTGGTCCTTTATTTATAGTTCTTG
Beta vulgaris vulgaris        TGATCTTTTATATTTCATAGGAGATCTTGGTCCTTTATTTATAGTTCTTG
Citrullus lanatus             TAATCTTTTCTATTTCATAGGAGATCUTGGTCCTTTATTTATAGTTCTTG
Cucurbita pepo                TAATCTTTTCTATTTCATAGGGGATCUTGGTCCTTTATTTATAGTTCTTG
Arabidopsis thaliana          TGATATTTTCTATTTTATAGGGGCTCTTGGTCCTTTATTTATAGTTCTTG
Brassica napus                TGAGATTTTCTATTTTATAGGGGCTCTTGGTCCTTTATTTATAGTTCTTG
Silene latifolia              TGATCTTTTATATTTCATAGGAGATCTTGGTCCTTTATTTATAGTTCTTG
RRII105_atp6                  TGATCTTTTCTATTTCATAGGGGATCUTGGTCCTTTATTTATAGTTCTTG
PB235_atp6                    TGATCTTTTCTATTTCATAGGGGATCUTGGTCCTTTATTTATAGTTCTTG
BPM24_atp6                    TGATCTTTTCTATTTCATAGGGGATCUTGGTCCTTTATTTATAGTTCTTG
RRIC110_atp6                  TGATCTTTTCTATTTCATAGGGGATCNTGGTCCTTTATTTATAGTTCTTG
RRIT251_atp6                  TGATCTTTTCTATTTCATAGGGGATCUTGGTCCTTTATTTATAGTTCTTG
RRIM600_atp6                  TGATCTTTTCTATTTCATAGGGGATCNTGGTCCTTTATTTATAGTTCTTG
Millettia pinnata             TGATCTTTTCTATTTCATAGGAGATCTTGGTCCTTTATTTATAGTTCTTG
Oryza sativa Japonica         TAATATTTTCTATTTCATAGGAGATCTTGGTCCCTTATTTATAGTTCTAG
                                   *  * *****   **    ** **            ****  * *

Megaceros aenigmaticus        CATCAACTGGTTTAGAATTAGGTGTCGGTGTATCACAAGCTCATGTTCCT
Phaeoceros laevis             TATCAACTGGTTCAGAATTAGGTGTCGGTATATCACAAGTTTATGTTTCT
Cycas taitungensis            CACCGACCGGTTCGGAATTAGGTGTAGCTATATCACAAGCTCATGTTTCT
Beta macrocarpa               CATTAACCGGTCTTGAATTAGGTGTAGCTATATTACAAGCTUATGTTTTT
Beta vulgaris maritima        CATTAACCGGTCTTGAATTAGGTGTAGCTATATTACAAGCTUATGTTTTT
Beta vulgaris vulgaris        CATTAACCGGTCTTGAATTAGGTGTAGCTATATTACAAGCTUATGTTTTT
Citrullus lanatus             CATTAACCGGTTTGGAATTAGGTGTAGCTATATUACAAGCTUATGTTTUT
Cucurbita pepo                CATTAACCGGTTTGGAATTAGGTGTAGCTATATUACAAGCTUATGTTTUT
Arabidopsis thaliana          CATTAACCGGTCTGGAATTAGGTGTAGCTATATTACAAGCTTATGTTTTT
Brassica napus                CATTAACCGGTCTGGAATTAGGTGTAGCTATATTACAAGCTTATGTTTTT
Silene latifolia              CATTAACCGGTCTTGAATTAGGTGTAGCTATATTACAAGCTUATGTTTTT
RRII105_atp6                  CATTAACCGGTCTGGAATTAGGTGTAGCTATATTACAAGCTUATGTTTUT
PB235_atp6                    CATTAACCGGTCTGGAATTAGGTGTAGCTATATTACAAGCTUATGTTTUT
BPM24_atp6                    CATTAACCGGTCTGGAATTAGGTGTAGCTATATTACAAGCTUATGTTTUT
```

```
RRIC110_atp6                  CATTAACCGGTCTGGAATTAGGTGTAGCTATATTACAAGCTUATGTTTUT
RRIT251_atp6                  CATTAACCGGTCTGGAATTAGGTGTAGCTATATTACAAGCTNATGTTTNT
RRIM600_atp6                  CATTAACCGGTCTGGAATTAGGTGTAGCTATATTACAAGCTNATGTTTNT
Millettia pinnata             CATTAACCGGTCTGGAATTAGGTGTAGCTATATUACAAGCTUATGTTTUT
Oryza sativa Japonica         CATTAACCGGTCTGGAATTAGGTGTAGCTATATTACAAGCTUATGTTTUT
                               *   ** ***   *********** * * *** ***** * *****  *

Megaceros aenigmaticus        ACTACTCCAAATCGCACTTACCTCAATGATGCTATAAACCTTCATTAA--
Phaeoceros laevis             ACTACTCCAAATTGCACTTACTTCAATGATGCTATAAACCTTCATTAA--
Cycas taitungensis            ACAATCTCAATTTGTACTTACTCGAATGATGCTACAAATCCTCATUGAAG
Beta macrocarpa               ACGATCTTAATCTGTATTTACTTGAATGATGCTAUAAATCTCCATUAAAA
Beta vulgaris maritima        ACGATCTTAATCTGTATTTACTTGAATGATGCTAUAAATCTCCATUAAAA
Beta vulgaris vulgaris        ACGATCTTAATCTGTATTTACTTGAATGATGCTAUAAATCTCCATUAAAA
Citrullus lanatus             ACGATCTUAATCTGTATTTACTTGAATGATGCTAUAAATCTCCATUAAAA
Cucurbita pepo                ACGATCTCAATCTGTATTTACTTGAATGATGCTACAAATCTCCATCAAAA
Arabidopsis thaliana          ACGATCTTAATCTGTATTTACTTGAATGATGCTATAAATCTCCATTAAAG
Brassica napus                ACGATCTTAATCTGTATTTACTTGAATGATGCTATAAATCTCCATTAAAG
Silene latifolia              ACGATCTTAATCTGTATTTACTTGAATGATGCTAUAAATCTCCATUAAAA
RRII105_atp6                  ACGATCTTAATCTGTATTTACTTGAATGATGCTACAAATCTCCATCAAAG
PB235_atp6                    ACGATCTTAATCTGTATTTACTTGAATGATGCTACAAATCTCCATCAAAG
BPM24_atp6                    ACGATCTTAATCTGTATTTACTTGAATGATGCTACAAATCTCCATCAAAG
RRIC110_atp6                  ACGATCTTAATCTGTATTTACTTGAATGATGCTACAAATCTCCATCAAAG
RRIT251_atp6                  ACGATCTTAATCTGTATTTACTTGAATGATGCTACAAATCTCCATCAAAG
RRIM600_atp6                  ACGATCTTAATCTGTATTTACTTGAATGATGCTACAAATCTCCATCAAAG
Millettia pinnata             ACGATCTUAATATGTATTTACTTGAATGATGCTATAAATCTCCATUAAAC
Oryza sativa Japonica         ACGATCTUAATTTGTATTTACTTGAATGATGCTATAAATCTCCATUAAAA
                              ** *    **   * * ****   ********** *** *  ***  *

Megaceros aenigmaticus        --------------------------------------------------
Phaeoceros laevis             --------------------------------------------------
Cycas taitungensis            GATCAATTTTTTTT----------ATAATTGAACAAAAGCTTCGAGCAGG
Beta macrocarpa               TTCTTTTTTCTTT-----------TTATTAGAATTTTTATAATTGAACAA
Beta vulgaris maritima        TTCTTTTTTCTTT-----------TTATTAGAATTTTTATAATTGAACAA
Beta vulgaris vulgaris        TTCTTTTTTCTTT-----------TTATTAGAATTTTTATAATTGAACAA
Citrullus lanatus             TGGTTATTTTTTT-----------ATAATTGAACAAAAGCGA--GGCTCG
Cucurbita pepo                TAGTTATTTATTT-----------ATAATTGAACAAAAGCGA--GGAGAG
Arabidopsis thaliana          TTCTTCTTTCTTTTATTTATATTTATAATTGAACAAAAGCGA--GGGATG
Brassica napus                TTCTTCTTTCTTTTATTTAGATTTATAATTGAACAAAAGCGA--GGGATG
Silene latifolia              TGGTTCTTTTTTTAGAGTAAAGGGA-GGTTCTTCAGTAGCCA--TCGCAG
RRII105_atp6                  TGGTTCTTTTTTT-----------ATAATTGAACAAAAGCGA--GGAGCG
PB235_atp6                    TGGTTCTTTTTTT-----------ATAATTGAACAAAAGCGA--GGAGCG
BPM24_atp6                    TGGTTCTTTTTTT-----------ATAATTGAACAAAAGCGA--GGAGCG
RRIC110_atp6                  TGGTTCTTTTTTT-----------ATAATTGAACAAAAGCGA--GGAGCG
RRIT251_atp6                  TGGTTCTTTTTTT-----------ATAATTGAACAAAAGCGA--GGAGCG
RRIM600_atp6                  TGGTTCTTTTTTT-----------ATAATTGAACAAAAGCGA--GGAGCG
Millettia pinnata             TTTTTTGTTATTT-----------ATAATTGAACAAAAGCGA--GGAGCG
Oryza sativa Japonica         TGAGTAATTTCATAATTGAATAAAAACGAGGAGCCGAAGATTTTAGGGGG

Megaceros aenigmaticus        --------------------------------------------------
Phaeoceros laevis             --------------------------------------------------
Cycas taitungensis            GTGCGCCTGCTCTTTTTCTCCCCCGAAGAACAAGACGGGATAGATTCAAT
Beta macrocarpa               AAGCGAGGTATTTATGCTTAAGTCATCGCTTCAAATCCAATAAGGGATCA
Beta vulgaris maritima        AAGCGAGGTATTTATGCTTAAGTCATCGCTTCAAATCCAATAAGGGATCA
Beta vulgaris vulgaris        AAGCGAGGTATTTATGCTTAAGTCATCGCTTCAAATCCAATAAGGGATCA
Citrullus lanatus             ATGTAATTGAGCTCGAACGAGGATCCCTTTGAAGAAGGAATTGAGAGTCA
Cucurbita pepo                AGAGAAGCT-GCGAGACCGTAAAG----TAAAGGACAGAAGAGAAGATGC
Arabidopsis thaliana          GATGTCTGAGCGGTTGAAAGAGTCGGTCTTGAAAACCGAAGTATTTCTAG
Brassica napus                AGAGT---AGTGTTATTTAGAG-CAGTTACACAGCCC----CTCTCCTTG
Silene latifolia              GGTTCAT-TGCTTTTTTTTTTTTCAATAAAAAGGAGACTAGTCCCCGAAA
RRII105_atp6                  GAAGCCA-GTCGACGGAGAGGAGCGGATATGAAAACGAAGGCTAGATTTT
PB235_atp6                    GAAGCCA-GTCGACGGAGAGGAGCGGATATGAAAACGAAGGCTAGATTTT
```

```
BPM24_atp6                       GAAGCCA-GTCGACGGAGAGGAGCGGATATGAAAACGAAGGCTAGATTTT
RRIC110_atp6                     GAAGCCA-GTCGACGGAGAGGAGCGGATATGAAAACGAAGGCTAGATTTT
RRIT251_atp6                     GAAGCCA-GTCGACGGAGAGGAGCGGATATGAAAACGAAGGCTAGATTTT
RRIM600_atp6                     GAAGCCA-GTCGACGGAGAGGAGCGGATATGAAAACGAAGGCTAGATTTT
Millettia pinnata                AAGT-----GAGCATATAAGAAGAAGTGCTTAAAACG-AAGTAAATGGGC
Oryza sativa Japonica            CGGGAGAAAAGGTCAAGTCTAAGCGACATATGGCACGAAAAGGAAATCCA


Megaceros aenigmaticus           --------------------------------------------------
Phaeoceros laevis                --------------------------------------------------
Cycas taitungensis               GGTAGATCGATCGGCCAATAAACC--------------------------
Beta macrocarpa                  GACCC--CTCCTCGTGTTCAAACTAGTC----------------------
Beta vulgaris maritima           GACCC--CTCCTCGTGTTCAAACTAGTC----------------------
Beta vulgaris vulgaris           GACCC--CTCCTCGTGTTCAAACTAGTC----------------------
Citrullus lanatus                TATTCAATCGCCCGGGACTTTTTTGAAG----------------------
Cucurbita pepo                   AAACAAAACACCTATTTACTGAGTGAAAGAAT------------------
Arabidopsis thaliana             GAATACCGGGGGTTCGAATCCCTCTCCATCCGCGAAGTCATAAGTTCTCT
Brassica napus                   CAGTCGAGTGACTTCG--CCCCTGAATGT---------------------
Silene latifolia                 ATGCTCCGCGCCTAGGCGCCTAGGTTCGTTTGT-----------------
RRII105_atp6                     CTTTTGTCTCTTCCCCGACCAATTCATACCTTTTAGTGAGTAGTCGTCCA
PB235_atp6                       CTTTTGTCTCTTCCCCGACCAATTCATACCTTTTAGTGAGTAGTCGTCCA
BPM24_atp6                       CTTTTGTCTCTTCCCCGACCAATTCATACCTTTTAGTGAGTAGTCGTCCA
RRIC110_atp6                     CTTTTGTCTCTTCCCCGACCAATTCATACCTTTTAGTGAGTAGTCGTCCA
RRIT251_atp6                     CTTTTGTCTCTTCCCCGACCAATTCATACCTTTTAGTGAGTAGTCGTCCA
RRIM600_atp6                     CTTTTGTCTCTTCCCCGACCAATTCATACCTTTTAGTGAGTAGTCGTCCA
Millettia pinnata                GACCACCTTCAACATGAATGACTTT-------------------------
Oryza sativa Japonica            ATTTCGGTAATCCGAAGCCCACCTTACCTTAATCCATTTTCTTGAGGATC


Megaceros aenigmaticus           --------------------------------------------------
Phaeoceros laevis                --------------------------------------------------
Cycas taitungensis               --------------------------------------------------
Beta macrocarpa                  --------------------------------------------------
Beta vulgaris maritima           --------------------------------------------------
Beta vulgaris vulgaris           --------------------------------------------------
Citrullus lanatus                --------------------------------------------------
Cucurbita pepo                   --------------------------------------------------
Arabidopsis thaliana             CTTGCCGCCTGATAAGAACGAATCGGATCGACTCGACTGATATGATA---
Brassica napus                   --------------------------------------------------
Silene latifolia                 --------------------------------------------------
RRII105_atp6                     ATTCCGGCCGGTATTTGCTCCCTTTCTCCGGTCTTACTAATTTGTAAAGA
PB235_atp6                       ATTCCGGCCGGTATTTGCTCCCTTTCTCCGGTCTTACTAATTTGTAAAGA
BPM24_atp6                       ATTCCGGCCGGTATTTGCTCCCTTTCTCCGGTCTTACTAATTTGTAAAGA
RRIC110_atp6                     ATTCCGGCCGGTATTTGCTCCCTTTCTCCGGTCTTACTAATTTGTAAAGA
RRIT251_atp6                     ATTCCGGCCGGTATTTGCTCCCTTTCTCCGGTCTTACTAATTTGTAAAGA
RRIM600_atp6                     ATTCCGGCCGGTATTTGCTCCCTTTCTCCGGTCTTACTAATTTGTAAAGA
Millettia pinnata                --------------------------------------------------
Oryza sativa Japonica            --------------------------------------------------


Megaceros aenigmaticus           ----------------------------------
Phaeoceros laevis                ----------------------------------
Cycas taitungensis               ----------------------------------
Beta macrocarpa                  ----------------------------------
Beta vulgaris maritima           ----------------------------------
Beta vulgaris vulgaris           ----------------------------------
Citrullus lanatus                ----------------------------------
Cucurbita pepo                   ----------------------------------
Arabidopsis thaliana             ----------------------------------
Brassica napus                   ----------------------------------
Silene latifolia                 ----------------------------------
RRII105_atp6                     TTTGAGATTCCAGGGCAACATCCTATTATTTTAA
```

```
PB235_atp6                        TTTGAGATTCCAGGGCAACATCCTATTATTTTAA
BPM24_atp6                        TTTGAGATTCCAGGGCAACATCCTATTATTTTAA
RRIC110_atp6                      TTTGAGATTCCAGGGCAACATCCTATTATTTTAA
RRIT251_atp6                      TTTGAGATTCCAGGGCAACATCCTATTATTTTAA
RRIM600_atp6                      TTTGAGATTCCAGGGCAACATCCTATTATTTTAA
Millettia pinnata                 ----------------------------------
Oryza sativa Japonica             ----------------------------------


###################
```

atp8 multiple sequence alignment

```
Citrullus lanatus                 CTATAGTTCTTCACTACTCGTACAGGAAGGAGT---CTCTTTCTGTCTGG
Cucurbita pepo                    ----------TCACTACTCGTCCAGGAAGGAGT---CTCTTTCTGTCTGG
Beta macrocarpa                   ----------TTAACCACCATTTCTAGAGGCTT-GACGGAGTTCAGCTGT
Beta vulgaris maritima            ----------TTAACCACCATTTCTAGAGGCTT-GACGGAGTTCAGCTGT
Beta vulgaris vulgaris            ----------TTAACCACCATTTCTAGAGGCTT-GACGGAGTTCAGCTGT
Silene latifolia                  -----TTTTAACAACCACCAAAACTAGAGGCTT-G-CTTTTTTTCGTTAA
BPM24_atp8                        --------------------------------------------------
PB235_atp8                        --------------------------------------------------
Lotus japonicus                   --------CTTCAAGACTCCTACAT-GACATGAAGGGACTCTCTTTCTGT
Millettia pinnata                 ---------TCTAACTTTCGTTCTTCAAGACTCCGGGACTCTCTTTCTGT
RRIC110_atp8                      --------------------------------------------------
RRIM600_atp8                      --------------------------------------------------
RRIT251_atp8                      --------------------------------------------------
RRII105_atp8                      --------------------------------------------------
Cycas taitungensis                ----TCTCTCTATACCTTTACTACTCGTACAAGGGTCTATTTCTGGATAT


Citrullus lanatus                 ----AGGGAAT------CATTTTTTCTCAATCAAGA---ATGCCTCAACT
Cucurbita pepo                    CTGGAGGGAATAATAATCATTTTTTCTCAATCAAGA---ATGCCTCAACT
Beta macrocarpa                   CTGGAGGGAAG------AATTCAAAATCAAAACGTTCTTATGCCTCAACT
Beta vulgaris maritima            CTGGAGGGAAG------AATTCAAAATCAAAACGTTCTTATGCCTCAACT
Beta vulgaris vulgaris            CTGGAGGGAAG------AATTCAAAATCAAAACGTTCTTATGCCTCAACT
Silene latifolia                  CTGGAGGGAAG------AAGAAAAAAG-AAAAGATTCTTATGCCTCAACT
BPM24_atp8                        ---------------------------------------ATGCCTCAACT
PB235_atp8                        ---------------------------------------ATGCCTCAACT
Lotus japonicus                   CCGGAGGGAAT------CATTGTTTCTCAATCAATC---ATGCCTCAACT
Millettia pinnata                 ATGGAGGGAAT------CATTGTTTCTCAATCAATC---ATGCCTCAACT
RRIC110_atp8                      ---------------------------------------ATGCCTCAACT
RRIM600_atp8                      ---------------------------------------ATGCCTCAACT
RRIT251_atp8                      ---------------------------------------ATGCCTCAACT
RRII105_atp8                      ---------------------------------------ATGCCTCAACT
Cycas taitungensis                GGGAAAGGGAAG-----AAATAGAACTAAATAAATG---ATTCCTCAACC
                                                                         ** *******

Citrullus lanatus                 GGATAAATTGACTTATTTCACACAATTCTTCTGGTUATGCCTTTTCTTCT
Cucurbita pepo                    GGATAAATTGACTTATTTCACACAATTCTTCTGGTUATGCCTTTTCTTCT
Beta macrocarpa                   GGATCAATTTACTTATTTUACACAATTCTTCTGGTUATGCCTTTTCTTCT
Beta vulgaris maritima            GGATCAATTTACTTATTTUACACAATTCTTCTGGTUATGCCTTTTCTTCT
Beta vulgaris vulgaris            GGATCAATTTACTTATTTUACACAATTCTTCTGGTUATGCCTTTTCTTCT
Silene latifolia                  GGATAAATTTACTTATTTUACACAATTCTTCTGGTUATGCCTTTTTTTCT
BPM24_atp8                        GGATAAATTCACTTATTTTACACAATTCTTCTGGTCATGCCTTTTCCTCT
PB235_atp8                        GGATAAATTCACTTATTTTACACAATTCTTCTGGTCATGCCTTTTCCTCT
Lotus japonicus                   GGATAAATTCACTTATTTCACACAATTTTTCTGGTUATGCCTTTTCUTCT
Millettia pinnata                 GGATAAATTCACTTATTTCACACAATTTTTCTGGTUATGCCTTTTCUTCT
RRIC110_atp8                      GGATAAATTCACTTATTTTACACAATTCTTCTGGTCATGCCTTTTCCTCT
RRIM600_atp8                      GGATAAATTCACTTATTTTACACAATTCTTCTGGTCATGCCTTTTCCTCT
RRIT251_atp8                      GGATAAATTCACTTATTTTACACAATTCTTCTGGTCATGCCTTTTCCTCT
RRII105_atp8                      GGATAAATTCACTTATTTTACACAATTCTTCTGGTCATGCCTTTTCCTCT
Cycas taitungensis                GGATAAATTCACTTATTCCACACAATTCTTCCGGTTATGCCTGATCTTCT
                                  **** **** *******  ******** *** *** ******  *  ***
```

```
Citrullus lanatus               TGACTTTCTATATTUUCATATGCAATGATGGAGATGGAGTACTTGGGATC
Cucurbita pepo                  TTACTTTCTATATTUUCATATGCAATGATGGAGATGGAGTACTTGGGATC
Beta macrocarpa                 TGACTTTCTATATTCTAATATGCAATGATAGAGATGGAGTACTTGGGATC
Beta vulgaris maritima          TGACTTTCTATATTCTAATATGCAATGATAGAGATGGAGTACTTGGGATC
Beta vulgaris vulgaris          TGACTTTCTATATTCTAATATGCAATGATAGAGATGGAGTACTTGGGATC
Silene latifolia                TTACTTTCTATATTCTAATATGCAATGATAGAGATGGAGTACTTGGGATC
BPM24_atp8                      TTACTTTCTATATTCCCATATGCAATGATGGAGATGGAGTACTTGGGATC
PB235_atp8                      TTACTTTCTATATTCCCATATGCAATGATGGAGATGGAGTACTTGGGATC
Lotus japonicus                 TTACTTTCTATATTCUCATATGCAATGATGGAGATGGAGTACTTGGGATC
Millettia pinnata               TTACTTTTTATATTCUCATATGCAATGATGGAGATGGAGTACTTGGGATC
RRIC110_atp8                    TTACTTTCTATATTCCCATATGCAATGATGGAGATGGAGTACTTGGGATC
RRIM600_atp8                    TTACTTTCTATATTCCCATATGCAATGATGGAGATGGAGTACTTGGGATC
RRIT251_atp8                    TTACTTTCTATATTCCCATATGCAATGATGGAGATGGAGTACTTGGGATC
RRII105_atp8                    TTACTTTCTATATTCCCATATGCAATGATGGAGATGGAGTACTTGGGATC
Cycas taitungensis              TCACTTCTTATATTCCCCCATGCAAT------GATGGAGTACTTAGGATC
                                * ****  ******     *******      ************ *****

Citrullus lanatus               AGTAGAATTCTAAAACTACGGAACCAACTGGTTTCACACC------GGGG
Cucurbita pepo                  AGTAGAATTCTCAAACTCCGGAACCAACTGGTTTCACACC------GGGG
Beta macrocarpa                 AGCAGAATTCTAAAACTACGAAATCAACTGCTTTCACACC------GGGG
Beta vulgaris maritima          AGCAGAATTCTAAAACTACGAAATCAACTGCTTTCACACC------GGGG
Beta vulgaris vulgaris          AGCAGAATTCTAAAACTACGAAATCAACTGCTTTCACACC------GGGG
Silene latifolia                AGCAGAATTCTAAAACTACGAAATCAACTGCTTTCACACC------GGGG
BPM24_atp8                      AGCAGAATTCTAAAACTACGGAACCAACTGGTTTCACACC------GGGG
PB235_atp8                      AGCAGAATTCTAAAACTACGGAACCAACTGGTTTCACACC------GGGG
Lotus japonicus                 AGCAGAATTCTAAAACTACGGAACCAACTGGTTTCAAACC------GGGG
Millettia pinnata               AGCAGAATTCTAAAACTACGGAACCAACTGGTTTCAAACC------GGGG
RRIC110_atp8                    AGCAGAATTCTAAAACTACGGAACCAACTGGTTTCACACC------GGGG
RRIM600_atp8                    AGCAGAATTCTAAAACTACGGAACCAACTGGTTTCACACC------GGGG
RRIT251_atp8                    AGCAGAATTCTAAAACTACGGAACCAACTGGTTTCACACC------GGGG
RRII105_atp8                    AGCAGAATTCTAAAACTACGGAACCAACTGGTTTCACACC------GGGG
Cycas taitungensis              AGCAGAATTCCCAAACCACGGAACCAACTGGTTCCGCACCAACGAAGGGG
                                ** *******  ****  ** ** ****** ** *  ***      ****

Citrullus lanatus               GAACAACATACGGAGCAACGACCCCAACAGTTTGGAAGATATCTTGAGAA
Cucurbita pepo                  GAACAAGATACGGAGCAACGACCCCAACAGTTTGGAAGATATCTTGAGAA
Beta macrocarpa                 GAACAACATCCAAAGCAAGGACCCAAACAGTTTGCAAGATATCTTGAGAA
Beta vulgaris maritima          GAACAACATCCAAAGCAAGGACCCAAACAGTTTGCAAGATATCTTGAGAA
Beta vulgaris vulgaris          GAACAACATCCAAAGCAAGGACCCAAACAGTTTGCAAGATATCTTGAGAA
Silene latifolia                GAACGACATCCAAAGCAAGGACCCCAACAGTTTGGAAGATATCTTGAGAA
BPM24_atp8                      GAACAAGATCCGGAGCAAGGACCCCAACAGTTTGGAAGATATCTTGAGAA
PB235_atp8                      GAACAAGATCCGGAGCAAGGACCCCAACAGTTTGGAAGATATCTTGAGAA
Lotus japonicus                 GAACAAAATCCGGAGCAACGACCCCAACAGTTTGGAAGATATCTTTAGAA
Millettia pinnata               GAACAAAATCCGGAGCAACGACCCCAAGAGTTTGGAAGATATCTTTAGAA
RRIC110_atp8                    GAACAAGATCCGGAGCAAGGACCCCAACAGTTTGGAAGATATCTTGAGAA
RRIM600_atp8                    GAACAAGATCCGGAGCAAGGACCCCAACAGTTTGGAAGATATCTTGAGAA
RRIT251_atp8                    GAACAAGATCCGGAGCAAGGACCCCAACAGTTTGGAAGATATCTTGAGAA
RRII105_atp8                    GAACAAGATCCGGAGCAAGGACCCCAACAGTTTGGAAGATATCTTGAGAA
Cycas taitungensis              GAACAACATCCGGAGCAACGACCCCAACAGTTTGGAAGAGATTTCGAGAA
                                **** * ** *  ***** ***** ** ****** **** ** *  ****

Citrullus lanatus               AAGGTTTGAGCACCGGTGTATCCTATATGTACTCTAGTTTATTCGAAGTA
Cucurbita pepo                  AAGGTTTTAGCACCGGTGTATCCTATATGTACTCTAGTTTATTCGAAGTA
Beta macrocarpa                 AGGGTTTTAACACAGGTGTATCCTATATGTACTCTAGTTTATTCGAAGTA
Beta vulgaris maritima          AGGGTTTTAACACAGGTGTATCCTATATGTACTCTAGTTTATTCGAAGTA
Beta vulgaris vulgaris          AGGGTTTTAACACAGGTGTATCCTATATGTACTCTAGTTTATTCGAAGTA
Silene latifolia                AAGGTTTTAACACAGGTGTATCCTATATGTACTCTAGTTTATTCGAAGTA
BPM24_atp8                      AAGGTTTTAGTACCGGTGTATCCTATATGTACTCTAGTTTATTCGAAGTA
PB235_atp8                      AAGGTTTTAGTACCGGTGTATCCTATATGTACTCTAGTTTATTCGAAGTA
Lotus japonicus                 AAGGTTTTAGCACCGGTGTATCCTATATGTACTCTAGTTTATTCGAAGTA
Millettia pinnata               AAGGTTTTAGCACCGGTGTATCCTATATGTACTCTAGTTTATTCGAAGTA
RRIC110_atp8                    AAGGTTTTAGTACCGGTGTATCCTATATGTACTCTAGTTTATTCGAAGTA
```

```
RRIM600_atp8                      AAGGTTTTAGTACCGGTGTATCCTATATGTACTCTAGTTTATTCGAAGTA
RRIT251_atp8                      AAGGTTTTAGTACCGGTGTATCCTATATGTACTCTAGTTTATTCGAAGTA
RRII105_atp8                      AAGGTTTTAGTACCGGTGTATCCTATATGTACTCTAGTTTATTCGAAGTA
Cycas taitungensis                AAGGTTTTAGCACCGGTGTATCCCATATGTACTCTAGTTCATTCGAAGTA
                                  * ***** *  ** ********* *************** **********

Citrullus lanatus                 TCCCAATGGTGTAACGCCGTCGACTTATTGGGAAAAAGGAGGAAAA---T
Cucurbita pepo                    TCCCAATGGTGTAACGCCGTCGACTTATTGGGAAAAAGGAGGAAGAAGAT
Beta macrocarpa                   TCCCAATGGTGTAAGGCCGTCGACTTATTTGGAAAAAGGAAGAAAA---T
Beta vulgaris maritima            TCCCAATGGTGTAAGGCCGTCGACTTATTTGGAAAAAGGAAGAAAA---T
Beta vulgaris vulgaris            TCCCAATGGTGTAAGGCCGTCGACTTATTTGGAAAAAGGAAGAAAA---T
Silene latifolia                  TCCCAATGGTGTAAGGCCGTCGACTTATTTGGAAAAAGGAAGAAAA---T
BPM24_atp8                        TCCCAATGGTGTAAGGCTGTCGACTTATTGGGAAAAAGGAGGAAAA---T
PB235_atp8                        TCCCAATGGTGTAAGGCTGTCGACTTATTGGGAAAAAGGAGGAAAA---T
Lotus japonicus                   TCCCAATGGTGTAACGCCGTCGACTTATTGGGAAAAAGGAGGAAAA---T
Millettia pinnata                 TCCCAATGGTGTAACGCCGTCGACTTATTGGGAAAAAGGAGGATAA---T
RRIC110_atp8                      TCCCAATGGTGTAAGGCTGTCGACTTATTGGGAAAAAGGAGGAAAA---T
RRIM600_atp8                      TCCCAATGGTGTAAGGCTGTCGACTTATTGGGAAAAAGGAGGAAAA---T
RRIT251_atp8                      TCCCAATGGTGTAAGGCTGTCGACTTATTGGGAAAAAGGAGGAAAA---T
RRII105_atp8                      TCCCAATGGTGTAAGGCTGTCGACTTATTGGGAAAAAGGAGGAAAA---T
Cycas taitungensis                TCCCAATGGTGTAACGCCGTCGACTTATTGGGGAAAAGGAAGAGAA---T
                                  ************** ** *********** ** ******* **  *   *

Citrullus lanatus                 CACTTTGATCTCTTGTTTCGGAGAAATAAGTGGCTCACGAGGTCTCGAAA
Cucurbita pepo                    CACTTTTCTCTCTTGTTTCGGAGAAATAAGTGGCTCACGAGGTCTCGAAA
Beta macrocarpa                   CACTTTGATCTCTTGTTTCGGAGAAATAAGTGGCTCACGAGGAATGGAAA
Beta vulgaris maritima            CACTTTGATCTCTTGTTTCGGAGAAATAAGTGGCTCACGAGGAATGGAAA
Beta vulgaris vulgaris            CACTTTGATCTCTTGTTTCGGAGAAATAAGTGGCTCACGAGGAATGGAAA
Silene latifolia                  AACTTTGATCTCTTGTTTCGGAGAAATAATTGGCTCAGGAGGAATAGAAG
BPM24_atp8                        CACTTTGATCTCTTGTTTCGGAGAAATAAGTGGCTCGCGAGGAATGGAAA
PB235_atp8                        CACTTTGATCTCTTGTTTCGGAGAAATAAGTGGCTCGCGAGGAATGGAAA
Lotus japonicus                   AACTTTTCTCTCTTGTTTCGGAGAAATAAGTGGCTCACGAGGAATGGAAA
Millettia pinnata                 CACTTTTCTCTCTTGTTTCGGAGAAATAAGTGGCTCACGAGGAATGGAAA
RRIC110_atp8                      CACTTTGATCTCTTGTTTCGGAGAAATAAGTGGCTCGCGAGGAATGGAAA
RRIM600_atp8                      CACTTTGATCTCTTGTTTCGGAGAAATAAGTGGCTCGCGAGGAATGGAAA
RRIT251_atp8                      CACTTTGATCTCTTGTTTCGGAGAAATAAGTGGCTCGCGAGGAATGGAAA
RRII105_atp8                      CACTTTGATCTCTTGTTTCGGAGAAATAAGTGGCTCGCGAGGAATGGAAA
Cycas taitungensis                CACTTATATATCCTGTTTCGGAGAAATAAGTGGCTCACGAGGAATGGAAA
                                   ****   * ** *************** ******  ****  * ***

Citrullus lanatus                 GAAATATATTCTATTTGATCTCGAAGTCCTCATATAGCACTTCTTCC---
Cucurbita pepo                    GAAATATATTCTATTTGATCTCGAAGTCCTCATATAGCACTTCTTCC---
Beta macrocarpa                   GAAACATATTCTATTTGATCTCGAAGTCTTCATATAGCACTTCTTCC---
Beta vulgaris maritima            GAAACATATTCTATTTGATCTCGAAGTCTTCATATAGCACTTCTTCC---
Beta vulgaris vulgaris            GAAACATATTCTATTTGATCTCGAAGTCTTCATATAGCACTTCTTCC---
Silene latifolia                  GAAACATATTCTATTTGATCTTGGAGTCTTCATATAGCACTTCGTTCTGG
BPM24_atp8                        GAAACATATTCTATTTGATCTCGAAGTCCTCATATAGCATTTCTTCT---
PB235_atp8                        GAAACATATTCTATTTGATCTCGAAGTCCTCATATAGCATTTCTTCT---
Lotus japonicus                   GAAACATCTTATATTTTATCTCGAAGTCCTCATATGGCGCTTCTTCTTCA
Millettia pinnata                 GAAACATCTTATATTTGATCTCGAAGTCCTCATATGGCGCTTCTTCTTCA
RRIC110_atp8                      GAAACATATTCTATTTGATCTCGAAGTCCTCATATAGCATTTCTTCT---
RRIM600_atp8                      GAAACATATTCTATTTGATCTCGAAGTCCTCATATAGCATTTCTTCT---
RRIT251_atp8                      GAAACATATTCTATTTGATCTCGAAGTCCTCATATAGCATTTCTTCT---
RRII105_atp8                      GAAACATATTCTATTTGATCTCGAAGTCCTCATATAGCATTTCTTCT---
Cycas taitungensis                GGAACATTCTATATCTGATTTCGAAGTATCCATATAGCACTTATTCC---
                                  * ** **  * *** * ** * * ***    ***** **  **  *

Citrullus lanatus                 -AATCCT-----------GGATGGGGGATCGCTTGTA------GGAATGA
Cucurbita pepo                    -AATCCT-----------GGATGGGGGATCGCTTGTA------GGAATGA
Beta macrocarpa                   -AATCCT-----------GGATGGGTGATCACTTGTA------AGAATGA
Beta vulgaris maritima            -AATCCT-----------GGATGGGTGATCACTTGTA------AGAATGA
Beta vulgaris vulgaris            -AATCCT-----------GGATGGGTGATCACTTGTA------AGAATGA
```

```
Silene latifolia                  GAATTCCTTTCAAGGACGGGATGGGGGATCACTGGTA------AGAATGA
BPM24_atp8                        -AATCCT-----------GGATGGGGGATCACTTGTACTTGTAGTAATGA
PB235_atp8                        -AATCCT-----------GGATGGGGGATCACTTGTACTTGTAGTAATGA
Lotus japonicus                   -AATCCT-----------GGATGGGTGATCACTTGTA------GGAATGA
Millettia pinnata                 -AATCCT-----------GGGTGGGTGATCACTTGTA------GGAATGA
RRIC110_atp8                      -AATCCT-----------GGATGGGGGATCACTTGTACTTGTAGTAATGA
RRIM600_atp8                      -AATCCT-----------GGATGGGGGATCACTTGTACTTGTAGTAATGA
RRIT251_atp8                      -AATCCT-----------GGATGGGGGATCACTTGTACTTGTAGTAATGA
RRII105_atp8                      -AATCCT-----------GGATGGGGGATCACTTGTACTTGTAGTAATGA
Cycas taitungensis                -AATCCT-----ATATCTGGATGGAGGATTCCTTGTA------GGAATGA
                                   *** *            ** ***  ***  ** ***        *****

Citrullus lanatus                 CATAATGCTAATCCATGTTCTACACGGCCAAGGAAGCATCGTTTTTTAAT
Cucurbita pepo                    CATAATGCTAATCCATGTTCTACACGGCCAAGGAAGCATCGTTTTTTAAT
Beta macrocarpa                   CATAATGCTAATCCATGTTCTACACGGCCAAGAAAG--TGGGAAAATAGA
Beta vulgaris maritima            CATAATGCTAATCCATGTTCTACACGGCCAAGAAAG--TGGGAAAATAGA
Beta vulgaris vulgaris            CATAATGCTAATCCATGTTCTACACGGCCAAGAAAG--TGGGAAAATAGA
Silene latifolia                  CATAACGCTAATCCATGCTCTAAACGGCCAAGAAAG--CGGAAACATAAA
BPM24_atp8                        CATAATGCTAATCCATGTTCUACACGGCCAAGGAAG--CATCGTTTTTTA
PB235_atp8                        CATAATGCTAATCCATGTTCUACACGGCCAAGGAAG--CATCGTTTTTTA
Lotus japonicus                   CATAATGCTAATCCATGTTCUACACGGCCAAGGAAGAATCAAAAAATAA-
Millettia pinnata                 CATAATGCTAATCCATGTTCUACACGGCCAAGGAAGAATAAAAAAATAAT
RRIC110_atp8                      CATAATGCTAATCCATGTTCUACACGGCCAAGGAAGCATCGTTTTTTAA-
RRIM600_atp8                      CATAATGCTAATCCATGTTCUACACGGCCAAGGAAGCATCGTTTTTTAA-
RRIT251_atp8                      CATAATGCTAATCCATGTTCUACACGGCCAAGGAAGCATCGTTTTTTAA-
RRII105_atp8                      CATAATGCTAATCCATGTTCUACACGGCCAAGGAAGCATCGTTTTTTAA-
Cycas taitungensis                CATAATGCTGATCCATGTTTTACACGGCCAGGTAAACATCGTTTGTUAAG
                                  ***** *** ******* *  * ******* * **

Citrullus lanatus                 CTCATTATGACTTGGTCGTTCGGAAGAGATTCTTTCTCGATCAGA----A
Cucurbita pepo                    CTCATTATGACTTGGTCGTTCGGAAGAGATTCTTTCTCGATCAGAT---A
Beta macrocarpa                   AAGATGTTAATAACCATCATAGTAGGCATTTTGCTCTTAGTTGTT-----
Beta vulgaris maritima            AAGATGTTAATAACCATCATAGTAGGCATTTTGCTCTTAGTTGTT-----
Beta vulgaris vulgaris            AAGATGTTAATAACCATCATAGTAGGCATTTTGCTCTTAGTTGTT-----
Silene latifolia                  AAAATTTATATAACCATCATGGTGGGCATTCTTTTTACACTACTTTCCCT
BPM24_atp8                        A-------------------------------------------------
PB235_atp8                        A-------------------------------------------------
Lotus japonicus                   --GAATGGTATTGCTTCTTTTATAATAG-----CGTTCGTTCATA-----
Millettia pinnata                 AAAAAAGGTATTGCTTTTTTTATAATAGTTGCTCGTTCGTTCATA-----
RRIC110_atp8                      --------------------------------------------------
RRIM600_atp8                      --------------------------------------------------
RRIT251_atp8                      --------------------------------------------------
RRII105_atp8                      --------------------------------------------------
Cycas taitungensis                TGGTAATTCCCTGGTCGTTTGGAAGAGAAACCCGGGACAGACCGTTTCTT


Citrullus lanatus                 TAGTAGAAAGCATTACAAGAGATA-----------------------
Cucurbita pepo                    TAGTAGAAAGCATTACAAGAGAT------------------------
Beta macrocarpa                   -------ATTTATTTT-CAATATTATCACCTTACAACCCAAAAGAAG
Beta vulgaris maritima            -------ATTTATTTT-CAATATTATCACCTTACAACCCAAAAGAAG
Beta vulgaris vulgaris            -------ATTTATTTT-CAATATTATCACCTTACAACCCAAAAGAAG
Silene latifolia                  CCTGGGGATTTACTTT-CTATTTTATCATGT----ATCCAAGAAA--
BPM24_atp8                        -----------------------------------------------
PB235_atp8                        -----------------------------------------------
Lotus japonicus                   ------AATTAACTCGACCACTTGTTAGTCTTGCTACACGA------
Millettia pinnata                 ------AATTCACTCGACCACTTGTTAGTCTTG--------------
RRIC110_atp8                      -----------------------------------------------
RRIM600_atp8                      -----------------------------------------------
RRIT251_atp8                      -----------------------------------------------
RRII105_atp8                      -----------------------------------------------
Cycas taitungensis                T------ATTTGTTCGATAAGGGCGGG--------------------
```

```
###################
atp9 multiple sequence alignment
Arabidopsis thaliana              --ATGACAAAGCGTGAGTATA--ATTCTCAA--CCCGAGATGTTAGAAGG
Brassica napus                    GAATGACAAAGCGTGAGGAGA--ATTATCAA--CCCGAGATGTTAGAAGG
Citrullus lanatus                 ----CCAAAAGCGTGAGAATATTATCAACCCTACCCTAGATGTTAGAAGG
Cucurbita pepo                    ----CAAAAAGCGTGACGAT--CATTATTTATTCTCGAGATGTTAGAAGG
Beta vulgaris maritima            -AATGAAAAAGCGTTAGGCAA--ATGATCTAC-TCTGTTATGTTAGAAGG
Beta vulgaris vulgaris            ---TGAAAAAGCGTTAGGCAA--ATGATCTAC-TCTGTTATGTTAGAAGG
Beta macrocarpa                   -AATGAAAAAGCGTTAGGCGA--ATGATCTAC-TCTGTTATGTTAGAAGG
Megaceros aenigmaticus            ---------------------------------------ATGCTAGAAGG
Phaeoceros laevis                 ---------------------------------------ATGCTAGAAGG
Cycas taitungensis                ------AAGCGGGAACTAACGAGACAAAAACTTCTCGATATGTCGGAAGG
Silene latifolia                  ------GCAAAGCGTATTCTTAATAAGAATTTTCTAATTATGTTAGAAGG
BPM24_atp9                        ---------------------------------------ATGTTAGAAGG
RRII105_atp9                      ---------------------------------------ATGTTAGAAGG
RRIC110_atp9                      ---------------------------------------ATGTTAGAAGG
PB235_atp9                        ---------------------------------------ATGTTAGAAGG
RRIT251_atp9                      ---------------------------------------ATGTTAGAAGG
RRIM600_atp9                      ---------------------------------------ATGTTAGAAGG
Pleurozia purpurea                ---------------------------------------ATGCTAGAAGG
Oryza sativa Indica               ---GCGTGACGAG-CAAAGTCAAGTCTCCACGACTCGACATGTTAGAAGG
Oryza sativa Japonica             ---GCGTGACGAG-CAAAGTCAAGTCTCCACGACTCGACATGTTAGAAGG
Zea mays mays                     ---GTGAAACCCGACAATGTCAACTCTC---AACTCTACATGTTAGAAGG
Lotus japonicus                   ------TGAAAGAGCGTGACGATTCTTTCTCCATTCGAGATGTTAGAAGG
Millettia pinnata                 ------TGAAAGAGCGTGACGATTCTTTCTCCATTCGAGATGTTAGAAGG
                                                                         ***   *****

Arabidopsis thaliana              TGCAAAATUAATAGGTGCCGGAGCTGCTACAATTGCTTUAGCGGGAGCTG
Brassica napus                    TGCAAAATUAATAGGTGCCGGAGCTGCTACAATTGCTTUAGCGGGAGCTG
Citrullus lanatus                 TGCAAAATUAATGGGTGCCGGAGCTGCTACAATTGCTTUAGCGGGAGCTG
Cucurbita pepo                    TGCAAAATUAATGGGTGCCGGAGCTGCTACAATTGCTTUAGCGGGAGCTG
Beta vulgaris maritima            CGCAAAATUAATAGGTGCCGGAGCCGCTACAATTGCTTUAGCGGGAGCTG
Beta vulgaris vulgaris            CGCAAAATUAATAGGTGCCGGAGCCGCTACAATTGCTTUAGCGGGAGCTG
Beta macrocarpa                   CGCAAAATUAATAGGTGCCGGAGCCGCTACAATTGCTTUAGCGGGAGCTG
Megaceros aenigmaticus            TGCAAAATTAATTGGAGCAGGAGCAGCTACCATTGCTCCAGCGGGAGCTG
Phaeoceros laevis                 TGCAAAATTAATTGGAGCAGGAGCAGCTATCATAGCTTCAGCGGGAGCTG
Cycas taitungensis                TGCAAAATTAATAGGTGCCGGAGCAGCTACAATTGCTTCAGCGGGAGCTG
Silene latifolia                  AGCTAAATUAATAGGTGCCGGAGCTGCTACAATTGCTTUAGCGGGAGCTG
BPM24_atp9                        TGCAAAATCAATCGGTGCAGGAGCTGCTACAATTGCTTTGGCGGGAGCTG
RRII105_atp9                      TGCAAAATCAATCGGTGCAGGAGCTGCTACAATTGCTTTGGCGGGAGCTG
RRIC110_atp9                      TGCAAAATCAATCGGTGCAGGAGCTGCTACAATTGCTTTGGCGGGAGCTG
PB235_atp9                        TGCAAAATCAATCGGTGCAGGAGCTGCTACAATTGCTTTGGCGGGAGCTG
RRIT251_atp9                      TGCAAAATCAATCGGTGCAGGAGCTGCTACAATTGCTTTGGCGGGAGCTG
RRIM600_atp9                      TGCAAAATCAATCGGTGCAGGAGCTGCTACAATTGCTTTGGCGGGAGCTG
Pleurozia purpurea                TGCAAAATTAATTGGAGCAGGAGCAGCGACCATTGCTTTAGCGGGGGCTG
Oryza sativa Indica               AGCTAAATCAATAGGTGCCGGAGCTGCTACAATTGCTTTAGCGGGAGCTG
Oryza sativa Japonica             AGCTAAATUAATAGGTGCCGGAGCTGCTACAATTGCTTTAGCGGGAGCTG
Zea mays mays                     AGCTAAATUAATAGGTGCTGGAGCTGCTACAATTGCTTTAGCGGGAGCTG
Lotus japonicus                   TGCAAAATTAATAGGTGCCGGAGCTGCTACAATTGCTTUAGCGGGAGCTG
Millettia pinnata                 TGCAAAATUAATGGGTGCCGGAGCTGCTACAATTGCTTUAGCGGGAGCTG
                                   ** **** *** ** ** ***** ** *  ** ***   ***** ****

Arabidopsis thaliana              CTATCGGTATTGGAAACGTATTCAGTTCTTTGAT---------TCATTCT
Brassica napus                    CTATCGGTATTGGAAACGTCTTCAGTTCTTTGAT---------CCATTCT
Citrullus lanatus                 CTGTCGGTATTGGAAACGTCTTCAGTTCTTTGAT---------UCATTCC
Cucurbita pepo                    CTGTCGGTATTGGAAACGTGTTCAGTTCTTTGAT---------CCATTCC
Beta vulgaris maritima            CTATCGGTATTGGAAACGTTTTTAGTTCCTTGAT---------UCATTCC
Beta vulgaris vulgaris            CTATCGGTATTGGAAACGTTTTTAGTTCCTTGAT---------UCATTCC
Beta macrocarpa                   CTATCGGTATTGGAAACGTTTTTAGTTCCTTGAT---------UCATTCC
Megaceros aenigmaticus            CCATAGGTATTGGAAACGTATCTAGTTCTTCGACGTGCGGAGCTCATTCT
Phaeoceros laevis                 CCACAGGTATTGGAAACGTATTTAGTTCTTCGAT---------TCATTCT
```

```
Cycas taitungensis               CTGTCGGTATTGGAAACGTCCTTAGTTCCTCGAT---------CCATCCC
Silene latifolia                 CTGTCGGTATTGGAAACGTCTTTAGTTCTTTGAT---------TCATTCC
BPM24_atp9                       CTGTCGGTATTGGAAATGTCTTCAGTTCTTTGAT---------CCATTCC
RRII105_atp9                     CTGTCGGTATTGGAAATGTCTTCAGTTCTTTGAT---------CCATTCC
RRIC110_atp9                     CTGTCGGTATTGGAAATGTCTTCAGTTCTTTGAT---------CCATTCC
PB235_atp9                       CTGTCGGTATTGGAAATGTCTTCAGTTCTTTGAT---------CCATTCC
RRIT251_atp9                     CTGTCGGTATTGGAAATGTCTTCAGTTCTTTGAT---------CCATTCC
RRIM600_atp9                     CTGTCGGTATTGGAAATGTCTTCAGTTCTTTGAT---------CCATTCC
Pleurozia purpurea               CTGTAGGTATTGGAAACGTTTTTAGTTCTTTGAT---------TCATTCT
Oryza sativa Indica              CTGTCGGTATTGGAAACGTCCTCAGTTCTTCGAT---------TCATTCC
Oryza sativa Japonica            CTGTCGGTATTGGAAACGTUUTCAGTTCTTUGAT---------TCATTCC
Zea mays mays                    CTGTCGGTATTGGAAACGTTUTCAGTTCTTUGAT---------TCATTCC
Lotus japonicus                  CTGTAGGTATTGGAAACGTATTCAGTTCATTAAT---------TCATTCC
Millettia pinnata                CTGTAGGTATTGGAAACGTATTCAGTTCATTAAT---------TCATTCC
                                 *    *********** **    ***** *  *           *** *

Arabidopsis thaliana             GTGGCGCGAAATCCATCATTGGCTAAACAATUATTTGGTTATGCCATTTT
Brassica napus                   GTGGCGCGAAATCCATCATTGGCTAAACAATUATTTGGTTATGCCATTTT
Citrullus lanatus                GTGGCGCGAAATCCATCATTGGCTAAACAATUATTTGGTTATGCCATTTT
Cucurbita pepo                   GTGGCGCGAAATCCCTCATTGGCTAAACAATUATTTGGTTATGCCATTTT
Beta vulgaris maritima           GTGGCGCGAAATCCGTCATTGGCGAAACAATTATTTGGTTATGCTATTTT
Beta vulgaris vulgaris           GTGGCGCGAAATCCGTCATTGGCGAAACAATTATTTGGTTATGCTATTTT
Beta macrocarpa                  GTGGCGCGAAATCCGTCATTGGCGAAACAATTATTTGGTTATGCTATTTT
Megaceros aenigmaticus           GTTGCGCGAAATCCATCCCCGGCTAAGCAATCATCTGGTTATGCTACCTC
Phaeoceros laevis                GTTGCGCGAAATTTATTCTTGGCTAAGCAATTATTTGGTTATGTTACCTT
Cycas taitungensis               GTGGCGCGAAATCCATCATTGGCTAAACAATCATTTGGTCATGCCATTTC
Silene latifolia                 GTGGCTAGAAATCCATCATTGGCTAAACAACTATTUGGTTATGCTATTTT
BPM24_atp9                       GTGGCTAGAAATCCGTCATTAGCTAAACAATTATTTGGTTATGCCATTTT
RRII105_atp9                     GTGGCTAGAAATCCGTCATTAGCTAAACAATTATTTGGTTATGCCATTTT
RRIC110_atp9                     GTGGCTAGAAATCCGTCATTAGCTAAACAATTATTTGGTTATGCCATTTT
PB235_atp9                       GTGGCTAGAAATCCGTCATTAGCTAAACAATTATTTGGTTATGCCATTTT
RRIT251_atp9                     GTGGCTAGAAATCCGTCATTAGCTAAACAATTATTTGGTTATGCCATTTT
RRIM600_atp9                     GTGGCTAGAAATCCGTCATTAGCTAAACAATTATTTGGTTATGCCATTTT
Pleurozia purpurea               GTTGCGCGAAATCCATCATTGGCTAAGCAATCATTTGGTTATGCTATTTT
Oryza sativa Indica              GTGGCGCGAAATCCTTCATTGGCTAAACAATTATTTGGTTATGCCATTTT
Oryza sativa Japonica            GTGGCGCGAAATCCTTCATTGGCTAAACAATTATTTGGTTATGCCATTTT
Zea mays mays                    GTGGCGCGAAATCCTTCATTGGCTAAACAATUATTCGGTTATGCCATTTT
Lotus japonicus                  GTGGCAAGAAATCCATCATTGGCAAAGCAGTTATTCGGATATGCAATCCT
Millettia pinnata                GTGGCAAGAAATCCATCATTGGCAAAACAGTTATTCGGATATGCAATCCT
                                 ** **  *****   *     ** ** **   **  **  ***  *

Arabidopsis thaliana             GGGCTTTGCTCTAACCGAAGCTATTGCATTGTTTGCCCUAATGATGGCCT
Brassica napus                   GGGCTTTGCTCTAACCGAAGCTATTGCATTGTTTGCCCUAATGATGGCCT
Citrullus lanatus                GGGCTTTGCTCTAACCGAAGCTATTGCATTGTTTGCTCUAATGATGGCCT
Cucurbita pepo                   GGGCTTTGCTCTAACCGAAGCTATTGCATTGTTTGCTCUAATGATGGCCT
Beta vulgaris maritima           GGGCTTTGCTCTAACCGAAGCTATCGCATTGTTTGCCCTAATGATGGCCT
Beta vulgaris vulgaris           GGGCTTTGCTCTAACCGAAGCTATCGCATTGTTTGCCCTAATGATGGCCT
Beta macrocarpa                  GGGCTTTGCTCTAACCGAAGCTATCGCATTGTTTGCCCTAATGATGGCCT
Megaceros aenigmaticus           AGGTTTTGCGTCAACCGAAGCTATTGCTTCGCCTGCCTCAACGACGGCGT
Phaeoceros laevis                AGGTTCTGCGTTAACTGAAGTTATTGCTTTGTTTGCCTCAACGACGGCGT
Cycas taitungensis               GGGGTTTGCTTTAACCGAAGCTATTGCATTGTTTGCCTCAATGATGGCGT
Silene latifolia                 AGGCTTTGCTTTAACCGAGGCTATTGCCTTATTCGCCCTAATGATGGCCT
BPM24_atp9                       GGGATTTGCTCTAACGGAAGCTATTGCCTTATTTGCCTTAATGATGGCCT
RRII105_atp9                     GGGATTTGCTCTAACGGAAGCTATTGCCTTATTTGCCTTAATGATGGCCT
RRIC110_atp9                     GGGATTTGCTCTAACGGAAGCTATTGCCTTATTTGCCTTAATGATGGCCT
PB235_atp9                       GGGATTTGCTCTAACGGAAGCTATTGCCTTATTTGCCTTAATGATGGCCT
RRIT251_atp9                     GGGATTTGCTCTAACGGAAGCTATTGCCTTATTTGCCTTAATGATGGCCT
RRIM600_atp9                     GGGATTTGCTCTAACGGAAGCTATTGCCTTATTTGCCTTAATGATGGCCT
Pleurozia purpurea               AGGTTTTGCTCTAACTGAAGCTATTGCTTCGTTTGCCTTAATGATGGCAT
Oryza sativa Indica              GGGCTTTGCTCTCACCGAAGCTATTGCATTGTTTGCCCCAATGATGGCCT
Oryza sativa Japonica            GGGCTTTGCTCTCACCGAAGCTATTGCATTGTTTGCCCUAATGATGGCCT
Zea mays mays                    GGGCTTTGCTCTCACCGAAGCTATTGCATUGTTTGCCCUAATGATGGCCT
```

```
Lotus japonicus                  GGGCTTTGCTCTAACCGAGGCTATTGCCTTGTTCGCATTAATGATGGCCT
Millettia pinnata                GGGCTTTGCTCTAACCGAGGCTATTGCCTTGTTCGCATTAATGATGGCCT
                                  ** * ***     ** ** * *** ** *      **    ** ** *** *

Arabidopsis thaliana             TTTTGATCTTATTCGTATTCTGA---------------------------
Brassica napus                   TTTTGATCTTATTCGTATTCTGAT---------CGAAGAAAGAAGGTTTC
Citrullus lanatus                TTTTGATCTUATTCGTATTCUGAT---------CGAAGAAAGA----AGG
Cucurbita pepo                   TTTTGATCTUATTCGTATTCUGAT---------CGAAGAAGGG----TTC
Beta vulgaris maritima           TTTTGATTTUATTCGTATTCUGATT---TTTTTCAAAGAAAGGGAAGCTG
Beta vulgaris vulgaris           TTTTGATTTUATTCGTATTCUGATT---TTTTTCAAAGAAAGGGAAGCTG
Beta macrocarpa                  TTTTGATTTUATTCGTATTCUGATT---TTTTTCAAAGAAAGGGAAGCTG
Megaceros aenigmaticus           CTTCAACATCATTTGCCTTTTAA---------------------------
Phaeoceros laevis                CTTCAATATCATTTGCCTTTTAA---------------------------
Cycas taitungensis               TTTTGATCTCATCCGTATTCUGAT---AGAGTTGGGGGAGGAACAAAGAG
Silene latifolia                 TTTTAATCTUATTCGTCTTCTGA------GCTTCTGTCTTTCATTCGTAT
BPM24_atp9                       TTTTGATCTTATTTGTATTCUGATCGAAGGTTTCTTTTTCTCTCATAAAG
RRII105_atp9                     TTTTGATCTTATTTGTATTCUGATCGAAGGTTTCTTTTTCTCTCATAAAG
RRIC110_atp9                     TTTTGATCTTATTTGTATTCUGATCGAAGGTTTCTTTTTCTCTCATAAAG
PB235_atp9                       TTTTGATCTTATTTGTATTCUGATCGAAGGTTTCTTTTTCTCTCATAAAG
RRIT251_atp9                     TTTTGATCTTATTTGTATTCUGATCGAAGGTTTCTTTTTCTCTCATAAAG
RRIM600_atp9                     TTTTGATCTTATTTGTATTCUGATCGAAGGTTTCTTTTTCTCTCATAAAG
Pleurozia purpurea               TTTCAATATCATTCGTCTTTUAA---------------------------
Oryza sativa Indica              TTCTGATTTCATTCGTTTTCUGATCACATATTTCTTGGTGTAGATATCTC
Oryza sativa Japonica            TTUTGATTTUATTCGTTTTCUGATCACATATTTCTTGGTGTAGATATCTC
Zea mays mays                    TTCTGATCTUATTCGTTTTCTAATTCGTAAAAATAAGGTGTAGTTTCTCC
Lotus japonicus                  TTTTGATTCTCTTTGTTTTCTAAGTTTCTCCTTTTGAAAGGTGTAGTCCC
Millettia pinnata                TTTTGATTCTATTTGTTTTCTAACTTTCTCCTTTTGAAAGGTGTAGTCCC
                                  *   *     *  *  **   *

Arabidopsis thaliana             ------------------------------------------
Brassica napus                   ATTCAGTCTCATAAA---------------------------
Citrullus lanatus                TTTCAGTCTCATAAAGCAA-----------------------
Cucurbita pepo                   AGTC-GTCTCATAAAGCAAGAC--------------------
Beta vulgaris maritima           GCGGGGCCC---------------------------------
Beta vulgaris vulgaris           GCGGGGCCCCA-------------------------------
Beta macrocarpa                  GCGGGGCCC---------------------------------
Megaceros aenigmaticus           ------------------------------------------
Phaeoceros laevis                ------------------------------------------
Cycas taitungensis               ATAGAGTTATT-------------------------------
Silene latifolia                 TCCGAGATACTACA----------------------------
BPM24_atp9                       AAAGGATCAAAGAACAGGCAGCGAACCAGACTATTTCGCTAG
RRII105_atp9                     AAAGGATCAAAGAACAGGCAGCGAACCAGACTATTTCGCTAG
RRIC110_atp9                     AAAGGATCAAAGAACAGGCAGCGAACCAGACTATTTCGCTAG
PB235_atp9                       AAAGGATCAAAGAACAGGCAGCGAACCAGACTATTTCGCTAG
RRIT251_atp9                     AAAGGATCAAAGAACAGGCAGCGAACCAGACTATTTCGCTAG
RRIM600_atp9                     AAAGGATCAAAGAACAGGCAGCGAACCAGACTATTTCGCTAG
Pleurozia purpurea               ------------------------------------------
Oryza sativa Indica              TCTTTG------------------------------------
Oryza sativa Japonica            TCTTTG------------------------------------
Zea mays mays                    GTCTTCCT----------------------------------
Lotus japonicus                  TGAGGACG----------------------------------
Millettia pinnata                TGAGGAAG----------------------------------
```

###################

ccmFc multiple sequence alignment

```
Beta macrocarpa                  ATGGTCCAACTACATAACTTTTTCTTTTTCATTACTTUCATGGTCGTGCU
Beta vulgaris maritima           ATGGTCCAACTACATAACTTTTTCTTTTTCATTACTTUCATGGTCGTGCU
Silene latifolia                 ATGGTCCAACTACATAACTTTTTCTTTTTCATTACTTUCATGGTCGTGCU
PB235_ccmFc                      ATGGTCCAACTACATAACTTTTTCTTTTTCATTACTTCCATGGTCGTGCC
RRIT251_ccmFc                    ATGGTCCAACTACATAACTTTTTCTTTTTCATTACTTCCATGGTCGTGCC
RRIC110_ccmFc                    ATGGTCCAACTACATAACTTTTTCTTTTTCATTACTTCCATGGTCGTGCC
```

```
BPM24_ccmFc                    ATGGTCCAACTACATAACTTTTTCTTTTTCATTACTTCCATGGTCGTGCC
RRII105_ccmFc                  ATGGTCCAACTACATAACTTTTTCTTTTTCATTACTTCCATGGTCGTGCC
RRIM600_ccmFc                  ATGGTCCAACTACATAACTTTTTCTTTTTCATTACTTCCATGGTCGTGCC
Brassica napus                 ATGGTCCAACTACATAACTTTTTCTTTTTTATTCTTTTTATGGTCGTGCU
Carica papaya                  ATGGTCCAACTACATAACTTTTTCTTTTTTATTATTTTGATGGTCGTGCC
Citrullus lanatus              ATGGTCCAACTAAATAACTTTTTCTTTTTCATTACTTCCATGGTCGTGCC
Cucurbita pepo                 ATGGTCCAACTAAATAACTTTTTCTTTTTCATTACTTCCATGGTCGTGCC
Lotus japonicus                ATGGTCCAACTACATAACTTTTTCTTTTTCATTACTTUTATGGTCGTGCU
Millettia pinnata              ATGGTCCAACTACATAACTTTTTATTTTTCATTACTTUTATGGTCGTGCU
Vigna radiata                  ATGGTCCAACTACATAACTTTTTCTTTTTCATTACTTCTATGGTCGTGCC
Oryza sativa Indica            ATGGTCCAACTACAGAACTTCTTCTTTTTCATTACTTCCATGGTCGTGCC
Oryza sativa Japonica          ATGGTCCAACTACAGAACTTCTTCTTTTTCATTACTTUCATGGTCGTGCU
Tripsacum dactyloides          ATGGTCCAACTACAGAACTTCTTCTTTTTCATTACTTCCATGGTCGTGCC
Zea luxurians                  ATGGTCCAACTACAGAACTTCTTCTTTTTCATTACTTCCATGGTCGTGCC
Zea mays parviglumis           AUGGTCCAACTACAGAACTTCTTCTTTTTCATTACTTCCATGGTCGTGCC
Zea perennis                   ATGGTCCAACTACAGAACTTCTTCTTTTTCATTACTTCCATGGTCGTGCC
Sorghum bicolor                ATGGTCCAACTACAGAACTTCTTCTTTTTCATTACTTCCATGGTCGTGCC
Cycas taitungensis             ATGGTCCAACCACAGAACTTCTTCTTTTTCATTACTTTCATGGTCGTGCC
                               * ******** * * ***** ** ***** ***  **  **********

Beta macrocarpa                TTGTGGCACGGCAGCACCCGTACTATTGAAATGGTTCGTCAGTAGAGATG
Beta vulgaris maritima         TTGTGGCACGGCAGCACCCGTACTATTGAAATGGTTCGTCAGTAGAGATG
Silene latifolia               TTGTGGCACGGCAGCACCCGTACTATTGAAATGGTTCGTCAGTAGAGATG
PB235_ccmFc                    TCGTGGCACGGCAGCACCCGTACTATTGAAATGGTTTGTCAGTAGAGATG
RRIT251_ccmFc                  TCGTGGCACGGCAGCACCCGTACTATTGAAATGGTTTGTCAGTAGAGATG
RRIC110_ccmFc                  TCGTGGCACGGCAGCACCCGTACTATTGAAATGGTTTGTCAGTAGAGATG
BPM24_ccmFc                    TCGTGGCACGGCAGCACCCGTACTATTGAAATGGTTTGTCAGTAGAGATG
RRII105_ccmFc                  TCGTGGCACGGCAGCACCCGTACTATTGAAATGGTTTGTCAGTAGAGATG
RRIM600_ccmFc                  TCGTGGCACGGCAGCACCCGTACTATTGAAATGGTTTGTCAGTAGAGATG
Brassica napus                 TTGTGGCACGGCAGCACCCGTACTATTGAAATGGTTCGTCAGTAGAGATG
Carica papaya                  TCGTGGCACGGCAGCACCCGTACTATTGAAATGGTTCGTCAGTAGAGATG
Citrullus lanatus              TCGTGGCACGGCAGCACCCGTACTATTGAAATGGTTTGTCAGTAGAGATG
Cucurbita pepo                 TCGTGGCACGGCAGCACCCGTACTATTGAAATGGTTTGTCAGTAGAGATG
Lotus japonicus                TUGTGGCACGGCAGCACCCTTACTATTGAAATGGTTCGTCAGTAGAGATG
Millettia pinnata              TUGTGGCACGGCAGCACCCTTACTATTGAAATGGTTCGTCAGTAGAGATG
Vigna radiata                  TCGTGGCACGGCAGCACCCTTACTATTGAAATGGTTCGTCAGTAGAGATG
Oryza sativa Indica            CCGTGGCACGGCAGCACCCGTACTATTGAAATGGTTCGTCAGTAGAGATG
Oryza sativa Japonica          CUGTGGCACGGCAGCACCCGTACTATTGAAATGGTTCGTCAGTAGAGATG
Tripsacum dactyloides          CCGTGGCACGGCAGCACCCGTACTATTGAAATGGTTCGTCAGTAGAGATG
Zea luxurians                  CCGTGGCACGGCAGCACCCGTACTATTGAAATGGTTCGTCAGTAGAGATG
Zea mays parviglumis           CCGTGGCACGGCAGCACCCGTACTATTGAAATGGTTCGTCAGTAGAGATG
Zea perennis                   CCGTGGCACGGCAGCACCCGTACTATTGAAATGGTTCGTCAGTAGAGATG
Sorghum bicolor                CCGTGGCACGGCAGCACCCGTACTATTGAAATGGTTCGTCAGTAGAGATG
Cycas taitungensis             TCGTGGCACGGCAGCACCCGTACTATTGAAATGGTTCGTCAGTAGAGATG
                                 ***************** **************** *************

Beta macrocarpa                TTTCCACAGGTGCCCCTTTTTUUAATGGTACTTTAATTCCTATTCTTATC
Beta vulgaris maritima         TTTCCACAGGTGCCCCTTTTTUUAATGGTACTTTAATTCCTATTCTTATC
Silene latifolia               TTUCCACAGGTGCCCCTTTTTUUAATGGTACTTTAATTCCTATTCTTATC
PB235_ccmFc                    TTCCCATAGGTGCCCCTTCTTCCAATGGTACTATAAATCCTATTCCTATA
RRIT251_ccmFc                  TTCCCATAGGTGCCCCTTCTTCCAATGGTACTATAAATCCTATTCCTATA
RRIC110_ccmFc                  TTCCCATAGGTGCCCCTTCTTCCAATGGTACTATAAATCCTATTCCTATA
BPM24_ccmFc                    TTCCCATAGGTGCCCCTTCTTCCAATGGTACTATAAATCCTATTCCTATA
RRII105_ccmFc                  TTCCCATAGGTGCCCCTTCTTCCAATGGTACTATAAATCCTATTCCTATA
RRIM600_ccmFc                  TTCCCATAGGTGCCCCTTCTTCCAATGGTACTATAAATCCTATTCCTATA
Brassica napus                 TTUCCACGGGTGCCCCTTTTUCCAATGGTACTATAATTCCTATTCUTATC
Carica papaya                  TTCCCACAGGTGCCCCTTCTTCCAATGGTACTATAATTCCTATTCCTATC
Citrullus lanatus              TTUCCACAGGTGCCCCTTUTTUCAATGGTACTATAATTCCTATTCUTATC
Cucurbita pepo                 TTUCCACAGGTGCCCCTTUTTUCAATGGTACTATAATTCCTATTCUTATC
Lotus japonicus                TTUCCACAGGTGCCCCTTTTTUCAATGGGACTATAATTCCTATTCTTATC
Millettia pinnata              TTUCCACAGGTGCCCCTTTTTUCAATGGGACTATAATTCCTATTCTTATC
Vigna radiata                  TTCCCACTGGTGCCCCTTTTTCCAATGGGACTATAATTCCTATTCTTATC
```

```
Oryza sativa Indica              TTCCCACTGGTGCCTCTTCTTCCAATGGTACTATAATTCCTATTCCTATC
Oryza sativa Japonica            TTUCCACTGGTGCCTCTTUTTUUAATGGTACTATAATTCCTATTCUTATC
Tripsacum dactyloides            TTCCCACTGGTGCCTCTTCTTCCAATGGTACTATAATTCCTATTCCTATC
Zea luxurians                    TTCCCACTGGTGCCTCTTCTTCCAATGGTACTATAATTCCTATTCCTATC
Zea mays parviglumis             TTCCCACTGGTGCCTCTTCTTCCAATGGTACTATAATTCCTATTCCTATC
Zea perennis                     TTCCCACTGGTGCCTCTTCTTCCAATGGTACTATAATTCCTATTCCTATC
Sorghum bicolor                  TTCCCACTGGTGCCTCTTCTTCCAATGGTACTATAATTCCTATTCCTATC
Cycas taitungensis               TTCCCACAGGTGCTCCTTCTCCCAGTGGTACTCTAATTCCTATTCCTATA
                                 ** ***  *****  *** *   * *** *** *** ******** ***

Beta macrocarpa                  UCTTUATTCCTTCTTTTGGTTTATUTACATTCCAGGAAATTCATACGCTC
Beta vulgaris maritima           UCTTUATTCCTTCTTTTGGTTTATUTACATTCCAGGAAATTCATACGCTC
Silene latifolia                 UCTTUATTCCTTCTTTTGGTTTATCTACATTCCAGGAAATTCATACGCTC
PB235_ccmFc                      TCTTCATTCCCTCTTTTGGTCTATCTACATTCCAGGAAATTCATACGCTC
RRIT251_ccmFc                    TCTTCATTCCCTCTTTTGGTCTATCTACATTCCAGGAAATTCATACGCTC
RRIC110_ccmFc                    TCTTCATTCCCTCTTTTGGTCTATCTACATTCCAGGAAATTCATACGCTC
BPM24_ccmFc                      TCTTCATTCCCTCTTTTGGTCTATCTACATTCCAGGAAATTCATACGCTC
RRII105_ccmFc                    TCTTCATTCCCTCTTTTGGTCTATCTACATTCCAGGAAATTCATACGCTC
RRIM600_ccmFc                    TCTTCATTCCCTCTTTTGGTCTATCTACATTCCAGGAAATTCATACGCTC
Brassica napus                   TCTTUATTCUCTCTTTTGGTCTATCTACATTCCAGGAAAATCATACGCTC
Carica papaya                    CCTTCATTCCCTCTTTTGGTCTATCTACATTCCAGGAAATTCATACGCTC
Citrullus lanatus                TCTTTATTCUCTCTTTTGGTCTATCTACATTCCAGGAAATTCATACGCTC
Cucurbita pepo                   TCTTTATTCUCTCTTTTGGTCTATCTACATTCCAGGAAATTCATACGCTC
Lotus japonicus                  UCTTUATTCCCTCTTTTGGTCTATCTACATTCCAGGAAATTCATACGCTC
Millettia pinnata                UCTTUATTCCCTCTTTTGGTCTATCTACATTCCAGGAAATTCATACGCTC
Vigna radiata                    CCTTCATTCCCTCTTTTGGTCTATCTACATTCCAGGAAATTCATACGCTC
Oryza sativa Indica              TCTTTATTCCCTTTTTTGGTCTATCTACATTCAAGGAAATTCATACGCTC
Oryza sativa Japonica            TCTTTATTCUCTTTTTTGGTCTATCTACATTCAAGGAAATTUATACGCTC
Tripsacum dactyloides            TCTTTATTCCCTTTTTTGGTCTATCTACATTCAAGGAAATTCATACGCTC
Zea luxurians                    TCTTTATTCCCTTTTTTGGTCTATCTACATTCAAGGAAATTCATACGCTC
Zea mays parviglumis             TCTTTATTCCCTTTTTTGGTCTATCTACATTCAAGGAAATTCATACGCTC
Zea perennis                     TCTTTATTCCCTTTTTTGGTCTATCTACATTCAAGGAAATTCATACGCTC
Sorghum bicolor                  TCTTTATTCCCTTTTTTGGTCTATCTACATTCAAGGAAATTCATACGCTC
Cycas taitungensis               TCTTCATTACCTTTTCTAGTCCATCTGCATTCCAGGGAATTCATACGCTC
                                  *** ***   * ** * **  ** * ***** *** ** * ********

Beta macrocarpa                  TATGGACGGAGTAAAAAGTGGAGTCTTGGTCAGAGCAAGTTGCCCTATTT
Beta vulgaris maritima           TATGGACGGAGTAAAAAGTGGAGTCTTGGTCAGAGCAAGTTGCCCTATTT
Silene latifolia                 CATGGACAGAGTAAAAAGTGGAGTCTTGGTCAGAGCAAGCTGCCCTATTT
PB235_ccmFc                      CATGGACGGAGCAAAAAGTGGAGTCTTGGTCAGAGCAAGCCGCCCTATTC
RRIT251_ccmFc                    CATGGACGGAGCAAAAAGTGGAGTCTTGGTCAGAGCAAGCCGCCCTATTC
RRIC110_ccmFc                    CATGGACGGAGCAAAAAGTGGAGTCTTGGTCAGAGCAAGCCGCCCTATTC
BPM24_ccmFc                      CATGGACGGAGCAAAAAGTGGAGTCTTGGTCAGAGCAAGCCGCCCTATTC
RRII105_ccmFc                    CATGGACGGAGCAAAAAGTGGAGTCTTGGTCAGAGCAAGCCGCCCTATTC
RRIM600_ccmFc                    CATGGACGGAGCAAAAAGTGGAGTCTTGGTCAGAGCAAGCCGCCCTATTC
Brassica napus                   CATGGACGGAGCAAAAAGTGGAGTCTTGGTCAGAGCAAGTCGCCCTATTC
Carica papaya                    CATGGACGGAGCAAAAAGTGGAGTTTTGGTCAGAGCAAGTCGCCCTATTC
Citrullus lanatus                CATGGACGGAGCAAAAAGTGGAGTCTTGGTCAGAGCAAGCCGCCCTATTC
Cucurbita pepo                   TCTGGACGGAGCAAAAAGTGGAGTCTTGGTCAGAGCAAGCCGCCCTATTC
Lotus japonicus                  CATGGACGGAGCAAAAAGTGGAGTCTTGGTCAGAGCAAGCCGCCCTATTC
Millettia pinnata                CATGGACGGAGCAAAAAGCGGAGTCTTGGTCAGAGCAAGCCGCCCTATTC
Vigna radiata                    CATGGACGGAGCAAAAAGTGGAGTCTTGGTCAGAGCAAGCCGCCCTATTC
Oryza sativa Indica              CATGGACAGAGCAAAAAGTGGAGTGTTGGTCAAAGCAAGCCGCCCTATTC
Oryza sativa Japonica            CATGGACAGAGCAAAAAGTGGAGTGTTGGTCAAAGCAAGCCGCCCTATTC
Tripsacum dactyloides            CATGGACAGAGCAAAAAGTGGAGTGTTGGTCAAAGCAAGCCGCCCTATTC
Zea luxurians                    CATGGACAGAGCAAAAAGTGGAGTGTTGGTCAAAGCAAGCCGCCCTATTC
Zea mays parviglumis             CATGGACAGAGCAAAAAGTGGAGTGTTGGTCAAAGCAAGCCGCCCTATTC
Zea perennis                     CATGGACAGAGCAAAAAGTGGAGTGTTGGTCAAAGCAAGCCGCCCTATTC
Sorghum bicolor                  CATGGACAGAGCAAAAAGTGGAGTGTTGGTCAAAGCAAGCCGCCCTATTC
Cycas taitungensis               TATGGACGAAGCGAAAAGTATAGTGCTGGTTGGAGCAAGCCGCCCTATCC
                                   *****  **  *****   ***  ****   ******  *******
```

```
Beta macrocarpa                   TATTACCAGACATAATTGGGAGAAGCTCATCCGAAACTAGAGCTAGAAAC
Beta vulgaris maritima            TATTACCAGACATAATTGGGAGAAGCTCATCCGAAACTAGAGCTAGAAAC
Silene latifolia                  TATTACCAGACATAATTGGGAGAAGCTCCTCCGAAACTAGAGCTAGAAAC
PB235_ccmFc                       TATTACCAGACATAATTGGGAGAAGCTCACCCGAAACTAGAGCTAGAAAC
RRIT251_ccmFc                     TATTACCAGACATAATTGGGAGAAGCTCACCCGAAACTAGAGCTAGAAAC
RRIC110_ccmFc                     TATTACCAGACATAATTGGGAGAAGCTCACCCGAAACTAGAGCTAGAAAC
BPM24_ccmFc                       TATTACCAGACATAATTGGGAGAAGCTCACCCGAAACTAGAGCTAGAAAC
RRII105_ccmFc                     TATTACCAGACATAATTGGGAGAAGCTCACCCGAAACTAGAGCTAGAAAC
RRIM600_ccmFc                     TATTACCAGACATAATTGGGAGAAGCTCACCCGAAACTAGAGCTAGAAAC
Brassica napus                    TATTACCAGACATAATTGGGAGAAGCTCATCCGAAACTAGAGCTAGAAAG
Carica papaya                     TATTACCAGACATAATTGGGAGAAGCTCATCCGAAACTAGAGCTAGAAAG
Citrullus lanatus                 TATTACCAGACATAATTGGGAGAAGCTCATCCGAAACTCGAGCTAGAAAC
Cucurbita pepo                    TATTACCAGACATAATTGGGAGAAGCTCATCCGAAACTCGAGCTAGAAAC
Lotus japonicus                   TATTACCAGACATAATTGGGAGAAGCTCATCCGAAACTAGAGCTGGAAAC
Millettia pinnata                 TATTACCAGACATAATTGGGAGAAGCTCATCCGAAACTAGAGCTGGAAAC
Vigna radiata                     TATTACCAGACATAATTGGGAGAAGCTCATCCGAAACTAGAGCTGGAAAC
Oryza sativa Indica               TATTACCAGACAAAATAGGGAGAAGCTCATCCG---------CTAGAAAT
Oryza sativa Japonica             TATTACCAGACAAAATAGGGAGAAGCTCATCCG---------CTAGAAAT
Tripsacum dactyloides             TATTACCAGACAAAATTGGGAGAAGCTCATCCG---------CTAGAAAT
Zea luxurians                     TATTACCAGACAAAATTGGGAGAAGCTCATCCG---------CTAGAAAT
Zea mays parviglumis              TATTACCAGACAAAATTGGGAGAAGCTCATCCG---------CTAGAAAT
Zea perennis                      TATTACCAGACAAAATTGGGAGAAGCTCATCCG---------CTAGAAAT
Sorghum bicolor                   TATTACCAGACAAAATTGGGAGAAGCTCATCCG---------CTAGAAAT
Cycas taitungensis                TATTACCAGACATAATTGGGAGAAGCTCACCCGGAACTGGAGCTAGAAAC
                                  ************ *** ***********  ***         ** **** 

Beta macrocarpa                   GCCTTATTTUGTTTCGTTCCCATTCTTCATTTTCTTCTTCTCGAATTCAA
Beta vulgaris maritima            GCCTTATTTUGTTTCGTTCCCATTCTTCATTTTCTTCTTCTCGAATTCAA
Silene latifolia                  GCCTTATTTAGTTTCGTTCCCATTCTTCATTTTCTTCTTCTCGAATCTTA
PB235_ccmFc                       GCCTTATTTUGTTTCGTTCCCCTTCTTCATTTCCTTCTTCTCGAATCCAA
RRIT251_ccmFc                     GCCTTATTTUGTTTCGTTCCCCTTCTTCATTTCCTTCTTCTCGAATCCAA
RRIC110_ccmFc                     GCCTTATTTUGTTTCGTTCCCCTTCTTCATTTCCTTCTTCTCGAATCCAA
BPM24_ccmFc                       GCCTTATTTUGTTTCGTTCCCCTTCTTCATTTCCTTCTTCTCGAATCCAA
RRII105_ccmFc                     GCCTTATTTUGTTTCGTTCCCCTTCTTCATTTCCTTCTTCTCGAATCCAA
RRIM600_ccmFc                     GCCTTATTTUGTTTCGTTCCCCTTCTTCATTTCCTTCTTCTCGAATCCAA
Brassica napus                    GCCTTATTTTTTTTCGTTCCCGTTCTTCATTTCUGTCTTCTCGAATCCAA
Carica papaya                     GCCTCATTTTGTTTCGTTCCCGTTCTTCATTTCCTTCTTCTCGAATCCAA
Citrullus lanatus                 GCCTUATTTUGTTTCGTTCCCGTTCTTCATTTCCTTCTTCTTGAATCCAA
Cucurbita pepo                    GCCTUATTTUGTTTCGTTCCCCTTCTTCATTTCCTTCTTCTTTCATCCAA
Lotus japonicus                   GCCTCATTTUGTTTCGTTCCCGTTCTTCATTTCCTTCTTCTCGAATCCAA
Millettia pinnata                 GCCTCATTTUGTTTCGTTCCCGTTCTTCATTTCCTTCTTCTCGAATCCAA
Vigna radiata                     GCCTCATTTCGTTTCGTTCCCGTTCTTCATTTCCTTCTTATCGAATCCAA
Oryza sativa Indica               GCTTTATTTCGTTTCGTTCCCGTTCTTCATTTCCTTATTATCGAATCCAT
Oryza sativa Japonica             GCTTTATTTUGTTTUGTTCCCGTTCTTCATTTCUTTATTATCGAATCCAT
Tripsacum dactyloides             GCTTTATTTCGTTTCGTTCCCGTTCTTCATTTCCTTATTATCGAATCCAT
Zea luxurians                     GCTTTATTTCGTTTCGTTCCCGTTCTTCATTTCCTTATTATCGAATCCAT
Zea mays parviglumis              GCTTTATTTCGTTTCGTTCCCGTTCTTCATTTCCTTATTATCGAATCCAT
Zea perennis                      GCTTTATTTCGTTTCGTTCCCGTTCTTCATTTCCTTATTATCGAATCCAT
Sorghum bicolor                   GCTTTATTTCGTTTCGTTCCCGTTCTTCATTTCCTTATTATCGAATCCAT
Cycas taitungensis                GCATCATTCTGTTTCGTTCTCGTTCTTCATCTTATTCTTCTCGGATTCGT
                                  ** * ***   *** **** * ******** *   * ** *   **

Beta macrocarpa                   ------GGGGGACTTCCCCTATTTAGAATCTTTTTGCGGTGTGCTCUGTT
Beta vulgaris maritima            ------GGGGGACTTCCCCTATTTAGAATCTTTTTGCGGTGTGCTCUGTT
Silene latifolia                  TAACTTAGGGGACTTACCCTATTTAGAATCTTTTTGCGGTGTGCTCUGTT
PB235_ccmFc                       ------GGGGGACTTCTCATATTTAGAATCTTTCTGCGGTGTGCTCTGTT
RRIT251_ccmFc                     ------GGGGGACTTCTCATATTTAGAATCTTTCTGCGGTGTGCTCTGTT
RRIC110_ccmFc                     ------GGGGGACTTCTCATATTTAGAATCTTTCTGCGGTGTGCTCTGTT
BPM24_ccmFc                       ------GGGGGACTTCTCATATTTAGAATCTTTCTGCGGTGTGCTCTGTT
RRII105_ccmFc                     ------GGGGGACTTCTCATATTTAGAATCTTTCTGCGGTGTGCTCTGTT
RRIM600_ccmFc                     ------GGGGGACTTCTCATATTTAGAATCTTTCTGCGGTGTGCTCTGTT
Brassica napus                    ------GGGGGACTTCTCATATTTAGAATCTTTCTGCGGTGTGCTCTGTT
```

```
Carica papaya                     ------GGGGGACTTCTCATATTTAGAATCTTTCTGCGGTGTGCTCCGTT
Citrullus lanatus                 ------GGGAGACTTCTCATATTTTGAATCTTTCTGCGGTGTGCTCTGTT
Cucurbita pepo                    ------GGGAGACTTCTCATATTTTTCATCTTTCTGCGGTGTGCTCTGTT
Lotus japonicus                   ------GGGGGACTTCTCATATTTAGAATCTTTCTGCGGTGTGCTCTGTT
Millettia pinnata                 ------GGGGGACTTCTCATATTTAGAATCTTTCTGCGGTGTGCTCTGTT
Vigna radiata                     ------GGGGGACTTCTCATATTTAGAATCTTTCTGCGGTGTGCTCTGTT
Oryza sativa Indica               ------GGGGGACTTGTCATATTTAGAATCTTTCTGCGGTCTGCTCTGTT
Oryza sativa Japonica             ------GGGGGACTTGTCATATTTAGAATCTTTCTGCGGTCTGCTCTGTT
Tripsacum dactyloides             ------GGGGGACTTGTCATATTTAGAATCTTTCTGCGGTCTGCTCTGTT
Zea luxurians                     ------GGGGGACTTGTCATATTTAGAATCTTTCTGCGGTCTGCTCTGTT
Zea mays parviglumis              ------GGGGGACTTGTCATATTTAGAATCTTTCTGCGGTCTGCTCTGTT
Zea perennis                      ------GGGGGACTTGTCATATTTAGAATCTTTCTGCGGTCTGCTCTGTT
Sorghum bicolor                   ------GGGGGACTTGTCATATTTAGAATCTTTCTGCGGTCTGCTCTGTT
Cycas taitungensis                ------GGGAGACTTGTCATATTCAGAATCTTTCCGCGGTGTGCTCCGTT
                                         ** *****  * ****    ******  ***** ***** ***

Beta macrocarpa                   TACTATTCTTTUGTACTTTCTTCTCTTTACC---------ACGCGATAGG
Beta vulgaris maritima            TACTATTCTTTUGTACTTTCTTCTCTTTACC---------ACGCGATAGG
Silene latifolia                  TACTATTCTTTUGTACTTTCTTCTCTTTATC---------ACGCGATAGG
PB235_ccmFc                       TACTATTCTTTUGTACTTTCTTCTCTTTACC---------ACGCGATAGG
RRIT251_ccmFc                     TACTATTCTTTUGTACTTTCTTCTCTTTACC---------ACGCGATAGG
RRIC110_ccmFc                     TACTATTCTTTUGTACTTTCTTCTCTTTACC---------ACGCGATAGG
BPM24_ccmFc                       TACTATTCTTTUGTACTTTCTTCTCTTTACC---------ACGCGATAGG
RRII105_ccmFc                     TACTATTCTTTUGTACTTTCTTCTCTTTACC---------ACGCGATAGG
RRIM600_ccmFc                     TACTATTCTTTUGTACTTTCTTCTCTTTACC---------ACGCGATAGG
Brassica napus                    TACTATTCTTTUGTACTTTCUTCTTTTTAGC---------ACGCGATAGG
Carica papaya                     TACTATTCTTTCGTACTTTCTTCTTTTTACC---------ACGCGATAGG
Citrullus lanatus                 TACTATTCTTTUGTACTCTCTTCTCTTTACC---------ACGCCATAGT
Cucurbita pepo                    TACTATTCTTTUGTACTCTCTTCTCTTTACC---------ACGCCATAGT
Lotus japonicus                   TACTATTCTTTUGTACTCTCTTCTCTTTACC---------ACGCGATAGG
Millettia pinnata                 TACTATTCTTTUGTACTCTCTTCTCTTTACC---------ACGCGATAGG
Vigna radiata                     TACTATTCTTTCGTACTCTCTTCTCTTTACC---------ACGCGATAGG
Oryza sativa Indica               TACAATTCTTTCGTACTCTCTTCTCTTTACC---------ACGCGATAGG
Oryza sativa Japonica             TACAATTCTTTUGTACTCTCTTCTCTTTACC---------ACGCGATAGG
Tripsacum dactyloides             TACAATTCTTTCGTACTCTCTTCTCTTTACC---------ACGCGATAGG
Zea luxurians                     TACAATTCTTTCGTACTCTCTTCTCTTTACC---------ACGCGATAGG
Zea mays parviglumis              TACAATTCTTTCGTACTCTCTTCTCTTTACC---------ACGCGATAGG
Zea perennis                      TACAATTCTTTCGTACTCTCTTCTCTTTACC---------ACGCGATAGG
Sorghum bicolor                   TACAATTCTTTCGTACTCTCTTCTCTTTACC---------ACGCGATAGG
Cycas taitungensis                TACTATTATTTCGTACCCTTTTCCTACCATATAATCATGGGCGCGATAGG
                                  *** *** *** ****  *  **     *            *** ****

Beta macrocarpa                   TCAGCGAA------GCGTGAGCGGGCGCGGAGAAGAGAAGGCCAAACACT
Beta vulgaris maritima            TCAGCGAA------GCGTGAGCGGGCGCGGAGAAGAGAAGGCCAAACACT
Silene latifolia                  TCAGCGAA------GCGTGAGCGGGCGCGGAGAAGAAAACGCCAAACACT
PB235_ccmFc                       TCAGCGAA------GCGTGAGCGGGCGCGGAGAAGGAAACGCCAAACACT
RRIT251_ccmFc                     TCAGCGAA------GCGTGAGCGGGCGCGGAGAAGGAAACGCCAAACACT
RRIC110_ccmFc                     TCAGCGAA------GCGTGAGCGGGCGCGGAGAAGGAAACGCCAAACACT
BPM24_ccmFc                       TCAGCGAA------GCGTGAGCGGGCGCGGAGAAGGAAACGCCAAACACT
RRII105_ccmFc                     TCAGCGAA------GCGTGAGCGGGCGCGGAGAAGGAAACGCCAAACACT
RRIM600_ccmFc                     TCAGCGAA------GCGTGAGCGGGCGCGGAGAAGGAAACGCCAAACACT
Brassica napus                    TCAGCGAA------GCGTGAGCGGGCGCGGAGAAGGAAAGGCCAAACACT
Carica papaya                     TCAGCGAA------GCGTGAGCGGGCGCGGAGAAGGAAAGGCCAAAGACT
Citrullus lanatus                 TCAGCGAA------GCGTGAGCGGGCGCGGAGAAGGAAAGGCCAAACACT
Cucurbita pepo                    TCAGCGAA------GCGTGAGCGGGCGCGGAGAAGTAAAGGCCAAACACT
Lotus japonicus                   TCAGCAAA------GCGTGAGCGGGCGCGAAGAAGGAAACGCCAAAGACT
Millettia pinnata                 TCAGCAAA------GCGTGAGCGGGCGCGGAGAAGGAAACGCCAAAGACT
Vigna radiata                     TCAGCAAA------GCGTGAGCGGGCGCGGAGAAGGAAACGCCAAACACT
Oryza sativa Indica               TCAGCGAA------GCGTGAGCGGGCGCTCCGAAGTAAAGGCCAAACACT
Oryza sativa Japonica             TCAGCGAA------GCGTGAGCGGGCGCTCCGAAGTAAAGGCCAAACACT
Tripsacum dactyloides             TCAGCGAA------GCGTGAGCGGGCGCTCCGAAGTAAAGGCCAAACACT
Zea luxurians                     TCAGCGAA------GCGTGAGCGGGCGCTCCGAAGTAAAGGCCAAACACT
```

```
Zea mays parviglumis          TCAGCGAA------GCGTGAGCGGGCGCTCCGAAGTAAAGGCCAAACACT
Zea perennis                  TCAGCGAA------GCGTGAGCGGGCGCTCCGAAGTAAAGGCCAAACACT
Sorghum bicolor               TCAGCGAA------GCGTGAGCGGGCGCTCCGAAGTAAAGGCCAAACACT
Cycas taitungensis            TTAGCGAACAAAGGGCGTGAGCGGGCGCGGAGAAGAAAACGCCAAACGCT
                              * *** **      **************   ****  ** ******  **

Beta macrocarpa               TCGGCCTAA------CGGGAATGAGCAACGACGAAATGAAAAGAGAAAGT
Beta vulgaris maritima        TCGGCCTAA------CGGGAATGAGCAACGACGAAATGAAAAGAGAAAGT
Silene latifolia              TCGGACTAA------TGGGAATGAGCAACGACGAAATGACAAGATAAAGT
PB235_ccmFc                   TCGGCCTAA------CGGGAATGAGCAACGACGAAATGACAAGATGAGGT
RRIT251_ccmFc                 TCGGCCTAA------CGGGAATGAGCAACGACGAAATGACAAGATGAGGT
RRIC110_ccmFc                 TCGGCCTAA------CGGGAATGAGCAACGACGAAATGACAAGATGAGGT
BPM24_ccmFc                   TCGGCCTAA------CGGGAATGAGCAACGACGAAATGACAAGATGAGGT
RRII105_ccmFc                 TCGGCCTAA------CGGGAATGAGCAACGACGAAATGACAAGATGAGGT
RRIM600_ccmFc                 TCGGCCTAA------CGGGAATGAGCAACGACGAAATGACAAGATGAGGT
Brassica napus                TCGGCCGAA------CGGGAATGAGCAACGACGAAATGACAAGATGAGGT
Carica papaya                 TCGGCCTAA------CGGGAATGAGCAAGGACGAAATGACAAGATGAGGT
Citrullus lanatus             CAGGCCTAA------CGGGAATGAGCAAGGACTCAATTCCAAGATGAGGT
Cucurbita pepo                CAGGCCTAA------CGGGAATGAGCAAGGATGCAATGAGAAGATGAGGT
Lotus japonicus               TCGGCCTAACCCTAACGGGAAT----------------------------
Millettia pinnata             TCGGCCTAAGCCTAACGGGAATGAGCAACAACGAAATGACAAGATGGGGT
Vigna radiata                 TCGGCCTAAGCCTAACGGGAATGAGCAACAACGAAATGACAAGATGGGGT
Oryza sativa Indica           TCGGCCTAA------GGGGAATGAGCAACAA---AATGACAAGATGAGGT
Oryza sativa Japonica         TUGGCCTAA------GGGGAATGAGCAACAA---AATGACAAGATGAGGT
Tripsacum dactyloides         TCGGCCTAA------GGGGAATGAGCAACAA---AATGACAAGATGAGGT
Zea luxurians                 TCGGCCTAA------GGGGAATGAGCAACAA---AATGACAAGATGAGGT
Zea mays parviglumis          TCGGCCTAA------GGGGAATGAGCAACAA---AATGACAAGATGAGGT
Zea perennis                  TCGGCCTAA------GGGGAATGAGCAACAA---AATGACAAGATGAGGT
Sorghum bicolor               TCGGCCTAA------GGGGAATGAGCAACAA---AATGACAAGATGAGGT
Cycas taitungensis            TCGGCCTAA------CGGGAATGAGCAACGACGAAATGACAAGATAAGGT
                                ** * **       ******

Beta macrocarpa               GCCCCGGG------------------CGCCCCCATTTAGA------AAGA
Beta vulgaris maritima        GCCCCGGG------------------CGCCCCCATTTAGA------AAGA
Silene latifolia              GCCCCGGG------------------CGCCCCCATTTAGA------AAGA
PB235_ccmFc                   TTTCTTCT------------------TTCCTCCATTTAGA------AAGA
RRIT251_ccmFc                 TTTCTTCT------------------TTCCTCCATTTAGA------AAGA
RRIC110_ccmFc                 TTTCTTCT------------------TTCCTCCATTTAGA------AAGA
BPM24_ccmFc                   TTTCTTCT------------------TTCCTCCATTTAGA------AAGA
RRII105_ccmFc                 TTTCTTCT------------------TTCCTCCATTTAGA------AAGA
RRIM600_ccmFc                 TTTCTTCT------------------TTCCTCCATTTAGA------AAGA
Brassica napus                GCTCCGGG------------------CACCCCCATTTAGATTTAGAAAGA
Carica papaya                 GCTCTGGG------------------CACCCCCATTTAGA------AAGA
Citrullus lanatus             GCTCCGGG------------------CACCCCCATTTAGA------AAGA
Cucurbita pepo                GCTCCGGG------------------CACCCCCATTTAGA------AAGA
Lotus japonicus               --------------------------------------------------
Millettia pinnata             GCTCCGGG------------------CACCCCCATTTAGATTTAGAAAGA
Vigna radiata                 GCTCCGGG------------------CACCCCCATTTAGA------AAGA
Oryza sativa Indica           GCCCCGGG------------------CACCCCCATATAGA------AAGA
Oryza sativa Japonica         GCCCCGGG------------------CACCCCCATATAGA------AAGA
Tripsacum dactyloides         GCCCCGGG------------------CACCCCCATATAGA------AAGA
Zea luxurians                 GCCCCGGG------------------CACCCCCATATAGA------AAGA
Zea mays parviglumis          GCCCCGGG------------------CACCCCCATATAGA------AAGA
Zea perennis                  GCCCCGGG------------------CACCCCCATATAGA------AAGA
Sorghum bicolor               GCCCCGGG------------------CACCCCCATATAGA------AAGA
Cycas taitungensis            GCCCCGGGAGCTCGTGCCCCCCCCCACCCCCCCATTTAGA------AAGA


Beta macrocarpa               AGGGTCGAAGGGTTTGGGCCTGTAGCTTTCCCCGTCCCCCCTTCGTCGGG
Beta vulgaris maritima        AGGGTCGAAGGGTTTGGGCCTGTAGCTTTCCCCGTCCCCCCTTCGTCGGG
Silene latifolia              AGGGTCGAAGGGTTTGGACCTGTAGCTTTCCCCGTGCCCCCTTCGTCGGG
PB235_ccmFc                   AGGGTCGAAGGTTTTGGGCCTGTAGCTTTCCCCGTCCCCCCTTCGTCGGG
```

```
RRIT251_ccmFc                     AGGGTCGAAGGTTTTGGGCCTGTAGCTTTCCCCGTCCCCCCTTCGTCGGG
RRIC110_ccmFc                     AGGGTCGAAGGTTTTGGGCCTGTAGCTTTCCCCGTCCCCCCTTCGTCGGG
BPM24_ccmFc                       AGGGTCGAAGGTTTTGGGCCTGTAGCTTTCCCCGTCCCCCCTTCGTCGGG
RRII105_ccmFc                     AGGGTCGAAGGTTTTGGGCCTGTAGCTTTCCCCGTCCCCCCTTCGTCGGG
RRIM600_ccmFc                     AGGGTCGAAGGTTTTGGGCCTGTAGCTTTCCCCGTCCCCCCTTCGTCGGG
Brassica napus                    AGGGTCGAAGGTTTTGGGCCTCTAGCTTTCCCCGTCCCCCCTGAGTTGGG
Carica papaya                     GGGGTCGAAGGTTTTGGGCCTGTAGCTTTCCCCGTCCCCCCTTCGTCGGG
Citrullus lanatus                 AGGGTCGAAGGTTTTGGGCCTGTAGCTTTCCCCGTCCGCCCTTCGTCGGG
Cucurbita pepo                    AGGTTCGAAGGTTTTGGGCCTGTAGCTTTC---GTCCGTCCTTCGTCGGG
Lotus japonicus                   --------------------------------------------------
Millettia pinnata                 AGGGTUGAAGGTTTTGGGCCTGTAGCTTTCCCCGTCCCCCCTTCGTCGGG
Vigna radiata                     AGGGTCGAAGGTTTTTGGCCTGTAGCTTTCCCCGTCCCCCCTTCGTCGGG
Oryza sativa Indica               AGGGTCGAAGGTTTTGGGCCTGTAGCTTTCCCCGCTCCCCCCTCGTCGAG
Oryza sativa Japonica             AGGGTUGAAGGTTTTGGGCCTGTAGCTTTCCCCGCTCCCCCCTUGTCGAG
Tripsacum dactyloides             AGGGTCGAAGGTTTTGGGCCTGTAGCTTTCCCCGCCCCCCCCTCGTCGAG
Zea luxurians                     AGGGTCGAAGGTTTTGGGCCTGTAGCTTTCCCCGCCCCCCCCTCGTCGAG
Zea mays parviglumis              AGGGTCGAAGGTTTTGGGCCTGTAGCTTTCCCCGCCCCCCCCTCGTCGAG
Zea perennis                      AGGGTCGAAGGTTTTGGGCCTGTAGCTTTCCCCGCCCCCCCCTCGTCGAG
Sorghum bicolor                   AGGGTCGAAGGTTTTGGGCCTGTAGCTTTCCCCGCCCCCCCCTCGTCGAG
Cycas taitungensis                AGGGGCGAAGGTTTTGGGCCTGTAGCTTCCCCCGTCCCCCCTTCGTCGGG


Beta macrocarpa                   TGGTGCTTGCATGGGGGGTGTGCTACCTGAA------ATCGGG------C
Beta vulgaris maritima            TGGTGCTTGCATGGGGGGTGTGCTACCTGAA------ATCGGG------C
Silene latifolia                  TGGTGCTTGCATGGGGGGTGTGCTACCTGAA------ATCGGG------C
PB235_ccmFc                       CGGTGCTTGTGTGTGGGGTGTGCCACCAGAA------ATCGGG------C
RRIT251_ccmFc                     CGGTGCTTGTGTGTGGGGTGTGCCACCAGAA------ATCGGG------C
RRIC110_ccmFc                     CGGTGCTTGTGTGTGGGGTGTGCCACCAGAA------ATCGGG------C
BPM24_ccmFc                       CGGTGCTTGTGTGTGGGGTGTGCCACCAGAA------ATCGGG------C
RRII105_ccmFc                     CGGTGCTTGTGTGTGGGGTGTGCCACCAGAA------ATCGGG------C
RRIM600_ccmFc                     CGGTGCTTGTGTGTGGGGTGTGCCACCAGAA------ATCGGG------C
Brassica napus                    TGGTGCTTGTGTGGGGGGCGTGCCACCAGAA------ATCGGG------C
Carica papaya                     TGGTGCTTGTGTGGGGGGTGTGCCACCAGAA------ATCGGG------C
Citrullus lanatus                 TGGTGCTTGTGTGTGGGGTGTGCTACCAGAA------ATCGGG------C
Cucurbita pepo                    TGGTACTTGTGTGTGGGGTGTGCTACCAGAA------ATCGGG------C
Lotus japonicus                   --------------------------------------------------
Millettia pinnata                 TGGTGCTTGTGTGGGGGGTGTGCCACCTGAACCTGAAATCGGG------C
Vigna radiata                     TGGTGCTTGTGTGGGGGGTGTGCCACCTGAACCTGAAATCGGG------C
Oryza sativa Indica               TGGTGCTTGTTTGGGG------CCACCTGAA------ATCGGG------C
Oryza sativa Japonica             TGGTGCTTGTTTGGGG------CCACCTGAA------ATCGGG------C
Tripsacum dactyloides             TGGTGCTTGTTTGGGGGGTGTGCCACCTGAA------ATCGGG------C
Zea luxurians                     TGGTGCTTGTTTGGGGGGTGTGCCACCTGAA------ATCGGG------C
Zea mays parviglumis              TGGTGCTTGTTTGGGGGGTGTGCCACCTGAA------ATCGGG------C
Zea perennis                      TGGTGCTTGTTTGGGGGGTGTGCCACCTGAA------ATCGGG------C
Sorghum bicolor                   TGGTGCTTGTTTGGGGGGTGTGCCACCTGAA------ATCGGG------C
Cycas taitungensis                TGGTGCTTGTGGGGGGGGTGTGCCACCCGAGGCAGAGATTGGGTACTATC


Beta macrocarpa                   TTGAAGC------TCCCGCCTTACCAACGAGCCGACAGCTGATGGCT---
Beta vulgaris maritima            TTGAAGC------TCCCGCCTTACCAACGAGCCGACAGCTGATGGCT---
Silene latifolia                  TTGAAGC------TCCCGCCTTACCAACGAGCCGACAGCTGATGGCT---
PB235_ccmFc                       GTGAAGT------TCTCGCCTTACCAACGAGCCGACAGCTGATGGCT---
RRIT251_ccmFc                     GTGAAGT------TCTCGCCTTACCAACGAGCCGACAGCTGATGGCT---
RRIC110_ccmFc                     GTGAAGT------TCTCGCCTTACCAACGAGCCGACAGCTGATGGCT---
BPM24_ccmFc                       GTGAAGT------TCTCGCCTTACCAACGAGCCGACAGCTGATGGCT---
RRII105_ccmFc                     GTGAAGT------TCTCGCCTTACCAACGAGCCGACAGCTGATGGCT---
RRIM600_ccmFc                     GTGAAGT------TCTCGCCTTACCAACGAGCCGACAGCTGATGGCT---
Brassica napus                    TTGAAGC------TCTCGCCTTACCAAGGAGCCGACAGCTGATGGCTATG
Carica papaya                     TTGAAGC------TCTCGCCTTACCAACGAGCCGACAGCTGATGGCTATG
Citrullus lanatus                 TTGAAGC------TCTCGCCTTACCAACGAGCCGACAGCTGATGGCT---
Cucurbita pepo                    TTGAAGC------TCTCGCCTTACCAACGAGCCGGCAGCTGATGGCT---
Lotus japonicus                   --------------------------ACGAGCCGACAGCTGATGGCT---
```

```
Millettia pinnata                 TTGAAGC------TCTCGCCTTACCAACGAGCCGACAGCTGATGGCT---
Vigna radiata                     TTGAAGC------TCTCGCCTTACCAACGAGCCGACAGCTGATGGCT---
Oryza sativa Indica               TTGAAGC------TCTCGCCTTACCAACGAGCCGACTGCTGATGGCT---
Oryza sativa Japonica             TTGAAGC------TCTCGCCTTACCAACGAGCCGACTGCTGATGGCT---
Tripsacum dactyloides             TTGAAGC------TCTCGCCTTACCAACGAGCCGACTGTTGATGGCT---
Zea luxurians                     TTGAAGC------TCTCGCCTTACCAACGAGCCGACTGTTGATGGCT---
Zea mays parviglumis              TTGAAGC------TCTCGCCTTACCAACGAGCCGACTGTTGATGGCT---
Zea perennis                      TTGAAGC------TCTCGCCTTACCAACGAGCCGACTGTTGATGGCT---
Sorghum bicolor                   TTGAAGC------TCTCGCCTTACCAACGAGCCGACTGTTGATGGCT---
Cycas taitungensis                TTGAAGCGCGTTCTCTCGCCTTACCAACGAGTCGACTGCTGATGGCT---
                                                            * *** ** * * ********

Beta macrocarpa                   ---GTTGGTCACGACTACTACCAAAAGGTGAACATGAAGATGAATATTTC
Beta vulgaris maritima            ---GTTGGTCACGACTACTACCAAAAGGTGAACATGAAGATGAATATTTC
Silene latifolia                  ---GTTGGTCACGACTACTACCAAAAAGTGAACATGAGGATGACTATTTC
PB235_ccmFc                       ---GTTGGTCACGACTACTACCAAAAAGCTCCAATGAAGATGAATATTTC
RRIT251_ccmFc                     ---GTTGGTCACGACTACTACCAAAAAGCTCCAATGAAGATGAATATTTC
RRIC110_ccmFc                     ---GTTGGTCACGACTACTACCAAAAAGCTCCAATGAAGATGAATATTTC
BPM24_ccmFc                       ---GTTGGTCACGACTACTACCAAAAAGCTCCAATGAAGATGAATATTTC
RRII105_ccmFc                     ---GTTGGTCACGACTACTACCAAAAAGCTCCAATGAAGATGAATATTTC
RRIM600_ccmFc                     ---GTTGGTCACGACTACTACCAAAAAGCTCCAATGAAGATGAATATTTC
Brassica napus                    GCTGTTGGTCACGACTACTACCAAAAGGTTCCAATGAAGATGAATATTTC
Carica papaya                     GCTGTTGGTCACGACTACTACCAAAAGGCTCCAATGAAGATGAATATTTC
Citrullus lanatus                 ---GTTGGTCACGACTACTACCAAAAAGCTCCAATGAAGATGAATATTTC
Cucurbita pepo                    ---GTTGGTCACGACTACTACCAAAAAGCTCCAATGAAGATGAATATTTC
Lotus japonicus                   ---GTTGGTCACGACTACCACCAAAAAGCTCCAATGAAGATGAATATTTC
Millettia pinnata                 ---GTTGGTCACGACTACCACCAAAAAGCTCCAATGAAGATGAATATTTC
Vigna radiata                     ---GTTGGTCACGACTACCACCAAAAAGCTCCAATGAAGATGAATATTTC
Oryza sativa Indica               ---GTTGGTCACGACTACTACAAAAAAGTGAAGATGAATCTTTCTATTTC
Oryza sativa Japonica             ---GTTGGTCACGACTACTACAAAAAAGTGAAGATGAATCTTTCTATTTC
Tripsacum dactyloides             ---GTTGGTCACGACTACTACAAAAAAGTGAAGATGAATCTTTCTATTTC
Zea luxurians                     ---GTTGGTCACGACTACTACAAAAAAGTGAAGATGAATCTTTCTATTTC
Zea mays parviglumis              ---GTTGGTCACGACTACTACAAAAAAGTGAAGATGAATCTTTCTATTTC
Zea perennis                      ---GTTGGTCACGACTACTACAAAAAAGTGAAGATGAATCTTTCTATTTC
Sorghum bicolor                   ---GTTGGTCACGACTACTACAAAAAAGTGAAGATGAATCTTTCTATTTC
Cycas taitungensis                ---GTTGGTCACGACTGCTACCGAAAAGCTCCAATGAATATGAATATTTC
                                     ************* * **  *** *      ****   *  ******

Beta macrocarpa                   ACATGGGGGAGTGTGCATCTTTATGTTGGGTGTTCTTCTGTC-TAACACA
Beta vulgaris maritima            ACATGGGGGAGTGTGCATCTTTATGTTGGGTGTTCTTCTGTC-TAACACA
Silene latifolia                  ACATGGGGGAGTGTGCATCTTTATCTTGGGTGTTCTTCTGTC-TAACACA
PB235_ccmFc                       ACATGGAGGAGTGTGCATCTGTATGTTGGGTGTTCTTCTGTC-TAACACA
RRIT251_ccmFc                     ACATGGAGGAGTGTGCATCTGTATGTTGGGTGTTCTTCTGTC-TAACACA
RRIC110_ccmFc                     ACATGGAGGAGTGTGCATCTGTATGTTGGGTGTTCTTCTGTC-TAACACA
BPM24_ccmFc                       ACATGGAGGAGTGTGCATCTGTATGTTGGGTGTTCTTCTGTC-TAACACA
RRII105_ccmFc                     ACATGGAGGAGTGTGCATCTGTATGTTGGGTGTTCTTCTGTC-TAACACA
RRIM600_ccmFc                     ACATGGAGGAGTGTGCATCTGTATGTTGGGTGTTCTTCTGTC-TAACACA
Brassica napus                    ACATGGAGGAGTGTGCATCTGTATGTTGGGTGTTCTTCTGTC-GAACACA
Carica papaya                     ACATGGAGGAGTGTGCATCTGTATGTTGGGTGTTCTTCTGTC-TAACACA
Citrullus lanatus                 ACATGGAGGAGTGTGCATCTTTATGTTGGGTGTTCTTCTGTC-TAACACA
Cucurbita pepo                    ACATGGAGGAGTGTGCATCTTTATCTTGGGTGTTCTTCTGTC-TAACACA
Lotus japonicus                   ACATTTTGGAGTGTGCATCTGTATGTTGGGTGTTCTTCTGTC-TAACACA
Millettia pinnata                 ACATTTTGGAGTGTGCATCTGTATGTTGGGTGTTCTTCTGTC-TAACACA
Vigna radiata                     ACATTTTGGAGTGTGCATCTGTATGTTGGGTGTTCTTCTGTC-TAACACA
Oryza sativa Indica               ACATGGAGGAGTGTGCATCTTTATGTTGGGTGTTCTTCTGTCCTAATACG
Oryza sativa Japonica             ACATGGAGGAGTGTGCATCTTTATGTTGGGTGTTCTTCTGTC-TAATACG
Tripsacum dactyloides             ACATGGAGGAGTGTGCATCTTTATGTTGGGTGTTCTTCTGTC-TAATACG
Zea luxurians                     ACATGGAGGAGTGTGCATCTTTATGTTGGGTGTTCTTCTGTC-TAATACG
Zea mays parviglumis              ACATGGAGGAGTGTGCATCTTTATGTTGGGTGTTCTTCTGTC-TAATACG
Zea perennis                      ACATGGAGGAGTGTGCATCTTTATGTTGGGTGTTCTTCTGTC-TAATACG
Sorghum bicolor                   ACATGGAGGAGTGTGCATCTTTATGTTGGGTGTTCTTCTGTC-TAATACG
Cycas taitungensis                ACATGGAGGAGTGTGCATCTTTATTATGGGTGTTATTTTGTC-TGACACG
```

```
                                  ****   ************* ***  ******** ** ****   * **

Beta macrocarpa                   AAGAAGATACAGTTCACTCAACGATTGCCTTTGGGTTCCGAACTCCATAT
Beta vulgaris maritima            AAGAAGATACAGTTCACTCAACGATTGCCTTTGGGTTCCGAACTCCATAT
Silene latifolia                  AAGAAGATACAGTTCACTCAACGATTGCCTTTGGGTTCCGAACTCCATAT
PB235_ccmFc                       AAGAAGATACAGTTCACTCAACGATTGCCTTTGGGTTCCGAACTCCATAT
RRIT251_ccmFc                     AAGAAGATACAGTTCACTCAACGATTGCCTTTGGGTTCCGAACTCCATAT
RRIC110_ccmFc                     AAGAAGATACAGTTCACTCAACGATTGCCTTTGGGTTCCGAACTCCATAT
BPM24_ccmFc                       AAGAAGATACAGTTCACTCAACGATTGCCTTTGGGTTCCGAACTCCATAT
RRII105_ccmFc                     AAGAAGATACAGTTCACTCAACGATTGCCTTTGGGTTCCGAACTCCATAT
RRIM600_ccmFc                     AAGAAGATACAGTTCACTCAACGATTGCCTTTGGGTTCCGAACTCCATAT
Brassica napus                    AAGAAGATACAGTTCACTCAACGATTGCCTTTGGGTTCCGAACTCCATAT
Carica papaya                     AAGAAGATACAGTTCACTCAACGATTGCCTTTGGGTTCCGAACTCCATAT
Citrullus lanatus                 AAGAAGATACAGTTCACTCAACGATTGCCTTTGGGTTCCGAACTCCATAT
Cucurbita pepo                    AAGAAGATAGAGTTCACTCAACGATTGCCTTTGGGTTCCGAACTCCATAT
Lotus japonicus                   AAGAAGATACAGTTCACTCAACGATTGCCTTTGGGTTCCGAACTCCATAT
Millettia pinnata                 AAGAAGATACAGTTCACTCAACGATTGCCTTTGGGTTCCGAACTCCATAT
Vigna radiata                     AAGAAGATACAGTTCACTCAACGATTGCCTTTGGGTTCCGAACTCCATAT
Oryza sativa Indica               AATAAGATACAGTTCACTCAACGATTGCCTTTGGGTTCCGAACTCCATAT
Oryza sativa Japonica             AATAAGATACAGTTCACTCAACGATTGCCTTTGGGTTCCGAACTCCATAT
Tripsacum dactyloides             AATAAGATACAGTTCACTCAACGATTGCCTTTGGGTCCCGAACTCCATAT
Zea luxurians                     AATAAGATACAGTTCACTCAACGATTGCCTTTGGGTCCCGAACTCCATAT
Zea mays parviglumis              AATAAGATACAGTTCACTCAACGATTGCCTTTGGGTCCCGAACTCCATAT
Zea perennis                      AATAAGATACAGTTCACTCAACGATTGCCTTTGGGTCCCGAACTCCATAT
Sorghum bicolor                   AATAAGATACAGTTCACTCAACGATTGCCTTTGGGTCCCGAACTCCATAT
Cycas taitungensis                AAAAAGATACAGTTTACTCAACGATTGCCTTTGGGTCCCGAACTCCATAT
                                  ** ****** **** ********************* *************

Beta macrocarpa                   GGGGAAGGAACGTTGTTGTTTGCGGGGTCTCGATCATTTACATGGACCCA
Beta vulgaris maritima            GGGGAAGGAACGTTGTTGTTTGCGGGGTCTCGATCATTTACATGGACCCA
Silene latifolia                  GGGGAAGGAACGTTGTTGTTTGCGGGGTCTCGATCATTTACATGGACCCA
PB235_ccmFc                       GGGGAAGGAGCGTTGTTGTTTGCGAGGTCTCGATCATTTACATGGACCCA
RRIT251_ccmFc                     GGGGAAGGAGCGTTGTTGTTTGCGAGGTCTCGATCATTTACATGGACCCA
RRIC110_ccmFc                     GGGGAAGGAGCGTTGTTGTTTGCGAGGTCTCGATCATTTACATGGACCCA
BPM24_ccmFc                       GGGGAAGGAGCGTTGTTGTTTGCGAGGTCTCGATCATTTACATGGACCCA
RRII105_ccmFc                     GGGGAAGGAGCGTTGTTGTTTGCGAGGTCTCGATCATTTACATGGACCCA
RRIM600_ccmFc                     GGGGAAGGAGCGTTGTTGTTTGCGAGGTCTCGATCATTTACATGGACCCA
Brassica napus                    GGGGAAGGAGCGTTGTTGTTTGCGAGGTCTCGATCATTTACATGGACCCA
Carica papaya                     GGGGAAGGAGCGTTGTTGTTTGCGAGGTCTCGATCATTTACATGGACCCA
Citrullus lanatus                 GGGGAAGGAGCGTTGTTGTTTGCGAGGTCTCGATCATTTACATGGACCCA
Cucurbita pepo                    GGGGAAGGAGCGTTGTTGTTTGCGAGGTATCGACCATTTACATGGACCTA
Lotus japonicus                   GGGGAAGGAGCGTTGTTGTTTCCGAGGTCTCGATCATTTACATGGACCCA
Millettia pinnata                 GGGGAAGGAGCGTTGTTGTTTCCGAGGTCTCGATCATTTACATGGACCCA
Vigna radiata                     GGGGAAGGAGCGTTGTTGTTTCCGAGGTCTCGATCATTTACATGGACCCA
Oryza sativa Indica               GGGGAAGGAACGTTGTTGTTTGCGAGGTCTCGATCATTTACATGGACCCA
Oryza sativa Japonica             GGGGAAGGAACGTTGTTGTTTGCGAGGTCTCGATCATTTACATGGACCCA
Tripsacum dactyloides             GGGGAAGGAACGTTGTTGTTTGCGAGGTCTCGATCATTTACATGGACCCA
Zea luxurians                     GGGGAAGGAACGTTGTTGTTTGCGAGGTCTCGATCATTTACATGGACCCA
Zea mays parviglumis              GGGGAAGGAACGTTGTTGTTTGCGAGGTCTCGATCATTTACATGGACCCA
Zea perennis                      GGGGAAGGAACGTTGTTGTTTGCGAGGTCTCGATCATTTACATGGACCCA
Sorghum bicolor                   GGGGAAGGAACGTTGTTGTTTGCGAGGTCTCGATCATTTACATGGACCCA
Cycas taitungensis                GGGGAAGGAGCGTTGTTGTTTGCGAGGTTCTGATCATTCACATGGACCCA
                                  ********* *********** ** ***   ** **** ********* *

Beta macrocarpa                   CTTTTCATTCCATTTGTGGGAATTTGATGATCTATAAACCGTCCTTAACG
Beta vulgaris maritima            CTTTTCATTCCATTTGTGGGAATTTGATGATCTATAAACCGTCCTTAACG
Silene latifolia                  CTTTTCATTCCATTTGTGGGAATTTTTTTATCTATAAACCGTCCGTAACG
PB235_ccmFc                       CTTUTCATTCCATTTGTGGGAATTTGATGATCTATAAACCGTCCCTAACG
RRIT251_ccmFc                     CTTUTCATTCCATTTGTGGGAATTTGATGATCTATAAACCGTCCCTAACG
RRIC110_ccmFc                     CTTUTCATTCCATTTGTGGGAATTTGATGATCTATAAACCGTCCCTAACG
BPM24_ccmFc                       CTTUTCATTCCATTTGTGGGAATTTGATGATCTATAAACCGTCCCTAACG
RRII105_ccmFc                     CTTNTCATTCCATTTGTGGGAATTTGATGATCTATAAACCGTCCCTAACG
```

```
RRIM600_ccmFc                     CTTUTCATTCCATTTGTGGGAATTTGATGATCTATAAACCGTCCCTAACG
Brassica napus                    CTTUTCATTCCATTTGTGGGAATTTGATGATCTATAAACCGTCCCTAACG
Carica papaya                     CTTCTCATTCCATTTGTGGGAATTTGATGATCTATAAACCGTCCCTAACG
Citrullus lanatus                 CTTUTCATTCCATTTGTGGTAATTTATGATCTATAAACCGTCCCTAACG
Cucurbita pepo                    CTTUTCATTCCATTTGTGGGAATTTGCTGATCTATAAACCGTCCCTAACG
Lotus japonicus                   CTTUTCATTCCATTTGTGGGAATTTGATGATCTATAAACCGTCCCTAACG
Millettia pinnata                 CTTUTCATTCCATTTGTGGGAATTTGATGATCTATAAACCGTCCCTAACG
Vigna radiata                     CTTCTCATTCCATTTGTGGGAATTTGATGATCTATAAACCGTCCCTAACG
Oryza sativa Indica               CTTCTCATTCCATTTGTGGGAATTTGATGATTTATAAACCGTCCCCAACG
Oryza sativa Japonica             CTTUTCATTCCATTTGTGGGAATTTGATGATTTATAAACCGTCUCCAACG
Tripsacum dactyloides             CTTCTCATTCCATTTGTGGGAATTTTTGATTTATAAACCGTCCCCAACG
Zea luxurians                     CTTCTCATTCCATTTGTGGGAATTTTATGATTTATAAACCGTCCCCAACG
Zea mays parviglumis              CTTCTCATTCCATTTGTGGGAATTTTATGATTTATAAACCGTCCCCAACG
Zea perennis                      CTTCTCATTCCATTTGTGGGAATTTTATGATTTATAAACCGTCCCCAACG
Sorghum bicolor                   CTTCTCATTCCATTTGTGGGAATTTTTTGATTTATAAACCGTCCCCAACG
Cycas taitungensis                CTTTTCATTCCATTTGTGGTAATTCGATGATCTATAAACCGTCCCTAACG
                                  *** *************** ****   * ** ************  ****

Beta macrocarpa                   AACGATCGGCTCATCTTTGAGCATGATGAATCACTTCGTGCC------GA
Beta vulgaris maritima            AACGATCGGCTCATCTTTGAGCATGATGAATCACTTCGTGCC------GA
Silene latifolia                  AACGATCGGCTCATCTTTGAGCATGATGAATCACTTCGTGCC------GA
PB235_ccmFc                       AACGATCGGCTCATGTTTGAGCATGATGAATCACTTCGTGCC------GA
RRIT251_ccmFc                     AACGATCGGCTCATGTTTGAGCATGATGAATCACTTCGTGCC------GA
RRIC110_ccmFc                     AACGATCGGCTCATGTTTGAGCATGATGAATCACTTCGTGCC------GA
BPM24_ccmFc                       AACGATCGGCTCATGTTTGAGCATGATGAATCACTTCGTGCC------GA
RRII105_ccmFc                     AACGATCGGCTCATGTTTGAGCATGATGAATCACTTCGTGCC------GA
RRIM600_ccmFc                     AACGATCGGCTCATGTTTGAGCATGATGAATCACTTCGTGCC------GA
Brassica napus                    AACGATCGGCTCATGTTTGAGCATGATGAATCACTTCATGCC------GA
Carica papaya                     AACGATCGGCTCATGTTTGAGCATGATGAATCACTTCGTGCC------GA
Citrullus lanatus                 AACGATCGGCTCATCTTTGAGCATGATAAATCACTTCGTGCC------GA
Cucurbita pepo                    AACGATCGGCTCATCTTTGAGCATGATAAAGAACTTCGTGCC------GA
Lotus japonicus                   AACGATCGCCTCATGTTTGAGCATGATGAATCACTTCGTGCC------GA
Millettia pinnata                 AACGATCGCCTCATGTTTGAGCATGATGAATCACTTCGTGCC------GA
Vigna radiata                     AATGATCGCCTCATGTTTGAGCATGATGAATCACTTCGTGCC------GA
Oryza sativa Indica               AGCGAAAGGTTCATGTTTGAACATGATGAATCACTTCGTGCC------GA
Oryza sativa Japonica             AGCGAAAGGTTCATGTTTGAACATGATGAATCACTTCGTGCC------GA
Tripsacum dactyloides             AGCGAAAGGTTCATGTTTGAACATGATGAATCACTTCGTGCC------GA
Zea luxurians                     AGCGAAAGGTTCATGTTTGAACATGATGAATCACTTCGTGCC------GA
Zea mays parviglumis              AGCGAAAGGTTCATGTTTGAACATGATGAATCACTTCGTGCC------GA
Zea perennis                      AGCGAAAGGTTCATGTTTGAACATGATGAATCACTTCGTGCC------GA
Sorghum bicolor                   AGCGAAAGGTTCATGTTTGAACATGATGAATCACTTCGTGCC------GA
Cycas taitungensis                AAC---CCATTCATGCTTGAGCATGATGAATCACTTCGTGCCATAATCGA
                                  *         ****  **** ****** **  ***** ****      **

Beta macrocarpa                   CCTGTTGTCAATAAACTTTTGGGCCTCATATGAGAATGGAAAACTGGAGC
Beta vulgaris maritima            CCTGTTGTCAATAAACTTTTGGGCCTCATATGAGAATGGAAAACTGGAGC
Silene latifolia                  CCTGTTGTCAATAAACTTTTGGGCCTCATATGAGAATGGAAAACTGGAGC
PB235_ccmFc                       CCTGTTGCTAATAAACTTTCCGGCCTCATATGAGAATGGAAAACTGGAGC
RRIT251_ccmFc                     CCTGTTGCTAATAAACTTTCCGGCCTCATATGAGAATGGAAAACTGGAGC
RRIC110_ccmFc                     CCTGTTGCTAATAAACTTTCCGGCCTCATATGAGAATGGAAAACTGGAGC
BPM24_ccmFc                       CCTGTTGCTAATAAACTTTCCGGCCTCATATGAGAATGGAAAACTGGAGC
RRII105_ccmFc                     CCTGTTGCTAATAAACTTTCCGGCCTCATATGAGAATGGAAAACTGGAGC
RRIM600_ccmFc                     CCTGTTGCTAATAAACTTTCCGGCCTCATATGAGAATGGAAAACTGGAGC
Brassica napus                    CCTCTTGCTAATAAACTTTCCGGCCTCATATAAGAATGGAAAACTTGAGC
Carica papaya                     CCTCTTGCTAATCAACTTTCCGGCCTCATATGAGAATGGAAAACTGGAGC
Citrullus lanatus                 CCAGTTGCCAATAAACTTTCCGGCCTCATATGAGAATGGAAAACTGGAGC
Cucurbita pepo                    CCAGTTGCCAATAAACTTTCCGGCCTCATATGAGAATGGAAAACAGGAGC
Lotus japonicus                   CCTGTTGCCAATAAACTTTCCGGCCTCATATGATAATGGAAAACTGGAAA
Millettia pinnata                 CCTCTTGCCAATAAACTTTCCGGCCTCATATGAGAATGGAAAACTGGATA
Vigna radiata                     CCTGTTGCCAATAAACTTTCCGGCCTCATATGAGAATGGAAAACTGGAGA
Oryza sativa Indica               CCTGTTGCCCATAAACTTTCCTGCCTCATATGAGAATGGAAAACTGGAAG
Oryza sativa Japonica             CCTGTTGCCUATAAACTTTCCTGCCTCATATGAGAATGGAAAACTGGAAG
```

```
Tripsacum dactyloides          CCTGTTGCCCATAAACTTTCCTGCCTCATATGAGAATGGAAAACTGGAAG
Zea luxurians                  CCTGTTGCCCATAAACTTTCCTGCCTCATATGAGAATGGAAAACTGGAAG
Zea mays parviglumis           CCTGTTGCCCATAAACTTTCCTGCCTCATATGAGAATGGAAAACTGGAAG
Zea perennis                   CCTGTTGCCCATAAACTTTCCTGCCTCATATGAGAATGGAAAACTGGAAG
Sorghum bicolor                CCTGTTGCCCATAAACTTTCCTGCCTCATATGAGAATGGAAAACTGGAAG
Cycas taitungensis             CCTGTTGCCAATCCACTTTTCGGCCTCATATGGGAATGGAAAACTGGATC
                               **  ***   **  *****   *********   **********  **

Beta macrocarpa                ATTTTCTGCATCG------------GTGGATGAAGAATCGCGAACATCCA
Beta vulgaris maritima         ATTTTCTGCATCG------------GTGGATGAAGAATCGCGAACATCCA
Silene latifolia               ATTTTCTGCATCG------------GTGGATGAAGAATCGCGAACATAAA
PB235_ccmFc                    ATTTTCTGCATCG------------GTGGATGAAGAATCGCGAACATAAT
RRIT251_ccmFc                  ATTTTCTGCATCG------------GTGGATGAAGAATCGCGAACATAAT
RRIC110_ccmFc                  ATTTTCTGCATCG------------GTGGATGAAGAATCGCGAACATAAT
BPM24_ccmFc                    ATTTTCTGCATCG------------GTGGATGAAGAATCGCGAACATAAT
RRII105_ccmFc                  ATTTTCTGCATCG------------GTGGATGAAGAATCGCGAACATAAT
RRIM600_ccmFc                  ATTTTCTGCATCG------------GTGGATGAAGAATCGCGAACATAAT
Brassica napus                 ATTTTCTGCATTG------------GTGGATGAAGAATCGCAAACATAAT
Carica papaya                  ATTTTCTGCATCG------------GTGGATGAAGAATCGCAAACATAAG
Citrullus lanatus              ATTTTCTGCATCA------------GTGGATGACGAATCGCGAACATAAT
Cucurbita pepo                 ATTTTCTACATCA------------GTGGATGACGAATCGCGAACATCAG
Lotus japonicus                ATTTTCTGCATCG------------GTGGATGAAGAATCGCGAACATAAT
Millettia pinnata              ATTTTCTGCATCG------------GTGGATGAAAAATCGCAAACATAAT
Vigna radiata                  ATTTTCTGCATCG------------GTGGATGAAAAATCGCGAACATAAG
Oryza sativa Indica            ATTTTCTGCATCG------------GTGGATGAAGAATCACGAACATAAG
Oryza sativa Japonica          ATTTTUTGCATCG------------GTGGATGAAGAATCACGAACATAAG
Tripsacum dactyloides          ATTTTCTG---------------------ATGAAGAATCACGAACATAAG
Zea luxurians                  ATTTTCTG---------------------ATGAAGAATCACGAACATAAG
Zea mays parviglumis           ATTTTCTG---------------------ATGAAGAATCACGAACATAAG
Zea perennis                   ATTTTCTG---------------------ATGAAGAATCACGAACATAAG
Sorghum bicolor                ATTTTCTGCATCG------------GTGGATGAAGAATCACGAACATAAG
Cycas taitungensis             ATTTTCTGCATCGTGAAAGATCGTGGTGGATAGAGAATCGCGAACATAAT
                               ***** *                      **    **** * *****

Beta macrocarpa                AATTTCTGGTTAAGCATGTTCCCAGAAAAAAGATACTTTC---------G
Beta vulgaris maritima         AATTTCTGGTTAAGCATGTTCCCAGAAAAAAGATACTTTC---------G
Silene latifolia               AATTTCTGGTTAAGCATGTTCCCAGAAAAAAGATACTTTC---------G
PB235_ccmFc                    AATTTCTGGTTGACCATGTTCCCAGAAAAAAGATACTTTC---------G
RRIT251_ccmFc                  AATTTCTGGTTGACCATGTTCCCAGAAAAAAGATACTTTC---------G
RRIC110_ccmFc                  AATTTCTGGTTGACCATGTTCCCAGAAAAAAGATACTTTC---------G
BPM24_ccmFc                    AATTTCTGGTTGACCATGTTCCCAGAAAAAAGATACTTTC---------G
RRII105_ccmFc                  AATTTCTGGTTGACCATGTTCCCAGAAAAAAGATACTTTC---------G
RRIM600_ccmFc                  AATTTCTGGTTGACCATGTTCCCAGAAAAAAGATACTTTC---------G
Brassica napus                 AATTTTTGGTTAACCATGTTCCCAGAAAAAAGATACTTTC---------G
Carica papaya                  CATTTTTGGTTAACCATGTTCCCAGAAAAAAGATACTTTC---------G
Citrullus lanatus              AATTTCTGGTTGACCATGTTCCCAGAAAAAAGATACTTTC---------G
Cucurbita pepo                 AATTTCTGGTTGACCATGTTCCCAGAAAAAAGATACTTTC---------G
Lotus japonicus                AATTTATGGTTGACCATGTTCCCAGAAAAAAGATACTTTA---------G
Millettia pinnata              AATTTATGGTTGATCATGTTCCCAGAAAAAAGAGACTTTA---------G
Vigna radiata                  AATTTTTTGTTGATCATGTTCCCAGAAAAAAGATACTTTA---------G
Oryza sativa Indica            AATTTCAGGTTTAGCATGTTCCCAGAAAGAAGATACTTTTTTTCCATTCG
Oryza sativa Japonica          AATTTCAGGTTTAGCATGTTCCCAGAAAGAAGATACTTTTTTTCUATTCG
Tripsacum dactyloides          AATTTCTGGTTTAGCATGTTCCCAGAAAGAAGATACTACTGGTCG---CG
Zea luxurians                  AATTTCTGGTTTAGCATGTTCCCAGAAAGAAGATACTACTGGTCG---CG
Zea mays parviglumis           AATTTCTGGTTTAGCATGTTCCCAGAAAGAAGATACTACTGGTCG---CG
Zea perennis                   AATTTCTGGTTTAGCATGTTCCCAGAAAGAAGATACTACTGGTCG---CG
Sorghum bicolor                AATTTCTGGTTTAGCATGTTCCCAGAAAGAAGATACTTTTTTTCCATTCG
Cycas taitungensis             AATTCCTGGTTGACCATGTCCCCAGAAAAAAGATACTTTTTCTCCATTCA
                                ***    *** * ***** ******** **** ***

Beta macrocarpa                AGAAACAACGAGCACGACTGAAGTGGCTATCCATACAAATCUATTTACGG
Beta vulgaris maritima         AGAAACAACGAGCACGACTGAAGTGGCTATCCATACAAATCUATTTACGG
```

```
Silene latifolia                  AGAAACAACGAGCACGACTGAAGTGGCTATCCATACAAATCUATTTACGG
PB235_ccmFc                       AGAAACGACGAGCACGACTGAAGTGGCTATACATACAAATCTATTTACGG
RRIT251_ccmFc                     AGAAACGACGAGCACGACTGAAGTGGCTATACATACAAATCTATTTACGG
RRIC110_ccmFc                     AGAAACGACGAGCACGACTGAAGTGGCTATACATACAAATCTATTTACGG
BPM24_ccmFc                       AGAAACGACGAGCACGACTGAAGTGGCTATACATACAAATCTATTTACGG
RRII105_ccmFc                     AGAAACGACGAGCACGACTGAAGTGGCTATACATACAAATCTATTTACGG
RRIM600_ccmFc                     AGAAACGACGAGCACGACTGAAGTGGCTATACATACAAATCTATTTACGG
Brassica napus                    AGAAAGGACGAGCACGGCTGAAGTGGCTATACATACAAATCTATTTACGG
Carica papaya                     AGAAAGGATGAGCACGACTGAAGTGGCTATACATACAAATCTATTTACGG
Citrullus lanatus                 AGAAACGACGAGCACGACTGAAGTAGCTATACATACAAATCUATTTACGG
Cucurbita pepo                    AGAAAGGACGAGCACGACTGAAGTAGCTATACATACTAATCUATTTACAG
Lotus japonicus                   AGAAACAACGAGCACGACTGAAGTGGCTATACATACAAATCUATTTACGG
Millettia pinnata                 AGAAACAACGAGCACGACTGAAGTGGCTATACATACAAATCUATTTACGG
Vigna radiata                     AGAAACAACGAGCACGACTGAAGTGGCTATACATACAAATCCATTTACGG
Oryza sativa Indica               AGAAACGAGGAGCACGACTGAAGTGGCTATACATACAAATCCATTTACGG
Oryza sativa Japonica             AGAAACGAGGAGCACGACTGAAGTGGCTATACATACAAATCUATTTACGG
Tripsacum dactyloides             AGAAACGAGGAGCACGACTGAAGTGGCTATACATACAAATCCATTTACGG
Zea luxurians                     AGAAACGAGGAGCACGACTGAAGTGGCTATACATACAAATCCATTGACGG
Zea mays parviglumis              AGAAACGAGGAGCACGACTGAAGTGGCTATACATACAAATCCATTGACGG
Zea perennis                      AGAAACGAGGAGCACGACTGAAGTGGCTATACATACAAATCCATTGACGG
Sorghum bicolor                   AGAAACGAGGAGCACGACTGAAGTGGCTATACATACAAATCCATTTACGG
Cycas taitungensis                GGAAACGACGAGCGCGACTGAAGTTGCTATACATACAAATCTATTTACGG
                                   ****  * **** ** ******* ***** ***** **** *** ** *

Beta macrocarpa                   ATCTATATGCTTUGATTGGAACTGGAAGTTCAAGAACAGGCGGCTGGTAT
Beta vulgaris maritima            ATCTATATGCTTUGATTGGAACTGGAAGTTCAAGAACAGGCGGCTGGTAT
Silene latifolia                  ATCTATATGCTTUGATTGGAACTGGAAGTTCAAGAACAGGCGGCTGGTAT
PB235_ccmFc                       ATCTATATGCTTCGATTGGAACTGGAAGTTCCAGAACAGGCGGCTGGTAT
RRIT251_ccmFc                     ATCTATATGCTTCGATTGGAACTGGAAGTTCCAGAACAGGCGGCTGGTAT
RRIC110_ccmFc                     ATCTATATGCTTCGATTGGAACTGGAAGTTCCAGAACAGGCGGCTGGTAT
BPM24_ccmFc                       ATCTATATGCTTCGATTGGAACTGGAAGTTCCAGAACAGGCGGCTGGTAT
RRII105_ccmFc                     ATCTATATGCTTCGATTGGAACTGGAAGTTCCAGAACAGGCGGCTGGTAT
RRIM600_ccmFc                     ATCTATATGCTTCGATTGGAACTGGAAGTTCCAGAACAGGCGGCTGGTAT
Brassica napus                    ATCTATATGCTTUGATTGGAACTGGAAGTTCCAGAACAGGAGGTTGGTAT
Carica papaya                     ATCTATATGCTTCGATTGGAACTGGAAGTTCCAGAACAGGAGGCTGGTAT
Citrullus lanatus                 ATCTATATGCTTUGATTGGAACTGGAAGTTCCAGAACAGGCGGCTGGTAT
Cucurbita pepo                    ATUTATATGCTTUGATTGGAACTGGAAGTTCCAGAACAGGCGGCTGGTAT
Lotus japonicus                   ATAGATATGCTTUGATTGGAACTGGAAGTTCCAGAACAGGCGGCTGGTAT
Millettia pinnata                 ATAGATATGCTTUGATTGGAACTGGAAGTTCCAGAACAGGCGGCTGGTAT
Vigna radiata                     ATAGATATGCTTCGATTGGAACTGGAAGTTCCAGAACAGGCGGCTGGTAT
Oryza sativa Indica               ATCTATATGCTTCGATTGGAACTGGAAGTTCCAGAACTGGCGGCTGGTAT
Oryza sativa Japonica             ATCTATATGCTTUGATTGGAACTGGAAGTTCCAGAACTGGCGGCTGGTAT
Tripsacum dactyloides             ATCTATATGCTCCGATTGGAACTGGAAGTTCCAGAACTGGCGGCTGGTAT
Zea luxurians                     ATCTATATGCTCCGATTGGAACTGGAAGTTCCAGAACTGGCGGCTGGTAT
Zea mays parviglumis              ATCTATATGCTCCGATTGGAACTGGAAGTTCCAGAACTGGCGGCTGGTAT
Zea perennis                      ATCTATATGCTCCGATTGGAACTGGAAGTTCCAGAACTGGCGGCTGGTAT
Sorghum bicolor                   ATCTATATGCTCCGATTGGAACTGGAAGTTCCAGAACTGGCGGCTGGTAT
Cycas taitungensis                ATCCATATGCTCCGATTGGAACTGGAAGTTTCGGAACAGGCGGCTGGTAT
                                  **  *******  *****************   **** ** ** ******

Beta macrocarpa                   ACTACCATAATGAAACTGCCTTTTCTTTTTTTTTATTUGGATAGGATTTCT
Beta vulgaris maritima            ACTACCATAATGAAACTGCCTTTTCTTTTTTTTTATTUGGATAGGATTTCT
Silene latifolia                  ACTACTATAATGAAACTGCCTTTTATTTTTTTTTATTUGGATAGGATTTCT
PB235_ccmFc                       ACCACCATAATGAAACTGCCTTTTATTTTTTTTTATTCGGATAGGATTTAT
RRIT251_ccmFc                     ACCACCATAATGAAACTGCCTTTTATTTTTTTTTATTCGGATAGGATTTAT
RRIC110_ccmFc                     ACCACCATAATGAAACTGCCTTTTATTTTTTTTTATTCGGATAGGATTTAT
BPM24_ccmFc                       ACCACCATAATGAAACTGCCTTTTATTTTTTTTTATTCGGATAGGATTTAT
RRII105_ccmFc                     ACCACCATAATGAAACTGCCTTTTATTTTTTTTTATTCGGATAGGATTTAT
RRIM600_ccmFc                     ACCACCATAATGAAACTGCCTTTTATTTTTTTTTATTCGGATAGGATTTAT
Brassica napus                    ACCACCATAATGAAACTGCCTTTTATTTTTTTTTATTUGGATAGGATTTAT
Carica papaya                     ACCACCATAATGAAACTGCCTTTTCTTTTTTTGATTCGGATAGGATTTAT
Citrullus lanatus                 ACCACCATAATGAAACTGCCTTTTATTTTTTTTTATTCGGATAGGATTTCT
```

```
Cucurbita pepo                    ACCACCATAATGAAACTGCCTTTTCTTTTTTTGATTUGGATAGGATTTCT
Lotus japonicus                   ACCACCATAATGAAACTGCCTTTTATTTTTTTTATTUGGATAGGATTTAT
Millettia pinnata                 ACCACCATAATGAAACTGCCTTTTCTTTTTTTTATTUGGATAGGATTTAT
Vigna radiata                     ACCACCATAATGAAACTGCCTTTTCTTTTTTTTATTCGGATAGGATTTAT
Oryza sativa Indica               ACCACCATAATGAAATTGCCTTTTCTTTTTAGTATTCGGATAGGATTTCT
Oryza sativa Japonica             ACCACCATAATGAAATTGCCTTTTCTTTTTAGTATTUGGATAGGATTTCT
Tripsacum dactyloides             ACCACCATAATGAAATTGCCTTTTATTTTTAGTATTCGGATAGGATTTCT
Zea luxurians                     ACCACCATAATGAAATTGCCTTTTATTTTTAGTATTCGGATAGGATTTCT
Zea mays parviglumis              ACCACCATAATGAAATTGCCTTTTATTTTTAGTATTCGGATAGGATTTCT
Zea perennis                      ACCACCATAATGAAATTGCCTTTTATTTTTAGTATTCGGATAGGATTTCT
Sorghum bicolor                   ACCACCATAATGAAATTGCCTTTTATTTTTAGTATTCGGATAGGATTTCT
Cycas taitungensis                ACCACCATAATGGAACTGCCTCTTATTTTTCGCATTCGGATAGGATTTCT
                                  ** ** ****** ** ***** ** *****   *** *********** *

Beta macrocarpa                   GTTGGCTTCGCTGGGAGGCTCGCGTAGTTTGTTACGTCAACTCCAAAAGG
Beta vulgaris maritima            GTTGGCTTCGCTGGGAGGCTCGCGTAGTTTGTTACGTCAA----------
Silene latifolia                  GTTGGCTTCGTTGGGGGGCTCGCGTAGTTTGTTACGTCAACTCCAAAAGG
PB235_ccmFc                       GTTGGCTTCGTTAGGAGGCTUGCGTAGTTTGTTACGTCAGCTCCAAAAGG
RRIT251_ccmFc                     GTTGGCTTCGTTAGGAGGCTUGCGTAGTTTGTTACGTCAGCTCCAAAAGG
RRIC110_ccmFc                     GTTGGCTTCGTTAGGAGGCTUGCGTAGTTTGTTACGTCAGCTCCAAAAGG
BPM24_ccmFc                       GTTGGCTTCGTTAGGAGGCTUGCGTAGTTTGTTACGTCAGCTCCAAAAGG
RRII105_ccmFc                     GTTGGCTTCGTTAGGAGGCTUGCGTAGTTTGTTACGTCAGCTCCAAAAGG
RRIM600_ccmFc                     GTTGGCTTCGTTAGGAGGCTNGCGTAGTTTGTTACGTCAGCTCCAAAAGG
Brassica napus                    GTTGGCTTCGTTGGGAGGCTTGCCTAGTTTGTTACGTCAGCTCCAAAAGG
Carica papaya                     GTTGGCTTCGTTGGGAGGCTCGCGTAGTTTGTTACGTCAGCTCCAAAAGG
Citrullus lanatus                 GTTGGCTTCGTTGGGAGGCTCGCGTAGTTTGTTACGTCAGCTCCAAAAGG
Cucurbita pepo                    GTTGGCTTCGTTGGGAGGCTCGCGTAGTTTGTTACGTCAGCTACAAAAGG
Lotus japonicus                   GTTGGCTTCGTUGGGAGGCTCGCGTAGTTTGTTACGTCAGCTCCAAAAGG
Millettia pinnata                 GTTGGCTTCGTUGGGAGGCTCGCGTAGTTTGTTACGTCAGCTCCAAAAGG
Vigna radiata                     GTTGGCTTCGTCGGGAGGCTCGCGTAGTTTGTTACGTCAGCTCCAAAAGG
Oryza sativa Indica               GTTGGCTTCATCGGGGGGCTCGCGTAGTTTGTTACGTCAACTCCAAAAGG
Oryza sativa Japonica             GTTGGCTTCATUGGGGGGCTCGCGTAGTTTGTTACGTCAACTCCAAAAGG
Tripsacum dactyloides             GTTGGCTTCATCGGGAGGCTCGCGTAGTTTGTTACGTCAACTCCAAAAGG
Zea luxurians                     GTTGGCTTCATCGGGAGGCTCGCGTAGTTTGTTACGTCAACTCCAAAAGG
Zea mays parviglumis              GTTGGCTTCATCGGGAGGCTCGCGTAGTTTGTTACGTCAACTCCAAAAGG
Zea perennis                      GTTGGCTTCATCGGGAGGCTCGCGTAGTTTGTTACGTCAACTCCAAAAGG
Sorghum bicolor                   GTTGGCTTCATCGGGAGGCTCGCGTAGTTTGTTACGTCAACTCCAAAAGG
Cycas taitungensis                GTTGGCTTCGCCGGGGGGCTTGCGTAGTTTGTTACGTCAGCTCCAAAAGG
                                  *********    ** **** ** ***************

Beta macrocarpa                   AGAAATTGCGTTGGAATTGA------------------------------
Beta vulgaris maritima            --------------------------------------------------
Silene latifolia                  AGAAATTGCGTTGGAATTGA------------------------------
PB235_ccmFc                       ATAAGTTGCGTTGGCATUGAGAAAGTTCCGTGGAGTTTATAATTGCATAA
RRIT251_ccmFc                     ATAAGTTGCGTTGGCATUGAGAAAGTTCCGTGGAGTTTATAATTGCATAA
RRIC110_ccmFc                     ATAAGTTGCGTTGGCATUGAGAAAGTTCCGTGGAGTTTATAATTGCATAA
BPM24_ccmFc                       ATAAGTTGCGTTGGCATUGAGAAAGTTCCGTGGAGTTTATAATTGCATAA
RRII105_ccmFc                     ATAAGTTGCGTTGGCATUGAGAAAGTTCCGTGGAGTTTATAATTGCATAA
RRIM600_ccmFc                     ATAAGTTGCGTTGGCATNGAGAAAGTTCCGTGGAGTTTATAATTGCATAA
Brassica napus                    ATAAGTTGCGTTGGAATUGAGAAAGTTCCGTGGAGTTCATAATTGCATAA
Carica papaya                     ATAAGTTGCGTTGGAATUGA------------------------------
Citrullus lanatus                 ATAAGTTGCGTTGGAATTGA------------------------------
Cucurbita pepo                    ATAAGTTGCGTTGGAATTGA------------------------------
Lotus japonicus                   ATAAGTTGCGTTGGAATUGA------------------------------
Millettia pinnata                 ATAAGTTGCGTTGGAATUGA------------------------------
Vigna radiata                     ATGAGTTGCGTTGGAATUGA------------------------------
Oryza sativa Indica               ATAAGTTGCATTGGUAT---------------------------------
Oryza sativa Japonica             ATAAGTTGCATTGGAA----------------------------------
Tripsacum dactyloides             ATAAGTTGCATTGGAATUCA------------------------------
Zea luxurians                     ATAAGTTGCATTGGAATUCA------------------------------
Zea mays parviglumis              ATAAGTTGCATTGGAATCGA------------------------------
Zea perennis                      ATAAGTTGCATTGGAATUGA------------------------------
```

```
Sorghum bicolor                  ATAAGTTGCATTGGAATUGA------------------------------
Cycas taitungensis               ATAAGTTGCATTGGAATUGA------------------------------
```

###################

cob multiple sequence alignment

```
Arabidopsis thaliana             ---CAAAAGTGTGTAGGAGAAAGAATT--CTAAAAACGTCGACGCTTA--
Brassica napus                   GGGCAAAGGTGTGTAGGAGAAAGAATT--CTAAAAACGTCGACGCTTA--
PB235_cob                        --------------------------------------------------
Lotus japonicus                  ----------GTAACCCAGGATGACCC--CTTCGACGCTCGCTTCTTTCG
Millettia pinnata                --------CCGTAACCCAGG-----CC--CTTCGACGCTCGCTTCTTTCG
BPM24_cob                        --------------------------------------------------
Citrullus lanatus                --------GGAAAAACCAACGCAAATA--TCATATTGACTTTCTCTC---
Cucurbita pepo                   -------TGGAAAACCCAAGGCAAATA--TCATATAGACTTTCTCTC---
RRII105_cob                      --------------------------------------------------
Beta macrocarpa                  -------------------------------AGGTAATTCCTATTTTAAT
Beta vulgaris vulgaris           -------------------------------AGGTAATTCCTATTTTAAT
Beta vulgaris maritima           -------------------------------AGGTAATTCCTATTTTAAT
RRIT251_cob                      --------------------------------------------------
Silene latifolia                 ---------------------TCTCCG--ATATGCAAGGGGTGTCGTCTG
RRIM600_cob                      --------------------------------------------------
RRIC110_cob                      --------------------------------------------------
Oryza sativa Japonica            --------------TCGAGAATCAACTAACCGACAAATCCGTAGCCCAGG
Megaceros aenigmaticus           --------------------------------------------------


Arabidopsis thaliana             --ATTCGCCCCCTCCAT---CCTTCAAAAGTAAAAGAAAGGCTCAAATAT
Brassica napus                   --ATTCGCCCCCGAGGAATGCTTTCAAAAGTCAAAGAAAGGCTCAAATAT
PB235_cob                        --------------------------------------------------
Lotus japonicus                  CTGTATACCCCCTCCAT---CCTTCGGAGGT----GGAAGAAAGAAAAGG
Millettia pinnata                CTGTATACCCCCTCAAT---CCTTCGGAGGTAGGTGGAAGAAAGAAAAGG
BPM24_cob                        --------------------------------------------------
Citrullus lanatus                --GCCCTACTTCTCAGT---CTCGATAGAAAAGGTTGGAGAGAGTGACTT
Cucurbita pepo                   --GCCCTACTTCTCAGT---CTCGATAGAAAAGGTTGGAGAGAGTGACTT
RRII105_cob                      --------------------------------------------------
Beta macrocarpa                  ACTTGGAACAAGTAAGGGTCCAGATCATTCCATTGGGGAGAAAGCAGGAG
Beta vulgaris vulgaris           ACTTGGAACAAGTAAGGGTCCAGATCATTCCATTGGGGAGAAAGCAGGAG
Beta vulgaris maritima           ACTTGGAACAAGTAAGGGTCCAGATCATTCCATTGGGGAGAAAGCAGGAG
RRIT251_cob                      --------------------------------------------------
Silene latifolia                 GTATAGAACCAGAACTTGGGCGGAGGTAGTTGTTTTCCAGTAGCCAGAAC
RRIM600_cob                      --------------------------------------------------
RRIC110_cob                      --------------------------------------------------
Oryza sativa Japonica            TGATTCGCTGCCTCCCT---CTCGCCAAAATGGGATGAATCTTCTCATGC
Megaceros aenigmaticus           --------------------------------------------------


Arabidopsis thaliana             CAATATTATATATATATTTGAG----GATATTTTAGGGCCCT---AGAAC
Brassica napus                   CAA-----TATATATATTTTAG----GATATTTTAGGGCCCT---AGAAC
PB235_cob                        --------------------------------------------------
Lotus japonicus                  TACTAAAATATATATATATT----TTATTTTAGTAGGGCCCC---AGAAC
Millettia pinnata                TACTATAAATAATAAATAATGGATTCTTTTTTTTAGGGCCCC---AGAAC
BPM24_cob                        --------------------------------------------------
Citrullus lanatus                TATGAAATTCTCTCCTTTCTAAAGCAGCGCAAGGCGGCCCCCCCCAGAAC
Cucurbita pepo                   TATGAAATTCTCTCCTTTCTAAAGCAGCGAAAGGCGGCCCCCCC-AGAAC
RRII105_cob                      --------------------------------------------------
Beta macrocarpa                  TCATCCATATATAGATGAATAGTACCAGTGGCGTCTAGGAGGAAGACGGG
Beta vulgaris vulgaris           TCATCCATATATAGATGAATAGTACCAGTGGCGTCTAGGAGGAAGACGGG
Beta vulgaris maritima           TCATCCATATATAGATGAATAGTACCAGTGGCGTCTAGGAGGAAGACGGG
RRIT251_cob                      --------------------------------------------------
Silene latifolia                 T--TTCTTTCCTGGTTGTAGGGT-CTATAAACCCATAG-----AGAGGTG
RRIM600_cob                      --------------------------------------------------
RRIC110_cob                      --------------------------------------------------
```

```
Oryza sativa Japonica             AGCTT--TTTTCTTGTTCAGGGCGCAGCGAAGCCAATTTCCATCAAGGCA
Megaceros aenigmaticus            --------------------------------------------------


Arabidopsis thaliana              GCAAAAAAAAGGTGGGTGAACAAGAGTTGTCACGATAGGAAAGAGAAA--
Brassica napus                    GCAAAAAAAAG-TGGGTGAACAAGAGTTGTCACGATAGGAAAGAGAAA--
PB235_cob                         -----------------------------------------------A--
Lotus japonicus                   G-AAAAAAAAGGTGGGGGAACCAGAGTTGTCACGATAGGAAAGATAAAAA
Millettia pinnata                 G-AAAAAAA-GGTGGGGGAACCAGAGTTGTCACGATAGGAAAGAGAAAAA
BPM24_cob                         --------------------------------------------------
Citrullus lanatus                 GCTAAAAA--GGTGGGGGAACAAGAGTTGTCACGATAGGAAAGAGAAA--
Cucurbita pepo                    GCTAAAAA--GGTGGGGGAACAAGAGTTGTCACGATAGGAAAGAGAAA--
RRII105_cob                       -----------------------------------------------A--
Beta macrocarpa                   GAGTGACTGGGGTGGG-GAAGAAGAGTTGTCACGATAGAAAGAATCTTGG
Beta vulgaris vulgaris            GAGTGACTGGGGTGGG-GAAGAAGAGTTGTCACGATAGAAAGAATCTTGG
Beta vulgaris maritima            GAGTGACTGGGGTGGG-GAAGAAGAGTTGTCACGATAGAAAGAATCTTGG
RRIT251_cob                       --------------------------------------------------
Silene latifolia                  GTTTTTCTGGGGTGCG-GAAGAAGAATTGTAACGATAGAAAAAGTCTTCG
RRIM600_cob                       --------------------------------------------------
RRIC110_cob                       --------------------------------------------------
Oryza sativa Japonica             AGGGGGTAAATAAGGGGGAAGAGGAGTTGTCACGATAGAAAAGAGAAA--
Megaceros aenigmaticus            --------------------------------------------------


Arabidopsis thaliana              -----TGACTA------TAAGGAACCAACGATTCTCTCTTCTTAAACAAC
Brassica napus                    -----TGACTA------TAAGGAACCAUCGATTCTCTCTTCTTAAACAAC
PB235_cob                         -----TGACTA------TAAGGAACCAACGATTCTCTCTTCTTAAACAAC
Lotus japonicus                   ATAAATGACTAGCGGAATAAGGAACCAACGACTCTCTATTCTAAAACAAC
Millettia pinnata                 A---ATGACTA------TAAGGAACCAACGATTCTCTCTTCTTAAACAAC
BPM24_cob                         ----ATGACTA------TAAGGAACCAACGATTCTCTCTTCTTAAACAAC
Citrullus lanatus                 -----TGACTA------AAAGAAACCAACGATTCTCTCTTCTTAAACAAC
Cucurbita pepo                    -----TGACTA------AAAGAAACCAACGATTCTCTCTTCTTAAACAAC
RRII105_cob                       -----TGACTA------TAAGGAACCAACGATTCTCTCTTCTTAAACAAC
Beta macrocarpa                   CAAAGCAAATGA--GTCTAAGAAACCAACGATTCTCTGTTCTTAAACAAC
Beta vulgaris vulgaris            CAAAGCAAATGA--GTCTAAGAAACCAACGATTCTCTGTTCTTAAACAAC
Beta vulgaris maritima            CAAAGCAAATGA--GTCTAAGAAACCAACGATTCTCTGTTCTTAAACAAC
RRIT251_cob                       --------ATGA--CTATAAGGAACCAACGATTCTCTCTTCTTAAACAAC
Silene latifolia                  CAAAGCAAATGA--GTATAAGGAACCAACGATTCTCTGTTCTTAAACAAC
RRIM600_cob                       --------ATGA--CTATAAGGAACCAACGATTCTCTCTTCTTAAACAAC
RRIC110_cob                       ----ATGACTA------TAAGGAACCAACGATTCTCTCTTCTTAAACAAC
Oryza sativa Japonica             -----TGACTA------TAAGGAACCAACGATTCTCTCTTCTTAAACAAC
Megaceros aenigmaticus            -------------------AUGGCAAGACGACTTTCGATTCTTAAACAAC
                                                     *        *** * **  **** *******

Arabidopsis thaliana              CTATATCCTCCACACTTAATCAGCATTTAGTAGATTATCCAACCCCGAGC
Brassica napus                    CTATATCCTCCACACTTAATCAGCATTTAGTAGATTATCCAACCCCGAGC
PB235_cob                         CTATATCCTCCACACTTAATCAGCATTTGATAGATTATCCAACCCCGAGC
Lotus japonicus                   CTATATCCTCCACACTTAATCAACATTTGATAGATTATCCAACCCCGAGC
Millettia pinnata                 CTATATCCTCCACACTTAATCAACATTTGATAGATTATCCAACCCCGAGC
BPM24_cob                         CTATATCCTCCACACTTAATCAGCATTTGATAGATTATCCAACCCCGAGC
Citrullus lanatus                 CTATATCCTCCACACTTAATCAGCATTTGATAGATTATCCAACCCCGAGC
Cucurbita pepo                    CTATATCCTCCACACTGAATCAGCATTTGATAGATTATCCAACCCCGAGT
RRII105_cob                       CTATATCCTCCACACTTAATCAGCATTTGATAGATTATCCAACCCCGAGC
Beta macrocarpa                   CUATATTTTCCACACTTAATCAGCATTTGATAGATTATCCAACCCCGAGC
Beta vulgaris vulgaris            CUATATTTTCCACACTTAATCAGCATTTGATAGATTATCCAACCCCGAGC
Beta vulgaris maritima            CUATATTTTCCACACTTAATCAGCATTTGATAGATTATCCAACCCCGAGC
RRIT251_cob                       CTATATCCTCCACACTTAATCAGCATTTGATAGATTATCCAACCCCGAGC
Silene latifolia                  CCATATTTTCCACACTTAATCAGCATTTGATAGATTATCCAACCCCGAGC
RRIM600_cob                       CTATATCCTCCACACTTAATCAGCATTTGATAGATTATCCAACCCCGAGC
RRIC110_cob                       CTATATCCTCCACACTTAATCAGCATTTGATAGATTATCCAACCCCGAGC
Oryza sativa Japonica             CTATATACTCCACACTTAACCAGCATTTGATAGATTATCCAACTCCGAGC
Megaceros aenigmaticus            CTATATTTTCTACAGTTAACAATCATTCGATAGACTATCCAACTCTAAGC
```

```
                                 * ****  ** *** * **  * ****   **** ******** *  **

Arabidopsis thaliana             AATCTTAGTTATTGGTGGGGGTTCGGTCCGTTAGCTGGTATTTGTTTAGT
Brassica napus                   AATCTTAGTTATTGGTGGGGGTTCGGTCCGTTAGCTGGTATTTGTTTAGT
PB235_cob                        AATCTTAGTTATTGGTGGGGGTTCGGTUCGTTAGCTGGTATTTGTTTAGT
Lotus japonicus                  AATCTTAGTTATTGGTGGGGCTTCGGTTCGTTAGCTGGTATTTGTTTAGT
Millettia pinnata                AATCTTAGTTATTGGTGGGGCTTCGGTTCGTTAGCTGGTATTTGTTTAGT
BPM24_cob                        AATCTTAGTTATTGGTGGGGGTTCGGTUCGTTAGCTGGTATTTGTTTAGT
Citrullus lanatus                AATCTTAGTTATTGGTGGGGGTTCGGTUCGTTAGCAGGTCTTTGTTTAGT
Cucurbita pepo                   AATCTTAGTTATTGGTGGGGGTTCGGTUCGTTAGCAGGTCTTTGTTTAGT
RRII105_cob                      AATCTTAGTTATTGGTGGGGGTTCGGTUCGTTAGCTGGTATTTGTTTAGT
Beta macrocarpa                  AATATTAGTTATTGGTGGGGATTCGGTTCGTTAGCTGGGATTTGTTTAGT
Beta vulgaris vulgaris           AATATTAGTTATTGGTGGGGATTCGGTTCGTTAGCTGGGATTTGTTTAGT
Beta vulgaris maritima           AATATTAGTTATTGGTGGGGATTCGGTTCGTTAGCTGGGATTTGTTTAGT
RRIT251_cob                      AATCTTAGTTATTGGTGGGGGTTCGGTUCGTTAGCTGGTATTTGTTTAGT
Silene latifolia                 AATATTAGTTATTGGTGGGGATTCGGTTCGTTAGCTGGGATTTGTTTAGT
RRIM600_cob                      AATCTTAGTTATTGGTGGGGGTTCGGTNCGTTAGCTGGTATTTGTTTAGT
RRIC110_cob                      AATCTTAGTTATTGGTGGGGGTTCGGTUCGTTAGCTGGTATTTGTTTAGT
Oryza sativa Japonica            AATCTTAGTTATTGGTGGGGGTTCGGTTCGTTAGCAGGTATTTGTTTAGT
Megaceros aenigmaticus           AATTTAAGTTATTGGTGGGGTTTTGGTTCGTTGGCTGCTATTCGTTTGGT
                                 *** * ************** ** *** **** ** *   ** **** **

Arabidopsis thaliana             CATTCAGATAGTGACTGGCGTTTTTTTAGCTATGCATTACACACCTCATG
Brassica napus                   CATTCAGATAGTGACTGGCGTTTTTTTAGCTATGCATTACACACCTCATG
PB235_cob                        CATTCAGATAGTGACTGGCGTTTTTTTAGCTATGCATUACACACCTCATG
Lotus japonicus                  CATTCAGATAGTGACTGGCGTTTTTTTAGCTATGCATTACACACCTCATG
Millettia pinnata                CATTCAGATAGTGACTGGCGTTTTTTTAGCTATGCATTACACACCTCATG
BPM24_cob                        CATTCAGATAGTGACTGGCGTTTTTTTAGCTATGCATUACACACCTCATG
Citrullus lanatus                CATTCAGATAGTTACTGGCGTTTTTTTAGCTATGCATTACACACCTCATG
Cucurbita pepo                   CATTCAGATAGTTACTGGCGTTTTTTTAGCTATGCATTACACCCCTCATG
RRII105_cob                      CATTCAGATAGTGACTGGCGTTTTTTTAGCTATGCATUACACACCTCATG
Beta macrocarpa                  CATTCAGATAGTGACTGGCGTTTTTTTAGCTATGCATTATACACCTCATG
Beta vulgaris vulgaris           CATTCAGATAGTGACTGGCGTTTTTTTAGCTATGCATTATACACCTCATG
Beta vulgaris maritima           CATTCAGATAGTGACTGGCGTTTTTTTAGCTATGCATTATACACCTCATG
RRIT251_cob                      CATTCAGATAGTGACTGGCGTTTTTTTAGCTATGCATUACACACCTCATG
Silene latifolia                 CATTCAGATAGTGACTGGCGTTTTTTTAGCTATGCATTATACACCTCATG
RRIM600_cob                      CATTCAGATAGTGACTGGCGTTTTTTTAGCTATGCATNACACACCTCATG
RRIC110_cob                      CATTCAGATAGTGACTGGCGTTTTTTTAGCTATGCATUACACACCTCATG
Oryza sativa Japonica            CATTCAGATAGTGACTGGCGTTTTTTTAGCTATGCATUACACACCTCATG
Megaceros aenigmaticus           TACTCAGATAATTACAGGTGTTTCCTTAGCTATGCATCATACACCTCATG
                                  * ******* * ** ** ****  ************ * ** *******

Arabidopsis thaliana             TGGATTTAGCTTTCAACAGCGTAGAACACATTATGAGAGATGTTGAAGGG
Brassica napus                   TGGATTTAGCTTTCAACAGCGTAGAACACATTATGAGAGATGTTGAAGGG
PB235_cob                        TGGATCTAGCTTTCAACAGCGTAGAACACATTATGAGAGATGTTGAAGGG
Lotus japonicus                  TGGATCTAGCTTTCAACAGCGTAGAACACGTTATGAGAGATGTTGAAGGG
Millettia pinnata                TGGATCTAGCTTTCAACAGCGTAGAACACGTTATGAGAGATGTTGAAGGG
BPM24_cob                        TGGATCTAGCTTTCAACAGCGTAGAACACATTATGAGAGATGTTGAAGGG
Citrullus lanatus                TGGATCTAGCTTTCAACAGCGTAGAACACATTATGAGAGATGTTGAAGGG
Cucurbita pepo                   TGGATCTAGCTTTCAACAGCGTAGAACACGTTATGAGAGATGTTGAAGGG
RRII105_cob                      TGGATCTAGCTTTCAACAGCGTAGAACACATTATGAGAGATGTTGAAGGG
Beta macrocarpa                  TGGATCTAGCTTTCAACAGCGTAGAACACATTATGAGAGATGTTGAAGGG
Beta vulgaris vulgaris           TGGATCTAGCTTTCAACAGCGTAGAACACATTATGAGAGATGTTGAAGGG
Beta vulgaris maritima           TGGATCTAGCTTTCAACAGCGTAGAACACATTATGAGAGATGTTGAAGGG
RRIT251_cob                      TGGATCTAGCTTTCAACAGCGTAGAACACATTATGAGAGATGTTGAAGGG
Silene latifolia                 TGGATCTAGCTTTCAACAGCGTAGAACACATTATGAGAGATGTTGAAGGG
RRIM600_cob                      TGGATCTAGCTTTCAACAGCGTAGAACACATTATGAGAGATGTTGAAGGG
RRIC110_cob                      TGGATCTAGCTTTCAACAGCGTAGAACACATTATGAGAGATGTTGAAGGG
Oryza sativa Japonica            TGGATCTAGCTTTCAACAGCGTAGAACACATTATGAGAGATGTTGAAGGG
Megaceros aenigmaticus           TGGATCTAGCTTTCTTAAGTGTAGAACACATAATGAGAGATGTGAAAGGA
                                 ***** ********   ** ********* * ***********  ****
```

```
Arabidopsis thaliana          GGCTGGTTGCTCCGTTATATGCATGCTAATGGGGCAAGTATGTTTUTTAT
Brassica napus                GGCTGGTTGCTCCGTTATATGCATGCTAATGGGGCAAGTATGTTTUTTAT
PB235_cob                     GGCTGGTTGCTCCGTTATATGCATGCTAATGGGGCAAGTATGTTTUTCAT
Lotus japonicus               GGCTGGTTGCTCCGTTATATGCATGCTAATGGGGCAAGTATGTTTUTCAT
Millettia pinnata             GGCTGGTTGCTCCGTTATATGCATGCTAATGGGGCAAGTATGTTTUTCAT
BPM24_cob                     GGCTGGTTGCTCCGTTATATGCATGCTAATGGGGCAAGTATGTTTUTCAT
Citrullus lanatus             GGCTGGTTGCTCCGTTATATGCATGCTAATGGGGCAAGTATGTTTUTCAT
Cucurbita pepo                GGCTGGTTGCTCCGTTATATGCATGCGAATGGGGCTAGTATGTTTUTCAT
RRII105_cob                   GGCTGGTTGCTCCGTTATATGCATGCTAATGGGGCAAGTATGTTTUTCAT
Beta macrocarpa               GGCTGGTTGCTCCGTTATATGCATGCTAATGGGGCAAGTATGTTTUTCAT
Beta vulgaris vulgaris        GGCTGGTTGCTCCGTTATATGCATGCTAATGGGGCAAGTATGTTTUTCAT
Beta vulgaris maritima        GGCTGGTTGCTCCGTTATATGCATGCTAATGGGGCAAGTATGTTTUTCAT
RRIT251_cob                   GGCTGGTTGCTCCGTTATATGCATGCTAATGGGGCAAGTATGTTTUTCAT
Silene latifolia              GGCTGGTTGCTCCGTTATATGCATGCTAATGGGGCAAGTATGTTTUTCAT
RRIM600_cob                   GGCTGGTTGCTCCGTTATATGCATGCTAATGGGGCAAGTATGTTTUTCAT
RRIC110_cob                   GGCTGGTTGCTCCGTTATATGCATGCTAATGGGGCAAGTATGTTTUTCAT
Oryza sativa Japonica         GGCTGGTTGCTCCGTTATATGCATGCTAATGGGGCAAGTATGTTTUTCAT
Megaceros aenigmaticus        GGCTGGTTACTTCGTTACATGCATGCTAATGGTGCCAGTATGTTCTTTAT
                              ******** ** ***** ******** ***** ** ********  * **

Arabidopsis thaliana          TGTGGTTTACCTTCATATTTTTCGTGGTCTATATUATGCGAGTTATAGCA
Brassica napus                TGTGGTTTACCTTCATATTTTTCGTGGTCTATATUATGCGAGTTATAGCA
PB235_cob                     TGTGGTTCACCTTCATATTTTTCGCGGTCTATATUATGCGAGTTATAGCA
Lotus japonicus               TGTGGTTUACCTTCATATTTTTCGTGGTCTATATUATGCGAGTTATAGCA
Millettia pinnata             TGTGGTTUACCTTCATATTTTTCGTGGTCTATATUATGCGAGTTATAGCA
BPM24_cob                     TGTGGTTCACCTTCATATTTTTCGCGGTCTATATUATGCGAGTTATAGCA
Citrullus lanatus             TGTGGTTUACCTTCATATTTTTCGTGGTCTATATUATGCGAGTTATAGCA
Cucurbita pepo                TGTGGTTUACCTTCATATTTTTCGTGGTCTATATUATGCGAGTTATAGCA
RRII105_cob                   TGTGGTTCACCTTCATATTTTTCGCGGTCTATATNATGCGAGTTATAGCA
Beta macrocarpa               TGTGGTTUACCTTCATATTTTTCGTGGTCTATATUATGCGAGTTATAGCA
Beta vulgaris vulgaris        TGTGGTTUACCTTCATATTTTTCGTGGTCTATATUATGCGAGTTATAGCA
Beta vulgaris maritima        TGTGGTTUACCTTCATATTTTTCGTGGTCTATATUATGCGAGTTATAGCA
RRIT251_cob                   TGTGGTTCACCTTCATATTTTTCGCGGTCTATATUATGCGAGTTATAGCA
Silene latifolia              TGTGGTTUACCTTCATATTTTTCGTGGTCTATATUATGCGAGTTATAGCA
RRIM600_cob                   TGTGGTTCACCTTCATATTTTTCGCGGTCTATATNATGCGAGTTATAGCA
RRIC110_cob                   TGTGGTTCACCTTCATATTTTTCGCGGTCTATATNATGCGAGTTATAGCA
Oryza sativa Japonica         TGTGGTTUACCTTCATATTTTTCGTGGTCTATATUATGCGAGTTATAGCA
Megaceros aenigmaticus        TACGGTTTATCTTCATATTCTTCGTGGTTTATATTATGGAAGTTATGCGA
                              *  **** * ********* **** *** ***** ***  ******   *

Arabidopsis thaliana          GTCCTAGGGAATTTGTTTGGTGTCTTGGAGTTGTAATCTTCCTATTAATG
Brassica napus                GTCCTAGGGAATTTGTTTGGTGTCTTGGAGTTGTAATCTTCCTATTAATG
PB235_cob                     GTCCTAGGGAATTTGTTUGGTGTCTCGGAGTTGTAATATTCCTATTAATG
Lotus japonicus               GTCCTAGGGAATTTGTTUGGTGTCTCGGAGTTGTAATCTTCCTATTAATG
Millettia pinnata             GTCCTAGGGAATTTGTTUGGTGTCTCGGAGTTGTAATCTTCCTATTAATG
BPM24_cob                     GTCCTAGGGAATTTGTTUGGTGTCTCGGAGTTGTAATATTCCTATTAATG
Citrullus lanatus             GTCCTAGGGAATTTGTTUGGTGTCTCGGAGTTGTAATCTTCCTATTAATG
Cucurbita pepo                GTCCTAGGGAATTTGTTUGGTGTCTCGGAGTTGTAATCTTCCTATTAATG
RRII105_cob                   GTCCTAGGGAATTTGTTUGGTGTCTCGGAGTTGTAATATTCCTATTAATG
Beta macrocarpa               GCCCTAGGGAATTTGTTUGGTGTCTCGGAGTTGTAATCTTCTTATTAATG
Beta vulgaris vulgaris        GCCCTAGGGAATTTGTTUGGTGTCTCGGAGTTGTAATCTTCTTATTAATG
Beta vulgaris maritima        GCCCTAGGGAATTTGTTUGGTGTCTCGGAGTTGTAATCTTCTTATTAATG
RRIT251_cob                   GTCCTAGGGAATTTGTTUGGTGTCTCGGAGTTGTAATATTCCTATTAATG
Silene latifolia              GTCCTAGGGAATTTGTTUGGTGTCTCGGGGTTGTAATCTTCTTATTAATG
RRIM600_cob                   GTCCTAGGGAATTTGTTNGGTGTCTCGGAGTTGTAATATTCCTATTAATG
RRIC110_cob                   GTCCTAGGGAATTTGTTUGGTGTCTCGGAGTTGTAATATTCCTATTAATG
Oryza sativa Japonica         GTCCTAGGGAATTTGTTTGGTGTCTCGGAGTTGTCATATTCCTATTAATG
Megaceros aenigmaticus        GTCCTAGGGAATTAGTTTGGTGTCTCGGAGTGGTCATTTTATTATTAATG
                              * *********** *** ******* ** ** ** ** **  ********

Arabidopsis thaliana          ATTGTGACAGCTTTTATAGGATATGTACTACCTTGGGGTCAGATGAGCTT
Brassica napus                ATTGTGACAGCTTTTATAGGATATGTACTACCTTGGGGTCAGATGAGCTT
```

```
PB235_cob                         ATTGTGACAGCTTTTATAGGATACGTACTACCTTGGGGTCAGATGAGCTT
Lotus japonicus                   ATTGTGACAGCTTTTAUAGGATACGTACUACCTTGGGGTCAGATGAGCTT
Millettia pinnata                 ATTGTGACAGCTTTTAUAGGATACGTACTACCTTGGGGTCAGATGAGCTT
BPM24_cob                         ATTGTGACAGCTTTTATAGGATACGTACTACCTTGGGGTCAGATGAGCTT
Citrullus lanatus                 ATTGTGACAGCTTTTAUAGGATACGTACTACCTTGGGGTCAGATGAGCTT
Cucurbita pepo                    ATTGTGACAGCTTTTAUAGGATACGTACTACCTTGGGGTCAGATGAGCTT
RRII105_cob                       ATTGTGACAGCTTTTATAGGATACGTACTACCTTGGGGTCAGATGAGCTT
Beta macrocarpa                   ATTGTGACAGCTTTTATAGGATAUGTACTACCTTGGGGTCAGATGAGCTT
Beta vulgaris vulgaris            ATTGTGACAGCTTTTATAGGATAUGTACTACCTTGGGGTCAGATGAGCTT
Beta vulgaris maritima            ATTGTGACAGCTTTTATAGGATAUGTACTACCTTGGGGTCAGATGAGCTT
RRIT251_cob                       ATTGTGACAGCTTTTATAGGATACGTACTACCTTGGGGTCAGATGAGCTT
Silene latifolia                  ATTGTGACAGCTTTTATAGGATAUGTACTACCTTGGGGTCAGATGAGCTT
RRIM600_cob                       ATTGTGACAGCTTTTATAGGATACGTACTACCTTGGGGTCAGATGAGCTT
RRIC110_cob                       ATTGTGACAGCTTTTATAGGATACGTACTACCTTGGGGTCAGATGAGCTT
Oryza sativa Japonica             ATTGTGACAGCTTTTATAGGATACGTACUACCTTGGGGTCAGATGAGCTT
Megaceros aenigmaticus            ATTATAACAGCTTTTATAGGCTACGTACTACCTCGGGGTCAAATGAGCTT
                                  *** * ********** *** ** **** **** ******* ********

Arabidopsis thaliana              TTGGGGAGCTACAGTAATTACAAGCTTAGCTAGCGCCATACCTGTAGTAG
Brassica napus                    TTGGGGAGCTACAGTAATTACAAGCTTAGCTAGCGCCATACCTGTAGTAG
PB235_cob                         TTGGGGAGCTACAGTAATTACAAGCTTAGCTAGCGCCATACCTGTAGTAG
Lotus japonicus                   TTGGGGAGCTACAGTAATTACAAGCTTAGCTAGCGCCATACCTGTAGTAG
Millettia pinnata                 TTGGGGAGCTACAGTAATTACAAGCTTAGCTAGCGCCATACCTGTAGTAG
BPM24_cob                         TTGGGGAGCTACAGTAATTACAAGCTTAGCTAGCGCCATACCTGTAGTAG
Citrullus lanatus                 TTGGGGAGCAACTGTAATTACAAGCTTAGCTAGCGCCATACCCGTAGTAG
Cucurbita pepo                    TTGGGGAGCAACTGTAATTACAAGCTTAGCTAGCGCCATACCCGTAGTAG
RRII105_cob                       TTGGGGAGCTACAGTAATTACAAGCTTAGCTAGCGCCATACCTGTAGTAG
Beta macrocarpa                   TTGGGGAGCTACAGTAATTACAAGCTTAGCTAGCGCTATACCTGTAGTAG
Beta vulgaris vulgaris            TTGGGGAGCTACAGTAATTACAAGCTTAGCTAGCGCTATACCTGTAGTAG
Beta vulgaris maritima            TTGGGGAGCTACAGTAATTACAAGCTTAGCTAGCGCTATACCTGTAGTAG
RRIT251_cob                       TTGGGGAGCTACAGTAATTACAAGCTTAGCTAGCGCCATACCTGTAGTAG
Silene latifolia                  TTGGGGAGCTACAGTAATTACAAGCTTAGCTAGCGCTATACCTGTAGTAG
RRIM600_cob                       TTGGGGAGCTACAGTAATTACAAGCTTAGCTAGCGCCATACCTGTAGTAG
RRIC110_cob                       TTGGGGAGCTACAGTAATTACAAGCTTAGCTAGCGCCATACCTGTAGTAG
Oryza sativa Japonica             TTGGGGAGCAACAGTAATTACAAGCTTAGCTAGCGCCATACCAGTAGTAG
Megaceros aenigmaticus            TTGGGGAGCAACGGTAATTACAAGCTTAGCTAGCGCCATACCTGTAGTAG
                                  ********* ** *********************** ***** *******

Arabidopsis thaliana              GAGATACCATAGTGACTTGGCTTTGGGGTGGTTTCTCCGTGGACAATGCC
Brassica napus                    GAGATACCATAGTGACTTGGCTTTGGGGTGGTTTCTCCGTGGACAATGCC
PB235_cob                         GAGATACCATAGTGACTTGGCTTTGGGGTGGTTTCTCCGTGGACAATGCC
Lotus japonicus                   GAGATACCATAGTGACTTGGCTTTGGGGTGGTTTCTCCGTGGACAATGCC
Millettia pinnata                 GAGATACCATAGTGACTTGGCTTTGGGGTGGTTTCTCCGTGGACAATGCC
BPM24_cob                         GAGATACCATAGTGACTTGGCTTTGGGGTGGTTTCTCCGTGGACAATGCC
Citrullus lanatus                 GAGATACCATAGTGACTTGGCTTTGGGGTGGTTTCTCCGTGGACAATGCC
Cucurbita pepo                    GAGATACCATAGTGACTTGGCTTTGGGGTGGTTTCTCCGTGGACAATGCC
RRII105_cob                       GAGATACCATAGTGACTTGGCTTTGGGGTGGTTTCTCCGTGGACAATGCC
Beta macrocarpa                   GAGATAGCATAGTGACTTGGCTTTGGGGCGGTTTCTCCGTGGACAATGCC
Beta vulgaris vulgaris            GAGATAGCATAGTGACTTGGCTTTGGGGCGGTTTCTCCGTGGACAATGCC
Beta vulgaris maritima            GAGATAGCATAGTGACTTGGCTTTGGGGCGGTTTCTCCGTGGACAATGCC
RRIT251_cob                       GAGATACCATAGTGACTTGGCTTTGGGGTGGTTTCTCCGTGGACAATGCC
Silene latifolia                  GAGATAGCATAGTGACTTGGCTTTGGGGTGGTTTCTCCGTGGACAATGCC
RRIM600_cob                       GAGATACCATAGTGACTTGGCTTTGGGGTGGTTTCTCCGTGGACAATGCC
RRIC110_cob                       GAGATACCATAGTGACTTGGCTTTGGGGTGGTTTCTCCGTGGACAATGCC
Oryza sativa Japonica             GAGATACCATAGTGACTTGGCTTTGGGGTGGTTTCTCCGTGGACAATGCC
Megaceros aenigmaticus            GAGACACTATAGTAACTCGGCTTTGGGGTGGCTTTCCCGTAGACAATGCT
                                  **** *  ***** *** ********** ** **  **** ********

Arabidopsis thaliana              ACCTTAAATCGTTTTTTTAGTCTTCATUATTTACTCCCCTTTATTTTAGT
Brassica napus                    ACCTTAAATCGTTTTTTTAGTCTTCATUATTTACTCCCCTTTATTTTAGT
PB235_cob                         ACCTTAAATCGTTTTTTTAGTCTTCATUATTTACTCCCCTTTATTTTAGT
Lotus japonicus                   ACCTTAAATCGTTTTTTTAGTCTTCATUATTTACTCCCCTTTATTTTAGT
```

```
Millettia pinnata               ACCTTAAATCGTTTTTTTAGTCTTCATUATTTACTCCCCTTTATTTTAGT
BPM24_cob                       ACCTTAAATCGTTTTTTTAGTCTTCATUATTTACTCCCCTTTATTTTAGT
Citrullus lanatus               ACCTTAAATCGTTTTTTTAGTCTTCATUATTTACTCCCTTTTCTTTTAGC
Cucurbita pepo                  ACCTTAAATCGTTTTTTTAGTCTTCATUATTTACTCCCTTTTCTTTTAGC
RRII105_cob                     ACCTTAAATCGTTTTTTTAGTCTTCATUATTTACTCCCCTTTATTTTAGT
Beta macrocarpa                 ACCTTAAATCGTTTTTTTAGTCTTCATUATTTACTCCCTTTTATTTTAGT
Beta vulgaris vulgaris          ACCTTAAATCGTTTTTTTAGTCTTCATUATTTACTCCCTTTTATTTTAGT
Beta vulgaris maritima          ACCTTAAATCGTTTTTTTAGTCTTCATUATTTACTCCCTTTTATTTTAGT
RRIT251_cob                     ACCTTAAATCGTTTTTTTAGTCTTCATUATTTACTCCCCTTTATTTTAGT
Silene latifolia                ACCTTAAATCGTTTTTTTAGTCTTCATUATTTACTCCCTTTTCTTTTAGT
RRIM600_cob                     ACCTTAAATCGTTTTTTTAGTCTTCATNATTTACTCCCCTTTATTTTAGT
RRIC110_cob                     ACCTTAAATCGTTTTTTTAGTCTTCATUATTTACTCCCCTTTATTTTAGT
Oryza sativa Japonica           ACCTTAAATCGTTTTTTTAGTCTUCATUATTTACTCCCCUTTATTTTAGT
Megaceros aenigmaticus          ACCTTAAATCGTTTTTCTAGCCTTCACCACTTACCTCCTTTTCTCATTGC
                                **************** *** ** **  * ****  **  ** *  * *

Arabidopsis thaliana            AGGCGCCAGTCTTCTTCATCTGGCCGCATTGCATCAATATGGATCAAATA
Brassica napus                  AGGCGCCAGTCTTCTTCATCTGGCCGCATTGCATCAATATGGATCAAATA
PB235_cob                       AGGCGCCAGTCTTCTTCATCTGGCTGCATTGCATCAATATGGATCAAATA
Lotus japonicus                 AGGCGCCAGTCTTCTTCATCTGGCCGCATTGCATCAATATGGATCAAATA
Millettia pinnata               AGGCGCCAGTCTTCTTCATCTGGCCGCATTGCATCAATATGGATCAAATA
BPM24_cob                       AGGCGCCAGTCTTCTTCATCTGGCTGCATTGCATCAATATGGATCAAATA
Citrullus lanatus               AGGCGCCAGTCTTCTTCATCTGGCCGCATTGCATCAATATGGATCAAATA
Cucurbita pepo                  AGGCGCCAGTCTTCTTCATCTGGCCGCATTGCATCAATATGGATCCAATA
RRII105_cob                     AGGCGCCAGTCTTCTTCATCTGGCTGCATTGCATCAATATGGATCAAATA
Beta macrocarpa                 AGGCGCCAGTCTTCTTCATCTGGCCGCATTGCATCAATATGGATCAAATA
Beta vulgaris vulgaris          AGGCGCCAGTCTTCTTCATCTGGCCGCATTGCATCAATATGGATCAAATA
Beta vulgaris maritima          AGGCGCCAGTCTTCTTCATCTGGCCGCATTGCATCAATATGGATCAAATA
RRIT251_cob                     AGGCGCCAGTCTTCTTCATCTGGCTGCATTGCATCAATATGGATCAAATA
Silene latifolia                AGGCGCCAGTCTTCTTCATCTGGCCGCATTGCATCAATATGGATCAAATA
RRIM600_cob                     AGGCGCCAGTCTTCTTCATCTGGCTGCATTGCATCAATATGGATCAAATA
RRIC110_cob                     AGGCGCCAGTCTTCTTCATCTGGCTGCATTGCATCAATATGGATCAAATA
Oryza sativa Japonica           AGGCGCCAGTCTTCTTCATCTGGCTGCATTGCATCAATATGGATCAAATA
Megaceros aenigmaticus          AGGTGTTAGTATTCTTCATCCGGCTGCATTACATCAGTATGGTTCCAATA
                                *** *  *** ********* *** ***** ***** ***** ** ****

Arabidopsis thaliana            ATCCATTGGGTGTACATTCTGAGATGGATAAAATAGCTTTTTACCCTTAT
Brassica napus                  ATCCATTGGGTGTACATTCTGAGATGGATAAAATAGCTTTTTACCCTTAT
PB235_cob                       ATCCATTGGGTGTACATTCAGAGATGGATAAAATTGCTTTTTACCCTTAT
Lotus japonicus                 ATCCATTGGGTGTACATTCAGAGATGGATCAAATTTCTTTTTACCCTTAT
Millettia pinnata               ATCCATTGGGTGTACATTCAGAGATGGATCAAATTTATTTTTACCCTTAT
BPM24_cob                       ATCCATTGGGTGTACATTCAGAGATGGATAAAATTGCTTTTTACCCTTAT
Citrullus lanatus               ATCCATTGGGTGTACATTCAGAGATGGATAAAATTTCTTTTTACCCTTAT
Cucurbita pepo                  ATCCATTGGGTGTACATTCTGAGATGGATAAAATCTCTTTTTACCCTTAT
RRII105_cob                     ATCCATTGGGTGTACATTCAGAGATGGATAAAATTGCTTTTTACCCTTAT
Beta macrocarpa                 ATCCATTAGGTGTACATTCAGAGATGGATAAAATTGCCUTTTACCCTTAT
Beta vulgaris vulgaris          ATCCATTAGGTGTACATTCAGAGATGGATAAAATTGCCUTTTACCCTTAT
Beta vulgaris maritima          ATCCATTAGGTGTACATTCAGAGATGGATAAAATTGCCUTTTACCCTTAT
RRIT251_cob                     ATCCATTGGGTGTACATTCAGAGATGGATAAAATTGCTTTTTACCCTTAT
Silene latifolia                ATCCATTAGGTGTACATTCAGAGATGGATAAAATTTCTTTTTACCCTTAT
RRIM600_cob                     ATCCATTGGGTGTACATTCAGAGATGGATAAAATTGCTTTTTACCCTTAT
RRIC110_cob                     ATCCATTGGGTGTACATTCAGAGATGGATAAAATTGCTTTTTACCCTTAT
Oryza sativa Japonica           ATCCATTGGGTGTACATTCTGAGATGGATAAAATAGCTTUTTACCCTTAT
Megaceros aenigmaticus          ATCCATTGGGTATCAATTCTTCTGTAGATAAAATAGCTTCGTATCTCTAT
                                ******* *** *  ****     * *** ****       ** *  ***

Arabidopsis thaliana            TTTTATGTCAAGGATCTAGTTGGTTGGGTAGCTTTTGCTATCTTTTTTTC
Brassica napus                  TTTTATGTCAAGGATCTAGTTGGTTGGGTAGCTTTTGCTATCTTTTTTTC
PB235_cob                       TTTTATGTAAAGGATCTAGTAGGTTGGGTAGCTTTTGCTATCTTTTTTTC
Lotus japonicus                 TTTTATGTAAAGGATCTAGTAGGTTGGGTAGCTTTTGCTATCTTTTTTTC
Millettia pinnata               TTTTATGTAAAGGATCTAGTAGGTTGGGTAGCTTTTGCTATCTTTTTTTC
BPM24_cob                       TTTTATGTAAAGGATCTAGTAGGTTGGGTAGCTTTTGCTATCTTTTTTTC
```

```
Citrullus lanatus              TTTTATGTAAAGGATCTAGTGGGTTGGGTAGCTTTTGCTATCTTTTTTTC
Cucurbita pepo                 TTTTATGTAAAGGATCTAGTAGGTTGGGTAGCTTTTGCTATCTTTTTTTC
RRII105_cob                    TTTTATGTAAAGGATCTAGTAGGTTGGGTAGCTTTTGCTATCTTTTTTTC
Beta macrocarpa                TTTTATGTAAAGGATCTAGTAGGTTGGGTAGCTTTTGCTATCTTTTTTTC
Beta vulgaris vulgaris         TTTTATGTAAAGGATCTAGTAGGTTGGGTAGCTTTTGCTATCTTTTTTTC
Beta vulgaris maritima         TTTTATGTAAAGGATCTAGTAGGTTGGGTAGCTTTTGCTATCTTTTTTTC
RRIT251_cob                    TTTTATGTAAAGGATCTAGTAGGTTGGGTAGCTTTTGCTATCTTTTTTTC
Silene latifolia               TTTTATGTAAAGGATCTAGTAGGTTGGGTAGCTTTTGCTATCTTTTTTTC
RRIM600_cob                    TTTTATGTAAAGGATCTAGTAGGTTGGGTAGCTTTTGCTATCTTTTTTTC
RRIC110_cob                    TTTTATGTAAAGGATCTAGTAGGTTGGGTAGCTTTTGCTATCTTTTTTTC
Oryza sativa Japonica          TTTTATGTAAAGGATCTTGTAGGTUGGGTAGCTTUTGCTATCTTTTTTTC
Megaceros aenigmaticus         TCTTACGTAAAAGATCCAGTGGGTCGGGTAGCATTTGCCATCTTTTTCTC
                               * *** ** ** ****  ** *** ******* * *** ******** **

Arabidopsis thaliana           TATTTGGATTTTTTATGCTCCTAATGTTTTGGGACATCCCGACAATTATA
Brassica napus                 TATTTGGATTTTTTATGCTCCTAATGTTTTGGGACATCCCGACAATTATA
PB235_cob                      CATTTGGATTTTTTATGCTCCTAATGTTTTGGGGCATCCCGACAATTATA
Lotus japonicus                CATTTGGATTTTTTATGCTCCTAATGTTTTGGGGCATCCCGACAATTATA
Millettia pinnata              CATTTGGATTTTTTATGCTCCTAATGTTTTGGGCCATCCCGACAATTATA
BPM24_cob                      CATTTGGATTTTTTATGCTCCTAATGTTTTGGGGCATCCCGACAATTATA
Citrullus lanatus              ACTTTGGATTTTTTATGCTCCTAATGTTTTGGGGCATCCCGACAATTATA
Cucurbita pepo                 CATTTGGATTTTTTATGCTCCTAATGTTTTGGGGCATCCCGACAATTATA
RRII105_cob                    CATTTGGATTTTTTATGCTCCTAATGTTTTGGGGCATCCCGACAATTATA
Beta macrocarpa                CTTTTGGATTTTTTATGCTCCTAATGTTTTGGGGCATCCCGACAATTATA
Beta vulgaris vulgaris         CTTTTGGATTTTTTATGCTCCTAATGTTTTGGGGCATCCCGACAATTATA
Beta vulgaris maritima         CTTTTGGATTTTTTATGCTCCTAATGTTTTGGGGCATCCCGACAATTATA
RRIT251_cob                    CATTTGGATTTTTTATGCTCCTAATGTTTTGGGGCATCCCGACAATTATA
Silene latifolia               CTTTTGGATTTTTTATGCTCCTAATGTTTTGGGGCATCCCGACAATTATA
RRIM600_cob                    CATTTGGATTTTTTATGCTCCTAATGTTTTGGGGCATCCCGACAATTATA
RRIC110_cob                    CATTTGGATTTTTTATGCTCCTAATGTTTTGGGGCATCCCGACAATTATA
Oryza sativa Japonica          CATTTGGATTTTTTTTGCTCCTAATGTTTTGGGGCATCCCGACAATTATA
Megaceros aenigmaticus         TATTTTTGTTTCTTACGCACCTAATGTCTTGGGGCATCCCGACAACTATA
                                 ***   *** **  ** ******** ***** *********** ****

Arabidopsis thaliana           TACCTGCTAATCCGATGTCCACCCCGCCTCATATTGTGCCGGAATGGTAT
Brassica napus                 TACCTGCTAATCCGATGTCCACCCCGCCTCATATTGTGCCGGAATGGTAT
PB235_cob                      TACCTGCTAATCCGATGUCCACCCCGCCTCATATTGTGCCGGAATGGTAT
Lotus japonicus                TACCTGCTAATCCGATGUCCACCCCGCCTCATATTGTGCCGGAATGGTAT
Millettia pinnata              TACCTGCTAATCCGATGUCCACCCCGCCTCATATTGTGCCGGAATGGTAT
BPM24_cob                      TACCTGCTAATCCGATGUCCACCCCGCCTCATATTGTGCCGGAATGGTAT
Citrullus lanatus              TACCTGCTAATCCGATGUCCACCCCGCCTCATATTGTGCCGGAATGGTAT
Cucurbita pepo                 TACCTGCTAATCCGATGUCCACCCCGCCTCATATTGTGCCGGAATGGTAT
RRII105_cob                    TACCTGCTAATCCGATGUCCACCCCGCCTCATATTGTGCCGGAATGGTAT
Beta macrocarpa                TACCTGCTAATCCGATGUCCACCCCGCCTCATATTGTGCCGGAATGGTAT
Beta vulgaris vulgaris         TACCTGCTAATCCGATGUCCACCCCGCCTCATATTGTGCCGGAATGGTAT
Beta vulgaris maritima         TACCTGCTAATCCGATGUCCACCCCGCCTCATATTGTGCCGGAATGGTAT
RRIT251_cob                    TACCTGCTAATCCGATGUCCACCCCGCCTCATATTGTGCCGGAATGGTAT
Silene latifolia               TACCTGCTAATCCGATGUCCACCCCGCCTCATATTGTGCCGGAATGGTAT
RRIM600_cob                    TACCTGCTAATCCGATGUCCACCCCGCCTCATATTGTGCCGGAATGGTAT
RRIC110_cob                    TACCTGCTAATCCGATGUCCACCCCGCCTCATATTGTGCCGGAATGGTAT
Oryza sativa Japonica          TACCTGCTAATCCGATGUCCACCCCGCCTCATATTGTGCCGGAATGGTAT
Megaceros aenigmaticus         TACCCGCAAATCCGATGTCAACCCCGGCTCATATTGTGCCAGAATGGTAT
                               **** ** ********* * ****** ************* *********

Arabidopsis thaliana           TTCCTACCGATCUATGCCATTCTTCGTAGTATACCTGACAAAGCGGGAGG
Brassica napus                 TTCCTACCGATCUATGCCATTCTTCGTAGTATACCTGACAAAGCGGGAGG
PB235_cob                      TTCCTACCGATCUATGCCATTCTTCGTAGTATACCTGACAAATCGGGAGG
Lotus japonicus                TTCCTACCGATCUATGCCATTCTTCGTAGTATACCTGACAAATCGGGAGG
Millettia pinnata              TTCCTACCGATCUATGCCATTCTTCGTAGTATACCTGACAAATCGGGAGG
BPM24_cob                      TTCCTACCGATCUATGCCATTCTTCGTAGTATACCTGACAAATCGGGAGG
Citrullus lanatus              TTCCTACCGATCUATGCCATTCTTCGTAGTATACCTGACAAAGCGGGAGG
Cucurbita pepo                 TTCCTACCGATCUATGCCATTCTTCGTAGTATACCTGACAAAGCGGGAGG
```

```
RRII105_cob                       TTCCTACCGATCUATGCCATTCTTCGTAGTATACCTGACAAATCGGGAGG
Beta macrocarpa                   TTCTTACCCATCTATGCCATTCTTCGTAGTATACCTGACAAATCGGGAGG
Beta vulgaris vulgaris            TTCTTACCCATCTATGCCATTCTTCGTAGTATACCTGACAAATCGGGAGG
Beta vulgaris maritima            TTCTTACCCATCTATGCCATTCTTCGTAGTATACCTGACAAATCGGGAGG
RRIT251_cob                       TTCCTACCGATCUATGCCATTCTTCGTAGTATACCTGACAAATCGGGAGG
Silene latifolia                  TTCTTACCCATCTATGCCATTCTTCGTAGTATACCTGACAAATCGGGAGG
RRIM600_cob                       TTCCTACCGATCUATGCCATTCTTCGTAGTATACCTGACAAATCGGGAGG
RRIC110_cob                       TTCCTACCGATCUATGCCATTCTTCGTAGTATACCTGACAAATCGGGAGG
Oryza sativa Japonica             TTCCTACCGATCUATGCCATTCTTCGCAGTATACCTGACAAAGCGGGAGG
Megaceros aenigmaticus            TTCTTACCAGTCTATGCTATTCTTCGAAGTATACCTAACAAATTAGGGGG
                                  *** ****  ** **** ******** ********* *****   ** **

Arabidopsis thaliana              TGTAGCCGCAATAGCACUAGTTTTTATATGTCTCTTGGCTTTACCTTTTT
Brassica napus                    TGTAGCCGCAATAGCACUAGTTTTTATATGTCTCTTGGCTTTACCTTTTT
PB235_cob                         TGTAGCCGCAATAGCACCAGTTTTTATATGTCTGTTAGCTTTACCTTTTT
Lotus japonicus                   TGTAGCCGCAATAGCACUTGTTTTTATATGTCTGTTGGCTTTACCTTTTT
Millettia pinnata                 TGTAGCCGCAATAGCACUAGTTTTTATATGTCTGTTGGCTTTACCTTTTT
BPM24_cob                         TGTAGCCGCAATAGCACCAGTTTTTATATGTCTGTTAGCTTTACCTTTTT
Citrullus lanatus                 TGTAGCCGCAATAGCACUAGTTTTTATATGTCTGTTGGCTTTACCTTTTT
Cucurbita pepo                    TGTAGCCGCAATAGCACUAGTTTTTATATGTCTGTTGGCTTTACCTTTTT
RRII105_cob                       TGTAGCCGCAATAGCACCAGTTTTTATATGTCTGTTAGCTTTACCTTTTT
Beta macrocarpa                   TGTAGCCGCAATAGCACUCGTTTTTATATGTCTUTTGGCTTTACCTTTTT
Beta vulgaris vulgaris            TGTAGCCGCAATAGCACUCGTTTTTATATGTCTUTTGGCTTTACCTTTTT
Beta vulgaris maritima            TGTAGCCGCAATAGCACUCGTTTTTATATGTCTUTTGGCTTTACCTTTTT
RRIT251_cob                       TGTAGCCGCAATAGCACCAGTTTTTATATGTCTGTTAGCTTTACCTTTTT
Silene latifolia                  TGTAGCCGCAATAGCACUCGTTTTTATATGTCTCTTGGCTTTACCTTTTT
RRIM600_cob                       TGTAGCCGCAATAGCACCAGTTTTTATATGTCTGTTAGCTTTACCTTTTT
RRIC110_cob                       TGTAGCCGCAATAGCACCAGTTTTTATATGTCTGTTAGCTTTACCTTTTT
Oryza sativa Japonica             TGTAGCCGCAATAGCACUAGTTTTTATATCTCTCTTGGCTTTACCTTTTT
Megaceros aenigmaticus            TGTAGCTGCCATAGGACCAGTTTCCGTGTCATTATTTGCTCTACCTTTTA
                                  ****** ** **** **  ****   * *   * ** *** ********

Arabidopsis thaliana              TTAAAAGTATGTATGTGCGTAGTTCAAGTTTTCGACCGATTUACCAAGGA
Brassica napus                    TTAAAAGTATGTATGTGCGTAGTTCAAGTTTTCGACCGATTUACCAAGGA
PB235_cob                         TTAAAAGTATGTATGTGCGTAGTTCAAGTTTTCGCCCGATTUACCAAGGA
Lotus japonicus                   TTAAAAGTATGTACGTGCGTAGTTCAAGTTTTCGCCCTATTUACCAAGGA
Millettia pinnata                 TTAAAAGTATGTACGTGCGTAGTTCAAGTTTTCGCCCTATTUACCAAGGA
BPM24_cob                         TTAAAAGTATGTATGTGCGTAGTTCAAGTTTTCGCCCGATTUACCAAGGA
Citrullus lanatus                 TTAAAAGTATGTATGTGCGTAGTTCAAGTTTTCGCCCTATTUACCAAGGA
Cucurbita pepo                    TTAAAAGTATGTATGTGCGTAGTTCAAGTTTTCGCCCTATTUACCAAGGA
RRII105_cob                       TTAAAAGTATGTATGTGCGTAGTTCAAGTTTTCGCCCGATTUACCAAGGA
Beta macrocarpa                   TTAAAAGTATGTATGTACGTAGTTCAAGTTTTCGCCCGATTTACCAAGGA
Beta vulgaris vulgaris            TTAAAAGTATGTATGTACGTAGTTCAAGTTTTCGCCCGATTTACCAAGGA
Beta vulgaris maritima            TTAAAAGTATGTATGTACGTAGTTCAAGTTTTCGCCCGATTTACCAAGGA
RRIT251_cob                       TTAAAAGTATGTATGTGCGTAGTTCAAGTTTTCGCCCGATTUACCAAGGA
Silene latifolia                  TTAAGAGTATGTATGTACGTAGTTCCAGTTTTCGCCCGATTTACCAAGGA
RRIM600_cob                       TTAAAAGTATGTATGTGCGTAGTTCAAGTTTTCGCCCGATTNACCAAGGA
RRIC110_cob                       TTAAAAGTATGTATGTGCGTAGTTCAAGTTTTCGCCCGATTUACCAAGGA
Oryza sativa Japonica             TTAAAGAAATGTATGTGCGTAGTTCAAGTTTTCGACCGATTUACCAAGGA
Megaceros aenigmaticus            TTAACACATCATATGTACGTAGTTCAAGTTTCCGACCGATTCACCAAAAA
                                  ****       ** ** ******** ***** ** ** *** *****  *

Arabidopsis thaliana              ATGTTTTGGTTGCTTTTGGCGGATTGCTTACTACTAGGTTGGATCGGATG
Brassica napus                    ATGTTTTGGTTGCTTTTGGCGGATTGCTTACTACTAGGTTGGATCGGATG
PB235_cob                         ATCTTTTGGTTGCTTTTGGCGGATTGCTTACTACTAGGTTGGATCGGATG
Lotus japonicus                   ATATTTTGGTTGCTTTTGGCGGATUGCTTACTACTAGGTTGGATCGGATG
Millettia pinnata                 ATATTTTGGTTGCTTTTGGCAGATUGCTTACTACTAGGTTGGATCGGATG
BPM24_cob                         ATCTTTTGGTTGCTTTTGGCGGATTGCTTACTACTAGGTTGGATCGGATG
Citrullus lanatus                 CTATTTTGGTTGCTTTTGGCGGATTGCTTACTACTAGGTTGGATCGGATG
Cucurbita pepo                    CTATTTTGGTTGCTTTTGGCAGATTGCTTACTACTAGGTTGGATCGGATG
RRII105_cob                       ATCTTTTGGTTGCTTTTGGCGGATTGCTTACTACTAGGTTGGATCGGATG
Beta macrocarpa                   CTCTTTTGGTTGCTTTTGGCGGATTGCTTACTACTAGGTTGGATCGGATG
```

```
Beta vulgaris vulgaris         CTCTTTTGGTTGCTTTTGGCGGATTGCTTACTACTAGGTTGGATCGGATG
Beta vulgaris maritima         CTCTTTTGGTTGCTTTTGGCGGATTGCTTACTACTAGGTTGGATCGGATG
RRIT251_cob                    ATCTTTTGGTTGCTTTTGGCGGATTGCTTACTACTAGGTTGGATCGGATG
Silene latifolia               ATCTTTTGGTTGCTTTTGGCGGATTGCTTACTACTAGGTTGGATCGGATG
RRIM600_cob                    ATCTTTTGGTTGCTTTTGGCGGATTGCTTACTACTAGGTTGGATCGGATG
RRIC110_cob                    ATCTTTTGGTTGCTTTTGGCGGATTGCTTACTACTAGGTTGGATCGGATG
Oryza sativa Japonica          ATATTTTGGTTGCTTTTGGCGGATTGCTTACTACTAGGTTGGATCGGATG
Megaceros aenigmaticus         ATGTTTTGGTTGCCCTTGGCAGATTGCCTACTTTTAGGTTGGATTGGATG
                                * **********  ***** *** ** ****  ********** *****

Arabidopsis thaliana           TCAACCTGTGGAGGCTCCATTTGTTACTATTGGACAAATTTCTUCTTTGG
Brassica napus                 TCAACCTGTGGAAGCACCATTTGTTACTATTGGACAAATTTCTUCTTTGG
PB235_cob                      TCAACCTGTGGAGGCACCATTTGTTACTATTGGACAAATTTCTUCTTTTG
Lotus japonicus                TCAACCTGTGGAGGCACCATTTGTTACTATTGGACAAATTUCTUCTTTAG
Millettia pinnata              TCAACCTGTGGAGGCACCATTTGTTACTATTGGACAAATTUCTUCTTTTG
BPM24_cob                      TCAACCTGTGGAGGCACCATTTGTTACTATTGGACAAATTTCTUCTTTTG
Citrullus lanatus              TCAACCTGTGGAGGCACCATTTGTTACTATTGGACAAATTTCTUCTTTTG
Cucurbita pepo                 TCAACCTGTGGAGGCACCATTTGTTACTATTGGACAAATTTCTUCTTTTG
RRII105_cob                    TCAACCTGTGGAGGCACCATTTGTTACTATTGGACAAATTTCTUCTTTTG
Beta macrocarpa                TCAACCTGTGGAAGCACCCTTTGTTACTATTGGACAAATTUCTTCTTTTC
Beta vulgaris vulgaris         TCAACCTGTGGAAGCACCCTTTGTTACTATTGGACAAATTUCTTCTTTTC
Beta vulgaris maritima         TCAACCTGTGGAAGCACCCTTTGTTACTATTGGACAAATTUCTTCTTTTC
RRIT251_cob                    TCAACCTGTGGAGGCACCATTTGTTACTATTGGACAAATTTCTUCTTTTG
Silene latifolia               TCAACCTGTGGAAGCACCATTTGTTACTATCGGACAAATTTCTTCTTTAG
RRIM600_cob                    TCAACCTGTGGAGGCACCATTTGTTACTATTGGACAAATTTCTNCTTTTG
RRIC110_cob                    TCAACCTGTGGAGGCACCATTTGTTACTATTGGACAAATTTCTUCTTTTG
Oryza sativa Japonica          TCAACCTGTGGAGGCACCATTTGTTACTATTGGACAAATTUCTTCTTTCT
Megaceros aenigmaticus         TCAACCTGTGGAGGCACCATATGTTACTATTGGCCAAATTGCTTCGGTTG
                               ************ ** ** * ********* ** ****** ** *  *

Arabidopsis thaliana           TTTTCTTCTTGTTCTTTGCCATAACGCCCATTCTGGGACGAGTTGGAAGA
Brassica napus                 TTTTCTTCTTGTTCTTTGCCATAACGCCCATTCTGGGACGAGTTGGAAGA
PB235_cob                      TTTTCTTCTTGTTCTTTGCCATAACGCCCATTCUGGGACGAGTTGGAAAA
Lotus japonicus                TTTTCTTCTTGTTCTTTGCCATAACGCCCATTCUGGGACGAGTTGGAATC
Millettia pinnata              TTTTCTTCTTGTTCTTTGCCATAACGCCCATTCUGGGACGAGTTGGAAGA
BPM24_cob                      TTTTCTTCTTGTTCTTTGCCATAACGCCCATTCUGGGACGAGTTGGAAAA
Citrullus lanatus              TTTTCTTCTTGTTCTTTGCCATAACGCCCATTCUGGGACGAGTTGGAAGA
Cucurbita pepo                 TTTTCTTCTTGTTCTTTGCCATAACGCCTATTCUGGGACGAGTTGGAAGA
RRII105_cob                    TTTTCTTCTTGTTCTTTGCCATAACGCCCATTCUGGGACGAGTTGGAAAA
Beta macrocarpa                TTTTCTTCTTGTTCTTTGCCATAACGCCCATTCTGGGACGAGTTGGAAGA
Beta vulgaris vulgaris         TTTTCTTCTTGTTCTTTGCCATAACGCCCATTCTGGGACGAGTTGGAAGA
Beta vulgaris maritima         TTTTCTTCTTGTTCTTTGCCATAACGCCCATTCTGGGACGAGTTGGAAGA
RRIT251_cob                    TTTTCTTCTTGTTCTTTGCCATAACGCCCATTCUGGGACGAGTTGGAAAA
Silene latifolia               TTTTCTTCTTGTTCTTTGCCATAACGCCCATTCTGGGACGAGTTGGAAGA
RRIM600_cob                    TTTTCTTCTTGTTCTTTGCCATAACGCCCATTCNGGGACGAGTTGGAAAA
RRIC110_cob                    TTTTCTTCTTGTTCTTTGCCATAACGCCCATTCUGGGACGAGTTGGAAAA
Oryza sativa Japonica          TTTTCTTUTTGTTCTTTGCCATAACGCCUATTCUGGGACGAGTTGGAAGA
Megaceros aenigmaticus         CTTCTTTCTTATACTTTGTTATTACGCCTATTCTTGG-CAAATTAGAAGA
                                **  ** ** * *****  ** ***** ****  ** * * ** ***

Arabidopsis thaliana           GGAATTC-CTAATTCTTACACGGATGAGACTGA-----------TCACAC
Brassica napus                 GGAATTC-CTAATTCTTACACGGATGAGACTGA-----------TCACAC
PB235_cob                      GGAATTC-CTAATTCTTACACGGATGAGACTGA-----------TCACAC
Lotus japonicus                GGAATTC-CGAATTCTTACACGGATGAGACTGA-----------TCA---
Millettia pinnata              GGAATTC-CGAATTCTTACACGGATGAGACTGA-----------TCA---
BPM24_cob                      GGAATTC-CTAATTCTTACACGGATGAGACTGA-----------TCA---
Citrullus lanatus              GGAATTC-CTAATTCTTACACGGATGAGCCCTAGCCCTGTAAGCTCACAC
Cucurbita pepo                 GGAATCC-CTAATTCTTACACAGATGAGCCCTAGCCCTGTAAGCTCACAC
RRII105_cob                    GGAATTC-CTAATTCTTACACGGATGAGACTGA-----------TCACAC
Beta macrocarpa                AGAATTC-CTAATTCTTACAUGGATGAGACTGAGAACACCTGATC-----
Beta vulgaris vulgaris         AGAATTC-CTAATTCTTACAUGGATGAGACTGAGAACACCTGATC-----
Beta vulgaris maritima         AGAATTC-CTAATTCTTACAUGGATGAGACTGAGAACACCTGATC-----
```

```
RRIT251_cob                       GGAATTC-CTAATTCTTACACGGATGAGACTGATCACACCTGA-------
Silene latifolia                  AGAATTC-CTAATTCTTACAUGGATGAGACTGATCACACCTGAATTTAAT
RRIM600_cob                       GGAATTC-CTAATTCTTACACGGATGAGACTGATCACACCTGA-------
RRIC110_cob                       GGAATTC-CTAATTCTTACACGGATGAGACTGATCACACCTGA-------
Oryza sativa Japonica             GGAATTC-CAAAATATTACACGGATGAGACTCATCGCACCGGATCAGTCT
Megaceros aenigmaticus            CAGATTAATCAATACTTCAAGCGGGGAAGGTAAGCTTCCC----------
                                     **     **   **  *  *  **     *

Arabidopsis thaliana              CTGATCAGTGAAAAATTCTGA-CACCAATCATT------TACATATTACA
Brassica napus                    CTGATCAGTGAAAAATTTTGA-CACCAATCATT------TACATATTACA
PB235_cob                         CTGA----------------------------------------------
Lotus japonicus                   -------GTGAAAAATTCTGA-CCCCAATCATT------TACGTATTACA
Millettia pinnata                 -------GTGAAAAATTCTGA-CACCAATCATT------TACGTATTACA
BPM24_cob                         -------CACCTGA------------------------------------
Citrullus lanatus                 CTAATCAGTGAAAAATTCTGA-CACAAATCATTGTAGAGTGAGTATTACA
Cucurbita pepo                    CTAATCAGTGCAAAATTCTGA-CACAAATCATTGTAGAGTTAGTATTACA
RRII105_cob                       CTGA----------------------------------------------
Beta macrocarpa                   ------AGTGAAAAATTCTTA-CACCAAGAATTGACGAGTGAGTATTACA
Beta vulgaris vulgaris            ------AGTGAAAAATTCTTA-CACCAAGAATTGACGAGTGAGTATTACA
Beta vulgaris maritima            ------AGTGAAAAATTCTTA-CACCAAGAATTGACGAGTGAGTATTACA
RRIT251_cob                       --------------------------------------------------
Silene latifolia                  TTTAA-AATTAAAAATTATTA-CACCAAGAATTGACGAGTGAGTCTTACA
RRIM600_cob                       --------------------------------------------------
RRIC110_cob                       --------------------------------------------------
Oryza sativa Japonica             CTTA--GACGGATGAGACTGATCACACCTGATCAGTGATCAATTCTGGCA
Megaceros aenigmaticus            --------TGCTTCTTCGTCACCACCAGT-ATTATGTATGCGATTTCGTC


Arabidopsis thaliana              CCAAGAATT----GACAAGCAGATAAGT--TTTCTAGTTTGCTATGTTGA
Brassica napus                    CCAAGAATT----GACAAGCGGATAAGT--TTTCTAGTTTGCTATGTTGA
PB235_cob                         --------------------------------------------------
Lotus japonicus                   CCAAGAATTCATTGACAAGCAAATGAAT--TGGCTATGTTG-TCCGTCGG
Millettia pinnata                 CCAAGAATTAATTGACAAGCGCATTAGT--T--TTATGGC--TATGTTGA
BPM24_cob                         --------------------------------------------------
Citrullus lanatus                 CCAAGAATTCATTGACAAGCGGAT------TTTCTAGTTGGCTTTGTTGA
Cucurbita pepo                    CCAAGAATGAATTGACAAGCAGAT------TTTCTAGTTGGCTTTGTTGA
RRII105_cob                       --------------------------------------------------
Beta macrocarpa                   CCAAGAATT----GACAAGCGGATTCGTTTTTTCTAGTTGGCTATGTTGA
Beta vulgaris vulgaris            CCAAGAATT----GACAAGCGGATTCGTTTTTTCTAGTTGGCTATGTTGA
Beta vulgaris maritima            CCAAGAATT----GACAAGCGGATTCGTTTTTTCTAGTTGGCTATGTTGA
RRIT251_cob                       --------------------------------------------------
Silene latifolia                  CCAAGAATT----GACAAGCGGATTCGTTTTTTCTAGTTGGCTATAAGGG
RRIM600_cob                       --------------------------------------------------
RRIC110_cob                       --------------------------------------------------
Oryza sativa Japonica             CAATGAATT----TACGAGTTATTTTACACAATGAATTTA--CAAGCAGA
Megaceros aenigmaticus            CCTCGGTTGA----------------------------------------


Arabidopsis thaliana              TATAGCTTAGATAGGGAAAAGA----TAACT--CCACTATAGA-GTAGGG
Brassica napus                    TATAGCTTAGATAGGGAAAAGA----TAACT--CCACTATAGG-GTAGGG
PB235_cob                         --------------------------------------------------
Lotus japonicus                   TGC-GCTCATTCCGCCGGATTT----TTTTTTACCAGGACAGGAGTCAAA
Millettia pinnata                 TATAGCTTAGAAAAGGGAAAAG----------AGAGTGACAGAACTGAAA
BPM24_cob                         --------------------------------------------------
Citrullus lanatus                 TATAGCTTAGATAGGGAAAAGA----T--------ACGAGAGG-GTAGGG
Cucurbita pepo                    TATAGCTTAGATAGGGAAAAGA----T--------ACGAGAGG-GTAGGG
RRII105_cob                       --------------------------------------------------
Beta macrocarpa                   TATAGCTTAGATAGGGAAAAGAAAGTCTACTATAGGGTAAGGGTGAACTT
Beta vulgaris vulgaris            TATAGCTTAGATAGGGAAAAGAAAGTCTACTATAGGGTAAGGGTGAACTT
Beta vulgaris maritima            TATAGCTTAGATAGGGAAAAGAAAGTCTACTATAGGGTAAGGGTGAACTT
RRIT251_cob                       --------------------------------------------------
Silene latifolia                  -ATGCCTAAAAAAGCCTAGCG-----CTGATAAAGGTAATTGAT--ATTT
```

```
RRIM600_cob                       --------------------------------------------------
RRIC110_cob                       --------------------------------------------------
Oryza sativa Japonica             TG-AGTTTGCAACGGTAGACCTA--TCTCCTGAAAAGAGTTCAGTAAACA
Megaceros aenigmaticus            --------------------------------------------------


Arabidopsis thaliana              CTGTACTTCAAAAATCAAAAAGGGTCCCTCTCCCCCTTTTTTTATTAAAA
Brassica napus                    CTGTACTTCAAAAATCAAAAAGGGTCCCTCTCCCCCTTTTTT-ATTAAAA
PB235_cob                         --------------------------------------------------
Lotus japonicus                   GAAGAGCGGATCCAAGACCAAGACGACTTCTTTAGAAAGAAGGGCAGAAA
Millettia pinnata                 AAATGACCTAAACACCCTTAAGAATCCATAGGGAATAAATAGGGACGTGA
BPM24_cob                         --------------------------------------------------
Citrullus lanatus                 CTGAACTTTGAA-ATCCGGGGGCTTTGTTC--------------------
Cucurbita pepo                    CTGCACTTTGAA-ATCCGGGGGCTTTGTTA--------------------
RRII105_cob                       --------------------------------------------------
Beta macrocarpa                   TCAAATCCGGTTGTTCCCGAAACTCCCCGTCCCTTAC-------------
Beta vulgaris vulgaris            TCAAATCCGGTTGTTCCCGAAACTCCCCGTCCCTTAC-------------
Beta vulgaris maritima            TCAAATCCGGTTGTTCCCGAAACTCCCCGTCCCTTAC-------------
RRIT251_cob                       --------------------------------------------------
Silene latifolia                  AAGCATACCGCTTACCAAGAACAGTTTCTTTCTTC---------------
RRIM600_cob                       --------------------------------------------------
RRIC110_cob                       --------------------------------------------------
Oryza sativa Japonica             AGGGAACGAAGCGACCGATAACGTCCCCTCGGGGAGGAGTGTTTTA----
Megaceros aenigmaticus            --------------------------------------------------


Arabidopsis thaliana              ----------
Brassica napus                    A---------
PB235_cob                         ----------
Lotus japonicus                   ATCCA-----
Millettia pinnata                 GTCCCCTCTC
BPM24_cob                         ----------
Citrullus lanatus                 ----------
Cucurbita pepo                    ----------
RRII105_cob                       ----------
Beta macrocarpa                   ----------
Beta vulgaris vulgaris            ----------
Beta vulgaris maritima            ----------
RRIT251_cob                       ----------
Silene latifolia                  ----------
RRIM600_cob                       ----------
RRIC110_cob                       ----------
Oryza sativa Japonica             ----------
Megaceros aenigmaticus            ----------
```

###################

cox1 multiple sequence alignment

```
Brassica napus                    CAAATTT---ATGAAAAATCTGGTTC---GATGGCTGTTCTCCACAAACC
PB235_cox1                        ----------ATGACAAATCTGGTUC---GATGGCTGTTCTCCACTAACC
Citrullus lanatus                 ----------ACGACAAATCUGGTCC---GATGGCTGTTCTCCACTAACC
BPM24_cox1                        ----------ATGACAAATCTGGTUC---GATGGCTGTTCTCCACTAACC
RRIC110_cox1                      ----------ATGACAAATCTGGTUC---GATGGCTGTTCTCCACTAACC
Cucurbita pepo                    ----------ACGAGAAATCTGGTCC---GATGGCTGTTCTCCACTAACC
Lotus japonicus                   ----------ATGACAAATCUGGTUC---GATGGCTGTTCTCCACTAACC
Millettia pinnata                 ----------ATGACAAATCUGGTUC---GATGGCTGTTCTCCACTAACC
RRIT251_cox1                      ----------ATGACAAATCTGGTUC---GATGGCTGTTCTCCACTAACC
Oryza sativa Japonica             AAATCAA---ATGACAAATCTGGTCC---GATGGCTCTTCTCCACTAACC
RRIM600_cox1                      ----------ATGACAAATCTGGTUC---GATGGCTGTTCTCCACTAACC
RRII105_cox1                      ----------ATGACAAATCTGGTUC---GATGGCTGTTCTCCACTAACC
Cycas taitungensis                TAATCTT---AUGACAAATTTGGTCC---GATGGCCGTTCCCCACCAACC
Megaceros aenigmaticus            ----------ATGAAAAATTTTGCACAAAGATGGCTTTTTTCCACAAACC
```

```
Phaeoceros laevis              GGGTTCTGAAATGAAAAATTTTGCACAAAGATGGCTTTTTTCCACAAACC
                                         * ** ****   *  *   ******  **  **** ****

Brassica napus                 ACAAGGATATAGGGACTCTCTATTTCATTTTCGGTGCCATTGCTGGAGTG
PB235_cox1                     ACAAGGATATAGGGACTTTATATTTCATCTTCGGTGCCATTGCTGGAGTG
Citrullus lanatus              ACAAGGATATAGGGACTCTATATTTCATCTTCGGTGCCATTGCTGGAGTG
BPM24_cox1                     ACAAGGATATAGGGACTTTATATTTCATCTTCGGTGCCATTGCTGGAGTG
RRIC110_cox1                   ACAAGGATATAGGGACTTTATATTTCATCTTCGGTGCCATTGCTGGAGTG
Cucurbita pepo                 ACAAGGATATAGGGACTCTATATTTCATCTTCGGTGCCATTGCAGGAGTG
Lotus japonicus                ACAAGGATATAGGGACTCTATATTTCATCTTCGGTGCCATTGCTGGAGTG
Millettia pinnata              ACAAGGATATAGGGACTCTATATTTCATCTTCGGTGCCATTGCTGGAGTG
RRIT251_cox1                   ACAAGGATATAGGGACTTTATATTTCATCTTCGGTGCCATTGCTGGAGTG
Oryza sativa Japonica          ACAAGGATATCGGTACTCTATATTTCATCTTCGGTGCCATTGCAGGAGTG
RRIM600_cox1                   ACAAGGATATAGGGACTTTATATTTCATCTTCGGTGCCATTGCTGGAGTG
RRII105_cox1                   ACAAGGATATAGGGACTTTATATTTCATCTTCGGTGCCATTGCTGGAGTG
Cycas taitungensis             ACAGGGATATAGGGACTCTATATTCAATCTTTGGTGCCATTGCTGGAGTG
Megaceros aenigmaticus         ACAAAGATATAGGTACTCCACATTCAATTTTCGGTGCTATTGCTGGAGTA
Phaeoceros laevis              ACAAAGATATAGGTACTCCATATTCAATTTTCGGTGCTATTGCTGGAGTA
                               ***  ***** ** ***    ***  ** ** ***** ***** *****

Brassica napus                 ATGGGCACATGCTTCTCAGTACTTATTCGTATGGAATTAGCACGACCCGG
PB235_cox1                     ATGGGCACATGCTTCTCAGTACTGATTCGTATGGAATTAGCACGACCGGG
Citrullus lanatus              ATGGGCACATGCTTCTCAGTACTGATTCGTATGGAATTAGCACGACCCGG
BPM24_cox1                     ATGGGCACATGCTTCTCAGTACTGATTCGTATGGAATTAGCACGACCGGG
RRIC110_cox1                   ATGGGCACATGCTTCTCAGTACTGATTCGTATGGAATTAGCACGACCGGG
Cucurbita pepo                 ATGGGCACATGCTTCTCAGTACTGATTCGTATGGAATTAGCACGACCCGG
Lotus japonicus                ATGGGCACATGCTTCTCAGTATTGATTCGTATGGAATTAGCACGACCCGG
Millettia pinnata              ATGGGCACATGCTTCTCAGTATTGATTCGTATGGAATTAGCACGACCCGG
RRIT251_cox1                   ATGGGCACATGCTTCTCAGTACTGATTCGTATGGAATTAGCACGACCGGG
Oryza sativa Japonica          ATGGGCACATGCTTCTCCGTACTGATTCGTATGGAATTAGCCCGACCCGG
RRIM600_cox1                   ATGGGCACATGCTTCTCAGTACTGATTCGTATGGAATTAGCACGACCGGG
RRII105_cox1                   ATGGGCACATGCTTCTCAGTACTGATTCGTATGGAATTAGCACGACCGGG
Cycas taitungensis             ATGGGCACATGCTTCTCAGTACCAATTCGTATGGAATTGGCACAACCCGG
Megaceros aenigmaticus         ATGGGTACATGCTTCTTAGTATCAATTCGTATGGAATTAGCACAACCCGG
Phaeoceros laevis              ATGGGTACATGCTTTTCAGTATTAATTTGTATGGAATTAGCACAACTCGG
                               ***** ******** *  ***   *** ********** ** * **  **

Brassica napus                 CGATCAAATTCTTGGTGGGAATCATCAACTTTATAATGTTTTAATAACAG
PB235_cox1                     CGATCAAATTCTTGGTGGGAATCATCAACTTTATAATGTTTTAATAACGG
Citrullus lanatus              CGATCAAATTCTTGGTGGGAATCATCAACTTTATAATGTTTTAATAACGG
BPM24_cox1                     CGATCAAATTCTTGGTGGGAATCATCAACTTTATAATGTTTTAATAACGG
RRIC110_cox1                   CGATCAAATTCTTGGTGGGAATCATCAACTTTATAATGTTTTAATAACGG
Cucurbita pepo                 CGATCAAATTCTTGGTGGGAATCATCAACTTTATAATGTTTTAATAACGG
Lotus japonicus                CGATCAAATTCTTGGTGGGAATCATCAACTTTATAATGTTTTAATAACGG
Millettia pinnata              CGATCAAATTCTTGGTGGGAATCATCAACTTTATAATGTTTTAATAACGG
RRIT251_cox1                   CGATCAAATTCTTGGTGGGAATCATCAACTTTATAATGTTTTAATAACGG
Oryza sativa Japonica          CGATCAAATTCTTGGTGGGAATCATCAACTTTATAATGTTTTAATAACGG
RRIM600_cox1                   CGATCAAATTCTTGGTGGGAATCATCAACTTTATAATGTTTTAATAACGG
RRII105_cox1                   CGATCAAATTCTTGGTGGGAATCATCAACTTTATAATGTTTTAATAACGG
Cycas taitungensis             CGATCAAATTCTTGGTGGAAATCATCAACTTCATAATGTGTTAATAACGG
Megaceros aenigmaticus         CAATCAAATTCTCAGTGGAAATCATCAACTCTATAATGTGTTAATAACAG
Phaeoceros laevis              CAATCAAATTCTCAGTGGAAATCATCAACTTTATAATGTGTTAATAACAG
                               * **********  **** ***********  ******* ******** *

Brassica napus                 CTCATGCTTTTTTAATGATCTTTTTTATGGTTATGCCGGCGATGATAGGT
PB235_cox1                     CTCACGCTTTTTTAATGATCTTTTTTATGGTTATGCCGGCGATGATAGGT
Citrullus lanatus              CTCACGCTTTTTTAATGATCTTTTTTATGGTTATGCCGGCGATGATAGGT
BPM24_cox1                     CTCACGCTTTTTTAATGATCTTTTTTATGGTTATGCCGGCGATGATAGGT
RRIC110_cox1                   CTCACGCTTTTTTAATGATCTTTTTTATGGTTATGCCGGCGATGATAGGT
Cucurbita pepo                 CTCACGCTTTTTTAATGATCTTTTTTATGGTTATGCCGGCGATGATAGGT
Lotus japonicus                CTCACGCTTTTTTAATGATATTTTTTATGGTTATGCCGGCGATGATAGGT
Millettia pinnata              CTCACGCTTTTTTAATGATCTTTTTTATGGTTATGCCGGCGATGATAGGT
```

```
RRIT251_cox1                      CTCACGCTTTTTTAATGATCTTTTTTATGGTTATGCCGGCGATGATAGGT
Oryza sativa Japonica             CTCACGCTTTTTTAATGATCTTTTTTATGGTTATGCCGGCGATGATAGGT
RRIM600_cox1                      CTCACGCTTTTTTAATGATCTTTTTTATGGTTATGCCGGCGATGATAGGT
RRII105_cox1                      CTCACGCTTTTTTAATGATCTTTTTTATGGTTATGCCGGCGATGATAGGT
Cycas taitungensis                CTCACGCTTCTTTAATGATCTCTTTTATGGTCATGCCGGCGATGATAGGT
Megaceros aenigmaticus            CTCACGCTTTTTTAATGATATCTTTTATGGTTACGCCTGCCATGATAGGT
Phaeoceros laevis                 CTTACGTTTTTTTAATGATATTTTTTATGGTTATGCTTGCCATGATAGGT
                                  ** * * ** ********* * ********* * **  ** *********

Brassica napus                    GGATTTGGTAATTGGTTTGTTCCGATTCTGATAGGTGCACCTGACATGGC
PB235_cox1                        GGATUTGGTAATTGGTUTGTTCCGATTCTGATAGGTGCACCTGACATGGC
Citrullus lanatus                 GGATUTGGTAATTGGTUTGTTCCGATTCTGATAGGTGCACCTGACATGGC
BPM24_cox1                        GGATUTGGTAATTGGTUTGTTCCGATTCTGATAGGTGCACCTGACATGGC
RRIC110_cox1                      GGATUTGGTAATTGGTUTGTTCCGATTCTGATAGGTGCACCTGACATGGC
Cucurbita pepo                    GGATUTGGTAATTGGTUTGTTCCGATTCTGATAGGTGCACCTGACATGGC
Lotus japonicus                   GGATUTGGTAATTGGTUTGTTCCGATTCTGATAGGTGCACCTGACATGGC
Millettia pinnata                 GGATUTGGTAATTGGTUTGTTCCGATTCTGATAGGTGCACCTGACATGGC
RRIT251_cox1                      GGATUTGGTAATTGGTUTGTTCCGATTCTGATAGGTGCACCTGACATGGC
Oryza sativa Japonica             GGATTTGGTAATTGGTTTGTTCCGATTCTGATAGGTGCACCTGACATGGC
RRIM600_cox1                      GGATNTGGTAATTGGTNTGTTCCGATTCTGATAGGTGCACCTGACATGGC
RRII105_cox1                      GGATUTGGTAATTGGTUTGTTCCGATTCTGATAGGTGCACCTGACATGGC
Cycas taitungensis                GGATTTGGTAATTGGTTCGTTCCGATTCTTATAGGTGCACCTGACATGGC
Megaceros aenigmaticus            GGATTTGGTAATTGGTTTGTTCCGATTCTCATAGGTGCACCTGACATGGC
Phaeoceros laevis                 GGATTTGGTAATTGGTTTGTTTTGATTCTTATAGGTGCACCTGACATGGC
                                  **** ***********  ***  ****** ********************

Brassica napus                    ATTTCCACGATTAAATAATATTTCATTCTGGTTGTTGCCACCAAGTCUCT
PB235_cox1                        ATTTCCACGATTAAATAATATTTCATTCTGGTTGTTGCCACCAAGTCTCT
Citrullus lanatus                 ATTTCCACGATTAAATAATATTTCATTCTGGTTGTTGCCACCAAGTCTCT
BPM24_cox1                        ATTTCCACGATTAAATAATATTTCATTCTGGTTGTTGCCACCAAGTCTCT
RRIC110_cox1                      ATTTCCACGATTAAATAATATTTCATTCTGGTTGTTGCCACCAAGTCTCT
Cucurbita pepo                    ATTTCCCCGATTAAATAATATTTCATTCTGGTTGTTGCCACCAAGTCTCT
Lotus japonicus                   ATTTCCACGATTAAATAATATTTCATTCTGGTTGTTGCCGCCAAGTCTCT
Millettia pinnata                 ATTTCCACGATTAAATAATATTTCATTCTGGTTGTTGCCGCCAAGTCTCT
RRIT251_cox1                      ATTTCCACGATTAAATAATATTTCATTCTGGTTGTTGCCACCAAGTCTCT
Oryza sativa Japonica             ATTTCCACGATTAAATAATATATCATTCTGGTTGTTGCCACCAAGTCTCT
RRIM600_cox1                      ATTTCCACGATTAAATAATATTTCATTCTGGTTGTTGCCACCAAGTCTCT
RRII105_cox1                      ATTTCCACGATTAAATAATATTTCATTCTGGTTGTTGCCACCAAGTCTCT
Cycas taitungensis                ATTTCCACGATTAAATAATATCTCATTCCGGTTGCTGCCACCTTCGCTGT
Megaceros aenigmaticus            ATTTCCACGATTGAATAATATCAGTTCTTGGCTATTACCGCCGTCACTGC
Phaeoceros laevis                 ATTTTTACGATTGAATAATATCAGTTTTTGGCTATTACCGCCGTCACTGC
                                  ****   ***** ********    *   ** *  * ** **    *

Brassica napus                    TGCTCCTATTAAGCTCAGCCTTAGTAGAAGTAGGTAGCGGCACTGGGTGG
PB235_cox1                        TACTCNTATTAAGCTCAGCCTTAGTGGAAGTGGGCAGCGGCACTGGGTGG
Citrullus lanatus                 TGCTCCTATTAAGCTCAGCCTTAGTAGAAGTGGGTAGCGGCACTGGGTGG
BPM24_cox1                        TACTCNTATTAAGCTCAGCCTTAGTGGAAGTGGGCAGCGGCACTGGGTGG
RRIC110_cox1                      TACTCNTATTAAGCTCAGCCTTAGTGGAAGTGGGCAGCGGCACTGGGTGG
Cucurbita pepo                    TGCTCCTATTAAGCTCAGCCTTAGTAGAAGTGGGTAGCGGCACTGGGTGG
Lotus japonicus                   TGCTCCTATTAAGCTCAGCCTTAGTAGAAGTGGGTAGCGGCACTGGGTGG
Millettia pinnata                 TGCTCCTATTAAGCTCAGCCTTAGTAGAAGTGGGTAGCGGCACTGGGTGG
RRIT251_cox1                      TACTCCTATTAAGCTCAGCCTTAGTGGAAGTGGGCAGCGGCACTGGGTGG
Oryza sativa Japonica             TGCTCCTATTAAGCTCAGCCTTAGTAGAAGTGGGCAGCGGCACTGGGTGG
RRIM600_cox1                      TACTCUTATTAAGCTCAGCCTTAGTGGAAGTGGGCAGCGGCACTGGGTGG
RRII105_cox1                      TACTCCTATTAAGCTCAGCCTTAGTGGAAGTGGGCAGCGGCACTGGGTGG
Cycas taitungensis                TGCTCCTATTAAGCTCAGCCCTGGTAGAAGTGGGTAGCGGCACTGGGTGG
Megaceros aenigmaticus            TACTTCTTTTAAGTCCTGCTTTGGTAGAAGTTGGCGCAGGTACCGGCTGG
Phaeoceros laevis                 TACTTCTTTTAAGTTCCGCTTTGGTAGAAGTTGGCGCAGGTACTGGCTGG
                                  * **  * *****  * **  * ** ***** **    ** ** ** ***

Brassica napus                    ACGGTCTATCCGCCCTTAAGTGGTATTACCAGTCATTCTGGAGGAGCAGT
PB235_cox1                        ACGGTCTATCCGCCCTTAAGTGGTATTACCAGCCATTCTGGAGGAGCAGT
```

```
Citrullus lanatus                 ACGGTCTATCCGCCCTTAAGTGGTATTACCAGCCATTCTGGAGGAGCAGT
BPM24_cox1                        ACGGTCTATCCGCCCTTAAGTGGTATTACCAGCCATTCTGGAGGAGCAGT
RRIC110_cox1                      ACGGTCTATCCGCCCTTAAGTGGTATTACCAGCCATTCTGGAGGAGCAGT
Cucurbita pepo                    ACGGTCTATCCGCCCTTAAGTGGTATTACCAGCCATTCTGGAGGAGCAGT
Lotus japonicus                   ACGGTCTATCCGCCCTTAAGTGGTATTACCAGCCATTCTGGAGGAGCAGT
Millettia pinnata                 ACGGTCTATCCGCCCTTAAGTGGTATTACCAGCCATTCTGGAGGAGCAGT
RRIT251_cox1                      ACGGTCTATCCGCCCTTAAGTGGTATTACCAGCCATTCTGGAGGAGCAGT
Oryza sativa Japonica             ACGGTCTATCCGCCCTTAAGTGGTATTACCAGCCATTCTGGAGGAGCAGT
RRIM600_cox1                      ACGGTCTATCCGCCCTTAAGTGGTATTACCAGCCATTCTGGAGGAGCAGT
RRII105_cox1                      ACGGTCTATCCGCCCTTAAGTGGTATTACCAGCCATTCTGGAGGAGCAGT
Cycas taitungensis                ACGGTCCATCCGCCCTTAAGTGGTATTACCAGTCATTCTGGAGGAGCTGC
Megaceros aenigmaticus            ACGGTCTATCCGCCTCTAAGTGGTATAACGAGTCATTCCGGAGGAGCCGT
Phaeoceros laevis                 ATGGTCTATTTGTTTCTAAGTGGTATAACGAGTTATTCCGGAGGAGCCGT
                                  * **** **  *    ********** ** **  **** ******** *

Brassica napus                    TGATTTAGCAATTTTTAGTCTTCATCTATCTGGTGTTTCATCCATTTTAG
PB235_cox1                        TGATTUAGCAATTTUTAGTCTTCATCTATCTGGTGTTTCATCCATTTTAG
Citrullus lanatus                 TGATTUAGCAATTTUTAGTCTTCATCTATCTGGTATTTCATCCATTTTAG
BPM24_cox1                        TGATTUAGCAATTTUTAGTCTTCATCTATCTGGTGTTTCATCCATTTTAG
RRIC110_cox1                      TGATTUAGCAATTTUTAGTCTTCATCTATCTGGTGTTTCATCCATTTTAG
Cucurbita pepo                    TGATTUAGCAATTTUTAGTCTTCATCTATCTGGTATTTCATCCATTTTAG
Lotus japonicus                   TGATTUAGCAATTTUTAGTCTTCATCTATCTGGTGTTTCATCCATTTTAG
Millettia pinnata                 TGATTUAGCAATTTUTAGTCTCCATCTATCTGGTGTTTCATCCATTTTAG
RRIT251_cox1                      TGATTUAGCAATTTUTAGTCTTCATCTATCTGGTGTTTCATCCATTTTAG
Oryza sativa Japonica             TGATTTAGCAATTTTTAGTCTTCATCTATCAGGTGTTTCATCAATTTTAG
RRIM600_cox1                      TGATTNAGCAATTTNTAGTCTTCATCTATCTGGTGTTTCATCCATTTTAG
RRII105_cox1                      TGATTUAGCAATTTUTAGTCTTCATCTATCTGGTGTTTCATCCATTTTAG
Cycas taitungensis                TGATTCAGCAATTTCTAGTCTTCATTTATCAGGTGTTTCATCCATTTCAG
Megaceros aenigmaticus            TGATTTAGCCATTTTTAGTCTTCATCTATCAGGTGTTTCACTTATTTCAG
Phaeoceros laevis                 TGATTTAGCAATTTTTAGTTCTTATTTATCAGGTGTTTTATTTATTTCAG
                                  ***** *** **** ****    ** **** *** *** *   **** **

Brassica napus                    GTTCTATCAATTTTATAACAACTATCTTCAACATGCGTGGACCTGGAATG
PB235_cox1                        GTTCTATCAATTTTATAACAACTATCTUCAACATGCGTGGACCTGGAATG
Citrullus lanatus                 GTTCTATCAATTTTATAACGACTATTTUCAACATGCGTGGACCTGGAATG
BPM24_cox1                        GTTCTATCAATTTTATAACAACTATCTUCAACATGCGTGGACCTGGAATG
RRIC110_cox1                      GTTCTATCAATTTTATAACAACTATCTUCAACATGCGTGGACCTGGAATG
Cucurbita pepo                    GTTCTATCAATTTTATAACGACTATTTUCAACATGCGTGGACCTGGAATG
Lotus japonicus                   GTTCTATCAATTTTATAACAACTATCTUCAACATGCGTGGACCTGGAATG
Millettia pinnata                 GTTCTATCAATTTTATAACAACTATCTUCAACATGCGTGGACCTGGAATG
RRIT251_cox1                      GTTCTATCAATTTTATAACAACTATCTUCAACATGCGTGGACCTGGAATG
Oryza sativa Japonica             GTTCTATCAATTTTATAACAACTATCTTCAACATGCGTGGACCTGGAATG
RRIM600_cox1                      GTTCTATCAATTTTATAACAACTATCTNCAACATGCGTGGACCTGGAATG
RRII105_cox1                      GTTCTATCAATTTTATAACAACTATCTUCAACATGCGTGGACCTGGAATG
Cycas taitungensis                GTTCTATCAATTCTATAACTACTATCCCCAACATGCGCGGACCTGGAATG
Megaceros aenigmaticus            GTGCTATTAATCTTATCAGTACTATCTTTAATATGCGCGGCCCTGGAATG
Phaeoceros laevis                 GTGCTATTAATTTTATCAGTACTATCTTTAATATGCGCGGCCCTGGAATG
                                  ** **** ***  *** *  *****    ** ***** ** *********

Brassica napus                    ACTATGCATAGATTACCCCTATTTGTGTGGTCCGTTCTAGTGACAGCATT
PB235_cox1                        ACTATGCATAGATUACCCCTATTTGTGTGGTCCGTTCTAGTGACAGCATT
Citrullus lanatus                 ACTATGCATAGATUACCCCTCTTTGTGTGGTCCGTTCTAGTGACAGCATT
BPM24_cox1                        ACTATGCATAGATUACCCCTATTTGTGTGGTCCGTTCTAGTGACAGCATT
RRIC110_cox1                      ACTATGCATAGATUACCCCTATTTGTGTGGTCCGTTCTAGTGACAGCATT
Cucurbita pepo                    ACTATGCATAGATUACCCCTCTTTGTCTGGTCCGTTCTAGTGACAGCATT
Lotus japonicus                   ACTATGCATAGATUACCCCTATTTGTGTGGTCCGTTCCAGTAACAGCATT
Millettia pinnata                 ACTATGCATAGATUACCCCTATTTGTGTGGTCCGTTCCAGTAACAGCATT
RRIT251_cox1                      ACTATGCATAGATUACCCCTATTTGTGTGGTCCGTTCTAGTGACAGCATT
Oryza sativa Japonica             ACTATGCATAGATTACCACTTTTTGTGTGGTCCGTTCTAGTGACAGCATT
RRIM600_cox1                      ACTATGCATAGATUACCCCTATTTGTGTGGTCCGTTCTAGTGACAGCATT
RRII105_cox1                      ACTATGCATAGATUACCCCTATTTGTGTGGTCCGTTCTAGTGACAGCATT
Cycas taitungensis                ACTATGCATAGATCACCCCTATTCGTGCGGTCCGTTCCAGTGACAGCATT
```

```
Megaceros aenigmaticus            ACTATGCATAAATTGCCTTTATTTGTGTGGCCTGTTTTAGTGACAGCATT
Phaeoceros laevis                 ACTATGCATAAATTGCTTTTATTTGTGTGGTTCGTTTTAGTGACAGCATT
                                  ********** **  *   * ** **  **   ***  *** ********

Brassica napus                    CCTACTTTTATTATCACTCCCGGTACTGGCAGGGGCAATTACCATGTTAT
PB235_cox1                        CCUACTTTTATTATCACTTCCGGTACTGGCAGGGGCAATTACCATGTTAT
Citrullus lanatus                 CCUACTTTTATTATCACTTCCGGTACTGGCAGGGGCTATTACCATGTTAT
BPM24_cox1                        CCUACTTTTATTATCACTTCCGGTACTGGCAGGGGCAATTACCATGTTAT
RRIC110_cox1                      CCUACTTTTATTATCACTTCCGGTACTGGCAGGGGCAATTACCATGTTAT
Cucurbita pepo                    CCUACTTTTATTATCACTTCCGGTACTGGCAGGGGCTATTACCATGTTAT
Lotus japonicus                   CCUACTTTTATTATCACTTCCGGTACTGGCAGGGGCAATTACAATGTTAT
Millettia pinnata                 CCUACTTTTATTATCACTTCCGGTACTGGCAGGGGCAATAACAATGTTAT
RRIT251_cox1                      CCUACTTTTATTATCACTTCCGGTACTGGCAGGGGCAATTACCATGTTAT
Oryza sativa Japonica             CCTACTTTTATTATCACTTCCGGTACTGGCAGGGGCAATTACAATGTTAT
RRIM600_cox1                      CCUACTTTTATTATCACTTCCGGTACTGGCAGGGGCAATTACCATGTTAT
RRII105_cox1                      CCUACTTTTATTATCACTTCCGGTACTGGCAGGGGCAATTACCATGTTAT
Cycas taitungensis                CCTACTTCTATTATCACTTCCGGTACCGGCAGGGGCAATTACCATGTTAT
Megaceros aenigmaticus            CCTACTCTTATTATCCCTTCCAGTACTGGCAGGTGCCATTACCATGTTAT
Phaeoceros laevis                 CTTACTTTTATTATTCTTTTAGTACTGGCAGGTGCCATTACCATGTTAT
                                  *  ***  ******   *    **** ****** ** ** ** *******

Brassica napus                    TAACCGATCGAAACTTTAATACAACCTTTTTTGATCCCGCTGGAGGGGGA
PB235_cox1                        TAACCGATCGAAACTTTAATACAACCTTTTTTGATCCCGCTGGAGGGGGA
Citrullus lanatus                 TAACCGATCGAAACTTTAATACAACCTTTTUTGATCCCGCTGGAGGGGGA
BPM24_cox1                        TAACCGATCGAAACTTTAATACAACCTTTTTTGATCCCGCTGGAGGGGGA
RRIC110_cox1                      TAACCGATCGAAACTTTAATACAACCTTTTTTGATCCCGCTGGAGGGGGA
Cucurbita pepo                    TAACCGATCGAAACTTTAATACAACCTTTTUTGATCCCGCTGGAGGGGGA
Lotus japonicus                   TAACCGATCGAAACTTTAATACAACCTTTTUTGATCCCGCAGGAGGGGGA
Millettia pinnata                 TAACCGATCGAAACTTTAATACAACCTTTTUTGATCCCGCAGGAGGGGGA
RRIT251_cox1                      TAACCGATCGAAACTTTAATACAACCTTTTTTGATCCCGCTGGAGGGGGA
Oryza sativa Japonica             TAACCGATCGAAACTTTAATACAACCTTTTTTGATCCTGCAGGAGGGGGA
RRIM600_cox1                      TAACCGATCGAAACTTTAATACAACCTTTTTTGATCCCGCTGGAGGGGGA
RRII105_cox1                      TAACCGATCGAAACTTTAATACAACCTTTTTTGATCCCGCTGGAGGGGGA
Cycas taitungensis                TGACCGATCGAAACTTTAATACAACCTTTTCTGATCCTGCTGGAGGGGGA
Megaceros aenigmaticus            TAACTGATAGAAACTTTAATACCACCTCTTTTGATCCCGCGGGAGGAGGA
Phaeoceros laevis                 TAACTGATAGAAACTTTAATACCACCTTTTTTGATCTCGCAGGAGGAGGA
                                  * ** *** ************* **** ** *****  ** ***** ***

Brassica napus                    GACCCAATTTTATACCAGCATCTCTTTTGGTTCTTCGGT-----------
PB235_cox1                        GACCCCATATTATACCAGCATCTCTTTTGGTTCTTCGGT-----------
Citrullus lanatus                 GACCCCATATTATACCAGCATCTCTTTTGGTTCTTCGGTTTTAAATGGCC
BPM24_cox1                        GACCCCATATTATACCAGCATCTCTTTTGGTTCTTCGGT-----------
RRIC110_cox1                      GACCCCATATTATACCAGCATCTCTTTTGGTTCTTCGGT-----------
Cucurbita pepo                    GACCCCATATTATACCAGCATCTCTTTUGGTTCTTCGGT-----------
Lotus japonicus                   GACCCCATATTATACCAGCATCTCTTTUGGTTCTTCGGT-----------
Millettia pinnata                 GACCCCATATTATACCAGCATCTCTTTUGGTTCTTCGGT-----------
RRIT251_cox1                      GACCCCATATTATACCAGCATCTCTTTTGGTTCTTCGGT-----------
Oryza sativa Japonica             GACCCAATATTATACCAGCATCTCTTTTGGTTCTTCGGT-----------
RRIM600_cox1                      GACCCCATATTATACCAGCATCTCTTTTGGTTCTTCGGT-----------
RRII105_cox1                      GACCCCATATTATACCAGCATCTCTTTTGGTTCTTCGGT-----------
Cycas taitungensis                GACCCGATATTATACCAGCATCTCTTTCGGTCCTTCGGT-----------
Megaceros aenigmaticus            GATCCGATTTTATACCAGCATCCATTTTGGTCTTTCGGT-----------
Phaeoceros laevis                 GATCTGATTTTATACCAGTATCTATTTTGGTTTTTTGGT-----------
                                  ** *  ** ********* ***  *** ***  ** ***

Brassica napus                    ------CATCCAGAGGTGTATATTCTCATTCTGCCTGGATTCGGTATCAT
PB235_cox1                        ------CACCCTGAAGTTTACATTCTAATTCTGCCGGGATUCGGTATCAT
Citrullus lanatus                 GAGAAGCACCCTGAAGTTTACATCCTAATTCTGCCTGGATUCGGTATCAT
BPM24_cox1                        ------CACCCTGAAGTTTACATTCTAATTCTGCCGGGATUCGGTATCAT
RRIC110_cox1                      ------CACCCTGAAGTTTACATTCTAATTCTGCCGGGATUCGGTATCAT
Cucurbita pepo                    ------CATCCAGAGGTGTATATTCUCATTCTGCCTGGATUCGGTATCAT
Lotus japonicus                   ------CATCCAGAGGTGTATATTCUCATTCTGCCTGGATUCGGTATCAT
```

```
Millettia pinnata                 ------CATCCAGAGGTGTATATTCUCATTCTGCCTGGATUCGGTATCAT
RRIT251_cox1                      ------CACCCTGAAGTTTACATTCTAATTCTGCCGGGATUCGGTATCAT
Oryza sativa Japonica             ------CATCCAGAGGTGTATATTCTCATTCTGCCTGGATTCGGTATTAT
RRIM600_cox1                      ------CACCCTGAAGTTTACATTCTAATTCTGCCGGGATNCGGTATCAT
RRII105_cox1                      ------CACCCTGAAGTTTACATTCTAATTCTGCCGGGATUCGGTATCAT
Cycas taitungensis                ------CATCCAGAGGTGCATATTCCCATTCCGCCCGGATCCGGTATCAT
Megaceros aenigmaticus            ------CATCCAGAGGTTCATATTCCAATTTTGCCAGGATTCGGTATAAT
Phaeoceros laevis                 ------TATCCAGAGGTTTATATTCTAATTTTGTCAGGATTCGGTATAAT
                                         * ** ** **  * ** *  ***  * * **** ****** **

Brassica napus                    AAGTCATATCGTTTCTACTTTTTCGGGAAAACCGGTCTTCGGGTATCTAG
PB235_cox1                        AAGTCATATCGTTTCGACTTTTTCGGGAAAACCGGTTTTCGGGTATCTAG
Citrullus lanatus                 AAGTCATATCGTTTCGACTTTTTCGGGAAAACCGGTTTTCGGGTATCTAG
BPM24_cox1                        AAGTCATATCGTTTCGACTTTTTCGGGAAAACCGGTTTTCGGGTATCTAG
RRIC110_cox1                      AAGTCATATCGTTTCGACTTTTTCGGGAAAACCGGTTTTCGGGTATCTAG
Cucurbita pepo                    CAGTCATATCGTTTCTACTTTTTCGGGAAAACCAGTTTTCGGGTATCTAG
Lotus japonicus                   AAGTCATATCGTTTCGACTTTTTCGGGAAAACCGGTCTTCGGGTATCTAG
Millettia pinnata                 AAGTCATATCGTTTCGACTTTTTCGGGAAAACCGGTCTTCGGGTATCTAG
RRIT251_cox1                      AAGTCATATCGTTTCGACTTTTTCGGGAAAACCGGTTTTCGGGTATCTAG
Oryza sativa Japonica             TAGTCATATCGTATCGACCTTTTCAAGAAAACCGGTCTTCGGGTATCTAG
RRIM600_cox1                      AAGTCATATCGTTTCGACTTTTTCGGGAAAACCGGTTTTCGGGTATCTAG
RRII105_cox1                      AAGTCATATCGTTTCGACTTTTTCGGGAAAACCGGTTTTCGGGTATCTAG
Cycas taitungensis                TAGTCATATCGTATCGACTTTTTCGGGAAAACCGGTCTTCGGGTATCTAG
Megaceros aenigmaticus            TAGTCATATCGTTTCCACGTTTCCAAGAAAACCTGTATTCGGTTATCTAG
Phaeoceros laevis                 TAGTCATATCGTTTTCACGTTTTTAAGAAAACTCGTATTCGGTTATCTAG
                                   *********** *  ** ***    ******  ** ***** *******

Brassica napus                    GCATGGTTTATGCCATGATCAGTATTGGTGTCTTAGGATTTCTTGTTTGG
PB235_cox1                        GCATGGTTTATGCCATGATCAGTATAGGTGTTCTTGGATTTCTTGTTTGG
Citrullus lanatus                 GCATGGTTTATGCCATGATCAGTATAGGTGTTCTTGGATTTCTTGTTTGG
BPM24_cox1                        GCATGGTTTATGCCATGATCAGTATAGGTGTTCTTGGATTTCTTGTTTGG
RRIC110_cox1                      GCATGGTTTATGCCATGATCAGTATAGGTGTTCTTGGATTTCTTGTTTGG
Cucurbita pepo                    GCATGGTTTATGCCATGATCAGTATAGGTGTTCTTGGATTTCTTGTTTGG
Lotus japonicus                   GCATGGTTTATGCCATGATCAGTATAGGTGTTCTTGGATTTCTTGTTTGG
Millettia pinnata                 GCATGGTTTATGCCATGATCAGTATAGGTGTTCTTGGATTTCTTGTTTGG
RRIT251_cox1                      GCATGGTTTATGCCATGATCAGTATAGGTGTTCTTGGATTTCTTGTTTGG
Oryza sativa Japonica             GCATGGTTTATGCCATGATAAGTATAGGTGTTCTTGGATTTCTAGTTTGG
RRIM600_cox1                      GCATGGTTTATGCCATGATCAGTATAGGTGTTCTTGGATTTCTTGTTTGG
RRII105_cox1                      GCATGGTTTATGCCATGATCAGTATAGGTGTTCTTGGATTTCTTGTTTGG
Cycas taitungensis                GCATGGTTTATGCCATGATCAGTATTGGTGTTCCTGGATCTCCTGTTCGG
Megaceros aenigmaticus            GCATGGTTTATGTCACGATCAGTATTGGAGTTCTCGGATTTATCGTGTGG
Phaeoceros laevis                 GCATGGTTTATGTTATGATCAGTATTGGAGTTCTCGGATTTATCGTGTGG
                                  ************  * *** ***** ** **    **** *   **  **

Brassica napus                    GCTCATCATATGTTTACTGTGGGCTTAGACGTAGATACCCGTGCCTACTT
PB235_cox1                        GCTCATCATATGTTTACTGTGGGCTTAGACGTTGATACCCGTGCCTACTT
Citrullus lanatus                 GCTCATCATATGTTTACTGTGGGCTTAGACGTTGATACCCGTGCCTACTT
BPM24_cox1                        GCTCATCATATGTTTACTGTGGGCTTAGACGTTGATACCCGTGCCTACTT
RRIC110_cox1                      GCTCATCATATGTTTACTGTGGGCTTAGACGTTGATACCCGTGCCTACTT
Cucurbita pepo                    GCTCATCATATGTTTACTGTGGGCTTAGACGTTGATACCCGTGCCTACTT
Lotus japonicus                   GCTCATCATATGTTTACTGTGGGCTTAGACGTTGATACCCGTGCCTACTT
Millettia pinnata                 GCTCATCATATGTTTACTGTGGGCTTAGACGTTGATACCCGTGCCTACTT
RRIT251_cox1                      GCTCATCATATGTTTACTGTGGGCTTAGACGTTGATACCCGTGCCTACTT
Oryza sativa Japonica             GCTCATCATATGTTTACTGTGGGCTTAGACGTTGATACGCGTGCCTACTT
RRIM600_cox1                      GCTCATCATATGTTTACTGTGGGCTTAGACGTTGATACCCGTGCCTACTT
RRII105_cox1                      GCTCATCATATGTTTACTGTGGGCTTAGACGTTGATACCCGTGCCTACTT
Cycas taitungensis                GCTCATCATATGTCTACTGTGGGCTCAGACGTTGATACGCGTGCTCACTC
Megaceros aenigmaticus            GCACACCACATGTTTACTGTAGGCTTAGACGTTGATACTCGTGCCTACTT
Phaeoceros laevis                 GCACACTACATGTTTACTGTAGGCTTAGACGTTGATACTTGTGCCTACTT
                                  ** **  * **** ****** **** ****** *****  ****  ***

Brassica napus                    CACCGCAGCTACCATGATCATAGCTGTCCCCACTGGAATCAAAATCTTTA
```

```
PB235_cox1                       CACCGCAGCTACCATGATCATAGCTGTCCCCACTGGAATCAAAATCTTTA
Citrullus lanatus                CACCGCTGCTACCATGATCATAGCAGTCCCCACTGGAATCAAAATCTTTA
BPM24_cox1                       CACCGCAGCTACCATGATCATAGCTGTCCCCACTGGAATCAAAATCTTTA
RRIC110_cox1                     CACCGCAGCTACCATGATCATAGCTGTCCCCACTGGAATCAAAATCTTTA
Cucurbita pepo                   CACCGCTGCTACCATGATCATAGCAGTCCCCACTGGAATCAAAATCTTTA
Lotus japonicus                  CACCGCAGCTACCATGATCATAGCTGTCCCCACTGGAATAAAAATATTTA
Millettia pinnata                CACCGCAGCTACCATGATCATAGCTGTCCCCACTGGAATTAAAATCTTTA
RRIT251_cox1                     CACCGCAGCTACCATGATCATAGCTGTCCCCACTGGAATCAAAATCTTTA
Oryza sativa Japonica            CACCGCAGCTACCATGATCATAGCTGTGCCCACTGGAATAAAAATCTTTA
RRIM600_cox1                     CACCGCAGCTACCATGATCATAGCTGTCCCCACTGGAATCAAAATCTTTA
RRII105_cox1                     CACCGCAGCTACCATGATCATAGCTGTCCCCACTGGAATCAAAATCTTTA
Cycas taitungensis               TACCGCAGCTACCATGATCATAGCTGTCCCCACTGGAATCAAAATCTCTA
Megaceros aenigmaticus           TACTGCGGCTACCATGATTACTGCTGTACCTACTGGAATTAAGATTTTTA
Phaeoceros laevis                TATTGCGGTTATCATGATTATTGTTGTGTCTACTGGAATTAAGATTCTTA
                                  *  ** * ** ****** *  *  **  * ******** ** **   **

Brassica napus                   GTTGGATCGCTACCATGTGGGGGGGTTCGATACAATACAAAACACCCATG
PB235_cox1                       GTTGGATCGCTACCATGTGGGGGGGTTCGATACAATACAAAACACCCATG
Citrullus lanatus                GTTGGATCGCTACCATGTGGGGGGGTTCGATACAATACAAAACACCCATG
BPM24_cox1                       GTTGGATCGCTACCATGTGGGGGGGTTCGATACAATACAAAACACCCATG
RRIC110_cox1                     GTTGGATCGCTACCATGTGGGGGGGTTCGATACAATACAAAACACCCATG
Cucurbita pepo                   GTTGGATCGCTACCATGTGGGGGGGTTCGATACAATACAAAACCCCCATG
Lotus japonicus                  GTTGGATCGCTACCATGTGGGGGGGTTCGATACAATACAAAACACCCATG
Millettia pinnata                GTTGGATCGCTACCATGTGGGGGGGTTCGATACAATACAAAACACCCATG
RRIT251_cox1                     GTTGGATCGCTACCATGTGGGGGGGTTCGATACAATACAAAACACCCATG
Oryza sativa Japonica            GTTGGATTGCTACCATGTGGGGAGGTTCGATACAATACAAAACACCCATG
RRIM600_cox1                     GTTGGATCGCTACCATGTGGGGGGGTTCGATACAATACAAAACACCCATG
RRII105_cox1                     GTTGGATCGCTACCATGTGGGGGGGTTCGATACAATACAAAACACCCATG
Cycas taitungensis               GTCGGATCGCTACCATGTGGGGAGGTTCGATACGATACAAAACACCCATG
Megaceros aenigmaticus           GTTGGGGCGCTACTATGTGGGGGGGTTCGATAGAGTACACCTCACCTCTT
Phaeoceros laevis                GTTGGGGCGTTATTATGTGGGGGGGTTCGATAGAGTACACCTCACTTCTT
                                 ** **   * **  ******** *********   ****   * *   *

Brassica napus                   TTATTTGCTGTAGGATTTATCTTTTTGTTCACCATAGGAGGACTCACTGG
PB235_cox1                       TTATTTGCTGTAGGGTTUATCTTTTTGTTCACCATAGGAGGACTCACTGG
Citrullus lanatus                TTATTTGCTGTAGGGTUCATCTTTTTGTTCACCATAGGAGGACTCACTGG
BPM24_cox1                       TTATTTGCTGTAGGGTTUATCTTTTTGTTCACCATAGGAGGACTCACTGG
RRIC110_cox1                     TTATTTGCTGTAGGGTTUATCTTTTTGTTCACCATAGGAGGACTCACTGG
Cucurbita pepo                   TTATTTGCTGTAGGGTTCATCTTTTTGTTCACCATAGGAGGACTCACTGG
Lotus japonicus                  TTATTTGCTGTAGGGTTCATCTTTTTGTTCACCATAGGAGGACTCACTGG
Millettia pinnata                TTATTTGCTGTAGGGTTCATCTTTTTGTTCACCATAGGAGGACTCACTGG
RRIT251_cox1                     TTATTTGCTGTAGGGTTUATCTTTTTGTTCACCATAGGAGGACTCACTGG
Oryza sativa Japonica            TTATTTGCTGTAGGGTTCATCTTTTTGTTCACCATAGGAGGGCTCACTGG
RRIM600_cox1                     TTATTTGCTGTAGGGTTUATCTTTTTGTTCACCATAGGAGGACTCACTGG
RRII105_cox1                     TTATTTGCTGTAGGGTTUATCTTTTTGTTCACCATAGGAGGACTCACTGG
Cycas taitungensis               TTACCTGCTGTAGGGTCTATCTCTTCGTCCACCATAGGAGGACCCACTGG
Megaceros aenigmaticus           TTTTTTGCAGTAGGTCTTATTTTCTTACTCACCGTGGGAGGCTGTACTGG
Phaeoceros laevis                TTTTTTGCAGTAGGTTTTATTTTCTTATTTACTGTGGGAGGCTGTATTGG
                                 **   *** *****    ** *  *     **  * *****    * ***

Brassica napus                   AATAGTCCTGGCAAATTCAGGGCTAGACATTGCTCTACATGATACTTATT
PB235_cox1                       AATAGTCCUGGCAAATTCTGGGCTAGACATTGCTCTACATGATACTTATT
Citrullus lanatus                AATAGTCCUGGCAAATTCAGGGCTAGACATTGCTCTACATGATACTTATT
BPM24_cox1                       AATAGTCCUGGCAAATTCTGGGCTAGACATTGCTCTACATGATACTTATT
RRIC110_cox1                     AATAGTCCUGGCAAATTCTGGGCTAGACATTGCTCTACATGATACTTATT
Cucurbita pepo                   AATAGTCCUGGCAAATTCAGGGCTAGACATTGCTCTACATGATACTTATT
Lotus japonicus                  AATAGTCCUGGCAAATTCTGGGCTAGACATTGCTCTACATGATACTTATT
Millettia pinnata                AATAGTCCUGGCAAATTCTGGGCTAGACATTGCTCTACATGATACTTATT
RRIT251_cox1                     AATAGTCCUGGCAAATTCTGGGCTAGACATTGCTCTACATGATACTTATT
Oryza sativa Japonica            AATTGTTCTAGCAAACTCTGGGCTAGACATTGCTCTACATGATACTTATT
RRIM600_cox1                     AATAGTCCUGGCAAATTCTGGGCTAGACATTGCTCTACATGATACTTATT
RRII105_cox1                     AATAGTCCUGGCAAATTCTGGGCTAGACATTGCTCTACATGATACTTATT
```

```
Cycas taitungensis                AATAGTCCCGGCAAATTCTGGGCTAGACATTGCTCCACATGATACTTATT
Megaceros aenigmaticus            AATAGTATTGGCCAATTCCGGGCTGGACATTGCTCCACATGATACTTATT
Phaeoceros laevis                 AACAGTATCGGCCAATTCCGGGCTGGACATTGCTCTACATGATACTTATT
                                  **  **    ** ** ** ***** ********** **************

Brassica napus                    ATGTGGTT-GCACATTTCCATTATGTACTTTCTATGGGAGCCGTTTTTGC
PB235_cox1                        ATGTGGTT-GCACATTTCCATTATGTACTTTCTATGGGAGCCGTTTTTGC
Citrullus lanatus                 ATGTGGTT-GCACATTTCCATTATGTACTTTCTATGGGAGCCGTTTTTGC
BPM24_cox1                        ATGTGGTT-GCACATTTCCATTATGTACTTTCTATGGGAGCCGTTTTTGC
RRIC110_cox1                      ATGTGGTT-GCACATTTCCATTATGTACTTTCTATGGGAGCCGTTTTTGC
Cucurbita pepo                    ATGTGGTT-GCGCATTTCCATTATGTACTTTCTATGGGAGCCGTTTTTGC
Lotus japonicus                   ATGTGGTT-GCACATTTCCATTATGTACTTTCTATGGGAGCCGTTTTTGC
Millettia pinnata                 ATGTGGTT-GCACATTTCCATTATGTACTTTCTATGGGAGCCGTTTTTGC
RRIT251_cox1                      ATGTGGTT-GCACATTTCCATTATGTACTTTCTATGGGAGCCGTTTTTGC
Oryza sativa Japonica             ATGTGGTTCGCACATTTCCATTATGTACTTTCTATGGGAGCCGTTTTTGC
RRIM600_cox1                      ATGTGGTT-GCACATTTCCATTATGTACTTTCTATGGGAGCCGTTTTTGC
RRII105_cox1                      ATGTGGTT-GCACATTTCCATTATGTACTTTCTATGGGAGCCGTTTTTGC
Cycas taitungensis                ATGTGGTT-GCACATTCCCATTATGTACTTTCTATGGGAGCCGTTTTTGC
Megaceros aenigmaticus            ACGTGGTT-GCACATCTTCATTATGTTCTTCCTATGGGAGCCGTTTCTGC
Phaeoceros laevis                 ACGTGGTT-GCATATTTTTATTATGTTTCTTTTATGGGAGCCGTTTTTGT
                                  * ****** **  **    *******   *  ************** **

Brassica napus                    TTTATTTGCAGGATTTTACTATTGGGTGGGTAAAATCTTTGGTCGGACAT
PB235_cox1                        TTTATTTGCAGGATTTUACTATTGGGTGGGTAAAATCTTTGGTCGGACAT
Citrullus lanatus                 TTTATTTGCAGGATTTUACTATTGGGTGGGTAAAATCTTTGGTCGGACAT
BPM24_cox1                        TTTATTTGCAGGATTTUACTATTGGGTGGGTAAAATCTTTGGTCGGACAT
RRIC110_cox1                      TTTATTTGCAGGATTTUACTATTGGGTGGGTAAAATCTTTGGTCGGACAT
Cucurbita pepo                    TTTATTTGCAGGATTTUACTATTGGGTGGGTAAAATCTTTGGTCGGACAT
Lotus japonicus                   TTTATTTGCAGGATTTUACTATTGGGTGGGTAAAATCTTTGGTCGGACAT
Millettia pinnata                 TTTATTTGCAGGATTTUACTATTGGGTGGGTAAAATCTTTGGTCGGACAT
RRIT251_cox1                      TTTATTTGCAGGATTTUACTATTGGGTGGGTAAAATCTTTGGTCGGACAT
Oryza sativa Japonica             TTTATTTGCTGGATTTTACTATTGGGTGGGTAAAATCTTTGGTCGGACAT
RRIM600_cox1                      TTTATTTGCAGGATTTUACTATTGGGTGGGTAAAATCTTTGGTCGGACAT
RRII105_cox1                      TTTATTTGCAGGATTTUACTATTGGGTGGGTAAAATCTTTGGTCGGACAT
Cycas taitungensis                TCCATTTGCAGGATCTCACTCTCGGGCGGGTAAAATCTCTGGTCGAACAT
Megaceros aenigmaticus            TTTATTTGCAGGATTTTATTATTGGATAGGTAAAATGACGGGTCTCCAAT
Phaeoceros laevis                 TTTATTTGCAGGATTTTATTATTGGATAGGTAAAATAACAGGTCTCCAAT
                                  *  ****** **** * * * * **   ********    ****    **

Brassica napus                    ACCCTGAAACTTTAGGTCAAATCCATTTTTGGATCACTTTTTTCGGGGTT
PB235_cox1                        ACCCTGAAACTTTAGGTCAAATCCATTTTTGGATCACTTTTTTCGGGGTT
Citrullus lanatus                 ACCCTGAAACTTTAGGTCAAATCCATTTTTGGATCACTTTTTTCGGGGTT
BPM24_cox1                        ACCCTGAAACTTTAGGTCAAATCCATTTTTGGATCACTTTTTTCGGGGTT
RRIC110_cox1                      ACCCTGAAACTTTAGGTCAAATCCATTTTTGGATCACTTTTTTCGGGGTT
Cucurbita pepo                    ACCCTGAGACTTTAGGGCAAATCCATTTTTGGATCACTTTTTTCGGGGTT
Lotus japonicus                   ACCCTGAAACTTTAGGTCAAATCCATTTTTGGATCACTTTTTTCGGGGTT
Millettia pinnata                 ACCCTGAAACTTTAGGTCAAATCCATTTTTGGATCACTTTTTTCGGGGTT
RRIT251_cox1                      ACCCTGAAACTTTAGGTCAAATCCATTTTTGGATCACTTTTTTCGGGGTT
Oryza sativa Japonica             ATCCTGAAACTTTAGGCCAAATCCATTTTTGGATTACTTTTTTCGGGGTT
RRIM600_cox1                      ACCCTGAAACTTTAGGTCAAATCCATTTTTGGATCACTTTTTTCGGGGTT
RRII105_cox1                      ACCCTGAAACTTTAGGTCAAATCCATTTTTGGATCACTTTTTTCGGGGTT
Cycas taitungensis                ACCCTGAAACTTTAGGTCAAATCCATTTCCGGATCACTTCTTTCGGGGTT
Megaceros aenigmaticus            ATCCAGAGACTTTAGGTCTAATACATTTTTGGATTACTTTCTTTGGTGTT
Phaeoceros laevis                 ATTTAGAGATTTTAGGTCTAATATATTTTTGGATTACTTTCTTTGGTGTC
                                  *    ** * ****** * ***  ****  **** ****  ** ** **

Brassica napus                    AATTTGACCTTCTTTCCTATGCATTTCTTAGGGCTTTCAGGTATGCCACG
PB235_cox1                        AATCUGACCTTCTTTCCUATGCATTTCTTAGGGCTTTCGGGTATGCCACG
Citrullus lanatus                 AATCUGACCUTCTTTCCCATGCATTTCTTAGGGCTTTCGGGTATGCCACG
BPM24_cox1                        AATCUGACCTTCTTTCCUATGCATTTCTTAGGGCTTTCGGGTATGCCACG
RRIC110_cox1                      AATCUGACCTTCTTTCCUATGCATTTCTTAGGGCTTTCGGGTATGCCACG
Cucurbita pepo                    AATCUGACCUTCTTTCCCATGCATTTCCTAGGGCTTTCGGGTATGCCACG
```

```
Lotus japonicus               AATCTGACCCTCTTTCCCATGCATTTCTTAGGGCTTTCGGGTATGCCACG
Millettia pinnata             AATCTGACCCTCTTTCCCATGCATTTCTTAGGGCTTTCGGGTATGCCACG
RRIT251_cox1                  AATCUGACCTTCTTTCCUATGCATTTCTTAGGGCTTTCGGGTATGCCACG
Oryza sativa Japonica         AATCTGACCTTCTTTCCCATGCATTTCTTAGGGCTTTCGGGCATGCCACG
RRIM600_cox1                  AATCUGACCTTCTTTCCNATGCATTTCTTAGGGCTTTCGGGTATGCCACG
RRII105_cox1                  AATCUGACCTTCTTTCCUATGCATTTCTTAGGGCTTTCGGGTATGCCACG
Cycas taitungensis            AATTCGACCTTCTTTCCCATGCATTTCTTGGGGCTTTCGGGTATGCCACG
Megaceros aenigmaticus        AACTTAACTTCCTTCCCAACGTATTTAGAACCGCTTGCAGGTATGCCACG
Phaeoceros laevis             AACCTAACTTCCTTTCTAATGCATTTAGAACCGCTTGCAGGTATGCCACG
                              **    **   *** *  * * ****      **** * ** ********

Brassica napus                TCGTATTCCAGATTATCCAGATGCTTACGCTGGATGGAATGCCCTTTCCA
PB235_cox1                    TCGCATTCCAGATTATCCAGATGCTTACGCTGGATGGAATGCCCTTAGCA
Citrullus lanatus             TCGCATTCCTGATTATCCAGATGCTTACGCTGGATGGAATGCCCTTAGCA
BPM24_cox1                    TCGCATTCCAGATTATCCAGATGCTTACGCTGGATGGAATGCCCTTAGCA
RRIC110_cox1                  TCGCATTCCAGATTATCCAGATGCTTACGCTGGATGGAATGCCCTTAGCA
Cucurbita pepo                GCGCATTCCAGATTATCCAGATGCTTACGCTGGATGGAATGCCCTTAGCA
Lotus japonicus               TCGCATTCCAGATTATCCAGATGCTTACGCTGGATGGAATGCCCTTAGCA
Millettia pinnata             TCGCATTCCAGATTATCCAGATGCTTACGCTGGATGGAATGCCCTTAGCA
RRIT251_cox1                  TCGCATTCCAGATTATCCAGATGCTTACGCTGGATGGAATGCCCTTAGCA
Oryza sativa Japonica         TCGCATTCCAGATTATCCAGATGCTTACGCCGGATGGAATGCTCTGAGCA
RRIM600_cox1                  TCGCATTCCAGATTATCCAGATGCTTACGCTGGATGGAATGCCCTTAGCA
RRII105_cox1                  TCGCATTCCAGATTATCCAGATGCTTACGCTGGATGGAATGCCCTTAGCA
Cycas taitungensis            TCGCATTCCAGATTATCCAGATGCTTACGCTGGATGGAATGCCCTTAGCA
Megaceros aenigmaticus        TCGCATTCCAGATTATCCAGATGCTTACGCCGGGTGGAATGCCTTTAGTA
Phaeoceros laevis             TCGCATTCCAGATTATCCGGATGCTTACGCCGGGTGGAATGCCTTTAGTA
                               ** ***** ******** *********** ** ********  *    *

Brassica napus                GTTTTGGCTCTTATATATCCGTAGTTGGGATTTGTTGTTTCTTCGTGGTC
PB235_cox1                    GTTTTGGCTCTTATATATCUGTAGTTGGGATTNGTNGTTTCTTCGTGGTC
Citrullus lanatus             GTTTTGGCTCTTATATATCCGTAGTTGGGATTUGTUGTTTCTTCGTGGTC
BPM24_cox1                    GTTTTGGCTCTTATATATCCGTAGTTGGGATTUGTUGTTTCTTCGTGGTC
RRIC110_cox1                  GTTTTGGCTCTTATATATCUGTAGTTGGGATTUGTNGTTTCTTCGTGGTC
Cucurbita pepo                GTTTTGGCTCTTATATATCCGTAGTTGGGATTUGTUGTTTCTTCGTGGTC
Lotus japonicus               GTTTTGGCTCTTATATATCCGTAGTTGGGATTUGTUGTTTCTTCGTGGTC
Millettia pinnata             GTTTTGGCTCTTATATATCCGTAGTTGGGATTUGTUGTTTCTTCGTGGTC
RRIT251_cox1                  GTTTTGGCTCTTATATATCUGTAGTTGGGATTUGTUGTTTCTTCGTGGTC
Oryza sativa Japonica         GTTTCGGTTCTTATATATCCGTAGTTGGGATTUGTUGTTTCTTCGTAGTT
RRIM600_cox1                  GTTTTGGCTCTTATATATCCGTAGTTGGGATTUGTUGTTTCTTCGTGGTC
RRII105_cox1                  GTTTTGGCTCTTATATATCUGTAGTTGGGATTUGTUGTTTCTTCGTGGTC
Cycas taitungensis            GTTTCGGCCCTTATATATCCGTAGTTGGGATTCGTCGTTTCTTCGTGGTC
Megaceros aenigmaticus        GTTTTGGCTCATACGTTTCTGTAGTAGGGATTTCCCGTCCTTCCGTAGTG
Phaeoceros laevis             GTTTCGGCTTATACGTTTTTGTAGTAGGGATTTCCCGTTCTTTCGTAGTT
                              **** **    **  * *  ***** ******    **   * *** **

Brassica napus                GTAACAATCACTTTAAGCAGTGGAAATAACAAAAGATGTGCTCCTAGTCC
PB235_cox1                    GTAACAATCACTTUAAGCAGTGGAAATAACAAAAGATGTGCTCCAAGTCC
Citrullus lanatus             GTAACAATCACTTUAAGCAGTGGAAATAACAAAAGATGTGCTCCAAGTCC
BPM24_cox1                    GTAACAATCACTTUAAGCAGTGGAAATAACAAAAGATGTGCTCCAAGTCC
RRIC110_cox1                  GTAACAATCACTTUAAGCAGTGGAAATAACAAAAGATGTGCTCCAAGTCC
Cucurbita pepo                GTCACAATCACTTUAAGCAGTGGAAATAACAAAAGATGTGCTCCAAGTCC
Lotus japonicus               GTCACAATCACTTUAAGCAGTGGAAATAACATAACAAGGGCGAACATTCC
Millettia pinnata             GTAACAATCACTTUAAGCAGTGGAAATAACATAACAAGGGCGAACATTCC
RRIT251_cox1                  GTAACAATCACTTUAAGCAGTGGAAATAACAAAAGATGTGCTCCAAGTCC
Oryza sativa Japonica         GTCGCAATCACTTUAAGCAGTGGAAAGAACAAAAGATGTGCGGAAAGTCC
RRIM600_cox1                  GTAACAATCACTTUAAGCAGTGGAAATAACAAAAGATGTGCTCCAAGTCC
RRII105_cox1                  GTAACAATCACTTUAAGCAGTGGAAATAACAAAAGATGTGCTCCAAGTCC
Cycas taitungensis            GTAACAATCACTTTAAGCAGTGGAAACAACAAAAGATGCGCTCCAAGTCC
Megaceros aenigmaticus        ATTTTTCTTACCCCAACCGGTGAAAACAA------GTATGCTCCAAGTCC
Phaeoceros laevis             ATTTTTCCCACCCCAACCGGTGAAAACAA------GTATGCTCCAAGTCC
                               *       **   ** * *** *** **          **    * ***
```

```
Brassica napus                    TTGGGCTCTTGAACTTAATTCAACTACACTGGAATGGATGGTACAAAGTC
PB235_cox1                        TTGGGCTGTTGAACAGAATUCAACCACACTGGAATGGATGGTACAAAGTC
Citrullus lanatus                 TTGGGCTGTTGAACAGAATTCAACCACACTGGAATGGATGGTACAAAGTC
BPM24_cox1                        TTGGGCTGTTGAACAGAATUCAACCACACTGGAATGGATGGTACAAAGTC
RRIC110_cox1                      TTGGGCTGTTGAACAGAATUCAACCACACTGGAATGGATGGTACAAAGTC
Cucurbita pepo                    TTGGGCTGTTGAACAGAATTCAACCACACTGGAATGGATGGTACAAAGTC
Lotus japonicus                   TTGGGCTGTTGAAGAAAATTCAACCACACTGGAATGGCTGGTACAAAGTC
Millettia pinnata                 TTGGGCTGTTGAACAAAATTCAACCACACTGGAATGGCTGGTACAAAGTC
RRIT251_cox1                      TTGGGCTGTTGAACAGAATUCAACCACACTGGAATGGATGGTACAAAGTC
Oryza sativa Japonica             TTGGGCTGTTGAACAGAATUCAACCACACTAGAATGGTTGGTACAAAGCC
RRIM600_cox1                      TTGGGCTGTTGAACAGAATNCAACCACACTGGAATGGATGGTACAAAGTC
RRII105_cox1                      TTGGGCTGTTGAACAGAATUCAACCACACTGGAATGGATGGTACAAAGTC
Cycas taitungensis                CTGGGCTGTTGAACATAATCCAACCACACCGGAATGGATGGTACAAAGCC
Megaceros aenigmaticus            TTGGGCTGTTGAACAGAATTCAACGACACCTGAATGGATGGTACAAAGCC
Phaeoceros laevis                 TTGGGCTGTTGAACAGAATTCAACGACATTTGAATGGATGGTACAAAGCC
                                   ****** *****   *** **** ***   ****** ********** *

Brassica napus                    CTCCTGCTTTTCATACTTTTGGAGAACTTCCAGCTATCAAGGAGAC----
PB235_cox1                        CTCCAGCTTTTCATACTTTTGGAGAACTTCCAGCTATCAAGGAGAC----
Citrullus lanatus                 CTCCAGCTTTTCATACTTTTGGAGAACTTCCTGCTATCAAGGAGAC----
BPM24_cox1                        CTCCAGCTTTTCATACTTTTGGAGAACTTCCAGCTATCAAGGAGAC----
RRIC110_cox1                      CTCCAGCTTTTCATACTTTTGGAGAACTTCCAGCTATCAAGGAGAC----
Cucurbita pepo                    CTCCTGCTTTTCATACTTTTGGAGAACTTCCAGCTATCAAGGAGAC----
Lotus japonicus                   CTCCAGCTTTTCATACTTTTGGAGAACTTCCAGCTATCAAGGAGAC----
Millettia pinnata                 CTCCAGCTTTTCATACTTTTGGAGAACTTCCTGCTATCAAGGAGAC----
RRIT251_cox1                      CTCCAGCTTTTCATACTTTTGGAGAACTTCCAGCTATCAAGGAGAC----
Oryza sativa Japonica             CTCCAGCCTTTCATACTTTTGGAGAACTTCCAGCTATCAAAGAGAC----
RRIM600_cox1                      CTCCAGCTTTTCATACTTTTGGAGAACTTCCAGCTATCAAGGAGAC----
RRII105_cox1                      CTCCAGCTTTTCATACTTTTGGAGAACTTCCAGCTATCAAGGAGAC----
Cycas taitungensis                CTCCAGCTTTTCATACTTCTGAAGAACTTCCAGCTGTCAAGGAGACCTTG
Megaceros aenigmaticus            CTCCAGCATTTCATACCTTTGAAGAAATTCCAGCTATAAAAGAAAGTATT
Phaeoceros laevis                 TTCCAGCATTTCATACCTTTGAAGAAATTTCAGCTATAAAAGAAAGTATT
                                   *** ** ******** * ** **** ** * *** * ** ** *

Brassica napus                    -GAAAAGCTATGTGAAGTAAAAGAAGAAAAGGTCGCCGACTGCTACTAAG
PB235_cox1                        -GAAAAGCTATGTGAAGTAA------------------------------
Citrullus lanatus                 -GAAAAGCTATGTGAAGTAA------------------------------
BPM24_cox1                        -GAAAAGCTATGTGAAGTAA------------------------------
RRIC110_cox1                      -GAAAAGCTATGTGAAGTAA------------------------------
Cucurbita pepo                    -GAAAAGCTATGTGAAGTAAAAGAAGAAAAAGTCGACGACTGCTACTAAG
Lotus japonicus                   -GAAAAGCTATGTGAAGTAAAAGAAGAAAAGGTCGCCGACTGCTACTAAG
Millettia pinnata                 -GAAAAGCTATGTGAAGTAAAAGAAGAAAAGGTCGCCGACTGCTACTAAG
RRIT251_cox1                      -GAAAAGCTATGTGAAGTAA------------------------------
Oryza sativa Japonica             -AAAAAGCTAGGCGCACCTCTCCTAACTATTGCCCATAAAAAATAATATA
RRIM600_cox1                      -GAAAAGCTATGTGAAGTAA------------------------------
RRII105_cox1                      -GAAAAGCTATGTGAAGTAA------------------------------
Cycas taitungensis                TGAGAAGCTATGTAAAGTAAAAGAAGAAACGGTCGCCGGCTGCCACTAAG
Megaceros aenigmaticus            TAG-----------------------------------------------
Phaeoceros laevis                 TAG-----------------------------------------------


Brassica napus                    ---AACCTAACAGAACTTTTATAAATTTCTGCATTGAGATTCCGTAAGTA
PB235_cox1                        --------------------------------------------------
Citrullus lanatus                 --------------------------------------------------
BPM24_cox1                        --------------------------------------------------
RRIC110_cox1                      --------------------------------------------------
Cucurbita pepo                    ---AACCTAACAGAACTTTTCCAAATGTGGGTTCTAAAACCACTTGTCTA
Lotus japonicus                   ---AACCTAACAGAACTTTTCTAAATGTGGGTTCCAACGAAGAAGAGTTG
Millettia pinnata                 ---AACCTAACAGAACTTTTCAAATGTGGGTTCCAACGAAGAAGAGTTG
RRIT251_cox1                      --------------------------------------------------
Oryza sativa Japonica             TTTAATATTATTATATATTATATATATTTGCCCTTATAAAATAATATATT
RRIM600_cox1                      --------------------------------------------------
```

```
RRII105_cox1                      --------------------------------------------------
Cycas taitungensis                ---AACCTAACAGAACTTTTCCAAATGTGGGTTATTATTGTTATTATTAC
Megaceros aenigmaticus            --------------------------------------------------
Phaeoceros laevis                 --------------------------------------------------


Brassica napus                    ACTCAGTGAGTGCTTTCTAAGAAAGAAGGGCAAGGAAGAAGAAAATGAAA
PB235_cox1                        --------------------------------------------------
Citrullus lanatus                 --------------------------------------------------
BPM24_cox1                        --------------------------------------------------
RRIC110_cox1                      --------------------------------------------------
Cucurbita pepo                    -------GGGCGGCCTTGAGAATTGGGGGGCCAACGAGGCTGGGGCCGGA
Lotus japonicus                   AG-----GACCAACTTCGACCTAATTTAAGAGTTAAGAAAGCAAGCTCAG
Millettia pinnata                 AG-----GACCAACTTCGACCTAATTTAAGAGTTAAGAAAGCAAGCTCAG
RRIT251_cox1                      --------------------------------------------------
Oryza sativa Japonica             TAATATATATTAATTCAATTTTCTATTGTGTGAAGTAAAAGAAGAAAAGG
RRIM600_cox1                      --------------------------------------------------
RRII105_cox1                      --------------------------------------------------
Cycas taitungensis                TATCA--TCATGATCATTGTGTGGGTCGGGGTCTGGATCTTTTTGGCCGG
Megaceros aenigmaticus            --------------------------------------------------
Phaeoceros laevis                 --------------------------------------------------


Brassica napus                    TAGGAACAACCGCGCTGGTCGTAATAGATCGACTTTCATGCTGAAGCAAG
PB235_cox1                        --------------------------------------------------
Citrullus lanatus                 --------------------------------------------------
BPM24_cox1                        --------------------------------------------------
RRIC110_cox1                      --------------------------------------------------
Cucurbita pepo                    TTTGACAGCAATGACCGGTCAAGCAAGGTCAATCAAAATGGATGGGGAAA
Lotus japonicus                   TTCAGAATGGCTACTTACCAACAGAGGATAGCGAGGTACTACAAGGCCAG
Millettia pinnata                 TTCAGAATGGCTACTTACCAACAGAGGATAGCGAGGTACTACAAGGCCAG
RRIT251_cox1                      --------------------------------------------------
Oryza sativa Japonica             TCTAGGCTCCGTCATGACGGAATCTAAGATAAGAACCTAACAGAACGTAA
RRIM600_cox1                      --------------------------------------------------
RRII105_cox1                      --------------------------------------------------
Cycas taitungensis                CCCAGA-TCTGTCTCCGATAGTGCTATCCCGAGATACCAATACTGTCGGG
Megaceros aenigmaticus            --------------------------------------------------
Phaeoceros laevis                 --------------------------------------------------


Brassica napus                    AACTAGCATGAAAGTTCCATTTCAGGGAAGGACGACGTACCCATGATACT
PB235_cox1                        --------------------------------------------------
Citrullus lanatus                 --------------------------------------------------
BPM24_cox1                        --------------------------------------------------
RRIC110_cox1                      --------------------------------------------------
Cucurbita pepo                    AACAAAGAGATGATTCTCAGCATAGAAGAGATCTTAACAAGGCAGATCAC
Lotus japonicus                   AGTAAAATTAGGAAATTTGATCGAGCTGAGCCGGAGCTGCTCCTACTGAC
Millettia pinnata                 AGTTAAATTTGGAAATTTGATCGAGCTGAGCCGGAGCTGCTCCTACTGAC
RRIT251_cox1                      --------------------------------------------------
Oryza sativa Japonica             AAAATGAAAAAAGATTCTAGACTGTTAATGTCTTGGCTCTGGAAGG----
RRIM600_cox1                      --------------------------------------------------
RRII105_cox1                      --------------------------------------------------
Cycas taitungensis                CACCTCTGAAGAGCTGCCGAAATGCAGCGAAACAGCCTTTCATGTA----
Megaceros aenigmaticus            --------------------------------------------------
Phaeoceros laevis                 --------------------------------------------------


Brassica napus                    TTCTGTTTTGT-----
PB235_cox1                        ----------------
Citrullus lanatus                 ----------------
BPM24_cox1                        ----------------
RRIC110_cox1                      ----------------
```

```
Cucurbita pepo                    CATCTCTAGAAGGATT
Lotus japonicus                   TATGACTGGACAGC--
Millettia pinnata                 TATGACTGGACAGC--
RRIT251_cox1                      ----------------
Oryza sativa Japonica             ----------------
RRIM600_cox1                      ----------------
RRII105_cox1                      ----------------
Cycas taitungensis                ----------------
Megaceros aenigmaticus            ----------------
Phaeoceros laevis                 ----------------
```

###################

cox2 multiple sequence alignment

```
Beta macrocarpa                   ------ATGATTGTTCGAGAATGGCTATTCTTCACAATGGCTCCTTGTGA
Beta vulgaris maritima            ------ATGATTGTTCGAGAATGGCTATTCTTCACAATGGCTCCTTGTGA
Beta vulgaris vulgaris            ------ATGATTGTTCGAGAATGGCTATTCTTCACAATGGCTCCTTGTGA
Silene latifolia                  ------ATGATTGTGATAGAATGGCTATTC---ACAATTGCTCCTTGTGA
Arabidopsis thaliana              ------ATGATTGTTCTAAAATGGTTATTUUTUACAATTTCTCCTTGTGA
Brassica napus                    ------ATGATTGTTCTAAAATGGTTATTCUTUACAATTTCTCCTTGTGA
PB235_cox2                        ------ATGATTGTTCTAGAATGGTTATTCCTUACAATTGCTCCTTGTGA
RRIT251_cox2                      ------ATGATTGTTCTAGAATGGTTATTCCTUACAATTGCTCCTTGTGA
RRIC110_cox2                      ------ATGATTGTTCTAGAATGGTTATTCCTUACAATTGCTCCTTGTGA
RRII105_cox2                      ------ATGATTGTTCTAGAATGGTTATTCCTUACAATTGCTCCTTGTGA
BPM24_cox2                        ------ATGATTGTTCTAGAATGGTTATTCCTUACAATTGCTCCTTGTGA
RRIM600_cox2                      ------ATGATTGTTCTAGAATGGTTATTCCTUACAATTGCTCCTTGTGA
Citrullus lanatus                 ------ATGATTGTTCTAAAATGGCTATTCCTCACAATTGCTCCTTGTGA
Cucurbita pepo                    ------ATGATTGTTCTCAAATGGCTATTCCTCACAATTGCTCUTTGTGA
Lotus japonicus                   ---------ATGAAATTAGAATGGCTATTCCTCACGATTGCTCCTTGTGA
Millettia pinnata                 ---------GTTAAAGACGAATGGCTATTCCTCACGAAAGCTCCTTGTGA
Oryza sativa Japonica             ATGATTCTTCGTTUATTAGAATGTCGATTUCTUACAATCGCTCTTTGTGA
Zea mays mays                     ATGATTCTTCGTTUATTAGAATGTCGATTCUTCACAATCGCTCTTTGTGA
Megaceros aenigmaticus            ---------ATGAAAAACACATGGCTATTTC---CAATTGCTTATCGTGA
Phaeoceros laevis                 ---------ATGAAAAACACATGGCTATTTC---CAATTGCTTATCGTGA
                                                      ***   ***     * *   **  * ****

Beta macrocarpa                   TGCAGCGGAACCATGGCAATTAGGATTTCAAGACGCAGCAACACCTATGA
Beta vulgaris maritima            TGCAGCGGAACCATGGCAATTAGGATTTCAAGACGCAGCAACACCTATGA
Beta vulgaris vulgaris            TGCAGCGGAACCATGGCAATTAGGATTTCAAGACGCAGCAACACCTATGA
Silene latifolia                  TGCAGCGGAACCGTGGCAATTAGGATTTCAAGACGCAGCAACACCTATGA
Arabidopsis thaliana              TGCAGCGGAACCATGGCAATTAGGATUTCAAGACGCAGCTACACCTATAA
Brassica napus                    TGCAGCGGAACCATGGCAATTAGGATUTCAAGACGCAGCTACACCTATAA
PB235_cox2                        TGCAGCGGAACCATGGCAATTAGGATUTCAAGACGCAGCAAGCCCTATGA
RRIT251_cox2                      TGCAGCGGAACCATGGCAATTAGGATUTCAAGACGCAGCAAGCCCTATGA
RRIC110_cox2                      TGCAGCGGAACCATGGCAATTAGGATUTCAAGACGCAGCAAGCCCTATGA
RRII105_cox2                      TGCAGCGGAACCATGGCAATTAGGATUTCAAGACGCAGCAAGCCCTATGA
BPM24_cox2                        TGCAGCGGAACCATGGCAATTAGGATUTCAAGACGCAGCAAGCCCTATGA
RRIM600_cox2                      TGCAGCGGAACCATGGCAATTAGGATUTCAAGACGCAGCAAGCCCTATGA
Citrullus lanatus                 TGCAGCGGAACCATGGCAATTAGGATUTCAAGACGCAGCAACACCTATGA
Cucurbita pepo                    TGCAGCGGAACCATGGCAATTAGGATUTCAAGACGCAGCAACACCTATGA
Lotus japonicus                   TGCAGCGGAACCATGGCAATTAGGATTTCAAGACGCAGCAACACCTATGA
Millettia pinnata                 TGCAGCGGAACCATGGCAATTAGGATTTCAAGACGCAGCAACACCTATGA
Oryza sativa Japonica             TGCTGCGGAACCATGGCAATTAGGATUTCAAGACGCTGCAACACCTATGA
Zea mays mays                     TGCTGCGGAACCATGGCAATTAGGATUTCAAGACGCAGCAACACCTATGA
Megaceros aenigmaticus            TGCTGCCGAACCTTGGCAATTGGGATTTCAAGACGCAGCAACACCTATGA
Phaeoceros laevis                 TGCTGCCGAACCTTGCTAATTGGGATTCTAAGACGCAGCAACACTAATGA
                                  *** ** ***** **  **** ****   ******* ** *  *  ** *

Beta macrocarpa                   TGCAAGGAATAATCGACTTACATCATGATATCTTTTTCTTCCTUATTCTT
Beta vulgaris maritima            TGCAAGGAATAATCGACTTACATCATGATATCTTTTTCTTCCTUATTCTT
Beta vulgaris vulgaris            TGCAAGGAATAATCGACTTACATCATGATATCTTTTTCTTCCTUATTCTT
Silene latifolia                  TGCAAGGAATAATAGACTTACATCATGATATCTTTTTCTTCCTTATTCTT
```

```
Arabidopsis thaliana                TGCAAGGAATAATAGACTTACATCACGATATCTTTTTCTTCCTUATTCTG
Brassica napus                      TGCAAGGAATAATAGACTTACATCACGATATCTTTTTCTTCCTUATTCTG
PB235_cox2                          TGCAAGGAATAATGGACTTACATCACGATATCTTTTTCTTCCTCATTCTG
RRIT251_cox2                        TGCAAGGAATAATGGACTTACATCACGATATCTTTTTCTTCCTCATTCTG
RRIC110_cox2                        TGCAAGGAATAATGGACTTACATCACGATATCTTTTTCTTCCTCATTCTG
RRII105_cox2                        TGCAAGGAATAATGGACTTACATCACGATATCTTTTTCTTCCTCATTCTG
BPM24_cox2                          TGCAAGGAATAATGGACTTACATCACGATATCTTTTTCTTCCTCATTCTG
RRIM600_cox2                        TGCAAGGAATAATGGACTTACATCACGATATCTTTTTCTTCCTCATTCTG
Citrullus lanatus                   TGCAAGGAATAATAGACTTACATCACGATATCTTTTTCTTCCTCATTCTG
Cucurbita pepo                      TGCAAGGAATAATAGACTTACATCACGATATCTTTTTCTTCCTCATTCTT
Lotus japonicus                     TGCAAGGAATAATAGACTTACATCACGATATCTTTTTCTTCCTCATTCTT
Millettia pinnata                   TGCAAGGAATAATAGACTTACATCACGATATCTTTTTCTTCCTCATTCTG
Oryza sativa Japonica               TGCAAGGAATCATTGACTTACATCACGATATCTTTTTCTTCCTUATTCTG
Zea mays mays                       TGCAAGGAATCATTGACTTACATCACGATATCTTTTTCTTCCTCATTCTG
Megaceros aenigmaticus              TGCAAGGAATGATTGACTTACATCATGATATTTTTTTCTTTCTACCCATC
Phaeoceros laevis                   TGCAAGGAATAATTGACTTACATCATGATATTTTTTTCTTTCTACTCATT
                                    ********** ** *********** ***** ******** **    *

Beta macrocarpa                     ATTTTGGTTTTCGTATUATGGATCTTGGTTCGCGCTTTATGGCATTTCCA
Beta vulgaris maritima              ATTTTGGTTTTCGTATUATGGATCTTGGTTCGCGCTTTATGGCATTTCCA
Beta vulgaris vulgaris              ATTTTGGTTTTCGTATUATGGATCTTGGTTCGCGCTTTATGGCATTTCCA
Silene latifolia                    ATTTTGGTTTTCGTATUATGGATCTTGGTTCGCGCTTTATGGCATTTCCA
Arabidopsis thaliana                ATTTTGGTTTTCGTATTATGGATCTTGGTTCGCGCTTTATGGCATTTCCA
Brassica napus                      ATTTTGGTTTTCGTATTATGGATCTTGGTTCGCGCTTTATGGCATTTCCA
PB235_cox2                          ATTTTGGTTTUGTATTAUGGATCTTGGTTCGCGCTTTATGGCATTTCCA
RRIT251_cox2                        ATTTTGGTTTUGTATTAUGGATCTTGGTTCGCGCTTTATGGCATTTCCA
RRIC110_cox2                        ATTTTGGTTTUGTATTAUGGATCTTGGTTCGCGCTTTATGGCATTTCCA
RRII105_cox2                        ATTTTGGTTTUGTATTAUGGATCTTGGTTCGCGCTTTATGGCATTTCCA
BPM24_cox2                          ATTTTGGTTTUGTATTAUGGATCTTGGTTCGCGCTTTATGGCATTTCCA
RRIM600_cox2                        ATTTTGGTTTUGTATTAUGGATCTTGGTTCGCGCTTTATGGCATTTCCA
Citrullus lanatus                   ATTTTTGTTTTCGTATCAUGGATCTTGGTTCGCGCTTTATGGCATTTCCA
Cucurbita pepo                      CTTTTTGTTTTCGTATCAUGGATCTTGGTTCGCGCTTTATGGCATTTCCA
Lotus japonicus                     ATTTTGGTTTTCGTATUAUGGATCTTGGTTCGCGCTTTATGGCATTTCCA
Millettia pinnata                   ATTTTTGTTTTCGTATUAUGGATCTTGGTTCGCGCTTTATGGCATTTCCA
Oryza sativa Japonica               ATTTTGGTTTTCGTATUAUGGATGTTGGTTCGCGCTTTATGGCATTTCAA
Zea mays mays                       ATTTTGGTTTTCGTATUAUGGATGTTGGTTCGCGCTTTATGGCATTTCAA
Megaceros aenigmaticus              ATTATGATCTTTGTATTATGGATGTTGGTTCGCGCTTTATGGCATCTTCA
Phaeoceros laevis                   ATTCTGATCTTTGTATCATGGATGTTGGTTTGCGCTTTATGGCATCTTCA
                                     ** *  * ** **** * **** ****** ************** *  *

Beta macrocarpa                     CTATAAAAAAAATCCAATCCCGCAAAGGATTGTTCATGGAACTACTATCG
Beta vulgaris maritima              CTATAAAAAAAATCCAATCCCGCAAAGGATTGTTCATGGAACTACTATCG
Beta vulgaris vulgaris              CTATAAAAAAAATCCAATCCCGCAAAGGATTGTTCATGGAACTACTATCG
Silene latifolia                    CTATAAAAAAAATCCAATCCCGCAAAGGATTGTTCATGGAACTACTATCG
Arabidopsis thaliana                CTATAAAAAAAATGCAATCCCGCAAAGGATTGTTCATGGAACTACTATCG
Brassica napus                      CTATAAAGAAAATGCAATCCCGCAAAGGATTGTTCATGGAACTACTATCG
PB235_cox2                          CTATCAAAAAAATCCAATCCCGCAAAGGATTGTTCATGGAACTACTATCG
RRIT251_cox2                        CTATCAAAAAAATCCAATCCCGCAAAGGATTGTTCATGGAACTACTATCG
RRIC110_cox2                        CTATCAAAAAAATCCAATCCCGCAAAGGATTGTTCATGGAACTACTATCG
RRII105_cox2                        CTATCAAAAAAATCCAATCCCGCAAAGGATTGTTCATGGAACTACTATCG
BPM24_cox2                          CTATCAAAAAAATCCAATCCCGCAAAGGATTGTTCATGGAACTACTATCG
RRIM600_cox2                        CTATCAAAAAAATCCAATCCCGCAAAGGATTGTTCATGGAACTACTATCG
Citrullus lanatus                   CTATCAAAAAAATAAAATACCGCAAAGGATTGTTCATGGAACTACTATCG
Cucurbita pepo                      CTATAAACAAAATCAAATACCGCAAAGGATTGTTCATGGAACTACTATCG
Lotus japonicus                     CTATCAAAAAAATCCAATCCCGCAAAGGATTGTTCATGGAACTACTATCG
Millettia pinnata                   CTATAAAAAAAATCCAATCCCGCAAAGGATTGTTCATGGAACTACTATCG
Oryza sativa Japonica               CGAGCAAACTAATCCAATCCCGCAAAGGATTGTTCATGGAACTACTATCG
Zea mays mays                       CGAGCAAACTAATCCAATCCCGCAAAGGATTGTTCATGGAACTACTATCG
Megaceros aenigmaticus              CTATAAGAGAAATCCAATTCCAGAAAGGATTGTTCATGGAACTACTATAG
Phaeoceros laevis                   CTATAAGAGAAATCCAATTCCAGAAAGGATTGTTCATGGAACTACTATAG
                                    * *  *    ***  *** **  ************************* *
```

```
Beta macrocarpa              AGATTATTUGGACCATCTTTCCCAGTATCATCCTGATGTTCATTGCTATA
Beta vulgaris maritima       AGATTATTUGGACCATCTTTCCCAGTATCATCCTGATGTTCATTGCTATA
Beta vulgaris vulgaris       AGATTATTUGGACCATCTTTCCCAGTATCATCCTGATGTTCATTGCTATA
Silene latifolia             AGATTATTUGGACCATCTTTCCTAGTATCATCCTGATGTTCATTGCTATA
Arabidopsis thaliana         AGATTCTTUGGACCATUTTTCCTAGTATCATCTUGATGTTCATTGCTATA
Brassica napus               AGATTCTTUGGACCATCTTTCCTAGTCTCATCTUGATGTTCATTGCTATA
PB235_cox2                   AGATTCTTUGGACCATATTTCCTAGTATCATCCUTATGTTCATTGCTATA
RRIT251_cox2                 AGATTCTTUGGACCATATTTCCTAGTATCATCCUTATGTTCATTGCTATA
RRIC110_cox2                 AGATTCTTUGGACCATATTTCCTAGTATCATCCUTATGTTCATTGCTATA
RRII105_cox2                 AGATTCTTUGGACCATATTTCCTAGTATCATCCUTATGTTCATTGCTATA
BPM24_cox2                   AGATTCTTUGGACCATATTTCCTAGTATCATCCUTATGTTCATTGCTATA
RRIM600_cox2                 AGATTCTTUGGACCATATTTCCTAGTATCATCCUTATGTTCATTGCTATA
Citrullus lanatus            AGATTCTTTGGACCATTTTTCCTAGTATCATTCUGATGTTCATTGCTATA
Cucurbita pepo               AGATTCTTTGGACCATTTTTCCTAGTATCATTCUGATGTTCATTGCTATA
Lotus japonicus              AGATTATTUGGACCATATTTCCTAGTATCATCCUGATGTTCATTGCTATA
Millettia pinnata            AGATTCTTUGGACCATATTTCCTAGTATCATCCUAATGTTCATTGCTATA
Oryza sativa Japonica        AAATTATTUGGACCATATTTCCTAGTGTCATTCTTTTGTTCATTGCTATA
Zea mays mays                AAATTATTUGGACCATTTTTCCTAGTGTCATTCUATTGTTCATTGCTATA
Megaceros aenigmaticus       AGATTATTCGGACTATTTTTCTTAGTATTATCTCGATGTTTATTGCTATA
Phaeoceros laevis            AGATTATTTGGACTATTTTTCTTAGTATTATCTCGATGTTTATTGCTATA
                             * *** ** **** ** ****  *** * **     **** *********

Beta macrocarpa              CCATCATTTGCTCTGTTATACTCAATGGACGAGGTAGTAGTAGATCCAGC
Beta vulgaris maritima       CCATCATTTGCTCTGTTATACTCAATGGACGAGGTAGTAGTAGATCCAGC
Beta vulgaris vulgaris       CCATCATTTGCTCTGTTATACTCAATGGACGAGGTAGTAGTAGATCCAGC
Silene latifolia             CCATCATTTGCTCTCTTATACTCAATGGACGAGGTAGTAGTAGATCCAGC
Arabidopsis thaliana         CCATCATTTGCTCTCTTATACTCAATGGACGAGGTAGTAGTAGATCCAGC
Brassica napus               CCATCATTTGCTCTCTTATACUCAATGGACGAGGTAGTAGTAGATCCAGC
PB235_cox2                   CCATCATTTGCTCTGTTATACTCAATGGACGAGGTAGTAGTAGATCCAGC
RRIT251_cox2                 CCATCATTTGCTCTGTTATACTCAATGGACGAGGTAGTAGTAGATCCAGC
RRIC110_cox2                 CCATCATTTGCTCTGTTATACTCAATGGACGAGGTAGTAGTAGATCCAGC
RRII105_cox2                 CCATCATTTGCTCTGTTATACTCAATGGACGAGGTAGTAGTAGATCCAGC
BPM24_cox2                   CCATCATTTGCTCTGTTATACTCAATGGACGAGGTAGTAGTAGATCCAGC
RRIM600_cox2                 CCATCATTTGCTCTGTTATACTCAATGGACGAGGTAGTAGTAGATCCAGC
Citrullus lanatus            CCATCCTTTGCTCTCTTATACTCAATGGACGAGGTAGTAGTAGATCCAGC
Cucurbita pepo               CCATCCTTTGCTCTCTTATACTCAATGGACGAGGTAGTAGTAGATCCAGC
Lotus japonicus              CCATCATTTGCTCTGTTATACTCAATGGACGAGGTAGTAGTAGATCCAGC
Millettia pinnata            CCATCATTTGCTCTGTTATACTCAATGGACGAGGTAGTAGTAGATCCAGC
Oryza sativa Japonica        CCATCGTTTGCTCTGTTATACTCAATGGACGGGGTATTAGTAGATCCAGC
Zea mays mays                CCATCGTTTGCTCTGTTATACTCAATGGACGGGGTATTAGTAGATCCAGC
Megaceros aenigmaticus       CCATCTTTTGCTTTATTATATTCAATGGACGAGGTA---GTAGATCCAGC
Phaeoceros laevis            CCATCTTTTGCTTTATTATATTCAATGGACGAAGTT---GTAGATCCAGC
                             ***** ****** * *****  *********  **    ***********

Beta macrocarpa              CATTACTATCAAAGCTATTGGACATCAATGGTATUGGACTTATGAGTATT
Beta vulgaris maritima       CATTACTATCAAAGCTATTGGACATCAATGGTATUGGACTTATGAGTATT
Beta vulgaris vulgaris       CATTACTATCAAAGCTATTGGACATCAATGGTATUGGACTTATGAGTATT
Silene latifolia             CATTACTATCAAAGCTATTGGACATCAATGGTATTGGACTTATGAGTATT
Arabidopsis thaliana         CATTACTATCAAAGCTATTGGACATCAATGGTATUGGACTTATGAGTATT
Brassica napus               CATTACTATCAAAGCTATTGGACATCAATGGTATTGGACTTATGAGTATT
PB235_cox2                   CATTACTATCAAAGCTATTGGACATCAATGGTATUGGACTTATGAGTATT
RRIT251_cox2                 CATTACTATCAAAGCTATTGGACATCAATGGTATUGGACTTATGAGTATT
RRIC110_cox2                 CATTACTATCAAAGCTATTGGACATCAATGGTATUGGACTTATGAGTATT
RRII105_cox2                 CATTACTATCAAAGCTATTGGACATCAATGGTATUGGACTTATGAGTATT
BPM24_cox2                   CATTACTATCAAAGCTATTGGACATCAATGGTATUGGACTTATGAGTATT
RRIM600_cox2                 CATTACTATCAAAGCTATTGGACATCAATGGTATUGGACTTATGAGTATT
Citrullus lanatus            CATGACTATCAAAGCTATTGGACATCAATGGTATUGGACTTATGAGTATT
Cucurbita pepo               CATGACTATCAAAGCTATTGGACATCAATGGTATUGGACTTATGAGTATT
Lotus japonicus              CATTACTATCAAAGCTATTGGACATCAATGGTATUGGACTTATGAGTATT
Millettia pinnata            CATTACTATCAAAGCTATTGGACATCAATGGTATUGGACTTATGAGTATT
Oryza sativa Japonica        CATTACTATCAAAGCTATTGGACATCAAT---------CTTATGAGTATT
Zea mays mays                CATTACTATCAAAGCTATTGGACATCAATGGTATUGGACTTATGAGTATT
```

```
Megaceros aenigmaticus            TGTTACTATCAAAGCTATTGGACATCAATGGTATTGGACTTATGAGTATT
Phaeoceros laevis                 TGTTACTATCAAAGCTATTGGACATCAATGGTATTGGATATCCTATTATT
                                    * *************************           *   * ****

Beta macrocarpa                   CGGACTATAACAGTTCCGATGAACAGTCACTCACTTTTGACAGTTATAUG
Beta vulgaris maritima            CGGACTATAACAGTTCCGATGAACAGTCACTCACTTTTGACAGTTATAUG
Beta vulgaris vulgaris            CGGACTATAACAGTTCCGATGAACAGTCACTCACTTTTGACAGTTATAUG
Silene latifolia                  CGGACTATAACAGTTCCGATGAACAGTCACTCACTTTTGACAGTTATATG
Arabidopsis thaliana              CTGACTATAACAGTTCCGATGAGCAGTCACTCACTTTTGACAGTTATATG
Brassica napus                    CTGACTATAACAGTTCCGATGAGCAGTCACTCACTTTTGACAGTTATATG
PB235_cox2                        CAGACTATAACAGTTCCGATGAAGAGTCACTCACTTTTGACAGTTATAUG
RRIT251_cox2                      CAGACTATAACAGTTCCGATGAAGAGTCACTCACTTTTGACAGTTATAUG
RRIC110_cox2                      CAGACTATAACAGTTCCGATGAAGAGTCACTCACTTTTGACAGTTATAUG
RRII105_cox2                      CAGACTATAACAGTTCCGATGAAGAGTCACTCACTTTTGACAGTTATAUG
BPM24_cox2                        CAGACTATAACAGTTCCGATGAAGAGTCACTCACTTTTGACAGTTATAUG
RRIM600_cox2                      CAGACTATAACAGTTCCGATGAAGAGTCACTCACTTTTGACAGTTATAUG
Citrullus lanatus                 CTGACTATAACAGTTCCGATGAACAGTCACTCACTTTTGACAGTTATAUG
Cucurbita pepo                    CTGACTATAACAGTTCCGATGAAGAGTCACTCACTTTTGACAGTTATAUG
Lotus japonicus                   CAGACTATAACAGTTCCGATGAACAGTCACTCACTTTTGACAGTTATAUG
Millettia pinnata                 CAGACTATAACAGTTCCGATGAACAGTCACTCACTTTTGACAGTTATAUG
Oryza sativa Japonica             CGGACTATAACAGTTCCGATGAACAGTCACTCACTTTTGACAGTTATAUG
Zea mays mays                     CGGACTATAACAGTTCCGATGAACAGTCACTCACTTTTGACAGTTATAUG
Megaceros aenigmaticus            CAGACTATAACAGTTCCGATGAACAGTCACTAACTTTTGACAGTTATATG
Phaeoceros laevis                 CCGACTATAACAGTTCCGATGAACAGTCACTAACTTTTGACAGTTATATG
                                  * ********************  *******  **************** *

Beta macrocarpa                   ATTCCAGAAGATGATCUAGAATTGGGTCAATUACGTTTATTAGAAGTGGA
Beta vulgaris maritima            ATTCCAGAAGATGATCUAGAATTGGGTCAATUACGTTTATTAGAAGTGGA
Beta vulgaris vulgaris            ATTCCAGAAGATGATCUAGAATTGGGTCAATUACGTTTATTAGAAGTGGA
Silene latifolia                  ATTCCAGAAGATGATTTAGAATTGGGTCAATTACGTTTATTAGAAGTGGA
Arabidopsis thaliana              ATTCCAGAAGAAGATCTAGAATTGGGTCAATUACGTTTATTAGAAGTGGA
Brassica napus                    ATTCCAGAAGAAGATCTAGAATTGGGTCAATUACGTTTATTAGAAGTGGA
PB235_cox2                        ATTCCAGAAGATGATCTAGAATTGGGTCAATUACGTTTATTAGAAGTGGA
RRIT251_cox2                      ATTCCAGAAGATGATCTAGAATTGGGTCAATUACGTTTATTAGAAGTGGA
RRIC110_cox2                      ATTCCAGAAGATGATCTAGAATTGGGTCAATUACGTTTATTAGAAGTGGA
RRII105_cox2                      ATTCCAGAAGATGATCTAGAATTGGGTCAATUACGTTTATTAGAAGTGGA
BPM24_cox2                        ATTCCAGAAGATGATCTAGAATTGGGTCAATUACGTTTATTAGAAGTGGA
RRIM600_cox2                      ATTCCAGAAGATGATCTAGAATTGGGTCAATUACGTTTATTAGAAGTGGA
Citrullus lanatus                 ATTCCAGAAGATGATCTAGAATTGGGTCAATUACGTTTATTAGAAGTGGA
Cucurbita pepo                    ATTCCAGAAGATGATCTAGAATTGGGTCAATUACGTTTATTAGAAGTGGA
Lotus japonicus                   ATTCCAGAAGATGATCTAGAATTGGGTCAATUACGTTTATTAGAAGTTGA
Millettia pinnata                 ATTCCAGAAGATGATCTAGAATTGGGTCAATUACGTTTATTAGAAGTGGA
Oryza sativa Japonica             ATTCCAGAAGATGATUUAGAATTGGGTCAATUACGTTTATTAGAAGTTGA
Zea mays mays                     ATTCCAGAAGATGATUUAGAATTGGGTCAATUACGTTTATTAGAAGTTGA
Megaceros aenigmaticus            ATTACGGAGGATGAGTTAGAATTAGGTGCATTACGTTTATTAGAAGTAGA
Phaeoceros laevis                 ATTACGGAGGATGAGTCAGAATTAGGTTCATTATGTTTATTAGAAGTAGA
                                  *** * ** ** **   ****** ***  ** * ************* **

Beta macrocarpa                   CAATAGAGTGGTTGTACCAGCCAAAACTCATATACGTATTATTGTAACAT
Beta vulgaris maritima            CAATAGAGTGGTTGTACCAGCCAAAACTCATATACGTATTATTGTAACAT
Beta vulgaris vulgaris            CAATAGAGTGGTTGTACCAGCCAAAACTCATATACGTATTATTGTAACAT
Silene latifolia                  CAATAGAGTGGTTGTACCAGCCAAAACTCATATACGTATTATTGTAACAT
Arabidopsis thaliana              CAATAGAGTGGTTGTACCAGCCAAAACTCATCTACGTATTATTGTAACAT
Brassica napus                    CAATAGAGTGGTTGTACCAGCCAAAACTCATCTACGTATTATTGTAACAT
PB235_cox2                        CAATAGAGTGGTTGTACCAGCCAAAACTCATCTACGTATTATTGTAACAT
RRIT251_cox2                      CAATAGAGTGGTTGTACCAGCCAAAACTCATCTACGTATTATTGTAACAT
RRIC110_cox2                      CAATAGAGTGGTTGTACCAGCCAAAACTCATCTACGTATTATTGTAACAT
RRII105_cox2                      CAATAGAGTGGTTGTACCAGCCAAAACTCATCTACGTATTATTGTAACAT
BPM24_cox2                        CAATAGAGTGGTTGTACCAGCCAAAACTCATCTACGTATTATTGTAACAT
RRIM600_cox2                      CAATAGAGTGGTTGTACCAGCCAAAACTCATCTACGTATTATTGTAACAT
Citrullus lanatus                 CAATAGAGTGGTTCTACCAGCCAAAACTCATCTACGTATTATTGTAACAU
Cucurbita pepo                    CAATAGAGTGGTTCTACCAGCCAAAACTCATCTACGTATTATTGTAACAU
```

```
Lotus japonicus              CAATAGAGTGGTTGTACCAGCCAAAACTCATCTACGTATTATTGTAACAU
Millettia pinnata            CAATAGAGTGGTTGTACCAGCCAAAACTCATCTACGTATTATTGTAACAU
Oryza sativa Japonica        CAATAGAGTGGTTGTACCAGCCAAAACTCATCTACGTATGATTGTAACAU
Zea mays mays                CAATAGAGTGGTTGTACCAGCCAAAACTCATCTACGTATGATTGTAACAU
Megaceros aenigmaticus       CAATAGAGTGATTGTACCAGCGAAAACTCATATCCGTCTGATTATCACAT
Phaeoceros laevis            TAATAGAGTGATTGTATCAGCGAAAACTCATATCCGTCTGATTATCACAT
                              ********* ** ** **** ********* * *** * *** * ***

Beta macrocarpa              CCGCTGATGTACTTCATAGTTGGGCTGTACCTTCCTUAGGTGTCAAATGT
Beta vulgaris maritima       CCGCTGATGTACTTCATAGTTGGGCTGTACCTTCCTUAGGTGTCAAATGT
Beta vulgaris vulgaris       CCGCTGATGTACTTCATAGTTGGGCTGTACCTTCCTUAGGTGTCAAATGT
Silene latifolia             CTGCTGATGTACTTCATAGTTGGGCTGTACCTTCCTTAGGTGTCAAATGT
Arabidopsis thaliana         CTGCTGATGTACUTCATAGTTGGGCTGTACCTTCCTUAGGTGTCAAATGT
Brassica napus               CTGCTGATGTACUTCATAGTTGGGCTGTACCTTCCTUAGGTGTCAAATGT
PB235_cox2                   CTGCTGATGTACUTCATAGTTGGGCTGTACCTTCCTUAGGTGTCAAATGT
RRIT251_cox2                 CTGCTGATGTACUTCATAGTTGGGCTGTACCTTCCTUAGGTGTCAAATGT
RRIC110_cox2                 CTGCTGATGTACUTCATAGTTGGGCTGTACCTTCCTUAGGTGTCAAATGT
RRII105_cox2                 CTGCTGATGTACUTCATAGTTGGGCTGTACCTTCCTUAGGTGTCAAATGT
BPM24_cox2                   CTGCTGATGTACUTCATAGTTGGGCTGTACCTTCCTUAGGTGTCAAATGT
RRIM600_cox2                 CTGCTGATGTACUTCATAGTTGGGCTGTACCTTCCTNAGGTGTCAAATGT
Citrullus lanatus            CTGCTGATGTACUTCATAGTTGGGCTGTACCTTCCTUAGGTGTCAAATGT
Cucurbita pepo               CTGCTGATGTACUTCATAGTTGGGCTGTACCTTCCTUAGGTGTCAAATGT
Lotus japonicus              CTGCTGATGTACUTCATAGTTGGGCTGTACCTTCCTTAGGTGTCAAATGT
Millettia pinnata            CTGCTGATGTACUTCATAGTTGGGCTGTACCTTCCTTAGGTGTCAAATGT
Oryza sativa Japonica        CCGCTGATGTACUTCATAGTTGGGCTGTACCTTCCTUAGGTGTCAAATGT
Zea mays mays                CCGCTGATGTACUTCATAGTTGGGCTGTACCTTCCTUAGGTGTCAAATGT
Megaceros aenigmaticus       CTGCTGATGTACCTTATAGTTGGGCTGTACCTTCCTCAGGTGTAAAATGC
Phaeoceros laevis            TTGCTGATGTACCTTATAGTTGGGCTGTACCTTCCTTAGGTGTAAAATGC
                               ********** * ********************* ****** *****

Beta macrocarpa              GATGCTGTACCTGGTCGTTTAAATCAGACCTCTATTTTGGTACAACGAGA
Beta vulgaris maritima       GATGCTGTACCTGGTCGTTTAAATCAGACCTCTATTTTGGTACAACGAGA
Beta vulgaris vulgaris       GATGCTGTACCTGGTCGTTTAAATCAGACCTCTATTTTGGTACAACGAGA
Silene latifolia             GATGCTGTACCTGGTCGTTTAAATCAGACCTCTATTTTGGTACAACGAGA
Arabidopsis thaliana         GATGCTGTACCTGGTCGTTTAAATCAAATCTCTATTTTGGTACAACGAGA
Brassica napus               GATGCTGTACCTGGTCGTTTAAATCAAATCTCTATTTTGGTACAACGAGA
PB235_cox2                   GATGCTGTACCTGGTCGTTTAAATCAGATCTCTATTTUGGTACAACGAGA
RRIT251_cox2                 GATGCTGTACCTGGTCGTTTAAATCAGATCTCTATTTUGGTACAACGAGA
RRIC110_cox2                 GATGCTGTACCTGGTCGTTTAAATCAGATCTCTATTTUGGTACAACGAGA
RRII105_cox2                 GATGCTGTACCTGGTCGTTTAAATCAGATCTCTATTTUGGTACAACGAGA
BPM24_cox2                   GATGCTGTACCTGGTCGTTTAAATCAGATCTCTATTTUGGTACAACGAGA
RRIM600_cox2                 GATGCTGTACCTGGTCGTTTAAATCAGATCTCTATTTCGGTACAACGAGA
Citrullus lanatus            GATGCTGTACCTGGTCGTTTAAATCAGATCTCCATTTTGGTACAACGAGA
Cucurbita pepo               GATGCTGTACCTGGTCGTTTAAATCAGATCTCCATTTTGGTACAACGAGA
Lotus japonicus              GATGCTGTACCTGGTCGTTTAAATCAGATATCTATTTUGGTACAACGAGA
Millettia pinnata            GATGCTGTACCTGGTCGTTTAAATCAGATCTCTATTTUGGTACAACGAGA
Oryza sativa Japonica        GATGCTGTACCTGGTCGTTUAAATCTTACCTCCATCTUGGTACAACGAGA
Zea mays mays                GATGCTGTACCTGGTCGTTUAAATCTTACCTCCATCTUGGTACAACGAGA
Megaceros aenigmaticus       GATGCTGTACCCGGTCGTTCAAATCAGACTTCCATTTTTATTAAACGAAA
Phaeoceros laevis            GATGCTGTACTCGGTTGTTTAAATCAGACTTCCATTTTTATTAAACGAAA
                             **********  *** *** *****  *  ** ** *   *  ***** *

Beta macrocarpa              AGGAGTTTACTATGGTCAGTGCAGTGAAATTTGTGGAACTAATCATGCTT
Beta vulgaris maritima       AGGAGTTTACTATGGTCAGTGCAGTGAAATTTGTGGAACTAATCATGCTT
Beta vulgaris vulgaris       AGGAGTTTACTATGGTCAGTGCAGTGAAATTTGTGGAACTAATCATGCTT
Silene latifolia             AGGAGTTTACTATGGTCAGTGCAGTGAAATTTGTGGAACTAATCATGCTT
Arabidopsis thaliana         AGGAGTTTACTATGGTCAGTGCAGTGAGATTTGTGGAACTAATCATGCCT
Brassica napus               AGGAGTTTACTATGGTCAGTGCAGTGAGATTTGTGGAACTAATCATGCCT
PB235_cox2                   AGGAGTTTACTATGGTCAGTGCAGTGAGATTTGTGGAACTAATCATGCCT
RRIT251_cox2                 AGGAGTTTACTATGGTCAGTGCAGTGAGATTTGTGGAACTAATCATGCCT
RRIC110_cox2                 AGGAGTTTACTATGGTCAGTGCAGTGAGATTTGTGGAACTAATCATGCCT
RRII105_cox2                 AGGAGTTTACTATGGTCAGTGCAGTGAGATTTGTGGAACTAATCATGCCT
```

```
BPM24_cox2                        AGGAGTTTACTATGGTCAGTGCAGTGAGATTTGTGGAACTAATCATGCCT
RRIM600_cox2                      AGGAGTTTACTATGGTCAGTGCAGTGAGATTTGTGGAACTAATCATGCCT
Citrullus lanatus                 AGGAGTTTACTATGGTCAGTGCAGTGAGATTTGTGGAACTAATCATGCCT
Cucurbita pepo                    AGGAGTTTACTATGGTCAGTGCAGTGAGATTTGTGGAACTAATCATGCCT
Lotus japonicus                   AGGGGTTTACTATGGTCAGTGCAGTGAGATTTGTGGAACTAATCATGCCT
Millettia pinnata                 AGGGGTTTACTATGGTCAGTGCAGTGAGATTTGTGGAACTAATCATGCCT
Oryza sativa Japonica             AGGAGTTTACTATGGTCAGTGCAGTGAGATTTGTGGAACTAATCATGCCT
Zea mays mays                     AGGAGTTTACTATGGTCAGTGCAGTGAGATTTGTGGAACTAATCATGCCT
Megaceros aenigmaticus            AGGAGTTTACTATGGTCAATGCAGTGAAATTTGTGGAACCAATCATGCGT
Phaeoceros laevis                 AGGAGTTTACTATGGTCAACGCAGTGAAATTCGTGGAACCAATTATGCGT
                                  *** **************  ******* *** ******* *** **** *

Beta macrocarpa                   TTATGCCTATCGTCGTAGAAGCTGTTTCTAGGAAAGATTATGGTTCTUGG
Beta vulgaris maritima            TTATGCCTATCGTCGTAGAAGCTGTTTCTAGGAAAGATTATGGTTCTUGG
Beta vulgaris vulgaris            TTATGCCTATCGTCGTAGAAGCTGTTTCTAGGAAAGATTATGGTTCTUGG
Silene latifolia                  TTATGCCTATCGTCGTAGAAGCTGTTTCTAGGAAAGATTATGGTTCTUGG
Arabidopsis thaliana              TTAUGTCTATCGTCGTAGAAGCTGTTUCTAGGAAAGATTATGGTTCTUGG
Brassica napus                    TTAUGCCTATCGTCGTAGAAGCTGTTUCTAGGAAAGATTATGGTTCTUGG
PB235_cox2                        TTAUGCCTATCGTCGTAGAAGCTGTTUCTAGGAAAGATTATGGTTCTUGG
RRIT251_cox2                      TTAUGCCTATCGTCGTAGAAGCTGTTUCTAGGAAAGATTATGGTTCTUGG
RRIC110_cox2                      TTAUGCCTATCGTCGTAGAAGCTGTTUCTAGGAAAGATTATGGTTCTUGG
RRII105_cox2                      TTAUGCCTATCGTCGTAGAAGCTGTTUCTAGGAAAGATTATGGTTCTUGG
BPM24_cox2                        TTAUGCCTATCGTCGTAGAAGCTGTTUCTAGGAAAGATTATGGTTCTUGG
RRIM600_cox2                      TTAUGCCTATCGTCGTAGAAGCTGTTNCTAGGAAAGATTATGGTTCTNGG
Citrullus lanatus                 TTAUGCCTATCGTCGTAGAAGCAGTTCCTAGGAAAGATTATGGTTCTCGG
Cucurbita pepo                    TTAUGCCTATCGTCGTAGAAGCAGTTUCTAGGAAAGATTATGGTTCTCGG
Lotus japonicus                   TTAUGCCTATCGTCGTAGAAGCTGTTUCTAGTAAAGATTATGGTTCTUGG
Millettia pinnata                 TTAUGCCTATCGTCGTAGAAGCTGTTUCTAGTAAAGATTATGGTTCTUGG
Oryza sativa Japonica             TTAUGCCTATCGTCGTAGAAGCGGTGACTTTGAAAGATTATGCGGATTGG
Zea mays mays                     TTAUGCCTATCGTCGTAGAAGCAGTGACTTTGAAAGATTATGCGGATTGG
Megaceros aenigmaticus            TTATGCCTATTGTCGTAGAAGCTGTTTCTTTGGATGATCATGTTTCTTGG
Phaeoceros laevis                 TTATGCCTATTGTCGTAGAAGCTGTTTCTTTGGATGATTATGTTTCTCGG
                                  *** * **** *********** **  **    * *** ***    * **

Beta macrocarpa                   GTATCTAATCAATTAATCCCCCAAACC---GGAGAAGCTTAA
Beta vulgaris maritima            GTATCTAATCAATTAATCCCCCAAACC---GGAGAAGCTTAA
Beta vulgaris vulgaris            GTATCTAATCAATTAATCCCCCAAACC---GGAGAAGCTTAA
Silene latifolia                  GTATCTACTCAATTAATCCCCCAAACC---GGAGAAGCTTAA
Arabidopsis thaliana              GTATCCAATCAATTAATCCCCCAAACC---GGGGAAGCTTAA
Brassica napus                    GTATCCAATCAATTAATCCCCCAAACC---GGGGAAGCTTAA
PB235_cox2                        GTATCCAATCAATTAATTCCCCAAACC---GGGGAAGCTTAA
RRIT251_cox2                      GTATCCAATCAATTAATTCCCCAAACC---GGGGAAGCTTAA
RRIC110_cox2                      GTATCCAATCAATTAATTCCCCAAACC---GGGGAAGCTTAA
RRII105_cox2                      GTATCCAATCAATTAATTCCCCAAACC---GGGGAAGCTTAA
BPM24_cox2                        GTATCCAATCAATTAATTCCCCAAACC---GGGGAAGCTTAA
RRIM600_cox2                      GTATCCAATCAATTAATTCCCCAAACC---GGGGAAGCTTAA
Citrullus lanatus                 GTATCCAATCAATTAATCCCCCAAACC---GGGGAAGCTTAA
Cucurbita pepo                    GTATCCAATCAATTAATCCCCAAAACC---GGGGAAGCTTAA
Lotus japonicus                   GTATCCAATCAATTAATCCCACA--------GGGAAGCTGA-
Millettia pinnata                 GTATCCAATCAATTAATCCCACAAACAACAGGGGAAGCTTAA
Oryza sativa Japonica             GTATCCAATCAATTAATCCTCCAAACC--------AACTAA-
Zea mays mays                     GTATCCAATCAATTAATCCTCCAAACC--------AACTAA-
Megaceros aenigmaticus            GTATCTAATGAATTAGACTAA---------------------
Phaeoceros laevis                 GTATCTAATGAATTAGACTGA---------------------
                                  ***** * * *****
```

###################

cox3 multiple sequence alignment

```
PB235_cox3                        ---------------------------------------------ATGAT
RRIT251_cox3                      ---------------------------------------------ATGAT
RRII105_cox3                      ---------------------------------------------ATGAT
BPM24_cox3                        ---------------------------------------------ATGAT
```

```
RRIC110_cox3                      ---------------------------------------------ATGAT
RRIM600_cox3                      ---------------------------------------------ATGAT
Citrullus lanatus                 ---------------------------------------------ATGAT
Cucurbita pepo                    ---------------------------------------------ATGAT
Lotus japonicus                   ---------------------------------------------ATGAT
Millettia pinnata                 ---------------------------------------------ATGAT
Oryza sativa Japonica             ATGGGGGTGAAGGGGGGTTTACATACAACCGGGGCAAAGTGGTTTATGAT
Arabidopsis thaliana              ---------------------------------------------ATGAT
Brassica napus                    ---------------------------------------------ATGAT
Beta macrocarpa                   ---------------------------------------------ATGAT
Beta vulgaris maritima            ---------------------------------------------ATGAT
Beta vulgaris vulgaris            ---------------------------------------------ATGAT
Silene latifolia                  ---------------------------------------------ATGAT
Anomodon rugelii                  ---------------------------------------------ATGAG
Anomodon attenuatus               ---------------------------------------------ATGAG
Huperzia squarrosa                ---------------------------------------------ATGAG
Megaceros aenigmaticus            ---------------------------------------------ATGAG
Phaeoceros laevis                 ---------------------------------------------ATGAG
                                                                               ****

PB235_cox3                        TGAATCTCAGAGGCATTCTTATCATTTGGTAGATCCAAGTCCATGGCCTA
RRIT251_cox3                      TGAATCTCAGAGGCATTCTTATCATTTGGTAGATCCAAGTCCATGGCCTA
RRII105_cox3                      TGAATCTCAGAGGCATTCTTATCATTTGGTAGATCCAAGTCCATGGCCTA
BPM24_cox3                        TGAATCTCAGAGGCATTCTTATCATTTGGTAGATCCAAGTCCATGGCCTA
RRIC110_cox3                      TGAATCTCAGAGGCATTCTTATCATTTGGTAGATCCAAGTCCATGGCCTA
RRIM600_cox3                      TGAATCTCAGAGGCATTCTTATCATTTGGTAGATCCAAGTCCATGGCCTA
Citrullus lanatus                 TGAATCTAAGAGGCATTCTTATCATTTGGTAGATCCAAGTCCATGGCCCA
Cucurbita pepo                    TGAATCTCAGAGGCATTCTTTTCATTTGGTAGATCCAAGCCCATGGCCCA
Lotus japonicus                   TGAATCTCAGAGGCATTCTTATCATTTGGTAGATCCAAGTCCATGGCCTA
Millettia pinnata                 TGAATCTCAGAGGCATTCTTATCATTTGGTAGATCCAAGTCCATGGCCTA
Oryza sativa Japonica             TGAATCTCAGAGGCATTCTTATCATTTGGTAGATCCAAGTCCATGGCCTA
Arabidopsis thaliana              TGAATCTCAGAGGCATTCTTATCATTTGGTAGATCCAAGTCCATGGCCTA
Brassica napus                    TGAATCTCAGAGGCATTCTTATCATTTGGTAGATCCAAGTCCATGGCCTA
Beta macrocarpa                   TGAATCTCAAAGGCATTCTTTTCATTTGGTAGATCCAAGTCCATGGCCTA
Beta vulgaris maritima            TGAATCTCAAAGGCATTCTTTTCATTTGGTAGATCCAAGTCCATGGCCTA
Beta vulgaris vulgaris            TGAATCTCAAAGGCATTCTTTTCATTTGGTAGATCCAAGTCCATGGCCTA
Silene latifolia                  TGAATCTCAACGGCATTCTTTTCATTTGGTAGATCCAAGTCCATGGCCTA
Anomodon rugelii                  TGTCTCTCAAAAGCATCCTTATCATTTAGTAGATCCAAGTCCATGGCCTC
Anomodon attenuatus               TGTCTCTCAAAAGCATCCTTATCATTTAGTAGATCCAAGTCCATGGCCTC
Huperzia squarrosa                TGTCTCTCAAAAGCATCCTTATCATTTAGTAGATCCAAGTCCATGGCCTC
Megaceros aenigmaticus            TGTTTCTCAAAAGCATCCTTACCATTTAGTAGATCCAAGTCCATGGCTAA
Phaeoceros laevis                 TGTCTCTCAAAAGCATCTTTATCATTTAGTAGATCCAAGTCCACGGCTAA
                                  **  *** *   ****  **  ***** *********** *** ***

PB235_cox3                        TTTCGGGTTCACTCGGAACTTTGGCAACCACCGTAGGAGGTGTGATGTAC
RRIT251_cox3                      TTTCGGGTTCACTCGGAACTTTGGCAACCACCGTAGGAGGTGTGATGTAC
RRII105_cox3                      TTTCGGGTTCACTCGGAACTTTGGCAACCACCGTAGGAGGTGTGATGTAC
BPM24_cox3                        TTTCGGGTTCACTCGGAACTTTGGCAACCACCGTAGGAGGTGTGATGTAC
RRIC110_cox3                      TTTCGGGTTCACTCGGAACTTTGGCAACCACCGTAGGAGGTGTGATGTAC
RRIM600_cox3                      TTTCGGGTTCACTCGGAACTTTGGCAACCACCGTAGGAGGTGTGATGTAC
Citrullus lanatus                 TTTCGGGTTCACTCGGAGCTTTGGCAACCACCGTAGGAGGTGTGATGTAC
Cucurbita pepo                    TTTCGGGTTCACTCGGAGCTTTGGCAACCACCGTAGGAGGTGTGATGTAC
Lotus japonicus                   TTTCGGGTTCACTCGGAGCTTTGGCAACCACCGTAGGAGGTGTGATGTAC
Millettia pinnata                 TTTCGGGTTCACTCGGAGCTTTGGCAACCACCGTAGGAGGTGTGATGTAC
Oryza sativa Japonica             TTTCGGGTTCACTCGGAGCTTTGGCAACCACCGTAGGAGGTGTGATGTAC
Arabidopsis thaliana              TTTCGGGTTCACTCGGAGCTTTGGCAACCACCGTAGGAGGTGTGATGTAC
Brassica napus                    TTTCGGGTTCACTCGGAGCTTTGGCAACCACCGTAGGAGGTGTGATGTAC
Beta macrocarpa                   TTTCGGGTTCACTCGGAGCTTTGGCAACGACCGTAGGAGGTGTGATGTAC
Beta vulgaris maritima            TTTCGGGTTCACTCGGAGCTTTGGCAACGACCGTAGGAGGTGTGATGTAC
Beta vulgaris vulgaris            TTTCGGGTTCACTCGGAGCTTTGGCAACGACCGTAGGAGGTGTGATGTAC
Silene latifolia                  TTTCGGGTTCACTCGGAGCTTTGGCAACCACCGTAGGAGGTGTGATGTAC
Anomodon rugelii                  TTTTGGGTTCATTGGGAGCTTTGGCAAGCACCATTGGTGGTGTTATGTAT
```

```
Anomodon attenuatus                TTTTGGGTTCATTGGGAGCTTTGGCAAGCACCATTGGTGGTGTTATGTAT
Huperzia squarrosa                 TTTTGGGTTCATTGGGAGCTTTGGCAAGCACCATTGGTGGTGTTATGTAT
Megaceros aenigmaticus             TTTTGGGTTCATTGGGAGCTTTGGCAAGCACCGTTGGTGGTGTTATGTAC
Phaeoceros laevis                  TTTTGGTTTTATTGGGAGCTTTGGCAAGCACCGTTGGTGGTGTTATGTAC
                                   *** ** ** * * *** *********  *** * ** ***** *****

PB235_cox3                         ATGCACTCATTTCAAGGGGGTGCAACACTTCTCAGTTTGGGCCTCATATT
RRIT251_cox3                       ATGCACTCATTTCAAGGGGGTGCAACACTTCTCAGTTTGGGCCTCATATT
RRII105_cox3                       ATGCACTCATTTCAAGGGGGTGCAACACTTCTCAGTTTGGGCCTCATATT
BPM24_cox3                         ATGCACTCATTTCAAGGGGGTGCAACACTTCTCAGTTTGGGCCTCATATT
RRIC110_cox3                       ATGCACTCATTTCAAGGGGGTGCAACACTTCTCAGTTTGGGCCTCATATT
RRIM600_cox3                       ATGCACTCATTTCAAGGGGGTGCAACACTTCTCAGTTTGGGCCTCATATT
Citrullus lanatus                  ATGCACTCATTTCAAGGGGGTGCAACACTTUTAAGTTTGGGCCTCATATT
Cucurbita pepo                     ATGCACTCATTTCAAGGGGGTGCAACACTGATCAGTTTGGGCCTCATATT
Lotus japonicus                    ATGCACTCATTTCAAGGGGGTGCAACACTTCTCAGTTTGGGCCTAATATT
Millettia pinnata                  ATGCACTCATTTCAAGGGGGTGCAACACTTCTCAGTTTGGGCCTAATATT
Oryza sativa Japonica              ATGCACTCATTTCAAGGGGGTGCAACACTTCTCAGTTTGGGCCTAATATT
Arabidopsis thaliana               ATGCACUCATTTCAAGGGGGTGCAAGACTTCTAAGTTTGGGCCTCATATT
Brassica napus                     ATGCACUCATTTCAAGGGGGTGCAAGACTTCTAAGTTTGGGCCTCATATT
Beta macrocarpa                    ATGCACTCATTTCAAGGGGGTGCAACACTTUTAAGTTTGGGCCTTATTTT
Beta vulgaris maritima             ATGCACTCATTTCAAGGGGGTGCAACACTTUTAAGTTTGGGCCTTATTTT
Beta vulgaris vulgaris             ATGCACTCATTTCAAGGGGGTGCAACACTTUTAAGTTTGGGCCTTATTTT
Silene latifolia                   ATGCACTCATTTCAAGGGGGTGCAACACTTCTCAGTTTGGGCCTTCTTTT
Anomodon rugelii                   ATGCACTCTTTTATGGGAGGTGGAACACTTCTTAGCTTAGGCTTGGGAAT
Anomodon attenuatus                ATGCACTCTTTTATGGGAGGTGGAACACTTCTTAGCTTAGGCTTGGGAAT
Huperzia squarrosa                 ATGCACTCTTTTATGGGAGGTGGAACACTTCTTAGCTTAGGCTTGGGAAT
Megaceros aenigmaticus             ATGCATTCCTTTACGGGAGGTGGAACACTTCTTAGTTTAGGCTTAGGAAT
Phaeoceros laevis                  ATGTATTCGTTTACGGGAGGTGGAACACTTCTTAGTTTAGGCTTAGGAAT
                                   *** *  * ***   ** **** ** ***  * ** ** *** *     *

PB235_cox3                         TATCCTATATACCATGTTTGTATGGTGGCGCGATGTTCTACGTGAGTCCA
RRIT251_cox3                       TATCCTATATACCATGTTTGTATGGTGGCGCGATGTTCTACGTGAGTCCA
RRII105_cox3                       TATCCTATATACCATGTTTGTATGGTGGCGCGATGTTCTACGTGAGTCCA
BPM24_cox3                         TATCCTATATACCATGTTTGTATGGTGGCGCGATGTTCTACGTGAGTCCA
RRIC110_cox3                       TATCCTATATACCATGTTTGTATGGTGGCGCGATGTTCTACGTGAGTCCA
RRIM600_cox3                       TATCCTATATACCATGTTTGTATGGTGGCGCGATGTTCTACGTGAGTCCA
Citrullus lanatus                  TATCCTATATACCATGTTCGTATGGTGGCGCGATGTTCTACGTGAATCCA
Cucurbita pepo                     TATCCTATATACCATGTTCGTATGGTGGCGCGATGTTCTACGTGAATCCA
Lotus japonicus                    TCTCCTATATACCATGTTTGTATGGTGGCGCGATGTTCTACGTGAATCCA
Millettia pinnata                  TATCCTATATACCATGTTTGTATGGTGGCGCGATGTTCTACGTGAATCCA
Oryza sativa Japonica              TCTCCTTTATACCATGTTCGTATGGTGGCGGGATGTTCTACGTGAATCCA
Arabidopsis thaliana               TATCCTATATACCATGTTCGTATGGTGGCGCGATGTTCTACGTGAATCCA
Brassica napus                     TCTCCTATATACCATGTTCGTATGGTGGCGCGATGTTCTACGTGAATCCA
Beta macrocarpa                    TATCCTATATACCATGTTTGTATGGTGGCGTGATGTTCTACGCGAATCCA
Beta vulgaris maritima             TATCCTATATACCATGTTTGTATGGTGGCGTGATGTTCTACGCGAATCCA
Beta vulgaris vulgaris             TATCCTATATACCATGTTTGTATGGTGGCGTGATGTTCTACGCGAATCCA
Silene latifolia                   TATCCTATATACCATGTTTGTGTGGTGGCGTGATGTTCTACGCGAATCCA
Anomodon rugelii                   GATCCTATATACTATGTTTGTATGGTGGCGCGATGTTATACGTGAATCCA
Anomodon attenuatus                GATCCTATATACTATGTTTGTATGGTGGCGCGATGTTATACGTGAATCCA
Huperzia squarrosa                 GATCCTATATACTATGTTTGTATGGTGGCGCGATGTTATACGTGAATCCA
Megaceros aenigmaticus             GATCCCATATACCATGATTGTATGGCGGCGCGACGTCATACGTGAATCCA
Phaeoceros laevis                  GATCCTATATACCATGATTGTATGGTGGCGCGACGTCATATGTGAATTCA
                                     ***  ***** *** * ** *** **** ** **  ** * ** * **

PB235_cox3                         CGTTGGAAGGACATCATACCAAAGTCGTACAATTAGGACUTCGATATGGT
RRIT251_cox3                       CGTTGGAAGGACATCATACCAAAGTCGTACAATTAGGACUTCGATATGGT
RRII105_cox3                       CGTTGGAAGGACATCATACCAAAGTCGTACAATTAGGACUTCGATATGGT
BPM24_cox3                         CGTTGGAAGGACATCATACCAAAGTCGTACAATTAGGACUTCGATATGGT
RRIC110_cox3                       CGTTGGAAGGACATCATACCAAAGTCGTACAATTAGGACUTCGATATGGT
RRIM600_cox3                       CGTTGGAAGGACATCATACCAAAGTCGTACAATTAGGACNTCGATATGGT
Citrullus lanatus                  CGTTGGAAGGACATCATACCAAAGTCGTACAATTAGGACUTCGATATGGT
Cucurbita pepo                     CGTTGGAAGGACATCATACCAAAGTCGTACAATTAGGACTTCGATATGGG
```

```
Lotus japonicus              CGTTGGAAGGACATCATACCAAAGTCGTACAATTAGGACUTCGATATGGT
Millettia pinnata            CGTTGGAAGGACATCATACCAAAGTCGTACAATTAGGACUTCGATATGGT
Oryza sativa Japonica        CGTTGGAAGGGCATCATACAAAAGCTGTACAATTAGGACCTCGATATGGT
Arabidopsis thaliana         CGTTGGAAGGACATCATACCAAAGTCGTACAATTAGGACUTCGATATGGT
Brassica napus               CGTTGGAAGGACATCATACCAAAGTCGTACAATTAGGACUTCGATATGGT
Beta macrocarpa              CGTTAGAAGGACATCATACCAAAGTCGTACAATTAGGACTTCGATATGGT
Beta vulgaris maritima       CGTTAGAAGGACATCATACCAAAGTCGTACAATTAGGACTTCGATATGGT
Beta vulgaris vulgaris       CGTTAGAAGGACATCATACCAAAGTCGTACAATTAGGACTTCGATATGGT
Silene latifolia             CGTTAGAAGGACATCATACCAAAGTCGTACAATTAGGACTTCGATATGGT
Anomodon rugelii             CTTACGAAGGACATCATACATTTGTGGTCCAATTAGGACTTCGCTATGGT
Anomodon attenuatus          CTTACGAAGGACATCATACATTTGTGGTCCAATTAGGACTTCGCTATGGT
Huperzia squarrosa           CTTACGAAGGACATCATACATTTGTGGTCCAATTAGGACTTCGCTATGGT
Megaceros aenigmaticus       CTTACGAAGGACATCATACATTTATGGTCCAATTAGGACTTCGTTATGGT
Phaeoceros laevis            CTTACGAAGGACATCATACATTTATGGTCCAATTAGGACTTCGTTATGGT
                             * *  ***** ********       ** ********** *** *****

PB235_cox3                   TTTATTCUGTTCATCGTATCGGAGGTTATGTTCUTTTTTGCTTTTTTTTG
RRIT251_cox3                 TTTATTCUGTTCATCGTATCGGAGGTTATGTTCUTTTTTGCTTTTTTTTG
RRII105_cox3                 TTTATTCUGTTCATCGTATCGGAGGTTATGTTCUTTTTTGCTTTTTTTTG
BPM24_cox3                   TTTATTCUGTTCATCGTATCGGAGGTTATGTTCNTTTTTGCTTTTTTTTG
RRIC110_cox3                 TTTATTCUGTTCATCGTATCGGAGGTTATGTTCNTTTTTGCTTTTTTTTG
RRIM600_cox3                 TTTATTCNGTTCATCGTATCGGAGGTTATGTTCNTTTTTGCTTTTTTTTG
Citrullus lanatus            TTTATTCTGTTTATCGTATCGGAGGTTATGTTCUTTTTTGCTTTTTTTUG
Cucurbita pepo               TTTATTCTGTTTATCGTATCGGAGGTGATGTTCUTTTTAGCTTTTTTTUG
Lotus japonicus              TUTATTCUGTTCATCGTATCGGAGGTTATGTTCUTTTTTGCTTTTTTTUG
Millettia pinnata            TUTATTCTGTTCATCGTATCGGAGGTTATGTTCUTTTTTGCTTTTTTTUG
Oryza sativa Japonica        TCTATTCTCTTCATAGTCTCGGAGGTTATGTTCCTTTTTGCTTTTTTTTG
Arabidopsis thaliana         TUTATTCTGTTCATCGTATCGGAGGTTATGTTCTTTTTTGCTTTTTTTTG
Brassica napus               TUTATTCTGTTCATCGTATCGGAGGTTATGTTCTTTTTTGCTTTTTTTTG
Beta macrocarpa              TTTATTTTGTTCATUGTATCGGAGGTTATGTTCTTTTTTGCTTTTTTTTG
Beta vulgaris maritima       TTTATTTTGTTCATUGTATCGGAGGTTATGTTCTTTTTTGCTTTTTTTTG
Beta vulgaris vulgaris       TTTATTTTGTTCATUGTATCGGAGGTTATGTTCTTTTTTGCTTTTTTTTG
Silene latifolia             TTTATTTTGTTCATCGTCTCGGAGGTTATGTTCTTTTTTGCTTTTTTTTG
Anomodon rugelii             ATGATTTTGTTCATCGTGTCTGAAGTCATGTTTTTTTTTAGCTTTCTTTTG
Anomodon attenuatus          ATGATTTTGTTCATCGTGTCTGAAGTCATGTTTTTTTTTAGCTTTCTTTTG
Huperzia squarrosa           ATGATTTTGTTCATCGTGTCTGAAGTCATGTTTTTTTTTAGCTTTCTTTTG
Megaceros aenigmaticus       ATGATTTTGTTTATCGTGTCTGAAGTCACGTTTTTTTTTAGCTTTCCTTTG
Phaeoceros laevis            ATGATTTTGTTTATTGTGTTTGAAGTCATGTTTTTTTTTAGCTTTCTTTCG
                                ***   ** ** ** *  ** ** * ***  **** *****  ** *

PB235_cox3                   GGCTTUTTTTCATTCTTCTTTGGCACCTACGGTAGAGATCGGAGGTATTT
RRIT251_cox3                 GGCTTUTTTTCATTCTTCTTTGGCACCTACGGTAGAGATCGGAGGTATTT
RRII105_cox3                 GGCTTUTTTTCATTCTTCTTTGGCACCTACGGTAGAGATCGGAGGTATTT
BPM24_cox3                   GGCTTNTTTTCATTCTTCTTTGGCACCTACGGTAGAGATCGGAGGTATTT
RRIC110_cox3                 GGCTTNTTTTCATTCTTCTTTGGCACCTACGGTAGAGATCGGAGGTATTT
RRIM600_cox3                 GGCTTNTTTTCATTCTTCTTTGGCACCTACGGTAGAGATCGGAGGTATTT
Citrullus lanatus            GGCTTUTTUTCATTCTTCTTTGGCACCTACGGTAGAGATCGGAGGTATTT
Cucurbita pepo               GGCTTUTTUTCATTCTTCTTTGGCACCCACAGTAGAGATCGGAGGTATTT
Lotus japonicus              GGCTTUTTUTCATTCTTCTTTGGCACCTACGGTAGAGATCGGGGGTATTT
Millettia pinnata            GGCTTUTTUTCATTCTTCTTTGGCACCTACGGTAGAGATCGGAGGTATTT
Oryza sativa Japonica        GGCTTCTTCTCATTCTTCTTTGGCACCTACGGTAGAGATCGGAGGTATTT
Arabidopsis thaliana         GGCTTUTTUTCATTCTTCTTTGGCACCTGCGGTAGAGATCGGAGGTATTT
Brassica napus               GGCTTUTTUTCATTCTTCTTTGGCACCTGCGGTAGAGATCGGAGGTATTT
Beta macrocarpa              GGCTTTTTUTCATTCTTCTTTGGCACCTGCGGTAGAGATCGGAGGTATTT
Beta vulgaris maritima       GGCTTTTTUTCATTCTTCTTTGGCACCTGCGGTAGAGATCGGAGGTATTT
Beta vulgaris vulgaris       GGCTTTTTUTCATTCTTCTTTGGCACCTGCGGTAGAGATCGGAGGTATTT
Silene latifolia             GGCTTTTTUTCATTCTTCTTTGGCACCTGCGGTAGAGATCGGAGGTATTT
Anomodon rugelii             GGCCTTTTTTCATTCTTCTTTGGCACCCACGGTAGAAATTGGAGCTATTC
Anomodon attenuatus          GGCCTTTTTTCATTCTTCTTTGGCACCCACGGTAGAAATTGGAGCTATTC
Huperzia squarrosa           GGCCTTTTTTCATTCTTCTTTGGCACCCACGGTAGAAATTGGAGCTATTC
Megaceros aenigmaticus       GGCCTTCCTTCACCCTCCTTTGGCACCCACGGTAGAGATTGGAGCTATTT
Phaeoceros laevis            GGCTTTTTTTCATTCTTCTTTGGCACCTACGGTAGAGATTGGAGCTATTT
```

```
                                  *** *    ***  ** **********  * ***** ** ** * ****

PB235_cox3                        GGCCCCCAAAAGGGATTGGGGTTTTAGATCCTUGGGAAATCCCTTTTCTT
RRIT251_cox3                      GGCCCCCAAAAGGGATTGGGGTTTTAGATCCTUGGGAAATCCCTTTTCTT
RRII105_cox3                      GGCCCCCAAAAGGGATTGGGGTTTTAGATCCTUGGGAAATCCCTTTTCTT
BPM24_cox3                        GGCCCCCAAAAGGGATTGGGGTTTTAGATCCTUGGGAAATCCCTTTTCTT
RRIC110_cox3                      GGCCCCCAAAAGGGATTGGGGTTTTAGATCCTUGGGAAATCCCTTTTCTT
RRIM600_cox3                      GGCCCCCAAAAGGGATTGGGGTTTTAGATCCTUGGGAAATCCCTTTTCTT
Citrullus lanatus                 GGCCCCCAAAAGGGATTGGGGTTTTAGATCCTUGGGAAATCCCTTTTCTT
Cucurbita pepo                    GGCCCCCAAAAGGGATTGGGGTTTTAGATCCTUGGGAAATCCCTTTTCTT
Lotus japonicus                   GGCCCCCAAAAGGGATTGGGGTTTTAGATCCTUGGGAAATCCCATTTCTT
Millettia pinnata                 GGCCCCCTTTAGGGATTTGGGTTTTAGATCCTUGGGAAATCCCTTTTCTT
Oryza sativa Japonica             GGCCCCCAAAAGGGATTGGGGTTTTAGATCCTTGGGAAATCCCTCTTCTT
Arabidopsis thaliana              GGCCCCCAAAAGGGATTGAGGTTTTAGATCCTTGGGAAATCCCTTTTCTT
Brassica napus                    GGCCCCCAAAAGGGATTGGGGTTTTAGATCCTTGGGAAATCCCTTTTCTT
Beta macrocarpa                   GGCCCCCAAAAGGGATTTGGGTTTTAGATCCTUGGGAAATCCCTTTTCTT
Beta vulgaris maritima            GGCCCCCAAAAGGGATTTGGGTTTTAGATCCTUGGGAAATCCCTTTTCTT
Beta vulgaris vulgaris            GGCCCCCAAAAGGGATTTGGGTTTTAGATCCTUGGGAAATCCCTTTTCTT
Silene latifolia                  GGCCCCCAAAAGGGATTTGGGTTTTAGATCCTTGGGAAATCCCTTTTCTT
Anomodon rugelii                  GGCCCCCCAAAGGAATTGATGTGTTAAATCCTTGGGGAATTCCTTTTCTA
Anomodon attenuatus               GGCCCCCCAAAGGAATTGATGTGTTAAATCCTTGGGGAATTCCTTTTCTA
Huperzia squarrosa                GGCCCCCCAAAGGAATTGATGTGTTAAATCCTTGGGGAATTCCTTTTCTA
Megaceros aenigmaticus            GGTCTCCTAAGGGAATTGATGTGTTAGATCCTTGGGGAATTCCTTTTTTA
Phaeoceros laevis                 GGCCTTCTAAGGGAATTGATGTGTTAGATCCTTGGGGAATTCCTTTTTTA
                                  ** *  *    ** ***   ** *** ***** *** *** **  ** *

PB235_cox3                        AATACCCUTATTCTCCTTTCATCCGGAGCTGCCGTAACTTGGGCTCATCA
RRIT251_cox3                      AATACCCUTATTCTCCTTTCATCCGGAGCTGCCGTAACTTGGGCTCATCA
RRII105_cox3                      AATACCCUTATTCTCCTTTCATCCGGAGCTGCCGTAACTTGGGCTCATCA
BPM24_cox3                        AATACCCUTATTCTCCTTTCATCCGGAGCTGCCGTAACTTGGGCTCATCA
RRIC110_cox3                      AATACCCUTATTCTCCTTTCATCCGGAGCTGCCGTAACTTGGGCTCATCA
RRIM600_cox3                      AATACCCUTATTCTCCTTTCATCCGGAGCTGCCGTAACTTGGGCTCATCA
Citrullus lanatus                 AATACCCUTATTCTCCTTTCATCCGGAGCAGCCGTAACTTGGGCTCATCA
Cucurbita pepo                    AATACCCTGATTCTCCTTTCATCCGGAGCTGCCGTAACTTGGGCTCATCA
Lotus japonicus                   AATACCCUTATTCTCCTTTCATCCGGAGCAGCCGTAACTTGGGCTCATCA
Millettia pinnata                 AATACCCUTATTCTCCTTTCATCCGGAGCAGCCGTAACTTGGGCTCATCA
Oryza sativa Japonica             AATACCCCTATTCTCCCTTCATCCGGAGCTGCCGTAACTTGGGCTCATCA
Arabidopsis thaliana              AATACCCUTATTCTCCUTTCATCCGGAGCTGCCGTAACTTGGGCTCATCA
Brassica napus                    AATACCCUTATTCTCCUTTCATCCGGAGCTGCCGTAACTTGGGCTCATCA
Beta macrocarpa                   AATACCCTTATTCTTCTTTCATCCGGAGCTGCCGTAACTTGGGCTCATCA
Beta vulgaris maritima            AATACCCTTATTCTTCTTTCATCCGGAGCTGCCGTAACTTGGGCTCATCA
Beta vulgaris vulgaris            AATACCCTTATTCTTCTTTCATCCGGAGCTGCCGTAACTTGGGCTCATCA
Silene latifolia                  AATACCCTTATTCTTCTTTCATCCGGAGCTGCCGTAACTTGGGCTCATCA
Anomodon rugelii                  AATACCCTTATTTTACTCTCATCCGGAGCTGCCGTGACTTGGGCTCATCA
Anomodon attenuatus               AATACCCTTATTTTACTCTCATCCGGAGCTGCCGTGACTTGGGCTCATCA
Huperzia squarrosa                AATACCCTTATTTTACTCTCATCCGGAGCTGCCGTGACTTGGGCTCATCA
Megaceros aenigmaticus            AATACACCTATTCTACCTTCATCGGGAGCCGCCATAACTTGGGCTCATCA
Phaeoceros laevis                 AATACACTTATTTTACTTCTATCGGGAGCCGCCATAACTTGGGCTCATCA
                                  ***** *  *** * *    *** ***** *** * **************

PB235_cox3                        TGCTATACTCGCGGGGAAGGAAAAACGAGCAGTTTATGCTTTAGTAGCTA
RRIT251_cox3                      TGCTATACTCGCGGGGAAGGAAAAACGAGCAGTTTATGCTTTAGTAGCTA
RRII105_cox3                      TGCTATACTCGCGGGGAAGGAAAAACGAGCAGTTTATGCTTTAGTAGCTA
BPM24_cox3                        TGCTATACTCGCGGGGAAGGAAAAACGAGCAGTTTATGCTTTAGTAGCTA
RRIC110_cox3                      TGCTATACTCGCGGGGAAGGAAAAACGAGCAGTTTATGCTTTAGTAGCTA
RRIM600_cox3                      TGCTATACTCGCGGGGAAGGAAAAACGAGCAGTTTATGCTTTAGTAGCTA
Citrullus lanatus                 TGCTATACTCGCGGGGAAGGAAAAACGAGCAGTTTACGCTTTAGTAGCTA
Cucurbita pepo                    TGCTATACTCGCGGGGAAGGAAAAAGGAGCTGTTTACGCTTTAGTAGCTA
Lotus japonicus                   TGCTATACTCGCGGGGAAGGAAAAACGAGCAGTTTACGCTTTAGTAGCAA
Millettia pinnata                 TGCTATACTCGCGGGGAAGGAAAAACGAGCAGTTTACGCTTTAGTAGCTA
Oryza sativa Japonica             TGCTATACTCGCGGGGAAGGAAAAACGAGCAGTTTACGCTTTAGTAGCAA
Arabidopsis thaliana              TGCTATACTCGCGGGGAAAGAAAAACGAGCAGTTTATGCTTTAGTAGCTA
```

```
Brassica napus                    TGCTATACTCGCGGGGAAGGAAAAACGAGCAGTTTATGCTTTAGTAGCTA
Beta macrocarpa                   TGCTATACTCGCGGGGAAGCAAAAACGAGCAGTTTACGCTTTAGTAGCTA
Beta vulgaris maritima            TGCTATACTCGCGGGGAAGCAAAAACGAGCAGTTTACGCTTTAGTAGCTA
Beta vulgaris vulgaris            TGCTATACTCGCGGGGAAGCAAAAACGAGCAGTTTACGCTTTAGTAGCTA
Silene latifolia                  TGCTATACTCGCGGGGAAGCAAAAACGAGCAGTTTACGCTTTAGTAGCTA
Anomodon rugelii                  TGCTATATTAGCTGGATTCAAAAAACAAGCTGTTTATGCTTTAGTAGCTA
Anomodon attenuatus               TGCTATATTAGCTGGATTCAAAAAACAAGCTGTTTATGCTTTAGTAGCTA
Huperzia squarrosa                TGCTATATTAGCTGGATTCAAAAAACAAGCTGTTTATGCTTTAGTAGCTA
Megaceros aenigmaticus            TGCTATATTAGCGGGATTGAAAGAAGAAGCTGTTTACGCTTTAGTAGCTA
Phaeoceros laevis                 TGCTATATTAGCGGGATTGAAAGAAGAAGCTGTTTACGCTTTAGTAGCTA
                                  ******* * ** **     ** **  *** ***** *********** *

PB235_cox3                        CCGTTTUACTGGCTCTAGTATTCACTGGCTTTCAAGGAATGGAATATTAT
RRIT251_cox3                      CCGTTTUACTGGCTCTAGTATTCACTGGCTTTCAAGGAATGGAATATTAT
RRII105_cox3                      CCGTTTUACTGGCTCTAGTATTCACTGGCTTTCAAGGAATGGAATATTAT
BPM24_cox3                        CCGTTTUACTGGCTCTAGTATTCACTGGCTTTCAAGGAATGGAATATTAT
RRIC110_cox3                      CCGTTTUACTGGCTCTAGTATTCACTGGCTTTCAAGGAATGGAATATTAT
RRIM600_cox3                      CCGTTTUACTGGCTCTAGTATTCACTGGCTTTCAAGGAATGGAATATTAT
Citrullus lanatus                 CCGTTTUACTGGCTCTAGTATTCACAGGCTTTCAAGGAATGGAATATTAT
Cucurbita pepo                    CCGTTTUACTGGCTCTAGTATTCACAGGCTTTCAAGGAATGGAATATTAT
Lotus japonicus                   CCGTTTUACTGGCTCTAGTATTCACTGGCTTTCAAGGAATGGAATATTAT
Millettia pinnata                 CCGTTTUACTGGCTCTAGTATTCACTGGCTTTCAAGGAATGGAATATTAT
Oryza sativa Japonica             CCGTTTCACTGGCTCTAGTATCCACTGGCTTTCAAGGAATGGAATATTAC
Arabidopsis thaliana              CCGTTTTATTGGCTCTAGTATTTACTGGCTTTCAAGGAATGGAATATTAT
Brassica napus                    CCGTTTTACTGGCTCTAGTATTTACTGGCTTTCAAGGAATGGAATATTAT
Beta macrocarpa                   CCGTTTTACTGGCTCTAGTCTTCACTGGCTTTCAAGGAATGGAATATTAT
Beta vulgaris maritima            CCGTTTTACTGGCTCTAGTCTTCACTGGCTTTCAAGGAATGGAATATTAT
Beta vulgaris vulgaris            CCGTTTTACTGGCTCTAGTCTTCACTGGCTTTCAAGGAATGGAATATTAT
Silene latifolia                  CCGTTTTACTGGCTCTAGTATTCACTGGCTTTCAAGGAATGGAATATTAT
Anomodon rugelii                  CCATTTTGCTGGCTCTAGTCTTCACCGGGTTTCAAGGAATGGAATATGTA
Anomodon attenuatus               CCATTTTGCTGGCTCTAGTCTTCACCGGGTTTCAAGGAATGGAATATGTA
Huperzia squarrosa                CCATTTTGCTGGCTCTAGTCTTCACCGGGTTTCAAGGAATGGAATATGTA
Megaceros aenigmaticus            CTGTTTCGCTGGCTTTAGTTTTCACTGGGCTTCAAGGAATGGAATATGTA
Phaeoceros laevis                 CTGTTTCGCTAGCTTTAGTTTTCACTGGGCTTCAAGGAATGGAATATGTA
                                  *  ***   * *** **** *  ** **  *****************

PB235_cox3                        CAAGCACCCTTCACTATTTCGGATAGTATTTATGGTTCTACCTTTTTCTT
RRIT251_cox3                      CAAGCACCCTTCACTATTTCGGATAGTATTTATGGTTCTACCTTTTTCTT
RRII105_cox3                      CAAGCACCCTTCACTATTTCGGATAGTATTTATGGTTCTACCTTTTTCTT
BPM24_cox3                        CAAGCACCCTTCACTATTTCGGATAGTATTTATGGTTCTACCTTTTTCTT
RRIC110_cox3                      CAAGCACCCTTCACTATTTCGGATAGTATTTATGGTTCTACCTTTTTCTT
RRIM600_cox3                      CAAGCACCCTTCACTATTTCGGATAGTATTTATGGTTCTACCTTTTTCTT
Citrullus lanatus                 CAAGCACCCTTCACTATTTCGGATAGTATTTATGGTTCTACCTTTTTCTT
Cucurbita pepo                    CAAGCACCCTTTACTATTTCTGATAGTATTTATGGTTCTACCTTTTTCTT
Lotus japonicus                   CAAGCACCCTTCACTATTTCGGATAGTATTTATGGTTCTACCTTTTTCTT
Millettia pinnata                 CAAGCACCCTTCACTATTTCGGATAGTATTTATGGTTCTACCTTTTTCTT
Oryza sativa Japonica             CAAGCACCCTCCACTATTTCGGATAGTATTTACGGTTCTACCTTTTTCTT
Arabidopsis thaliana              CAAGCACCCTTCACTATTTCGGATAGTATTTATGGTTCTACCTTTTTUTT
Brassica napus                    CAAGCACCCTTCACTATTTCGGATAGTATTTATGGTTCTACCTTTTTCTT
Beta macrocarpa                   GAAGCACCCTTCACTATTTCGGATAGTATTTATGGTTCTACCTTTTTCTT
Beta vulgaris maritima            GAAGCACCCTTCACTATTTCGGATAGTATTTATGGTTCTACCTTTTTCTT
Beta vulgaris vulgaris            GAAGCACCCTTCACTATTTCGGATAGTATTTATGGTTCTACCTTTTTCTT
Silene latifolia                  CAAGCACCCTTCACTATTTCGGATAGTATTTATGGTTCTACCTTTTTCTT
Anomodon rugelii                  GAAGCACCTTTCACGATTTCTGATGGTATTTATGGTTCTACCTTTTTCTT
Anomodon attenuatus               GAAGCACCTTTCACGATTTCTGATGGTATTTATGGTTCTACCTTTTTCTT
Huperzia squarrosa                GAAGCACCTTTCACGATTTCTGATGGTATTTATGGTTCTACCTTTTTCTT
Megaceros aenigmaticus            GAAGCACCTTTCACCATTTCTGATGGTATTTATGGTTCTACCTTTTTTTT
Phaeoceros laevis                 GAAGCACCTTTCACAATTCCTGATGGTATTTATGGTTTTACCTTTTCTTC
                                   ******* *  ** *** * *** ******* **** ********  *

PB235_cox3                        AGCAACTGGCTTTCATGGTTTTCATGTGATTATAGGTACTCTTTTCTTGA
RRIT251_cox3                      AGCAACTGGCTTTCATGGTTTTCATGTGATTATAGGTACTCTTTTCTTGA
```

```
RRII105_cox3                     AGCAACTGGCTTTCATGGTTTTCATGTGATTATAGGTACTCTTTTCTTGA
BPM24_cox3                       AGCAACTGGCTTTCATGGTTTTCATGTGATTATAGGTACTCTTTTCTTGA
RRIC110_cox3                     AGCAACTGGCTTTCATGGTTTTCATGTGATTATAGGTACTCTTTTCTTGA
RRIM600_cox3                     AGCAACTGGCTTTCATGGTTTTCATGTGATTATAGGTACTCTTTTCTTGA
Citrullus lanatus                AGCAACTGGCTTTCATGGTTTTCATGTGATTATAGGTACTCTTTTCTTGA
Cucurbita pepo                   AGCAACTGGCTTTCATGGGTTTCATGTGATTATAGGTACTCTTTTCTTGA
Lotus japonicus                  AGCAACTGGCTTTCATGGTTTTCATGTGATTATAGGTACTCTTTTCTTGA
Millettia pinnata                AGCAACTGGCTTTCATGGTTTTCATGTGATTATAGGTACTCTTTTCTTGA
Oryza sativa Japonica            AGCAACTGGCTTTCATGGTTTTCATGTGATTATAGGTACTCTTTTCTTGA
Arabidopsis thaliana             AGCAACAGGCTTTCATGGTTTTCATGTGATTATAGGTACTCTTTTCTTGA
Brassica napus                   AGCAACAGGCTTTCATGGTTTTCATGTGATTATAGGTACTCTTTTCTTGA
Beta macrocarpa                  AGCAACAGGCTTTCATGGTTTTCATGTGATTATAGGTACTCTTTTCTTGA
Beta vulgaris maritima           AGCAACAGGCTTTCATGGTTTTCATGTGATTATAGGTACTCTTTTCTTGA
Beta vulgaris vulgaris           AGCAACAGGCTTTCATGGTTTTCATGTGATTATAGGTACTCTTTTCTTGA
Silene latifolia                 AGCAACTGGCTTTCATGGTTTTCATGTGATTATAGGTACTCTTTTCTTGA
Anomodon rugelii                 AGCCACAGGGTTTCATGGTTTTCATGTTATTATAGGTACCATTTTCCTAA
Anomodon attenuatus              AGCCACAGGGTTTCATGGTTTTCATGTTATTATAGGTACCATTTTCCTAA
Huperzia squarrosa               AGCCACAGGGTTTCATGGTTTTCATGTTATTATAGGTACCATTTTCCTAA
Megaceros aenigmaticus           AGCTACGGGTTCTCATGGTTTTCATGCTATTGTGGGTACCATTTTCTTAA
Phaeoceros laevis                AGCTATGGGTTTTCATGGTTTTCATGTTATTGTGGGTACCATTTTCTTAA
                                 *** *  ** * ****** *******  *** * *****  ***** * *

PB235_cox3                       TCATATGTGGTATTCGCCAATATCTTGGTCATCTGACCAAGGAGCATCAC
RRIT251_cox3                     TCATATGTGGTATTCGCCAATATCTTGGTCATCTGACCAAGGAGCATCAC
RRII105_cox3                     TCATATGTGGTATTCGCCAATATCTTGGTCATCTGACCAAGGAGCATCAC
BPM24_cox3                       TCATATGTGGTATTCGCCAATATCTTGGTCATCTGACCAAGGAGCATCAC
RRIC110_cox3                     TCATATGTGGTATTCGCCAATATCTTGGTCATCTGACCAAGGAGCATCAC
RRIM600_cox3                     TCATATGTGGTATTCGCCAATATCTTGGTCATCTGACCAAGGAGCATCAC
Citrullus lanatus                TCATATGTGGTATTCGCCAATATCTTGGTCATCTGACCAAGGAGCATCAC
Cucurbita pepo                   TCATATGTGGTATTCGCCAATATCTTGGTCATCTGACCAAGGAGCATCAC
Lotus japonicus                  TCATATGTGGTATTCGCCAATATCTTGGTCATCTGACCAAGGAGCATCAC
Millettia pinnata                TCATATGTGGTATTCGCCAATATCTTGGTCATCTGACCAAGGAGCATCAC
Oryza sativa Japonica            TCGTATGTGGTATTCGCCAATATCTTGGTCATCTGACCAAGAAGCATCAC
Arabidopsis thaliana             TTATATGTGGTATTCGGCAATATCTTGGTCATCTGACGAAGGAGCATCAC
Brassica napus                   TTATATGTGGTATTCGGCAATATCTTGGTCATCTGACGAAGGAGCATCAC
Beta macrocarpa                  TTATATGTGGTATTCGCCAATATTTTGGTCATCTGACCAAGGAACATCAC
Beta vulgaris maritima           TTATATGTGGTATTCGCCAATATTTTGGTCATCTGACCAAGGAACATCAC
Beta vulgaris vulgaris           TTATATGTGGTATTCGCCAATATTTTGGTCATCTGACCAAGGAACATCAC
Silene latifolia                 TTATATGTGGTATTCGCCAATATTTTGGTCAGATGACCAAGGAACATCAC
Anomodon rugelii                 TAATATGCGCTATTCGTCAATATCTAGGGCATTTTACCCAAACGCATCAC
Anomodon attenuatus              TAATATGCGCTATTCGTCAATATCTAGGGCATTTTACCCAAACGCATCAC
Huperzia squarrosa               TAATATGCGCTATTCGTCAATATCTAGGGCATTTTACCCAAACGCATCAC
Megaceros aenigmaticus           CAACATGTGGTATTCGTGAATATCTGGGTCATTTTACTGAAAAGCATCAC
Phaeoceros laevis                TAATATGTGGTATTTGTGAATATCTGGGTCATTTTACTGAAAAGCATCAC
                                     *** * **** *  ***** * ** **  * **  *    ******

PB235_cox3                       GTTGGCTTTGAAGCAGCTGCATGGTACTGGCATTTTGTAGACGTGGTTUG
RRIT251_cox3                     GTTGGCTTTGAAGCAGCTGCATGGTACTGGCATTTTGTAGACGTGGTTUG
RRII105_cox3                     GTTGGCTTTGAAGCAGCTGCATGGTACTGGCATTTTGTAGACGTGGTTUG
BPM24_cox3                       GTTGGCTTTGAAGCAGCTGCATGGTACTGGCATTTTGTAGACGTGGTTUG
RRIC110_cox3                     GTTGGCTTTGAAGCAGCTGCATGGTACTGGCATTTTGTAGACGTGGTTUG
RRIM600_cox3                     GTTGGCTTTGAAGCAGCTGCATGGTACTGGCATTTTGTAGACGTGGTTNG
Citrullus lanatus                GTTGGCTTTGAAGCTGCTGCATGGTACTGGCATTTTGTAGACGTGGTTUG
Cucurbita pepo                   GTTGGCTTTGAAGCAGCTGCATGGTACTGGCATTTTGTAGACGTGGTTUG
Lotus japonicus                  GTTGGCTTTGAAGCAGCTGCATGGTACTGGCATTTTGTAGACGTGGTTUG
Millettia pinnata                GTTGGCTTTGAAGCAGCTGCATGGTACTGGCATTTTGTAGACGTGGTTTG
Oryza sativa Japonica            GTTGGCTTTGAAGCAGCTGCATGGTACTGGCATTTTGTAGACGTGGTTUG
Arabidopsis thaliana             GTTGGCTTTGAAGCAGCTGCATGGTACTGGCATTTTGTAGACGTAGTTTG
Brassica napus                   GTTGGCTTTGAAGCAGCTGCATGGTACTGGCATTTTGTAGACGTGGTTTG
Beta macrocarpa                  GTTGGCTTTGAAGCAGCTGCATGGTACTGGCATTTTGTAGACGTGGTTTG
Beta vulgaris maritima           GTTGGCTTTGAAGCAGCTGCATGGTACTGGCATTTTGTAGACGTGGTTTG
Beta vulgaris vulgaris           GTTGGCTTTGAAGCAGCTGCATGGTACTGGCATTTTGTAGACGTGGTTTG
```

```
Silene latifolia              GTTGGCTTTGAAGCAGCTGCATGGTACTGGCATTTTGTAGACGTGGTTTG
Anomodon rugelii              TTTGGCTTTGAAGCAGCTGCTTGGTACCGGCATTTTGTTGACGTGGTTTG
Anomodon attenuatus           TTTGGCTTTGAAGCAGCTGCTTGGTACCGGCATTTTGTTGACGTGGTTTG
Huperzia squarrosa            TTTGGCTTTGAAGCAGCTGCTTGGTACCGGCATTTTGTTGACGTGGTTTG
Megaceros aenigmaticus        TTCGGCTTTGAAGCAGCTGCTTGGCACCGGCATTTCGTGGATGTGGTTCG
Phaeoceros laevis             TTTGGCTTTGAAGCAGCTGCTTGGTACTGGCATTTTGTGGATGTGGTTTG
                               * *********** ***** *** ** ******* ** ** ** *** *

PB235_cox3                    GTTATTCCUATTTGTTTCTATCTATTGGTGGGGGGGTATATGA
RRIT251_cox3                  GTTATTCCUATTTGTTTCTATCTATTGGTGGGGGGGTATATGA
RRII105_cox3                  GTTATTCCUATTTGTTTCTATCTATTGGTGGGGGGGTATATGA
BPM24_cox3                    GTTATTCCUATTTGTTTCTATCTATTGGTGGGGGGGTATATGA
RRIC110_cox3                  GTTATTCCUATTTGTTTCTATCTATTGGTGGGGGGGTATATGA
RRIM600_cox3                  GTTATTCCNATTTGTTTCTATCTATTGGTGGGGGGGTATATGA
Citrullus lanatus             GTTATTCCTATTTGTCTCTATCTATTGGTGGGGAGGTATATGA
Cucurbita pepo                GTTATTCCTATTTGTCTCTATCTATTGGTGGGGAGGTATATGA
Lotus japonicus               GTTATTCCUATTTGTCTCTATCTATTGGTGGGGAGGTATATGA
Millettia pinnata             GTTATTCCTATTTGTCTCTATCTATTGGTGGGGAGGTATATGA
Oryza sativa Japonica         GTTATTCCCATTTGTCTCTATCTATTGGTGGGGAGGTATATGA
Arabidopsis thaliana          GTTATTCCTATTTGTCTCTATCTATTGGTGGGGAGGTATATGA
Brassica napus                GTTATTCCTATTTGTCTCTATCTATTGGTGGGGAGGTATATGA
Beta macrocarpa               GTTATTCTTATTTGTCTCTATCTATTGGTGGGGAGGTATATGA
Beta vulgaris maritima        GTTATTCTTATTTGTCTCTATCTATTGGTGGGGAGGTATATGA
Beta vulgaris vulgaris        GTTATTCTTATTTGTCTCTATCTATTGGTGGGGAGGTATATGA
Silene latifolia              GTTATTCTTATTTGTCTCTATCTATTGGTGGGGAGGTATATGA
Anomodon rugelii              GTTATTCCTATTCGTATCCATTTATTGGTGGGGAGGTAATUAA
Anomodon attenuatus           GTTATTCCTATTCGTATCCATTTATTGGTGGGGAGGTAATUAA
Huperzia squarrosa            GTTATTCCTATTCGTATCCATTTATTGGTGGGGAGGTAATUAA
Megaceros aenigmaticus        GTTATTTCTGCTCCTACCCATTCATCGGCGGGGAGGTAATTAA
Phaeoceros laevis             GTTATTTCTGTTTTTATCTATTTATTGGTGGGGAGGTAATTAA
                              ******     *  *  * **  ** ** **** ****    *
```

###################

matR multiple sequence alignment

```
Arabidopsis thaliana          --------------------------------------------------
Brassica napus                ----------------------CCACTCAGGGGGGTGCGGACACACCTGC
Lotus japonicus               --------------------------------------------------
Millettia pinnata             --------------------------------------GGACACACCTGC
Citrullus lanatus             AGAAATCTCCTTTCTTTCTCGTCCACTCAGGGGGGTGCGGACACACCTGC
Cucurbita pepo                --------------------------------------------------
RRIC110_matR                  --------------------------------------------------
Beta macrocarpa               --------------------------------------------------
Beta vulgaris maritima        --------------------------------------------------
Beta vulgaris vulgaris        --------------------------------------------------
Silene latifolia              --------------------------------------------------
BPM24_matR                    --------------------------------------------------
PB235_matR                    --------------------------------------------------
Zea mays mays                 --------------------------------------------------
RRIT251_matR                  --------------------------------------------------
RRII105_matR                  --------------------------------------------------
RRIM600_matR                  --------------------------------------------------
Cycas taitungensis            --------------------------------------------------


Arabidopsis thaliana          -------------GACGGTTACAAGAATG-CGGGGAAGTGAACAGTACCC
Brassica napus                GCGGATTACAGGTGACGGTTACAAGAATGGCGGGGAAGTGAACAGTACCC
Lotus japonicus               GCGGATTACAGGTGACGGTTACAAGAATGGCGGGGAAGTGAACAGTACCC
Millettia pinnata             GCGGATTACAGGTGACGGTTACAAGAATGGCGGGGAAGTGAACAGTACCC
Citrullus lanatus             GCGGATTACAGGTGACGGTTACAAGAATGGCGGGGAAGTGAACAGTACCC
Cucurbita pepo                ----------------GGTTACAAGAATGGCGGGGAAGTGAACAGTACCC
RRIC110_matR                  --------------------------------------------------
Beta macrocarpa               -----------GTGACGGTTACAAGAATGGCGGGGAAGTGAATAGTACCC
```

```
Beta vulgaris maritima          -----------GTGACGGTTACAAGAATGGCGGGGAAGTGAATAGTACCC
Beta vulgaris vulgaris          -----------GTGACGGTTACAAGAATGGCGGGGAAGTGAATAGTACCC
Silene latifolia                -------ACAGGTGACGGTTACAAGAATGGCGGGGAAGTGAATAGTACTC
BPM24_matR                      --------------------------------------------------
PB235_matR                      --------------------------------------------------
Zea mays mays                   --------GAGCGAACTGAGCGAGCCATGAGGCGCCTATCGGCAAGGCTT
RRIT251_matR                    --------------------------------------------------
RRII105_matR                    --------------------------------------------------
RRIM600_matR                    --------------------------------------------------
Cycas taitungensis              --------CTCAGAGCTGCTATTGCGAAAATACTGCTACTGGCGTCCCTC


Arabidopsis thaliana            G-ACGACATTCAGGGATGGATGTAGAC--CCATCGGGCAGGGA--TAATC
Brassica napus                  G-ACGACATTCAGGGATGGATGTAGAC--CCATCGGGCAGGGA--TAATC
Lotus japonicus                 G-ACGACATTCAGGGATGGATGTAGAC--CCATCGGGCAGGGA--TAATC
Millettia pinnata               G-ACGACATTCAGGGATGGATGTAGAC--CCATCGGGCAGGGA--TAATC
Citrullus lanatus               G-ACGACATTCAGGGATGGATGTAGAC--CCATCGGGCAGGGA--TAATC
Cucurbita pepo                  G-ACGACATTCAGGGATGGATGTAGAC--CCATCGGGCAGGGA--TAATC
RRIC110_matR                    --------------------------------------------------
Beta macrocarpa                 G-ACGACATTCAGGGATGGATGTAGAC--CCATCGAGCAGGGA--TAATC
Beta vulgaris maritima          G-ACGACATTCAGGGATGGATGTAGAC--CCATCGAGCAGGGA--TAATC
Beta vulgaris vulgaris          G-ACGACATTCAGGGATGGATGTAGAC--CCATCGAGCAGGGA--TAATC
Silene latifolia                G-ACGACATTCAGGGATGGATGTAGAC--CTATCGAGCAGGGA--TAATC
BPM24_matR                      --------------------------------------------------
PB235_matR                      --------------------------------------------------
Zea mays mays                   GGAAAAGCCTTAAGTTTAGGCTTAGGCTCCCTTCCTTCACTGAACTGATT
RRIT251_matR                    --------------------------------------------------
RRII105_matR                    --------------------------------------------------
RRIM600_matR                    --------------------------------------------------
Cycas taitungensis              CCCTTACTTATTAACGCGGAT-CAGGTCTAAACCAGGCACACC--CGGCT


Arabidopsis thaliana            ATTCCGGTCCTGGGAGAGGTATTGATTACTCGACTAAAAGGAGAGGAACC
Brassica napus                  ATTCCGGTCCTGGGAGAGGTGCT--TTA----------------GCAAC-
Lotus japonicus                 ATTCCGGTCCTGGGAGAGGT------------------------------
Millettia pinnata               ATTCCGGTCCTGGGAGAGGT------------------------------
Citrullus lanatus               ATTCTGGTCCTGGGAGAGGT------------------------------
Cucurbita pepo                  ATTCTGGTCCTGGGAGAGGT------------------------------
RRIC110_matR                    --------------------------------------------------
Beta macrocarpa                 ATTCCGGTCCTGGGAGAGGT------------------------------
Beta vulgaris maritima          ATTCCGGTCCTGGGAGAGGT------------------------------
Beta vulgaris vulgaris          ATTCCGGTCCTGGGAGAGGT------------------------------
Silene latifolia                ATTCCGGTCCTGGGAAAGGT------------------------------
BPM24_matR                      --------------------------------------------------
PB235_matR                      --------------------------------------------------
Zea mays mays                   CACCCGGGTCCAAACCTGCTGAGCTAGCTAATTTTTCCTTTACGAGATC-
RRIT251_matR                    --------------------------------------------------
RRII105_matR                    --------------------------------------------------
RRIM600_matR                    --------------------------------------------------
Cycas taitungensis              GGCTCATTTTTACGAGATGGTTTTCGGCTCCCAGAGCCCCGCCTCCTCTT


Arabidopsis thaliana            CCATCGACTGAAAGAAGAGGAGAGGTGGCGACCATTTTCAAGAACCAAAA
Brassica napus                  ---TCGACTGAAAGAAGAAGAGAGGTGGCGACCATTTTCAAGAACCAAAA
Lotus japonicus                 --------------------------GGCGACCATTCTCAAGAAC-----
Millettia pinnata               --------------------------GGCGACCATTCTCAAGAAC-----
Citrullus lanatus               --------------------------GGCGACCATTCTCAAGAACCAAAA
Cucurbita pepo                  --------------------------GGAGACCATTCTCAAGAACCAAAA
RRIC110_matR                    --------------------------------------------------
Beta macrocarpa                 --------------------------AGCGACCATTCTTAAGAACCAAAA
Beta vulgaris maritima          --------------------------AGCGACCATTCTTAAGAACCAAAA
Beta vulgaris vulgaris          --------------------------AGCGACCATTCTTAAGAACCAAAA
```

```
Silene latifolia              --------------------------AGCGACCATTCTCAAGAACCAAAA
BPM24_matR                    --------------------------------------------------
PB235_matR                    --------------------------------------------------
Zea mays mays                 ---TCGGCTCTTCGGAATGATTGGAGAGAGCCCCGCCTCCTCTTGGTTGG
RRIT251_matR                  --------------------------------------------------
RRII105_matR                  --------------------------------------------------
RRIM600_matR                  --------------------------------------------------
Cycas taitungensis            GCCAGGCGCCTTGTTCGAGCCCGCGAGAAGGGTGATGATACTACTTCCTT


Arabidopsis thaliana          AGACTGAACTGAGGGAAGCCCT---ATGAGTCACTGAAACGACGGCAGGA
Brassica napus                AGACTGAACTGAGGGAAGCCCT---ATGAGTCACTGAAACGAC----GGG
Lotus japonicus               ----TGAGCTGAGGGAAGCCTT---ATGAGTCACTGAAACGACGGCAGGG
Millettia pinnata             ----TGAGCT-----------T---ATGAGTCACTGAAACGACGGCAGGG
Citrullus lanatus             AGACTGAGCTGAGGGAAGCCCT---ATGAGTCACTGAAATGACAGCGGGA
Cucurbita pepo                AGACTGAGCTGAGGGAAGCCCT---ATGAGTCACTGAAATGACAG-----
RRIC110_matR                  --------------------------------------------------
Beta macrocarpa               AGACTGAGCTGAGGGAAGCCCC---ATAAGTCACTGAAACGACGGCAGGC
Beta vulgaris maritima        AGACTGAGCTGAGGGAAGCCCC---ATAAGTCACTGAAACGACGGCAGGC
Beta vulgaris vulgaris        AGACTGAGCTGAGGGAAGCCCC---ATAAGTCACTGAAACGACGGCAGGC
Silene latifolia              AGACTGAGCTGAGGGAAGCCCC---ATAAGTCACTGAAACGACGGCAGGC
BPM24_matR                    --------------------------------------------------
PB235_matR                    --------------------------------------------------
Zea mays mays                 TGCCGGGCCCGGGACTACTTCCTGAAAGAGCCTTTCGTTCGG-GCCGGCA
RRIT251_matR                  --------------------------------------------------
RRII105_matR                  --------------------------------------------------
RRIM600_matR                  --------------------------------------------------
Cycas taitungensis            AAATTCCGGAAAGATTGTTTCC---ATAAATCCGATCCGTTCCGGCAGG-


Arabidopsis thaliana          GGGGGCC--TTTGATTATAAAAAAGG------GGAGCAAAAAACGAGCTT
Brassica napus                TTCCTCC--CTTGATTATCAAAAAGG------GGAGCAAAAAACGAGCTT
Lotus japonicus               GGGCC---GTCGATCTATCAATAGAG------GGAGCAAAAAACGGGCTT
Millettia pinnata             GGGCCTTGATCTATCAATCAATAGAG------GGAGCAAAAAACGGGCTT
Citrullus lanatus             GGAGGGTCCTTGATCTATCAATAGAG------GGAGCAAAAAAGGGGCTT
Cucurbita pepo                --AGGGTCCTTGATCTATCAATAGAGATAGAGGAAGCAAAAAAGGGGCTT
RRIC110_matR                  --------------------------------------------------
Beta macrocarpa               AATGAAA---TTGTCTATCAATAGAG------GGAGCAAAAAACGGGCTT
Beta vulgaris maritima        AATGAAA---TTGTCTATCAATAGAG------GGAGCAAAAAACGGGCTT
Beta vulgaris vulgaris        AATGAAA---TTGTCTATCAATAGAG------GGAGCAAAAAACGGGCTT
Silene latifolia              AATGAAA---TTCTCTATCAATAGAG------GGAGCAAAAAACGGGCTT
BPM24_matR                    --------------------------------------------------
PB235_matR                    --------------------------------------------------
Zea mays mays                 GGAGGGGCCCTGATCTATCAATTGAG------GGAGCAAAAAACAGGCTT
RRIT251_matR                  --------------------------------------------------
RRII105_matR                  --------------------------------------------------
RRIM600_matR                  --------------------------------------------------
Cycas taitungensis            AGGGCCCCTTTCAGCTATCGAAATATATGG-GGGAGCAAAAAACGGGCTT


Arabidopsis thaliana          TTCCCCCCTT-TACAATATGAAGAAAGAAAGAAGGGTTGAAGTTTAGACC
Brassica napus                TTCCCCCCTT-TACAATATGAAGAAAGAAAGAAGGGTTGAAGTTTAGACC
Lotus japonicus               TGCTCCCCTT-TACAATATGAAGAAAGAAATAAGGGTCGAAGTTTAGACC
Millettia pinnata             TGCTCCCCTT-TACAATATGAAGAAAGAAATAAGGGTCGAAGTTTAGACC
Citrullus lanatus             TGCTCCCCTT-TACAATATGAAGAAAGAAATCCGGGTCGAAGTTTAGACC
Cucurbita pepo                TGCTCC-----TACAATATGAAGAAAGAAAGAAGGGTCGAAGTTTAGACC
RRIC110_matR                  --------------------------------------------------
Beta macrocarpa               TGCTCCCCTT-TACAATATGAAGAAAGAAATAAGGGTCGAAGTTTAGACC
Beta vulgaris maritima        TGCTCCCCTT-TACAATATGAAGAAAGAAATAAGGGTCGAAGTTTAGACC
Beta vulgaris vulgaris        TGCTCCCCTT-TACAATATGAAGAAAGAAATAAGGGTUGAAGTTTAGACC
Silene latifolia              TGCTCCCCTT-TACAATATGAAGAAAGAAAGAAGGGTCGAAGTTGAGACC
BPM24_matR                    --------------------------------------------------
```

```
PB235_matR                    --------------------------------------------------
Zea mays mays                 TGCTCCCCTTTTTTTAAATGAAGAAAGAAAGAAGGGTUGAAGTTTAGACC
RRIT251_matR                  --------------------------------------------------
RRII105_matR                  --------------------------------------------------
RRIM600_matR                  --------------------------------------------------
Cycas taitungensis            TGCTCCCCTT-TCCAATAGGAAGAAATAAATAGGGGTTGAAGTCCAGACC


Arabidopsis thaliana          GCTCACAGTAGTTCTACCTATAGAAAAGATCATGAAAGAGGCGATCAGAA
Brassica napus                GCTCACAGTAGTTCTACCTATAGAAAAGATCATGAAAGAGGCGATCAGAA
Lotus japonicus               GCTTACAGTAGTTCTACCTATAGAAAAGATCATGAAAGAGGCGATCAGAA
Millettia pinnata             GCTTACAGTAGTTCTACCTATAGAAAAGATCATGAAAGAGGCGATCAGAA
Citrullus lanatus             GCTCACAGTAGTTCGACCTATAGAAAAGATCATGAAAGAGGCGATCAGAA
Cucurbita pepo                GCTCACACTAGTTCGACCTATAGAAAAGATCATGAAAGAGGCGATCAGAA
RRIC110_matR                  -------------------------------ATGAAAGAGGCGATCAGAA
Beta macrocarpa               GCTCACAGTAGTTCTACCTATAGAAAAGATCATGAAAGAGGCGATCAGAA
Beta vulgaris maritima        GCTCACAGTAGTTCTACCTATAGAAAAGATCATGAAAGAGGCGATCAGAA
Beta vulgaris vulgaris        GCTCACAGTAGTTCTACCTATAGAAAAGATCATGAAAGAGGCGATCAGAA
Silene latifolia              GCTCACAGTAGTTCTGCCTATAGAAAAGATCATGAAAGAGGCGATCAGAA
BPM24_matR                    -------------------------------ATGAAAGAGGCGATCAGAA
PB235_matR                    -------------------------------ATGAAAGAGGCGATCAGAA
Zea mays mays                 GCTUACAGTAGTTCTACCCATAGAAAAGATCATGAAAGAGGCGATCAGAA
RRIT251_matR                  -------------------------------ATGAAAGAGGCGATCAGAA
RRII105_matR                  -------------------------------ATGAAAGAGGCGATCAGAA
RRIM600_matR                  -------------------------------ATGAAAGAGGCGATCAGAA
Cycas taitungensis            GCTCACAGTAGTAGTACCGATAGAAAAGATCATTAAAGAGGCGATAAGAG
                                                             ** *********** *** 

Arabidopsis thaliana          TGGTACTCGAATUCATTTACGATCCCGAGTTTCCAGACACATCGCATTTC
Brassica napus                TGGTACTCGAATCCATTTACGATCCCGAGTTTCCAGACACATCGCATTTC
Lotus japonicus               TGGTACTCGAATUCATTTATGATCCCGAGTTTCCAGACACATCGCACTTC
Millettia pinnata             TGGTACTCGAATUCATTTATGATCCCGAGTTTCCAGACACATCGCACTTC
Citrullus lanatus             TGGTACTCGAATCCATTTACGATCCCGAGTTTCCAGACACATCGCACTTC
Cucurbita pepo                TGGTACTCGAATCCATTTACGATCCCGAGTTTCCAGACACATCGCACTTC
RRIC110_matR                  TGGTACTCGAATUCATTTACGATTCCGAGTTTCCAGACACATCGCACTTC
Beta macrocarpa               TGGTACUCGAATCCATTTACGATCCCGAGTTTCCAGACACATCGCACTTC
Beta vulgaris maritima        TGGTACUCGAATCCATTTACGATCCCGAGTTTCCAGACACATCGCACTTC
Beta vulgaris vulgaris        TGGTACUCGAATCCATTTACGATCCCGAGTTTCCAGACACATCGCACTTC
Silene latifolia              TGGTACUCGAATCCATTTACGATCCCGAGTTTCCAGACACATCGCACTTC
BPM24_matR                    TGGTACTCGAATUCATTTACGATTCCGAGTTTCCAGACACATCGCACTTC
PB235_matR                    TGGTACTCGAATUCATTTACGATTCCGAGTTTCCAGACACATCGCACTTC
Zea mays mays                 TGGTACUCGAATUCATTTACGATCCCGAGTTTCCAGACACATCGCACTTC
RRIT251_matR                  TGGTACTCGAATUCATTTACGATTCCGAGTTTCCAGACACATCGCACTTC
RRII105_matR                  TGGTACTCGAATUCATTTACGATTCCGAGTTTCCAGACACATCGCACTTC
RRIM600_matR                  TGGTACTCGAATNCATTTACGATTCCGAGTTTCCAGACACATCGCACTTC
Cycas taitungensis            CGGTACCCGAATCCATTTACGATCCCGAGTTTCCGGACACATCGCACTTC
                               ***** ***** ****** *** ********** *********** ***

Arabidopsis thaliana          CGCTCGGGTCAAGGCTGCCACTCGGTCCTAAGACGGATCAAAGAAGAGTG
Brassica napus                CGCTCGGGTCAAGGCTGCCACTCGGTCCTAAGACGGATCAAAGAAGAGTG
Lotus japonicus               CGCTCGGGTCGAGGCCGCTACTCGGCCCTAAGACGGATCAAAGAAGAGTG
Millettia pinnata             CGCTCGGGTCGAGGCCGCTACTCGGCCCTAAGACGGATCAAAGAAGAGTG
Citrullus lanatus             CACTCGGGTCGAGGCCGCCACTCGGCCCTAAGACGGATCAAAGAAGAGTG
Cucurbita pepo                CACTCGGGGCGAGGCCGCCACTCGGCCCTAAGACGGATCAAAGAAGAGTG
RRIC110_matR                  CGCTCGGGTCGAGGCTGCCACTCGGTCCTAAGACGGATCAAAGAAGAGTG
Beta macrocarpa               CGCTCGGGTCGAGGCTGCCATTCGGCCCTAAGACGGATTAAAGAAGAGTG
Beta vulgaris maritima        CGCTCGGGTCGAGGCTGCCATTCGGCCCTAAGACGGATTAAAGAAGAGTG
Beta vulgaris vulgaris        CGCTCGGGTCGAGGCTGCCATTCGGCCCTAAGACGGATTAAAGAAGAGTG
Silene latifolia              CGCTCGGGTCGAGGCTGCCACTCGGCCCTAAGACGGATCAAAGAAGAGTG
BPM24_matR                    CGCTCGGGTCGAGGCTGCCACTCGGTCCTAAGACGGATCAAAGAAGAGTG
PB235_matR                    CGCTCGGGTCGAGGCTGCCACTCGGTCCTAAGACGGATCAAAGAAGAGTG
Zea mays mays                 CGCTCGGGTCGAGGCTGCCACTCGGCCCTCAGACGGATCAAAGAAGAGTG
```

```
RRIT251_matR                    CGCTCGGGTCGAGGCTGCCACTCGGTCCTAAGACGGATCAAAGAAGAGTG
RRII105_matR                    CGCTCGGGTCGAGGCTGCCACTCGGTCCTAAGACGGATCAAAGAAGAGTG
RRIM600_matR                    CGCTCGGGTCGAGGCTGCCACTCGGTCCTAAGACGGATCAAAGAAGAGTG
Cycas taitungensis              CGCCCGGGTCGAGGCTGCCACTCGGCCCTAATACGGATAAAAAAAGAGCG
                                * * **** * **** ** * **** *** * ****** *** ***** *

Arabidopsis thaliana            GGGAATCTCTCGCTGGTTTTTAGAATTCGACATCAGGAAGTGTTTTCACA
Brassica napus                  GGGAATCTCTCGCTGGTTTTTAGAATTCGACATCAGGAAGTGTTTTCACA
Lotus japonicus                 GGGAACCTCTCGCTGGTTTTTGGAATTCGACATCAGGAAGTGTTTTCACA
Millettia pinnata               GGGAACCTCTCGCTGGTTTTTGGAATTCGACATCAGGAAGTGTTTTCACA
Citrullus lanatus               GGGAACCTCTCGCTGGTTTTTGGAATTCGACATCAGGAAGTGTTTTCACA
Cucurbita pepo                  GGGAACCTCTCGCTGGTTTTTGGAATTCGACATCAGGAAGTGTTTTCACA
RRIC110_matR                    GGGAACCTCTCGCTGGTTTTTAGAATTCGACATCAGGAACTATTTTCACA
Beta macrocarpa                 GGGAACCTCTCGCTGGTTTTTGGAATTCGACATCAGGAAGTGTTTTCACA
Beta vulgaris maritima          GGGAACCTCTCGCTGGTTTTTGGAATTCGACATCAGGAAGTGTTTTCACA
Beta vulgaris vulgaris          GGGAACCTCTCGCTGGTTTTTGGAATTCGACATCAGGAAGTGTTTTCACA
Silene latifolia                GGGAACCTCTCGCTGGTTTTTGGAATTCGACATCAGGAAGTGTTTTCACA
BPM24_matR                      GGGAACCTCTCGCTGGTTTTTAGAATTCGACATCAGGAACTATTTTCACA
PB235_matR                      GGGAACCTCTCGCTGGTTTTTAGAATTCGACATCAGGAACTATTTTCACA
Zea mays mays                   GGGAACCTCTCGCTGGTTTTTGGAATTCGACATCAGGAAGTGTTTTCACA
RRIT251_matR                    GGGAACCTCTCGCTGGTTTTTAGAATTCGACATCAGGAACTATTTTCACA
RRII105_matR                    GGGAACCTCTCGCTGGTTTTTAGAATTCGACATCAGGAACTATTTTCACA
RRIM600_matR                    GGGAACCTCTCGCTGGTTTTTAGAATTCGACATCAGGAACTATTTTCACA
Cycas taitungensis              GGGAACCTCTCGTTGGTTCTTGGAATTCGGCATCCTAAAGTGTTTTCACA
                                ***** ****** ***** ** ******* ****   ** * ********

Arabidopsis thaliana            CCATCGACCGACATCGACTCATCUAAATTTTGAAGGAAGAGATCGACGAT
Brassica napus                  CCATCGATCGACATCGACTCATCCAAATTTTGAAGGAAGAGATCGACGAT
Lotus japonicus                 CCATCGACCGACATCGACTCATCUCAATCTTTAAGGAAGAGATCGACGAT
Millettia pinnata               CCATCGACCGACATCGACTCATCUCAATCTTTAAGGAAGAGATCGACGAT
Citrullus lanatus               CCATCGACCGACATCGACTCATCTCAATCTTTAAGGAAGAGATCGACGAT
Cucurbita pepo                  CCATCGACCGACATCGACTCATCTCAATCTTGAAGGAAGAGATCGACGAT
RRIC110_matR                    CCATCGACCGGCATCGACTCATCTCAATCTTTAAGGAAGAGATCGACGAT
Beta macrocarpa                 CCATCGACCGACATCGACTCATCUCAATCTTTAAGGAAGAGATCGACGAT
Beta vulgaris maritima          CCATCGACCGACATCGACTCATCUCAATCTTTAAGGAAGAGATCGACGAT
Beta vulgaris vulgaris          CCATCGACCGACATCGACTCATCUCAATCTTTAAGGAAGAGATCGACGAT
Silene latifolia                CCATCGACCGACATCGACTCATAUCAATCTTTAAGGAAGAGATCGACGAT
BPM24_matR                      CCATCGACCGGCATCGACTCATCTCAATCTTTAAGGAAGAGATCGACGAT
PB235_matR                      CCATCGACCGGCATCGACTCATCTCAATCTTTAAGGAAGAGATCGACGAT
Zea mays mays                   CCATCGACCGACATCGATTCATCTCAATCTTGAAGGAAGAGATCGACGAT
RRIT251_matR                    CCATCGACCGGCATCGACTCATCTCAATCTTTAAGGAAGAGATCGACGAT
RRII105_matR                    CCATCGACCGGCATCGACTCATCTCAATCTTTAAGGAAGAGATCGACGAT
RRIM600_matR                    CCATCGACCGGCATCGACTCATCTCAATCTTTAAGGAAGAGATCGACGAT
Cycas taitungensis              CCATCGACCGGCATCGACTCATCTCAATCTTTAAGGAAGAGATCGACGAT
                                ******* ** ****** ****   *** ** ******************

Arabidopsis thaliana            CCCAAGTTCTTTTACTUCATTCAGAAAGTATTTTCCGCCGGACGACTCGT
Brassica napus                  CCCAAGTTCTTTTACTCCATTCATAAAGTCTTTTCCGCCGGACGACTCGT
Lotus japonicus                 CCCAAGTTCTTTTACTCCATTCATAAAGTCTTTTCCGCCGGACGACTCGT
Millettia pinnata               CCCAAGTTCTTTTACTCCATTCATAAAGTCTTTTCCGCCGGACGACTCGT
Citrullus lanatus               CCCAAGTTCTTTTACTCCATTCAGAAAGTCTTTTCCGTCGGACGACTCGT
Cucurbita pepo                  CCCAAGTTCTTTTACTCCATTCATCAAGTCTTTTCCGGCAGACGACTCGT
RRIC110_matR                    CCCAAGTTCTTTTACTCCATTCAGAAAGTCTTTTCCGCCGGACGACTCGT
Beta macrocarpa                 CCCAAGTTCTTTTACTCGATTCAGAAAGTCTTTTCTGCCGGACGACTCGT
Beta vulgaris maritima          CCCAAGTTCTTTTACTCGATTCAGAAAGTCTTTTCTGCCGGACGACTCGT
Beta vulgaris vulgaris          CCCAAGTTCTTTTACTCGATTCAGAAAGTCTTTTCTGCCGGACGACTCGT
Silene latifolia                CCCAAGTTCTTTTACTCCATTCAGAAAGTCTTTTCCGCCGGACGACTCGT
BPM24_matR                      CCCAAGTTCTTTTACTCCATTCAGAAAGTCTTTTCCGCCGGACGACTCGT
PB235_matR                      CCCAAGTTCTTTTACTCCATTCAGAAAGTCTTTTCCGCCGGACGACTCGT
Zea mays mays                   TCCAAGTTCTTTTACUUCACTCAGAAACAGTTTTCCGCCGGACGACTCGT
RRIT251_matR                    CCCAAGTTCTTTTACTCCATTCAGAAAGTCTTTTCCGCCGGACGACTCGT
RRII105_matR                    CCCAAGTTCTTTTACTCCATTCAGAAAGTCTTTTCCGCCGGACGACTCGT
```

```
RRIM600_matR                      CCCAAGTTCTTTTACTCCATTCAGAAAGTCTTTTCCGCCGGACGACTCGT
Cycas taitungensis                CCCAAGTTCTTTTACCCCACTCATAAACTGCTTTCCGCCGGACGACTTGT
                                   **************   * ***  **    **** * * ******* **

Arabidopsis thaliana              AGGAGTTGAGAGGGG---CCCT---TACTCCGTCCCACACAGTGTACTAC
Brassica napus                    AGGAGTTGAGAGGGG---CCCT---TACTCCGTCCCACACAGTGTACTAC
Lotus japonicus                   AGGAGGTGAGAAGGG---CCCT---TACTCCGTCCCACACAGTGTACTAC
Millettia pinnata                 AGGAGGTGAGAAGGG---CCCT---TACTCCGTCCCACACAGTGTACTAC
Citrullus lanatus                 AGGAGGTGAGAAGGG---CCCTTACTACTCCGTCCCACACAGTGTACTAC
Cucurbita pepo                    AGGAGGTGAGAAGGG---CCCTGACTACTCCGTCCCACACAGTGTACTAC
RRIC110_matR                      AGGAGGTGAGAAGGC---CCCT---TACTCCGTCCCACACAGTGTACTAC
Beta macrocarpa                   AGGAGGTGAGAAGGG---CCCT---TACTCCGTCCCACACAGTGTACTAC
Beta vulgaris maritima            AGGAGGTGAGAAGGG---CCCT---TACTCCGTCCCACACAGTGTACTAC
Beta vulgaris vulgaris            AGGAGGTGAGAAGGG---CCCT---TACTCCGTCCCACACAGTGTACTAC
Silene latifolia                  AGGAGGTGAGAAGGG---AAAA---GACTCCGTCCCACACAGTGTACTAC
BPM24_matR                        AGGAGGTGAGAAGGC---CCCT---TACTCCGTCCCACACAGTGTACTAC
PB235_matR                        AGGAGGTGAGAAGGC---CCCT---TACTCCGTCCCACACAGTGTACTAC
Zea mays mays                     AGGAGGTGAGAAGGG---CCCT---GACTCCGTCCCAAACAGTGTACTAC
RRIT251_matR                      AGGAGGTGAGAAGGC---CCCT---TACTCCGTCCCACACAGTGTACTAC
RRII105_matR                      AGGAGGTGAGAAGGC---CCCT---TACTCCGTCCCACACAGTGTACTAC
RRIM600_matR                      AGGAGGTGAGAAGGC---CCCT---TACTCCGTCCCACACAGTGTACTAC
Cycas taitungensis                AGGAGGTGAGAAGGGTGGCCGCCCTTCCTCCGTCCCACACAGTGTACTAC
                                  ***** ***** **             ********** ************

Arabidopsis thaliana              TATCGGCCCTACUAGGCAACATCTACCTACACAAGCTCGATCAGGAGATA
Brassica napus                    TATCGGCCCTACUAGGCAACATCTACCTACACAAGCTCGATCAGGAGATA
Lotus japonicus                   TATCGGCCCTACUAGGCAACATCTACCTACACAAGCTCGATCAGGAGATA
Millettia pinnata                 TATCGGCCCTACUAGGCAACATCTACCTACACAAGCTCGATCAGGAGATA
Citrullus lanatus                 TATCGGCCCTACCAGGCAACATCTACCTACACAAGCTCGATCAGGAGATA
Cucurbita pepo                    TATCGGCCCTACCAGGCAACATCTACCTACACAAGCTCGATCAGGAGATA
RRIC110_matR                      TATCGGCCCTACUAGGCAACATCTACCTACACAAGCTCGATCAGGAGATA
Beta macrocarpa                   TATCGGCCCTACUAGGCAACATCTACTTACACAAGCTCGATCAGGAGATA
Beta vulgaris maritima            TATCGGCCCTACUAGGCAACATCTACTTACACAAGCTCGATCAGGAGATA
Beta vulgaris vulgaris            TATCGGCCCTACUAGGCAACATCTACTTACACAAGCTCGATCAGGAGATA
Silene latifolia                  TATCGGCCCTACUGGGCAACATCTACTTACACAAGCTCGATCAGGAGATA
BPM24_matR                        TATCGGCCCTACUAGGCAACATCTACCTACACAAGCTCGATCAGGAGATA
PB235_matR                        TATCGGCCCTACUAGGCAACATCTACCTACACAAGCTCGATCAGGAGATA
Zea mays mays                     TATCGGCCCTATTAGGCAATATCTACCTACACAAGCTCGATCAGGAGATA
RRIT251_matR                      TATCGGCCCTACNAGGCAACATCTACCTACACAAGCTCGATCAGGAGATA
RRII105_matR                      TATCGGCCCTACUAGGCAACATCTACCTACACAAGCTCGATCAGGAGATA
RRIM600_matR                      TATCGGCCCTACUAGGCAACATCTACCTACACAAGCTCGATCAGGAGATA
Cycas taitungensis                TATCGGCCCTACCAGGCAACATCTACCCACACAAGCTCGATCAGGAGATG
                                  ***********   ***** ******  *********************

Arabidopsis thaliana              GGGAGGATCCGACAGAAGTACGAAATTCCGATTGTTCAGAGAGTCAGATC
Brassica napus                    GGGAGGATCCGACAGAAGTATGAAATTCCGATTGTTCAGAGAGTCAGATU
Lotus japonicus                   GGAAGGATCCGACAAAAGTACGAAATTCCGA---------GAATAAGATC
Millettia pinnata                 GGAAGGATCCGACAAAAGTACGAAATTCCGA---------GAATAAGATC
Citrullus lanatus                 GGGAGGATCCGACAGAAGTACGAAATTCCGATTGTTCAGAGAATCAGATU
Cucurbita pepo                    GGGAGGATCCAACAGAAGTACGAAATTCCGATTGTTCAGAGAATCAGATC
RRIC110_matR                      GGGAGGATCCGACAGAAGTACGAAATTCCGATTGTTCAGAGAATCAGATC
Beta macrocarpa                   GGGAGGATTCGACAGAAGTACGAAATTCCGATTGTTCAGAGAATCAGATU
Beta vulgaris maritima            GGGAGGATTCGACAGAAGTACGAAATTCCGATTGTTCAGAGAATCAGATU
Beta vulgaris vulgaris            GGGAGGATTCGACAGAAGTACGAAATTCCGATTGTTCAGAGAATCAGATU
Silene latifolia                  GGAAGGATCCGACAGAAGTACGAAATTCCTATTGTTCAGAGAATCAGATU
BPM24_matR                        GGGAGGATCCGACAGAAGTACGAAATTCCGATTGTTCAGAGAATCAGATC
PB235_matR                        GGGAGGATCCGACAGAAGTACGAAATTCCGATTGTTCAGAGAATCAGATC
Zea mays mays                     GGGAGGATCCGGCAGAAGCACGAAATTCCTCTTGTAGTGAAAATAAGATC
RRIT251_matR                      GGGAGGATCCGACAGAAGTACGAAATTCCGATTGTTCAGAGAATCAGATC
RRII105_matR                      GGGAGGATCCGACAGAAGTACGAAATTCCGATTGTTCAGAGAATCAGATC
RRIM600_matR                      GGGAGGATCCGACAGAAGTACGAAATTCCGATTGTTCAGAGAATCAGATC
Cycas taitungensis                GAGAGGATCCGACAGAAGCACGAAATTCCGATTGTTCAGATAATCAAATT
```

```
                                  *  ***** *  ** *** * ********            * * * **

Arabidopsis thaliana              GGTTCTATTAAGGACAGGTCGTCGTATTGATG------------------
Brassica napus                    GGTTCTATTAAGGACAGGTCGTCGTATTGATG------------------
Lotus japonicus                   GGTTCTATTAAGGACAGGTC---GTATTGATG------------------
Millettia pinnata                 GGTTCTATTAAGGACAGGTC---GTATTGATG------------------
Citrullus lanatus                 GGTTCTATTAAGGACAGGTC---GTATTGATG------------------
Cucurbita pepo                    GGTTCTATTAAGGACAGGT---------GATG------------------
RRIC110_matR                      GGTTCTATTAAGGACAGGTC---GTATTGATG------------------
Beta macrocarpa                   GGTTCTATTAAAGACAAGTC---GTATTGATG------------------
Beta vulgaris maritima            GGTTCTATTAAAGACAAGTC---GTATTGATG------------------
Beta vulgaris vulgaris            GGTTCTATTAAAGACAAGTC---GTATTGATG------------------
Silene latifolia                  GGTTCTATTAAAGACAAGTC---GTATTGATGTAAAGACAAGTCGTATTG
BPM24_matR                        GGTTCTATTAAGGACAGGTC---GTATTGATG------------------
PB235_matR                        GGTTCTATTAAGGACAGGTC---GTATTGATG------------------
Zea mays mays                     GGTTCTATTAAGAATAGGTCGTCGTATTGATG------------------
RRIT251_matR                      GGTTCTATTAAGGACAGGTCGT---ATTGATG------------------
RRII105_matR                      GGTTCTATTAAGGACAGGTC---GTATTGATG------------------
RRIM600_matR                      GGTTCTATTAAGGACAGGTC---GTATTGATG------------------
Cycas taitungensis                GGTTCCATTAAAGACAGGTC---GTATTGATG------------------
                                  ***** *****  * * **         ****

Arabidopsis thaliana              ---ACCAAGAAAACCCTGGAGAAGAAGCAAGCTTCAACGCTCCCCAAGAC
Brassica napus                    ---ACCAAGAAAACCCTGGAGAAGAAGCAAGCTTCAACGCTCCCCAAGAC
Lotus japonicus                   ---ACCAAGAAAACTCTGGAGAAGAAGCAAGCTTCAACGCTCCCCAAGAC
Millettia pinnata                 ---ACCAAGAAAACTCAGGAGAAGAAGCAAGCTTCAACGCTCCCCAAGAC
Citrullus lanatus                 ---ACCAAGAAAACTCTGGAGAAGAAGCAAGCTTCAACGCTCCCCAAGAC
Cucurbita pepo                    ---ACCAATCAAACTCGGGAGAAGAAGCAAGCTTCAACGCTCCCCAAGAC
RRIC110_matR                      ---ACCAAGAAAATTCTGGAGAAGAAGCAAGCTTCAACGCTCCCCAAGGC
Beta macrocarpa                   ---ACCAAGAAAACTCTGGAGAAGAAGAAAGCTTCAACGCTCCACAAGAC
Beta vulgaris maritima            ---ACCAAGAAAACTCTGGAGAAGAAGAAAGCTTCAACGCTCCACAAGAC
Beta vulgaris vulgaris            ---ACCAAGAAAACTCTGGAGAAGAAGAAAGCTTCAACGCTCCACAAGAC
Silene latifolia                  ATGACCAAGAAAACTCTGGAGAAGAAGAAAGCTTCAACGCTCCCCAAGAC
BPM24_matR                        ---ACCAAGAAAATTCTGGAGAAGAAGCAAGCTTCAACGCTCCCCAAGGC
PB235_matR                        ---ACCAAGAAAATTCTGGAGAAGAAGCAAGCTTCAACGCTCCCCAAGGC
Zea mays mays                     ---ACCAAGAAAAGTATGGAAAAGAAGCAAGCTTCAACGCTCCCCAAGAC
RRIT251_matR                      ---ACCAAGAAAATTCTGGAGAAGAAGCAAGCTTCAACGCTCCCCAAGGC
RRII105_matR                      ---ACCAAGAAAATTCTGGAGAAGAAGCAAGCTTCAACGCTCCCCAAGGC
RRIM600_matR                      ---ACCAAGAAAATTCTGGAGAAGAAGCAAGCTTCAACGCTCCCCAAGGC
Cycas taitungensis                ---ACCAAGAAAACTCTGGAGAAGAAGCAAGCTTCAACTCTCCCGGAGAC
                                     *****  ***    *** ****** ********** ****   ** *

Arabidopsis thaliana              AACAGAGCCATCATTGTGGGGAGCGTTAAGAGCATGCAACGCAAAGCGGC
Brassica napus                    AACAGAGCCATCATTGTGGGGAGCGTTAAGAGCATGCAACGCAAAGCGGC
Lotus japonicus                   AACAGAGCCATCATTGTGGGCAGGGTCAAGAGCATTCAACGCAAAGCGGC
Millettia pinnata                 AACAGAGCCATCATTGTGGGCAGGGTCAAGAGCATTCAACGCAAAGCGGC
Citrullus lanatus                 TACAGAGCCCTCATTATGGGGAGGGTAAAGAGCTTCCAACGCAAAGCGGC
Cucurbita pepo                    TACAGAGCCCTCATTATGGGGAGGGTAAAGAGCTTCCAACGCAAAGCGGC
RRIC110_matR                      AACAGAGCCATTATTGTGGGGAGGGTAAAGAGCATCCAACGCAAAGCGGC
Beta macrocarpa                   AACAGAGCCATCATTGTGGATAGGGTAAAAAGCATCCAACGCAAAGCGGC
Beta vulgaris maritima            AACAGAGCCATCATTGTGGATAGGGTAAAAAGCATCCAACGCAAAGCGGC
Beta vulgaris vulgaris            AACAGAGCCATCATTGTGGATAGGGTAAAAAGCATCCAACGCAAAGCGGC
Silene latifolia                  AACAGAGCCATCATTGTGGATAGGGTAAAAAGCATCCAACGCAAAGCGGC
BPM24_matR                        AACAGAGCCATTATTGTGGGGAGGGTAAAGAGCATCCAACGCAAAGCGGC
PB235_matR                        AACAGAGCCATTATTGTGGGGAGGGTAAAGAGCATCCAACGCAAAGCGGC
Zea mays mays                     AACAGAGCCCTCATAGTGGGGAGGGTAAAGAGCATCCAACGCAAAGCGAC
RRIT251_matR                      AACAGAGCCATTATTGTGGGGAGGGTAAAGAGCATCCAACGCAAAGCGGC
RRII105_matR                      AACAGAGCCATTATTGTGGGGAGGGTAAAGAGCATCCAACGCAAAGCGGC
RRIM600_matR                      AACAGAGCCATTATTGTGGGGAGGGTAAAGAGCATCCAACGCAAAGCGGC
Cycas taitungensis                AACAGAGCCATCGTTGTGGGGAGGGTAAAAAGCATCCAGCGCAAAGCGGC
                                   ******** *  *  ***  ** ** ** *** * ** ********* *
```

```
Arabidopsis thaliana              CTTTCATTCCCTTGTTTCGTCGTGGCACACCCCCCCCACAAGCACCCTCC
Brassica napus                    CTTTCATTCCCTTGTTTCGTCGTGGCACACCCCCCCCACAAGCACCCTCC
Lotus japonicus                   CTTTCATTCCCTTGTTTCGTCGTGGCACACCCCCCCCACAAGCACCCCCC
Millettia pinnata                 CTTTCATTCCCTTGTTTCGTCGTGGCACACCCCCCCCACAAGCACCCCCC
Citrullus lanatus                 CTTTCATTCCCTTGTTTCGTCGTGGCACACCCCCCACACAAGCACCACCC
Cucurbita pepo                    CTTTCATTCCCTTGTTTCGTCGTGGCACACCCCACACACAAGCACCACAC
RRIC110_matR                      CTTTCATTCCCTTGTTTCGTCGTGGCACACCCCCCCCACAAGCACCCCCA
Beta macrocarpa                   CTTTCATTCCCTTGTTTCGTCGTGGCACACCCTCCCCGCAAGCACCCCCC
Beta vulgaris maritima            CTTTCATTCCCTTGTTTCGTCGTGGCACACCCTCCCCGCAAGCACCCCCC
Beta vulgaris vulgaris            CTTTCATTCCCTTGTTTCGTCGTGGCACACCCTCCCCGCAAGCACCCCCC
Silene latifolia                  CTTTCATTCCCTTGTTTCGTCGTGGCACAACCCCCCCGCAAGCACCCCCC
BPM24_matR                        CTTTCATTCCCTTGTTTCGTCGTGGCACACCCCCCCCACAAGCACCCCCA
PB235_matR                        CTTTCATTCCCTTGTTTCGTCGTGGCACACCCCCCCCACAAGCACCCCCA
Zea mays mays                     CTTTCATTCCCTTGTTTCGTCGTGGCACACCCCCCCCACAAGCACCCCCA
RRIT251_matR                      CTTTCATTCCCTTGTTTCGTCGTGGCACACCCCCCCCACAAGCACCCCCA
RRII105_matR                      CTTTCATTCCCTTGTTTCGTCGTGGCACACCCCCCCCACAAGCACCCCCA
RRIM600_matR                      CTTTCATTCCCTTGTTTCGTCGTGGCACACCCCCCCCACAAGCACCCCCA
Cycas taitungensis                GTTTTATTCCCTTGTTTTGTCGTGGCACACCCCCCCCACAAGCACCCCCC
                                   *** ************ *********** **  * * ********

Arabidopsis thaliana              GGCTCAGGGGGGACCAGAAAAGGCCTTTCGTTTTCCCCCCTTCGTCGG--
Brassica napus                    GGCTCAGGGGGGACCAGAAAAGGCCTTTCGTTTTCCCCCCTTCGTCGG--
Lotus japonicus                   GGCGAAGGGGGGACCAGAAAACGCCTTTCGTTTTCCCCCCTTCGTCGG--
Millettia pinnata                 GGCGAAGGGGGGACCAGAAAACGCCTTTCGTTTTCCCCCCTTCGTCGG--
Citrullus lanatus                 GGCTCAGGGGGGACCAGAAAACGCCTTTCGTTTTCCCCCCTTCGTCGG--
Cucurbita pepo                    GGCTCAGGGGAGACCAGAAAACGCCTTTCGGTTTACCCAC------GG--
RRIC110_matR                      GGCCCAGGGGGGACCAGAAAACGCCTTTCGTTTTCCCCCCTTCGTCGG--
Beta macrocarpa                   GGCTCAGGGGGGACCAGAAAAGGCCTTTCGTTTTCCCCCCTTCGTCGG--
Beta vulgaris maritima            GGCTCAGGGGGGACCAGAAAAGGCCTTTCGTTTTCCCCCCTTCGTCGG--
Beta vulgaris vulgaris            GGCTCAGGGGGGACCAGAAAAGGCCTTTCGTTTTCCCCCCTTCGTCGG--
Silene latifolia                  GGCTCAGGGGGGACCAGACCTCTCCTTTCGTTTTGCCCCCTTCGTCGG--
BPM24_matR                        GGCCCAGGGGGGACCAGAAAACGCCTTTCGTTTTCCCCCCTTCGTCGG--
PB235_matR                        GGCCCAGGGGGGACCAGAAAACGCCTTTCGTTTTCCCCCCTTCGTCGG--
Zea mays mays                     GGCGAAGGGGAGACCAGAAAACGCCTTTCGTTTTCCCTCCTTCC------
RRIT251_matR                      GGCCCAGGGGGGACCAGAAAACGCCTTTCGTTTTCCCCCCTTCGTCGG--
RRII105_matR                      GGCCCAGGGGGGACCAGAAAACGCCTTTCGTTTTCCCCCCTTCGTCGG--
RRIM600_matR                      GGCCCAGGGGGGACCAGAAAACGCCTTTCGTTTTCCCCCCTTCGTCGG--
Cycas taitungensis                GACGAAGGGGAGACCAGAAAACGCCTTTAGTTTTCCCCCCTTCGTCGGGG
                                  * *  ***** *******     ***** * *** **  *

Arabidopsis thaliana              ----CCCTTGCCGTCTTCCTTAACAAGCCCTCGAGCCTTCTTTGCGCCGC
Brassica napus                    ----CCCTTGCCGTCTTCCTTAACAAGCCCTCGAGCCTTCTTTGCGCCGC
Lotus japonicus                   ----CCCTTGCCGCCTTCCTTAACAAGCCCTCGAGCCTCCTTTGCGCCGC
Millettia pinnata                 ----CCCTTGCCGCCTTCCTTAACAAGCCCTCGAGCCTCCTTTGCGCCGC
Citrullus lanatus                 ----CCCTTGCCGCCTTCCTTAACAAGCCCTCGAGCCTCCTTTGCGCCGC
Cucurbita pepo                    ----CCCTTGCCGCCTTCCTTAACAAGCCCTCGAGCCTCCTTTGCGCCGC
RRIC110_matR                      ----CACTTGCCGCCTTTCTTAACAAGCCCTCGAGCCTCCTTTGCGCCGC
Beta macrocarpa                   ----CCCTTACCGCCTTCCTTAACAAGCCCTCGAGCCTCCTTTTCGCTGC
Beta vulgaris maritima            ----CCCTTACCGCCTTCCTTAACAAGCCCTCGAGCCTCCTTTTCGCTGC
Beta vulgaris vulgaris            ----CCCTTACCGCCTTCCTTAACAAGCCCTCGAGCCTCCTTTTCGCTGC
Silene latifolia                  ----CCCTTGCCGCCTTCCTTAACAAGCCCTCGAGCCTCCTTTTCGCTGC
BPM24_matR                        ----CACTTGCCGCCTTTCTTAACAAGCCCTCGAGCCTCCTTTGCGCCGC
PB235_matR                        ----CACTTGCCGCCTTTCTTAACAAGCCCTCGAGCCTCCTTTGCGCCGC
Zea mays mays                     ------CTAGCCGCCTTCCTTAACAAGCCCTCGAGCCTCCTTTGCGCCGC
RRIT251_matR                      ----CACTTGCCGCCTTTCTTAACAAGCCCTCGAGCCTCCTTTGCGCCGC
RRII105_matR                      ----CACTTGCCGCCTTTCTTAACAAGCCCTCGAGCCTCCTTTGCGCCGC
RRIM600_matR                      ----CACTTGCCGCCTTTCTTAACAAGCCCTCGAGCCTCCTTTGCGCCGC
Cycas taitungensis                GGCCCCCTTGCCGCCTTCTCTAACAAGCCCTCGAGCCTCCTTTGCGCCGC
                                        **  *** ***   ****************** **** *** **

Arabidopsis thaliana              CTTCCTCAT------AGAAGCCGCCGGGTTGACCCCGAAG---GCTGAAT
Brassica napus                    CTTCCTCAT------AGAAGCCGCCGGGTTGACCCCGAAG---GCCGAAT
```

```
Lotus japonicus                   CTTCTTCAT------AGAAGCCGCCGGGTTGACCCCGAAG---GCCGAAT
Millettia pinnata                 CTTCTTCAT------AGAAGCCGCCGGGTTGACCCCGAAG---GCCGAAT
Citrullus lanatus                 CTTCCTCAT------AGAAGCCACCGGGTTGACCCCTCAG---GCCGAAT
Cucurbita pepo                    CTTCCTCAT------AGAAGCCACCGGGTTGACCCCTCAG---GACGAAT
RRIC110_matR                      CTTCCTCAT------GGAATCCGCCGGTTTGACCCCGAAG---GCCGAAT
Beta macrocarpa                   GTTCCTCAT------AGAAGCCGCCGGGTTGACCCCGAAG---GCCGAAT
Beta vulgaris maritima            GTTCCTCAT------AGAAGCCGCCGGGTTGACCCCGAAG---GCCGAAT
Beta vulgaris vulgaris            GTTCCTCAT------AGAAGCCGCCGGGTTGACCCCGAAG---GCCGAAT
Silene latifolia                  CTTACTCAT------AGAAGCCCCCGGGTTGACCCCGAAG---GCCGAAT
BPM24_matR                        CTTCCTCAT------GGAATCCGCCGGTTTGACCCCGAAG---GCCGAAT
PB235_matR                        CTTCCTCAT------GGAATCCGCCGGTTTGACCCCGAAG---GCCGAAT
Zea mays mays                     CTTCCTCAT------AGAAGCCGCTGGGTTGACCCCGAAG---GCCGAAT
RRIT251_matR                      CTTCCTCAT------GGAATCCGCCGGTTTGACCCCGAAG---GCCGAAT
RRII105_matR                      CTTCCTCAT------GGAATCCGCCGGTTTGACCCCGAAG---GCCGAAT
RRIM600_matR                      CTTCCTCAT------GGAATCCGCCGGTTTGACCCCGAAG---GCCGAAT
Cycas taitungensis                CTTCCTCATTCGTAAAGAAGCCGCCGGGTTGACCCCGGAACGAAGAAAGG
                                   **  ****       *** ** * ** ********  *        *

Arabidopsis thaliana              TCTATGGTGGAGAACGCTG------TAATAATAATTGGG-----------
Brassica napus                    TCTATGGTGGAGAACGCTG------TAATAATAATTGGG-----------
Lotus japonicus                   TCTATGGTAGAGAATGCTGCT---TTAATAATAATTTGG-----------
Millettia pinnata                 TCTATGGTAGAGAAT---------TTAATAATAATTGGG-----------
Citrullus lanatus                 TCTATGGTCGAGAACGCTG------TAATAAGAAATTGG-----------
Cucurbita pepo                    TCTATGGTCGAGAACGCTG------TAATAAGAAATTGG-----------
RRIC110_matR                      TCTATGGTAGAGAACGCTG------TAATAATAATTGGG-----------
Beta macrocarpa                   TCTATGGTAGAGAATGCTG------TAATAATAATTGGG-----------
Beta vulgaris maritima            TCTATGGTAGAGAATGCTG------TAATAATAATTGGG-----------
Beta vulgaris vulgaris            TCTATGGTAGAGAATGCTG------TAATAATAATTGGG-----------
Silene latifolia                  TCTATGGTAGAGAATGCTT------TCATAATAATTGGG-----------
BPM24_matR                        TCTATGGTAGAGAACGCTG------TAATAATAATTGGG-----------
PB235_matR                        TCTATGGTAGAGAACGCTG------TAATAATAATTGGG-----------
Zea mays mays                     TCAATGGTAGAGAAGGCTT------TAATAAGAATTTGG-----------
RRIT251_matR                      TCTATGGTAGAGAACGCTG------TAATAATAATTGGG-----------
RRII105_matR                      TCTATGGTAGAGAACGCTG------TAATAATAATTGGG-----------
RRIM600_matR                      TCTATGGTAGAGAACGCTG------TAATAATAATTGGG-----------
Cycas taitungensis                CCGAAGGTAGAGAACGCCCTTTATCTAATAAGAAGAAGAACAGGGTAGCG
                                   * * *** *****           * **** **   *

Arabidopsis thaliana              ----CCATGAGAGACCTTCTTAAGTATTGCAAAAGAAAGGGCCTGCTGAT
Brassica napus                    ----CCATGAGAGACCTTCTTAAGTATTGCAAAAGAAAGGGCCTGCTGAT
Lotus japonicus                   ----CCATGAGAGACCTTATTAAGTATTGCAAAAGAAAGGGCCTGCTGAT
Millettia pinnata                 ----CCATGAGAGACCTTATTAAGTATTGCAAAAGAAAGGGCCTGCTGAT
Citrullus lanatus                 ----CCATGAGAGACCTTTTTAAGTATTGCAAAAGAAAAGGCCTGCTGAT
Cucurbita pepo                    ----CCATGAGAGAACCTGAGAAGTATTACAAAAGAAAAGGACTGATGAT
RRIC110_matR                      ----CCATGAGAGACCTTATTAAGTATTGCAAAAGAAAGGGCCTGCTGAT
Beta macrocarpa                   ----CCATGAGAGACCTTTTTAAGTATTGCAAAAGAAAGGGCCTGCTGAT
Beta vulgaris maritima            ----CCATGAGAGACCTTTTTAAGTATTGCAAAAGAAAGGGCCTGCTGAT
Beta vulgaris vulgaris            ----CCATGAGAGACCTTTTTAAGTATTGCAAAAGAAAGGGCCTGCTGAT
Silene latifolia                  ----CCATGAGAGACCTTTTTAAGTATTGCAAAAGAAAGGGCCTGCTGAT
BPM24_matR                        ----CCATGAGAGACCTTATTAAGTATTGCAAAAGAAAGGGCCTGCTGAT
PB235_matR                        ----CCATGAGAGACCTTATTAAGTATTGCAAAAGAAAGGGCCTGCTGAT
Zea mays mays                     ----CCATGAGAGACCTTCTTAAGTATTGCAAAAGAAGGGGCCCGCTGAT
RRIT251_matR                      ----CCATGAGAGACCTTATTAAGTATTGCAAAAGAAAGGGCCTGCTGAT
RRII105_matR                      ----CCATGAGAGACCTTATTAAGTATTGCAAAAGAAAGGGCCTGCTGAT
RRIM600_matR                      ----CCATGAGAGACCTTATTAAGTATTGCAAAAGAAAGGGCCTGCTGAT
Cycas taitungensis                TTCTCCATGAAAGACCCTATTAAGTATTGCAAGAGAAGGGGCCTGCTGAT
                                      ****** *** * *   ******* *** ****  ** * * ****

Arabidopsis thaliana              AGAGCTGGGCGGGGAGGCGATACTAGTTATCAGGTCAGAGAGAGGCCTGG
Brassica napus                    AGAGCTGGGCGGGGAGGCGATACTAGTTATCAGGTCAGAGAGAGGCCTGG
Lotus japonicus                   AGAGCTGGGCGGGGAGGCGAGACTGGTTATCAGGTCAGAGAGAGGCCTGG
Millettia pinnata                 AGAGCTGGGCGGGGAGGCGAGACTAGTTATCAGGTCAGAGAGAGGCCTGG
```

```
Citrullus lanatus                 AGAGCTGGGCGGGGAGGCGATACTAGTTATCAGGTCAGAGAGACGCCTGG
Cucurbita pepo                    AGAGCTGGGCGGGGAGGCGAGACTAGTTATCAGGTCAGAGAGACGCCTGG
RRIC110_matR                      AGAGCTGGGCGGGGAGGCGATACTAGTTATCAGGTCAGAGAGACGCCTGG
Beta macrocarpa                   AGAGCTGGGCGAGGCAGCGATACTAGTTATCAGGTCAGAGAAAGGCCTGG
Beta vulgaris maritima            AGAGCTGGGCGAGGCAGCGATACTAGTTATCAGGTCAGAGAAAGGCCTGG
Beta vulgaris vulgaris            AGAGCTGGGCGAGGCAGCGATACTAGTTATCAGGTCAGAGAAAGGCCTGG
Silene latifolia                  AGAGCTGGGCGAGGCAGGGATACTCGTTCTCAGGTCAGATAAAGGCCTGG
BPM24_matR                        AGAGCTGGGCGGGGAGGCGATACTAGTTATCAGGTCAGAGAGACGCCTGG
PB235_matR                        AGAGCTGGGCGGGGAGGCGATACTAGTTATCAGGTCAGAGAGACGCCTGG
Zea mays mays                     AGAGCTGGGCGGGGAGGCGATACTAGTTACCAGGTCAGAGAGAGGCCTGG
RRIT251_matR                      AGAGCTGGGCGGGGAGGCGATACTAGTTATCAGGTCAGAGAGACGCCTGG
RRII105_matR                      AGAGCTGGGCGGGGAGGCGATACTAGTTATCAGGTCAGAGAGACGCCTGG
RRIM600_matR                      AGAGCTGGGCGGGGAGGCGATACTAGTTATCAGGTCAGAGAGACGCCTGG
Cycas taitungensis                AGAGCTGGGCGGGGAGGCGATACCAGTTATCAGGTTAGAAAGACGCCTAG
                                  *********** **  * ** **  ***  ***** *** * * **** *

Arabidopsis thaliana              CCCGTAAGCAGG------CCCCCTT------AAAAACCCATTACTTA---
Brassica napus                    CCCGTAAGCAGG------CCCCCTT------AAAAACCCATTACTTA---
Lotus japonicus                   CCCGTAAACAGG------CCCCCTTCAAAATAAAAACCCATTATTTC---
Millettia pinnata                 CCCGTAAACTGG------CCCCCTTCAAAAAAAAAACCCATTTTTTC---
Citrullus lanatus                 CCCGTAAGCAGG------CCCCCTT------AAAAACCCATTACTTA---
Cucurbita pepo                    CCCCTAAGCTGG------CCCCCTT------AAAACCCCATTACTTA---
RRIC110_matR                      CCCGTAAGCTGG------CTCCCTT------AAAAAGCCATTACTTA---
Beta macrocarpa                   CCCGTAAGCTGG------CCCCCTT------TAAAACCCATTACTTA---
Beta vulgaris maritima            CCCGTAAGCTGG------CCCCCTT------TAAAACCCATTACTTA---
Beta vulgaris vulgaris            CCCGTAAGCTGG------CCCCCTT------TAAAACCCATTACTTA---
Silene latifolia                  CCCGTAAGCTGG------CCCCCTT------AAAAACCCATTACTTA---
BPM24_matR                        CCCGTAAGCTGG------CTCCCTT------AAAAAGCCATTACTTA---
PB235_matR                        CCCGTAAGCTGG------CTCCCTT------AAAAAGCCATTACTTA---
Zea mays mays                     CCCGTAAGCTGG------CCCCCTT------TAAAAGCCATTCCTTATTA
RRIT251_matR                      CCCGTAAGCTGG------CTCCCTT------AAAAAGCCATTACTTA---
RRII105_matR                      CCCGTAAGCTGG------CTCCCTT------AAAAAGCCATTACTTA---
RRIM600_matR                      CCCGTAAGCTGG------CTCCCTT------AAAAAGCCATTACTTA---
Cycas taitungensis                CCCGTAAGCAGGGGCTATCCCCCGTTCACTTAAATGACCTATACTTA---
                                  *** *** * **      * *** *       **   **  *  **

Arabidopsis thaliana              ATAAGGATTTGTTACGCGCGATATGCCGACGACTTACTACTGGGAATCGT
Brassica napus                    ATAAGGATTTGTTACGCGCGATATGCCGACGACTTACTACTGGGAATCGT
Lotus japonicus                   ATAAGGATTTGTTACGTGCGATATGCCGACGACTTACTACTGGGAATCGT
Millettia pinnata                 ATAAGGATTTGTTACGTGCGATATGCCGACGACTTACTACTGGGAATCGT
Citrullus lanatus                 ATAAGGATTTGTTACGCGCGATATGCCGACGACTTACTACTGGGAATCGT
Cucurbita pepo                    ATAAGGATTTCTTACGCGCGATATGCCGACGACTTACTACTGGGAATCGT
RRIC110_matR                      ATAAAGATTTCTTACGCGCGATATGCCGACGACTTACTACTGGGAATCGT
Beta macrocarpa                   ATAAGGATTTGTTACGCGCGATATGCCGACGACTTACTATTGGGAATCGT
Beta vulgaris maritima            ATAAGGATTTGTTACGCGCGATATGCCGACGACTTACTATTGGGAATCGT
Beta vulgaris vulgaris            ATAAGGATTTGTTACGCGCGATATGCCGACGACTTACTATTGGGAATCGT
Silene latifolia                  ATAAGGATTTGTTACGCGCGATATGCCGACGACTTACTATTTGGAATCGT
BPM24_matR                        ATAAAGATTTCTTACGCGCGATATGCCGACGACTTACTACTGGGAATCGT
PB235_matR                        ATAAAGATTTCTTACGCGCGATATGCCGACGACTTACTACTGGGAATCGT
Zea mays mays                     ATAAGGATTTGTTACGCGCGATATGCCGACGACTTACTACTGGGAATCGT
RRIT251_matR                      ATAAAGATTTCTTACGCGCGATATGCCGACGACTTACTACTGGGAATCGT
RRII105_matR                      ATAAAGATTTCTTACGCGCGATATGCCGACGACTTACTACTGGGAATCGT
RRIM600_matR                      ATAAAGATTTCTTACGCGCGATATGCCGACGACTTACTACTGGGAATCGT
Cycas taitungensis                ATCAGGATTTGTTACGCGCGATATGCCGACGACTTACCACTGGGAATCGT
                                  ** * ***** ***** ******************** * * ********

Arabidopsis thaliana              GGGTGCCGTAGAGCTTCTCATAGAAATACAAAAACGTATCGCCCATTTCC
Brassica napus                    GGGTGCCGTAGAGCTTCTCATAGAAATACAAAAACGTATCGCCCATTTCC
Lotus japonicus                   GGGTGCCGTAGAGCTTCTCATAGAAATACAAAAACGTATCGCCCACTTCC
Millettia pinnata                 GGGTGCCGTAGAGCTTCTCATAGAAATACAAAAACGTATCGCCCACTTCC
Citrullus lanatus                 GGGTGCCGTAGAGCTTCTCATAGAAATACAAAAACGTATCGCCCACTTCC
Cucurbita pepo                    GGGTGCCGTAGAGCTTCTCATAGAAATACAAAAACGTATCGCCCACTTCC
```

```
RRIC110_matR                      AGGTGCCGTAGAGCTTCTCATAGAAATACAAAAACGTATCGCCCAATTCC
Beta macrocarpa                   GGGTGCCGTAGAGCTTCTCATAGAAATACAAAAACGTATCGCCCACTTCC
Beta vulgaris maritima            GGGTGCCGTAGAGCTTCTCATAGAAATACAAAAACGTATCGCCCACTTCC
Beta vulgaris vulgaris            GGGTGCCGTAGAGCTTCTCATAGAAATACAAAAACGTATCGCCCACTTCC
Silene latifolia                  GGGTGCCGTAGAGCTTCTCATAGAAATACAAAAACGTATCGCCCACTTCC
BPM24_matR                        AGGTGCCGTAGAGCTTCTCATAGAAATACAAAAACGTATCGCCCAATTCC
PB235_matR                        AGGTGCCGTAGAGCTTCTCATAGAAATACAAAAACGTATCGCCCAATTCC
Zea mays mays                     GGGTGCCGTATTTCTTCTCATAGAAATACAAAAACGTATCACCCACTTCC
RRIT251_matR                      AGGTGCCGTAGAGCTTCTCATAGAAATACAAAAACGTATCGCCCAATTCC
RRII105_matR                      AGGTGCCGTAGAGCTTCTCATAGAAATACAAAAACGTATCGCCCAATTCC
RRIM600_matR                      AGGTGCCGTAGAGCTTCTCATAGAAATACAAAAACGTATCGCCCAATTCC
Cycas taitungensis                GGGTACCGTAGAGCTTATAAGAGGAATCCAAAAACGTATCACCCACTTCC
                                   *** *****   *** * * ** *** ************ **** ****

Arabidopsis thaliana              TACAATCTGGCCTGAACCTTTGGGTAGGCTCCGCAGGATCAACAACAATA
Brassica napus                    TACAATCCGGCCTGAACCTTTGGGTAGGCTCCGCAGGATCAACAACAATA
Lotus japonicus                   TACAATCCGGCCTGAACCTTTGGGTAGGCTCTGCAGGATCAACAACAATA
Millettia pinnata                 TACAATCCGGCCTGAACCTTTTGGTAGGCTCTGCAGGATCAACAACAATA
Citrullus lanatus                 TACAATCCGGCCTGAACCTTTGGGTAGGCTTTGCAGGAACAACAACAATA
Cucurbita pepo                    TACAATCCGGCCTGAACCTTTGGGTAGGCTTTGCAGGAACAACAACAATA
RRIC110_matR                      TACAATCCGGCCTGAACCTTTTGGTAGGCTCGGCAGGATCAACAACAATA
Beta macrocarpa                   TACAATCCGGCCTGAACCTTTGGGTAGGCTCTGCGGGATCAACAACAATA
Beta vulgaris maritima            TACAATCCGGCCTGAACCTTTGGGTAGGCTCTGCGGGATCAACAACAATA
Beta vulgaris vulgaris            TACAATCCGGCCTGAACCTTTGGGTAGGCTCTGCGGGATCAACAACAATA
Silene latifolia                  TACAATCCGGCCTGAACCTTTGGGTAGGCTCTGCGGGATCAACAACAATA
BPM24_matR                        TACAATCCGGCCTGAACCTTTTGGTAGGCTCGGCAGGATCAACAACAATA
PB235_matR                        TACAATCCGGCCTGAACCTTTTGGTAGGCTCGGCAGGATCAACAACAATA
Zea mays mays                     TACAATCCGGCCTGAACCTTTGGGTAGGCTCCGCAGGATCAACAACCATA
RRIT251_matR                      TACAATCCGGCCTGAACCTTTTGGTAGGCTCGGCAGGATCAACAACAATA
RRII105_matR                      TACAATCCGGCCTGAACCTTTTGGTAGGCTCGGCAGGATCAACAACAATA
RRIM600_matR                      TACAATCCGGCCTGAACCTTTTGGTAGGCTCGGCAGGATCAACAACAATA
Cycas taitungensis                TACAATCCGGCCCGAACCTTGAGGTAGGCTCTGCAAGATCAACTTACATA
                                  ******* **** *******  ********  **  ** ****    ***

Arabidopsis thaliana              GCTGCACGGAGTACGGTAGAATTCCTTGGTACGGTCATTCGGGAAGTCCC
Brassica napus                    GCTGCACGGAGTACGGTAGAATTCCTCGGTACGGTCATTCGGGAAGTCCC
Lotus japonicus                   GCTGCACGGAGTACGGTAGAATTCCUCGGTACGGTCATTCGGGAAGTCCC
Millettia pinnata                 GCTGCACGGAGTACGGTAGAATTCCUCGGTACGGTCATTCGGGAAGTCCC
Citrullus lanatus                 GCTGCACGGAGTACGGTAGAATTCCUTGGTACGGTCATTCGGGAAGTCCC
Cucurbita pepo                    GCTGCACGGAGTACGGTAGAATTCCUTGGTACCGTCATTCGGGAAGTCCC
RRIC110_matR                      GCTGCACGGAGTACGGTAGAATTCCTCGGTACGGTCATTCGGGAAGTTCC
Beta macrocarpa                   GCTGCACGGAGTACGGTAGAATTCCTCGGTACGGTCATTCGGGAAGTCCC
Beta vulgaris maritima            GCTGCACGGAGTACGGTAGAATTCCTCGGTACGGTCATTCGGGAAGTCCC
Beta vulgaris vulgaris            GCTGCACGGAGTACGGTAGAATTCCTCGGTACGGTCATTCGGGAAGTCCC
Silene latifolia                  GCTGCACGGAGTACGGTAGAATTCCTCGGTACGGTCATTCGGGAAGTCCC
BPM24_matR                        GCTGCACGGAGTACGGTAGAATTCCTCGGTACGGTCATTCGGGAAGTTCC
PB235_matR                        GCTGCACGGAGTACGGTAGAATTCCTCGGTACGGTCATTCGGGAAGTTCC
Zea mays mays                     GCTGCACGGAGTACGGTAGAATTCCUUGGTACGGTCATTCGGGAAGTCCC
RRIT251_matR                      GCTGCACGGAGTACGGTAGAATTCCTCGGTACGGTCATTCGGGAAGTTCC
RRII105_matR                      GCTGCACGGAGTACGGTAGAATTCCTCGGTACGGTCATTCGGGAAGTTCC
RRIM600_matR                      GCTGCACGGAGTACGGTAGAATTCCTCGGTACGGTCATTCGGGAAGTTCC
Cycas taitungensis                GCTGCACGGAGTACGGTAGAATCCCCCGGTACGGTCATTCGGGAAGTCCC
                                  ********************** **  ***** ************** **

Arabidopsis thaliana              TCCGAGGACGACTCCCATACAATTTTTGCGAGAGCTGGAAAAGCGTCTAC
Brassica napus                    TCCGAGGACGACTCCCATACAATTTTTGCGAGAGCTGGAAAAGCGTCTAC
Lotus japonicus                   TCCGAGGACGACTCCCATACAATTCTTGCGAGAGCTGGAGAAGCGTCTAC
Millettia pinnata                 TCCGAGGACGACTCCCATACAATTCTTGCGAGAGCTGGAGAAGCGTCTAC
Citrullus lanatus                 TCCGAGGACGACTCCCATACAATTCTTGCGAGAGCTGGAGAAGCGTCTAC
Cucurbita pepo                    TCCGAGGACGACTCCCATACAATTCTTGCGAGAGCTCGAGAAGCGTCTAC
RRIC110_matR                      TCCGAGGACGACCCCCATACAATTCTTTCGAGAGCTGGAGAAGCGTCTAC
Beta macrocarpa                   TCCGAAGACGACTCCCATACAATTTTTGCGAGAGCTGGAGAAGCGTCTAC
```

```
Beta vulgaris maritima            TCCGAAGACGACTCCCATACAATTTTTGCGAGAGCTGGAGAAGCGTCTAC
Beta vulgaris vulgaris            TCCGAAGACGACTCCCATACAATTTTTGCGAGAGCTGGAGAAGCGTCTAC
Silene latifolia                  TCCGAAGACGACTCCCATACAATTCTTGCGAGAGCTGGAGAAGCGTCTAC
BPM24_matR                        TCCGAGGACGACCCCCATACAATTCTTTCGAGAGCTGGAGAAGCGTCTAC
PB235_matR                        TCCGAGGACGACCCCCATACAATTCTTTCGAGAGCTGGAGAAGCGTCTAC
Zea mays mays                     CCCGAGGACGACTCCCATACAATTCTTGCGAGAGCTGGAGAAGCGTCTAC
RRIT251_matR                      TCCGAGGACGACCCCCATACAATTCTTTCGAGAGCTGGAGAAGCGTCTAC
RRII105_matR                      TCCGAGGACGACCCCCATACAATTCTTTCGAGAGCTGGAGAAGCGTCTAC
RRIM600_matR                      TCCGAGGACGACCCCCATACAATTCTTTCGAGAGCTGGAGAAGCGTCTAC
Cycas taitungensis                TTCGGGGACGACTCCCATACAATTATTGCGAGAGCTGGAGAAGCGTCGGA
                                    **  ****** *********** ** ******** ** *******

Arabidopsis thaliana              GGGTAAAGCACCGTATCCATATAACTGCTTGCCACCTACGCTCCGCCATC
Brassica napus                    GGGTAAAGCACCGTATCCATATAACTGCTTGCCACCTACGCTCCGCCATC
Lotus japonicus                   GGGTAAAGCACCGTATCCATATAACTGTTTGCCACCTACGCTCCGCCATC
Millettia pinnata                 GGGTAAAGCACCGTATCCATATAACTGTTTGCCACCTACGCTCCGCCATC
Citrullus lanatus                 GGGTAAAGCACCGTATCCATATAACAGCTTGCCACCTACGCTCCGCCATC
Cucurbita pepo                    GGGTAAAGCACCGTATCCATATAACTGCTTGCCACCTACGCTACGCCATC
RRIC110_matR                      GGGTAAAGCACCGTATCCATATAACTGCTTGCCACCTACGCTCCGCCATC
Beta macrocarpa                   GGGTAAAGCACCGTATCCATATAACTGCTTGCCACTTACGCTCTGCCATT
Beta vulgaris maritima            GGGTAAAGCACCGTATCCATATAACTGCTTGCCACTTACGCTCTGCCATT
Beta vulgaris vulgaris            GGGTAAAGCACCGTATCCATATAACTGCTTGCCACTTACGCTCTGCCATT
Silene latifolia                  GGGTAAAGTACCGTATCCATATAACTGCTTGCCACTTACGCTCTGCCATT
BPM24_matR                        GGGTAAAGCACCGTATCCATATAACTGCTTGCCACCTACGCTCCGCCATC
PB235_matR                        GGGTAAAGCACCGTATCCATATAACTGCTTGCCACCTACGCTCCGCCATC
Zea mays mays                     GGGTAAAGCACCGTATCCATATAACTGCCTGCCACCTACGCTCCGCCATC
RRIT251_matR                      GGGTAAAGCACCGTATCCATATAACTGCTTGCCACCTACGCTCCGCCATC
RRII105_matR                      GGGTAAAGCACCGTATCCATATAACTGCTTGCCACCTACGCTCCGCCATC
RRIM600_matR                      GGGTAAAGCACCGTATCCATATAACTGCTTGCCACCTACGCTCCGCCATC
Cycas taitungensis                GAGTAAAGCACCGTATCCATCTCACAGCTCCCCACCTACGCTCCGCCATC
                                  * ****** *********** * ** *    **** ******  *****

Arabidopsis thaliana              CATTCAAAGTTTAGGAACCTAGGTGATAGTATCCCGATCAAACAGCTGAC
Brassica napus                    CATTCAAAGTTTAGGAATCTAGGTGATAGTATCCCGATCAAACAGCTGAC
Lotus japonicus                   CATTCCAAGTTTAGGAACCTAGGGAAGGGGATACCGATCAAACAGCTGAC
Millettia pinnata                 CATTCCAAGTTTAGGAACCTAGGGAATAGTATCCCGATCAAACAGCTGAC
Citrullus lanatus                 CATTCAAACTTTAGGAACCTAGGTAATAGTATCCCGATCAAAGAGCTGAC
Cucurbita pepo                    CATTCAAACTTGACGAACCTAGGTAATAATATCCCGATAAAAGAGCTGAC
RRIC110_matR                      CATTCCAAGTTTAGGAACCTAGGTAATAGTATCCCGATCAAACAGCTGAC
Beta macrocarpa                   CATTCCAAGTTTAGGAACCTAGGTAATAGTATCCCGATCAAACAGCTGAC
Beta vulgaris maritima            CATTCCAAGTTTAGGAACCTAGGTAATAGTATCCCGATCAAACAGCTGAC
Beta vulgaris vulgaris            CATTCCAAGTTTAGGAACCTAGGTAATAGTATCCCGATCAAACAGCTGAC
Silene latifolia                  CATTCCAAGTTTAGGAACCTAGGTAAGAGTATACCGATCAAACAGCTGAC
BPM24_matR                        CATTCCAAGTTTAGGAACCTAGGTAATAGTATCCCGATCAAACAGCTGAC
PB235_matR                        CATTCCAAGTTTAGGAACCTAGGTAATAGTATCCCGATCAAACAGCTGAC
Zea mays mays                     CATTCCAAGTTAAGGGACCTAGGTTATAGTATCCCTATCAAAGAGCTGAC
RRIT251_matR                      CATTCCAAGTTTAGGAACCTAGGTAATAGTATCCCGATCAAACAGCTGAC
RRII105_matR                      CATTCCAAGTTTAGGAACCTAGGTAATAGTATCCCGATCAAACAGCTGAC
RRIM600_matR                      CATTCCAAGTTTAGGAACCTAGGTAATAGTATCCCGATCAAACAGCTGAC
Cycas taitungensis                CATTCCGAGTTAGAGGACCTAGGGAGGAGTATCCTGATCCAACAGCTGAC
                                  *****  * **   * * *****       ** *  **  ** *******

Arabidopsis thaliana              GAAGGGGATGAGCAAAACAGGGAGTCTACAGGACGGGGTTCAACTAGCGG
Brassica napus                    GAAGGGGATGAGCAAAACAGGGAGTCTACAGGACGGGGTTCAACTAGCGG
Lotus japonicus                   GAAGGGGATGAGCGAAAGAGGGAGTCTACTGGACGGGGTTCCACTAGCGG
Millettia pinnata                 GAAGGGGATGAGCGAAACAGGGAGTCTACTGGACGGGGTTCCACTAGCGG
Citrullus lanatus                 GAAGGGGATGAGCCAAACAGGGAGTCTACTGGACGGGGTTCAACTAGCGG
Cucurbita pepo                    GAAGGGGATAAGCCAAAGAGGGAGTCTACTGGACGGGGTTCAACTAGCAG
RRIC110_matR                      GAAGGAGATGAGCGAAACAGGGAGTCTACTGGACGGGGTTCAACTAGCGG
Beta macrocarpa                   GAAGGGGATGAGCAAAACAGGGAGTCTACTGGACGCGGTTCAACTAGCGG
Beta vulgaris maritima            GAAGGGGATGAGCAAAACAGGGAGTCTACTGGACGCGGTTCAACTAGCGG
Beta vulgaris vulgaris            GAAGGGGATGAGCAAAACAGGGAGTCTACTGGACGCGGTTCAACTAGCGG
```

```
Silene latifolia            GAAGGGGATGAGCAAAACAAGGAGTCTACTGGACGCGGTTCAACTAGCGG
BPM24_matR                  GAAGGAGATGAGCGAAACAGGGAGTCTACTGGACGGGGTTCAACTAGCGG
PB235_matR                  GAAGGAGATGAGCGAAACAGGGAGTCTACTGGACGGGGTTCAACTAGCGG
Zea mays mays               GAAGGGGATGAGCGGAAGAGGTCGTCTACTGGACGCGGTTCAACTAGCGG
RRIT251_matR                GAAGGAGATGAGCGAAACAGGGAGTCTACTGGACGGGGTTCAACTAGCGG
RRII105_matR                GAAGGAGATGAGCGAAACAGGGAGTCTACTGGACGGGGTTCAACTAGCGG
RRIM600_matR                GAAGGAGATGAGCGAAACAGGGAGTCTACTGGACGGGGTTCAACTAGCGG
Cycas taitungensis          GAGGGGGATGAGCGGAACAGGGAGTCTACAGGACGCGGTTCAACTGGCGG
                            ** ** *** ***  ** * *  ****** ***** ***** *** ** *

Arabidopsis thaliana        AGACTCTTGGAACAGCTGGAGTCAGAAGTCCCCAAGTTAGCG------TA
Brassica napus              AGACTCTTGGAACAGCTGGAGTCAGAAGTCCCCAAGTTAGCG------TA
Lotus japonicus             AGACTCTTGGAACAGCTGGAGTCAGAAGTCCCCAAGTAAGCG------TA
Millettia pinnata           AGACTCTTGGAACAGCTGGAGTAAGAAGTCCCCAAGTAAGCG------TA
Citrullus lanatus           AGACTCTTGGAACTGCTGGAGTAAGAAGTCCCCAAGTGAGCG------TA
Cucurbita pepo              AGACTCTTGGAACAGCTGGAGTAATCCGTCCCCAAGTGAGCG------TA
RRIC110_matR                AGACTCTTGGAACAACTGGAGTAAGAAGTCCCCAAGCGAGCG------TA
Beta macrocarpa             AAAGTCTTTCCACAGCTAGAGTAAGAAGTCCCCAAGTGAGCG------TA
Beta vulgaris maritima      AAAGTCTTTCCACAGCTAGAGTAAGAAGTCCCCAAGTGAGCG------TA
Beta vulgaris vulgaris      AAAGTCTTTCCACAGCTAGAGTAAGAAGTCCCCAAGTGAGCG------TA
Silene latifolia            AAACTCTTGGAACAGCTAGAGTAAGAAGCCCCCAAGTGAGCA------TA
BPM24_matR                  AGACTCTTGGAACAACTGGAGTAAGAAGTCCCCAAGCGAGCG------TA
PB235_matR                  AGACTCTTGGAACAACTGGAGTAAGAAGTCCCCAAGCGAGCG------TA
Zea mays mays               AGACTCTTGGAAAAG---------AAAGTCCCCAAGTTAGCG------TA
RRIT251_matR                AGACTCTTGGAACAACTGGAGTAAGAAGTCCCCAAGCGAGCG------TA
RRII105_matR                AGACTCTTGGAACAACTGGAGTAAGAAGTCCCCAAGCGAGCG------TA
RRIM600_matR                AGACTCTTGGAACAACTGGAGTAAGAAGTCCCCAAGCGAGCG------TA
Cycas taitungensis          AGACTCTTGGAACAGCTGGAGTAAGAAGTCCCCGAGTGAGCGCGAGCGTA
                            * * ****   *               * **** **  ***       **

Arabidopsis thaliana        TTATGGGGGACCGTCAAGCACATCCGGCAAGGA---TCAAGGGGGATCTC
Brassica napus              TTATGGGGGACCGTCAAGCACATCCGGCAAGGCGTTCCTCGGGGGATCTC
Lotus japonicus             TTATGGGGGACCGTCCAGCACATCTGGCAAGGA---TCAAGGGGGATCTC
Millettia pinnata           TTATGGGGGACCGTCCAGCACATCTGGCAAGGA---TCAAGGGGGATCTC
Citrullus lanatus           TTATGGGGGACCGTCAAGCACATCCGGCAAGGA---TCAAGGGGGATCTC
Cucurbita pepo              TTATGGGGGACCGTCAAGCACATCCGGCAAAGA---TCAAGGGGGATCTC
RRIC110_matR                TTATGGGGGACCGTAAAGCACATCCGGCAAGGA---TCAAGGGGGATCTC
Beta macrocarpa             TTATGGGAGACCGTCAAGCACATCCGGCAAGGA---TCAAGGGAGATCTC
Beta vulgaris maritima      TTATGGGAGACCGTCAAGCACATCCGGCAAGGA---TCAAGGGAGATCTC
Beta vulgaris vulgaris      TTATGGGAGACCGTCAAGCACATCCGGCAAGGA---TCAAGGGAGATCTC
Silene latifolia            TTATGGGAGACCGTCAAGCACATCCGGCAAGGA---TCAAGGGAGATCTC
BPM24_matR                  TTATGGGGGACCGTAAAGCACATCCGGCAAGGA---TCAAGGGGGATCTC
PB235_matR                  TTATGGGGGACCGTAAAGCACATCCGGCAAGGA---TCAAGGGGGATCTC
Zea mays mays               TTATGGGGGACCGTCAAGCACATCCGGCAAAGA---TCAAGGGGGATCTC
RRIT251_matR                TTATGGGGGACCGTAAAGCACATCCGGCAAGGA---TCAAGGGGGATCTC
RRII105_matR                TTATGGGGGACCGTAAAGCACATCCGGCAAGGA---TCAAGGGGGATCTC
RRIM600_matR                TTATGGGGGACCGTAAAGCACATCCGGCAAGGA---TCAAGGGGGATCTC
Cycas taitungensis          TTATGGGAGACCGTCAAGCACATCCGGCAAGGA---TCAAGGGAAATCTT
                            ******* ******  ******** ***** *     *  ***  ****

Arabidopsis thaliana        GTTCTTGCATAGCTCAGGTCGGAGCAACGCGTCATCGGACGTTCAACAGG
Brassica napus              GTTCTTGCATAGCTCAGGTCGGAGCAACGCGTCATCGGACGTTCAACAGG
Lotus japonicus             GTTGTTGCATAGCTCAGGTCGGAGCAACGCGCCATCGGACGTTCAAGAGG
Millettia pinnata           GTTGTTGCATAGCTCAGGTCGGAGCAAGGCGCCATCGGACGTTCAAGAGG
Citrullus lanatus           GTTGTTGCATAGCTCAGGTCGTAGCAACGCGCCATCGGACGTTCAACAGG
Cucurbita pepo              GTTGTTGCATAGCTCAGGTCGGAGCAAGGCCCCATCGGACGTTCAACAGG
RRIC110_matR                GTTGTTGCATAGCTCAGGTCGGAGCAAGGCGCCATCGGACGTTCAACAGG
Beta macrocarpa             GTTGTTGCATAGCTCAGGTCAGAGCAAGGTGCCATCGGACGTTCAACAGG
Beta vulgaris maritima      GTTGTTGCATAGCTCAGGTCAGAGCAAGGTGCCATCGGACGTTCAACAGG
Beta vulgaris vulgaris      GTTGTTGCATAGCTCAGGTCAGAGCAAGGTGCCATCGGACGTTCAACAGG
Silene latifolia            GTTGTTGCATAGCTCAGGTCAGAGCAAGGTGCCACCGGACGTTCAACAGG
BPM24_matR                  GTTGTTGCATAGCTCAGGTCGGAGCAAGGCGCCATCGGACGTTCAACAGG
```

```
PB235_matR                       GTTGTTGCATAGCTCAGGTCGGAGCAAGGCGCCATCGGACGTTCAACAGG
Zea mays mays                    GTTGTTGCATAGCTCAGGTCAGAGCAAGGTGCCATCAGGCGTTCAACAGG
RRIT251_matR                     GTTGTTGCATAGCTCAGGTCGGAGCAAGGCGCCATCGGACGTTCAACAGG
RRII105_matR                     GTTGTTGCATAGCTCAGGTCGGAGCAAGGCGCCATCGGACGTTCAACAGG
RRIM600_matR                     GTTGTTGCATAGCTCAGGTCGGAGCAAGGCGCCATCGGACGTTCAACAGG
Cycas taitungensis               GTTGTTGCATAGCCCAGGTCGGAGCAACGTGCCATCAGACGTTCAACAGG
                                 *** ********* ******  ***** *   ** * * ******* ***

Arabidopsis thaliana             TAGTCTCACGATCGGGCA------CTCATGCCCGTAAGTTGTC---ATTG
Brassica napus                   TAGTCTCACGATCGGGCA------CTCATGCCCGTAAGTTGTC---ATTG
Lotus japonicus                  CAGTCGCACGATCGGGCA------TGAGTGCCCGGAAGTTCTC---ATTG
Millettia pinnata                CAGTCGCACGA--------------AGTGTCCGGAAGTTGTC---ATTG
Citrullus lanatus                CAGTTTCACGATCGGGCA------TGAGTGCCCGGAAGTTGTC---ATTG
Cucurbita pepo                   CAGTTTCACGATCGGGCA------TGAGTGACGGGAAGTTGTC---ATTG
RRIC110_matR                     CAGTCTCACGATCGGGCA------TGAGTGTCCGGAAGTTGTC---ATTG
Beta macrocarpa                  CAGTCTTGCGATCGGGCA------TGAGTGTCCGGAAGTTGTC---ATTG
Beta vulgaris maritima           CAGTCTTGCGATCGGGCA------TGAGTGTCCGGAAGTTGTC---ATTG
Beta vulgaris vulgaris           CAGTCTTGCGATCGGGCA------TGAGTGTCCGGAAGTTGTC---ATTG
Silene latifolia                 TAGTCTCGCGATCGGGCA------TGAGTGCCCGGAAGTTGTCGGAAAAA
BPM24_matR                       CAGTCTCACGATCGGGCA------TGAGTGTCCGGAAGTTGTC---ATTG
PB235_matR                       CAGTCTCACGATCGGGCA------TGAGTGTCCGGAAGTTGTC---ATTG
Zea mays mays                    CAGTCTCACGATCGGGCA------TGAGTGTCCTGAAGAATAA---ATTG
RRIT251_matR                     CAGTCTCACGATCGGGCA------TGAGTGTCCGGAAGTTGTC---ATTG
RRII105_matR                     CAGTCTCACGATCGGGCA------TGAGTGTCCGGAAGTTGTC---ATTG
RRIM600_matR                     CAGTCTCACGATCGGGCA------TGAGTGTCCGGAAGTTGTC---ATTG
Cycas taitungensis               CAGTCTCACGATCGGGCAAATCGTTGAGCGTCTGGAAGTTGTC---ATTG
                                  ***    ***                  * *   ***        *

Arabidopsis thaliana             TATACTCCC------------CCGGGTCGGAAGGCGGCGGGGGAGGGAGG
Brassica napus                   TATACTCCC------------CCGGGTCGGAAGGCGGCGGGGGAGGGAGG
Lotus japonicus                  TATACTCCC------------GCGGGTCGGAAGGCGGCGGGGGAAGGAGG
Millettia pinnata                TATACTCCC------------GCGGGTCGGAAGGCGGCGGGGGAAGGAGG
Citrullus lanatus                TATACTCCC------------GCGGGTCGGAAGGCGGCGGGGGAAGGAGG
Cucurbita pepo                   TATACTCCT------------GCGGGTCGGAAGGCGGCGGGGGAAAGAGG
RRIC110_matR                     TATACTCCC------------GCGGGTCGGAAGGCGGCGGGGGAAGGAGG
Beta macrocarpa                  TATACTCTC------------GCGGGTCGGAAGGCGGCGGGGGAAGGAGG
Beta vulgaris maritima           TATACTCTC------------GCGGGTCGGAAGGCGGCGGGGGAAGGAGG
Beta vulgaris vulgaris           TATACTCTC------------GCGGGTCGGAAGGCGGCGGGGGAAGGAGG
Silene latifolia                 GAGACTCTC------------GCGGGTCGGAAGGCGGCGGGGGAAGGAGG
BPM24_matR                       TATACTCCC------------GCGGGTCGGAAGGCGGCGGGGGAAGGAGG
PB235_matR                       TATACTCCC------------GCGGGTCGGAAGGCGGCGGGGGAAGGAGG
Zea mays mays                    TATACTCCC------------TTTGGTCGGAAGGCGGCGGGGGAAGGAAG
RRIT251_matR                     TATACTCCC------------GCGGGTCGGAAGGCGGCGGGGGAAGGAGG
RRII105_matR                     TATACTCCC------------GCGGGTCGGAAGGCGGCGGGGGAAGGAGG
RRIM600_matR                     TATACTCCC------------GCGGGTCGGAAGGCGGCGGGGGAAGGAGG
Cycas taitungensis               CATACTCCCAGAGAAGCGCTGGGAGGTCGGAAGGCGGCGGGGGAAGGAGG
                                  * ****                 ********************  ** *

Arabidopsis thaliana             AGGACACTGGG------CGGGATCTATCAGCAGCGAATTCCCCATAAAGA
Brassica napus                   AGGACACTGGG------CGGGATCTATCAGCAGCGAATTCCCCGTAAAGA
Lotus japonicus                  AGGACACTGGG------CGGGATCTATCAGCAGCGAATTCCCCATACAGA
Millettia pinnata                AGGACACTGGG------CGGGATCTATCAGCAGCGAATTCCCCATACAGA
Citrullus lanatus                AGGACACTGGG------CGGGATCTATCAGCAGCGAATTCCCCATACATC
Cucurbita pepo                   AGGACACTGGG------CGGGATCTATCAGCAGCGAATTCCCCATACATC
RRIC110_matR                     AGGACACTGGG------CGGGATCTATCAGCAGCGAATTCCCUATACAGA
Beta macrocarpa                  GGGACACTGGT------CGAGATCTATCAGCAGCGAATTCCCCATACAGA
Beta vulgaris maritima           GGGACACTGGT------CGAGATCTATCAGCAGCGAATTCCCCATACAGA
Beta vulgaris vulgaris           GGGACACTGGT------CGAGATCTATCAGCAGCGAATTCCCCATACAGA
Silene latifolia                 GGGACACTGGG------CGAGATCTATCAGCAGCGAATTCCCCATACAGA
BPM24_matR                       AGGACACTGGG------CGGGATCTATCAGCAGCGAATTCCCUATACAGA
PB235_matR                       AGGACACTGGG------CGGGATCTATCAGCAGCGAATTCCCUATACAGA
Zea mays mays                    GGGACACTGGG------CGGGATCTTTCAGCAGCGAATTCCCCATACAGA
```

```
RRIT251_matR                      AGGACACTGGG------CGGGATCTATCAGCAGCGAATTCCCUATACAGA
RRII105_matR                      AGGACACTGGG------CGGGATCTATCAGCAGCGAATTCCCUATACAGA
RRIM600_matR                      AGGACACTGGG------CGGGATCTATCAGCAGCGAATTCCCNATACAGA
Cycas taitungensis                GGGACACTGGGAACAGACGGGATCTTTCAGCAGCAAATTACCCATACAGA
                                   *********       ** ***** ******** **** **  ** *

Arabidopsis thaliana              TAGAGGCACCTATAAAAAAGATACTCCGAAGGCTTCGGGATCGAGGTATC
Brassica napus                    TAGAGGCACCTATAAAAAAGATACTCCGAAGGCTTCGGGATCGAGGTATC
Lotus japonicus                   TAGAGGCGCCTATCAAAAAGATACTCCGAAGGCTTCGGGATCGAGGTATC
Millettia pinnata                 TAGAGGCGCCTATCAAAAAGATACTCCGAAGGCTTCGGGATCGAGGTATC
Citrullus lanatus                 TCGAGGCGCCTATCAAAAAGATACTCCGAAGGCTTCGGGATCGAGGTATC
Cucurbita pepo                    TCGAGGCGCCTATCAAAAAGATACTCCGAAGGCTTCGGGATCGAGGTATC
RRIC110_matR                      TAGAGGCGCCTATCAAAAAGATACTCCGAAGGCTTCGGGATCGAGGTATC
Beta macrocarpa                   TAGAAGCGCCTATCAAAAAGATACTCCGAAGGCTTCGAGATCGAGGTCTC
Beta vulgaris maritima            TAGAAGCGCCTATCAAAAAGATACTCCGAAGGCTTCGAGATCGAGGTCTC
Beta vulgaris vulgaris            TAGAAGCGCCTATCAAAAAGATACTCCGAAGGCTTCGAGATCGAGGTCTC
Silene latifolia                  TAGAAGCGCCTATCAAAAAGATACTCCGAAGGCTTCGAGATCGAGGTCTC
BPM24_matR                        TAGAGGCGCCTATCAAAAAGATACTCCGAAGGCTTCGGGATCGAGGTATC
PB235_matR                        TAGAGGCGCCTATCAAAAAGATACTCCGAAGGCTTCGGGATCGAGGTATC
Zea mays mays                     TAGAGGCGCCTATCAAAAAGATACTCCGAAGGCTTCGGGATCGAGGTCTC
RRIT251_matR                      TAGAGGCGCCTATCAAAAAGATACTCCGAAGGCTTCGGGATCGAGGTATC
RRII105_matR                      TAGAGGCGCCTATCAAAAAGATACTCCGAAGGCTTCGGGATCGAGGTATC
RRIM600_matR                      TAGAGGCGCCTATCAAAAAGATACTCCGAAGGCTTCGGGATCGAGGTATC
Cycas taitungensis                TAAAGGCGCCTATGAAAAAGATACTCCAAAGGCTTCGGGATCGAGGTATC
                                  *  * ** ***** ************* ********* ********* **

Arabidopsis thaliana              ATTAGCCGAAGAAGACCCTGGCCAATCCACGTGGCCTGTTTGACGAACGT
Brassica napus                    ATTAGCCGAAGAAGACCCTGGCCAATCCACGTGGCCTGCTTGACGAACGT
Lotus japonicus                   ATTAGCCGAAGAAGACCCTGGCCAATCCACGTGGCCTGCTTGACGAACGT
Millettia pinnata                 ATTAGCCGAAGAAGACCCTGGCCAATCCACGTGGCCTGCTTGACGAACGT
Citrullus lanatus                 ATTAGCCGAAGAAGACCCTGGCCAATCCACGTGGCCTGCTTGACGAACGT
Cucurbita pepo                    ATTAGCCGAAGAAGACCCTGGCCAATCCACGTGGCCTGCTTGACGAACGT
RRIC110_matR                      ATTAGCCGAAGAAGACCCTGGCCAATCCACGTGGCCTGCTTGACGAGCGT
Beta macrocarpa                   ATTAGCCGAAGAAGACCCTGGCCAATCCACGTGGCCTGCTTGACGAACGT
Beta vulgaris maritima            ATTAGCCGAAGAAGACCCTGGCCAATCCACGTGGCCTGCTTGACGAACGT
Beta vulgaris vulgaris            ATTAGCCGAAGAAGACCCTGGCCAATCCACGTGGCCTGCTTGACGAACGT
Silene latifolia                  ATTAGCCGAAGAAGACCCTGGCCAATCCACGTGGCCTGCTTGACGAACGT
BPM24_matR                        ATTAGCCGAAGAAGACCCTGGCCAATCCACGTGGCCTGCTTGACGAGCGT
PB235_matR                        ATTAGCCGAAGAAGACCCTGGCCAATCCACGTGGCCTGCTTGACGAGCGT
Zea mays mays                     ATTAGCCGAAGAAGACCCAGGCCAATCCACGCGGCCTCTTTGACCAACGT
RRIT251_matR                      ATTAGCCGAAGAAGACCCTGGCCAATCCACGTGGCCTGCTTGACGAGCGT
RRII105_matR                      ATTAGCCGAAGAAGACCCTGGCCAATCCACGTGGCCTGCTTGACGAGCGT
RRIM600_matR                      ATTAGCCGAAGAAGACCCTGGCCAATCCACGTGGCCTGCTTGACGAGCGT
Cycas taitungensis                ATTAGCCGGAGAAGACCCTGGCCAACCCACGTGGCCTGCTTGACGAACGT
                                  ******** ********* ****** ***** *****  ***** * ***

Arabidopsis thaliana              CAGCGACGAAGACATCGTAAATTGGTUCGCGGGCATCGCGATAAGTCUTC
Brassica napus                    CAGCGACGAAGACATCGTAAATTGGTUCGCGGGCATCGCGATAAGTCUTC
Lotus japonicus                   CAGCGACGGAGACATCGTAAATTGGTUTGCGGGCATCGCGATAAGTCUTC
Millettia pinnata                 CAGCGACGGAGACATCGTAAATTGGTUTGCGGGCATCGCGATAAGTCUTC
Citrullus lanatus                 CAGCGACGGAGACATCGTAAATTGGTUCGCGGGCATCGCAATAAGTCTTC
Cucurbita pepo                    CAGCGACGGAGACATCGTAAATTGGTUCGCGGGCATCGCAATCAGTCTTC
RRIC110_matR                      CAGCGACGGAGACATCGTAAATTGGTCCGCGGGCATCGCGATAAGTCCTC
Beta macrocarpa                   CAGCGACGGAGACATCGTAAATTGGTUCGCGGGCATCGCGATAAGTCUTC
Beta vulgaris maritima            CAGCGACGGAGACATCGTAAATTGGTUCGCGGGCATCGCGATAAGTCUTC
Beta vulgaris vulgaris            CAGCGACGGAGACATCGTAAATTGGTUCGCGGGCATCGCGATAAGTCUTC
Silene latifolia                  CAGCGACGGAGACATCGTAAATTGGTTCGCGGGCATCGCGATAAGTCUTC
BPM24_matR                        CAGCGACGGAGACATCGTAAATTGGTCCGCGGGCATCGCGATAAGTCCTC
PB235_matR                        CAGCGACGGAGACATCGTAAATTGGTCCGCGGGCATCGCGATAAGTCCTC
Zea mays mays                     CAGCGACAGAGACATAGTAAATTGGTUCGCGGGCATCGCGATAAGTCUTC
RRIT251_matR                      CAGCGACGGAGACATCGTAAATTGGTCCGCGGGCATCGCGATAAGTCCTC
RRII105_matR                      CAGCGACGGAGACATCGTAAATTGGTCCGCGGGCATCGCGATAAGTCCTC
```

```
RRIM600_matR                      CAGCGACGGAGACATCGTAAATTGGTCCGCGGGCATCGCGATAAGTCCTC
Cycas taitungensis                CAGCGACGAAGACATCGTAAATCGGTTCGCGGGCGTCGCGATAAGTCCTC
                                  *******  ****** ****** ***  ****** **** ** **** **

Arabidopsis thaliana              TGTCCTACTACAGGTGCUGCGACAACCTTTATCAAGTCCGAACGATTGTC
Brassica napus                    TGTCCTACTACAGGTGCUGCGACAACCTTTACCAAGTCCGAACGATTGTC
Lotus japonicus                   TGTCCTACTACAGGTGCTGCGACAACCTTTAUCAAGTCCGAACGATTGTC
Millettia pinnata                 TGTCCTACTACAGGTGCUGCGACAACCTTTAUCAAGTCCGAACGATTGTC
Citrullus lanatus                 TGTCCTACTACAGGTGCTGCGACAACCTTTAUCAAGTCCGAACGATTGTC
Cucurbita pepo                    TGTCCTACTACAGGTGCTGCGACAACCTTTAUCAAGTACGAACGATTGTC
RRIC110_matR                      TGTCCTACTACAGGTGCCGCGACAACCTTTACCAAGTCCGAACGATTGTC
Beta macrocarpa                   TGTCCTACTACAGGTGCUGCGACAACCTTTAUCAAGTCCGAACGATTGTC
Beta vulgaris maritima            TGTCCTACTACAGGTGCUGCGACAACCTTTAUCAAGTCCGAACGATTGTC
Beta vulgaris vulgaris            TGTCCTACTACAGGTGCUGCGACAACCTTTAUCAAGTCCGAACGATTGTC
Silene latifolia                  TGTCCTACTACAGGTGCUGCGACAACCTTTAUCAAGTCCGAACGATTGTC
BPM24_matR                        TGTCCTACTACAGGTGCCGCGACAACCTTTACCAAGTCCGAACGATTGTC
PB235_matR                        TGTCCTACTACAGGTGCCGCGACAACCTTTACCAAGTCCGAACGATTGTC
Zea mays mays                     TGTCCTACTACAGGTGCTGCGACAACCTTTAUCAAGTCCGAACGATTGTC
RRIT251_matR                      TGTCCTACTACAGGTGCCGCGACAACCTTTACCAAGTCCGAACGATTGTC
RRII105_matR                      TGTCCTACTACAGGTGCCGCGACAACCTTTACCAAGTCCGAACGATTGTC
RRIM600_matR                      TGTCCTACTACAGGTGCCGCGACAACCTTTACCAAGTCCGAACGATTGTC
Cycas taitungensis                TGTCCCACCATAGGTGCTGCGACAACCTCTATCAAGTCCGAACGATTGTC
                                  ***** ** * ****** ********** ** ***** ************

Arabidopsis thaliana              GACUACCAGATTCGCTGGTCTGCAATATTCACCCTAGCCCACAAGCACAA
Brassica napus                    GACUACCAGATTCGCTGGTCTGCAATATTCACCCTAGCCCACAAGCACAA
Lotus japonicus                   GACUACCAGATCCGCTGGTCTGCAATATTCACCCTAGCCCACAAGCACAA
Millettia pinnata                 GACUACCAGATCCGCTGGTCTGCAATATTCACCCTAGCCCACAAGCACAA
Citrullus lanatus                 GACCACCAGATCCGCTGGTCTGCAATATTCACCCTAGCCCACAAGCACAA
Cucurbita pepo                    GACCACCAGATCCGCTGGTCCGCTATATTCACCCTAGCCCACAAGCACAA
RRIC110_matR                      GACUACCAGATCCGCTGGTCTGCAATATTCACCCTAGCCCACAAGCACAA
Beta macrocarpa                   GACTACCAGATCCGCTGGTCTGCAATATTCACCCUAGCCCACAAGCACAA
Beta vulgaris maritima            GACTACCAGATCCGCTGGTCTGCAATATTCACCCUAGCCCACAAGCACAA
Beta vulgaris vulgaris            GACTACCAGATCCGCTGGTCTGCAATATTCACCCUAGCCCACAAGCACAA
Silene latifolia                  GACTACCAGATCCGCTGGTCTGCAATCTTCACCCTAGCCCACAAGCACAA
BPM24_matR                        GACCACCAGATCCGCTGGTCTGCAATATTCACCCTAGCCCACAAGCACAA
PB235_matR                        GACUACCAGATCCGCTGGTCTGCAATATTCACCCTAGCCCACAAGCACAA
Zea mays mays                     AACTACCAGATCCGCTGGTCCGCTATATTCACCCTAGCCCACAAGCACAA
RRIT251_matR                      GACUACCAGATCCGCTGGTCTGCAATATTCACCCTAGCCCACAAGCACAA
RRII105_matR                      GACUACCAGATCCGCTGGTCTGCAATATTCACCCTAGCCCACAAGCACAA
RRIM600_matR                      GACUACCAGATCCGCTGGTCTGCAATATTCACCCTAGCCCACAAGCACAA
Cycas taitungensis                GACTACCAGATCCGCTGGTCCGCTGTATTCACCCCTGCCCACAAGCACAA
                                   ** ******* ******** **  * *******  **************

Arabidopsis thaliana              ATCCTCGGCGCCGAATATAATCCTCAAGTACTCCAAAGACTUAAATATTG
Brassica napus                    ATCCTCGGCGCGGAATATAATCCUCAAGTACTCCAAAGACTUAAATATTG
Lotus japonicus                   ATCCTCGGCGCGGAATATAATCCUCAAGTACTCCAAAGACTUAAATATAG
Millettia pinnata                 ATCCTCGGCGCGGAATATAATCCUCAAGTACTCCAAAGACTUAAATATAG
Citrullus lanatus                 ATCCTCGGCGCGGAATATAATCCCAAAGTACTCCAAAGACCCAAATATAG
Cucurbita pepo                    ATCCTCGGCGCAGAATATAATCCUAAAGTACTCCAAAGACCCAAATATAG
RRIC110_matR                      ATCCTCGGCGCGGAATATAATCCUAAAGTACTCCAAAGACTUAAATATAG
Beta macrocarpa                   ATCCTCAGCGCGGAATATAATCCAAAAGTACTCCAAAGACTTAAATATAG
Beta vulgaris maritima            ATCCTCAGCGCGGAATATAATCCAAAAGTACTCCAAAGACTTAAATATAG
Beta vulgaris vulgaris            ATCCTCAGCGCGGAATATAATCCAAAAGTACTCCAAAGACTTAAATATAG
Silene latifolia                  ATCCTCAGCGCGGAATATAATCCUAAAGTACTCCAAAGACTTAAATATAG
BPM24_matR                        ATCCTCGGCGCGGAATATAATCCUAAAGTACTCCAAAGACTUAAATATAG
PB235_matR                        ATCCTCGGCGCGGAATATAATCCUAAAGTACTCCAAAGACTUAAATATAG
Zea mays mays                     ATCTTCGGCGCGGAATATAATCCUAAAGCACUCCAAAGACTUAAATATAG
RRIT251_matR                      ATCCTCGGCGCGGAATATAATCCUAAAGTACTCCAAAGACTUAAATATAG
RRII105_matR                      ATCCTCGGCGCGGAATATAATCCUAAAGTACTCCAAAGACTUAAATATAG
RRIM600_matR                      ATCCTCGGCGCGGAATATAATCCUAAAGTACTCCAAAGACTNAAATATAG
Cycas taitungensis                ATCCTCGGCGCGGAAGATAATCCCAAAGTACTCCAAAGACTCACATATAG
```

```
                                   *** ** **** *** *******  *** ** ********  * **** *

Arabidopsis thaliana               TAAATCAAGAAGGTGGCAAGATCCTTGCAGAGTTCCCCAACAGCATAGAG
Brassica napus                     TAAATCAAGAAGGTGGTAAGATCCTTGCAGAGTTCCCCAACAGCATAGAG
Lotus japonicus                    TAAATCAAGAAGGTGGTAAGACCCTTGCAGAGTTCCCCAACAGCATAGAG
Millettia pinnata                  TAAATCAAGAAGGTGGTAAGACCCTTGCAGAGTTCCCCAACAGCATAGAG
Citrullus lanatus                  TCAATCAAGAAGGTGGTCAGACTGAAGCGGAGTTCCCCAACAGCATAGAG
Cucurbita pepo                     TCAATCAAGAAGGGGGTCAGACTGAAGCGGAGTTCCCCAACAGCATAGAG
RRIC110_matR                       TAAATAAAGAAGGTGGTAAGACCCTTGCAGAGTTCCCCAACAGCATAGAG
Beta macrocarpa                    TAAATCAAGAAGGTGGCAAGACCCTTGCGGAGTTCCCCAACAGCATAGAG
Beta vulgaris maritima             TAAATCAAGAAGGTGGCAAGACCCTTGCGGAGTTCCCCAACAGCATAGAG
Beta vulgaris vulgaris             TAAATCAAGAAGGTGGCAAGACCCTTGCGGAGTTCCCCAACAGCATAGAG
Silene latifolia                   TAAATAAAGAAGGTGGCAAGACCCTTGCAGAGTTCCCCAACAGCAGAGAG
BPM24_matR                         TAAATAAAGAAGGTGGTAAGACCCTTGCAGAGTTCCCCAACAGCATAGAG
PB235_matR                         TAAATAAAGAAGGTGGTAAGACCCTTGCAGAGTTCCCCAACAGCATAGAG
Zea mays mays                      TUAATCAAGAAGGTGGTAAGACCCTTGCAGAGTTCCCAAACAGCATAGAG
RRIT251_matR                       TAAATAAAGAAGGTGGTAAGACCCTTGCAGAGTTCCCCAACAGCATAGAG
RRII105_matR                       TAAATAAAGAAGGTGGTAAGACCCTTGCAGAGTTCCCCAACAGCATAGAG
RRIM600_matR                       TAAATAAAGAAGGTGGTAAGACCCTTGCAGAGTTCCCCAACAGCATAGAG
Cycas taitungensis                 TCAATAAAGAAGGTGGTAAGACCCCTGCCGAGTTCCCCAACAGCATAGAG
                                   * *** ******* **  ***     ** ******** ******* ****

Arabidopsis thaliana               CTTGGGAAGCTCGGACCCGGTCAAGACCTGAACAAGAA-GGAACACTCAA
Brassica napus                     CTTGGGAAGCTCGGACCCGGTCAAGACCTGAACAAGAA-GGAACACTCAA
Lotus japonicus                    CTTGGGAAGCTCGGACCCGGTCAAGATCCGAACAACAA-GGAGCACTCCA
Millettia pinnata                  CTTGGGAAGCTCGGACCCGGTCAAGATCCGAACAACAA-GGAGCACTCCA
Citrullus lanatus                  CTTGGGAAGCTCGGACCCGGTCAAGATCCGAACCACTA-CTAG-ACTAGA
Cucurbita pepo                     CTTGGGAAGCTCGGACCCGGTCAAGATCCGAACCACTA-CTAG-ACTAGA
RRIC110_matR                       CTTGGGAAGCTCGGACCCGGGCAAGATCCGAACAACAA-GGAGCACTCAA
Beta macrocarpa                    CTTGGGAAGCTCGGACCCGGTCAAGATCCGAACAACAA-AGAGCACTCAA
Beta vulgaris maritima             CTTGGGAAGCTCGGACCCGGTCAAGATCCGAACAACAA-AGAGCACTCAA
Beta vulgaris vulgaris             CTTGGGAAGCTCGGACCCGGTCAAGATCCGAACAACAA-AGAGCACTCAA
Silene latifolia                   CTTGGAAAGCTCGGACCCGGGCAAGATCCGAACAACAA-AGAGCACTCAA
BPM24_matR                         CTTGGGAAGCTCGGACCCGGGCAAGATCCGAACAACAA-GGAGCACTCAA
PB235_matR                         CTTGGGAAGCTCGGACCCGGGCAAGATCCGAACAACAA-GGAGCACTCAA
Zea mays mays                      CTTGGGAAGCTCGGACTCGGTCAAGATCCGAACAACGGCGGAGCACTCAA
RRIT251_matR                       CTTGGGAAGCTCGGACCCGGGCAAGATCCGAACAACAA-GGAGCACTCAA
RRII105_matR                       CTTGGGAAGCTCGGACCCGGGCAAGATCCGAACAACAA-GGAGCACTCAA
RRIM600_matR                       CTTGGGAAGCTCGGACCCGGGCAAGATCCGAACAACAA-GGAGCACTCAA
Cycas taitungensis                 CTTAGGAAGCTCGGACCAGAGTCATTCCTTACAAGTCAAGATCTGAACAA
                                   *** * **********  *    *   *  *                  *

Arabidopsis thaliana               CTACTAGTCTAGTCTAGTAGTCGTTTTTTTCTATTAGTTGCGATGCGAAC
Brassica napus                     CTACTAGTCTAGTCTAGTAGTCGTTTTTTTCTATTAGTTGCGATGCGAAC
Lotus japonicus                    CTACTAGTCTAGTC-----GTT-TTTTT--CTATTAGTTGCGATGCGAAC
Millettia pinnata                  CTACTAGTCTAGTC-----GTT-TTTTTTTCTATTAGTTGCGATGCGAAC
Citrullus lanatus                  CTAGTCGTTGAGTGACGTAGTGGTTTTTT-CTATTAGTTGCGATGCGAAC
Cucurbita pepo                     C-----GTTGAGTGAGGTAGTGGTTTTTT-CTATT---------------
RRIC110_matR                       CTACTAGTCTAGTCTAG---------------------------------
Beta macrocarpa                    CTACTAGTCTAGTCTAGTAGTT-TTTTTTTCTAAAAGTTGCGATGTGAAC
Beta vulgaris maritima             CTACTAGTCTAGTCTAGTAGTT-TTTTTTTCTAAAAGTTGCGATGTGAAC
Beta vulgaris vulgaris             CTACTAGTCTAGTCTAGTAGTT-TTTTTTTCTAAAAGTTGCGATGTGAAC
Silene latifolia                   CTACTAGTCTAGTCTAGTAGTTGTTTTTTTCTATTAGTTGCGATGTGAAC
BPM24_matR                         CTACTAGTCTAGTCTAG---------------------------------
PB235_matR                         CTACTAGTCTAGTCTAG---------------------------------
Zea mays mays                      CTACATGTTTAATAA-GTAGTTGTCTTTTTCTATTTTGATTTTAGCGAAC
RRIT251_matR                       CTACTAGTCTAGTC---TAG------------------------------
RRII105_matR                       CTACTAGTCTAGTCTAG---------------------------------
RRIM600_matR                       CTACTAGTCTAGTCTAG---------------------------------
Cycas taitungensis                 CAAGGAGCACGCGTACAAGACCTTATATUAGGTGTACATGCGATATGTAC
                                   *     *
```

```
Arabidopsis thaliana            AGGCGTTTACTTATGAGATTAGTTGAGTAGACTTGCCTGAGTTGTCTGCT
Brassica napus                  AGGCGTTTACTTATGAGATTAGTTGAGTAGACTTGCCTGAGTTGTCTGCT
Lotus japonicus                 AGGCGTTTACTTATGAGATTCGTTGAGTAGGCTTGCGTGAGTTGTCTGCT
Millettia pinnata               AGGCGTTTACTTATGAGATTCGTTGAGTAGGCTTGCGTGAGTTGTCTGCT
Citrullus lanatus               AGGCGTTTACTTATGAGATTTGTTGAGTAGGCTTGCGTTAGTTGTCTGCT
Cucurbita pepo                  --------------GAGATTTGTTGAGTAAGCTTGCCTTAGTTGTCCGCT
RRIC110_matR                    --------------------------------------------------
Beta macrocarpa                 AGACCTTTACTTATGACAGGT-TGGTTTGAGGACCCCTTGGTCAAAGGAA
Beta vulgaris maritima          AGACCTTTACTTATGACAGGT-TGGTTTGAGGACCCCTTGGTCAAAGGAA
Beta vulgaris vulgaris          AGACCTTTACTTATGACAGGT-TGGTTTGAGGACCCCTTGGTCAAAGGAA
Silene latifolia                AGACCTTTACTTATGAT--CT-TAGTTTAGTATTCCCTTGGTCAAAGAAA
BPM24_matR                      --------------------------------------------------
PB235_matR                      --------------------------------------------------
Zea mays mays                   AGGCGTTTAC--ATGAGATTAGTTGAGTAGGCTTGCCTTAGTTGTCTGCT
RRIT251_matR                    --------------------------------------------------
RRII105_matR                    --------------------------------------------------
RRIM600_matR                    --------------------------------------------------
Cycas taitungensis              GAAGCGTGATCTATCGGTCTGAACCGTTGTTGCGCGGGGGGGTGTGGGGA

Arabidopsis thaliana            ATAAGATAGAGCTAGTTTTGGGGTAGGGCTTTTGACATAAAAAAGCCGGA
Brassica napus                  ATAAGATAGAGCTAGTTTTGGGGTGGGGCTTTTGACATAAAAAAGCCTAT
Lotus japonicus                 ATAAGATAG--CTAGTTTTGGGGCTTTCGACATAA-ATCAAAAAGCCTAT
Millettia pinnata               ATAAGATAG--CTAGTTTTGGGGCTTTCGACATTA-ATAAAAAAGCCTAT
Citrullus lanatus               ATAAGATAG--CTAGTTTTGGGGC-----TTTCGACATAAAAAAGCCGAT
Cucurbita pepo                  ATAAGATAG--CTAGTTTTGGGGC-----TTTCGACATCAAA-----GAT
RRIC110_matR                    --------------------------------------------------
Beta macrocarpa                 AGAGGGAAACGGGGGAGTTGGCTGATAAAGATGGACAGTAAGGA-TTGCG
Beta vulgaris maritima          AGAGGGAAACGGGGGAGTTGGCTGATAAAGATGGACAGTAAGGA-TTGCG
Beta vulgaris vulgaris          AGAGGGAAACGGGGGAGTTGGCTGATAAAGATGGACAGTAAGGA-TTGCG
Silene latifolia                GAAGGGTCGAAGTTGAGACCGCTCACAGTAAGTAGTTCTAAG-A-TCATC
BPM24_matR                      --------------------------------------------------
PB235_matR                      --------------------------------------------------
Zea mays mays                   ATAAGATAA-------GATAGCTAGTTGTGGTCGAAAAAAAA-----GGC
RRIT251_matR                    --------------------------------------------------
RRII105_matR                    --------------------------------------------------
RRIM600_matR                    --------------------------------------------------
Cycas taitungensis              GGGCGGCCCCACCCACCCTTGGATAGAGCATGCAG-ATGCGCCATGCATG

Arabidopsis thaliana            TAGGCTTGGCTTCGCTATCGCTC--ATGACTTGTATTGTAGTC-------
Brassica napus                  CCGGCTTGGCTTCGCTATCGCTC--ATGACTTGTATTGTAGTC-------
Lotus japonicus                 CCGGCTTGGCTTCGCTATCGCTC--ATGACTTGTATTGTAGTC-------
Millettia pinnata               CCGGCTTGGCTTCGCTATCGCTC--ATGACTTGTATTGTAGTC-------
Citrullus lanatus               CCGGCTTGGCTTCGCTATCGCTC--ATGACTTGTATTGTAGTCCTAGTCT
Cucurbita pepo                  CCGGCTTGGCTTCGCTATCGCTC--ATGACTTGTATTGTAGTCCTAGTCT
RRIC110_matR                    --------------------------------------------------
Beta macrocarpa                 TAATATCAATCGAATTATCACCC--TTT-CTTTTTTTATAACTTTGTT--
Beta vulgaris maritima          TAATATCAATCGAATTATCACCC--TTT-CTTTTTTTATAACTTTGTT--
Beta vulgaris vulgaris          TAATATCAATCGAATTATCACCC--TTT-CTTTTTTTATAACTTTGTT--
Silene latifolia                AGAGAGGAGACAAGCCATTTATG--ATT-CCAGCCTAGAAGACTAGTT--
BPM24_matR                      --------------------------------------------------
PB235_matR                      --------------------------------------------------
Zea mays mays                   TGAA---GGCTTCGCTATCGCTC--ATGGCTTGTATTGTAGTCGTAGTCT
RRIT251_matR                    --------------------------------------------------
RRII105_matR                    --------------------------------------------------
RRIM600_matR                    --------------------------------------------------
Cycas taitungensis              CAAGTTGGCCTTTTGGATCCTTTCGGTAACCGTTTCCGGAGGAGCTTCTC

Arabidopsis thaliana            -------GGCCCGGAATGCCTUGGTAGTCTTT-------CTAATGCCTTC
Brassica napus                  -------GGCCCGGAATGCCTCGGTAGTCTTT-------CTAATGCCTTC
```

```
Lotus japonicus                   -------GGCCCGGAATGCCTTTGTAGTCTTT-TTAATGCTAATGCCTTC
Millettia pinnata                 -------GGCCCGGAATGCCTTTGTAGTCTTT-TTAATGCTAATGCCTTC
Citrullus lanatus                 TGTAGTCGGCCTGGAATGCCTTTGTAGTCTTT-CTAATGCTAATGCCTTC
Cucurbita pepo                    TGTAGTCGGCCTGGAATGCCTTTGTAGTCTTTGCTAATGCGTCTGCCTTC
RRIC110_matR                      --------------------------------------------------
Beta macrocarpa                   ---TGAAAGAGCGGCCTATACTTG-GATTTTT-------GCTTTGCCTTA
Beta vulgaris maritima            ---TGAAAGAGCGGCCTATACTTG-GATTTTT-------GCTTTGCCTTA
Beta vulgaris vulgaris            ---TGAAAGAGCGGCCTATACTTG-GATTTTT-------GCTTTGCCTTA
Silene latifolia                  ---AAAGGAATGGAAAGAATCATGCGATTTTT-------GATCTATCTTT
BPM24_matR                        --------------------------------------------------
PB235_matR                        --------------------------------------------------
Zea mays mays                     TGTAGTCGGCCCTCCATGCCTCTTTAGTCTTT-CTAATCCTGAGGCCTTT
RRIT251_matR                      --------------------------------------------------
RRII105_matR                      --------------------------------------------------
RRIM600_matR                      --------------------------------------------------
Cycas taitungensis                AATGTCTCGACGCATCCAACGCAAAAGTGTTC--GGACGGAGCGGAAAAC


Arabidopsis thaliana              TTCCTTCATTCATTTTCTTTTAGTTGCGGTAGCTTCCGCGCCAGCAAGAT
Brassica napus                    TTCCTTCATTCATTTTCTTTTAGTTGCGGTAGCTTCCGCGCCAGCAAGAT
Lotus japonicus                   TTCCTTCATTCATTTTTTTTTAGTTGCGGCAGCTTCCGCGCCAGCAAGAT
Millettia pinnata                 TTCCTTCATTCATTTTCTTTTAGTTGCGGCAGCTTCCGCGCCAGCAAGAT
Citrullus lanatus                 TTCCTTCATTCATTTTCTTTTAGTTGCGGTAGCTCCCGCGCCAGCAAGAT
Cucurbita pepo                    TTCCTTCATTCATTTGCTTTTAGTTGCGGTAGCTCCCGCGCCAGCAAGAT
RRIC110_matR                      --------------------------------------------------
Beta macrocarpa                   TTT-GAGAAACTTGTCCCAAAAA---GGGCAAATACTTCTATTCCTGAGT
Beta vulgaris maritima            TTT-GAGAAACTTGTCCCAAAAA---GGGCAAATACTTCTATTCCTGAGT
Beta vulgaris vulgaris            TTT-GAGAAACTTGTCCCAAAAA---GGGCAAATACTTCTATTCCTGAGT
Silene latifolia                  TTC-TGCGTCCGTTTCTTACTTGCTCGCCTAGTTTATTTAAACTTTTGGG
BPM24_matR                        --------------------------------------------------
PB235_matR                        --------------------------------------------------
Zea mays mays                     TTCCTTCATTCATTTTGCGGTAGCTTACGCGTCAGAAAAAGGCCTCCTTT
RRIT251_matR                      --------------------------------------------------
RRII105_matR                      --------------------------------------------------
RRIM600_matR                      --------------------------------------------------
Cycas taitungensis                GCCCCTCAATCTCGTTCCAATAA-TGAAGAAGCCGGCCCCCTTCTTTTCC


Arabidopsis thaliana              ACGGACAGCGAAGCCAAAGCAATACTAAACAAGCGAGAAAAGTC------
Brassica napus                    ACGGACAGCGAAGCCAAAGCAATACTAAACAAGCGAGAAAAGTC------
Lotus japonicus                   ACGGACGGCGAAGCCAAAGCAATACTAAACAAGCGAGAAAAGCCCTATAT
Millettia pinnata                 ACGGACGGCGAAGCCAAAGCAATACTAAACAAGCGAGAAAAGCCCTATAT
Citrullus lanatus                 ACAGACG-------------------------------------------
Cucurbita pepo                    AGAGACGGCGAAGCCAAAGCAATACTAAACGAGC----------------
RRIC110_matR                      --------------------------------------------------
Beta macrocarpa                   CTGCGCTTCTTACTCGCGCTCATTCTTTTTAAGG----------------
Beta vulgaris maritima            CTGCGCTTCTTACTCGCGCTCATTCTTTTTAAGG----------------
Beta vulgaris vulgaris            CTGCGCTTCTTACTCGCGCTCATTCTTTTTAAGG----------------
Silene latifolia                  CAGCAGCTTTACTTTATCCCCTTTTTTACTGCATCT--------------
BPM24_matR                        --------------------------------------------------
PB235_matR                        --------------------------------------------------
Zea mays mays                     CCGGCCCGGAAGATCGCTTCGCTTCTGGCGACTAGCTTCTACCC------
RRIT251_matR                      --------------------------------------------------
RRII105_matR                      --------------------------------------------------
RRIM600_matR                      --------------------------------------------------
Cycas taitungensis                AATAATTGATAAGGCACTATTCTTGTATTGTATTGTATTGGGGAAAAGAT


Arabidopsis thaliana              --------------------------------------------------
Brassica napus                    --------------------------------------------------
Lotus japonicus                   ATT-----------------------------------------------
Millettia pinnata                 ATTCGCCTAAACAACTTATTGAAAACTAAAGCGCTAACGAGCAAGAAAAC
```

```
Citrullus lanatus                  --------------------------------------------------
Cucurbita pepo                     --------------------------------------------------
RRIC110_matR                       --------------------------------------------------
Beta macrocarpa                    --------------------------------------------------
Beta vulgaris maritima             --------------------------------------------------
Beta vulgaris vulgaris             --------------------------------------------------
Silene latifolia                   --------------------------------------------------
BPM24_matR                         --------------------------------------------------
PB235_matR                         --------------------------------------------------
Zea mays mays                      --------------------------------------------------
RRIT251_matR                       --------------------------------------------------
RRII105_matR                       --------------------------------------------------
RRIM600_matR                       --------------------------------------------------
Cycas taitungensis                 TGC-----------------------------------------------


###################
```

nad1 multiple sequence alignment

```
BPM24_nad1                         --------------------------------------------------
RRIC110_nad1                       --------------------------------------------------
RRII105_nad1                       --------------------------------------------------
PB235_nad1                         --------------------------------------------------
RRIT251_nad1                       --------------------------------------------------
RRIM600_nad1                       --------------------------------------------------
Arabidopsis thaliana               --------------------------------------------------
Citrullus lanatus                  GAGTGAAAT-ATCATGATCGGGTCGACCAGGCCAG-----ATCATGAGTG
Cucurbita pepo                     CAGTGAAAT-ATCATGATCGGGTCGACCAGGCCAG-----ATCGTCAGTG
Beta macrocarpa                    GAGTCAAAT-ATCATGATCGGGTCGACCAGGCCAG-----ATCATGAGTG
Beta vulgaris vulgaris             --GTCAAAT-ATCATGATCGGGTCGACCAGGCCAG-----ATCATGAGTG
Silene latifolia                   --GTAAAAT-ATCATGATCGGGTCGACCAGGCCAG-----ATCATGAGTG
Millettia pinnata                  TCCTTCAAT-ATCATGATTGGGTCTACCAGGCCAG-----ATCATGAGTG
Vigna radiata                      TCCTTCAAT-ATCATGATTGGGTCGACCAGGCCAG-----ATCATGAGTG
Lotus japonicus                    --CTTCAAT-ATCATGATTGGGTCGACCGGGCCAG-----ATCATGAGTG
Brassica napus                     --CTTCAAT-ATCATGATTGGGTCGACCAGGCCAG-----ATCATGAGTA
Oryza sativa Japonica              ------AGTGAATTTGATCGGGTCGACCAGGTCAGGCCCGATCATGAGTG
Zea mays parviglumis               -----CAAT-AGTTTGATCGGGTCGACCAGGTCAGGCCCGATCATGAGTG
Cycas taitungensis                 GAGTGAAAT-ATCATGATCGGGTCGACCAGGCCCG-----ATCATGAGTG
Megaceros aenigmaticus             --------------------------------------------------
Phaeoceros laevis                  --------------------------------------------------
Huperzia squarrosa                 --------------------------------------------------


BPM24_nad1                         ---------------AUGTACATAGCTGTTCCAGCTGAAATACTTGGAAT
RRIC110_nad1                       ---------------AUGTACATAGCTGTTCCAGCTGAAATACTTGGAAT
RRII105_nad1                       ---------------AUGTACATAGCTGTTCCAGCTGAAATACTTGGAAT
PB235_nad1                         ---------------AUGTACATAGCTGTTCCAGCTGAAATACTTGGAAT
RRIT251_nad1                       ---------------AUGTACATAGCTGTTCCAGCTGAAATACTTGGAAT
RRIM600_nad1                       ---------------ANGTACATAGCTGTTCCAGCTGAAATACTTGGAAT
Arabidopsis thaliana               ---------------AUGTACATAGCTGTTCCAGCTGAAATACTTGGAAT
Citrullus lanatus                  AATAGAAAATCGAAAAUGTACATAGCAGTCCCAGCTGAAATACTTGGAAT
Cucurbita pepo                     AATAGAAAATCGAAAAUGTACATAGCAGTTCCAGCTGAAATACTTGGAAT
Beta macrocarpa                    AATAGAAAATCGAAAAUGTACATAGCTGTTCCAGCTGAAATACTTGGAAT
Beta vulgaris vulgaris             AATAGAAAATCGAAAAUGTACATAGCTGTTCCAGCTGAAATACTTGGAAT
Silene latifolia                   AATAGAAAATCGAAAAUGTACATAGCTGTTCCAGCGGAAATACTTGGAAT
Millettia pinnata                  AATAGAAAATCGAAAAUGTACATAGCTGTTCCAGCTGAAATACTTGGAAT
Vigna radiata                      AATAGAAAATCGAAAAUGTACATAGCTGTTCCAGCTGAAATACTTGGAAT
Lotus japonicus                    AATAGAAAATCGAAAAUGTACATAGCTGTTCCAGCTGAAATACTTGGAAT
Brassica napus                     AATAAAAAATCGAAAAUGTACATAGCTGTTCCAGCTGAAATACTTGGAAT
Oryza sativa Japonica              AATAGAAAATCGAAAAUGTACATAGCTGTTCCAGCGGAAATACTTTGTTT
Zea mays parviglumis               AATAGAAAATCGAAAAUGTACATAGCTGTTCCAGCGGAAATACTTTGTTT
Cycas taitungensis                 AATGGGAAATCGAAAAUGTACATTGCTATTTCAGCTGAAATACCTGGAAT
Megaceros aenigmaticus             ---ATGAAACTTTATAT---CATTGGTATTTTAGCAAAAATACTTGGAAT
```

```
Phaeoceros laevis                  ---ATGAAACTTTATAT---CATTGGTATTTTAGCGAAAATACTTGGAAT
Huperzia squarrosa                 ---AUGAGACCGTATAT---CCTTGGTATCTTAGCTAAAATACTTGGAAT
                                                  *    * * *   *   ***  ****** * *  *

BPM24_nad1                         AATTUTACCACTTCTACTAGGAGTAGCCTTTTTAGTGCTAGCTGAACGTA
RRIC110_nad1                       AATTUTACCACTTCTACTAGGAGTAGCCTTTTTAGTGCTAGCTGAACGTA
RRII105_nad1                       AATTUTACCACTTCTACTAGGAGTAGCCTTTTTAGTGCTAGCTGAACGTA
PB235_nad1                         AATTUTACCACTTCTACTAGGAGTAGCCTTTTTAGTGCTAGCTGAACGTA
RRIT251_nad1                       AATTCTACCACTTCTACTAGGAGTAGCCTTTTTAGTGCTAGCTGAACGTA
RRIM600_nad1                       AATTNTACCACTTCTACTAGGAGTAGCCTTTTTAGTGCTAGCTGAACGTA
Arabidopsis thaliana               AATTCTACCACTTCTACTAGGAGTAGCCTTTTTAGTGCTAGCTGAACGTA
Citrullus lanatus                  AATTCTACCACTTCTACTAGGAGTAGCCTTTTTAGTGCTAGCTGAACGTA
Cucurbita pepo                     AATTCTACCACTTCTACTAGGAGTAGCCTTTTTAGTGCTAGCTGAACGTA
Beta macrocarpa                    AATTCTACCACTTCTACTAGGAGTAGCCTTTTTAGTGCTAGCTGAACGTA
Beta vulgaris vulgaris             AATTCTACCACTTCTACTAGGAGTAGCCTTTTTAGTGCTAGCTGAACGTA
Silene latifolia                   AATTCTACCACTTCTACTAGGAGTAGCCTTTTTAGTGCTAGCTGAACGTA
Millettia pinnata                  AATTCTACCACTTCTACTAGGAGTAGCCTTTTTAGTGCTAGCTGAACGTA
Vigna radiata                      AATTCTACCACTTCTACTAGGAGTAGCCTTTTTAGTGCTAGCTGAACGTA
Lotus japonicus                    AATTCTACCACTTCTACTAGGAGTAGCCTTTTTAGTGCTAGCTGAACGTA
Brassica napus                     AATTCTACCACTTCTACTAGGAGTAGCCTTTTTAGTGCTAGCTGAACGTA
Oryza sativa Japonica              AATTCTACCACTTCTACTAGGAGTAGCCTTTTTAGTGCTAGCTGAACGTA
Zea mays parviglumis               AATTCTACCACTTCTACTAGGAGTAGCCTTTTTAGTGCTAGCTGAACGTA
Cycas taitungensis                 AATCATACCACTTCCACTAGGAGTAGCCTTTTTAGTGCTGGCTGAACGTA
Megaceros aenigmaticus             AGTAATACCACCTTTACTAGGAGTAGCCCTTCTAGTTTCAGCTGAATGTA
Phaeoceros laevis                  AGTAATACCACCTTTACTAGGAGTAGCCCTTCTAGTTTCAGCTGAATGTA
Huperzia squarrosa                 AATAATACCACTTTCACCAGGAGTAGCTTTTTCAGTTTCAGCTGAACGTA
                                   * *  ****** *  ** *********  **  ***    ****** ***

BPM24_nad1                         AAGTAATGGCTTTTGTGCAACGTCGAAAGGGTCCTGATGTAGTGGGATTG
RRIC110_nad1                       AAGTAATGGCTTTTGTGCAACGTCGAAAGGGTCCTGATGTAGTGGGATTG
RRII105_nad1                       AAGTAATGGCTTTTGTGCAACGTCGAAAGGGTCCTGATGTAGTGGGATTG
PB235_nad1                         AAGTAATGGCTTTTGTGCAACGTCGAAAGGGTCCTGATGTAGTGGGATTG
RRIT251_nad1                       AAGTAATGGCTTTTGTGCAACGTCGAAAGGGTCCTGATGTAGTGGGATTG
RRIM600_nad1                       AAGTAATGGCTTTTGTGCAACGTCGAAAGGGTCCTGATGTAGTGGGATTG
Arabidopsis thaliana               AAGTAATGGCTTTTGTGCAACGTAGAAAGGGTCCTGATGTAGTGGGATCG
Citrullus lanatus                  AAGTAATGGCTTTTGTGCAACGTCGAAAGGGTCCTGATGTAGTGGGATCG
Cucurbita pepo                     AAGTAATGGCTTTTGTGCAACGTCGAAAGGGTCCTGATGTAGTGGGATCG
Beta macrocarpa                    AAGTAATGGCTTTTGTGCAACGTCGAAAGGGTCCTGATGTAGTGGGATCG
Beta vulgaris vulgaris             AAGTAATGGCTTTTGTGCAACGTCGAAAGGGTCCTGATGTAGTGGGATCG
Silene latifolia                   AAGTAATGGCTTTTGTGCAACGTCGAAAGGGTCCTGATGTAGTGGGATCG
Millettia pinnata                  AAGTAATGGCTTTTGTGCAACGTCGAAAGGGTCCTGATGTAGTGGGATCG
Vigna radiata                      AAGTAATGGCTTTTGTGCAACGTCGAAAGGGTCCTGATGTAGTGGGATCG
Lotus japonicus                    AAGTAATGGCTTTTGTGCAACGTCGAAAGGGTCCTGATGTAGTGGGATCG
Brassica napus                     AAGTAATGGCTTTTGTGCAACGTAGAAAGGGTCCTGATGTAGTGGGATCG
Oryza sativa Japonica              AAGTAATGGCTTTTGTGCAACGTCGAAAGGGTCCTGATGTAGTGGGATCG
Zea mays parviglumis               AAGTAATGGCTTTTGTGCAACGTCGAAAGGGTCCTGATGTAGTGGGATCG
Cycas taitungensis                 AAGTAATGGCTCCTGTGCAACGTCGAAAGGGTCCTGATGTAGTTGGATCG
Megaceros aenigmaticus             AAGTAACGGCCTCTATGCAACGTAGAAAGGGTCTTAATGTAGTAGGATTG
Phaeoceros laevis                  AAGTAACGGCCTCTACGCAACGTAGAAAGGGTCTTAATGTAGTAGGATTG
Huperzia squarrosa                 AAGTAATGGCTTCTATGCAACGTAGAAAGGGTCCTAATGTAGTAGGATTG
                                   ****** ***   *  ******* ********* * ******* **** *

BPM24_nad1                         TTCGGATTGTTACAACCTCTAGCAGATGGTTUGAAATTGATTCTAAAAGA
RRIC110_nad1                       TTCGGATTGTTACAACCTCTAGCAGATGGTTNGAAATTGATTCTAAAAGA
RRII105_nad1                       TTCGGATTGTTACAACCTCTAGCAGATGGTTUGAAATTGATTCTAAAAGA
PB235_nad1                         TTCGGATTGTTACAACCTCTAGCAGATGGTTUGAAATTGATTCTAAAAGA
RRIT251_nad1                       TTCGGATTGTTACAACCTCTAGCAGATGGTTUGAAATTGATTCTAAAAGA
RRIM600_nad1                       TTCGGATTGTTACAACCTCTAGCAGATGGTTNGAAATTGATTCTAAAAGA
Arabidopsis thaliana               TTCGGATTGTTACAACCTCTAGCAGATGGTTUGAAATTGATTCTAAAAGA
Citrullus lanatus                  TTCGGATTGTTACAACCTCTAGCAGATGGTTTGAAATTGATTCTAAAAGA
Cucurbita pepo                     TTCGGATTGTTACAACCTCTAGCAGATGGTTTGAAATTGATGATAAAAGA
Beta macrocarpa                    TTCGGATTGTTACAACCTCTAGCAGATGGTTUGAAATTGATTCTAAAAGA
```

```
Beta vulgaris vulgaris          TTCGGATTGTTACAACCTCTAGCAGATGGTTUGAAATTGATTCTAAAAGA
Silene latifolia                TTCGGATTGTTACAACCTCTAGCAGATGGTTUAAAATTGATTCTAAAAGA
Millettia pinnata               TTCGGATTGTTACAACCTCTAGCAGATGGTTTGAAATTGATTCTAAAAGA
Vigna radiata                   TTCGGATTGTTACAACCTCTAGCAGATGGTTTGAAATTGATTCTAAAAGA
Lotus japonicus                 TTCGGATTGTTACAACCTCTAGCAGATGGTTTGAAATTGATTCTAAAAGA
Brassica napus                  TTCGGATTGTTACAACCTCTAGCAGATGGTTUGAAATTGATTCTAAAAGA
Oryza sativa Japonica           TTCGGATTGTTACAACCTCTAGCAGATGGTTTGAAATTGATTCTAAAAGA
Zea mays parviglumis            TTCGGATTGTTACAACCTCTAGCAGATGGTTTTAAATTGATTCTAAAAGA
Cycas taitungensis              TTCGGATTGTTACAACCTTTAGCGGATGGTTCGAAATTGGTTCTGAAAGA
Megaceros aenigmaticus          TTCGGATCGTTACAACCCTTAGCAGATGGTTTGAAATTAATGATAAAAGA
Phaeoceros laevis               TTCGGATCGTTACAACCCTTAGCAGATGGTTTGAAATTAATGATAAAAGA
Huperzia squarrosa              TTTGGGTTGTTACAACCTCTAGCAGATGGTTTTAAATTAATTATAAAAGA
                                ** ** * *********  **** *******  *****  *  * *****

BPM24_nad1                      ACCTATTTCACCAAGTAGTGCTAATTTCTUCCTTTTTAGAATGGCTCCAG
RRIC110_nad1                    ACCTATTTCACCAAGTAGTGCTAATTTCTUCCTTTTTAGAATGGCTCCAG
RRII105_nad1                    ACCTATTTCACCAAGTAGTGCTAATTTCTUCCTTTTTAGAATGGCTCCAG
PB235_nad1                      ACCTATTTCACCAAGTAGTGCTAATTTCTUCCTTTTTAGAATGGCTCCAG
RRIT251_nad1                    ACCTATTTCACCAAGTAGTGCTAATTTCTUCCTTTTTAGAATGGCTCCAG
RRIM600_nad1                    ACCTATTTCACCAAGTAGTGCTAATTTCTNCCTTTTTAGAATGGCTCCAG
Arabidopsis thaliana            ACCTATTTCACCAAGTAGTGCTAATTTTTTCCTTTTTAGAATGGCTCCAG
Citrullus lanatus               ACCTCTTTCACCAAGTAGTGCAAATTTUTUCCTTTTTAGAATGGCTCCAG
Cucurbita pepo                  ACCTCTTTCACCAAGTAGTGCTAATTTCTUCCTTTTTAGAATGGCTCCAG
Beta macrocarpa                 ACCTATTTCACCAAGTAGTGCTAATTTCTUCCTTTTTAGAATGGCTCCAG
Beta vulgaris vulgaris          ACCTATTTCACCAAGTAGTGCTAATTTCTUCCTTTTTAGAATGGCTCCAG
Silene latifolia                ACCTATTTCACCAAGTAGTGCTAATTTCTUCCTTTTTAGAATGGCTCCAG
Millettia pinnata               ACCTATTTCACCAAGTAGTGCTAATTTCTUCCTTTTTAGAATGGCTCCAG
Vigna radiata                   ACCTATTTCACCAAGTAGTGCTAATTTCTCCCTTTTTAGAATGGCTCCAG
Lotus japonicus                 ACCTATTTCACCAAGTAGTGCTAATTTCTUCCTTTTTAGAATGGCTCCAG
Brassica napus                  ACCTATTTCACCAAGTAGTGCTAATTTTTTCCTTTTTAGAATGGCTCCAG
Oryza sativa Japonica           ACCTATTTCACCAAGTAGTGCTAATTTCTUCCTTTTTAGAATGGCTCCAG
Zea mays parviglumis            ACCTATTTCACCAAGTAGTGCTAATTTCTCCCTTTTTAGAATGGCTCCAG
Cycas taitungensis              ACCTATTTCACCAAGTAGTGCTAATTTATTTCTTTCCAGAATGGCTCCAG
Megaceros aenigmaticus          ACTTATTCTACCGAGTAGTGCTAATTTATTTATTTCTATAATGGCTCCAG
Phaeoceros laevis               ACTTATTCTACTGAGTAGTGCTAATTTATTTATTTCTATAATGGCTTCAG
Huperzia squarrosa              ACCTATTTTACCAAGTAGTGCTAATTTATTCATTTTTTCAATGGCTCCAG
                                ** * **  **  ******** ***** *   ***    ******* ***

BPM24_nad1                      TGGCTACATTTATGTTAAGTCTGGTCGCTUGGGCCGTTGTACCTTTTGAT
RRIC110_nad1                    TGGCTACATTTATGTTAAGTCTGGTCGCTUGGGCCGTTGTACCTTTTGAT
RRII105_nad1                    TGGCTACATTTATGTTAAGTCTGGTCGCTUGGGCCGTTGTACCTTTTGAT
PB235_nad1                      TGGCTACATTTATGTTAAGTCTGGTCGCTUGGGCCGTTGTACCTTTTGAT
RRIT251_nad1                    TGGCTACATTTATGTTAAGTCTGGTCGCTNGGGCCGTTGTACCTTTTGAT
RRIM600_nad1                    TGGCTACATTTATGTTAAGTCTGGTCGCTNGGGCCGTTGTACCTTTTGAT
Arabidopsis thaliana            TGGCTACATTTATGTTAAGTCTGGTCGCTUGGGCCGTTGTACCTTTTGAT
Citrullus lanatus               TGGCTACATTTATGTTAAGTCTGGTCGCTUGGGCCGTTGTACCTTTTGAT
Cucurbita pepo                  TGGCTACATTTATGTTAAGTCTGGTCGCTUGGGCCGTTGTACCTTTTGAT
Beta macrocarpa                 TCACTACATTTATGCTAAGTCTGGTTGCTUGGGCCGTTGTACCTTTTGAT
Beta vulgaris vulgaris          TCACTACATTTATGCTAAGTCTGGTTGCTUGGGCCGTTGTACCTTTTGAT
Silene latifolia                TCACTACATTTATGCTAAGTCTGGTTGCTUGGGCCGTTGTACCTTTTGAT
Millettia pinnata               TGGCTACATTTATGTTAAGTCTGGTCGCTUGGGCCGTTGTACCTTTTGAT
Vigna radiata                   TGGCTACATTTATGTTAAGTCTGGTCGCTCGGGCCGTTGTACCTTTTGAT
Lotus japonicus                 TGGCTACATTTATGTTAAGTCTGGTCGCTUGGGCCGTTGTACCTTTTGAT
Brassica napus                  TGGCTACATTTATGTTAAGTCTGGTCGCTUGGGCCGTTGTACCTTTTGAT
Oryza sativa Japonica           TGGCTACATTTATGTTAAGTCTGGTCGCTTGGGCCGTTGTACCTTTTGAT
Zea mays parviglumis            TGGCTACATTTATGTTAAGTCTGGTCGCTTGGGCCGTTGTACCTTTTGAT
Cycas taitungensis              TGGTTACATCTATGTCAAGTTCGGTTGCTCGGGCCGTTGTACCTTTTGAT
Megaceros aenigmaticus          TAATTACATTTATGTCAAGTTCGGTTGCTTGGGCTGTTATACCTTTTGAT
Phaeoceros laevis               TAATTACATTTATGTCAAGTTCGGTTGCTTGGGCTGTTATACTTTTTGAT
Huperzia squarrosa              TAATTACATTTACGTCAAGTTTGGTTGCTCGGGCTGTTACACCTTTCGAT
                                *   ***** ** *  ****  *** *** **** ***  ** *** ***
```

```
BPM24_nad1                 TATGGTATGGTATTGTCAGATCCGAACATAGGGCTACTTTATTTGTTTGC
RRIC110_nad1               TATGGTATGGTATTGTCAGATCCGAACATAGGGCTACTTTATTTGTTTGC
RRII105_nad1               TATGGTATGGTATTGTCAGATCCGAACATAGGGCTACTTTATTTGTTTGC
PB235_nad1                 TATGGTATGGTATTGTCAGATCCGAACATAGGGCTACTTTATTTGTTTGC
RRIT251_nad1               TATGGTATGGTATTGTCAGATCCGAACATAGGGCTACTTTATTTGTTTGC
RRIM600_nad1               TATGGTATGGTATTGTCAGATCCGAACATAGGGCTACTTTATTTGTTTGC
Arabidopsis thaliana       TATGGTATGGTATTGTCAGATUUGAACATAGGGCTACTTTATTTGTTTGC
Citrullus lanatus          TATGGTATGGTATTGTCAGATCUGAACATAGGGCTACTTTATTTGTTTGC
Cucurbita pepo             TATGGGATGGTATTGTCAGATCUGAACATAGGGCTACTTTATTTGTTTGC
Beta macrocarpa            TATGGTATGGTATTGTCAGATUUGAACATAGGGCTACTTTATTTGTTTGC
Beta vulgaris vulgaris     TATGGTATGGTATTGTCAGATUUGAACATAGGGCTACTTTATTTGTTTGC
Silene latifolia           TATGGTATGGTATTGTCAGATUUGAACATAGGGCTACTTTATTTGTTTGC
Millettia pinnata          TATGGTATGGTATTGTCAGATTUGAACATAGGGCTACTTTATTTGTTTGC
Vigna radiata              TATGGTATGGTATTGTCAGATTCGAACATAGGGCTACTTTATTTGTTTGC
Lotus japonicus            TATGGTATGGTATTGTCAGATTUGAACATAGGGCTACTTTATTTGTTTGC
Brassica napus             TATGGTATGGTATTGTCAGATUUGAACATAGGGCTACTTTATTTGTTTGC
Oryza sativa Japonica      TATGGTATGGTATTGTCAGATUUGAACATAGGGCTACTTTATTTGTTTGC
Zea mays parviglumis       TATGGTATGGTATTGTCAGATCCGAACATAGGGCTACTTTATTTGTTTGC
Cycas taitungensis         TATGGTATGGTATCGCCAGATTCGGACATAGGTATACTTCATCTGTTTGC
Megaceros aenigmaticus     TATGGTATGGTATTGTCAGATTCAAATGTAGGTATACTTCATCTATTCGC
Phaeoceros laevis          TATGGTATGGTATTGTCAGATTCAAATGTAGGTATACTTCATCTATTCGC
Huperzia squarrosa         TATGGTATGGTATTGTCAGATTCAAATGTAGGTATACTTTATTTATTTGC
                           ***** ******* * *****    *  ****  ***** ** * ** **

BPM24_nad1                 CATATCTTCGCTAGGTGTTTATGGAATTATTATAGCAGGTCGGTCTAGTA
RRIC110_nad1               CATATCTTCGCTAGGTGTTTATGGAATTATTATAGCAGGTCGGTCTAGTA
RRII105_nad1               CATATCTTCGCTAGGTGTTTATGGAATTATTATAGCAGGTCGGTCTAGTA
PB235_nad1                 CATATCTTCGCTAGGTGTTTATGGAATTATTATAGCAGGTCGGTCTAGTA
RRIT251_nad1               CATATCTTCGCTAGGTGTTTATGGAATTATTATAGCAGGTCGGTCTAGTA
RRIM600_nad1               CATATCTTCGCTAGGTGTTTATGGAATTATTATAGCAGGTCGGTCTAGTA
Arabidopsis thaliana       CATATCTTCGCTAGGTGTTTATGGAATTATTATAGCGGGTUGGTCTAGTA
Citrullus lanatus          CATATCTTCGCTAGGTGTTTATGGAATTATTATAGCGGGTTGGTCTAGTA
Cucurbita pepo             CATATCTTCGCTAGGTGTTTATGGAATTATTATCGCGGGTTGGTCTAGTA
Beta macrocarpa            CATATCTTCGCTAGGTGTTTATGGAATTATTATAGCAGGTTGGTCTAGTA
Beta vulgaris vulgaris     CATATCTTCGCTAGGTGTTTATGGAATTATTATAGCAGGTTGGTCTAGTA
Silene latifolia           CATATCTTCGCTAGGTGTTTATGGAATTATTATAGCAGGTTGGTCTAGTA
Millettia pinnata          CATATCTTCGTTAGGTGTTTATGGAATTATTAUAGCGGGTTGGTCTAGTA
Vigna radiata              CATATCTTCGTTAGGTGTTTATGGAATTATTACAGCGGGTTGGTCTAGTA
Lotus japonicus            CATATCTTCGTTAGGTGTTTATGGAATTATTAUAGCAGGTTGGTCTAGTA
Brassica napus             CATATCTTCGCTAGGTGTTTATGGAATTATTATAGCGGGTUGGTCTAGTA
Oryza sativa Japonica      CATATCTTCGCTAGGTGTTTATGGAATAATTATAGCAGGTTGGTCTAGTA
Zea mays parviglumis       CATATCTTCGCTAGGTGTTTATGGAATAATTATAGCAGGTTGGTCTAGTA
Cycas taitungensis         CATATCTTCGCTAGGTGTTCATGGGATTATTACAGCGGGTTGGTCTAGTA
Megaceros aenigmaticus     TATATCTTCTCTAGGTGTTTATGGGATTATTACGGTGGGTCGGTTAGTA
Phaeoceros laevis          TATATCCTCTCTAGGTGTTTATGGGATTATTACGGTGGGTCGGTTAGTA
Huperzia squarrosa         TATTTCTTCTCTAGGTGTTTATGGAATTATTACGGCGGGTTGGTCTAGTA
                            ** ** **  ******** **** ** ****  *  *** **  *****

BPM24_nad1                 AT-TCGAAATATGCCTTTCTAGGAGCATTACGATCTGCAGCTCAAATGGT
RRIC110_nad1               AT-TCGAAATATGCCTTTCTAGGAGCATTACGATCTGCAGCTCAAATGGT
RRII105_nad1               AT-TCGAAATATGCCTTTCTAGGAGCATTACGATCTGCAGCTCAAATGGT
PB235_nad1                 AT-TCGAAATATGCCTTTCTAGGAGCATTACGATCTGCAGCTCAAATGGT
RRIT251_nad1               AT-TCGAAATATGCCTTTCTAGGAGCATTACGATCTGCAGCTCAAATGGT
RRIM600_nad1               AT-TCGAAATATGCCTTTCTAGGAGCATTACGATCTGCAGCTCAAATGGT
Arabidopsis thaliana       AT-TCGAAATATGCCTTTCTAGGAGCATTACGATCTGCAGCTCAAATGGT
Citrullus lanatus          AT--TAGGGGGCGGCCGTTCGGTCGC-CTATGATACTAGGACCAATAGGT
Cucurbita pepo             AT--TAGGGGGCGGCCGTTCGGTCGC-CTATGATACTAGGACTAATAGGT
Beta macrocarpa            ATTATAGGGGGCGGCCGTTCGGTCGC-CTATGATACTAGGACCAATAGGT
Beta vulgaris vulgaris     ATTATAGGGGGCGGCCGTTCGGTCGC-CTATGATACTAGGACCAATAGGT
Silene latifolia           ATTTTAGGGGGCGGCCGTTCGGTCGC-CTATGATACTAGGACCAATAGGT
Millettia pinnata          AT--TAGGGGGCGGCCGTTCGGTCGC-CTATGATACTAGGACCAATAGGT
Vigna radiata              AT--TAGGGGGCGGCCGTTCGGTCGC-CTATGATACTAGGACCAATAGGT
```

```
Lotus japonicus                   AT--TAGGGGGCGGCCGTTCGGTCGC-CTATGATACTAGGACCAATAGGT
Brassica napus                    AT--TAGGGGGCGGCCGTTCGGTCGC-CTATGAGACTAGGACCAATAGGT
Oryza sativa Japonica             AG-ACGGGGGGCGGCCGTTCGGTCGC-CTATGATATACGGACCAATTGGT
Zea mays parviglumis              AG-ACGGGGGGCGGCCGTTCGGTCGC-CTATGATATACGGACCAATTGGT
Cycas taitungensis                AT-TAGGGGGGCGGCCGTTCGGTCGC-CTATGATACTAAGGCCAATGGGT
Megaceros aenigmaticus            AT-CCAAAATATGCGTCTTCAGGAGCATCACGATCTGCAGCTCAAACGGT
Phaeoceros laevis                 AT-CTAAAATATGTGTCTTCAGGAGCATCACGATTTGCAGTTCAAACGGT
Huperzia squarrosa                AT-TCTAAATATGCTTTTTCAGGAGCATTACGATCTGCAGCTCAAATGGT
                                  *           *    *   *  **   * **      *   **  ***

BPM24_nad1                        CTCTTATGAAGTCTCTATTGGTCTTATTCTTATTACTGTACTAATATGTG
RRIC110_nad1                      CTCTTATGAAGTCTCTATTGGTCTTATTCTTATTACTGTACTAATATGTG
RRII105_nad1                      CTCTTATGAAGTCTCTATTGGTCTTATTCTTATTACTGTACTAATATGTG
PB235_nad1                        CTCTTATGAAGTCTCTATTGGTCTTATTCTTATTACTGTACTAATATGTG
RRIT251_nad1                      CTCTTATGAAGTCTCTATTGGTCTTATTCTTATTACTGTACTAATATGTG
RRIM600_nad1                      CTCTTATGAAGTCTCTATTGGTCTTATTCTTATTACTGTACTAATATGTG
Arabidopsis thaliana              CTCTTATGAAGTATCTATTGGTCTTATTCTTATTACTGTACTAATATGTG
Citrullus lanatus                 CAAAAATGGGTT--------------------------------------
Cucurbita pepo                    CAAAAATGGGTT--------------------------------------
Beta macrocarpa                   AAAAAATAG-----------------------------------------
Beta vulgaris vulgaris            AAAAAATAGCT---------------------------------------
Silene latifolia                  AAAAAATGGCT---------------------------------------
Millettia pinnata                 CAAAAATGGGTT--------------------------------------
Vigna radiata                     CAAAAATGGGTT--------------------------------------
Lotus japonicus                   CAAAAATGGGTTTG------------------------------------
Brassica napus                    CAAAAATTGGTTTG------------------------------------
Oryza sativa Japonica             CAAAAATGGG----------------------------------------
Zea mays parviglumis              CAAAAATGGG----------------------------------------
Cycas taitungensis                CAAAAATTG-----------------------------------------
Megaceros aenigmaticus            TCTTTATGAAGTTTCTATTGGTCCCATTATTATTACTGTATCAATCCGTG
Phaeoceros laevis                 TCTTTATGAAGTTTCTATTGGTCCCATTATTATTACTGTATTAATCTGTG
Huperzia squarrosa                TTCTTATGAAGTCTCTATTGGTCTTATTATTATTACTGTACTAATCCGTG
                                       **

BPM24_nad1                        TAGGTCCCCGTAATTCGAGTGAGATTGTCATGGCGCAAAAGCAGATATGG
RRIC110_nad1                      TAGGTCCCCGTAATTCGAGTGAGATTGTCATGGCGCAAAAGCAGATATGG
RRII105_nad1                      TAGGTCCCCGTAATTCGAGTGAGATTGTCATGGCGCAAAAGCAGATATGG
PB235_nad1                        TAGGTCCCCGTAATTCGAGTGAGATTGTCATGGCGCAAAAGCAGATATGG
RRIT251_nad1                      TAGGTCCCCGTAATTCGAGTGAGATTGTCATGGCGCAAAAGCAGATATGG
RRIM600_nad1                      TAGGTCCCCGTAATTCGAGTGAGATTGTCATGGCGCAAAAGCAGATATGG
Arabidopsis thaliana              TAGGTUCUUGTAATTUGAGTGAGATTGTCATGGCGCAAAAGCAGATATGG
Citrullus lanatus                 --------------------------------------------------
Cucurbita pepo                    --------------------------------------------------
Beta macrocarpa                   --------------------------------------------------
Beta vulgaris vulgaris            --------------------------------------------------
Silene latifolia                  --------------------------------------------------
Millettia pinnata                 --------------------------------------------------
Vigna radiata                     --------------------------------------------------
Lotus japonicus                   --------------------------------------------------
Brassica napus                    --------------------------------------------------
Oryza sativa Japonica             --------------------------------------------------
Zea mays parviglumis              --------------------------------------------------
Cycas taitungensis                --------------------------------------------------
Megaceros aenigmaticus            TAGGTCCTCGTAATCTTAGTGAGATTGTCATGGCGCAAAAGCAGATATGG
Phaeoceros laevis                 TAGGTTCTCGTAATTTTAGTGAGATTGTCATAGCGCAAAAGCAGATATGG
Huperzia squarrosa                TAGGTTCTTGCAATTTCAGTGAGATTGTCATAGCACAAAAGCAAATATGG

BPM24_nad1                        TCTGGTATTCCCTTGTTCCCTGTATTGGTTATGTTCCTUATTTCTTGTCT
RRIC110_nad1                      TCTGGTATTCCCTTGTTCCCTGTATTGGTTATGTTCCTUATTTCTTGTCT
RRII105_nad1                      TCTGGTATTCCCTTGTTCCCTGTATTGGTTATGTTCCTCATTTCTTGTCT
PB235_nad1                        TCTGGTATTCCCTTGTTCCCTGTATTGGTTATGTTCCTUATTTCTTGTCT
```

```
RRIT251_nad1                    TCTGGTATTCCCTTGTTCCCTGTATTGGTTATGTTCCTNATTTCTTGTCT
RRIM600_nad1                    TCTGGTATTCCCTTGTTCCCTGTATTGGTTATGTTCCTNATTTCTTGTCT
Arabidopsis thaliana            TUTGGTATTCCUCTGTTCCCTGTATTGGTTATGTTCUTTATTTCTUGTCT
Citrullus lanatus               --------------------------------------------------
Cucurbita pepo                  --------------------------------------------------
Beta macrocarpa                 --------------------------------------------------
Beta vulgaris vulgaris          --------------------------------------------------
Silene latifolia                --------------------------------------------------
Millettia pinnata               --------------------------------------------------
Vigna radiata                   --------------------------------------------------
Lotus japonicus                 --------------------------------------------------
Brassica napus                  --------------------------------------------------
Oryza sativa Japonica           --------------------------------------------------
Zea mays parviglumis            --------------------------------------------------
Cycas taitungensis              --------------------------------------------------
Megaceros aenigmaticus          TTCGGCATTCCTTTGTTTCCTGTATTGATTATGTTTTTCATTTCTCGTCT
Phaeoceros laevis               TTCGGCATTCCTTTGTTTCCAGTATTGATTATGTTTTTCATTTCTTGTCT
Huperzia squarrosa              TTTGGCATTCCCTTGTTTCCTGTATTGATTATGTTTTTTATTTCTCGTCT


BPM24_nad1                      AGCAGAAACTAATCGAGCTCCGTTTGATCTCCCAGAAGCGGAAGCTGAAT
RRIC110_nad1                    AGCAGAAACTAATCGAGCTCCGTTTGATCTCCCAGAAGCGGAAGCTGAAT
RRII105_nad1                    AGCAGAAACTAATCGAGCTCCGTTTGATCTCCCAGAAGCGGAAGCTGAAT
PB235_nad1                      AGCAGAAACTAATCGAGCTCCGTTTGATCTCCCAGAAGCGGAAGCTGAAT
RRIT251_nad1                    AGCAGAAACTAATCGAGCTCCGTTTGATCTCCCAGAAGCGGAAGCTGAAT
RRIM600_nad1                    AGCAGAAACTAATCGAGCTCCGTTTGATCTCCCAGAAGCGGAAGCTGAAT
Arabidopsis thaliana            AGCAGAAACTAATCGAGCTCCGTTTGATCTCCCAGAAGCGGAAGCTGAAT
Citrullus lanatus               --------------------------------------------------
Cucurbita pepo                  --------------------------------------------------
Beta macrocarpa                 --------------------------------------------------
Beta vulgaris vulgaris          --------------------------------------------------
Silene latifolia                --------------------------------------------------
Millettia pinnata               --------------------------------------------------
Vigna radiata                   --------------------------------------------------
Lotus japonicus                 --------------------------------------------------
Brassica napus                  --------------------------------------------------
Oryza sativa Japonica           --------------------------------------------------
Zea mays parviglumis            --------------------------------------------------
Cycas taitungensis              --------------------------------------------------
Megaceros aenigmaticus          AGCAGAAACTAATAGGGCTTTTTCTGATCCACCAGAAGCAGAAGCGGAAT
Phaeoceros laevis               AGCAGAAATTAATAGGGCTTTTTTTGATCCACTAGAAGTAGAAGCGGAAT
Huperzia squarrosa              AGCAGAAACTAATCGGGCCCCGTTTGATCTACCAGAAGCAGAAGCTGAAT


BPM24_nad1                      UAGTTGCAGGCTATAATGTAGAATATTCTTCAATGGGGTCTGCTCTTTTT
RRIC110_nad1                    UAGTTGCAGGCTATAATGTAGAATATTCTTCAATGGGGTCTGCTCTTTTT
RRII105_nad1                    CAGTTGCAGGCTATAATGTAGAATATTCTTCAATGGGGTCTGCTCTTTTT
PB235_nad1                      CAGTTGCAGGCTATAATGTAGAATATTCTTCAATGGGGTCTGCTCTTTTT
RRIT251_nad1                    NAGTTGCAGGCTATAATGTAGAATATTCTTCAATGGGGTCTGCTCTTTTT
RRIM600_nad1                    NAGTTGCAGGCTATAATGTAGAATATTCTTCAATGGGGTCTGCTCTTTTT
Arabidopsis thaliana            UAGTTGCAGGCTATAATGTAGAATATTCTTCAATGGGGTCTGCTCTTTTT
Citrullus lanatus               --------------------------------------------------
Cucurbita pepo                  --------------------------------------------------
Beta macrocarpa                 --------------------------------------------------
Beta vulgaris vulgaris          --------------------------------------------------
Silene latifolia                --------------------------------------------------
Millettia pinnata               --------------------------------------------------
Vigna radiata                   --------------------------------------------------
Lotus japonicus                 --------------------------------------------------
Brassica napus                  --------------------------------------------------
Oryza sativa Japonica           --------------------------------------------------
Zea mays parviglumis            --------------------------------------------------
```

```
Cycas taitungensis                  --------------------------------------------------
Megaceros aenigmaticus              CAGTCGCGGGCTATAATGTAGAATATCCTCTAATGGGGTTTGCTCCCCCT
Phaeoceros laevis                   CAGTCGCGGGCTATAATGTAGAATATTTTTTAATGGGGTTTGCTCCTCTC
Huperzia squarrosa                  TAGTCGCGGGCTATAATGTGGAATATTCTTCAATGGGGTTTGCTCTTTTT


BPM24_nad1                          TTTTTGGGAGAGTATGCCAATATGATCTTAATGAGTGGTCCATGCACATC
RRIC110_nad1                        TTTTTGGGAGAGTATGCCAATATGATCTTAATGAGTGGTCCATGCACATC
RRII105_nad1                        TTTTTGGGAGAGTATGCCAATATGATCTTAATGAGTGGTCCATGCACATC
PB235_nad1                          TTTTTGGGAGAGTATGCCAATATGATCTTAATGAGTGGTCCATGCACATC
RRIT251_nad1                        TTTTTGGGAGAGTATGCCAATATGATCTTAATGAGTGGTCCATGCACATC
RRIM600_nad1                        TTTTTGGGAGAGTATGCCAATATGATCTTAATGAGTGGTCCATGCACATC
Arabidopsis thaliana                TTTTTGGGAGAGTATGCCAATATGATCTTAATGAGTGGTCUATGCACATT
Citrullus lanatus                   --------------------------------------------------
Cucurbita pepo                      --------------------------------------------------
Beta macrocarpa                     --------------------------------------------------
Beta vulgaris vulgaris              --------------------------------------------------
Silene latifolia                    --------------------------------------------------
Millettia pinnata                   --------------------------------------------------
Vigna radiata                       --------------------------------------------------
Lotus japonicus                     --------------------------------------------------
Brassica napus                      --------------------------------------------------
Oryza sativa Japonica               --------------------------------------------------
Zea mays parviglumis                --------------------------------------------------
Cycas taitungensis                  --------------------------------------------------
Megaceros aenigmaticus              TCCCTAGGAGAGTATGCTAATATGATTTTAATGAGTAGTCTATGTACATT
Phaeoceros laevis                   TCTTTAGGAGAGTATGCTAATATGATTTTAATGAGTAGTCTATGTACATT
Huperzia squarrosa                  TTTTCAGGTGAGTATGCTAATACGATCTTAATGAGTAGTCTATGTACATT


BPM24_nad1                          GCTCTCTCCAGGAGGTTGGCCGCCTATCCTAGATCTTCCCATTTTCAAAA
RRIC110_nad1                        GCTCTCTCCAGGAGGTTGGCCGCCTATCCTAGATCTTCCCATTTTCAAAA
RRII105_nad1                        GCTCTCTCCAGGAGGTTGGCCGCCTATCCTAGATCTTCCCATTTTCAAAA
PB235_nad1                          GCTCTCTCCAGGAGGTTGGCCGCCTATCCTAGATCTTCCCATTTTCAAAA
RRIT251_nad1                        GCTCTCTCCAGGAGGTTGGCCGCCTATCCTAGATCTTCCCATTTTCAAAA
RRIM600_nad1                        GCTCTCTCCAGGAGGTTGGCCGCCTATCCTAGATCTTCCCATTTTCAAAA
Arabidopsis thaliana                GTTCTTTCUAGGAGGTTGGCUGCCTATCUTAGATCTTCCCATTTTCAAGA
Citrullus lanatus                   --------------------------------------------------
Cucurbita pepo                      --------------------------------------------------
Beta macrocarpa                     --------------------------------------------------
Beta vulgaris vulgaris              --------------------------------------------------
Silene latifolia                    --------------------------------------------------
Millettia pinnata                   --------------------------------------------------
Vigna radiata                       --------------------------------------------------
Lotus japonicus                     --------------------------------------------------
Brassica napus                      --------------------------------------------------
Oryza sativa Japonica               --------------------------------------------------
Zea mays parviglumis                --------------------------------------------------
Cycas taitungensis                  --------------------------------------------------
Megaceros aenigmaticus              GCCCTCTTTAGGAGGTTGGCCGCCTATCCCAGATTTTCCTATTTTCGAGG
Phaeoceros laevis                   GCTCCTTTTAGGGGGTTGGCCGCCTATCTCAGATTTCCCTATTTTCGAGG
Huperzia squarrosa                  GCTTTTTTTGGGAGGTTGGCTGCCCATCCTAGATATTCCTATTCTCCGGG


BPM24_nad1                          AGATCCCGGGCTCGATCTGGTTTAGTATCAAGGTGATTUTCTTTCTCTTT
RRIC110_nad1                        AGATCCCGGGCTCGATCTGGTTTAGTATCAAGGTGATTUTCTTTCTCTTT
RRII105_nad1                        AGATCCCGGGCTCGATCTGGTTTAGTATCAAGGTGATTUTCTTTCTCTTT
PB235_nad1                          AGATCCCGGGCTCGATCTGGTTTAGTATCAAGGTGATTUTCTTTCTCTTT
RRIT251_nad1                        AGATCCCGGGCTCGATCTGGTTTAGTATCAAGGTGATTUTCTTTCTCTTT
RRIM600_nad1                        AGATCCCGGGCTCGATCTGGTTTAGTATCAAGGTGATTCTCTTTCTCTTT
Arabidopsis thaliana                AGATCCCGGGCTUGATCTGGTTTAGTATCAAGGTTCTTUTCTTTCTGTTC
Citrullus lanatus                   --------------------------------------------------
```

```
Cucurbita pepo                    --------------------------------------------------
Beta macrocarpa                   --------------------------------------------------
Beta vulgaris vulgaris            --------------------------------------------------
Silene latifolia                  --------------------------------------------------
Millettia pinnata                 --------------------------------------------------
Vigna radiata                     --------------------------------------------------
Lotus japonicus                   --------------------------------------------------
Brassica napus                    --------------------------------------------------
Oryza sativa Japonica             --------------------------------------------------
Zea mays parviglumis              --------------------------------------------------
Cycas taitungensis                --------------------------------------------------
Megaceros aenigmaticus            CGATTTCGGGCTCTATTTGGTTCAGTATCAAGGTTCTTTTCTTTCCGCCC
Phaeoceros laevis                 GGATTTCGGGCTCTATTTGGTTCAGTATCAAGGTTCCTTTCTTTTCGTTC
Huperzia squarrosa                TGATTCCGGGCTCGATCTGGTTCAGTATCAAGGTTCTTCTCTTTCTGTTT


BPM24_nad1                        CTATATATATGGGTCCGTGCAGCATTTCCACGATATCGTTATGATCAATT
RRIC110_nad1                      CTATATATATGGGTCCGTGCAGCATTTCCACGATATCGTTATGATCAATT
RRII105_nad1                      CTATATATATGGGTCCGTGCAGCATTTCCACGATATCGTTATGATCAATT
PB235_nad1                        CTATATATATGGGTCCGTGCAGCATTTCCACGATATCGTTATGATCAATT
RRIT251_nad1                      CTATATATATGGGTCCGTGCAGCATTTCCACGATATCGTTATGATCAATT
RRIM600_nad1                      CTATATATATGGGTCCGTGCAGCATTTCCACGATATCGTTATGATCAATT
Arabidopsis thaliana              CTATATATATGGGTCCGTGCAGCATTTCCACGATATCGTTATGATCAATT
Citrullus lanatus                 --------------------------------------------------
Cucurbita pepo                    --------------------------------------------------
Beta macrocarpa                   --------------------------------------------------
Beta vulgaris vulgaris            --------------------------------------------------
Silene latifolia                  --------------------------------------------------
Millettia pinnata                 --------------------------------------------------
Vigna radiata                     --------------------------------------------------
Lotus japonicus                   --------------------------------------------------
Brassica napus                    --------------------------------------------------
Oryza sativa Japonica             --------------------------------------------------
Zea mays parviglumis              --------------------------------------------------
Cycas taitungensis                --------------------------------------------------
Megaceros aenigmaticus            ATACATATACGGGTCCGTGCAGCATTTCCAAGATATCGTTATGATCAATC
Phaeoceros laevis                 ATACATATACGGGTCTGTGCAGCATTTTCAAGATATCGTTATGATCAATC
Huperzia squarrosa                GTATATATATGGGTCCGTGCGGCATTTCCACGATATCGTTATGATCAATT


BPM24_nad1                        AATGGGACTTGGCUGGAAAGTGTTCTTGCCTCTATCATTAGCTUGGGTAG
RRIC110_nad1                      AATGGGACTTGGCUGGAAAGTGTTCTTGCCTCTATCATTAGCTUGGGTAG
RRII105_nad1                      AATGGGACTTGGCUGGAAAGTGTTCTTGCCTCTATCATTAGCTUGGGTAG
PB235_nad1                        AATGGGACTTGGCUGGAAAGTGTTCTTGCCTCTATCATTAGCTUGGGTAG
RRIT251_nad1                      AATGGGACTTGGCUGGAAAGTGTTCTTGCCTCTATCATTAGCTUGGGTAG
RRIM600_nad1                      AATGGGACTTGGCNGGAAAGTGTTCTTGCCTCTATCATTAGCTNGGGTAG
Arabidopsis thaliana              AATGGGACTTGGCUGGAAAGTGTTCTTGCCTCTATCATTAGCTUGGGTAG
Citrullus lanatus                 --------------------------------------------------
Cucurbita pepo                    --------------------------------------------------
Beta macrocarpa                   --------------------------------------------------
Beta vulgaris vulgaris            --------------------------------------------------
Silene latifolia                  --------------------------------------------------
Millettia pinnata                 --------------------------------------------------
Vigna radiata                     --------------------------------------------------
Lotus japonicus                   --------------------------------------------------
Brassica napus                    --------------------------------------------------
Oryza sativa Japonica             --------------------------------------------------
Zea mays parviglumis              --------------------------------------------------
Cycas taitungensis                --------------------------------------------------
Megaceros aenigmaticus            AATGAGACTTGGCTGGAAAGTATTCTTGCCTTTATCATTAGCACGGGTAG
Phaeoceros laevis                 AATGAGACTTGGCTGGAAAGTATTCTTGCCTTTATCATTAGCATGGGTAG
Huperzia squarrosa                AATGAGACTTGGCTGGAAAGTATTTTTGCCTTTATCATTAGCATGGGTAG
```

```
BPM24_nad1                        TCUCCGTTTCTGGTGTTTTAGTCACCTTTCAATGGCTCCCTTAA
RRIC110_nad1                      TCUCCGTTTCTGGTGTTTTAGTCACCTTTCAATGGCTCCCTTAA
RRII105_nad1                      TCUCCGTTTCTGGTGTTTTAGTCACCTTTCAATGGCTCCCTTAA
PB235_nad1                        TCUCCGTTTCTGGTGTTTTAGTCACCTTTCAATGGCTCCCTTAA
RRIT251_nad1                      TCUCCGTTTCTGGTGTTTTAGTCACCTTTCAATGGCTCCCTTAA
RRIM600_nad1                      TCCCCGTTTCTGGTGTTTTAGTCACCTTTCAATGGCTCCCTTAA
Arabidopsis thaliana              TCUCTGTTTCTGGTCTTTTAGTCACCTTTCAATGGCTTCCTTAA
Citrullus lanatus                 --------------------------------------------
Cucurbita pepo                    --------------------------------------------
Beta macrocarpa                   --------------------------------------------
Beta vulgaris vulgaris            --------------------------------------------
Silene latifolia                  --------------------------------------------
Millettia pinnata                 --------------------------------------------
Vigna radiata                     --------------------------------------------
Lotus japonicus                   --------------------------------------------
Brassica napus                    --------------------------------------------
Oryza sativa Japonica             --------------------------------------------
Zea mays parviglumis              --------------------------------------------
Cycas taitungensis                --------------------------------------------
Megaceros aenigmaticus            TCTTTGTATCTGGTATTCCAGTAGCCTTTGACTGGCTCCCGTAA
Phaeoceros laevis                 TCTTTGTATCTGGTATTCCAGTAGCCTTTGACTGGCTCCCGUAA
Huperzia squarrosa                TCTTTGTATCTGGTGTTCTAATAACCTTTGACTGGCTTCCTUGA
```

###################

nad2 multiple sequence alignment

```
Arabidopsis thaliana              ATGAAAGCAGAATTCGTTCGGATCCTCCCACACATGTTCAATCTT---TT
Brassica napus                    ---------------------------------ATGTTCAATCTT---TT
Lotus japonicus                   ---------------------------------ATGTTCAATCTT---TT
Citrullus lanatus                 ---------------------------------ATGTTCAATCTT---TT
Cucurbita pepo                    ---------------------------------ATGTTCAATCTT---TT
Beta vulgaris maritima            ---------------------------------ATGTTCAATCTT---TT
Beta vulgaris vulgaris            ---------------------------------ATGTTCAATCTT---TT
Silene latifolia                  ---------------------------------ATGTTCAATCTT---TT
BPM24_nad2                        ---------------------------------ATGTTCAATCTT---TT
RRII105_nad2                      ---------------------------------ATGTTCAATCTT---TT
RRIC110_nad2                      ---------------------------------ATGTTCAATCTT---TT
PB235_nad2                        ---------------------------------ATGTTCAATCTT---TT
RRIT251_nad2                      ---------------------------------ATGTTCAATCTT---TT
RRIM600_nad2                      ---------------------------------ATGTTCAATCTT---TT
Megaceros aenigmaticus            ---------------------------------ATGTTTGACCATGATTT
Phaeoceros laevis                 ---------------------------------ATGTTTGACCATGATTT
                                                                   *****  * * *   **

Arabidopsis thaliana              TTTAGCGGTTTUCCCAGAGATCTTTATCATTAATGCAACCTUUATTTTGC
Brassica napus                    TTTAGCGGTTTUCCCAGAGATCTTTATCATTAATGCAACCTUUATTTTGC
Lotus japonicus                   TTTAGCGGTTTUCCCAGAGATCTTTCTCATTAATGCAACCTUCATTTTGC
Citrullus lanatus                 TTTAGCGGTTTCCCCAGAGATCTTTATCATTAATGCAACTTTCATTTTGC
Cucurbita pepo                    TTTAGCGGTTTUCCCAGAGATCTTTATCATTAATGCAACTTTCATTTTGC
Beta vulgaris maritima            TTTAGCGGTTTUCCCAGAGATCTTTCTCATTAATGCAACCTTCATTTTGC
Beta vulgaris vulgaris            TTTAGCGGTTTUCCCAGAGATCTTTCTCATTAATGCAACCTTCATTTTGC
Silene latifolia                  TTTAGCGGTTTUCCCAGAGATCTTTATCATTAATGCAACCTTCATTTTGC
BPM24_nad2                        TTTAGCGGTTTUCCCAGAGATCTTTATCATTAATGCAACCTUCATTTTGC
RRII105_nad2                      TTTAGCGGTTTUCCCAGAGATCTTTATCATTAATGCAACCTUCATTTTGC
RRIC110_nad2                      TTTAGCGGTTTUCCCAGAGATCTTTATCATTAATGCAACCTUCATTTTGC
PB235_nad2                        TTTAGCGGTTTUCCCAGAGATCTTTATCATTAATGCAACCTCCATTTTGC
RRIT251_nad2                      TTTAGCGGTTTNCCCAGAGATCTTTATCATTAATGCAACCTNCATTTTGC
RRIM600_nad2                      TTTAGCGGTTTNCCCAGAGATCTTTATCATTAATGCAACCTNCATTTTGC
Megaceros aenigmaticus            CTTAGCACTTTTCCCAGAGATCTTTCTTATTAACGCAACTATCACTTTGC
Phaeoceros laevis                 CTTAGCACTTTTCCTAGAGATCTTTCTTATTAACGCAACTATAACTTCGC
```

```
                                   *****  *** ** ********** * ***** *****    * ** **

Arabidopsis thaliana              TCATTCATGGAGTTGTATTTAGTACCTCTAAGAAATATGATTATCCGCCG
Brassica napus                    TCATTCATGGAGTTGTATTTAGTACCTCTAAGAAATATGATTATCCGCCG
Lotus japonicus                   TCATTCATGGGGTTGTATTTAGTACCTCTAAGAAATATGATTATCCACCG
Citrullus lanatus                 TCATTCATGGAGTTGTATTTAGTACCTCTAAGAAATATGATTATCCACCG
Cucurbita pepo                    TCATTCATGGAGTTGTATTTAGTACCTCTAAGAAATATGATTATCCACCG
Beta vulgaris maritima            TCATTCATGGAGTTATTTTTAGTACCTCTAAGAAATATGATTATCCACCG
Beta vulgaris vulgaris            TCATTCATGGAGTTATTTTTAGTACCTCTAAGAAATATGATTATCCACCG
Silene latifolia                  TCATTCATGGAGTTGTTTTTAGTACCTCTAAGAAATATGATTATCCACCG
BPM24_nad2                        TCATTUATGGAGTTGTATTTAGTACCTCTAAGAAATATGATTATCCGCCG
RRII105_nad2                      TCATTUATGGAGTTGTATTTAGTACCTCTAAGAAATATGATTATCCGCCG
RRIC110_nad2                      TCATTUATGGAGTTGTATTTAGTACCTCTAAGAAATATGATTATCCGCCG
PB235_nad2                        TCATTUATGGAGTTGTATTTAGTACCTCTAAGAAATATGATTATCCGCCG
RRIT251_nad2                      TCATTUATGGAGTTGTATTTAGTACCTCTAAGAAATATGATTATCCGCCG
RRIM600_nad2                      TCATTNATGGAGTTGTATTTAGTACCTCTAAGAAATATGATTATCCGCCG
Megaceros aenigmaticus            TTATTTATGGAGTTGTATTGAGTACCTCCGAAAGATATGATTATCCACCG
Phaeoceros laevis                 CTATTTATGGAGTTGTATTTAGTACCTCCAAAAGATATGATTATCCACCG
                                    *** **** *** * ** ********  * * ************ ***

Arabidopsis thaliana              TTAGCCAGTAATGTGGGTTGGCTTGGATTACTTAGTGTTCTAATAACCTT
Brassica napus                    TTAGCCAGTAATGTGGGTTGGCTTGGATTACTTAGTGTTCTAATAACCTT
Lotus japonicus                   TTAGTCAGTAATGTGGGTTGGCTTGGATTACTTAGTGTTCTAATAACCTT
Citrullus lanatus                 TTAGTCAGTAATGTGGGTTGGCTTGGATTACTTAGTGTTCTAATAACCTT
Cucurbita pepo                    TTAGTCAGTAATGTGGGTTGGCTTGGATTACTTAGTGTTCTAATAACCTT
Beta vulgaris maritima            TTAGTCAGTAATGTGGGTTGGCTTGGATTACTTAGTGTTCTAATAACCTT
Beta vulgaris vulgaris            TTAGTCAGTAATGTGGGTTGGCTTGGATTACTTAGTGTTCTAATAACCTT
Silene latifolia                  TTAGTTAGTAATGTGGGTTGGCTTGGATTACTTAGTGTTCTAATAACCTT
BPM24_nad2                        TTAGTCAGTAATGTGGGTTGGCTTGGATTACTTAGTGTTCTAATAACCTT
RRII105_nad2                      TTAGTCAGTAATGTGGGTTGGCTTGGATTACTTAGTGTTCTAATAACCTT
RRIC110_nad2                      TTAGTCAGTAATGTGGGTTGGCTTGGATTACTTAGTGTTCTAATAACCTT
PB235_nad2                        TTAGTCAGTAATGTGGGTTGGCTTGGATTACTTAGTGTTCTAATAACCTT
RRIT251_nad2                      TTAGTCAGTAATGTGGGTTGGCTTGGATTACTTAGTGTTCTAATAACCTT
RRIM600_nad2                      TTAGTCAGTAATGTGGGTTGGCTTGGATTACTTAGTGTTCTAATAACCTT
Megaceros aenigmaticus            TTAGTTTGTAATGTGGGTTGGCTTGGTTCACCTAGTGTTCTAATAACCAT
Phaeoceros laevis                 TTAGTTTGTAATGTGGGTTGGCTTGGTTTACCTAGTGTTCTAATAACCAT
                                  ****   ******************  * ** **************** *

Arabidopsis thaliana              GCTTCTGCTCGCCGCTGGCGCACCTCTCCTAACTATTGCCCATTTATTCT
Brassica napus                    GCTTCTGCTCGCCGCTGGCGCACCTCTCCTAACTATTGCCCATTTATTCT
Lotus japonicus                   GCTTCTGCTCGCCGCTGGCGCACCTCTCCTAACTATTGCCCATTTATTCC
Citrullus lanatus                 GCTTCTGCTCGCCGCTGGCGCACCTCTCCTAACTATTGCCCATTTATTCT
Cucurbita pepo                    GCTTCTGCTCGCCGCTGGCGCACCTCTCCTAACTATTGCCCATTTATTCT
Beta vulgaris maritima            GCTTTTGCTCGCTGCTGGCGCACCTCTCCTAACTATTGCCCATTTATTCT
Beta vulgaris vulgaris            GCTTTTGCTCGCTGCTGGCGCACCTCTCCTAACTATTGCCCATTTATTCT
Silene latifolia                  GCTTTTGCTCGCTGCTGGCGCACCTCTCCTAACTATTGCCCATTTATTCT
BPM24_nad2                        GCTTCTGCTCGCCGCTGGCGCACCTCTCCTAACTATTGCCCATTTATTCT
RRII105_nad2                      GCTTCTGCTCGCCGCTGGCGCACCTCTCCTAACTATTGCCCATTTATTCT
RRIC110_nad2                      GCTTCTGCTCGCCGCTGGCGCACCTCTCCTAACTATTGCCCATTTATTCT
PB235_nad2                        GCTTCTGCTCGCCGCTGGCGCACCTCTCCTAACTATTGCCCATTTATTCT
RRIT251_nad2                      GCTTCTGCTCGCCGCTGGCGCACCTCTCCTAACTATTGCCCATTTATTCT
RRIM600_nad2                      GCTTCTGCTCGCCGCTGGCGCACCTCTCCTAACTATTGCCCATTTATTCT
Megaceros aenigmaticus            CTTATTGGTTGCCGCTGGCGCACTG---------GTTGCCAATTTATTCT
Phaeoceros laevis                 CTTATTGGTTGCCGCTGGCGCACTG---------GTTGCCAATTTATTCT
                                    *  ** * ** **********            ***** ********

Arabidopsis thaliana              GGAATAATCTTTTTAGGAGGGACAATTTTACATATTTCTGCCAAATCTTT
Brassica napus                    GGAATAATCTTTTTAGGAGGGACAATTTTACATATTTUTGCCAAATCTTT
Lotus japonicus                   GGAATAATCTTTTTAGGAGGGACAATTTTACATATTTCTGCCAAATCCTT
Citrullus lanatus                 GGAATAATCTTTTTAGGAGGGACAATTTTACATATTTUTGCCAAATCCTT
Cucurbita pepo                    GGAATAATCTTTTTAGGAGGGACAATTTGACATATTTCTGCCAAATCCTT
Beta vulgaris maritima            GGAATAATUTTTTTAAGAGGGACAATTTTACATATTTCTGCCAAATCCTT
```

```
Beta vulgaris vulgaris            GGAATAATUTTTTTAAGAGGGACAATTTTACATATTTCTGCCAAATCCTT
Silene latifolia                  GGAATAATTTTTTTAAGAGGGACAATTTTACATATTTCTGCCAAATCTTT
BPM24_nad2                        GGAATAATCTTTTTAGGAGGGACAATTTTACATATTTCTGCCAAATCCTT
RRII105_nad2                      GGAATAATCTTTTTAGGAGGGACAATTTTACATATTTCTGCCAAATCCTT
RRIC110_nad2                      GGAATAATCTTTTTAGGAGGGACAATTTTACATATTTCTGCCAAATCCTT
PB235_nad2                        GGAATAATCTTTTTAGGAGGGACAATTTTACATATTTCTGCCAAATCCTT
RRIT251_nad2                      GGAATAATCTTTTTAGGAGGGACAATTTTACATATTTCTGCCAAATCCTT
RRIM600_nad2                      GGAATAATCTTTTTAGGAGGGACAATTTTACATATTTCTGCCAAATCCTT
Megaceros aenigmaticus            ATAATAA---TTTGATAATAGACAATTTTACATATTTTUGCCAAATTTTT
Phaeoceros laevis                 ATAATAA---TTTAATAATAGACAATTTTACATATTTTCGCCAAATTTTT
                                    *****   *** *  *  ******** ********  *******  **

Arabidopsis thaliana              CTATTATTAAGTACGGCTGGTACCATTTCGATGTGTTTCGATTUTTUCGA
Brassica napus                    CTATTATTAAGTACGGCTGGTACCATTTCGATGTGTTTCGATTUTTUCGA
Lotus japonicus                   CTATTATTAAGTACGGCTGGTACCATTTCGATGTGTTTCGATTUTTUCGA
Citrullus lanatus                 CTATTATTAAGTACGGCTGGTACCATTTCGATGTGTTTCGATTUTTTCGA
Cucurbita pepo                    CTATTATTAAGTACGGCTGGTACCATTTCGATGTGTTTUGATTUTTTCGA
Beta vulgaris maritima            CTATTATTAAGTACGGCTGGTACCATTTCGATGTGTTTTGATTTTTTCGA
Beta vulgaris vulgaris            CTATTATTAAGTACGGCTGGTACCATTTCGATGTGTTTTGATTTTTTCGA
Silene latifolia                  CTATTATTAAGTACGGCTGGTACCATTTCGATGTGTTTTGATTTTTTCGA
BPM24_nad2                        CTATTATTAAGTACGGCTGGTACCATTTCGATGTGTTTCGATTCTTCCGA
RRII105_nad2                      CTATTATTAAGTACGGCTGGTACCATTTCGATGTGTTTCGATTCTTCCGA
RRIC110_nad2                      CTATTATTAAGTACGGCTGGTACCATTTCGATGTGTTTCGATTCTTCCGA
PB235_nad2                        CTATTATTAAGTACGGCTGGTACCATTTCGATGTGTTTCGATTCTTCCGA
RRIT251_nad2                      CTATTATTAAGTACGGCTGGTACCATTTCGATGTGTTTCGATTCTTCCGA
RRIM600_nad2                      CTATTATTAAGTACGGCTGGTACCATTTCGATGTGTTTCGATTCTTCCGA
Megaceros aenigmaticus            CCATCATTAAGTACGGCTAGTACTATTCTTATGTGTTTGGATTATTTCAA
Phaeoceros laevis                 CCATCATTAAGTACGGCTAGTACTATTCCTACGTGTTTGGATTATTTCAA
                                  * ** ************* **** ***   * ****** **** ** * *

Arabidopsis thaliana              CCAAGAGAGGTTTGATGCTTTTGAATTCATTGTATTAATTCUACTTUCTA
Brassica napus                    CCAAGAGAGGTTTGATGCTTTTGAATTCATTGTATTAATTCUACTTUCTA
Lotus japonicus                   ACAAGAGAGGTTTGATGCTTTTGAATTCATTGTATTAATTCUACTTUCTA
Citrullus lanatus                 ACAAGAGAGGTTTGATGCTTTTGAATTCATTGTATTAATTCUACTTUCTA
Cucurbita pepo                    ACAAGAGAGGTTTGATGCTTTTGAATTCATTGTATTAATTCUACTTUCTA
Beta vulgaris maritima            ACAAGAGAGGTTTGATGCTTTTGAATTCATTGTATTAATTTUACTTUCTA
Beta vulgaris vulgaris            ACAAGAGAGGTTTGATGCTTTTGAATTCATTGTATTAATTTUACTTUCTA
Silene latifolia                  ACAAGAGAGGTTTGATGCTTTTGAATTCATTGTATTAATTTUACTTUCTA
BPM24_nad2                        ACAAGAGAGGTTTGATGCTTTTGAATTCAUTGTATTAATTCTACTTCCTA
RRII105_nad2                      ACAAGAGAGGTTTGATGCTTTTGAATTCAUTGTATTAATTCTACTTCCTA
RRIC110_nad2                      ACAAGAGAGGTTTGATGCTTTTGAATTCAUTGTATTAATTCTACTTCCTA
PB235_nad2                        ACAAGAGAGGTTTGATGCTTTTGAATTCAUTGTATTAATTCTACTTCCTA
RRIT251_nad2                      ACAAGAGAGGTTTGATGCTTTTGAATTCAUTGTATTAATTCTACTTCCTA
RRIM600_nad2                      ACAAGAGAGGTTTGATGCTTTTGAATTCANTGTATTAATTCTACTTCCTA
Megaceros aenigmaticus            AGAAGAGAGTTTGAATGCTTTTGAATTTATTATATTAATTCTACCTCCTA
Phaeoceros laevis                 AGAAGAGAGTTTGAATGCTCCTGAATTTATTATATTAATTTTACTTTCTA
                                    ******* **  *****  ****** * * ********* ** * ***

Arabidopsis thaliana              CTUGCGGTATGCTCTTTATGATCTCGGCTUATGATTTAATTGCUATGTAT
Brassica napus                    CTTGCGGTATGCTCTTTATGATCTCGGCTUATGATTTAATTGCCATGTAT
Lotus japonicus                   CTUGCAGTATGCTCTTTATGATCTCGGCTUATGATTUAATTGCCATGTAT
Citrullus lanatus                 CTUGCAGTATGCTCTTTATGATCTCGGCTUATGATTUAATTTCCATGTAT
Cucurbita pepo                    CTUGCAGTATGCTCTTTATGATCTCGGCTUATGATTUAATTTCCATGTAT
Beta vulgaris maritima            CTUGCAGTATGCTCTTTATGATCTCGGCTUATGATCTAATTGCCATGTAT
Beta vulgaris vulgaris            CTUGCAGTATGCTCTTTATGATCTCGGCTUATGATCTAATTGCCATGTAT
Silene latifolia                  CTUGCAGTATGCTCTTTATGATCTCGGCTUATGATTTAATTGCCATGTAT
BPM24_nad2                        CTCGCAGTATGCTCTTTATGATCTCGGCTCATGATTCAATTGCCATGTAT
RRII105_nad2                      CTCGCAGTATGCTCTTTATGATCTCGGCTCATGATTCAATTGCCATGTAT
RRIC110_nad2                      CTCGCAGTATGCTCTTTATGATCTCGGCTCATGATTCAATTGCCATGTAT
PB235_nad2                        CTCGCAGTATGCTCTTTATGATCTCGGCTCATGATTCAATTGCCATGTAT
RRIT251_nad2                      CTCGCAGTATGCTCTTTATGATCTCGGCTCATGATTCAATTGCCATGTAT
RRIM600_nad2                      CTCGCAGTATGCTCTTTATGATCTCGGCTCATGATTCAATTGCCATGTAT
```

```
Megaceros aenigmaticus            TCCGCAGTATGCCTCTTACGATTTTGGCTTACGATTCAATTGCGATGTAT
Phaeoceros laevis                 TTTGCAGTATGTTTTTTATGATTTTGGCTTACGATTTAATTGCGACGTAT
                                     ** *****    *** *** * **** * ***  **** * * ****

Arabidopsis thaliana              TTAGCTATTGAGCUTCAAAGTTTATGTTTTTATGTAATCGCAGCATCAAA
Brassica napus                    TTAGCTATTGAGCUTCAAAGTTTATGTTTTTATGTAATCGCAGCATCAAA
Lotus japonicus                   TTAGCTATTGAGCUTCAAAGTTTATGTTTTTATGTGATGGCAGCATCAAA
Citrullus lanatus                 TTAGCTATTGAGCUTCAAAGTTTATGTTTTTATGTGATGGCAGCATCAAA
Cucurbita pepo                    TTAGCTATTGAGCUTCAAAGTTTATGTTTTTATGTGATGGCAGCATCCAA
Beta vulgaris maritima            TTAGCTATTGAGCUTCAAAGTTTATGTTTTTATGTGATCGCAGCATCAAA
Beta vulgaris vulgaris            TTAGCTATTGAGCUTCAAAGTTTATGTTTTTATGTGATCGCAGCATCAAA
Silene latifolia                  TTAGCTATTGAGCUTCAAAGTTTATGTTTTTATGTGATCGCAGCATCAAA
BPM24_nad2                        TTAGCTATTGAGCCTCAAAGTTTATGTTTTTATGTGATCGCAGCATCAAA
RRII105_nad2                      TTAGCTATTGAGCCTCAAAGTTTATGTTTTTATGTGATCGCAGCATCAAA
RRIC110_nad2                      TTAGCTATTGAGCCTCAAAGTTTATGTTTTTATGTGATCGCAGCATCAAA
PB235_nad2                        TTAGCTATTGAGCCTCAAAGTTTATGTTTTTATGTGATCGCAGCATCAAA
RRIT251_nad2                      TTAGCTATTGAGCCTCAAAGTTTATGTTTTTATGTGATCGCAGCATCAAA
RRIM600_nad2                      TTAGCTATTGAGCCTCAAAGTTTATGTTTTTATGTGATCGCAGCATCAAA
Megaceros aenigmaticus            TCAGCTATTGAGCCTCAAAGTTTATGTTTCTATGTGATCGCAGCATCCAA
Phaeoceros laevis                 TCAGCTATTGAGCCTCAAAGTTTATGTTTCTATGTGATTGCAGCATCTAA
                                  * *********** *************** ***** ** ******** **

Arabidopsis thaliana              AAGAAAGTCTGAATTTTCCACGGAAGCCGGUTUGAAATATTTGATCTTAG
Brassica napus                    AAGAAAGTCTGAATTTTCCACGGAAGCCGGCTTGAAATATTTGATCTTAG
Lotus japonicus                   AAGAAAGTCTGAATTTTCCACGGAAGCCGGCTUGAAATATTTGATCTTAG
Citrullus lanatus                 AAGAAAGTCTGAATTTTCCACGGAAGCCGGCTUGAAATATTTGATCTTAG
Cucurbita pepo                    AAGAAAGTCTGAATTTTCCACGGAAGCCGGCTUGAAATATTTGATCTTAG
Beta vulgaris maritima            AAGAAAGTCTGAATTTTCTACGGAAGCCGGCTCGAAATATTTGATCTTAG
Beta vulgaris vulgaris            AAGAAAGTCTGAATTTTCTACGGAAGCCGGCTUGAAATATTTGATCTTAG
Silene latifolia                  ACGAAAGTCTGAATTTTCTACGGAAGCCGGCTUGAAATATTTGATCTTAG
BPM24_nad2                        AAGAAAGTCTGAATTTTCCACGGAAGCCGGCTCGAAATATTTGATCTTAG
RRII105_nad2                      AAGAAAGTCTGAATTTTCCACGGAAGCCGGCTCGAAATATTTGATCTTAG
RRIC110_nad2                      AAGAAAGTCTGAATTTTCCACGGAAGCCGGCTCGAAATATTTGATCTTAG
PB235_nad2                        AAGAAAGTCTGAATTTTCCACGGAAGCCGGCTCGAAATATTTGATCTTAG
RRIT251_nad2                      AAGAAAGTCTGAATTTTCCACGGAAGCCGGCTCGAAATATTTGATCTTAG
RRIM600_nad2                      AAGAAAGTCTGAATTTTCCACGGAAGCCGGCTCGAAATATTTGATCTTAG
Megaceros aenigmaticus            AAGAGACTCTGAATTTTCCACAGAAGCCGGCTTAAAATATTTTATTTTAG
Phaeoceros laevis                 AAGAGACTCTGAATTTTCCACAGAAGCCGGCTTAAAATATTTTATTTTAG
                                  * ** * *********** ** ******** *  ******** ** ****

Arabidopsis thaliana              GTGCATTTTCUTCTGGAATATTATTGTTTGG-------------------
Brassica napus                    GTGCATTTTCCTCTGGAATATTATTGTTTGG-------------------
Lotus japonicus                   GTGCATTTTCCTCTGGAATCTTACTGTTTGG-------------------
Citrullus lanatus                 GTGCATTTTCCTCTGGAATCTTACTGTTTGG-------------------
Cucurbita pepo                    GTGCATTTTCCTCTGGAATCTTACTGTTTGG-------------------
Beta vulgaris maritima            GTGCATTTTCTTCTGGAATATTATTGTTTGG-------------------
Beta vulgaris vulgaris            GTGCATTTTCTTCTGGAATATTATTGTTTGG-------------------
Silene latifolia                  GTGCATTTTCTTCTGGAATATTATTGTTTGG-------------------
BPM24_nad2                        GTGCATTTCCCTCTGGAATATTATTGTTTGGTTGTTCCATGATCTATGGG
RRII105_nad2                      GTGCATTTCCCTCTGGAATATTATTGTTTGGTTGTTCCATGATCTATGGG
RRIC110_nad2                      GTGCATTTCCCTCTGGAATATTATTGTTTGGTTGTTCCATGATCTATGGG
PB235_nad2                        GTGCATTTCCCTCTGGAATATTATTGTTTGGTTGTTCCATGATCTATGGG
RRIT251_nad2                      GTGCATTTCCCTCTGGAATATTATTGTTTGGTTGTTCCATGATCTATGGG
RRIM600_nad2                      GTGCATTTCCCTCTGGAATATTATTGTTTGGTTGTTCCATGATCTATGGG
Megaceros aenigmaticus            GTGCATTTTCATCTGGAATATTGTTGTTTGGTTGTTCCATGATTCATGGG
Phaeoceros laevis                 GTGCATTTCCATCTGGAATATTGTTGTTTGGTTGTTTCATGATTTATGGG
                                  ******** * ******** **  *******

Arabidopsis thaliana              --------------------------------------------------
Brassica napus                    --------------------------------------------------
Lotus japonicus                   --------------------------------------------------
Citrullus lanatus                 --------------------------------------------------
```

```
Cucurbita pepo                  --------------------------------------------------
Beta vulgaris maritima          --------------------------------------------------
Beta vulgaris vulgaris          --------------------------------------------------
Silene latifolia                --------------------------------------------------
BPM24_nad2                      TCTACAGGAGCTACCCACTTCGATCAATTAGCCAAGATTTTGACCGGATA
RRII105_nad2                    TCTACAGGAGCTACCCACTTCGATCAATTAGCCAAGATTTTGACCGGATA
RRIC110_nad2                    TCTACAGGAGCTACCCACTTCGATCAATTAGCCAAGATTTTGACUGGATA
PB235_nad2                      TCTACAGGAGCTACCCACTTCGATCAATTAGCCAAGATTTTGACUGGATA
RRIT251_nad2                    TCTACAGGAGCTACCCACTTCGATCAATTAGCCAAGATTTTGACCGGATA
RRIM600_nad2                    TCTACAGGAGCTACCCACTTCGATCAATTAGCCAAGATTTTGACNGGATA
Megaceros aenigmaticus          TTTACTGGAGTTACCAACTTTGAGGAATTAGCCAAGATCTTTACTGGATA
Phaeoceros laevis               TTTACTGGAGTTACCAACTTTGAGGAATTAGCCAAGATCTTTACTGGATA


Arabidopsis thaliana            --------------------------------------------------
Brassica napus                  --------------------------------------------------
Lotus japonicus                 --------------------------------------------------
Citrullus lanatus               --------------------------------------------------
Cucurbita pepo                  --------------------------------------------------
Beta vulgaris maritima          --------------------------------------------------
Beta vulgaris vulgaris          --------------------------------------------------
Silene latifolia                --------------------------------------------------
BPM24_nad2                      CGAAATCACT------GGTGCTCGATCTAGTGGTATTTTTATGGGGATTC
RRII105_nad2                    CGAAATCACT------GGTGCTCGATCTAGTGGTATTTTTATGGGGATTC
RRIC110_nad2                    CGAAATCACT------GGTGCTCGATCTAGTGGTATTTTTATGGGGATTC
PB235_nad2                      CGAAATCACT------GGTGCTCGATCTAGTGGTATTTTTATGGGGATTC
RRIT251_nad2                    CGAAATCACT------GGTGCTCGATCTAGTGGTATTTTTATGGGGATTC
RRIM600_nad2                    CGAAATCACT------GGTGCTCGATCTAGTGGTATTTTTATGGGGATTC
Megaceros aenigmaticus          TGAAATCACTTTATTCGGTGCTCAATCTAGTGGGATCTTTATGGGGATTC
Phaeoceros laevis               TGAAATCACTTTGTTCGGTGCTCAATCTAGTGGGATCTTTATGGGGATTC


Arabidopsis thaliana            --------------------------------------------------
Brassica napus                  --------------------------------------------------
Lotus japonicus                 --------------------------------------------------
Citrullus lanatus               --------------------------------------------------
Cucurbita pepo                  --------------------------------------------------
Beta vulgaris maritima          --------------------------------------------------
Beta vulgaris vulgaris          --------------------------------------------------
Silene latifolia                --------------------------------------------------
BPM24_nad2                      TATCTATCGCTGTAGGATTCCTATTCAAGATCACTGCAGTTCCTTTTCAT
RRII105_nad2                    TATUTATCGCTGTAGGATTCCTATTCAAGATCACTGCAGTTCCTTTTCAT
RRIC110_nad2                    TATUTATCGCTGTAGGATTCCTATTCAAGATCACTGCAGTTCCTTTTCAT
PB235_nad2                      TATUTATCGCTGTAGGATTCCTATTCAAGATCACTGCAGTTCCTTTTCAT
RRIT251_nad2                    TATCTATCGCTGTAGGATTCCTATTCAAGATCACTGCAGTTCCTTTTCAT
RRIM600_nad2                    TATNTATCGCTGTAGGATTCCTATTCAAGATCACTGCAGTTCCTTTTCAT
Megaceros aenigmaticus          TATTTATCGCCGTAGGTTTTTTATTTAAAATAACTGCAGTTCCTTTTCAT
Phaeoceros laevis               CATTTATCGCCGTAGGTTTTTTATTTAAAATAACTGCAGTTTCTTTTCAT


Arabidopsis thaliana            --------------------------------------------------
Brassica napus                  --------------------------------------------------
Lotus japonicus                 --------------------------------------------------
Citrullus lanatus               --------------------------------------------------
Cucurbita pepo                  --------------------------------------------------
Beta vulgaris maritima          --------------------------------------------------
Beta vulgaris vulgaris          --------------------------------------------------
Silene latifolia                --------------------------------------------------
BPM24_nad2                      ATGTGGGCACCTGATATCTATGAGGGTTCACCCACCCCCGTTACAGCATT
RRII105_nad2                    ATGTGGGCACCTGATATCTATGAGGGTTCACCCACCCCCGTTACAGCATT
RRIC110_nad2                    ATGTGGGCACCTGATATCTATGAGGGTTCACCCACCCCCGTTACAGCATT
PB235_nad2                      ATGTGGGCACCTGATATCTATGAGGGTTCACCCACCCCCGTTACAGCATT
```

```
RRIT251_nad2                      ATGTGGGCACCTGATATCTATGAGGGTTCACCCACCCCCGTTACAGCATT
RRIM600_nad2                      ATGTGGGCACCTGATATCTATGAGGGTTCACCCACCCCCGTTACAGCATT
Megaceros aenigmaticus            ATGTGGGCGCCTGATGTATACGAGGGTTCACCTACCCTGGTTACAGCATT
Phaeoceros laevis                 ATGTGGGCGCCTGATGTATACGAGGGTTCACCTACCCTGGTTACAGCATT


Arabidopsis thaliana              --------------------------------------------------
Brassica napus                    --------------------------------------------------
Lotus japonicus                   --------------------------------------------------
Citrullus lanatus                 --------------------------------------------------
Cucurbita pepo                    --------------------------------------------------
Beta vulgaris maritima            --------------------------------------------------
Beta vulgaris vulgaris            --------------------------------------------------
Silene latifolia                  --------------------------------------------------
BPM24_nad2                        CCTTTCTATTGCGCCTAAAATCTCTATTTTTGCTAATATTTUACGTGTTT
RRII105_nad2                      CCTTTCTATTGCGCCTAAAATCTCTATTTTTGCTAATATTTUACGTGTTT
RRIC110_nad2                      CCTTTCTATTGCGCCTAAAATCTCTATTTTTGCTAATATTTUACGTGTTT
PB235_nad2                        CCTTTCTATTGCGCCTAAAATCTCTATTTTTGCTAATATTTUACGTGTTT
RRIT251_nad2                      CCTTTCTATTGCGCCTAAAATCTCTATTTTTGCTAATATTTUACGTGTTT
RRIM600_nad2                      CCTTTCTATTGCGCCTAAAATCTCTATTTTTGCTAATATTTNACGTGTTT
Megaceros aenigmaticus            TTTCCCCATTGCACCTAAAATATCTATTCTTGCCAATATGTTACGTGTCT
Phaeoceros laevis                 TTTTTCCATTGCACCTAAAATCTCTATTCTTGCCAATATGTTACGTGTGT


Arabidopsis thaliana              --------------------------------------------------
Brassica napus                    --------------------------------------------------
Lotus japonicus                   --------------------------------------------------
Citrullus lanatus                 --------------------------------------------------
Cucurbita pepo                    --------------------------------------------------
Beta vulgaris maritima            --------------------------------------------------
Beta vulgaris vulgaris            --------------------------------------------------
Silene latifolia                  --------------------------------------------------
BPM24_nad2                        UTATTTATGGTTCCTATGGAGCTACATTGCAACAAATCTTCTTTTTCTGC
RRII105_nad2                      UTATTTATGGTTCCTATGGAGCTACATTGCAACAAATCTTCTTTTTCTGC
RRIC110_nad2                      UTATTTATGGTTCCTATGGAGCTACATTGCAACAAATCTTCTTTTTCTGC
PB235_nad2                        UTATTTATGGTTCCTATGGAGCTACATTGCAACAAATCTTCTTTTTCTGC
RRIT251_nad2                      UTATTTATGGTTCCTATGGAGCTACATTGCAACAAATCTTCTTTTTCTGC
RRIM600_nad2                      NTATTTATGGTTCCTATGGAGCTACATTGCAACAAATCTTCTTTTTCTGC
Megaceros aenigmaticus            TTATTTATAGTTTCTATGATCCGACATGGCAACAACTATTCTTTTTCTGC
Phaeoceros laevis                 TTATTTATAGTTTCTATGATCCGACATGGCAACAACTATTCTTCTTCTGC


Arabidopsis thaliana              --------------------------------------------------
Brassica napus                    --------------------------------------------------
Lotus japonicus                   --------------------------------------------------
Citrullus lanatus                 --------------------------------------------------
Cucurbita pepo                    --------------------------------------------------
Beta vulgaris maritima            --------------------------------------------------
Beta vulgaris vulgaris            --------------------------------------------------
Silene latifolia                  --------------------------------------------------
BPM24_nad2                        AGCATTGCTTCTATGATCTTAGGAGCACTGGCCGCCATGGCCCAAACGAA
RRII105_nad2                      AGCATTGCTTCTATGATCTTAGGAGCACTGGCCGCCATGGCCCAAACGAA
RRIC110_nad2                      AGCATTGCTTCTATGATCTTAGGAGCACTGGCCGCCATGGCCCAAACGAA
PB235_nad2                        AGCATTGCTTCTATGATCTTAGGAGCACTGGCCGCCATGGCCCAAACGAA
RRIT251_nad2                      AGCATTGCTTCTATGATCTTAGGAGCACTGGCCGCCATGGCCCAAACGAA
RRIM600_nad2                      AGCATTGCTTCTATGATCTTAGGAGCACTGGCCGCCATGGCCCAAACGAA
Megaceros aenigmaticus            AGCATTGCTTCTATGATTTTAGGAGCACTGGCTGCCATGGCCCAAAACAA
Phaeoceros laevis                 AGCATCGCTTTTATGATTTTAGGAGCACTGGCTGCCATGGCCCAAAACAA


Arabidopsis thaliana              --------------------------------------------------
Brassica napus                    --------------------------------------------------
```

```
Lotus japonicus                  --------------------------------------------------
Citrullus lanatus                --------------------------------------------------
Cucurbita pepo                   --------------------------------------------------
Beta vulgaris maritima           --------------------------------------------------
Beta vulgaris vulgaris           --------------------------------------------------
Silene latifolia                 --------------------------------------------------
BPM24_nad2                       AGTAAAAAGACUTCTAGCTUATAGTTCAATTGGACATGTAGGTTATATTC
RRII105_nad2                     AGTAAAAAGACUTCTAGCTUATAGTTCAATTGGACATGTAGGTTATATTC
RRIC110_nad2                     AGTAAAAAGACUTCTAGCTUATAGTTCAATTGGACATGTAGGTTATATTC
PB235_nad2                       AGTAAAAAGACNTCTAGCTNATAGTTCAATTGGACATGTAGGTTATATTC
RRIT251_nad2                     AGTAAAAAGACNTCTAGCTNATAGTTCAATTGGACATGTAGGTTATATTC
RRIM600_nad2                     AGTAAAAAGACNTCTAGCTNATAGTTCAATTGGACATGTAGGTTATATTC
Megaceros aenigmaticus           AGTAAAAAGACTTTTAGCTTATAGTTCTATTGGACACGTAGGTTATCTTC
Phaeoceros laevis                AGTAAAAAGACTTTTAGCTTATAGTTCTATTGGACACGTAGGTTATCTTC


Arabidopsis thaliana             --------------------------------------------------
Brassica napus                   --------------------------------------------------
Lotus japonicus                  --------------------------------------------------
Citrullus lanatus                --------------------------------------------------
Cucurbita pepo                   --------------------------------------------------
Beta vulgaris maritima           --------------------------------------------------
Beta vulgaris vulgaris           --------------------------------------------------
Silene latifolia                 --------------------------------------------------
BPM24_nad2                       GTACTGGGTTCTCATGTGGAACCATAGAAGGAATTCAATCACTACTAATT
RRII105_nad2                     GTACTGGGTTCTCATGTGGAACCATAGAAGGAATTCAATCACTACTAATT
RRIC110_nad2                     GTACTGGGTTCTCATGTGGAACCATAGAAGGAATTCAATCACTACTAATT
PB235_nad2                       GTACTGGGTTCTCATGTGGAACCATAGAAGGAATTCAATCACTACTAATT
RRIT251_nad2                     GTACTGGGTTCTCATGTGGAACCATAGAAGGAATTCAATCACTACTAATT
RRIM600_nad2                     GTACTGGGTTCTCATGTGGAACCATAGAAGGAATTCAATCACTACTAATT
Megaceros aenigmaticus           CAATTGGTTTTTCATGTGGAACCATAGAAGGGATTCAATCACTATTAATT
Phaeoceros laevis                CAATTGGTTTTTCACGTGGAACTATAGAAGGGATTCAATCACTATTAATT


Arabidopsis thaliana             --------------------------------------------------
Brassica napus                   --------------------------------------------------
Lotus japonicus                  --------------------------------------------------
Citrullus lanatus                --------------------------------------------------
Cucurbita pepo                   --------------------------------------------------
Beta vulgaris maritima           --------------------------------------------------
Beta vulgaris vulgaris           --------------------------------------------------
Silene latifolia                 --------------------------------------------------
BPM24_nad2                       GGTATCTTTATTTATGCATTAATGACGATAGATGCATTCGCCATAGTTTT
RRII105_nad2                     GGTATCTTTATTTATGCATTAATGACGATAGATGCATTCGCCATAGTTTT
RRIC110_nad2                     GGTATCTTTATTTATGCATTAATGACGATAGATGCATTCGCCATAGTTTT
PB235_nad2                       GGTATCTTTATTTATGCATTAATGACGATAGATGCATTCGCCATAGTTTT
RRIT251_nad2                     GGTATCTTTATTTATGCATTAATGACGATAGATGCATTCGCCATAGTTTT
RRIM600_nad2                     GGTATCTTTATTTATGCATTAATGACGATAGATGCATTCGCCATAGTTTT
Megaceros aenigmaticus           GCTATGTTTATTTATGTATTAATGACTATCAATGTATTCGTCATAGTTTT
Phaeoceros laevis                GCTATGTTTATTTATGTATTAATGACTATCAATGTATCCGTCACAGTTTT


Arabidopsis thaliana             --------------------------------------------------
Brassica napus                   --------------------------------------------------
Lotus japonicus                  --------------------------------------------------
Citrullus lanatus                --------------------------------------------------
Cucurbita pepo                   --------------------------------------------------
Beta vulgaris maritima           --------------------------------------------------
Beta vulgaris vulgaris           --------------------------------------------------
Silene latifolia                 --------------------------------------------------
BPM24_nad2                       AGCATTACGGCAAACCCGTGTCAAATATATAGCGGATTTGGGCGCTCTAG
RRII105_nad2                     AGCATTACGGCAAACCCGTGTCAAATATATAGCGGATTTGGGCGCTCTAG
```

```
RRIC110_nad2                  AGCATTACGGCAAACCCGTGTCAAATATATAGCGGATTTGGGCGCTCTAG
PB235_nad2                    AGCATTACGGCAAACCCGTGTCAAATATATAGCGGATTTGGGCGCTCTAG
RRIT251_nad2                  AGCATTACGGCAAACCCGTGTCAAATATATAGCGGATTTGGGCGCTCTAG
RRIM600_nad2                  AGCATTACGGCAAACCCGTGTCAAATATATAGCGGATTTGGGCGCTCTAG
Megaceros aenigmaticus        AGCACTACGTCAAAACCGCCTCAAATATATAGCGGATTTAGGTGCTTTAG
Phaeoceros laevis             AGCATTACGTAAAAACCGCCTCAAATATATAGCGGATTTAGGTGCTTTAG


Arabidopsis thaliana          --------------------------------------------------
Brassica napus                --------------------------------------------------
Lotus japonicus               --------------------------------------------------
Citrullus lanatus             --------------------------------------------------
Cucurbita pepo                --------------------------------------------------
Beta vulgaris maritima        --------------------------------------------------
Beta vulgaris vulgaris        --------------------------------------------------
Silene latifolia              --------------------------------------------------
BPM24_nad2                    CCAAAACGAATCCTATTTUGGCTATTACCTTCTCCATTACTATGTTCTCA
RRII105_nad2                  CCAAAACGAATCCTATTTUGGCTATTACCTTCTCCATTACTATGTTCTCA
RRIC110_nad2                  CCAAAACGAATCCTATTTUGGCTATTACCTTCTCCATTACTATGTTCTCA
PB235_nad2                    CCAAAACGAATCCTATTTUGGCTATTACCTTCTCCATTACTATGTTCTCA
RRIT251_nad2                  CCAAAACGAATCCTATTTUGGCTATTACCTTCTCCATTACTATGTTCTCA
RRIM600_nad2                  CCAAAACGAATCCTATTTNGGCTATTACCTTCTCCATTACTATGTTCTCA
Megaceros aenigmaticus        CCAAAACTAATCGTATTTTAGCAATTACCTTGCCTATTACTATGTTTTCA
Phaeoceros laevis             CCAAAACTAATCGTATTTTAGCAATTACCTTGTCTATTACTATGTCTCCA


Arabidopsis thaliana          --------------------------------------------------
Brassica napus                --------------------------------------------------
Lotus japonicus               --------------------------------------------------
Citrullus lanatus             --------------------------------------------------
Cucurbita pepo                --------------------------------------------------
Beta vulgaris maritima        --------------------------------------------------
Beta vulgaris vulgaris        --------------------------------------------------
Silene latifolia              --------------------------------------------------
BPM24_nad2                    TACGCAGGAATACCCCCGTTAGCCGGCTTTTGTAGCAAATTCTATTTGTT
RRII105_nad2                  TACGCAGGAATACCCCCGTTAGCCGGCTTTTGTAGCAAATTCTATTTGTT
RRIC110_nad2                  TACGCAGGAATACCCCCGTTAGCCGGCTTTTGTAGCAAATTCTATTTGTT
PB235_nad2                    TACGCAGGAATACCCCCGTTAGCCGGCTTTTGTAGCAAATTCTATTTGTT
RRIT251_nad2                  TACGCAGGAATACCCCCGTTAGCCGGCTTTTGTAGCAAATTCTATTTGTT
RRIM600_nad2                  TACGCAGGAATACCCCCGTTAGCCGGCTTTTGTAGCAAATTCTATTTGTT
Megaceros aenigmaticus        TACGCAGGAATACCTCCGTTAGCTGGATTTTGTAGCAAATTCTATTTATT
Phaeoceros laevis             TACGCAGGAATACTTTTGTTAGCTGGATTTTGTAGCAAATTCTATTTATT


Arabidopsis thaliana          --------------------------------------------------
Brassica napus                --------------------------------------------------
Lotus japonicus               --------------------------------------------------
Citrullus lanatus             --------------------------------------------------
Cucurbita pepo                --------------------------------------------------
Beta vulgaris maritima        --------------------------------------------------
Beta vulgaris vulgaris        --------------------------------------------------
Silene latifolia              --------------------------------------------------
BPM24_nad2                    CTTCGCCGCTTTGGGTTGTGGGGCTTACTTCCTAGCCCCAGTGGGAGTAG
RRII105_nad2                  CTTCGCCGCTTTGGGTTGTGGGGCTTACTTCCTAGCCCCAGTGGGAGTAG
RRIC110_nad2                  CTTCGCCGCTTTGGGTTGTGGGGCTTACTTCCTAGCCCCAGTGGGAGTAG
PB235_nad2                    CTTCGCCGCTTTGGGTTGTGGGGCTTACTTCCTAGCCCCAGTGGGAGTAG
RRIT251_nad2                  CTTCGCCGCTTTGGGTTGTGGGGCTTACTTCCTAGCCCCAGTGGGAGTAG
RRIM600_nad2                  CTTCGCCGCTTTGGGTTGTGGGGCTTACTTCCTAGCCCCAGTGGGAGTAG
Megaceros aenigmaticus        TTCCGCCGCTCCAGGCTGTGGAGCTTACTCACTAGCTTTTGTAGGAATAG
Phaeoceros laevis             TTTCGCTGCTCTAGGCTGTGGGGCTTACTCACTAGCTTTTGTAGGAATAG
```

```
Arabidopsis thaliana          --------------------------------------------------
Brassica napus                --------------------------------------------------
Lotus japonicus               --------------------------------------------------
Citrullus lanatus             --------------------------------------------------
Cucurbita pepo                --------------------------------------------------
Beta vulgaris maritima        --------------------------------------------------
Beta vulgaris vulgaris        --------------------------------------------------
Silene latifolia              --------------------------------------------------
BPM24_nad2                    TGACTAGCGTTATAGGTCGTTTTTATTATATACGCTTAGCGAAAAGAATG
RRII105_nad2                  TGACTAGCGTTATAGGTCGTTTTTATTATATACGCTTAGCGAAAAGAATG
RRIC110_nad2                  TGACTAGCGTTATAGGTCGTTTTTATTATATACGCTTAGCGAAAAGAATG
PB235_nad2                    TGACTAGCGTTATAGGTCGTTTTTATTATATACGCTTAGCGAAAAGAATG
RRIT251_nad2                  TGACTAGCGTTATAGGTCGTTTTTATTATATACGCTTAGCGAAAAGAATG
RRIM600_nad2                  TGACTAGCGTTATAGGTCGTTTTTATTATATACGCTTAGCGAAAAGAATG
Megaceros aenigmaticus        TTACTAGCGTTATCAGTTGTTTTCATTACATACGCTTCGTGAAAATAATG
Phaeoceros laevis             TTACTAGCGTTACCAGTCGTTTTTATTATATACGCTTTGTGAAAATAATG

Arabidopsis thaliana          --------------------------------------------------
Brassica napus                --------------------------------------------------
Lotus japonicus               --------------------------------------------------
Citrullus lanatus             --------------------------------------------------
Cucurbita pepo                --------------------------------------------------
Beta vulgaris maritima        --------------------------------------------------
Beta vulgaris vulgaris        --------------------------------------------------
Silene latifolia              --------------------------------------------------
BPM24_nad2                    TTTTTTGATACACCTAGGACATGGATTCTATATGAACCAATGGATCGTAA
RRII105_nad2                  TTTTTTGATACACCTAGGACATGGATTCTATATGAACCAATGGATCGTAA
RRIC110_nad2                  TTTTTTGATACACCTAGGACATGGATTCTATATGAACCAATGGATCGTAA
PB235_nad2                    TTTTTTGATACACCTAGGACATGGATTCTATATGAACCAATGGATCGTAA
RRIT251_nad2                  TTTTTTGATACACCTAGGACATGGATTCTATATGAACCAATGGATCGTAA
RRIM600_nad2                  TTTTTTGATACACCTAGGACATGGATTCTATATGAACCAATGGATCGTAA
Megaceros aenigmaticus        TATTTCGATACACCCGAAACATGGATTATATATAAACCCATGGATCGTGA
Phaeoceros laevis             TATTTCGATACACCGGAAACATGGATTATATATAAACCCATGGATCGTGA

Arabidopsis thaliana          --------------------------------------------------
Brassica napus                --------------------------------------------------
Lotus japonicus               --------------------------------------------------
Citrullus lanatus             --------------------------------------------------
Cucurbita pepo                --------------------------------------------------
Beta vulgaris maritima        --------------------------------------------------
Beta vulgaris vulgaris        --------------------------------------------------
Silene latifolia              --------------------------------------------------
BPM24_nad2                    CAAGTCGTTACTACTAGCAATGACTTCCTCTTTCATTACTTUATCCTTTC
RRII105_nad2                  CAAGTCGTTACTACTAGCAATGACTTCCTCTTTCATTACTTUATCCTTTC
RRIC110_nad2                  CAAGTCGTTACTACTAGCAATGACTTCCTCTTTCATTACTTUATCCTTTC
PB235_nad2                    CAAGTCGTTACTACTAGCAATGACTTCCTCTTTCATTACTTUATCCTTTC
RRIT251_nad2                  CAAGTCGTTACTACTAGCAATGACTTCCTCTTTCATTACTTUATCCTTTC
RRIM600_nad2                  CAAGTCGTTACTACTAGCAATGACTTCCTCTTTCATTACTTUATCCTTTC
Megaceros aenigmaticus        GAAATCGTTACTATTAGCAATTACTTTATTTCCTACCACTTCTTTCTTTC
Phaeoceros laevis             GAAATTGTTACTATTAGCAATTACTTTATTCTCTACCACTTCTTTCTTTC

Arabidopsis thaliana          --------------------------------------------------
Brassica napus                --------------------------------------------------
Lotus japonicus               --------------------------------------------------
Citrullus lanatus             --------------------------------------------------
Cucurbita pepo                --------------------------------------------------
Beta vulgaris maritima        --------------------------------------------------
Beta vulgaris vulgaris        --------------------------------------------------
Silene latifolia              --------------------------------------------------
```

```
BPM24_nad2                        TATATCCCTCTCCCTTGTTCTCAGTTACTCATCAAATGGCACTCAGTTCA
RRII105_nad2                      TATATCCCTCTCCCTTGTTCTCAGTTACTCATCAAATGGCACTCAGTTCA
RRIC110_nad2                      TATATCCCTCTCCCTTGTTCTCAGTTACTCATCAAATGGCACTCAGTTCA
PB235_nad2                        TATATCCCTCTCCCTTGTTCTCAGTTACTCATCAAATGGCACTCAGTTCA
RRIT251_nad2                      TATATCCCTCTCCCTTGTTCTCAGTTACTCATCAAATGGCACTCAGTTCA
RRIM600_nad2                      TATATCCCTCTCCCTTGTTCTCAGTTACTCATCAAATGGCACTCAGTTCA
Megaceros aenigmaticus            TATATCCTTCTCCCTCGTTTTTAGTTACTCACGAAATGGCACCAAGTTTA
Phaeoceros laevis                 TATATCCTTCTCCCTTGTTTTTAGTTACTCACGAAATGGCGCTAAGTTTA


Arabidopsis thaliana              ---------
Brassica napus                    ---------
Lotus japonicus                   ---------
Citrullus lanatus                 ---------
Cucurbita pepo                    ---------
Beta vulgaris maritima            ---------
Beta vulgaris vulgaris            ---------
Silene latifolia                  ---------
BPM24_nad2                        TATCTTTAA
RRII105_nad2                      TATCTTTAA
RRIC110_nad2                      TATCTTTAA
PB235_nad2                        TATCTTTAA
RRIT251_nad2                      TATCTTTAA
RRIM600_nad2                      TATCTTTAA
Megaceros aenigmaticus            TGTTTGTAA
Phaeoceros laevis                 TGTTTGTAA
```

###################

nad4L multiple sequence alignment

```
Arabidopsis thaliana              ---CATCTAATAACATTT--GAATTTATCTTAGATTCCACGTTCCCGAAA
Brassica napus                    ---CATCTAATAACATTT--GAATTTCTCTTAGATTACACGTTCCCGAAA
BPM24_nad4L                       -------------------------------------------------A
RRII105_nad4L                     -------------------------------------------------A
Citrullus lanatus                 ---CTTTTTATAACATTT--GAATTTCTCTGACATTCTACGTTCCCGAAA
Cucurbita pepo                    -CTGGAGTTATAACATTT--GAATTTCTCTGACATTCTACGTTCCCGAAA
Silene latifolia                  ---GGAGTTATAACATTT--GAATTTCTCTATTATTCTACGTTCCCGAAA
Vigna radiata                     -TATTGGTTATAACATTT--GAATTTCTCTTACATTCCACGTTTCCGAAA
RRIT251_nad4L                     -------------------------------------------------A
Oryza sativa Indica               -TAAGCCTCACATAATTT--GAATTTCTCTGACATTCCATGTTTCCGAAU
Oryza sativa Japonica             -TAGGCCTCACATAATTT--GAATTTCTCTGACATTCCATGTTTCCGAAA
PB235_nad4L                       -------------------------------------------------A
RRIC110_nad4L                     -------------------------------------------------A
RRIM600_nad4L                     -------------------------------------------------A
Cycas taitungensis                -----ATTAAGTTCCTTCCAGAATTCCTCCTCCGTTCCACGTTCCCGAAA
Pleurozia purpurea                -------------------------------------------------A
Megaceros aenigmaticus            GCTAGGCTGAAGTAGTGTT-GGACACCGGTAAAA--AAAGACTCTTATTA
Phaeoceros laevis                 --TAGGCTGAAGTAGTGTT-GGACACGGGTAAAA--AAAGACTCTTATTA


Arabidopsis thaliana              TGGATCTTATCAAATATTTCACATTTTCTATGATTATTTUTATTTTAGGT
Brassica napus                    TGGATCUTATCAAATATTTCACATTTTCTATGATTATTTUTATTTTAGGT
BPM24_nad4L                       TGGATCUTATCAAATATTTCACATTTTCTATGATCATCTUTATTTTAGGT
RRII105_nad4L                     TGGATCUTATCAAATATTTCACATTTTCTATGATCATCTUTATTTTAGGT
Citrullus lanatus                 UGGATCCTATCAAATATTTCACATTTTCTATGATUATCTUTATTTTAGGT
Cucurbita pepo                    UGGATCCTATCAAATATTTCACATTTTCTATGATUATCTUTATTTTAGGT
Silene latifolia                  UGGATTCTATCAAATATTTTACATTTTCTATGATCATTTTTATTTUAGGT
Vigna radiata                     UGGATCCTATCAAATATTTTACATTTTCTATGATCATCTCTATTTTAGGT
RRIT251_nad4L                     TGGATCCTATCAAATATTTCACATTTTCTATGATCATCTNTATTTTAGGT
Oryza sativa Indica               CGGATCCTATAAAATATTTCACTTTTTCTATGATCATCTCTATTTTAGGT
Oryza sativa Japonica             UGGATCUTATAAAATATTTCACTTTTTCTATGATCATCTUTATTTTAGGT
PB235_nad4L                       TGGATCUTATCAAATATTTCACATTTTCTATGATCATCTUTATTTTAGGT
```

```
RRIC110_nad4L                     TGGATCUTATCAAATATTTCACATTTTCTATGATCATCTNATTTTAGGT
RRIM600_nad4L                     TGGATCCTATCAAATATTTCACATTTTCTATGATCATCTCTATTTTAGGT
Cycas taitungensis                TGGATCTTGTCAAATATTTAACATTTCCTATGATCATTTCTCTCTCAGGT
Pleurozia purpurea                UGGATCTAGTAAAATATTTAACATTTTCTATGATTCTTTTTCTTTTAGGT
Megaceros aenigmaticus            TGGATCTAGTCAACTATTTAACATTCTCCATGATTCCTTCTTTTTTGGGT
Phaeoceros laevis                 TGGATCTAGTCAACTATTTAACATTCTCTATGATTCCTTTTTTTTTGGGT
                                   ****    * ** ***** ** **  * *****    * * * *  ***

Arabidopsis thaliana              ATTUGGGGAATCCTCCTTAATAGACGAAATATTCUTATTATGTUAATGUC
Brassica napus                    ATTUGGGGAATCCTCCTTAATAGACGAAATATTCUTATTATGTUAATGUC
BPM24_nad4L                       ATTCGGGGAATCCTCCTTAATAGACGAAATATTCCTATTATGTCAATGCC
RRII105_nad4L                     ATTCGGGGAATCCTCCTTAATAGACGAAATATTCCTATTATGTCAATGCC
Citrullus lanatus                 ATTUGGGGAATCCTCCTTAATAGACGAAATATTCUTATTATGTUAATGUC
Cucurbita pepo                    ATTUGGGGAATCCTCCTTAATAGACGAAATATTCUTATTATGTUAATGUC
Silene latifolia                  ATTUGGGGAATTCTCCTTAATAGACGAAATATTCTTATTATGTUAATGTC
Vigna radiata                     ATTCGGGGAATCCTCCTTAATAGACGAAATATTCCTATTATGTCAATGCC
RRIT251_nad4L                     ATTCGGGGAATCCTCCTTAATAGACGAAATATTCCTATTATGTCAATGCC
Oryza sativa Indica               ATTCGGGGAATCCTCCTTAATAGACGAAATATTCTTATTATGTCAATGCC
Oryza sativa Japonica             ATTUGGGGAATCCTCCTTAATAGACGAAATATTCTTATTATGTUAATGUC
PB235_nad4L                       ATTCGGGGAATCCTCCTTAATAGACGAAATATTCCTATTATGTCAATGCC
RRIC110_nad4L                     ATTCGGGGAATCCTCCTTAATAGACGAAATATTCCTATTATGTCAATGCC
RRIM600_nad4L                     ATTCGGGGAATCCTCCTTAATAGACGAAATATTCCTATTATGTCAATGCC
Cycas taitungensis                ATTCGGGGAATCTTCCTGAATAGACGAAATATTATTATTATGTCAATGTC
Pleurozia purpurea                ATTTGGGGAATTTTCTTAAATAGAAAAAATATTCTTATTATGTCAATGCC
Megaceros aenigmaticus            ATTTGGGGAATTCTTCCGAATAAAAGAAATATTCCTATTATGCCAACGCT
Phaeoceros laevis                 ATTTGGGGAATTTTTCCGAATAAAAGAAATATTCCTATTATGCCAACGCT
                                  *** *******  *    **** *  *******  *******  ** *

Arabidopsis thaliana              AATTGAATUAATGTTATTAGCTGTGAATTCGAACTTTTTGGTATTTTCCG
Brassica napus                    AATTGAATUAATGTTATTAGCTGTGAATTUGAACTTTTTGGTATTTTCCG
BPM24_nad4L                       AATTGAATCAATGTTATTAGCTGTGAATTTGAACTTTTTGGTATTTTCCG
RRII105_nad4L                     AATTGAATCAATGTTATTAGCTGTGAATTTGAACTTTTTGGTATTTTCCG
Citrullus lanatus                 AATTGAATUAATGTTATTAGCTGTGAATTUGAACTTTTTGGTATTTTCCG
Cucurbita pepo                    AATGGAATUAATGTTATTAGCTGTGAATTUGAACTTTTTGGTATTTTCGG
Silene latifolia                  AATTGAATUAATGTTATTAGCTGTGAATTUGAACTTTTTGGTCTTTTCTG
Vigna radiata                     AATTGAATCAATGTTATTAGCTGTGAATTCGAACTTTTTGGTATTTTCCG
RRIT251_nad4L                     AATTGAATCAATGTTATTAGCTGTGAATTTGAACTTTTTGGTATTTTCCG
Oryza sativa Indica               AATTGAATCAATGTTATTAGCTGTCAATTTGAACTTTTTGGTATTTTCCG
Oryza sativa Japonica             AATTGAATUAATGTTATTAGCTGTCAATTTGAACTTTTTGGTATTTTCCG
PB235_nad4L                       AATTGAATCAATGTTATTAGCTGTGAATTTGAACTTTTTGGTATTTTCCG
RRIC110_nad4L                     AATTGAATCAATGTTATTAGCTGTGAATTTGAACTTTTTGGTATTTTCCG
RRIM600_nad4L                     AATTGAATCAATGTTATTAGCTGTGAATTTGAACTTTTTGGTATTTTCCG
Cycas taitungensis                AATTGAATCAATGTTACTAGCTGCCAATTCGAACTTTTTGGTATTTCCCG
Pleurozia purpurea                AATTGAGTTAATGTTACTTGCTGTCAATTTGAACTTTTTGGTATTTTCTG
Megaceros aenigmaticus            GATTGAGTCAATGTTACTTGCAGTCAATTTGAATTCCTTGGTATTTCTGG
Phaeoceros laevis                 GATTGAGTTAATGTTACCTGTAGTCAATTTGAATTCCTTGGTATTTCTGG
                                   ** ** * *******   *  *  **** *** *  ***** ***   *

Arabidopsis thaliana              TTTCTTUGGATGATATGATGGGTCAAGTATTTGCTTUATTGGTTCUAACG
Brassica napus                    TTTCTTUGGATGATATGATGGGTCAAGTATTTGCTTCATTGGTTCCAACG
BPM24_nad4L                       TTTCTTCGGATGATATGATGGGTCAATCATTTGCTTCATTGGTTCUAACG
RRII105_nad4L                     TTTCTTCGGATGATATGATGGGTCAATCATTTGCTTCATTGGTTCUAACG
Citrullus lanatus                 TTTCTTUGGATGATATGATGGGTCAATUATTTGCTTUATTGGTTCUAACG
Cucurbita pepo                    TTTCTTUGGATGATATGATGGGTCAATUATTTGCTTUATTGGTTCUAACG
Silene latifolia                  TTTCTTUGGATGATATGATGGGTCAATTATTTGCTTTATTGGTTCUAACG
Vigna radiata                     TTTCTTCGGATGATATGATGGGTCAATCATTTGCTTCATTGGTTTCAACG
RRIT251_nad4L                     TTTCTTCGGATGATATGATGGGTCAATCATTTGCTTCATTGGTTCNAACG
Oryza sativa Indica               TTTCTTTGGATGATATGATGGGTCAATCATTTGCTTCATTAGTTCCAACA
Oryza sativa Japonica             TTTCTTTGGATGATATGATGGGTCAATUATTTGCTTUATTAGTTCUAACA
PB235_nad4L                       TTTCTTCGGATGATATGATGGGTCAATCATTTGCTTCATTGGTTCUAACG
RRIC110_nad4L                     TTTCTTCGGATGATATGATGGGTCAATCATTTGCTTCATTGGTTCUAACG
RRIM600_nad4L                     TTTCTTCGGATGATATGATGGGTCAATCATTTGCTTCATTGGTTCCAACG
```

```
Cycas taitungensis                TTTATCCGGATGATATGATGGGTCAATTATTTGCTTCATCGGTGTTAACG
Pleurozia purpurea                TTTATTCGGATGATATGATGGGTCAATTATTTGCTTTATTCGTTTTAACG
Megaceros aenigmaticus            TTCATTTGGATGATATGATGGGTCAATTATCCGCTCTATTTGCTTTAACG
Phaeoceros laevis                 TTCATTTGGATGATATGATGGGTCAATTATCCGCTCTATTTGCTTTAACG
                                  **  *  *******************  **  ***  **  *    ***

Arabidopsis thaliana              GTGGCAGCTGCGGAATCCGCTATTGGGTTAGCCATTTTCGTTATAACTTT
Brassica napus                    GTGGCAGCTGCGGAATCCGCTATTGGGTTAGCCATTTTCGTTATAACTTT
BPM24_nad4L                       GTGGCAGCTGCGGAATCTGCTATTGGGTTAGCCATTTTCGTTATTACTTT
RRII105_nad4L                     GTGGCAGCTGCGGAATCTGCTATTGGGTTAGCCATTTTCGTTATTACTTT
Citrullus lanatus                 GTGGCAGCTGCGGAATCCGCTATTGGGTTAGCCATTTTCGTTATTACTTT
Cucurbita pepo                    GTGGCAGCTGCGGAATCTGCTATTGGGTTAGCCATTTTCGTGATGACTTT
Silene latifolia                  GTGGCAGCTGCGGAATCTGCTATTGGGTTAGCTATTTTCGTTATTACTTT
Vigna radiata                     GTGGCAGCTGCGGAATCCGCTATTGGGTTAGCCATTTTCGTTATAACTTT
RRIT251_nad4L                     GTGGCAGCTGCGGAATCTGCTATTGGGTTAGCCATTTTCGTTATTACTTT
Oryza sativa Indica               GTGGCAGCTGCGGAATCTGCTATTGGATTAGCCATTTTCGTTATTACTTT
Oryza sativa Japonica             GTGGCAGCTGCGGAATCTGCTATTGGATTAGCCATTTTCGTTATTACTTT
PB235_nad4L                       GTGGCAGCTGCGGAATCTGCTATTGGGTTAGCCATTTTCGTTATTACTTT
RRIC110_nad4L                     GTGGCAGCTGCGGAATCTGCTATTGGGTTAGCCATTTTCGTTATTACTTT
RRIM600_nad4L                     GTGGCAGCTGCGGAATCTGCTATTGGGTTAGCCATTTTCGTTATTACTTT
Cycas taitungensis                GTGGCAGCTGCGGAATCTGCTATTGGGTCGGCCATTCCGGTTATTACTTT
Pleurozia purpurea                GTGGCTGCTGCGGAATCCGCTATTGGGTTAGCCATTCTGGTTATTACTTC
Megaceros aenigmaticus            ATGGCTGCTGTGGAATCTGCTATTGGGCCGGCCATTCTGGTCATTACTTT
Phaeoceros laevis                 ATGGCTGCTGTGGAATCTGCTATTGGGCCGGCCATTCTGGTCATTACTTT
                                   **** **** ****** ********    ** ***   ** ** ****

Arabidopsis thaliana              CCGAGTCCGAGGTACTATTGCTGTAGAATTTATTAATAGCATTCAAGGTT
Brassica napus                    CCGAGTCCGAGGGACTATTGCTGTAGAATTTATTAATAGCATTCAAGGTT
BPM24_nad4L                       CCGAGTCCGAGGGACTATTGCTGTAGAATTTATTAATAGCATTCAAGGTT
RRII105_nad4L                     CCGAGTCCGAGGGACTATTGCTGTAGAATTTATTAATAGCATTCAAGGTT
Citrullus lanatus                 CCGAGTCCGAGGGACTATTGCTGTAGAATTTATTAATAGCATTCAAGGTT
Cucurbita pepo                    CCGAGTCCGAGGGACTATTGCTGTAGAATTTATTAATAGCATTCAAGGTT
Silene latifolia                  CCGAGTCCGAGGGACTATTGCTGTAGAATUTATTAATAGCATTCAAGGTT
Vigna radiata                     CCGAGTCCGAGGGACTATTGCTGTAGAATTTATTAATAGCATTCAAGGTT
RRIT251_nad4L                     CCGAGTCCGAGGGACTATTGCTGTAGAATTTATTAATAGCATTCAAGGTT
Oryza sativa Indica               TCGAGTCCGAGGGACTATTGCTGTCGAATTTATAAATTGCATTCAAGGTT
Oryza sativa Japonica             TCGAGTCCGAGGGACTATTGCTGTCGAATTTATAAATTGCATTCAAGGTT
PB235_nad4L                       CCGAGTCCGAGGGACTATTGCTGTAGAATTTATTAATAGCATTCAAGGTT
RRIC110_nad4L                     CCGAGTCCGAGGGACTATTGCTGTAGAATTTATTAATAGCATTCAAGGTT
RRIM600_nad4L                     CCGAGTCCGAGGGACTATTGCTGTAGAATTTATTAATAGCATTCAAGGTT
Cycas taitungensis                CCGAATTCGAGGGACCATTGCAGTAGAATCTATTAATTGCATGAAGGGTU
Pleurozia purpurea                TCGAATTCGCGGTACTATTGCAGTGGAATTTATCAATTGCATGAAGGGTT
Megaceros aenigmaticus            TCGAATTCGCGGAACTATTGCTGTGGAATTGATTAGTTGCATGAAGGGTU
Phaeoceros laevis                 TCGAATTCGCGGAACTATTGCAGTGGAATTGATTAATTGCATGAAGGGTU
                                   *** * ** ** ** ***** ** ****  ** * * ****  * ***

Arabidopsis thaliana              AACCATTCCTCTCCTTTCAGTCGCTCGTCGAGACCTG-GTGGACCTTAC-
Brassica napus                    AACCATTCCTCTCCTTTCAGTCGA--GACTTCTCCTG-GTGGACCTTACA
BPM24_nad4L                       AA------------------------------------------------
RRII105_nad4L                     AA------------------------------------------------
Citrullus lanatus                 AAACATGACT---CCTAGAGAATTACCAAAATACGAA-GTTCTCTTCTCC
Cucurbita pepo                    AAAGATGACTACTCCTAGAGAATTACCAAAATACGAA-GTTATCTTC---
Silene latifolia                  AAACATGACT---CCTAGAGAGTGACCAAAATACGAA-GTTCTTTCGTTC
Vigna radiata                     AAACATGACT---CCTAGAGAATTACCAAAATACGAA-GTTCTCTTTTCT
RRIT251_nad4L                     AA------------------------------------------------
Oryza sativa Indica               AAACATAACT---ACAGGGGAGTTACCAAA-TACAAA-GTTCTGTTTTCC
Oryza sativa Japonica             AAACATAACT---ACAGGGGAGTTACCAAA-TACAAA-GTTCTGTTTTCC
PB235_nad4L                       AA------------------------------------------------
RRIC110_nad4L                     AA------------------------------------------------
RRIM600_nad4L                     AA------------------------------------------------
Cycas taitungensis                AAGCAGCACTGATTCTTCTTAGGAAGAAAAAGTTGTTTGTTCTCCGCT--
Pleurozia purpurea                AA------------------------------------------------
```

```
Megaceros aenigmaticus            AAACACAAAAA--CCAAAAGAATTGTTTTCTTA--GAAGTCTATTTCTGA
Phaeoceros laevis                 AAACACAAAAA--CCAAAATCATTTTTTTCTTCTCGAATACGAAGTA---
                                  **

Arabidopsis thaliana              --
Brassica napus                    G-
BPM24_nad4L                       --
RRII105_nad4L                     --
Citrullus lanatus                 TT
Cucurbita pepo                    --
Silene latifolia                  TC
Vigna radiata                     --
RRIT251_nad4L                     --
Oryza sativa Indica               T-
Oryza sativa Japonica             T-
PB235_nad4L                       --
RRIC110_nad4L                     --
RRIM600_nad4L                     --
Cycas taitungensis                --
Pleurozia purpurea                --
Megaceros aenigmaticus            --
Phaeoceros laevis                 --
```

###################

nad4 multiple sequence alignment

```
Beta macrocarpa                   ATGTTAGAACATTTCTGTGAATGCTATTUTGATCTAAGTGGTCTTATTCT
Beta vulgaris vulgaris            ATGTTAGAACATTTCTGTGAATGCTATTUTGATCTAAGTGGTCTTATTCT
Silene latifolia                  ATGTTAGAACATTTCTGTGAATGCTATTTTGATCTAAGTGGTCTTATTCT
Arabidopsis thaliana              ATGTTAGAACATTTCTGTGAATGCTATTUTAATCTAAGTGGTCTTATTCT
Brassica napus                    ATGTTAGAACATTTCTGTGAATGCTATTUTAATCTAAGTGGTCTTATTCT
Citrullus lanatus                 ATGTTAGAACATTTCTGTGAATGCTATTUTGATTTAAGTGGTCUTATTCT
Cucurbita pepo                    ATGTTAGAACATTTCTGTGAATGCTATTUTGATTTAAGTGGTCUTATTCT
Lotus japonicus                   ATGTTAGAACATTTCTGTGAATGCTATTUTGATCTAAGTGGTCUTATTCT
Millettia pinnata                 ATGTTAGAACATTTCTGTGAATGCTATTUTGATCTAAGTGGTCUTATTCT
BPM24_nad4                        ATGTTAGAACATTTCTGTGAATGCTATTUTGATCTAAGTGGTCUTATTCT
RRII105_nad4                      ATGTTAGAACATTTCTGTGAATGCTATTUTGATCTAAGTGGTCUTATTCT
RRIC110_nad4                      ATGTTAGAACATTTCTGTGAATGCTATTUTGATCTAAGTGGTCUTATTCT
RRIT251_nad4                      ATGTTAGAACATTTCTGTGAATGCTATTUTGATCTAAGTGGTCUTATTCT
PB235_nad4                        ATGTTAGAACATTTCTGTGAATGCTATTCTGATCTAAGTGGTCCTATTCT
RRIM600_nad4                      ATGTTAGAACATTTCTGTGAATGCTATTNTGATCTAAGTGGTCNTATTCT
Oryza sativa Japonica             ATGTTAGAACATTTCTGTGAATGCTATTTCGATCTAAGTGGTCUTATTCT
Cycas taitungensis                ATGTCACAACAATTTCGTGAATGCTATTCCAATCTAAGTGGTCCTATTCC
Megaceros aenigmaticus            AUGTTACAATTTCCAGCTTCACCTCATCTCAATCTAAGTGGTCTTATTTT
Phaeoceros laevis                 ACGTTACAATTTCCAGCTTTATCTCATCTCAATCTAAGTGGTCTTATTTT
Pleurozia purpurea                AUGTTACAAGTTTTAGCTCCATTTTCTTCCAATTTAAGTGGTCTCATTTT
                                  * ** * **        *  *     *    ** *********  ***

Beta macrocarpa                   GTGTCCCGTGCTAGGAAGCATTAUTCTTCTTTTCATTCCAAATTCAAGAA
Beta vulgaris vulgaris            GTGTCCCGTGCTAGGAAGCATTAUTCTTCTTTTCATTCCAAATTCAAGAA
Silene latifolia                  GTGTCCCGTGCTAGGAAGCATTAUTCTTCTTTTCATTCCAAATTCAAGAA
Arabidopsis thaliana              GTGTCCTGTGCTAGGAAGCATTAUTCTTCTTTUATTCCAAATTCAAGAA
Brassica napus                    GTGTCCTGTGCTAGGAAGCATTAUTCUTCTTTTCATTCCAAATTCAAGAA
Citrullus lanatus                 GTGTCCCGTGCTAGGAAGCATTAUTCUTCTTTTCATTCCAAATTCAAGAA
Cucurbita pepo                    GTGTCCCGTGCTAGGAAGCATTGUTCUTCTTTTCATTCCAAATGAAAGAA
Lotus japonicus                   GTGTCCCGTGCTAGGAAGCATTATTCUTCTTTTCATTCCAAATTCAAGAA
Millettia pinnata                 GTGTCCCGTGCTAGGAAGCATTATTCUTCTTTTCATTCCAAATTCAAGAA
BPM24_nad4                        ATGTCCCGTGCTAGGAAGCATTACTCCTCTTTTCATTCCAAATTCAAGAA
RRII105_nad4                      ATGTCCCGTGCTAGGAAGCATTACTCCTCTTTTCATTCCAAATTCAAGAA
RRIC110_nad4                      ATGTCCCGTGCTAGGAAGCATTACTCCTCTTTTCATTCCAAATTCAAGAA
RRIT251_nad4                      ATGTCCCGTGCTAGGAAGCATTACTCCTCTTTTCATTCCAAATTCAAGAA
PB235_nad4                        ATGTCCCGTGCTAGGAAGCATTACTCCTCTTTTCATTCCAAATTCAAGAA
```

```
RRIM600_nad4                      ATGTCCCGTGCTAGGAAGCATTACTCCTCTTTTCATTCCAAATTCAAGAA
Oryza sativa Japonica             CTGTCCCGTGCTAGGAAGCATTAUTCTTCTTTTCATTCCAAATTCTTCAA
Cycas taitungensis                GTGTCCCGTGCTAGGAAGCATTATTCCTCTTTTCCTTCCAAATTCGAGAA
Megaceros aenigmaticus            GCGTCCTTTGTTGGGAAGCATTATTCTTCTCGTTATCCCTGATTCAAGAA
Phaeoceros laevis                 GCGTCTTTTGCTGGGAAGCATTATTCTTCTCCTTATTCCTGATTCAAGAA
Pleurozia purpurea                GCTCCCTTTGCTAGGAAGCCTTATTATTTTGGTGACCCCGAATTCGAGAG
                                      *   ** * ****** **  *  * *  *    **  **     *

Beta macrocarpa                   TACGATUGATACGATTGATTGGTCTGTGCGCTTCTCTTATTACTTTTTTG
Beta vulgaris vulgaris            TACGATUGATACGATTGATTGGTCTGTGCGCTTCTCTTATTACTTTTTTG
Silene latifolia                  TACGATUGATACGATTGATTGGTCTGTGCGCTTCTCTTATTACTTTTTTG
Arabidopsis thaliana              TACGACUGATACGATTAATTGGTUTGTGTGCCTCTCTTATTACTTTTTTG
Brassica napus                    TACGACUGATACGATTAATTGGTUTGTGTGCCTCTCTTATTACTTTTTTG
Citrullus lanatus                 TACGACUGATACGATTGATTGGTCTATGTGCCTCTCTTATTACTTTTTTG
Cucurbita pepo                    TACGCCUGATACGATTGATTGGTCTATGTGCCTCTCTTATTACTTTTTTG
Lotus japonicus                   TACGACUGATACGATTGATTGGTCTGTGTGCCTCTCTTATTACTTTTTTG
Millettia pinnata                 TACGACUGATACGATTGATTGGTCTGTGTGCCTCTCTTATTACTTTTTTG
BPM24_nad4                        TACGACCGATACGATTGATTGGTCTGTGTGCCTCTCTTATTACTTTTTTG
RRII105_nad4                      TACGACCGATACGATTGATTGGTCTGTGTGCCTCTCTTATTACTTTTTTG
RRIC110_nad4                      TACGACCGATACGATTGATTGGTCTGTGTGCCTCTCTTATTACTTTTTTG
RRIT251_nad4                      TACGACCGATACGATTGATTGGTCTGTGTGCCTCTCTTATTACTTTTTTG
PB235_nad4                        TACGACCGATACGATTGATTGGTCTGTGTGCCTCTCTTATTACTTTTTTG
RRIM600_nad4                      TACGACCGATACGATTGATTGGTCTGTGTGCCTCTCTTATTACTTTTTTG
Oryza sativa Japonica             TAAGACTGATACGATTGATTGGTCTGTGCGTTTCTCTTATTACTTTTTTG
Cycas taitungensis                TGCGACTGATACGATCTATTGGTCTGTGCGCCTCTCTTCTTACTTTTTCG
Megaceros aenigmaticus            TCCAACTGATACGAAGTATTGGTCTGCGCACCTTTTTGATCACTTTCTTA
Phaeoceros laevis                 TACAACTGATACGAAGTATTGGTCCGCGCACCTTTTTGATCACTTTTTTA
Pleurozia purpurea                TACGTCTGATACGAGGTATCACAATTTGGACCTCTTTGATCACTTTTTTA
                                  *      *******   **        *    * * *  * ***** *

Beta macrocarpa                   TATTCTCUTGTTCTTUGGATACAATTTGATCCTTCTACGGCCAAATUTCA
Beta vulgaris vulgaris            TATTCTCUTGTTCTTUGGATACAATTTGATCCTTCTACGGCCAAATUTCA
Silene latifolia                  TATTCTCUTGTTCTTUGGATACAATTTGATCCTTCTACGGCCAAATUTCA
Arabidopsis thaliana              TATTCTCUTGTTCUTUGGATACAATTCGACTCTTCTACGGCCAAATUTCA
Brassica napus                    TATTCTCUTGTTCUTUGGATACAATTCGACTCTTCTACGGCCAAATUTCA
Citrullus lanatus                 TATUCCCUTGTTCUTUGGATACAATTCGATCCTTCTACGGCCAAATUTCA
Cucurbita pepo                    TATUCCCUTGTTCUTUGGATACAATTCGATCCTTCTACGGCCAAATUTCA
Lotus japonicus                   TATTCCCUTGTTCUTUGGATACAATTCGATCCTTCTACGGCCAAATUTCA
Millettia pinnata                 TATTCCCUTGTTCUTUGGATACAATTCGATCCTTCTACGGCCAAATUTCA
BPM24_nad4                        TATTCCCCTGTTCTTCGGATACAATTCGATCCTTCTACGGCCAAATCTCA
RRII105_nad4                      TATTCCCCTGTTCTTCGGATACAATTCGATCCTTCTACGGCCAAATCTCA
RRIC110_nad4                      TATTCCCCTGTTCTTCGGATACAATTCGATCCTTCTACGGCCAAATCTCA
RRIT251_nad4                      TATTCCCCTGTTCTTCGGATACAATTCGATCCTTCTACGGCCAAATCTCA
PB235_nad4                        TATTCCCCTGTTCTTCGGATACAATTCGATCCTTCTACGGCCAAATCTCA
RRIM600_nad4                      TATTCCCCTGTTCTTCGGATACAATTCGATCCTTCTACGGCCAAATCTCA
Oryza sativa Japonica             TATUCCCTTGTTCUTUGGATACAATTCGATCCTTCTACGGCCAAATUTCA
Cycas taitungensis                TACCCCCCTGTTCCTCGGATACAATTCGATTCTTCTACGGCCAAATCCCA
Megaceros aenigmaticus            TATTCTCCCTTTCTTTGGATAGAATTCGATAATTCTACAGCCAAATTCCA
Phaeoceros laevis                 TATTCTCCTTTTTTTTGGATAGAATTCGATAATTCTACAGCCAAATTTCA
Pleurozia purpurea                TATTCTCTTTTCTTTTGGATACGCTTCGAGAATGATACAGCAAAATTTCA
                                  **  * *   *   * *****   ** **   *  *** ** ****  **

Beta macrocarpa                   ATTTGTGGAAAGCCTTCGATGGCTTCCTTATGAAAACATCCATTTTTATT
Beta vulgaris vulgaris            ATTTGTGGAAAGCCTTCGATGGCTTCCTTATGAAAACATCCATTTTTATT
Silene latifolia                  ATTTGTCGAAAGCCTTCGATGGCTTCCTTATGAAAACATAAATTTTTATT
Arabidopsis thaliana              ATTTGTGGAAAGCCTTCGATGGCTTCCTTATGAAAACATCAATTTTTATT
Brassica napus                    ATTTGTGGAAAGCCTTCGATGGCTTCCTTATGAAAACATCAATTTTTATT
Citrullus lanatus                 ATTTGTGGAAAGCCTTCGATGGCTTCCTTATGAAAACATCAATTTTTATT
Cucurbita pepo                    ATTTGTGGAAAGCCTTCGATGGCTTCCTTATGAAAACATCAATTTTTCTT
Lotus japonicus                   ATTTGTGGAAAGCCTTCGATGGCTTCCTTATTCAAACATCAATTTTCATT
Millettia pinnata                 ATTTGTGGAAAGCCTTCGATGGCTTCCTTATGAAAACATCAATTTGAATT
BPM24_nad4                        ATTTGTGGAAAGCCTTCGATGGCTTCCTTATGAGAACATAAATTTTTATT
```

```
RRII105_nad4                      ATTTGTGGAAAGCCTTCGATGGCTTCCTTATGAGAACATAAATTTTTATT
RRIC110_nad4                      ATTTGTGGAAAGCCTTCGATGGCTTCCTTATGAGAACATAAATTTTTATT
RRIT251_nad4                      ATTTGTGGAAAGCCTTCGATGGCTTCCTTATGAGAACATAAATTTTTATT
PB235_nad4                        ATTTGTGGAAAGCCTTCGATGGCTTCCTTATGAGAACATAAATTTTTATT
RRIM600_nad4                      ATTTGTGGAAAGCCTTCGATGGCTTCCTTATGAGAACATAAATTTTTATT
Oryza sativa Japonica             ATTTGTGGAAAGCCTTCGATGGCTTCCTTATGAAAACATCCATTTGTATA
Cycas taitungensis                ATTTGTGGAAACCATTCGATGGCTTCCTTATGGAAGCATCAATTTGTATA
Megaceros aenigmaticus            ATTTGTGGAAAGCATTCGATGGCTTCCTTATTCAAACATCAATTTCTATA
Phaeoceros laevis                 ATTTGTGGAAAGCATTCGATGGCCTCCTTATTCAAACATCAATTTATATA
Pleurozia purpurea                GTTTGTGGAAACTATTCGATGGCTTCCGTATCCAAATATCAATTTCTATA
                                   ***** ****   ********* *** ***   *  **  ****   *

Beta macrocarpa                   TGGGTATAGAUGGTATCTCTTTATTCTTCGTGATATTGACCACATTTTTG
Beta vulgaris vulgaris            TGGGTATAGAUGGTATCTCTTTATTCTTCGTGATATTGACCACATTTTTG
Silene latifolia                  TGGGTATAGACGGTATCTCTTTATTCTTCGTGATATTGACCACATTTTTG
Arabidopsis thaliana              TGGGTATAGACGGTATCTCTTTATTCTTCGTGATATTGACCACATTTCTG
Brassica napus                    TGGGTATAGACGGTATCTCTTTATTCTTCGTGATATTGACCACATTTCTG
Citrullus lanatus                 TGGGTATAGAUGGTATCTCTTTATTCTTCGTGATATTGACCACATTTCTG
Cucurbita pepo                    TGGGTATAGACGGTATCTCTTTATTCTTCGTGATATTGACCACATTTCTG
Lotus japonicus                   TGGGTATAGACGGTCTCTCTTTTTTCTTCGTGATATTGACCACATTTCTG
Millettia pinnata                 TGGGTATAGACGGTCTCTCTTTTTTCTTCGTGATATTGACCACATTTCTG
BPM24_nad4                        TGGGTATAGACGGTATCTCTTTATTCTTCGTGATATTGACCACATTTCTG
RRII105_nad4                      TGGGTATAGACGGTATCTCTTTATTCTTCGTGATATTGACCACATTTCTG
RRIC110_nad4                      TGGGTATAGACGGTATCTCTTTATTCTTCGTGATATTGACCACATTTCTG
RRIT251_nad4                      TGGGTATAGACGGTATCTCTTTATTCTTCGTGATATTGACCACATTTCTG
PB235_nad4                        TGGGTATAGACGGTATCTCTTTATTCTTCGTGATATTGACCACATTTCTG
RRIM600_nad4                      TGGGTATAGACGGTATCTCTTTATTCTTCGTGATATTGACCACATTTCTG
Oryza sativa Japonica             TGGGTATAGACGGTCTTTCATTATTCTTCGTGATATTGACCACATTTCTG
Cycas taitungensis                TGGGTATAGATGGTATCCCTTCATCCTCCGTGATATTGACCACATTTTCA
Megaceros aenigmaticus            TAGGTATAGATGGTATCTCTTTATCTTTTGTGGTCTTGACCACGTTTCTA
Phaeoceros laevis                 TAGGTATAGATGGTATCTTTTTATCTTCTGCGGTCTTGACCACGTTTTTA
Pleurozia purpurea                TAGGTATAGATGGTATCTCGTTATTCTTTGTGATCTTGACCACGTTTTTA
                                  * ******** *** *    *  *  *  * * * ******** ***

Beta macrocarpa                   ATCCCTATTTGCATTTTAGTGGGTTGGTCTGGTATGAGAAGTTATGGGAA
Beta vulgaris vulgaris            ATCCCTATTTGCATTTTAGTGGGTTGGTCTGGTATGAGAAGTTATGGGAA
Silene latifolia                  ATCCCTATTTGCATTTTAGTGGGTTGGTCTGGTATGAGAAGTTATGGGAA
Arabidopsis thaliana              ATCCCTATTTGCATTTUAGTGGGTTGGTCTGGTATGAGAAGTTATGGGAA
Brassica napus                    ATCCCTATTTGCATTTUAGTGGGTTGGTCTGGTATGAGAAGTTATGGGAA
Citrullus lanatus                 ATCCCTATTTGCATTTTAGTGGGTTGGTCTGGTATGAGAAGTTATGGGAA
Cucurbita pepo                    ATCCCTATTTGCATTTTAGTGGGTTGGTCTGGTATGAGAAGTTATGGGAA
Lotus japonicus                   ATCCCTATTTGTATTTTAGTGGGTTGGTCTGGTATGAGAAGTTATGGGAA
Millettia pinnata                 ATCCCTATTTGTATTTTAGTGGGTTGGTCTGGTATGAGAAGTTATGGGAA
BPM24_nad4                        ATCCCTATTTGCATTTUAGTGGGTTGGTCTGGTATGAGAAGTTATGGGAA
RRII105_nad4                      ATCCCTATTTGCATTTUAGTGGGTTGGTCTGGTATGAGAAGTTATGGGAA
RRIC110_nad4                      ATCCCTATTTGCATTTUAGTGGGTTGGTCTGGTATGAGAAGTTATGGGAA
RRIT251_nad4                      ATCCCTATTTGCATTTUAGTGGGTTGGTCTGGTATGAGAAGTTATGGGAA
PB235_nad4                        ATCCCTATTTGCATTTUAGTGGGTTGGTCTGGTATGAGAAGTTATGGGAA
RRIM600_nad4                      ATCCCTATTTGCATTTNAGTGGGTTGGTCTGGTATGAGAAGTTATGGGAA
Oryza sativa Japonica             ATCCCTATTTGCATTTUAGTGGGTTGGTCTGGTATGAGAAGTTTTGGGAA
Cycas taitungensis                ATCCCTATTCGCATTTTAGTAGGTTGGTCCAGTATGAAAAGTTATGGGAA
Megaceros aenigmaticus            ATTCCTATTCGCATCTTAGTAGGTTGGTCCAGTACCAAGAGTTATAAGAA
Phaeoceros laevis                 ATTCCTATTCGCATTTCGGTAGGTTGGTCCAGTATCAAGAGTTATAAGAA
Pleurozia purpurea                ACCCCTATTTGCATTCTTGTTGGCTTTTATAGCGTCAAGAGTTATAAAAA
                                  *  ****** * **    ** ** *  *   *    *  **** *   **

Beta macrocarpa                   AGAGTATATTAUAGCATUTCTAATTUGTGAATTTCTAATGATCGCCGTGT
Beta vulgaris vulgaris            AGAGTATATTAUAGCATUTCTAATTUGTGAATTTCTAATGATCGCCGTGT
Silene latifolia                  AGAGTATATTAUAGCATUTCTAATTUGTGAATTTCTAATGATCGCCGTGT
Arabidopsis thaliana              AGAGTATATTAUAGCATTTTAATTUGTGAATTTCTAATGATCGCCGTGT
Brassica napus                    AGAGTATATTAUAGCATTTTAATTUGTGAATTTCTAATGATCGCCGTGT
Citrullus lanatus                 AGAGTATATTAUAGCATUTCTAATTUGTGAATTTCTAATGATCGCCGTGT
```

```
Cucurbita pepo                    AGAGTATATTAUAGCATUTCTAATTUGTGAATTTCTAATGATCGCCGTGT
Lotus japonicus                   AGAGTATATTAUAGCATUTCTAATTUGTGAATTTCTAATGATCGCCGTGT
Millettia pinnata                 AGAGTATATTAUAGCATUTCTAATTUGTGAATTTCTAATGATCGCCGTGT
BPM24_nad4                        AGAGTATATTACAGCATCTCTAATTCGTGAATTTCTAATGATCGCCGTGT
RRII105_nad4                      AGAGTATATTACAGCATCTCTAATTCGTGAATTTCTAATGATCGCCGTGT
RRIC110_nad4                      AGAGTATATTACAGCATCTCTAATTCGTGAATTTCTAATGATCGCCGTGT
RRIT251_nad4                      AGAGTATATTACAGCATCTCTAATTCGTGAATTTCTAATGATCGCCGTGT
PB235_nad4                        AGAGTATATTACAGCATCTCTAATTCGTGAATTTCTAATGATCGCCGTGT
RRIM600_nad4                      AGAGTATATTACAGCATCTCTAATTCGTGAATTTCTAATGATCGCCGTGT
Oryza sativa Japonica             AGAGTATATTAUAGCATTTCTAATTUGTGAATTTCTAATGATCGCCGTGT
Cycas taitungensis                GGAGTATATTATAGCATTTCCAATTCGTGAATTTCCAATGATCGCCGTGT
Megaceros aenigmaticus            AGAGTATATGATAGCGTTTTTAATTTGTGAATCTCTTATGATTGCTGTGT
Phaeoceros laevis                 AGAGTATATGATAGCGTTTTTAATTCGTGAATCTCTTATGATTGCTGTGT
Pleurozia purpurea                AGAGTATATGATAGCGTTTTTCGTTTGTGAATCTTTCCCAATTGCTGTGT
                                   ******** * *** * *    ** ****** *      ** ** ****

Beta macrocarpa                   TCTGCATGCTGGATCTTCTACTATTCTATGTTUTTTUCGAAAGCGTGCUA
Beta vulgaris vulgaris            TCTGCATGCTGGATCTTCTACTATTCTATGTTUTTTUCGAAAGCGTGCUA
Silene latifolia                  TCTGCATGCTGGATCTTCTACTATTCTATGTTUTTTUCGAAAGCGTGCTA
Arabidopsis thaliana              TUUGCATGCTAGATCTTUTACTATTCTATGTTTTTCCCGAAAGCGTGCUA
Brassica napus                    TCUGCATGCTGGATCTTCTACTATTCTATGTTTTTUUCGAAAGCGTGCUA
Citrullus lanatus                 TCUGCATGCTGGATCUTCTACTATTCTATGTTUTTUUCGAAAGCGTGCUA
Cucurbita pepo                    TCTGCATGCTGGATCUTCTACTATTCTATGTTUTTUUCGAAAGCGTGCTA
Lotus japonicus                   TCUGCATGCTGGATCUTCTACTATTCTATGTTUTTUUCGAAAGCGTGCTA
Millettia pinnata                 TCUGCATGCTGGATCUTCTACTATTCTATGTTUTTUUCGAAAGCGTGCTA
BPM24_nad4                        TCCGCATGCTGGATCCTCTACTATTCTATGTTCTTCCCGAAAGCGTGCTA
RRII105_nad4                      TCCGCATGCTGGATCCTCTACTATTCTATGTTCTTCCCGAAAGCGTGCTA
RRIC110_nad4                      TCCGCATGCTGGATCCTCTACTATTCTATGTTCTTCCCGAAAGCGTGCTA
RRIT251_nad4                      TCCGCATGCTGGATCCTCTACTATTCTATGTTCTTCCCGAAAGCGTGCTA
PB235_nad4                        TCCGCATGCTGGATCCTCTACTATTCTATGTTCTTCCCGAAAGCGTGCTA
RRIM600_nad4                      TCCGCATGCTGGATCCTCTACTATTCTATGTTCTTCCCGAAAGCGTGCTA
Oryza sativa Japonica             UCTGCATGCTGGATCUTCTACTATTCTATGTTUTTTUCGAAAGCGTGCUA
Cycas taitungensis                TTCGCATGCCGGATCCTTTACTATTTCATGTTCTTCCCGAAAGCGTGCTA
Megaceros aenigmaticus            CTCGCATGCCGGATCCTTTACTATCTCATGTCTTTCTTGAAAGCGTGTCA
Phaeoceros laevis                 CTCGCATGTCGGATTTTTTACTATTTCATGTCTTTTTTGAAAGCGTGTCA
Pleurozia purpurea                TTTGCTCACTCGATCTTTTAATATTTTACGTTTTTCTTGAAAGCGTATTA
                                     **      ***  * ** ***   * **  **   ********   *

Beta macrocarpa                   ATCCCTATGTTC---ATTATTATAGGGGTATGGGGTTCGAGACAAAGAAA
Beta vulgaris vulgaris            ATCCCTATGTTC---ATTATTATAGGGGTATGGGGTTCGAGACAAAGAAA
Silene latifolia                  ATCCCTATGTTC---ATTATTATAGGGGTCTGGGGTTCGAGACAAAGAAA
Arabidopsis thaliana              ATCCCTATGTTC---ATTATTATAGGAGTATGGGGTTCGAGACAAAGAAA
Brassica napus                    ATCCCTATGTTC---ATTATTATAGGAGTATGGGGTTCGAGACAAAGAAA
Citrullus lanatus                 ATCCCTATGTTC---ATTATTATAGGGGTATGGGGTTCGAGACAAAGAAA
Cucurbita pepo                    ATCCCTATGTTC---ATTATTATAGGGGTATGGGGTTCGAGACAAAGAAA
Lotus japonicus                   ATCCCTATGTTC---ATTATTATAGGGGTATGGGGTTCGAGACAAAGAAA
Millettia pinnata                 ATCCCTATGTTC---ATTATTATAGGGGTATGGGGTTCGAGACAAAGAAA
BPM24_nad4                        ATCCCTATGTTC---ATTATTATAGGGGTATGGGGTTCGAGACAAAGAAA
RRII105_nad4                      ATCCCTATGTTC---ATTATTATAGGGGTATGGGGTTCGAGACAAAGAAA
RRIC110_nad4                      ATCCCTATGTTC---ATTATTATAGGGGTATGGGGTTCGAGACAAAGAAA
RRIT251_nad4                      ATCCCTATGTTC---ATTATTATAGGGGTATGGGGTTCGAGACAAAGAAA
PB235_nad4                        ATCCCTATGTTC---ATTATTATAGGGGTATGGGGTTCGAGACAAAGAAA
RRIM600_nad4                      ATCCCTATGTTC---ATTATTATAGGGGTATGGGGTTCGAGACAAAGAAA
Oryza sativa Japonica             ATCCCTATGTTC---ATCATTATAGGGGTATGGGGTTCGAGACAAAGAAA
Cycas taitungensis                ATCCCTATGTTT---ATTATTATAGGGGTATGGGGTTCGAGACAAAGAAA
Megaceros aenigmaticus            ATTCTCATGTTTCTTATCATTATAGGGATATGGGGCTCTAGACAAAGAAA
Phaeoceros laevis                 ATTTCCATGTTT---------------------------AGACAAAGAAA
Pleurozia purpurea                ATTCCCATGTTT---ATTATTATAGGTGTTTGGGGGTCCAGACAAAGGAA
                                  **    *****                            ******** **

Beta macrocarpa                   GATCAAGGCAGCATATCAGTTTTTCCTTTATACTTTACTTGGATCTGTTT
Beta vulgaris vulgaris            GATCAAGGCAGCATATCAGTTTTTCCTTTATACTTTACTTGGATCTGTTT
```

```
Silene latifolia                  GATCAAGGCAGCATATCAGTTTTTCCTTTATACTTTACTTGGATCTGTTT
Arabidopsis thaliana              GATCAAGGCAGCATATCAGTTTTTCCTTTATACTTTACTTGGATCTCTTT
Brassica napus                    GATCAAGGCAGCATATCAGTTTTTCCTTTATACTTTACTTGGATCTCTTT
Citrullus lanatus                 GATCAAGGCAGCATATCAGTTTTTCCTTTATACTTTACTGGGATCCGTTT
Cucurbita pepo                    GATCAAGGCAGCATATCAGTTTTTCCTTTATACTTTACTGGGATCTGTTT
Lotus japonicus                   GATCAAGGCAGCATATCAGTTTTTCCTTTATACTTTACTTGGATCTCTTT
Millettia pinnata                 GATCAAGGCAGCATATCAGTTTTTCCTTTATACTTTACTTGGATCTGTTT
BPM24_nad4                        GATCAAGGCAGCATATCAGTTTTTCCTTTATACTTTACTTGGATCTGTTT
RRII105_nad4                      GATCAAGGCAGCATATCAGTTTTTCCTTTATACTTTACTTGGATCTGTTT
RRIC110_nad4                      GATCAAGGCAGCATATCAGTTTTTCCTTTATACTTTACTTGGATCTGTTT
RRIT251_nad4                      GATCAAGGCAGCATATCAGTTTTTCCTTTATACTTTACTTGGATCTGTTT
PB235_nad4                        GATCAAGGCAGCATATCAGTTTTTCCTTTATACTTTACTTGGATCTGTTT
RRIM600_nad4                      GATCAAGGCAGCATATCAGTTTTTCCTTTATACTTTACTTGGATCTGTTT
Oryza sativa Japonica             GATCAAGGCAGCATATCAGTTTTTCCTATATACTTTACTGGGATCCGTTT
Cycas taitungensis                GATCAAGGCAGCACATCAGTTTCTCCCATATACCTCACTCGGATCTGTTC
Megaceros aenigmaticus            AATACAAGCGGCATATCAGTTTTTCTTATATACCTTACTTGGATCTGTGT
Phaeoceros laevis                 AATACAAGCGGCATATCAGTCTTTCTTATATACCTTATCTGGATTTGTCT
Pleurozia purpurea                AATCAAAGCAGCATATCAGTTCTTCTTGTACACCTTGATGGGATCCTTGT
                                   **  * ** *** ******   **   ** ** *     ****   *

Beta macrocarpa                   TTATGCTATTAGCTATTCTGTTGATTCTTCTCCAAACAGGAACCACCGAT
Beta vulgaris vulgaris            TTATGCTATTAGCTATTCTGTTGATTCTTCTCCAAACAGGAACCACCGAT
Silene latifolia                  TTATGCTATTAGCTATTCTGTTGATTCTTCTCCAAACAGGAACCACCGAT
Arabidopsis thaliana              TTATGCTATTAGCTATTCTGTTGATTCTTTTCCAAACAGGAACCACCGAT
Brassica napus                    TTATGCTATTAGCTATTCTGTTGATTCTTTTCCAAACAGGAACCACCGAT
Citrullus lanatus                 TTATGCTATTAGCTATTCTGTTGATTCTTCTCCAAACAGGAACCACCGAT
Cucurbita pepo                    TTATGCTATTAGCTATTCTGTTGATTCTTCTCCAAACAGGAACCACCGAT
Lotus japonicus                   TTATGCTATTAGCTATTCTGTTGATTCTTTTCCAAACAGGGACCGCCGAT
Millettia pinnata                 TTATGCTATTAGCTATTTTGTTGATTCTTTTCCAAACAGGGACCGCCGAT
BPM24_nad4                        TTATGCTATTAGCTATTCTCTTGATTCTTCTCCAAACAGGAACCACCGAT
RRII105_nad4                      TTATGCTATTAGCTATTCTCTTGATTCTTCTCCAAACAGGAACCACCGAT
RRIC110_nad4                      TTATGCTATTAGCTATTCTCTTGATTCTTCTCCAAACAGGAACCACCGAT
RRIT251_nad4                      TTATGCTATTAGCTATTCTCTTGATTCTTCTCCAAACAGGAACCACCGAT
PB235_nad4                        TTATGCTATTAGCTATTCTCTTGATTCTTCTCCAAACAGGAACCACCGAT
RRIM600_nad4                      TTATGCTATTAGCTATTCTCTTGATTCTTCTCCAAACAGGAACCACCGAT
Oryza sativa Japonica             TTATGCTATTAGCTATTCTGTTGATTCTTCTCCAAACAGGAACCACCGAT
Cycas taitungensis                TTATGCTACTAGCTATTCCGTTTATTTTCTCCCAAACAGGAACCACCGAT
Megaceros aenigmaticus            TTATGCTCTTAGCCATCTCATCTATTTCTTTCCAAACAGGAACTACTGAT
Phaeoceros laevis                 TTATGCTTTTAGCCATTTCATCTATTTCTTTCCAAACAGGAACTACTGAT
Pleurozia purpurea                TCATGCTCTTAGCCATTTTATTTATTTTTTTCCAAACAGGAACCACTGAT
                                  * *****  **** **    *  ***     ********* **  * ***

Beta macrocarpa                   TTACAAATCTTATTAACCACAGAATTTAGTGAGCGGCGCCAAATCTTTTT
Beta vulgaris vulgaris            TTACAAATCTTATTAACCACAGAATTTAGTGAGCGGCGCCAAATCTTTTT
Silene latifolia                  TTACAAATCTTATTAACCACAGAATTTAGTGAGCGGCGCCAAATCTTTTT
Arabidopsis thaliana              TTACAAATATUATTAACCACAGAATTTAGTGAGCGGCGCCAAATCTTTCT
Brassica napus                    TTACAAATATUATTAACCACAGAATTTAGTGAGCGGCGCCAAATCTTTCT
Citrullus lanatus                 TTACAAATATUATTAACCACAGAATTTAGTGAGCGGCGCCAAATCTTTCT
Cucurbita pepo                    TTACAAATCTUATTAACCACTGAATTTAGTGAGCGGCGCCAAATCTTTCT
Lotus japonicus                   TTACAAATATUATTAACCACAGAATTTAGTGAGCGGCGCCAAATCTTTCT
Millettia pinnata                 TTACAAATATUATTAACCACAGAATTTAGTGAGCGGCGCCAAATCTTTCT
BPM24_nad4                        TTACAAATATUATTAACCACAGAATTTAGTGAGCGGCGCCAAATCTTTCT
RRII105_nad4                      TTACAAATATUATTAACCACAGAATTTAGTGAGCGGCGCCAAATCTTTCT
RRIC110_nad4                      TTACAAATATUATTAACCACAGAATTTAGTGAGCGGCGCCAAATCTTTCT
RRIT251_nad4                      TTACAAATATUATTAACCACAGAATTTAGTGAGCGGCGCCAAATCTTTCT
PB235_nad4                        TTACAAATATUATTAACCACAGAATTTAGTGAGCGGCGCCAAATCTTTCT
RRIM600_nad4                      TTACAAATATNATTAACCACAGAATTTAGTGAGCGGCGCCAAATCTTTCT
Oryza sativa Japonica             TTACAAATTTTATTAACCACAGAATTTAGTGAGCGGCGCCAAATCCTTCT
Cycas taitungensis                TCACAAATCTTATTAACCACAGAATTCAGTGAGCGGCGCCAAATCTTTCC
Megaceros aenigmaticus            TTACGAATATTATTAACCACAGAATTTAGTGAGCGGCGCCAAGTTTTGCT
Phaeoceros laevis                 TTACGAATATTATTAACCACAGAATCTAGTGAGTGGCGCCAAGTTTTGCT
Pleurozia purpurea                CTACAAATATTACTAACCACAGAATTTAGTGAGCGGCGCCAAATCTTGCT
```

```
                                  ** *** * * ******* ****  ****** ******** *  *

Beta macrocarpa                 ATGGATTGCTTTTTTCGCCTCTTTCGCTGTCAAAGTTCCTATGGTACCAG
Beta vulgaris vulgaris          ATGGATTGCTTTTTTCGCCTCTTTCGCTGTCAAAGTTCCTATGGTACCAG
Silene latifolia                ATGGATTGCTTTTTTCGCCTCTTTCGCTGTCAAAGTTCCTATGGTACCAG
Arabidopsis thaliana            ATGGATTGCTTUTTTCGCCTCTTTCGCCGTCAAAGTGCCTATGGTACCAG
Brassica napus                  ATGGATTGCTTUTTTCGCCTUTTTCGCCGTCAAAGTGCCTATGGTACCAG
Citrullus lanatus               ATGGATTGCTTUTTTCGCCTCTTTCGCCGTCAAAGTGCCTATGGTACCAG
Cucurbita pepo                  ATGGATTGCTTUTTTCGCCTCTTTCGCCGTCAAAGTGCCTATGGTACCAG
Lotus japonicus                 ATGGATTGCTTUTTTCGCCTCTTTCGCCGTCAAAGTGCCTATGGTACCAG
Millettia pinnata               ATGGATTGCTTUTTTCGCCGCTTTCGCCGTCAAAGTGCCTATGGTACCTG
BPM24_nad4                      ATGGATTGCTTUTTTCGCCTCTTTCGCCGTCAAAGTGCCTATGGTACCAG
RRII105_nad4                    ATGGATTGCTTUTTTCGCCTCTTTCGCCGTCAAAGTGCCTATGGTACCAG
RRIC110_nad4                    ATGGATTGCTTUTTTCGCCTCTTTCGCCGTCAAAGTGCCTATGGTACCAG
RRIT251_nad4                    ATGGATTGCTTUTTTCGCCTCTTTCGCCGTCAAAGTGCCTATGGTACCAG
PB235_nad4                      ATGGATTGCTTUTTTCGCCTCTTTCGCCGTCAAAGTGCCTATGGTACCAG
RRIM600_nad4                    ATGGATTGCTTNTTTCGCCTCTTTCGCCGTCAAAGTGCCTATGGTACCAG
Oryza sativa Japonica           ATGGATTGCTTTTTTCGCCTCTTTTGCCGTCAAAGTGCCTATGGTACCAG
Cycas taitungensis              ATGGATTGCTTCTTCCGCTCCTTTCCCCGTAAAAGTGCCTATGGTACCAG
Megaceros aenigmaticus          ACGGATTGCTTTTTTCGCTTCTTTTGCTGTGAAAGTGCCTATGGTACCTG
Phaeoceros laevis               ACGGATTGCTTTTTTCGCTTCTTTTGCTGTGAAAGTGCCTATGGTACCTG
Pleurozia purpurea              ATGGATTGCTTTTTTCGCTTCTTTTTCCGTGAAAGTGCCTATGGTACCAG
                                * ********* ** ***   ***  * ** ***** *********** *

Beta macrocarpa                 TTCATATTTGGTTACCCGAAGCTCATGTAGAGGCACCCACGGCAGGATCC
Beta vulgaris vulgaris          TTCATATTTGGTTACCCGAAGCTCATGTAGAGGCACCCACGGCAGGATCC
Silene latifolia                TTCATATTTGGTTACCCGAAGCTCATGTAGAGGCACCCACGGCAGGATCC
Arabidopsis thaliana            TTCATATTTGGTTACCTGAAGCTCATGTAGAGGCACCTACGGCAGGATCC
Brassica napus                  TTCATATTTGGTTACCCGAAGCTCATGTAGAGGCACCTACGGCAGGATCC
Citrullus lanatus               TTCATATTTGGTTACCCGAAGCTCATGTAGAGGCACCTACGGCAGGATCU
Cucurbita pepo                  TTCATATTTGGTTACCCGAAGCTCATGTAGAGGCACCTACGGCAGGATCC
Lotus japonicus                 TTCATATTTGGTTACCCGAAGCTCATGTAGAGGCACCTACGGCAGGATCC
Millettia pinnata               TTCATATTTGGTTACCCGAAGCTCATGTAGAGGCACCTACGGCTGGATCC
BPM24_nad4                      TTCATATTTGGTTACCCGAAGCTCATGTAGAGGCACCTACGGCAGGATCC
RRII105_nad4                    TTCATATTTGGTTACCCGAAGCTCATGTAGAGGCACCTACGGCAGGATCC
RRIC110_nad4                    TTCATATTTGGTTACCCGAAGCTCATGTAGAGGCACCTACGGCAGGATCC
RRIT251_nad4                    TTCATATTTGGTTACCCGAAGCTCATGTAGAGGCACCTACGGCAGGATCC
PB235_nad4                      TTCATATTTGGTTACCCGAAGCTCATGTAGAGGCACCTACGGCAGGATCC
RRIM600_nad4                    TTCATATTTGGTTACCCGAAGCTCATGTAGAGGCACCTACGGCAGGATCC
Oryza sativa Japonica           TTCATATTTGGTTACCCGAAGCCCATGTAGAGGCACCTACGGCTGGATCC
Cycas taitungensis              TTCATATCTGGTCACCCGAAGCTCATGTAGAGGCACCTACGGCTGGATCC
Megaceros aenigmaticus          TTCATATTTGGTTACCCGAAGCTCATGTAGAGGCACCTACGGCTGGATCT
Phaeoceros laevis               TTCATATTCGGTTACCCGAAGCTTATGTAGAGGCACCTACGGCTGGATCT
Pleurozia purpurea              TTCATATTTGGTTACCTGAAGCTCATGTGGAGGCACCTACGGCTGGATCT
                                *******  *** *** *****  **** ******** ***** *****

Beta macrocarpa                 GTTATCTTGGCAGGAATTCTTTTAAAATTGGGAACTTACGGGTTTTTAAG
Beta vulgaris vulgaris          GTTATCTTGGCAGGAATTCTTTTAAAATTGGGAACTTACGGGTTTTTAAG
Silene latifolia                GTTATCTTGGCAGGAATTCTTTTAAAATTGGGAACTTACGGGTTTTTAAG
Arabidopsis thaliana            GTCATCTTGGCAGGAATTCUTTTAAAATTTGGAACCUACGGGTTTTTAAG
Brassica napus                  GTCATCTTGGCAGGAATTCUTTTAAAATTTGGAACCUACGGGTTTTTAAG
Citrullus lanatus               GTCATCTTGGCAGGAATTCTTTTAAAATTGGGAACCUACGGGTTTTTAAG
Cucurbita pepo                  GTCATCTTGGCAGGAATTCTTTTAAAATTGGGAACCUACGGGTTTTTAAG
Lotus japonicus                 GTCATCTTGGCAGGAATTCUTTCAAAATTGGGAACCUACGGGTTTTTAAG
Millettia pinnata               GTCATCTTGGCAGGAATTCUTTCAAAATTGGGAACCUACGGGTTTTTAAG
BPM24_nad4                      GTCATCTTGGCAGGAATTCUTTTAAAATTGGGAACCTACGGCTTTTTAAG
RRII105_nad4                    GTCATCTTGGCAGGAATTCUTTTAAAATTGGGAACCTACGGCTTTTTAAG
RRIC110_nad4                    GTCATCTTGGCAGGAATTCUTTTAAAATTGGGAACCTACGGCTTTTTAAG
RRIT251_nad4                    GTCATCTTGGCAGGAATTCUTTTAAAATTGGGAACCTACGGCTTTTTAAG
PB235_nad4                      GTCATCTTGGCAGGAATTCUTTTAAAATTGGGAACCTACGGCTTTTTAAG
RRIM600_nad4                    GTCATCTTGGCAGGAATTCNTTTAAAATTGGGAACCTACGGCTTTTTAAG
Oryza sativa Japonica           GTCATCTTGGCTGGAATTCTTTTAAAATTGGGAACCTACGGGTTTTTAAG
```

```
Cycas taitungensis                GTCATCTCGGCAGGAATTCCTTCGAAATTGGGAACCTATGGGTTTTTGAG
Megaceros aenigmaticus            GTAATTTTGGCAGGAATTTTATCAAAATTGGGAACCTATGGTTTTTTAAG
Phaeoceros laevis                 GTAATCTCGGCAGGAATTTTATCAAAATTGGGAACCTATGGTTCTTTAAG
Pleurozia purpurea                GTAATCTTGGCAGGAATTCTTTTAAAATTGGGAACCTATGGTTTTTTAAG
                                  ** ** * *** ******   *  ***** *****  * ** * *** **

Beta macrocarpa                   ATTTTCAATACCCATGTTTCCTGAAGCGACACTTTGTTTCACTCCTTTCA
Beta vulgaris vulgaris            ATTTTCAATACCCATGTTTCCTGAAGCGACACTTTGTTTCACTCCTTTCA
Silene latifolia                  ATTTTCAATACCCATGTTTCCTGAAGCGACACTTTGTTTCACTCCCTTCA
Arabidopsis thaliana              ATTTTCAATACCCATGTTTCCCGAAGCGACACTTTGTTUTACTCCTTTCA
Brassica napus                    ATTTTCAATACCCATGTTTCCCGAAGCGACACTTTGTTUTACTCCTTTCA
Citrullus lanatus                 ATTTTCAATACCCATGTTTCCCGAAGCGACACTTTGTGCCACTCCTTTCA
Cucurbita pepo                    ATTTTCTATACCCATGTTTCCCGAAGCCACACTTTGTGCCACTCCTTTCA
Lotus japonicus                   ATTTTCAATACCCATGTTTCCCGAAGCAACACTTTGTTUCACTCCTTTCA
Millettia pinnata                 ATTTTCAATACCTATGTTTCCCGAAGCAACACTTTGTTUCACTCCTTTCA
BPM24_nad4                        ATTTTCAATACCCATGTTTCCCGAAGCGACACTTTCTTCCACTCCTTTCA
RRII105_nad4                      ATTTTCAATACCCATGTTTCCCGAAGCGACACTTTCTTCCACTCCTTTCA
RRIC110_nad4                      ATTTTCAATACCCATGTTTCCCGAAGCGACACTTTCTTCCACTCCTTTCA
RRIT251_nad4                      ATTTTCAATACCCATGTTTCCCGAAGCGACACTTTCTTCCACTCCTTTCA
PB235_nad4                        ATTTTCAATACCCATGTTTCCCGAAGCGACACTTTCTTCCACTCCTTTCA
RRIM600_nad4                      ATTTTCAATACCCATGTTTCCCGAAGCGACACTTTCTTCCACTCCTTTCA
Oryza sativa Japonica             ATTTTCAATACCCATGTTTCCCGAAGCGACACTTTGTTTCACTCCTTTCA
Cycas taitungensis                ATTTTCCATACCCATGTTTCCCGAAGCGACGCTTTGTCTCACTCCTTCCA
Megaceros aenigmaticus            ATTTTCCATACCCATGTTTCCTGAAGCGACCTTTTTTTTCATTCCCTTCA
Phaeoceros laevis                 ATTTTCCATACTCATGTTTCCTGAAGTGACCTTTTTTTTCATTTCTTTCA
Pleurozia purpurea                ATTTTCCATACCCATGTTTCCTGAAGCGACACTTTATTTCACTCCTTTCA
                                  ****** ****  ******** ****  **  *** *   * * * * **

Beta macrocarpa                   TTTATACTCTAAGCGCGATTGCTATAATATATACTTCCTTGACCACTTTA
Beta vulgaris vulgaris            TTTATACTCTAAGCGCGATTGCTATAATATATACTTCCTTGACCACTTTA
Silene latifolia                  TTTATACTCTAAGCGCGATTGCTATAATATATACTTCCTTGACCACTTTA
Arabidopsis thaliana              TTTATACTTTAAGCGCGATTGCTATAATATATACTTCCTTGACCACTTUA
Brassica napus                    TTTATACTTTAAGCGCGATTGCTATAATATATACTTCCTTGACCACTTUA
Citrullus lanatus                 TTTATACTUTAAGCGCGATTGCTATAATATATACTTCCTTGACCACTTUA
Cucurbita pepo                    TTTATACTUTAAGCGCGATTGCTATAATATATACTTCCTTGACCACTTUA
Lotus japonicus                   TTTATACTCCAAGCGCGATTGCTATAATATATACTTCCTTGACCACTTUA
Millettia pinnata                 TTTATACTCCAAGCGCGATTGCTATAATATATACTTCCTTGACCACTTUA
BPM24_nad4                        TTTATACTCCAAGCGCGATTGCTATAATATATACTTCCTTGACCACTTCA
RRII105_nad4                      TTTATACTCCAAGCGCGATTGCTATAATATATACTTCCTTGACCACTTCA
RRIC110_nad4                      TTTATACTCCAAGCGCGATTGCTATAATATATACTTCCTTGACCACTTCA
RRIT251_nad4                      TTTATACTCCAAGCGCGATTGCTATAATATATACTTCCTTGACCACTTCA
PB235_nad4                        TTTATACTCCAAGCGCGATTGCTATAATATATACTTCCTTGACCACTTCA
RRIM600_nad4                      TTTATACTCCAAGCGCGATTGCTATAATATATACTTCCTTGACCACTTCA
Oryza sativa Japonica             TTTATACTCTAAGCGCGATTGCTATAATATATACTTCCTTGACCACTTTA
Cycas taitungensis                TTTATACTCTAAGCGCGATCGCTACTATATATACTTCCCCGACCACTCCG
Megaceros aenigmaticus            TTTGTACTCTAAGCGTGATTGCTATTATATATACTCCCTTGACTACAGTG
Phaeoceros laevis                 TTTGTACTCTAAGCGTGATTGCTATTATATATACTCCCTTGACTACAGTG
Pleurozia purpurea                TTTATGTTCTAAGCGTGATTGCTATTATATACACTTCCTTAACTACAATA
                                  *** *  *  ***** *** ****  ***** *** **   ** **

Beta macrocarpa                   AGACAGATCGATCTTAAGAAGATCATTGCTTACTCCTCAGTAGCCCATAT
Beta vulgaris vulgaris            AGACAGATCGATCTTAAGAAGATCATTGCTTACTCCTCAGTAGCCCATAT
Silene latifolia                  AGACAGATCGATCTTAAGAAGATCATTGCTTACTCCTCAGTAGCCCATAT
Arabidopsis thaliana              AGACAGATCGATCTAAAGAAGATCATTGCTTACTCCTCAGTAGCCCATAT
Brassica napus                    AGACAGATCGATCTAAAGAAGATCATTGCTTACTCCTCAGTAGCCCATAT
Citrullus lanatus                 AGACAGATCGATCTTAAGAAGATCATTGCTTACTCCTCAGTAGCCCATAT
Cucurbita pepo                    AGACAGATCGATCTTAAGAAGATCATTGCTTACTCCTCAGTAGCCCATAT
Lotus japonicus                   AGACAGATCGATCTTAAGAAGATCATTGCTTACTCCTCAGTAGCCCATAT
Millettia pinnata                 AGACAGATCGATCTTAAGAAGATCATTGCTTACTCCTCAGTAGCCCATAT
BPM24_nad4                        AGACAGATCGATCTTAAGAAGATCATTGCTTACTCCTCAGTAGCCCATAT
RRII105_nad4                      AGACAGATCGATCTTAAGAAGATCATTGCTTACTCCTCAGTAGCCCATAT
RRIC110_nad4                      AGACAGATCGATCTTAAGAAGATCATTGCTTACTCCTCAGTAGCCCATAT
```

```
RRIT251_nad4                      AGACAGATCGATCTTAAGAAGATCATTGCTTACTCCTCAGTAGCCCATAT
PB235_nad4                        AGACAGATCGATCTTAAGAAGATCATTGCTTACTCCTCAGTAGCCCATAT
RRIM600_nad4                      AGACAGATCGATCTTAAGAAGATCATTGCTTACTCCTCAGTAGCCCATAT
Oryza sativa Japonica             AGACAGATCGATCTTAAGAAGATCATTGCCTACTCCTCAGTAGCCCATAT
Cycas taitungensis                AGACAAATCGATCCTAAGAAGATCATTGCCTACTCCCCAGTAGCCCATAC
Megaceros aenigmaticus            AGACAAATCGATCTGAAAAAAATGATTGCCTACTCTTCAGTAGCTCATAT
Phaeoceros laevis                 AGACAAATCGATCTGAAGAAAATGATTGCCTACTTCTCAGTAGCCCATAT
Pleurozia purpurea                AGACAAATCGATTTGAAGAAAATTATTGCTTACTCTTCAGTAGCCCATAT
                                  ***** ******   ** ** ** ***** ****   ******* ****

Beta macrocarpa                   GAATTTTGTGACTATTGGTATGTTTAGTCTGAACATACAGGGAATTGGAG
Beta vulgaris vulgaris            GAATTTTGTGACTATTGGTATGTTTAGTCTGAACATACAGGGAATTGGAG
Silene latifolia                  GAATTTTGTGACTATTGGTATGTTTAGTCTGAACATACAGGGAATTGGAG
Arabidopsis thaliana              GAATCTGGTGACTATTGGTATGTTTAGTCCGAACATACAGGGAATTGGAG
Brassica napus                    GAATCTGGTGACTATTGGTATGTTTAGTCCGAACATACAGGGAATTGGAG
Citrullus lanatus                 GAATCTGGTGACTATTGGTATGTTTAGTCUGAACATACAGGGAATTGGAG
Cucurbita pepo                    GAATCTGGTGACTATTGGTATCTTTAGTCUGAACATACAGGGAATTGGAG
Lotus japonicus                   GAATCTGGTGACTATTGGTATGTTTAGTCCGAACATACAGGGAATTGGAG
Millettia pinnata                 GAATCTGGTGACTATTGGTATGTTTAGTCCGAACATACAGGGAATTGGAG
BPM24_nad4                        GAATCTGGTGACTATTGGTATGTTTAGTCCGAACATACAGGGAATTGGAG
RRII105_nad4                      GAATCTGGTGACTATTGGTATGTTTAGTCCGAACATACAGGGAATTGGAG
RRIC110_nad4                      GAATCTGGTGACTATTGGTATGTTTAGTCCGAACATACAGGGAATTGGAG
RRIT251_nad4                      GAATCTGGTGACTATTGGTATGTTTAGTCCGAACATACAGGGAATTGGAG
PB235_nad4                        GAATCTGGTGACTATTGGTATGTTTAGTCCGAACATACAGGGAATTGGAG
RRIM600_nad4                      GAATCTGGTGACTATTGGTATGTTTAGTCCGAACATACAGGGAATTGGAG
Oryza sativa Japonica             GAATTTGGTGACTATTGGTATGTTTAGTCUGAACATACAGGGAATTGGGG
Cycas taitungensis                GAATTTCGTGACTATTGGTATGTCCAGTCCGAACATACAGGGAATTGGAG
Megaceros aenigmaticus            GAATTTTGTGACTATTGGTATGTTCAGTCTAAACATACAGGGAATTGAAG
Phaeoceros laevis                 GAATTTTGTGACTATTGGTATGTTCAGTCTAAACATACAGGGAATTGAAG
Pleurozia purpurea                GAATTTTGTGACTATTGGTATGTTCAGTCTGAACATACAGGGAATTGAAG
                                  **** * ************** *  ****  ****************  *

Beta macrocarpa                   GTAGCATTCTACUGATGTTAAGTCATGGACTGGTTTCTTCAGCCCTTTTT
Beta vulgaris vulgaris            GTAGCATTCTACUGATGTTAAGTCATGGACTGGTTTCTTCAGCCCTTTTT
Silene latifolia                  GTAGCATTCTACUGATGTTAAGTCATGGACTGGTTTCTTCAGCCCTTTTT
Arabidopsis thaliana              GTAGCATTCTACUGATGTTAAGTCATGGACTGGTTUCTTCAGCCCTTTTT
Brassica napus                    GTAGTATTCTACUGATGTTAAGTCATGGACTGGTTUCTTCAGCCCTTTTT
Citrullus lanatus                 GTAGCATTUTACUGATGTTAAGTCATGGACTGGTTUCTTCAGCCCTTTTT
Cucurbita pepo                    GTAGCATTUTACTGATGTTAAGTCATGGACTGGTTUCTTCAGCCCTTTTT
Lotus japonicus                   GTAGCATTCCACUGATGTTAAGTCATGGACTGGTTUCTTCAGCCCTTTTT
Millettia pinnata                 GTAGCATTCCACUGATGTTAAGTCATGGACTGGTTUCTTCAGCCCTTTTT
BPM24_nad4                        GTAGCATTCTACUGATGTTAAGTCATGGACTGGTTTCTTCAGCCCTTTTT
RRII105_nad4                      GTAGCATTCTACUGATGTTAAGTCATGGACTGGTTTCTTCAGCCCTTTTT
RRIC110_nad4                      GTAGCATTCTACUGATGTTAAGTCATGGACTGGTTTCTTCAGCCCTTTTT
RRIT251_nad4                      GTAGCATTCTACNGATGTTAAGTCATGGACTGGTTTCTTCAGCCCTTTTT
PB235_nad4                        GTAGCATTCTACUGATGTTAAGTCATGGACTGGTTTCTTCAGCCCTTTTT
RRIM600_nad4                      GTAGCATTCTACNGATGTTAAGTCATGGACTGGTTTCTTCAGCCCTTTTT
Oryza sativa Japonica             GTAGCATTTTACTTATGTTAAGTCATGGACTGGTTTCTTCAGCCCTTTTT
Cycas taitungensis                GTAGCATTCCACCGATGTCAAGTCATGGACCGGTTCCTTCAGCCCCTTTT
Megaceros aenigmaticus            GTAGCATTTCACTTATGTTAAGTCACGGACTTGTTTCCTTAGCCCCTCCT
Phaeoceros laevis                 GTAGCATTTCACCTATGTTAAGTCACGGACTGGTTCTCTTAGCCCCTCCT
Pleurozia purpurea                GTAGCATTTTACTTATGTTAAGTCATGGACTGGTTTCTTCAGCCCTTTTT
                                  **** ***  **  **** ****** ****  ***   * ***** *  *

Beta macrocarpa                   CTATGTGTTGGTGTTCTATATGACCGACATAAGACTCGACTTGTTAGATA
Beta vulgaris vulgaris            CTATGTGTTGGTGTTCTATATGACCGACATAAGACTCGACTTGTTAGATA
Silene latifolia                  CTATGTGTTGGTGTTCTATATGACCGACATAAGACTCGACTTGTTAGATA
Arabidopsis thaliana              CTATGTGTTGGTGTTTTATATGACCGACATAAGACTCGACTTGTTAGATA
Brassica napus                    CTATGTGTTGGTGTTCTATATGACCGACATAAGACTCGACTTGTTAGATA
Citrullus lanatus                 CTATGTGTTGGTGTTCTATATGACCGACATAAGACTCGACTTGTTAGATA
Cucurbita pepo                    CTATGTGTTGGGGTTCTATATGACCGACATAAGACTCGACTTGTTAGATA
Lotus japonicus                   CTATGTGTTGGTGTTCTATATGACCGACATAAGACTCGACTTGTTAGATA
```

```
Millettia pinnata                 CTATGTGTTGGTGTTCTATATGACCGACATAAGACTCGACTTGTTAGATA
BPM24_nad4                        CTATGTGTTGGTGTTCTATATGACCGACATAAGACTCGACTTGTTAGATA
RRII105_nad4                      CTATGTGTTGGTGTTCTATATGACCGACATAAGACTCGACTTGTTAGATA
RRIC110_nad4                      CTATGTGTTGGTGTTCTATATGACCGACATAAGACTCGACTTGTTAGATA
RRIT251_nad4                      CTATGTGTTGGTGTTCTATATGACCGACATAAGACTCGACTTGTTAGATA
PB235_nad4                        CTATGTGTTGGTGTTCTATATGACCGACATAAGACTCGACTTGTTAGATA
RRIM600_nad4                      CTATGTGTTGGTGTTCTATATGACCGACATAAGACTCGACTTGTTAGATA
Oryza sativa Japonica             CTATGTGTTGGTGTTCTATATGACCGACATAAGACTCGACTTGTTAGATA
Cycas taitungensis                CCATGTGTTGGTGTTCCATATGACCGACATAAGACTCGACCTGCTAAATA
Megaceros aenigmaticus            CCATGTGTTGGTGCCCTATATGACAGACATAAGACTCGACTTGTTAAATA
Phaeoceros laevis                 TTATGTGTTGGTGCTCTATATGACAGACATAAGACTCGACTTGTGAAATA
Pleurozia purpurea                TTATGTGTTGGGGCTCTATACGACCGACACAAGACAAGAATTGTTAAATA
                                    ********* *    *** *** **** *****  **  **  * ***

Beta macrocarpa                   TTAUGGAGGTTTAGTGAGCACCATGCCGAATTTCTCTACCATTTTCTTCT
Beta vulgaris vulgaris            TTAUGGAGGTTTAGTGAGCACCATGCCGAATTTCTCTACCATTTTCTTCT
Silene latifolia                  TTATGGGGGTTTAGTGAGCACCATGCCGAATTTCTCTACCATTTTCTTCT
Arabidopsis thaliana              TTAUGGAGGTTTAGTGAGCACCATGCCGAATUTCTCTACCATTTTCTTTT
Brassica napus                    TTAUGGAGGTTTAGTGAGCACCATGCCGAATUTCTCTACCATTTTCTTTT
Citrullus lanatus                 TTAUGGAGGTTUAGTGAGCACCATGCCGAATUTCTCTACCATTTUCTTCT
Cucurbita pepo                    TTAUGGAGGTTTAGTGAGCACCATGCCGAATUTCTCTACCATTTUCTTCT
Lotus japonicus                   TTAUGGAGGGTTAGTGAGCACCATGCCGAATUTCTCTACCATTTCCTTCT
Millettia pinnata                 TTAUGGAGGGTTAGTGAGCACCATGCCGAATUTCTCTACCATTTCCTTCT
BPM24_nad4                        TTACGGAGGTTTAGTGAGCACCATGCCGAATUTCTCTACCATTTTCTTCT
RRII105_nad4                      TTAUGGAGGTTTAGTGAGCACCATGCCGAATUTCTCTACCATTTTCTTCT
RRIC110_nad4                      TTACGGAGGTTTAGTGAGCACCATGCCGAATUTCTCTACCATTTTCTTCT
RRIT251_nad4                      TTAUGGAGGTTTAGTGAGCACCATGCCGAATUTCTCTACCATTTTCTTCT
PB235_nad4                        TTAUGGAGGTTTAGTGAGCACCATGCCGAATUTCTCTACCATTTTCTTCT
RRIM600_nad4                      TTANGGAGGTTTAGTGAGCACCATGCCGAATNTCTCTACCATTTTCTTCT
Oryza sativa Japonica             TTATGGAGGTTTAGTGAGCACCATGCCGAATTTCTCTACCATTTTCTTCT
Cycas taitungensis                TTATGGAGGTTCAGTAAGCACCATGCCAAATCCCTCTACCATTTTCTTCT
Megaceros aenigmaticus            TTATGGAGGGTTAGTTAGCACCATGCCAATTTTCTGTACCATTTCCTTAT
Phaeoceros laevis                 TTATGGAGGGTTAGTTAGCACCATGTCAATTTTCTGTACCATTTCCTTAT
Pleurozia purpurea                TTATGGAGGTTTAGTCAGCACCATGCCTATTTTCTCTACCATTTTCTTAT
                                  *** ** ** * *** ********* * * *  ** ******** *** *

Beta macrocarpa                   TTTTCACTTTAGCCAATATGAGTTTACCTGGTACTAGCAGCTTTATCGGG
Beta vulgaris vulgaris            TTTTCACTTTAGCCAATATGAGTTTACCTGGTACTAGCAGCTTTATCGGG
Silene latifolia                  TTTTCACTTTAGCCAATATGAGTTTACCTGGTACTAGCAGCTTTATCGGG
Arabidopsis thaliana              CTTTTACTTTGGCCAATATGAGTTUACCTGGTACTAGCAGCTTTATCGGG
Brassica napus                    UTTTTACTTTGGCCAATATGAGTTUACCTGGTACTAGCAGCTTTATCGGG
Citrullus lanatus                 UTTTCACTTTGGCCAATATGAGTTTACCTGGTACTAGCAGCTTTATCGGG
Cucurbita pepo                    UTTTCACTTTGGCCAATATGAGTTTACCTGGTACTAGCAGCTTTATCGGG
Lotus japonicus                   UTTCCACTTTGGCCAATATGAGTTUACCTGGTACTAGCAGCTTTATCGGG
Millettia pinnata                 UTTCCACTTTGGCCAATATGAGTTUACCTGGTACTAGCAGCTTTATCGGG
BPM24_nad4                        CTTCTACTTTGGCCAATATGAGTTUACCTGGTACTAGCAGCTTTATTGGG
RRII105_nad4                      CTTCTACTTTGGCCAATATGAGTTUACCTGGTACTAGCAGCTTTATTGGG
RRIC110_nad4                      CTTCTACTTTGGCCAATATGAGTTNACCTGGTACTAGCAGCTTTATTGGG
RRIT251_nad4                      CTTCTACTTTGGCCAATATGAGTTNACCTGGTACTAGCAGCTTTATTGGG
PB235_nad4                        CTTCTACTTTGGCCAATATGAGTTNACCTGGTACTAGCAGCTTTATTGGG
RRIM600_nad4                      CTTCTACTTTGGCCAATATGAGTTNACCTGGTACTAGCAGCTTTATTGGG
Oryza sativa Japonica             TTTTCACTTTAGCCAATATGAGTTTACCCGGCACTAGCAGCTTTATTGGG
Cycas taitungensis                TTTCCACTTTAGCCAATATGGGTTTACCTGGTACTAGCAGCTCTATCGGG
Megaceros aenigmaticus            TTTTCACTTTAGCGAATATGAGTTTACCGGGTAGTAGCAGCTTTATCGGG
Phaeoceros laevis                 TTTTCACTTTAGCGAATATGAGTTTATTGGGTAGTAGCAGCTTTATCGGG
Pleurozia purpurea                TTTTTACCCTAGCCAATATGAGTTTACCCGGTACTAGCAGCTTTATCGGG
                                   **  **  * ** ****** *** *   ** * ******** *** ***

Beta macrocarpa                   GAATTTCTCATCTTAGTAGGAGCTTTCCAAAGAAATAGCTTAGTAGCCAC
Beta vulgaris vulgaris            GAATTTCTCATCTTAGTAGGAGCTTTCCAAAGAAATAGCTTAGTAGCCAC
Silene latifolia                  GAATTTCTCATCTTAGTAGGAGCTTTCCAAAGAAATAGCTTAGTAGCCAC
Arabidopsis thaliana              GAATTTCTCATCTTAGTAGGAGCTTTCCAAAGAAATAGCTTAGTAGCCAC
```

```
Brassica napus                  GAATTTCUCATCTTAGTAGGAGCTTTCCAAAGAAATAGCTTAGTAGCCAC
Citrullus lanatus               GAATTTCUCATCTUAGTAGGAGCTTTCCAAAGAAATAGCTTAGTAGCCAC
Cucurbita pepo                  GAATTTCUCATCTUAGTAGGAGCTTTCCAAAGAAATAGCTTAGTAGCCAC
Lotus japonicus                 GAATTTCUCATCTCAGTAGGAGCTTTCCAAAGAAATAGCTTAGTAGCCAC
Millettia pinnata               GAATTTCUCATCTCAGTAGGAGCTTTCCAAAGAAATAGCTTAGTAGCCAC
BPM24_nad4                      GAATTTCTCATCTUAGTAGGAGCTTTCCAAAAAAATAGCTTAGTAGCCAC
RRII105_nad4                    GAATTTCTCATCTUAGTAGGAGCTTTCCAAAAAAATAGCTTAGTAGCCAC
RRIC110_nad4                    GAATTTCTCATCTUAGTAGGAGCTTTCCAAAAAAATAGCTTAGTAGCCAC
RRIT251_nad4                    GAATTTCTCATCTNAGTAGGAGCTTTCCAAAAAAATAGCTTAGTAGCCAC
PB235_nad4                      GAATTTCTCATCTNAGTAGGAGCTTTCCAAAAAAATAGCTTAGTAGCCAC
RRIM600_nad4                    GAATTTCTCATCTNAGTAGGAGCTTTCCAAAAAAATAGCTTAGTAGCCAC
Oryza sativa Japonica           GAATTTCTAATCTTAGTAGGAGCTTTCCAAAGAAATAGCTTAGTAGCCAC
Cycas taitungensis              GAATTTCCCATCTTAGTAGGAGCTTTCCAAAGAAATGGCTTAGTAGCCAC
Megaceros aenigmaticus          GAATTTCCCATCCCAACAGGAGCTTTCGAAAGGAATAGCTTAGTGGCCAC
Phaeoceros laevis               GAATTTCTCATCCTAATAGGAGCTTTCGAAAGGAATAGCTTAGTGGCCAC
Pleurozia purpurea              GAATTTCTCATTTTAGTAGGGGCTTTCCAAAGAAATAGCTTAGTGGCCAC
                                *******  **   *  *** ****** ***  *** ******* *****

Beta macrocarpa                 ATTAGCAGCGCTTGGGATGATTTTAGGCGCGGCCTATTCCCTTTGGCTAT
Beta vulgaris vulgaris          ATTAGCAGCGCTTGGGATGATTTTAGGCGCGGCCTATTCCCTTTGGCTAT
Silene latifolia                ATTAGCAGCGCTTGGGATGATTTTAGGCGCGGCCTATTCCCTTTGGCTAT
Arabidopsis thaliana            ATTAGCAGCGCTTGGGATGATTTTAGGTGCGGCCTATTCCCTTTGGCTAT
Brassica napus                  ATTAGCAGCGCTTGGGATGATTTTAGGTGCGGCCTATTCCCTTTGGCTAT
Citrullus lanatus               ATTAGCCGCGCTTGGGATGATTTTAGGCGCGGCGTATTCCCTTTGGCTAT
Cucurbita pepo                  ATTAGCCGCGCTTGGGATGATTTTAGGGGCGGCGTATTCCCTTTGGCTAT
Lotus japonicus                 ATTAGCAGCGCTTGGGATGATTTTAGGCGCGGCGTATTCCCTTTGGCTAT
Millettia pinnata               ATTAGCAGCGCTTGGGATGATTTTAGGCGCGGCGTATTCCCTTTGGCTAT
BPM24_nad4                      ATTAGCAGCGCTTGGGATGATTTTAGGCGCGGCCTATTCCCTTTGGCTAT
RRII105_nad4                    ATTAGCAGCGCTTGGGATGATTTTAGGCGCGGCCTATTCCCTTTGGCTAT
RRIC110_nad4                    ATTAGCAGCGCTTGGGATGATTTTAGGCGCGGCCTATTCCCTTTGGCTAT
RRIT251_nad4                    ATTAGCAGCGCTTGGGATGATTTTAGGCGCGGCCTATTCCCTTTGGCTAT
PB235_nad4                      ATTAGCAGCGCTTGGGATGATTTTAGGCGCGGCCTATTCCCTTTGGCTAT
RRIM600_nad4                    ATTAGCAGCGCTTGGGATGATTTTAGGCGCGGCCTATTCCCTTTGGCTAT
Oryza sativa Japonica           ATTAGCAGCGCTTGGGATGATTTTAGGCGCGGCGTATTCCCTTTGGCTAT
Cycas taitungensis              ATCAGCAGCGCTTGGGATGATTTCAGGCGCAGCGTATCCCCCTTGGCCAT
Megaceros aenigmaticus          ACTAGCGGCACTCGGGATGATTCTAGGCGCAGCTTATTCTCCTCGGTTAC
Phaeoceros laevis               ACCAGCGGCACTCGGGATGATTCTAGGCGCAGCTTATTTTCTTTGGTTAT
Pleurozia purpurea              ATTAGCAGCACTTGGGATGATTTTAGGCGCCGCTTACTCCCTCTGGCTAT
                                *  *** ** ** *********  *** ** ** **    *   **  *

Beta macrocarpa                 ATAATCGTGTGGTTTCTGGAAATTTAAAACCCGACTTCCTCCATAAATTC
Beta vulgaris vulgaris          ATAATCGTGTGGTTTCTGGAAATTTAAAACCCGACTTCCTCCATAAATTC
Silene latifolia                ATAATCGTGTGGTTTCTGGAAATTTAAAACCCGACTTCCTCCATAAATTC
Arabidopsis thaliana            ATAATCGTGTGGTTTCTGGAAATTTAAAACCTGATTTCCTCCATAAATTC
Brassica napus                  ATAATCGTGTGGTTTCTGGAAATTTAAAACCCGATTTCCTCCATAAATTC
Citrullus lanatus               ATAATCGTGTGGTTTCTGGAAATTTAAAACCCGATTTCCTCCATAAATTC
Cucurbita pepo                  ATAATCGTGTGGTTTCTGGAAATTTAAAAACCGATTTCCTCCATAAATTC
Lotus japonicus                 ATAATCGTGTGGTTTCTGGAAATTTAAAAGCCGATTTCCTCCATAAATTC
Millettia pinnata               ATAATCGTGTGGTTTCTGGAAATTTAAAAGCCGATTTCCTCCATAAATTC
BPM24_nad4                      ATAATCGTGTGGTTTCTGGAAATTTAAAACCCGATTTCCTCCATAAATTC
RRII105_nad4                    ATAATCGTGTGGTTTCTGGAAATTTAAAACCCGATTTCCTCCATAAATTC
RRIC110_nad4                    ATAATCGTGTGGTTTCTGGAAATTTAAAACCCGATTTCCTCCATAAATTC
RRIT251_nad4                    ATAATCGTGTGGTTTCTGGAAATTTAAAACCCGATTTCCTCCATAAATTC
PB235_nad4                      ATAATCGTGTGGTTTCTGGAAATTTAAAACCCGATTTCCTCCATAAATTC
RRIM600_nad4                    ATAATCGTGTGGTTTCTGGAAATTTAAAACCCGATTTCCTCCATAAATTC
Oryza sativa Japonica           ATAATCGTGTGGTTTCTGGAAATTTAAAACCCGATTTCCTCTATAAATTC
Cycas taitungensis              ATAATCGTGCGGTTCTTGGAAATTCAAAACCCAATTTCCCCCATAAATCC
Megaceros aenigmaticus          ATAATCGTGTGGTTTTTGGGAATTTCAAACCTAATTTCCTAAAAAAATTT
Phaeoceros laevis               ATAATTGTGTGGTTTTTGGGAATTTCAAACCTAATTTCCTAAAAAAATTT
Pleurozia purpurea              ATAATCGTGTGGTTTTCGGTAATTTCAAACCCAATTTTATCCTCAAATTC
                                ***** *** ****   ** ****  *** *  * **       ****
```

```
Beta macrocarpa                  TCCGATCUAAATGGCAGAGAAGTTTTCATATTTATACCCTTTCTTGTTGG
Beta vulgaris vulgaris           TCCGATCUAAATGGCAGAGAAGTTTTCATATTTATACCCTTTCTTGTTGG
Silene latifolia                 TCCGATCUAAATGGCAGAGAAATTTTCATATTTATACCCTTTCTTGTTGG
Arabidopsis thaliana             TCCGATTUAAATGGCAGAGAAGTTTUCATATTTATACCTTTTCTTGTTGG
Brassica napus                   TCCGATTUAAATGGCAGAGAAGTTTUCATATTTATACCTTTTCTTGTTGG
Citrullus lanatus                TCCGATCUAAATGGCAGAGAAGTTTUCATATTTATACCTTTTCTTGTTGG
Cucurbita pepo                   TCCGATCUAAATGGCAGAGAAGTTTUCATATTTATACCTTTTCTTGTTGG
Lotus japonicus                  TCCGATCUAAATGGCAGAGAAGTTTUCATATTTATACCTTTTCTTGTTGG
Millettia pinnata                TCCGATCUAAATGGCAGAGAAGTTTUCATATTTATACCTTTTCTTGTTGG
BPM24_nad4                       TCCGATTUAAATGGCAGAGAAGTTTCCATATTTATACCTTTTCTTGTTGG
RRII105_nad4                     TCCGATTUAAATGGCAGAGAAGTTTCCATATTTATACCTTTTCTTGTTGG
RRIC110_nad4                     TCCGATTUAAATGGCAGAGAAGTTTCCATATTTATACCTTTTCTTGTTGG
RRIT251_nad4                     TCCGATTUAAATGGCAGAGAAGTTTCCATATTTATACCTTTTCTTGTTGG
PB235_nad4                       TCCGATTUAAATGGCAGAGAAGTTTCCATATTTATACCTTTTCTTGTTGG
RRIM600_nad4                     TCCGATTNAAATGGCAGAGAAGTTTCCATATTTATACCTTTTCTTGTTGG
Oryza sativa Japonica            TCCGATCTAAATGGCAGAGAAGTTTUCATATTTCTACCTTTTCTTGTTGG
Cycas taitungensis               TCCGATCCAAATGGAAGAGAAGTTCCGATATCTCCACCTCTTACTGTTGG
Megaceros aenigmaticus           TCCGATTCAAATAGAAGAGAAGTTTTCATTTTTTTACCCTTTATTGTTGG
Phaeoceros laevis                TCCGATTTAAATAGAAGAGAAGTTTTCATTTCTTTACTCTTTATTGTTGG
Pleurozia purpurea               TCCGATTTAAATAGAAGAGAAGTTCTCATCTTTTTACCTTTTATTGCTGG
                                 ******  **** * ****** **   ** * *  **   **  ** ***

Beta macrocarpa                  AGTTGTTTGGATGGGTGTTUACCCCAAAGTGTTCCUGGACUGCATGCATA
Beta vulgaris vulgaris           AGTTGTTTGGATGGGTGTTUACCCCAAAGTGTTCCUGGACUGCATGCATA
Silene latifolia                 AGTTGTTUGGATGGGTGTTUACCCCAAAGTGTTCCUGGACUGCATGCATA
Arabidopsis thaliana             ACTCGTTUGGATGGGTGTTUACCCCAAAGTGTTCCUGGACTGCATGCATA
Brassica napus                   ACTCGTTUGGATGGGTGTTUACCCCAAAGTGTTCCUGGACTGCATGCATA
Citrullus lanatus                AGTTGTTUGGATGGGTGTTUACCCCAAAGTGTTCCUGGACUACATGCATA
Cucurbita pepo                   AGTTGTTCGGATGGGTGTTCACCCCAAAGTGTTCCCGGACTACATGCATA
Lotus japonicus                  AGTTGTTUGGATGGGTGTTUACCCCAAAGTGTTCCUGGACUGCATGCATA
Millettia pinnata                AGTTGTTUGGATGGGTGTTUACCCCAAAGTGTTCCUGGACUGCATGCATA
BPM24_nad4                       AGTTGTTTGGATGGGTGTTUACCCCAAAGTGTTCCUGGACTGCATGCATA
RRII105_nad4                     AGTTGTTTGGATGGGTGTTUACCCCAAAGTGTTCCUGGACTGCATGCATA
RRIC110_nad4                     AGTTGTTTGGATGGGTGTTUACCCCAAAGTGTTCCUGGACTGCATGCATA
RRIT251_nad4                     AGTTGTTTGGATGGGTGTTUACCCCAAAGTGTTCCUGGACTGCATGCATA
PB235_nad4                       AGTTGTTTGGATGGGTGTTUACCCCAAAGTGTTCCUGGACTGCATGCATA
RRIM600_nad4                     AGTTGTTTGGATGGGTGTTUACCCCAAAGTGTTCCCGGACTGCATGCATA
Oryza sativa Japonica            AGTTGTTUGGATGGGTGTTUACCCCAAAGTGTTCUUGGATTGCATGCATA
Cycas taitungensis               AGTTGTTCGGATGGGTGTTCACCCCAAGGTGTTCCCGGACCGTATGCATA
Megaceros aenigmaticus           AGTTATTCGGATGGGTGTTTACCCTGAAGTGTTCTTAGAGTGTATGCATA
Phaeoceros laevis                AGTTATTTGGATGGGTGTTTACCTTGAAGTGTTCTTAGAGTGTATGTATA
Pleurozia purpurea               AGTTATTTGGATGGGTGTTTACCCCGAAGTGTTCCTAGAGTGTATGCATA
                                 * *  ** *********** ***   * ******   **    *** ***

Beta macrocarpa                  CATCCGTAAGTAACTTAGTGCAACATGGAAAATTTCATTGA
Beta vulgaris vulgaris           CATCCGTAAGTAACTTAGTGCAACATGGAAAATTTCATTGA
Silene latifolia                 CATCCGTAAGTAACTTAGTGCAACATGGAAAATTTCATTGA
Arabidopsis thaliana             CATCCGTAAGTAACTTAGTGCAACATGGCAAATTTCATTGA
Brassica napus                   CATCCGTAAGTAACTTAGTGCAACATGGCAAATTTCATTGA
Citrullus lanatus                CATCCGTAAGTAACTTAGTGCAACATGGCAAATTTCATTGA
Cucurbita pepo                   CATCCGTAAGTAACTTAGTGCAACATGGCAAATTTCATTGA
Lotus japonicus                  CATACGTAAGTAACTTAGTGCAACATGGCAAATTTCATTGA
Millettia pinnata                CATACGTAAGTAACTTAGTGCAACATGGGAAATTTAATTGA
BPM24_nad4                       CATCCGTAAGTAACTTAGTGCAACATGGCAAATTTCATTGA
RRII105_nad4                     CATCCGTAAGTAACTTAGTGCAACATGGCAAATTTCATTGA
RRIC110_nad4                     CATCCGTAAGTAACTTAGTGCAACATGGCAAATTTCATTGA
RRIT251_nad4                     CATCCGTAAGTAACTTAGTGCAACATGGCAAATTTCATTGA
PB235_nad4                       CATCCGTAAGTAACTTAGTGCAACATGGCAAATTTCATTGA
RRIM600_nad4                     CATCCGTAAGTAACTTAGTGCAACATGGCAAATTTCATTGA
Oryza sativa Japonica            CATCCGTAAGTAACTTAGTGCAACATGGCAAATTTCATTGA
Cycas taitungensis               CCCCCGTAAGTAACTCAGTGCAACATGGCAAATTCCATUGA
Megaceros aenigmaticus           CTTGTGTGAGTAACTTAGTGCAACATGGAAAATTTGATTAA
```

```
Phaeoceros laevis                 CTTGTGTGAGTAACTTAGTGCAACATGGAAAATTTGATTAA
Pleurozia purpurea                CTTCCGTGAGTAACTTAGTGCAACATGGAAAATTTGATTAA
                                  *    ** ******* ************ *****  **  *
```

###################

nad5 multiple sequence alignment

```
Beta macrocarpa                   ATGTATTTACTTATCGTCTTTTTGCCCTTGCTTGGTAGTTCCGTAGCAGG
Beta vulgaris vulgaris            ATGTATTTACTTATCGTCTTTTTGCCCTTGCTTGGTAGTTCCGTAGCAGG
Silene latifolia                  ATGTATTTACTAATAGTCTTTTTGCCCTTGCTTGGTAGTTCCGTTGCAGG
RRIC110_nad5                      ATGTATCTACTTATCGTATTTTTGCCTCTGCTCGGTAGTTCCATAGCAGG
PB235_nad5                        ATGTATCTACTTATCGTATTTTTGCCTCTGCTCGGTAGTTCCATAGCAGG
RRIT251_nad5                      ATGTATCTACTTATCGTATTTTTGCCTCTGCTCGGTAGTTCCATAGCAGG
RRIM600_nad5                      ATGTATCTACTTATCGTATTTTTGCCTCTGCTCGGTAGTTCCATAGCAGG
RRII105_nad5                      ATGTATCTACTTATCGTATTTTTGCCTCTGCTCGGTAGTTCCATAGCAGG
BPM24_nad5                        ATGTATCTACTTATCGTATTTTTGCCTCTGCTCGGTAGTTCCATAGCAGG
Citrullus lanatus                 ATGTATCTACTTATCGTATTTTTGCCCCTGCTCGGTAGTTCCGTAGCAGG
Cucurbita pepo                    ATGTATCTCCTTATCGTCTTTTTGCCCCTGCTCGGTAGTTCCGTAGCTGG
Lotus japonicus                   ATGTATCTACTTATCGTATTTTTGCCCCTGCTCGGTAGTTCCGTAGCAGG
Millettia pinnata                 ATGTATCTACTTATCGTATTTTTGCCCCTTATCGGTAGTTCCTTAGCAGG
Brassica napus                    ATGTATCTACTTATCGTATTTTTACCCCTGCTCGGTAGTTCCGTAGCAGG
Megaceros aenigmaticus            ATGTATTTACTAATCGTCACTTTACCTTTGCCAGGTAGTTTCGTAGCAGG
Phaeoceros laevis                 ATGTATTTACTAATCGTAACTTTACTTTTGCCAGGTAGTTTTGTAGCAGG
                                  ****** * ** ** **   *** *   *    *******   * ** **

Beta macrocarpa                   TTTTTTCGGACGTTTTCTAGGATCAGAAGGAACTGCTATAGTGACTACTA
Beta vulgaris vulgaris            TTTTTTCGGACGTTTTCTAGGATCAGAAGGAACTGCTATAGTGACTACTA
Silene latifolia                  TTTTTTCGGACGTTTTCTAGGATCAGAAGGAACCGCTATAGTGACTACTA
RRIC110_nad5                      TTTTTTCGGACGTTTTCTAGGATCAGAAGGAACCGCTATAATGACCACTA
PB235_nad5                        TTTTTTCGGACGTTTTCTAGGATCAGAAGGAACCGCTATAATGACCACTA
RRIT251_nad5                      TTTTTTCGGACGTTTTCTAGGATCAGAAGGAACCGCTATAATGACCACTA
RRIM600_nad5                      TTTTTTCGGACGTTTTCTAGGATCAGAAGGAACCGCTATAATGACCACTA
RRII105_nad5                      TTTTTTCGGACGTTTTCTAGGATCAGAAGGAACCGCTATAATGACCACTA
BPM24_nad5                        TTTTTTCGGACGTTTTCTAGGATCAGAAGGAACCGCTATAATGACCACTA
Citrullus lanatus                 TTTTTTCGGACGTTTTCTAGGATCAGAAGGAACCGCTATAATCACCACTA
Cucurbita pepo                    TTTTTTCGGACGTTTTCTAGGATCAGAAGGAACCGCTATAATCACCACTA
Lotus japonicus                   TTTTTTCGGACGTTTTCTAGGATCAGAAGGAACCGCTATAATGACCACTA
Millettia pinnata                 TTTTTTCGGACGTTTTCTAGGATCAGAAGGAAGCGCTATAATGACCACTA
Brassica napus                    TTTTTTCGGACGTTTTCTAGGATCAGAAGGAAGCGCTATTCTGACCACTA
Megaceros aenigmaticus            TGCCTTTGGTCGTTTTCCTGGTTCACGAGGAACTGCTACAATCACAACCA
Phaeoceros laevis                 TGCCTTTGGTCGTTTTCTTGGTTCACGAGGAACTGCTACAATCACAACCA
                                  *   ** ** *******  ** ***  *****  ****   * ** ** *

Beta macrocarpa                   CGTGCGTTTCATTCTCTTCGATCTTATCTTTGATTGCTTTTTATGAAGTC
Beta vulgaris vulgaris            CGTGCGTTTCATTCTCTTCGATCTTATCTTTGATTGCTTTTTATGAAGTC
Silene latifolia                  CGTGCGTTTCATTCTCTTCGATCTTATCTTTTCTTGCTTTTTATGAAGTC
RRIC110_nad5                      CGTGCGTTTCATTCTCTTCGATCTTATCTTTGATTGCTTTTTATGAAGTC
PB235_nad5                        CGTGCGTTTCATTCTCTTCGATCTTATCTTTGATTGCTTTTTATGAAGTC
RRIT251_nad5                      CGTGCGTTTCATTCTCTTCGATCTTATCTTTGATTGCTTTTTATGAAGTC
RRIM600_nad5                      CGTGCGTTTCATTCTCTTCGATCTTATCTTTGATTGCTTTTTATGAAGTC
RRII105_nad5                      CGTGCGTTTCATTCTCTTCGATCTTATCTTTGATTGCTTTTTATGAAGTC
BPM24_nad5                        CGTGCGTTTCATTCTCTTCGATCTTATCTTTGATTGCTTTTTATGAAGTC
Citrullus lanatus                 CGTGCGTTTCATTCTCTTCGATCTTCTCTTTTCTTGCTTTTTATGAAGTC
Cucurbita pepo                    CGTGCGTTTCATTCTCTTCGATCTTATCTTTTCTTGCTTTTTATGAAGTC
Lotus japonicus                   CGTGCGTTTCATTCTCTTCGATCTTCTCTTTGATTGCTTTTTATGAAGTC
Millettia pinnata                 CGTGCGTTTCATTCTCTTCGATCTTATCTTTGATTGCTTTTTATGAAGTC
Brassica napus                    CGTGCGTTTCATTCTCTTCGATCTTATCTTTGATTGCTTTTTATGAAGTC
Megaceros aenigmaticus            TGTGTGTCTCATTATCTTGTATTTTCCCTTTGATTGCCTTTTATGAAGTT
Phaeoceros laevis                 TGTGTGTCTCATTATGTTCTATTTTCCTTTTGATTGCCTTTTATGAAGTT
                                   *** ** ***** * **  ** **   ***  **** ***********

Beta macrocarpa                   GCACUGGGAGCTAGTGCTTGCTATCTAAGAATTGCTCCATGGATTTCATC
Beta vulgaris vulgaris            GCACUGGGAGCTAGTGCTTGCTATCTAAGAATTGCTCCATGGATTTCATC
```

```
Silene latifolia                  GCACUGGGAGCTAGTGCTTGCTATCTAAGAATTGCTCCATGGATTTCATC
RRIC110_nad5                      GCACUGGGAGCTAGTGCTTGCTATCTAAGAATTGCTCCATGGATCTCATC
PB235_nad5                        GCACUGGGAGCTAGTGCTTGCTATCTAAGAATTGCTCCATGGATCTCATC
RRIT251_nad5                      GCACUGGGAGCTAGTGCTTGCTATCTAAGAATTGCTCCATGGATCTCATC
RRIM600_nad5                      GCACUGGGAGCTAGTGCTTGCTATCTAAGAATTGCTCCATGGATCTCATC
RRII105_nad5                      GCACUGGGAGCTAGTGCTTGCTATCTAAGAATTGCTCCATGGATCTCATC
BPM24_nad5                        GCACUGGGAGCTAGTGCTTGCTATCTAAGAATTGCTCCATGGATCTCATC
Citrullus lanatus                 GCACTGGGAGCTAGTGCTTGCTATCTAAGAATTGCTCCATGGATCTCATC
Cucurbita pepo                    GCACTGGGAGCTAGTGCTTGCTATCTAAGAATTGCTCCATGGATCTCATC
Lotus japonicus                   GCACUGGGAGCTAGTGCTTGCTATCTAAGAATTGCTCCATGGATCTCATC
Millettia pinnata                 GCACUGGGAGCTAGTGCTTGCTATCTAAGAATTGCTCCATGGATCTCATC
Brassica napus                    GCACUGGGAGCTAGTGCTTGCTATCTAAGAATTGCTCCATGGATCTCATC
Megaceros aenigmaticus            GCACTGGGAGCTAGTGTTTGCTATATAAAGATTGCTTCATGGATTATCTC
Phaeoceros laevis                 GCACTGGGAGCTAGTGTTTGCTATATAAAGATTGCTTCATGGATTATCTC
                                  **** *********** ******* ***  ****** *******    **

Beta macrocarpa                   GGAAATGTTTGATGCTTCTTGGGGCTTCTTGTTCGATAGCCUGACCGTAG
Beta vulgaris vulgaris            GGAAATGTTTGATGCTTCTTGGGGCTTCTTGTTCGATAGCCUGACCGTAG
Silene latifolia                  GGAAATGTTTGATGCTTCTTGGGGCTTCTT----------CUGACCGTAG
RRIC110_nad5                      GGAAATGTTTGATGCTTCTTGGGGCTTCTTGTTCGATAGCCTGACCGTAG
PB235_nad5                        GGAAATGTTTGATGCTTCTTGGGGCTTCTTGTTCGATAGCCTGACCGTAG
RRIT251_nad5                      GGAAATGTTTGATGCTTCTTGGGGCTTCTTGTTCGATAGCCTGACCGTAG
RRIM600_nad5                      GGAAATGTTTGATGCTTCTTGGGGCTTCTTGTTCGATAGCCTGACCGTAG
RRII105_nad5                      GGAAATGTTTGATGCTTCTTGGGGCTTCTTGTTCGATAGCCTGACCGTAG
BPM24_nad5                        GGAAATGTTTGATGCTTCTTGGGGCTTCTTGTTCGATAGCCTGACCGTAG
Citrullus lanatus                 GGAAATGTTTGATGCTTCTTGGGGCTTCTTGTTCGATAGCCUGACCGTAG
Cucurbita pepo                    GGAAATGTTTGATGCTTCTTGGGGCTTCTTGTTCGATAGCCTGACCGTAG
Lotus japonicus                   GGAAATGTTTGATGCTTCTTGGGGCTTCTTGTTCGATAGCCUGACCGTAG
Millettia pinnata                 GGAAATGTTTGATGCTTCTTGGGGCTTCTTGTTCGATAGCCUGACCGTAG
Brassica napus                    GGAAATGTTTGATGCTTCTTGGGGCTTCTTGTTCGATAGCCUGACCGTAG
Megaceros aenigmaticus            GGAGATGTTTGATGCTTTTCGGGGCTCCTTGTCTGATAGTCTGACTGTAG
Phaeoceros laevis                 GGAGATGTTTGATGCTTTTCGGGGCTCCTTGTTTGATAGTCTGACTGTAG
                                  *** ************* * ****** ***          * *** ****

Beta macrocarpa                   TGATGTTAATTGTGGTTACATTCATAAGTAGCTTGGTCCATCTTTATTCT
Beta vulgaris vulgaris            TGATGTTAATTGTGGTTACATTCATAAGTAGCTTGGTCCATCTTTATTCT
Silene latifolia                  TGATGTTAATTGTGGTTACATTCATAAGTAGCTTGGTCCATCTTTATTCT
RRIC110_nad5                      TGATGTTAATTGTGGTTACATTCATAAGTAGCTTGGTCCATCTTTATTCC
PB235_nad5                        TGATGTTAATTGTGGTTACATTCATAAGTAGCTTGGTCCATCTTTATTCC
RRIT251_nad5                      TGATGTTAATTGTGGTTACATTCATAAGTAGCTTGGTCCATCTTTATTCC
RRIM600_nad5                      TGATGTTAATTGTGGTTACATTCATAAGTAGCTTGGTCCATCTTTATTCC
RRII105_nad5                      TGATGTTAATTGTGGTTACATTCATAAGTAGCTTGGTCCATCTTTATTCC
BPM24_nad5                        TGATGTTAATTGTGGTTACATTCATAAGTAGCTTGGTCCATCTTTATTCC
Citrullus lanatus                 TGATGTTAATTGTGGTTACATUCATAAGTAGCTTGGTCCATCTTTATTCC
Cucurbita pepo                    TGATGTTAATTGTGGTTACATTCATAAGTAGCTTGGTCCATCTTTATTCC
Lotus japonicus                   TGATGTTAATTGTGGTTACATUCATAAGTAGCTTGGTCCATCTTTATTCC
Millettia pinnata                 TGATGTTAATTGTGGTTACATUCATAAGTAGCTTGGTCCATCTTTATTCC
Brassica napus                    TGATGTTAATTGTGGTTACATUCATAAGTAGCTTGGTCCATCTTTATTCC
Megaceros aenigmaticus            TTATGCTAATTGTGGTTACATTTGTAAGTAGCTTGGTTCATCTTTATCCC
Phaeoceros laevis                 TTATGTTAATTGTGGTTACATTTGTAAGTAGCTTGGTTCATCTTTATTTT
                                  * *** ***************   ************* *********

Beta macrocarpa                   ATTTCATATATGTCTGAGGATCCGCATAGCCCTCGATTTATGTGTTATTT
Beta vulgaris vulgaris            ATTTCATATATGTCTGAGGATCCGCATAGCCCTCGATTTATGTGTTATTT
Silene latifolia                  ATTTCATATATGTCTGAGGATCCGCATAGCCCTCGATTTATGTGTTATTT
RRIC110_nad5                      ATTTCATATATGTCTGAGGATCCGCATAGCCCTCGATTTATGTGTTATTT
PB235_nad5                        ATTTCATATATGTCTGAGGATCCGCATAGCCCTCGATTTATGTGTTATTT
RRIT251_nad5                      ATTTCATATATGTCTGAGGATCCGCATAGCCCTCGATTTATGTGTTATTT
RRIM600_nad5                      ATTTCATATATGTCTGAGGATCCGCATAGCCCTCGATTTATGTGTTATTT
RRII105_nad5                      ATTTCATATATGTCTGAGGATCCGCATAGCCCTCGATTTATGTGTTATTT
BPM24_nad5                        ATTTCATATATGTCTGAGGATCCGCATAGCCCTCGATTTATGTGTTATTT
Citrullus lanatus                 ATTTCATATATGTCUGAGGATCCGCATAGCCCTCGATTTATGTGTTATTT
```

```
Cucurbita pepo              ATTTCATATATGTCTGAGGATCCGCATAGCCCTCGATTTATGTGTTATTT
Lotus japonicus             ATTTCATATATGTCTGAGGATCCGCATAGCCCTCGATTTATGTGTTATTT
Millettia pinnata           ATTTCATATATGTCTGAGGATCCGCATAGCCCTCGATTTATGTGTTATTT
Brassica napus              ATTTCATATATGTCCGAGGATCCGCATAGCCCTCGATTTATGTGTTATTT
Megaceros aenigmaticus      ATTTCACATATGTCCGAGGATCCGCATAGCTCACGATTTATGTGTTATTT
Phaeoceros laevis           ATTTCACATATGTCCGAGGATCCGCATAGCTCCCGATTTATGTGTTATTT
                            ****** ******* *************** * *****************

Beta macrocarpa             ATCCATTUTTACTTTTTTTATGCUAATGTTGGTGACTGGAGATAACTUTC
Beta vulgaris vulgaris      ATCCATTUTTACTTTTTTTATGCUAATGTTGGTGACTGGAGATAACTUTC
Silene latifolia            ATCCATTUTTACTTTTTTTATGCUAATGTTGGTGACTGGAGATAACTUTC
RRIC110_nad5                ATCCATTTTTACTTTTTTTATGCUAATGTTGGTGACTGGAGATAACTUTC
PB235_nad5                  ATCCATTTTTACTTTTTTTATGCUAATGTTGGTGACTGGAGATAACTUTC
RRIT251_nad5                ATCCATTTTTACTTTTTTTATGCUAATGTTGGTGACTGGAGATAACTUTC
RRIM600_nad5                ATCCATTTTTACTTTTTTTATGCNAATGTTGGTGACTGGAGATAACTNTC
RRII105_nad5                ATCCATTTTTACTTTTTTTATGCUAATGTTGGTGACTGGAGATAACTUTC
BPM24_nad5                  ATCCATTTTTACTTTTTTTATGCUAATGTTGGTGACTGGAGATAACTUTC
Citrullus lanatus           ATCCATTTTTACTTTTTTTATGCUAATGTTGGTGACTGGAGATAACTUTC
Cucurbita pepo              ATCCATTTTTACTTTTTTTATGCUAATGTTGGTGACTGGAGATAACTUTC
Lotus japonicus             ATCCATTUTTACTTTTTTTATGCUAATGTTGGTGACTGGAGATAACTUTC
Millettia pinnata           ATCCATTUTTACTTTTTTTATGCUAATGTTGGTGACTGGAGATAACTUTC
Brassica napus              ATCCATTUTTACTTTTTTTATGCUAATGTTGGTGACTGGAGATAACTUTC
Megaceros aenigmaticus      ATCTATTCCTACTTTTTTTATGCCAATGCCGGTTACCGGAGATAACTCTA
Phaeoceros laevis           ATTTATTCCTACTCCTTTTATGCTAATGCTGGTTACCGGAGATAACTCTA
                            **  ***  ****  ******** ****  *** ** ********** * 

Beta macrocarpa             TTCAATTATTCTTGGGATGGGAGGGAGTAGGTCTTGCTTCATATTTGTTA
Beta vulgaris vulgaris      TTCAATTATTCTTGGGATGGGAGGGAGTAGGTCTTGCTTCATATTTGTTA
Silene latifolia            TTCAATTATTCTTGGGATGGGAGGGAGTAGGTCTTGCTTCATATTTGTTA
RRIC110_nad5                TTCAATTATTTCTGGGATGGGAAGGAGTAGGTCTTGCTTCATATTTGTTA
PB235_nad5                  TTCAATTATTTCTGGGATGGGAAGGAGTAGGTCTTGCTTCATATTTGTTA
RRIT251_nad5                TTCAATTATTTCTGGGATGGGAAGGAGTAGGTCTTGCTTCATATTTGTTA
RRIM600_nad5                TTCAATTATTTCTGGGATGGGAAGGAGTAGGTCTTGCTTCATATTTGTTA
RRII105_nad5                TTCAATTATTTCTGGGATGGGAAGGAGTAGGTCTTGCTTCATATTTGTTA
BPM24_nad5                  TTCAATTATTTCTGGGATGGGAAGGAGTAGGTCTTGCTTCATATTTGTTA
Citrullus lanatus           TTCAATTATTCCTGGGATGGGAGGGAGTAGGTCTTGCTTCATATTTGTTA
Cucurbita pepo              TTCAATTATTTCTGGGATGGGAAGGAGTAGGTCTTGCTTCATATTTGTTA
Lotus japonicus             TTCAATTATTCCTGGGATGGGAGGGAGTAGGTCTTGCTTCATATTTGTTA
Millettia pinnata           TTCAATTATTCCTGGGATGGGAGGGAGTAGGTCTTGCTTCATATTTGTTA
Brassica napus              TTCAATTATTCCTGGGATGGGAGGGAGTAGGTCTTGCTTCATATTTGTTA
Megaceros aenigmaticus      TTCAATTATTTCTAGGATGGGAGGGAGTCGGTCCTGCTTTATATTTGTCA
Phaeoceros laevis           TTCAATTATTTCTAGGATGGGAGGGAGTCGGTCTTGCTTTATATTTGTCA
                            **********  * ******** ***** **** ***** ******** *

Beta macrocarpa             ATTCATTTTTGGTTTACACGACTTCAGGCAGATAAAGCAGCTATAAAAGC
Beta vulgaris vulgaris      ATTCATTTTTGGTTTACACGACTTCAGGCAGATAAAGCAGCTATAAAAGC
Silene latifolia            ATTCATTTTTGGTTTACACGACTTCAGGCAGATAAAGCAGCTATAAAAGC
RRIC110_nad5                ATTCATTTCTGGTTTACACGACTTCAGGCAGATAAAGCAGCTATAAAAGC
PB235_nad5                  ATTCATTTCTGGTTTACACGACTTCAGGCAGATAAAGCAGCTATAAAAGC
RRIT251_nad5                ATTCATTTCTGGTTTACACGACTTCAGGCAGATAAAGCAGCTATAAAAGC
RRIM600_nad5                ATTCATTTCTGGTTTACACGACTTCAGGCAGATAAAGCAGCTATAAAAGC
RRII105_nad5                ATTCATTTCTGGTTTACACGACTTCAGGCAGATAAAGCAGCTATAAAAGC
BPM24_nad5                  ATTCATTTCTGGTTTACACGACTTCAGGCAGATAAAGCAGCTATAAAAGC
Citrullus lanatus           ATTCATTTCTGGTTTACACGACTTCAGGCAGATAAAGCAGCTATAAAAGC
Cucurbita pepo              ATTCATTTCTGGTTTACCCGACTTCAGGCAGATAAAGCAGCTATAAAAGC
Lotus japonicus             ATTCATTTCTGGTTTACACGACTTCAGGCAGATAAAGCAGCTAUAAAAGC
Millettia pinnata           ATTCATTTCTGGTTTACACGACTTCAGGCAGATAAAGCAGCTAUAAAAGC
Brassica napus              ATTCATTTTTGGTTTACACGACTTCAGGCAGATAAAGCAGCTAUAAAAGC
Megaceros aenigmaticus      ATTAATTTTTGGTTCACGAGACTTCAGGCCAATAAAGCAGCTATCAAAGC
Phaeoceros laevis           ATTAATTTTTGGTTCACGCGACTTCAGGCAAATAAGGCAGCTATCAAAGT
                            *** **** ***** **  **********  **** *******  **** 
```

```
Beta macrocarpa                  TATGCTTGTCAATCGAGTAGGTGATTTTGGATTAGCTCTTGGGATTTUGG
Beta vulgaris vulgaris           TATGCTTGTCAATCGAGTAGGTGATTTTGGATTAGCTCTTGGGATTTUGG
Silene latifolia                 TATGCTTGTCAATCGAGTAGGTGATTTTGGGTTAGCTCTTGGGATTTUGG
RRIC110_nad5                     TATGCTTGTCAATCGAGTAGGTGATTTTGGATTAGCTCUTGGGATTTCGG
PB235_nad5                       TATGCTTGTCAATCGAGTAGGTGATTTTGGATTAGCTCUTGGGATTTCGG
RRIT251_nad5                     TATGCTTGTCAATCGAGTAGGTGATTTTGGATTAGCTCNTGGGATTTCGG
RRIM600_nad5                     TATGCTTGTCAATCGAGTAGGTGATTTTGGATTAGCTCNTGGGATTTCGG
RRII105_nad5                     TATGCTTGTCAATCGAGTAGGTGATTTTGGATTAGCTCNTGGGATTTCGG
BPM24_nad5                       TATGCTTGTCAATCGAGTAGGTGATTTTGGATTAGCTCUTGGGATTTCGG
Citrullus lanatus                TATGCUTGTCAATCGAGTAGGTGATTTTGGATTAGCTCUTGGGATTTUGG
Cucurbita pepo                   TATGCUTGTCAATCGAGTAGGTGATTTTGGATTAGCTCUTGGGATTTUGG
Lotus japonicus                  TATGCUTGTCAATCGAGTAGGTGATTTTGGATTAGCTCUTGGGATTTUGG
Millettia pinnata                TATGCUTGTCAATCGAGTAGGTGATTTTGGATTAGCTCUTGGGATTTUGG
Brassica napus                   TATGCTTGTCAATCGAGTAGGTGATTTTGGATTAGCTCUTGGGATTTUGG
Megaceros aenigmaticus           TATGCCTGTGAATCGAGTAGGTGACTTTGGATTAGCTCCCGGGATTATGG
Phaeoceros laevis                TATGCTTGTGAATCGAGTAGGTGACTTTGGATTAGCTCTCGGGATTATGG
                                 ***** *** ************** ***** *******  ******  **

Beta macrocarpa                  GTTGTTTTACTCTCTTTCAAACAGTAGACTTTTCTACCATTTTTGCTTGT
Beta vulgaris vulgaris           GTTGTTTTACTCTCTTTCAAACAGTAGACTTTTCTACCATTTTTGCTTGT
Silene latifolia                 GTUGTTTTACTCTCTTTCAAACAGTAGACTTTTCTACCATTTTTTCTTGT
RRIC110_nad5                     GTCGTTTTACTCTCTTTCAAACAGTAGACTTTTCAACCATTTTTGCTCGT
PB235_nad5                       GTCGTTTTACTCTCTTTCAAACAGTAGACTTTTCAACCATTTTTGCTCGT
RRIT251_nad5                     GTCGTTTTACTCTCTTTCAAACAGTAGACTTTTCAACCATTTTTGCTCGT
RRIM600_nad5                     GTCGTTTTACTCTCTTTCAAACAGTAGACTTTTCAACCATTTTTGCTCGT
RRII105_nad5                     GTCGTTTTACTCTCTTTCAAACAGTAGACTTTTCAACCATTTTTGCTCGT
BPM24_nad5                       GTCGTTTTACTCTCTTTCAAACAGTAGACTTTTCAACCATTTTTGCTCGT
Citrullus lanatus                GTUGTTTTACTCTCTTTCAAACAGTAGACTTTTCCACCATTTTTGCTUGT
Cucurbita pepo                   GTUGTTTTACTCTCTTTCAAACAGTAGACTTTTCAACCATTTTTGCTUGT
Lotus japonicus                  GTUGTTTTACTCTCTTTCAAACAGTAGACTTTTCAACCATTTTTGCTUGT
Millettia pinnata                GTUGTTTTACTCTCTTTCAAACAGTAGACTTTTCAACCATTTTTGCTUGT
Brassica napus                   GTUGTTTTACTCTCTTTCAAACAGTAGACTTTTCAACCATTTTTGCTUGT
Megaceros aenigmaticus           GTGGTTTTACCCTTTTTCAAACAGTAGACTTTTCTACTATTTTTGCTCGT
Phaeoceros laevis                GTGGTTTTACCCTTTTTCAAATAGTAGACTTTTCTACTATTTTTGCTTGT
                                 ** ******* ** ******* ************ ** ****** ** **

Beta macrocarpa                  GCTAGTGUC---------CCTAGAAATTCTTGGATTTTTGCAATATGAG
Beta vulgaris vulgaris           GCTAGTGUC---------CCTAGAAATTCTTGGATTTTTGCAATATGAG
Silene latifolia                 GCTAGTGUC---------CCTAGAAATTCTTGGATTTUTTGCAATATGAG
RRIC110_nad5                     GCTAGTGCC---------CCCAGAAATTCTTGGATTTCTTGCAATATGAG
PB235_nad5                       GCTAGTGCC---------CCCAGAAATTCTTGGATTTCTTGCAATATGAG
RRIT251_nad5                     GCTAGTGCC---------CCCAGAAATTCTTGGATTTCTTGCAATATGAG
RRIM600_nad5                     GCTAGTGCC---------CCCAGAAATTCTTGGATTTCTTGCAATATGAG
RRII105_nad5                     GCTAGTGCC---------CCCAGAAATTCTTGGATTTCTTGCAATATGAG
BPM24_nad5                       GCTAGTGCC---------CCCAGAAATTCTTGGATTTCTTGCAATATGAG
Citrullus lanatus                GCTAGTGUC---------CCCAGAAATTCTTGGATTTUTTGCAATATGAG
Cucurbita pepo                   GCTAGTGUC---------CCCAGAAATTCTTGGATTTUTTGCAATATGAG
Lotus japonicus                  GCTAGTGCC---------CCCAGAAATTCTTGGATTTUTTGCAATATGAG
Millettia pinnata                GCTAGTGCC---------CCCAGAAATTCTTGGATTTUTTGCAATATGAG
Brassica napus                   GCTAGTGUC---------CCCAGAAATTCTTGGATTTUTTGCAATATGAG
Megaceros aenigmaticus           GCTAGTGCTTTCTCTGAACCCAACCATTTCTTTGTTTTTTGTAATATGAG
Phaeoceros laevis                GCTAGTGCCTTCTCTGAACCCAACCATTTTTTTCTTTTTTGTAATATGAG
                                 *******           ** *   ***  *   *** *** ********

Beta macrocarpa                  ATTGAATGCCATAACTCTTATTTGTATTTTACTTTTTATTGGTGCTGTTG
Beta vulgaris vulgaris           ATTGAATGCCATAACTCTTATTTGTATTTTACTTTTTATTGGTGCTGTTG
Silene latifolia                 ATTGAATGCCATAACTCTTATTTGTATTTTACTTTTTATTGGTGCTGTTG
RRIC110_nad5                     ATTGAATGCCATAACTCTTATTTGTATTTTACTTCTTATTGGTGCTGTTG
PB235_nad5                       ATTGAATGCCATAACTCTTATTTGTATTTTACTTCTTATTGGTGCTGTTG
RRIT251_nad5                     ATTGAATGCCATAACTCTTATTTGTATTTTACTTCTTATTGGTGCTGTTG
RRIM600_nad5                     ATTGAATGCCATAACTCTTATTTGTATTTTACTTCTTATTGGTGCTGTTG
RRII105_nad5                     ATTGAATGCCATAACTCTTATTTGTATTTTACTTCTTATTGGTGCTGTTG
```

```
BPM24_nad5                       ATTGAATGCCATAACTCTTATTTGTATTTTACTTCTTATTGGTGCTGTTG
Citrullus lanatus                ATTGAATGCCATAACTCTGATTTGTATTTTACTTUTTATTGGTGCTGTTG
Cucurbita pepo                   ATTGAATGCCATAACTCTGATTTGTATTTTACTTUTTATTGGTGCTGTTG
Lotus japonicus                  ATTGAATGCCATAACTCTGATTTGTATTTTACTTUTTATTGGTGCTGTTG
Millettia pinnata                ATTGAATGCCATAACTCTTATTTGTATTTTACTTUTTATTGGTGCTGTTG
Brassica napus                   ATTTAATGCCATAAGTCTTATTTGTATTTTACTTUTTATTGGTGCTGTTG
Megaceros aenigmaticus           ATTTCATGCTATAACTGTTATTTGTATTTTACCGCTCATTGGCGCTGTCG
Phaeoceros laevis                ATTTCATGCTATAACTGTTATTTGTATTTTACTGTTCATTGGCGCTGTTG
                                 ***  **** **** * * *************   * ***** ***** *

Beta macrocarpa                  GAAAATCTGCACAGATAGGATUGCATACTTGGTUACCCGATGCTATGGAG
Beta vulgaris vulgaris           GAAAATCTGCACAGATAGGATUGCATACTTGGTUACCCGATGCTATGGAG
Silene latifolia                 GAAAATCTGCACAGATAGGATUGCATACTTGGTUACCCGATGCTATGGAG
RRIC110_nad5                     GGAAATCTGCACAGATAGGATUGCATACTTGGTUACCCGATGCTATGGAG
PB235_nad5                       GGAAATCTGCACAGATAGGATUGCATACTTGGTUACCCGATGCTATGGAG
RRIT251_nad5                     GGAAATCTGCACAGATAGGATUGCATACTTGGTUACCCGATGCTATGGAG
RRIM600_nad5                     GGAAATCTGCACAGATAGGATUGCATACTTGGTNACCCGATGCTATGGAG
RRII105_nad5                     GGAAATCTGCACAGATAGGATUGCATACTTGGTUACCCGATGCTATGGAG
BPM24_nad5                       GGAAATCTGCACAGATAGGATUGCATACTTGGTUACCCGATGCTATGGAG
Citrullus lanatus                GGAAATCCGCACAGATAGGATUGCATACTTGGTUACCCGATGCTATGGAG
Cucurbita pepo                   GTAAATCCGCACAGATAGGATUGCATACTTGGTUACCCGATGCTATGGAG
Lotus japonicus                  GGAAATCTGCACAGATAGGATUGCATACTTGGTUACCCGATGCTATGGAG
Millettia pinnata                GGAAATCTGCACAGATAGGATUGCATACTTGGTUACCCGATGCTATGGAG
Brassica napus                   GGAAATCTGCACAGATAGGATUGCATACTTGGTUACCCGATGCTATGGAG
Megaceros aenigmaticus           GAAAATCTGCACAAATAGGATTGCATACTTGGTTACCCGACGTCATGGAG
Phaeoceros laevis                GAAAATTTGCACAAATAGGATTGCATACTTGGTTACCCGACGTCATGGAG
                                 * ****  ***** ******* *********** ****** *  ******

Beta macrocarpa                  GGTCCCACTCCAGTATCCGCTTTGATTCATGCAGCTACTATGGTAACAGC
Beta vulgaris vulgaris           GGTCCCACTCCAGTATCCGCTTTGATTCATGCAGCTACTATGGTAACAGC
Silene latifolia                 GGTCCCACTCCAGTATCCGCTTTGATTCATGCAGCTACTATGGTAACAGC
RRIC110_nad5                     GGTCCCACTCCAGTATCCGCTTTGATTCATGCAGCTACTATGGTAACGGC
PB235_nad5                       GGTCCCACTCCAGTATCCGCTTTGATTCATGCAGCTACTATGGTAACGGC
RRIT251_nad5                     GGTCCCACTCCAGTATCCGCTTTGATTCATGCAGCTACTATGGTAACGGC
RRIM600_nad5                     GGTCCCACTCCAGTATCCGCTTTGATTCATGCAGCTACTATGGTAACGGC
RRII105_nad5                     GGTCCCACTCCAGTATCCGCTTTGATTCATGCAGCTACTATGGTAACGGC
BPM24_nad5                       GGTCCCACTCCAGTATCCGCTTTGATTCATGCAGCTACTATGGTAACGGC
Citrullus lanatus                GGTCCCACTCCAGTATCCGCTTUGATTCATGCAGCTACTATGGTAACAGC
Cucurbita pepo                   GGGCCCACTCCAGTATCCGCTTUGATTCATGCAGCTACTATGGTAACAGC
Lotus japonicus                  GGTCCCACTCCAGTATCCGCTTTGATTCATGCAGCTACTATGGTCACAGC
Millettia pinnata                GGTCCCACTCCAGTATCCGCTTTGATTCATGCAGCTACTATGGTCACAGC
Brassica napus                   GGTCCCACTCCAGTATCCGCTTUGATTCATGCAGCTACTATGGTAACAGC
Megaceros aenigmaticus           GGTCCCACTCCAGTATCCGCTTCGATTCATGCAGCTACTACGGTCACAGC
Phaeoceros laevis                GGTCCCACTCCAGTATCCGCTTTGATTCATGCAGCTACTATGGTCACAGC
                                 ** ******************* ***************** *** ** **

Beta macrocarpa                  TGGCGTTTTCATGATAGCAAGGTGTTCCCCTTTATTTGAATACUCACCTA
Beta vulgaris vulgaris           TGGCGTTTTCATGATAGCAAGGTGTTCCCCTTTATTTGAATACUCACCTA
Silene latifolia                 TGGCGTTTTCATGATAGCAAGGTGTTCCCCTTTATTTGAATACUCACCTA
RRIC110_nad5                     TGGCGTTTTCATGATAGCAAGGTGCTCCCCTTTATTTGAATACTCACCTA
PB235_nad5                       TGGCGTTTTCATGATAGCAAGGTGCTCCCCTTTATTTGAATACTCACCTA
RRIT251_nad5                     TGGCGTTTTCATGATAGCAAGGTGCTCCCCTTTATTTGAATACTCACCTA
RRIM600_nad5                     TGGCGTTTTCATGATAGCAAGGTGCTCCCCTTTATTTGAATACTCACCTA
RRII105_nad5                     TGGCGTTTTCATGATAGCAAGGTGCTCCCCTTTATTTGAATACTCACCTA
BPM24_nad5                       TGGCGTTTTCATGATAGCAAGGTGCTCCCCTTTATTTGAATACTCACCTA
Citrullus lanatus                TGGCGTTTTCATGATAGCAAGGTGCTCCCCTTTATTTGAATACUCACCTA
Cucurbita pepo                   TGGCGTTTTCATGATAGCAAGGTGCTCCCCTTTATTTGAATACUCACCTA
Lotus japonicus                  TGGCGTTTTCATGATAGCAAGGTGCTCCCCTTTATTTGAATACUCACCTA
Millettia pinnata                TGGCGTTTTCATGATAGCAAGGTGCTCCCCTTTATTTGAATACUCACCTA
Brassica napus                   TGGCGTTTTCATGATAGCAAGGTGCTCCCCTTTATTTGAATACUCACCTA
Megaceros aenigmaticus           AGGCGTTTTTATGATAGCGAGGTGCCCTCCTTATTTGAATACCCGCCTA
Phaeoceros laevis                AGGCGTTTCTATGATAGCGAGGTGCCTTCTCTTATTTGAATACTTACCTA
```

```
                             *******  ******** *****    *  ************   ****

Beta macrocarpa             CGGCTTTAATTGTTATTACTTTTGCAGGAGCTATGACGTCATTCCTTGCG
Beta vulgaris vulgaris      CGGCTTTAATTGTTATTACTTTTGCAGGAGCTATGACGTCATTCCTTGCG
Silene latifolia            CGGCTTTAATTGTTATTACTTTTGCAGGAGCTATGACGTCATTCCTTGCG
RRIC110_nad5                CGGCTTTGATTGTTATTACTTTTGCAGGAGCTATGACGTCATTCCTTGCG
PB235_nad5                  CGGCTTTGATTGTTATTACTTTTGCAGGAGCTATGACGTCATTCCTTGCG
RRIT251_nad5                CGGCTTTGATTGTTATTACTTTTGCAGGAGCTATGACGTCATTCCTTGCG
RRIM600_nad5                CGGCTTTGATTGTTATTACTTTTGCAGGAGCTATGACGTCATTCCTTGCG
RRII105_nad5                CGGCTTTGATTGTTATTACTTTTGCAGGAGCTATGACGTCATTCCTTGCG
BPM24_nad5                  CGGCTTTGATTGTTATTACTTTTGCAGGAGCTATGACGTCATTCCTTGCG
Citrullus lanatus           CGGCTTTGATTGTTATTACTTUTGCAGGAGCTAUGACGTCATTCCTTGCG
Cucurbita pepo              CTGCTTTGATTGTTATTACTTUTGCAGGAGCTAUGACGTCATTCCTTGCG
Lotus japonicus             CGGCTTTGATTGTTATTACTTUTGCAGGAGCTAUGACGTCATTCCTTGCG
Millettia pinnata           CGGCTTTGATTGTTATTACTTUTGCAGGAGCTAUGACGTCATTCCTTGCG
Brassica napus              CGGCTTTGATTGTTATTACTTUTGCAGGAGCTAUGACGTCATTCCTTGCG
Megaceros aenigmaticus      ATGCTTTGATTGTTGTTACTCCTGTAGGAGCTATGACGCCATTCTTTGCG
Phaeoceros laevis           ATGCTTTGATTGTTGTTACTTCTGTAGGAGCTATGATGCTATTTTTTGCG
                              ***** ****** *****  ** ******** ** *  ***  *****

Beta macrocarpa             GCAACCACTGGAATATTACAGAACGATCTAAAGAGGGTCATAGCTTATTC
Beta vulgaris vulgaris      GCAACCACTGGAATATTACAGAACGATCTAAAGAGGGTCATAGCTTATTC
Silene latifolia            GCAACCACTGGAATATTACAGAACGATCTAAAGAGGGTCATAGCTTATTC
RRIC110_nad5                GCAACTACTGGAATATTACAGAACGATCTAAAGAGGGTCATAGCTTATTC
PB235_nad5                  GCAACTACTGGAATATTACAGAACGATCTAAAGAGGGTCATAGCTTATTC
RRIT251_nad5                GCAACTACTGGAATATTACAGAACGATCTAAAGAGGGTCATAGCTTATTC
RRIM600_nad5                GCAACTACTGGAATATTACAGAACGATCTAAAGAGGGTCATAGCTTATTC
RRII105_nad5                GCAACTACTGGAATATTACAGAACGATCTAAAGAGGGTCATAGCTTATTC
BPM24_nad5                  GCAACTACTGGAATATTACAGAACGATCTAAAGAGGGTCATAGCTTATTC
Citrullus lanatus           GCAACCACGGGAATATTACAGAACGATCTAAAGAGGGTCATAGCTTATTC
Cucurbita pepo              GCAACCACTGGAATATTACAGAACGATCTAAAGAGGGTCATAGCTTATTC
Lotus japonicus             GCAACCACTGGAATATTACAGAACGATCTAAAGAGGGTCATAGCTTATTC
Millettia pinnata           GCAACCACTGGAATATTACAGAACGATCTAAAGAGGGTCATAGCTTATTC
Brassica napus              GCAACCACTGGAATATTACAGAACGATCTAAAGAGGGTCATCGCTTATTC
Megaceros aenigmaticus      GCAACCACAGGAATATTACAGAACGATTTGAAGAGGGTCATAGCTTATTC
Phaeoceros laevis           GCAACCACAGGAATATTACAGAACGATTTGAAGAGGGTCATAGCTTATTT
                            ***** ** ****************** * *********** *******

Beta macrocarpa             AACTTGCAGTCAATTAGGCTATATGATCTTTGCTTGCGGCATTTCTAACT
Beta vulgaris vulgaris      AACTTGCAGTCAATTAGGCTATATGATCTTTGCTTGCGGCATTTCTAACT
Silene latifolia            AACTTGCAGTCAATTAGGCTATATGATCTTTGCTTGCGGCATTTCTAACT
RRIC110_nad5                AACTTGCAGTCAATTAGGCTATATGATCTTTGCTTGCGGCATCTCTAACT
PB235_nad5                  AACTTGCAGTCAATTAGGCTATATGATCTTTGCTTGCGGCATCTCTAACT
RRIT251_nad5                AACTTGCAGTCAATTAGGCTATATGATCTTTGCTTGCGGCATCTCTAACT
RRIM600_nad5                AACTTGCAGTCAATTAGGCTATATGATCTTTGCTTGCGGCATCTCTAACT
RRII105_nad5                AACTTGCAGTCAATTAGGCTATATGATCTTTGCTTGCGGCATCTCTAACT
BPM24_nad5                  AACTTGCAGTCAATTAGGCTATATGATCTTTGCTTGCGGCATCTCTAACT
Citrullus lanatus           AACTTGCAGTCAATTAGGCTATATGATCTTTGCTTGCGGCATCTCTAACT
Cucurbita pepo              AACTTGCAGTCAATTAGGCTATATGATCTTTGCTTGCGGCATCTCTAACT
Lotus japonicus             AACTTGCAGTCAATTAGGCTATATGATCTTTGCTTGCGGCATCTCTAACT
Millettia pinnata           AACTTGCAGTCAATTAGGCTATATGATCTTTGCTTGCGGCATCTCTAACT
Brassica napus              AACTTGTAGTCAATTAGGCTATATGATCTTTGCTTGCGGCATCTCTAACT
Megaceros aenigmaticus      CATTTGTAGTCAATCAGGCTATATGATCTTCGCTTGCGGCATTTCCAACT
Phaeoceros laevis           CATTTGTAGTCAATTAGGCTATATGATCTTTGCTTGCGGCATTTTCAACT
                             * *** ******* *************** *********** *  ****

Beta macrocarpa             ATTCGGTTAGCGTCTTTCATTTAATGAATCACGCCTTTTTCAAAGCATTA
Beta vulgaris vulgaris      ATTCGGTTAGCGTCTTTCATTTAATGAATCACGCCTTTTTCAAAGCATTA
Silene latifolia            ATTCGGTTAGCGTCTTTCATTTAATGAATCACGCCTTTTTCAAAGCATTA
RRIC110_nad5                ATTCGGTTAGCGTCTTTCACTTAATGAATCACGCGTTTTTCAAAGCATTA
PB235_nad5                  ATTCGGTTAGCGTCTTTCACTTAATGAATCACGCGTTTTTCAAAGCATTA
RRIT251_nad5                ATTCGGTTAGCGTCTTTCACTTAATGAATCACGCGTTTTTCAAAGCATTA
```

```
RRIM600_nad5                      ATTCGGTTAGCGTCTTTCACTTAATGAATCACGCGTTTTTCAAAGCATTA
RRII105_nad5                      ATTCGGTTAGCGTCTTTCACTTAATGAATCACGCGTTTTTCAAAGCATTA
BPM24_nad5                        ATTCGGTTAGCGTCTTTCACTTAATGAATCACGCGTTTTTCAAAGCATTA
Citrullus lanatus                 ATTCGGTTAGCGTCTTTCACTTAATGAATCACGCGTTTTTCAAAGCATTA
Cucurbita pepo                    ATTCGGTTAGCGTCTTTCACTTAATGAATCACGCCTTTTTCAAAGCATTA
Lotus japonicus                   ATTCGGTTAGCGTCTTTCACTTAATGAATCACGCCTTTTTCAAAGCATTA
Millettia pinnata                 ATTCGGTTAGCGTCTTTCACTTAATGAATCACGCCTTTTTCAAAGCATTA
Brassica napus                    ATTCGGTTAGCGTCTTTCACTTAATGAATCACGCCTTTTTCAAAGCATTA
Megaceros aenigmaticus            ATTCTGTTAGCGTATTTCACTTGATGAATCATGCTTTTCTTAAAGCATCA
Phaeoceros laevis                 ATTCTGTTAGCGTATTTCACTTGATGAATTATGCTTTTTTTAAAGTATTA
                                  **** ******** ***** ** ****** * ** *** * **** ** *

Beta macrocarpa                   CTATTCCTGAGTGCAGGTTCGGTGATTCATGCCATGTCGGATGAGCAAGA
Beta vulgaris vulgaris            CTATTCCTGAGTGCAGGTTCGGTGATTCATGCCATGTCGGATGAGCAAGA
Silene latifolia                  CTATTCCTGAGTGCGGGTTCGGTGATTCATGCCATGTCGGATGAGCAAGA
RRIC110_nad5                      CTCTTCCTGAGTGCAGGTTCGGTGATTCATGCCATGTCGGATGAGCAAGA
PB235_nad5                        CTCTTCCTGAGTGCAGGTTCGGTGATTCATGCCATGTCGGATGAGCAAGA
RRIT251_nad5                      CTCTTCCTGAGTGCAGGTTCGGTGATTCATGCCATGTCGGATGAGCAAGA
RRIM600_nad5                      CTCTTCCTGAGTGCAGGTTCGGTGATTCATGCCATGTCGGATGAGCAAGA
RRII105_nad5                      CTCTTCCTGAGTGCAGGTTCGGTGATTCATGCCATGTCGGATGAGCAAGA
BPM24_nad5                        CTCTTCCTGAGTGCAGGTTCGGTGATTCATGCCATGTCGGATGAGCAAGA
Citrullus lanatus                 CTATTCCTGAGTGCAGGTTCGGTGATTCATGCCATGTCGGATGAGCAAGA
Cucurbita pepo                    CTATTCCTGAGTGCTGGTTCGGTCATTCATGCCATGTCGGATGAGCAAGA
Lotus japonicus                   CTATTCCTGAGTGCAGGTTCGGTGATTCATGCCATGTCGGATGAGCAAGA
Millettia pinnata                 CTATTCCTGAGTGCAGGTTCGGTGATTCATGCCATGTCGGATGAGCAAGA
Brassica napus                    CTATTCCTGAGTGCTGGTTCGGTGATTCATGCCATGTCGGATGAGCAAGA
Megaceros aenigmaticus            CCCTTTTTGAGTGCAGGTCCAGTGATTCATGCCATGTCGGATGAGCAAGA
Phaeoceros laevis                 CCCTCTTTGAGTGCAGGTCCAGTGATTCATGCCATGTCGGATGAGCAAGA
                                  *  *   ******* *** * ** **************************

Beta macrocarpa                   TATGCGGAAGATGGGGGGGCTCGCCTCCTCGTTCCCCTTTACCTATGCCA
Beta vulgaris vulgaris            TATGCGGAAGATGGGGGGGCTCGCCTCCTCGTTCCCCTTTACCTATGCCA
Silene latifolia                  TATGCGGAAGATGGGGGGGCTCGCCTCCTCGTTCCCTTTTACCTATGCCA
RRIC110_nad5                      TATGCGGAAGATGGGGGGGCTTGCCTCCTCGTTCCCTTTTACCTATGCCA
PB235_nad5                        TATGCGGAAGATGGGGGGGCTTGCCTCCTCGTTCCCTTTTACCTATGCCA
RRIT251_nad5                      TATGCGGAAGATGGGGGGGCTTGCCTCCTCGTTCCCTTTTACCTATGCCA
RRIM600_nad5                      TATGCGGAAGATGGGGGGGCTTGCCTCCTCGTTCCCTTTTACCTATGCCA
RRII105_nad5                      TATGCGGAAGATGGGGGGGCTTGCCTCCTCGTTCCCTTTTACCTATGCCA
BPM24_nad5                        TATGCGGAAGATGGGGGGGCTTGCCTCCTCGTTCCCTTTTACCTATGCCA
Citrullus lanatus                 TATGCGGAAGATGGGGGGGCTTGCCTCCTCGTTCCCTTTTACCTATGCCA
Cucurbita pepo                    TATGCGTAAGATGGGGGGGCTTGCCTCCTCGTTCCCTTTGACCTATGCCA
Lotus japonicus                   TATGCGGAAGATGGGGGGGCTTGCCTCCTCGTTCCCTTTTACCTATGCCA
Millettia pinnata                 TATGCGGAAGATGGGGGGGCTTGCCTCCTCGTTCCCTTTTACCTATGCCA
Brassica napus                    TATGCGGAAGATGGGGGGGCTTGCCTCCTCCTTCCCTTTGACCTATGCCA
Megaceros aenigmaticus            TATGCGTAAGATGGGAGGACTTGCTTCCCTGTTACCCTTTACCTATGCTA
Phaeoceros laevis                 TATGCGTAAGATGGGAGGACTTGCTTCCCTGTTACCCTTTACCTATGCTA
                                  ****** ******** ** ** ** ***   ** ** ** ******** *

Beta macrocarpa                   TGATGCTCATGGGCAGCTTATCTCTAATTGGATTTCCTTTTCTAACTGGA
Beta vulgaris vulgaris            TGATGCTCATGGGCAGCTTATCTCTAATTGGATTTCCTTTTCTAACTGGA
Silene latifolia                  TGATGCTCATGGGCAGCTTATCTCTAATTGGATTTCCTTTTCTAACTGGA
RRIC110_nad5                      TGATGCTCATAGGCAGCTTATCTCTAATTGGATTTCCTTTTCTAACTGGA
PB235_nad5                        TGATGCTCATAGGCAGCTTATCTCTAATTGGATTTCCTTTTCTAACTGGA
RRIT251_nad5                      TGATGCTCATAGGCAGCTTATCTCTAATTGGATTTCCTTTTCTAACTGGA
RRIM600_nad5                      TGATGCTCATAGGCAGCTTATCTCTAATTGGATTTCCTTTTCTAACTGGA
RRII105_nad5                      TGATGCTCATAGGCAGCTTATCTCTAATTGGATTTCCTTTTCTAACTGGA
BPM24_nad5                        TGATGCTCATAGGCAGCTTATCTCTAATTGGATTTCCTTTTCTAACTGGA
Citrullus lanatus                 TGATGCTCATGGGCAGCTTATCTCTAATTGGATTTCCTTTTCTAACTGGA
Cucurbita pepo                    TGATGCTCATGGGCAGCTTATCTCTAATTGGATTTCCTTTTCTAACTGGA
Lotus japonicus                   TGATGCTCATGGGCAGCTTATCTCTAATTGGATTTCCTTTTCTAACTGGA
Millettia pinnata                 TGATGCTCATGGGCAGCTTATCTCTAATTGGATTTCCTTTTCTAACTGGA
Brassica napus                    TGATGCTCATAGGCAGCTTATCTCTAATTGGATTTCCTTTTCTAACTGGA
```

```
Megaceros aenigmaticus         TGATGCTTATAGGCAGCTTATCTCCAATCGGATTTCCTTTTTTAACTGGA
Phaeoceros laevis              TGATGCTTATAGGCAGCTTATCTCCAATCGGATTTCCTTTTTTAACTGGA
                               ******* ** ************* *** ************ ********

Beta macrocarpa                TTTTATTCCAAAGATGTGATCTTAGAGCTCGCTTACACTAAGTATACCAT
Beta vulgaris vulgaris         TTTTATTCCAAAGATGTGATCTTAGAGCTCGCTTACACTAAGTATACCAT
Silene latifolia               TTTTATTCCAAAGATGTGATCTTAGAGCTCGCTTACACTAAGTATACCAT
RRIC110_nad5                   TTTTATTCCAAAGATGTGATCTTAGAGCTCGCTTACACTAAGTATACCAT
PB235_nad5                     TTTTATTCCAAAGATGTGATCTTAGAGCTCGCTTACACTAAGTATACCAT
RRIT251_nad5                   TTTTATTCCAAAGATGTGATCTTAGAGCTCGCTTACACTAAGTATACCAT
RRIM600_nad5                   TTTTATTCCAAAGATGTGATCTTAGAGCTCGCTTACACTAAGTATACCAT
RRII105_nad5                   TTTTATTCCAAAGATGTGATCTTAGAGCTCGCTTACACTAAGTATACCAT
BPM24_nad5                     TTTTATTCCAAAGATGTGATCTTAGAGCTCGCTTACACTAAGTATACCAT
Citrullus lanatus              TTTTATTCCAAAGATGTGATCTTAGAGCTCGCTTACACTAAGTATACCAT
Cucurbita pepo                 TTTTATTCCAAAGATGTGATCTTAGAGCTCGCTTACACTAAGTATACCAT
Lotus japonicus                TTTTATTCCAAAGATGTGATATTAGAGCTCGCTTACACTAAGTATACCAT
Millettia pinnata              TTTTATTCCAAAGATGTGATATTAGAGCTCGCTTACACTAAGTATACCAT
Brassica napus                 TTTTATTCCAAAGATGTGATCTTAGAGCTCGCTTACACTAAGTATACCAT
Megaceros aenigmaticus         TTTTATTCCAAAGATGTGATTCTGGAGCCTGCTTACACTAAATATACAAT
Phaeoceros laevis              TTTTATTCCAAAGATGTGATTCTAGAGCCTGCTTACACTAAATATACAAT
                               ********************  * ****  *********** ***** **

Beta macrocarpa                CAGTGGGAACTTTGCTTTCTGGTTGGGAAGTGTCTCTGTCCTTTTCACTT
Beta vulgaris vulgaris         CAGTGGGAACTTTGCTTTCTGGTTGGGAAGTGTCTCTGTCCTTTTCACTT
Silene latifolia               CAGTGGGAACTTTGCTTTCTGGTTGGGAAGTGTCTCTGTCCTTTTCACTT
RRIC110_nad5                   CAGTGGGAACTTTGCTTTCTGGTTGGGAAGTGTCTCTGTCCTTTTCACTT
PB235_nad5                     CAGTGGGAACTTTGCTTTCTGGTTGGGAAGTGTCTCTGTCCTTTTCACTT
RRIT251_nad5                   CAGTGGGAACTTTGCTTTCTGGTTGGGAAGTGTCTCTGTCCTTTTCACTT
RRIM600_nad5                   CAGTGGGAACTTTGCTTTCTGGTTGGGAAGTGTCTCTGTCCTTTTCACTT
RRII105_nad5                   CAGTGGGAACTTTGCTTTCTGGTTGGGAAGTGTCTCTGTCCTTTTCACTT
BPM24_nad5                     CAGTGGGAACTTTGCTTTCTGGTTGGGAAGTGTCTCTGTCCTTTTCACTT
Citrullus lanatus              CAGTGGGAACTTTGCTTTCTGGTTGGGAAGTGTCTCTGTCCTTTTCACTT
Cucurbita pepo                 CAGTGGGAACTTTGCTTTCTGGTTGGGAAGTGTCTCTGTCCTTTTCACTT
Lotus japonicus                AAGTGGTAATTTTGCTTTCTGGTTGGGAAGTGTCTCTGTCCTTTTCACTT
Millettia pinnata              AAGTGGGAATTTTGCTTTCTGGTTGGGAAGTGTCTCTGTCCTTTTCACTT
Brassica napus                 CAGTGGGAACTTTGCTTTCTGGTTGGGAAGTATTTCTGTCCTTTTCACTT
Megaceros aenigmaticus         TAGTGGTAACTCTGCTTTCTGGTTGGGAAGTGTCTCTGTCTTCTTCACTT
Phaeoceros laevis              TAGTGGTAACTTTGCTTTCTGGTTGGGAAGTGTCTCTGTCTTCTTCACTT
                                ***** ** * ****************** * ****** * *******

Beta macrocarpa                CTTATTACTCTTTTCGTTTACTTTTTCTAACATTTCTAGTACCAACTAAT
Beta vulgaris vulgaris         CTTATTACTCTTTTCGTTTACTTTTTCTAACATTTCTAGTACCAACTAAT
Silene latifolia               CTTATTACTCTTTTCGTTUACTTTTTCTAACATTTCTAGTACCAACTAAT
RRIC110_nad5                   CTTATTACTCTTTTCGTTUACTTTTTCTAACATTTCTAGTACCAACTAAT
PB235_nad5                     CTTATTACTCTTTTCGTTUACTTTTTCTAACATTTCTAGTACCAACTAAT
RRIT251_nad5                   CTTATTACTCTTTTCGTTUACTTTTTCTAACATTTCTAGTACCAACTAAT
RRIM600_nad5                   CTTATTACTCTTTTCGTTNACTTTTTCTAACATTTCTAGTACCAACTAAT
RRII105_nad5                   CTTATTACTCTTTTCGTTUACTTTTTCTAACATTTCTAGTACCAACTAAT
BPM24_nad5                     CTTATTACTCTTTTCGTTUACTTTTTCTAACATTTCTAGTACCAACTAAT
Citrullus lanatus              CTTATTACTCTTTTCGTTUACTTTTTCTAACATTTCTAGTACCAACTAAT
Cucurbita pepo                 CTTATTACTCTTTTCGTTUACTTTTTCTAACATTTCTAGTACCAACTAAT
Lotus japonicus                CTTATTACTCTTTTCGTTCACTTTTTCTAACATTTCTAGTACCAACTAAT
Millettia pinnata              CTTATTACTCTTTTCGTTCACTTTTTCTAACATTTCTAGTACCAACTAAT
Brassica napus                 CTTATTACTCTTTTCGTTTACTTTTTCTCACATTTCTAGTACCAACTAAT
Megaceros aenigmaticus         CTTACCACTTCTTTCGCTTACTCTTCTTAACATTCTTAGCACCAACCAAT
Phaeoceros laevis              CTTACCACTTCTTTCGCTTACTTTTCTTAACATTTTTAGCACCAACTAAT
                               ****  ***  ***** * *** **  * *****  *** ****** ***

Beta macrocarpa                TCATTCGGGCGAGACATCTTACGATGTCATGATGCGCCCATTCCTATGGC
Beta vulgaris vulgaris         TCATTCGGGCGAGACATCTTACGATGTCATGATGCGCCCATTCCTATGGC
Silene latifolia               TCATTCGGGCGAGACATCTTACGATGTCATGATGCGCCCATTCCTATGGC
RRIC110_nad5                   TCATTCGGGCGAGACATCGTAAGATGTCATGATGCGCCCATTCCTATGGC
```

```
PB235_nad5                  TCATTCGGGCGAGACATCGTAAGATGTCATGATGCGCCCATTCCTATGGC
RRIT251_nad5                TCATTCGGGCGAGACATCGTAAGATGTCATGATGCGCCCATTCCTATGGC
RRIM600_nad5                TCATTCGGGCGAGACATCGTAAGATGTCATGATGCGCCCATTCCTATGGC
RRII105_nad5                TCATTCGGGCGAGACATCGTAAGATGTCATGATGCGCCCATTCCTATGGC
BPM24_nad5                  TCATTCGGGCGAGACATCGTAAGATGTCATGATGCGCCCATTCCTATGGC
Citrullus lanatus           TCATTCGGGCGAGACATCTTACGATGTCATGATGCGCCCATTCCTATGGC
Cucurbita pepo              TCATTCGGGCGAGACATCGTCAGATGCCATGATGCGCCCATTCCTATGGC
Lotus japonicus             TCATTCGGGCGAGACATCGTAAGATGTCATGATGCGCCCATTCCTATGGC
Millettia pinnata           TCATTCGGGCGAGACATCTTACGATGTCATGATGCGCCCATTCCTATGGC
Brassica napus              TCGTTCGGGCGAGACATCTCACGATGTCATGATGCGCCCATTCCTATGGC
Megaceros aenigmaticus      TCATTCAAGCAAGACATGTTACGATGTCATGATGCGCCCATTCTCATGGC
Phaeoceros laevis           TCATTCAAGCGAGACATGTTACGATGTCATGATGCGCCCATTCTCATGGC
                            ** ***  ** ******     **** ****************  *****

Beta macrocarpa             CATTCCTTTAATACTTCTGGCTTTCGGGAGTCTCTTTGTAGGATACTTGG
Beta vulgaris vulgaris      CATTCCTTTAATACTTCTGGCTTTCGGGAGTCTCTTTGTAGGATACTTGG
Silene latifolia            CATTCCTTTAATACTTCTGGCTTTCGGGAGTCTCTTTGTAGGATACTTGG
RRIC110_nad5                CATTCCTTTAATACTTCTGGCTCTCGGGAGTCTCTTTGTAGGATACTTGG
PB235_nad5                  CATTCCTTTAATACTTCTGGCTCTCGGGAGTCTCTTTGTAGGATACTTGG
RRIT251_nad5                CATTCCTTTAATACTTCTGGCTCTCGGGAGTCTCTTTGTAGGATACTTGG
RRIM600_nad5                CATTCCTTTAATACTTCTGGCTCTCGGGAGTCTCTTTGTAGGATACTTGG
RRII105_nad5                CATTCCTTTAATACTTCTGGCTCTCGGGAGTCTCTTTGTAGGATACTTGG
BPM24_nad5                  CATTCCTTTAATACTTCTGGCTCTCGGGAGTCTCTTTGTAGGATACTTGG
Citrullus lanatus           CATTCCTTUAATACTTCTGGCTCTCGGGAGTCTCTTTGTAGGATACTTGG
Cucurbita pepo              CATTCCTTTAATCCTTCTGGCTCTCGGGAGTCTCTTTGTAGGATACTTGG
Lotus japonicus             CATTCCTTTAATACTTCTGGCTCTCGGGAGTCTCTTTGTAGGATACTTGG
Millettia pinnata           CATTCCTTTAATACTTCTGGCTCTCGGGAGTCTCTTTGTAGGATACTTGG
Brassica napus              CATTCCTTUAATACTTCTGGCTCTCGGGAGTCTCTTTGTAGGATACTTGG
Megaceros aenigmaticus      AATCCCTTTAATCTGTTTGGCTTTTGGAAGTATTTCTGTAGGACACTTGG
Phaeoceros laevis           AATCCTTTTAATCTGTTTGGCTTTTGGAAGTATTTTTGTAGGATACTTGG
                             ** * ** ***   * ***** * ** *** * * ******* ******

Beta macrocarpa             CCAAA---------------------------------------------
Beta vulgaris vulgaris      CCAAA---------------------------------------------
Silene latifolia            CCAAA---------------------------------------------
RRIC110_nad5                CCAAAGATATGATGATTGGTTTAGGTACCAATTTTTGGGCCAATTCCCUC
PB235_nad5                  CCAAAGATATGATGATTGGTTTAGGTACCAATTTTTGGGCCAATTCCCUC
RRIT251_nad5                CCAAAGATATGATGATTGGTTTAGGTACCAATTTTTGGGCCAATTCCCNC
RRIM600_nad5                CCAAAGATATGATGATTGGTTTAGGTACCAATTTTTGGGCCAATTCCCNC
RRII105_nad5                CCAAAGATATGATGATTGGTTTAGGTACCAATTTTTGGGCCAATTCCCUC
BPM24_nad5                  CCAAAGATATGATGATTGGTTTAGGTACCAATTTTTGGGCCAATTCCCUC
Citrullus lanatus           CCAAA---------------------------------------------
Cucurbita pepo              CCAAA---------------------------------------------
Lotus japonicus             CCAAA---------------------------------------------
Millettia pinnata           CCAAA---------------------------------------------
Brassica napus              CCAAA---------------------------------------------
Megaceros aenigmaticus      CCAAAGATATGATGATTGGCTTAGGTACTAATTTTTGGGCTAATTCTGTT
Phaeoceros laevis           CCAAAGATATGATGATTGGCTTAGGTACTAATTTTTGGGCCAATTTTCTT
                            *****

Beta macrocarpa             --------------------------------------------------
Beta vulgaris vulgaris      --------------------------------------------------
Silene latifolia            --------------------------------------------------
RRIC110_nad5                TTTGTACTACCAAAAAAATGAGATTCTTGCCGAATCCGAGTTTGCTGCTCC
PB235_nad5                  TTTGTACTACCAAAAAAATGAGATTCTTGCCGAATCCGAGTTTGCTGCTCC
RRIT251_nad5                TTTGTACTACCAAAAAAATGAGATTCTTGCCGAATCCGAGTTTGCTGCTCC
RRIM600_nad5                TTTGTACTACCAAAAAAATGAGATTCTTGCCGAATCCGAGTTTGCTGCTCC
RRII105_nad5                TTTGTACTACCAAAAAAATGAGATTCTTGCCGAATCCGAGTTTGCTGCTCC
BPM24_nad5                  TTTGTACTACCAAAAAAATGAGATTCTTGCCGAATCCGAGTTTGCTGCTCC
Citrullus lanatus           --------------------------------------------------
Cucurbita pepo              --------------------------------------------------
Lotus japonicus             --------------------------------------------------
```

```
Millettia pinnata                 --------------------------------------------------
Brassica napus                    --------------------------------------------------
Megaceros aenigmaticus            TTCATATCACCCAAAAATGAGATTCTTGCTGAATCTGAGTTTGCTACTCC
Phaeoceros laevis                 TTCATATCACCCAAAAATGAGATTCTTGCTGAATCTGAGTTTGCTACTCC

Beta macrocarpa                   --------------------------------------------------
Beta vulgaris vulgaris            --------------------------------------------------
Silene latifolia                  --------------------------------------------------
RRIC110_nad5                      AACCATTAUCAAACTAATACCTATTCCGTTTAGTACTTCAGGTGCTTNTG
PB235_nad5                        AACCATTAUCAAACTAATACCTATTCCGTTTAGTACTTCAGGTGCTTNTG
RRIT251_nad5                      AACCATTAUCAAACTAATACCTATTCCGTTTAGTACTTCAGGTGCTTUTG
RRIM600_nad5                      AACCATTAUCAAACTAATACCTATTCCGTTTAGTACTTCAGGTGCTTNTG
RRII105_nad5                      AACCATTAUCAAACTAATACCTATTCCGTTTAGTACTTCAGGTGCTTUTG
BPM24_nad5                        AACCATTAUCAAACTAATACCTATTCCGTTTAGTACTTCAGGTGCTTUTG
Citrullus lanatus                 --------------------------------------------------
Cucurbita pepo                    --------------------------------------------------
Lotus japonicus                   --------------------------------------------------
Millettia pinnata                 --------------------------------------------------
Brassica napus                    --------------------------------------------------
Megaceros aenigmaticus            AACCATTATAAAACTAATTCCTATCCTGTTTAGTACTTTAGGCGCATTTG
Phaeoceros laevis                 AACCATTATCAAACTAATTCCTATTCTGTTTAGTACTTTAGGCGCATTTG

Beta macrocarpa                   --------------------------------------------------
Beta vulgaris vulgaris            --------------------------------------------------
Silene latifolia                  --------------------------------------------------
RRIC110_nad5                      TTGCGTATAATGTAAATCUCGTAGCGGATCAATTCCAACAAGCCTTTCAA
PB235_nad5                        TTGCGTATAATGTAAATCUCGTAGCGGATCAATTCCAACAAGCCTTTCAA
RRIT251_nad5                      TTGCGTATAATGTAAATCUCGTAGCGGATCAATTCCAACAAGCCTTTCAA
RRIM600_nad5                      TTGCGTATAATGTAAATCNCGTAGCGGATCAATTCCAACAAGCCTTTCAA
RRII105_nad5                      TTGCGTATAATGTAAATCUCGTAGCGGATCAATTCCAACAAGCCTTTCAA
BPM24_nad5                        TTGCGTATAATGTAAATCUCGTAGCGGATCAATTCCAACAAGCCTTTCAA
Citrullus lanatus                 --------------------------------------------------
Cucurbita pepo                    --------------------------------------------------
Lotus japonicus                   --------------------------------------------------
Millettia pinnata                 --------------------------------------------------
Brassica napus                    --------------------------------------------------
Megaceros aenigmaticus            TGGCGTATAATGTCAATTTTATAGCAAATGAATTTATCTTCGCTTTGAAA
Phaeoceros laevis                 TGGCGTATAATGTAAATTTTATAGCAAATGAATTTATCTTCGCTTTGAAA

Beta macrocarpa                   --------------------------------------------------
Beta vulgaris vulgaris            --------------------------------------------------
Silene latifolia                  --------------------------------------------------
RRIC110_nad5                      ACTAGTACTTTTTGTAATCGACTCTATAGCTTCTTCAATAAACGCTGGTT
PB235_nad5                        ACTAGTACTTTTTGTAATCGACTCTATAGCTTCTTCAATAAACGCTGGTT
RRIT251_nad5                      ACTAGTACTTTTTGTAATCGACTCTATAGCTTCTTCAATAAACGCTGGTT
RRIM600_nad5                      ACTAGTACTTTTTGTAATCGACTCTATAGCTTCTTCAATAAACGCTGGTT
RRII105_nad5                      ACTAGTACTTTTTGTAATCGACTCTATAGCTTCTTCAATAAACGCTGGTT
BPM24_nad5                        ACTAGTACTTTTTGTAATCGACTCTATAGCTTCTTCAATAAACGCTGGTT
Citrullus lanatus                 --------------------------------------------------
Cucurbita pepo                    --------------------------------------------------
Lotus japonicus                   --------------------------------------------------
Millettia pinnata                 --------------------------------------------------
Brassica napus                    --------------------------------------------------
Megaceros aenigmaticus            ACAACTTCTTTTGGTAATGGACTATATTGCTTCTTAAATAAGCGTTGGTT
Phaeoceros laevis                 ACAACCTCTTTTGGTAATGGACTATATTGCTTCTTAAATAAGCGTTGGTT

Beta macrocarpa                   --------------------------------------------------
Beta vulgaris vulgaris            --------------------------------------------------
```

```
Silene latifolia                  --------------------------------------------------
RRIC110_nad5                      CTTCGATCAAGTTTTGAATGACTTTCTAGTCAGATCCTTCCTGCGTTTCG
PB235_nad5                        CTTCGATCAAGTTTTGAATGACTTTCTAGTCAGATCCTTCCTGCGTTTCG
RRIT251_nad5                      CTTCGATCAAGTTTTGAATGACTTTCTAGTCAGATCCTTCCTGCGTTTCG
RRIM600_nad5                      CTTCGATCAAGTTTTGAATGACTTTCTAGTCAGATCCTTCCTGCGTTTCG
RRII105_nad5                      CTTCGATCAAGTTTTGAATGACTTTCTAGTCAGATCCTTCCTGCGTTTCG
BPM24_nad5                        CTTCGATCAAGTTTTGAATGACTTTCTAGTCAGATCCTTCCTGCGTTTCG
Citrullus lanatus                 --------------------------------------------------
Cucurbita pepo                    --------------------------------------------------
Lotus japonicus                   --------------------------------------------------
Millettia pinnata                 --------------------------------------------------
Brassica napus                    --------------------------------------------------
Megaceros aenigmaticus            CTTTGATAAAGTCTTTAATGACTTCATAGTGAGATCTTTTTTACGTTTTG
Phaeoceros laevis                 CTTTGATAAAGTGTTTAATGACTTCATAGTGAGATCCTTTTTACGTTTTG

Beta macrocarpa                   --------------------------------------------------
Beta vulgaris vulgaris            --------------------------------------------------
Silene latifolia                  --------------------------------------------------
RRIC110_nad5                      GATATGAAGTCTCATTCGAAGCTTTAGACAAAGGTGCTATTGAGATATTG
PB235_nad5                        GATATGAAGTCTCATTCGAAGCTTTAGACAAAGGTGCTATTGAGATATTG
RRIT251_nad5                      GATATGAAGTCTCATTCGAAGCTTTAGACAAAGGTGCTATTGAGATATTG
RRIM600_nad5                      GATATGAAGTCTCATTCGAAGCTTTAGACAAAGGTGCTATTGAGATATTG
RRII105_nad5                      GATATGAAGTCTCATTCGAAGCTTTAGACAAAGGTGCTATTGAGATATTG
BPM24_nad5                        GATATGAAGTCTCATTCGAAGCTTTAGACAAAGGTGCTATTGAGATATTG
Citrullus lanatus                 --------------------------------------------------
Cucurbita pepo                    --------------------------------------------------
Lotus japonicus                   --------------------------------------------------
Millettia pinnata                 --------------------------------------------------
Brassica napus                    --------------------------------------------------
Megaceros aenigmaticus            GATATGAAGTGTCATTTAAAGCTTTAGACAAGGGTGCTATTGAAATCTTG
Phaeoceros laevis                 GATATGAAGTGTCATTTAAAGCTTTAGACAAGGGTGCTATTGAAATCTTG

Beta macrocarpa                   --------------------------------------------------
Beta vulgaris vulgaris            --------------------------------------------------
Silene latifolia                  --------------------------------------------------
RRIC110_nad5                      GGCCCTTATGGTATCTCGTACACATTCCGACGATTGGCCGAGCGAATAAG
PB235_nad5                        GGCCCTTATGGTATCTCGTACACATTCCGACGATTGGCCGAGCGAATAAG
RRIT251_nad5                      GGCCCTTATGGTATCTCGTACACATTCCGACGATTGGCCGAGCGAATAAG
RRIM600_nad5                      GGCCCTTATGGTATCTCGTACACATTCCGACGATTGGCCGAGCGAATAAG
RRII105_nad5                      GGCCCTTATGGTATCTCGTACACATTCCGACGATTGGCCGAGCGAATAAG
BPM24_nad5                        GGCCCTTATGGTATCTCGTACACATTCCGACGATTGGCCGAGCGAATAAG
Citrullus lanatus                 --------------------------------------------------
Cucurbita pepo                    --------------------------------------------------
Lotus japonicus                   --------------------------------------------------
Millettia pinnata                 --------------------------------------------------
Brassica napus                    --------------------------------------------------
Megaceros aenigmaticus            GGTCCTTATGGGATTTCGTACACATTTGGAGAATTAGCTAAGCAAATAAG
Phaeoceros laevis                 GGTCCTTATGGGATTTCGTACACATTTGGAGAATTAGCTAAGCAAATAAG

Beta macrocarpa                   --------------------------------------------------
Beta vulgaris vulgaris            --------------------------------------------------
Silene latifolia                  --------------------------------------------------
RRIC110_nad5                      TCAACTTCAAAGTGGATTTGTTTATCATTATGCCTTTGCAATGTTACTTG
PB235_nad5                        TCAACTTCAAAGTGGATTTGTTTATCATTATGCCTTTGCAATGTTACTTG
RRIT251_nad5                      TCAACTTCAAAGTGGATTTGTTTATCATTATGCCTTTGCAATGTTACTTG
RRIM600_nad5                      TCAACTTCAAAGTGGATTTGTTTATCATTATGCCTTTGCAATGTTACTTG
RRII105_nad5                      TCAACTTCAAAGTGGATTTGTTTATCATTATGCCTTTGCAATGTTACTTG
BPM24_nad5                        TCAACTTCAAAGTGGATTTGTTTATCATTATGCCTTTGCAATGTTACTTG
Citrullus lanatus                 --------------------------------------------------
```

```
Cucurbita pepo                    --------------------------------------------------
Lotus japonicus                   --------------------------------------------------
Millettia pinnata                 --------------------------------------------------
Brassica napus                    --------------------------------------------------
Megaceros aenigmaticus            TAAACTTCAAAGTGGATTTGTTTATCATTACGCTTTCGCTATGCTGATTG
Phaeoceros laevis                 TAAACTTCAAAGTGGATTTGTTTATCATTACGCTTTCGCTAT--------


Beta macrocarpa                   --------------------------------------------------
Beta vulgaris vulgaris            --------------------------------------------------
Silene latifolia                  --------------------------------------------------
RRIC110_nad5                      GTTCAACTCTATTTGTGACCTTTTCTCGTATGTGGGACTCTCTATCTTCT
PB235_nad5                        GTTCAACTCTATTTGTGACCTTTTCTCGTATGTGGGACTCTCTATCTTCT
RRIT251_nad5                      GTTCAACTCTATTTGTGACCTTTTCTCGTATGTGGGACTCTCTATCTTCT
RRIM600_nad5                      GTTCAACTCTATTTGTGACCTTTTCTCGTATGTGGGACTCTCTATCTTCT
RRII105_nad5                      GTTCAACTCTATTTGTGACCTTTTCTCGTATGTGGGACTCTCTATCTTCT
BPM24_nad5                        GTTCAACTCTATTTGTGACCTTTTCTCGTATGTGGGACTCTCTATCTTCT
Citrullus lanatus                 --------------------------------------------------
Cucurbita pepo                    --------------------------------------------------
Lotus japonicus                   --------------------------------------------------
Millettia pinnata                 --------------------------------------------------
Brassica napus                    --------------------------------------------------
Megaceros aenigmaticus            GTTTAACCATATTTATTACTATTATAGGTCTGTGGGATTTTCTCTCCTTT
Phaeoceros laevis                 --------------------------------------------------


Beta macrocarpa                   --------------------------------------------------
Beta vulgaris vulgaris            --------------------------------------------------
Silene latifolia                  --------------------------------------------------
RRIC110_nad5                      TGGGTAGATAATCGATCGTCTTTCATTTTGATAGTGAGTACTTTTTATAA
PB235_nad5                        TGGGTAGATAATCGATCGTCTTTCATTTTGATAGTGAGTACTTTTTATAA
RRIT251_nad5                      TGGGTAGATAATCGATCGTCTTTCATTTTGATAGTGAGTACTTTTTATAA
RRIM600_nad5                      TGGGTAGATAATCGATCGTCTTTCATTTTGATAGTGAGTACTTTTTATAA
RRII105_nad5                      TGGGTAGATAATCGATCGTCTTTCATTTTGATAGTGAGTACTTTTTATAA
BPM24_nad5                        TGGGTAGATAATCGATCGTCTTTCATTTTGATAGTGAGTACTTTTTATAA
Citrullus lanatus                 --------------------------------------------------
Cucurbita pepo                    --------------------------------------------------
Lotus japonicus                   --------------------------------------------------
Millettia pinnata                 --------------------------------------------------
Brassica napus                    --------------------------------------------------
Megaceros aenigmaticus            TGGGTAGATAATAGATTGTATTTTATTTACACAGTGACTTTCTCTTTTAT
Phaeoceros laevis                 --------------------------------------------------


Beta macrocarpa                   -------------------------------
Beta vulgaris vulgaris            -------------------------------
Silene latifolia                  -------------------------------
RRIC110_nad5                      TAATAAGTTAAGTCAAGAATAA---------
PB235_nad5                        TAATAAGTTAAGTCAAGAATAA---------
RRIT251_nad5                      TAATAAGTTAAGTCAAGAATAA---------
RRIM600_nad5                      TAATAAGTTAAGTCAAGAATAA---------
RRII105_nad5                      TAATAAGTTAAGTCAAGAATAA---------
BPM24_nad5                        TAATAAGTTAAGTCAAGAATAA---------
Citrullus lanatus                 -------------------------------
Cucurbita pepo                    -------------------------------
Lotus japonicus                   -------------------------------
Millettia pinnata                 -------------------------------
Brassica napus                    -------------------------------
Megaceros aenigmaticus            TAATCTTGAGGGAGACATTCGCACAAATTAA
Phaeoceros laevis                 -------------------------------
```

```
###################
```

nad6 multiple sequence alignment

```
Arabidopsis thaliana           --TAGGAACAACCGCGCTGGTU----GTAATAGATCGACTTTCATGCTGA
Brassica napus                 AATAGGAACAACCGCGCTGGTC----GTAATAGATCGACTTTCATGCTGA
RRII105_nad6                   --------------------------------------------------
Beta macrocarpa                --------CAACCGCGCTGGTCGTACGTAATAGATCGACTTTCATGCTAG
Beta vulgaris maritima         --------CAACCGCGCTGGTCGTACGTAATAGATCGACTTTCATGCTAG
Beta vulgaris vulgaris         --------CAACCGCGCTGGTCGTACGTAATAGATCGACTTTCATGCTAG
Silene latifolia               --------CAACCGCGCTGGTCGTACGTAATAGATCGACTTTCATGCTAG
Oryza sativa Japonica          -ATACGAACAACCGCGCTGGTC----GTAATAGATCGACTTGAATGCGTC
RRIC110_nad6                   --------------------------------------------------
Millettia pinnata              -------------CTGTTGGTCA---ACAACCAAGCACAGCTCTATATAG
BPM24_nad6                     --------------------------------------------------
Lotus japonicus                -----------CCGCGCTGGTCG----TAATAGATCGACTTTCATGCTAG
PB235_nad6                     --------------------------------------------------
RRIT251_nad6                   --------------------------------------------------
RRIM600_nad6                   --------------------------------------------------
Cycas taitungensis             -------ACAACCGGGACCGCTGGTCGTAATAGATCAACTCGAATGCCAG
Zea mays mays                  ------CCAATATTTATCTTTTGTTTGGAAAAACCAAACCCACATATTGA
Megaceros aenigmaticus         --------------------------------------------------
Phaeoceros laevis              --------------------------------------------------
Pleurozia purpurea             --------------------------TTGGAAAATTTCGCTTTCTGCCGA


Arabidopsis thaliana           AGCAAGA-ACTAG-----CATGAAAGTTCC----ATTTCAGGGAAGGACG
Brassica napus                 AGCAAGA-ACTAG-----CATGAAAGTTCC----ATTTCAGGGAAGGACG
RRII105_nad6                   --------------------------------------------------
Beta macrocarpa                TTCTTAC-TCCAAACCAGCATGAAAGTTCC----ATTTCAGTGAAGGACG
Beta vulgaris maritima         TTCTTAC-TCCAAACCAGCATGAAAGTTCC----ATTTCAGTGAAGGACG
Beta vulgaris vulgaris         TTCTTAC-TCCAAACCAGCATGAAAGTTCC----ATTTCAGTGAAGGACG
Silene latifolia               TTCTTGC-TCCAAACCAGCATGAAAGTTCC----ATTTCAGTGAAGGACG
Oryza sativa Japonica          TTCTTGC-TCCAA-----CATTCAAGTTCC----ATTTCAAGGGAGGACG
RRIC110_nad6                   --------------------------------------------------
Millettia pinnata              TCAAAAC-ACTAGT----CCTACAGGCTTTCGGAGTTCAGTCGGAATCA
BPM24_nad6                     --------------------------------------------------
Lotus japonicus                TTCTTGC-TCCAG-----CATGAAAGTTCC----ATTTCAGGGAAGGCTT
PB235_nad6                     --------------------------------------------------
RRIT251_nad6                   --------------------------------------------------
RRIM600_nad6                   --------------------------------------------------
Cycas taitungensis             TTCTTGC-TCCAG-----CATGAAAGTGAC----ATTTTGGGGAAGGACG
Zea mays mays                  TCTTTAAGTCTCCCTTTCTCTTTTGGGAGCAGATCTTTCAAAGATGGGAA
Megaceros aenigmaticus         --------------------------------------------------
Phaeoceros laevis              --------------------------------------------------
Pleurozia purpurea             CGCGGTTCGCAGCAAAAATATAGATTTTTTGTTCACAGCTAATAAAATAA


Arabidopsis thaliana           ACGGTA---------CCATGATACTTTCT---GTTTTGTCGAGCCUTGCT
Brassica napus                 ACGTAC---------CCATGATACTTTCT---GTTTTGTCGAGCCUTGCT
RRII105_nad6                   -----------------ATGATACTTTCT---GTTTTGTCGAGCCUTGCT
Beta macrocarpa                ACGTA----------CCATGATACTTTCT---GTTTTGTCGAGCCUGGCT
Beta vulgaris maritima         ACGTA----------CCATGATACTTTCT---GTTTTGTCGAGCCUGGCT
Beta vulgaris vulgaris         ACGTA----------CCATGATACTTTCT---GTTTTGTCGAGCCUGGCT
Silene latifolia               ACGTA----------CCATGATACTTTCT---GTTTTGTCGAGCCUAGCT
Oryza sativa Japonica          ACGTA----------CCATGATACTTTCA---GTTTTGTCTAGCUUTGCT
RRIC110_nad6                   -----------------ATGATACTTTCT---GTTTTGTCGAGCCUTGCT
Millettia pinnata              TTTCTTCTCAA--TGCCATGATACTTTCT---GTTTTGTCGAGCCUTGCT
BPM24_nad6                     -----------------ATGATACTTTCT---GTTTTGTCGAGCCUTGCT
Lotus japonicus                TTTGGACTAAGGGTACCATGATACTTTCT---GTTTTGTCGAGCCUTGCT
PB235_nad6                     -----------------ATGATACTTTCT---GTTTTGTCGAGCCUTGCT
RRIT251_nad6                   -----------------ATGATACTTTCT---GTTTTGTCGAGCCNTGCT
RRIM600_nad6                   -----------------ATGATACTTTCT---GTTTTGTCGAGCCNTGCT
Cycas taitungensis             ACGTA----------CTAUGATACTTTCT---GTTTCGTCGAGCCCTGCT
```

```
Zea mays mays                     ATTC-----------CAATGATTCTTTCC---GTTTTGTCGAGCUUTGCT
Megaceros aenigmaticus            -----------------ATGATACTTTTTTCTGTTCCTTCGAGCATTGCT
Phaeoceros laevis                 -----------------ATGATACTTTTTTCTGTTCCTTCGAGCATTGCT
Pleurozia purpurea                AAGGGGAAAATTTCACCAUGATACTTTTTTATGTTTTTGTGGTCCTCGCT
                                                   * *** ****     ***        *   ***

Arabidopsis thaliana              TTGGTCTCTGGTTTGATGGTTGUACGTGCTAAAAATCCGGTACATTCCGT
Brassica napus                    TTGGTCTCTGGTTTGATGGTTGUACGTGCTAAAAATCCGGTACATTCCGT
RRII105_nad6                      TTGGTCTCTGGTTTGATGGTTGCACGTGCTAAAAATCCGGTACATTCCGT
Beta macrocarpa                   TTGGTCTCTGGTTTGATGGTTGTACGTGCTAAAAATCCGGTACATTCCGT
Beta vulgaris maritima            TTGGTCTCTGGTTTGATGGTTGTACGTGCTAAAAATCCGGTACATTCCGT
Beta vulgaris vulgaris            TTGGTCTCTGGTTTGATGGTTGTACGTGCTAAAAATCCGGTACATTCCGT
Silene latifolia                  TTGGTCTCTGGTTTGATGGTTGTACGTGCTAAAAATCCGGTACATTCCGT
Oryza sativa Japonica             TTGGTCTCTGGTTTGATGGTTGTACGTGCTAAAAATCCGGTACATTCCGT
RRIC110_nad6                      TTGGTCTCTGGTTTGATGGTTGCACGTGCTAAAAATCCGGTACATTCCGT
Millettia pinnata                 TTGGTCTCTGGTTTGATGGTTGUACGTGCTAAAAATCCGGTACATTCCGT
BPM24_nad6                        TTGGTCTCTGGTTTGATGGTTGCACGTGCTAAAAATCCGGTACATTCCGT
Lotus japonicus                   TTGGTCTCTGGTTTGATGGTTGUACGTGCTAAAAATCCGGTACATTCCGT
PB235_nad6                        TTGGTCTCTGGTTTGATGGTTGCACGTGCTAAAAATCCGGTACATTCCGT
RRIT251_nad6                      TTGGTCTCTGGTTTGATGGTTGCACGTGCTAAAAATCCGGTACATTCCGT
RRIM600_nad6                      TTGGTCTCTGGTTTGATGGTTGCACGTGCTAAAAATCCGGTACATTCCGT
Cycas taitungensis                TTGGTCCCTGGTTTGATGGTTGTACGTGCTGAAAATCCAGTACATCCCGT
Zea mays mays                     TTGGTCTCTGGTTTGATGGTTGTACGTGCTAAAAATCCGGTACATTCCGT
Megaceros aenigmaticus            TTAGTCTCCGGTGTTACGGCGACACGTGCCAAAAATCCAGTCCATTCCGT
Phaeoceros laevis                 TTAGTCTCCGGTGTTACGGCGACACGTGCCAAAAATCTAGTCCATTTCGT
Pleurozia purpurea                TTAGTGTCAGGCGCTATGGTGATACGTGCCAAAAATCCAGTTCATTCTGT
                                  ** **  * **    * **    ******  ******  ** ***   **

Arabidopsis thaliana              TTTGTTTUUCATCCUAGTCTTTUGCGACACTTCAGGTTTACTTCTTTTGT
Brassica napus                    TTTGTTTUUCATCCUAGTCTTTUGCGACACTTCAGGTTTACTTCTTTTGT
RRII105_nad6                      TTTGTTTCCCATCCTAGTCTTTCGCGATACTTCAGGTTTACTTCTTTTGT
Beta macrocarpa                   TTTGTTTUUUATCUUAGTCTTTUGCAACACTTCAGGTTTACTTCTTTTGT
Beta vulgaris maritima            TTTGTTTUUUATCUUAGTCTTTUGCAACACTTCAGGTTTACTTCTTTTGT
Beta vulgaris vulgaris            TTTGTTTUUUATCUUAGTCTTTUGCAACACTTCAGGTTTACTTCTTTTGT
Silene latifolia                  TTTGTTTUUUATCUUAGTCTTTUGCAACACTTCAGGTTTACTTCTTTTGT
Oryza sativa Japonica             TTTGTTTUUCATCCTAATCTTTTGCGACACAGCTGGTTTACTTATTTTGT
RRIC110_nad6                      TTTGTTTCCCATCCTAGTCTTTCGCGATACTTCAGGTTTACTTCTTTTGT
Millettia pinnata                 TTTGTTTUUCATCCUAGTCTTTUGCGACACTTCAGGTTTACTTCTTTTGT
BPM24_nad6                        TTTGTTTCCCATCCTAGTCTTTCGCGATACTTCAGGTTTACTTCTTTTGT
Lotus japonicus                   TTTGTTTUUCATCCUAGTCTTTUGCGACACTTCAGGTTTACTTCTTTTGT
PB235_nad6                        TTTGTTTCCCATCCTAGTCTTTCGCGATACTTCAGGTTTACTTCTTTTGT
RRIT251_nad6                      TTTGTTTCCCATCCTAGTCTTTCGCGATACTTCAGGTTTACTTCTTTTGT
RRIM600_nad6                      TTTGTTTCCCATCCTAGTCTTTCGCGATACTTCAGGTTTACTTCTTTTGT
Cycas taitungensis                TTCGTTCCCCATCCCAGTGTTTCGCAACACTTCAGGTTTACTTATTTTGT
Zea mays mays                     TTTGTTTUUCATCCTAGTCTTTTGCGACACTTCTGGTTTACTTATTTTGT
Megaceros aenigmaticus            TTTATTTTCAATACCCGTCTTTCGCAACACTTCAGGTTTACTTGTTTTGC
Phaeoceros laevis                 TTTATTTTTAATACCCGTCTTTCGCAATACTTCAGGTTTACTTGTTTTGT
Pleurozia purpurea                TTTATTTTTAATCTCAGTTTTTCGCAATACTTCCGGTTTACTCGTTTTGT
                                  **  **    **     * *** ** * **  * ********  *****

Arabidopsis thaliana              TAGGTCTCGACTTCTTCGCTATGATCTTCCUAGTAGTTUATATAGGAGCT
Brassica napus                    TAGGTCTCGACTTCTTCGCTATGATCTTCCUAGTAGTTUATATAGGAGCT
RRII105_nad6                      TAGGTCTCGACTTCTUCGCTATGATCTTCCUAGTAGTTTATATAGGAGCT
Beta macrocarpa                   TAGGTCTCGATTTTTUCGCTATGATCTTCCUAGTAGTTTATATAGGAGCT
Beta vulgaris maritima            TAGGTCTCGATTTTTUCGCTATGATCTTCCUAGTAGTTTATATAGGAGCT
Beta vulgaris vulgaris            TAGGTCTCGATTTTTUCGCTATGATCTTCCUAGTAGTTTATATAGGAGCT
Silene latifolia                  TAGGTCTCGACTTTTUCGCTATGATCTTCTTAGTAGTTTATATAGGAGCT
Oryza sativa Japonica             TAGGTCTCGACTTCTUCGCTATGATCTUUCUAGTAATTUATATAGGAGCT
RRIC110_nad6                      TAGGTCTCGACTTCTUCGCTATGATCTTCCUAGTAGTTTATATAGGAGCT
Millettia pinnata                 TAGGTCTCGACTTCTUCGCTATGATCTTCCUAGTAGTTUATATAGGAGCT
BPM24_nad6                        TAGGTCTCGACTTCTUCGCTATGATCTTCCUAGTAGTTTATATAGGAGCT
Lotus japonicus                   TAGGTCTCGACTTCTUCGCTATGATCTTCCUAGTAGTTUATATAGGAGCT
```

```
PB235_nad6                        TAGGTCTCGACTTCTUCGCTATGATCTTCCUAGTAGTTTATATAGGAGCT
RRIT251_nad6                      TAGGTCTCGACTTCTUCGCTATGATCTTCCUAGTAGTTTATATAGGAGCT
RRIM600_nad6                      TAGGTCTCGACTTCTNCGCTATGATCTTCCNAGTAGTTTATATAGGAGCT
Cycas taitungensis                CAGGTCCTGACCTCTCTGCTATGATCTCCCCAGTAGTTCATATAGGAGCT
Zea mays mays                     TAGGTCTUGACTTCTUCGCTATGATCTTUCUAGTAGTTUATATAGGAGCT
Megaceros aenigmaticus            TAGGTCTCGACCCCTTCGCTATGATTTTTTTAGTGGTTTATGTAGGAGCT
Phaeoceros laevis                 TAGGTCTCGACTCCTTCGCTATGATTTTTTTAGTGGTTTATGCAGGAGCT
Pleurozia purpurea                TAGGTCCTGACTTCTTTGCTATGATTTTCCTAGTGGTTTATGTAGGAGCT
                                   *****  **    *  ******** *    ***  ** **  *******

Arabidopsis thaliana              ATAGCCGTTTUATTCCTTTTCGTTGTTATGATGTTCCATATTCAAATAGC
Brassica napus                    ATAGCCGTTTUATTCCTTTTCGTTGTTATGATGTTCCATATTCAAATAGC
RRII105_nad6                      ATAGCCGTTTTATTCCTATTCGTTGTTATGATGTTCCATATTCAAATAGC
Beta macrocarpa                   ATAGCCGTTTUATTCCTATTCGTTGTTATGATGTTCCATATTCAAATAGC
Beta vulgaris maritima            ATAGCCGTTTUATTCCTATTCGTTGTTATGATGTTCCATATTCAAATAGC
Beta vulgaris vulgaris            ATAGCCGTTTUATTCCTATTCGTTGTTATGATGTTCCATATTCAAATAGC
Silene latifolia                  ATAGCCGTTTUATTCCTATTCGTTGTTATGATGTTCCATATTCAAATAGC
Oryza sativa Japonica             ATTGCCGTTTUATTCCTATTCGTGGTTATGATGTTCAATATTCAAATAGC
RRIC110_nad6                      ATAGCCGTTTTATTCCTATTCGTTGTTATGATGTTCCATATTCAAATAGC
Millettia pinnata                 ATAGCCGTATUATTCCTATTCGTTGTTATGATGTTCCATATTCAAATAGC
BPM24_nad6                        ATAGCCGTTTTATTCCTATTCGTTGTTATGATGTTCCATATTCAAATAGC
Lotus japonicus                   ATAGCCGTTTUATTCCTATTCGTTGTTATGATGTTCCATATTCAAATAGC
PB235_nad6                        ATAGCCGTTTTATTCCTATTCGTTGTTATGATGTTCCATATTCAAATAGC
RRIT251_nad6                      ATAGCCGTTTTATTCCTATTCGTTGTTATGATGTTCCATATTCAAATAGC
RRIM600_nad6                      ATAGCCGTTTTATTCCTATTCGTTGTTATGATGTTCCATATTCAAATAGC
Cycas taitungensis                ATTGCCGTTCTATTCTCATCCGTGGTTATGATGTTAAATATTCAAATAGC
Zea mays mays                     ATTGCCGTTTUATTCCTATTCGTGGTTATGATGTTCAATATTCAAATAGC
Megaceros aenigmaticus            ATTGCTGTTTCACCTTCATCTGTAGCTACGACGTCGAATATTAAAATAGC
Phaeoceros laevis                 ATTGCTGCTTCATCTCCATCTGTAGCTATGACGTCAAATATTAAAATAGC
Pleurozia purpurea                ATTGCCGTTTCATTCTCGTTTGTTGTTATGATGTTACATACAAGGATAGA
                                  ** ** *    *      *  ** * ** ** **   ***     ****

Arabidopsis thaliana              GGAGATTCACGAAGAAGTATTGCGCTATTTACCAGTGAGTGGGATTATTG
Brassica napus                    GGAGATTCACGAAGAAGTATTGCGCTATTTACCAGTGAGTGGGATTATTG
RRII105_nad6                      GGAGATTCACGAAGAAGTCTTGCGCTATTTACCAGTGAGTGGTATTATTG
Beta macrocarpa                   GGAGATTCACGAAGAAGTATTGCGCTATTTACCAGTGAGTGGTATTATTG
Beta vulgaris maritima            GGAGATTCACGAAGAAGTATTGCGCTATTTACCAGTGAGTGGTATTATTG
Beta vulgaris vulgaris            GGAGATTCACGAAGAAGTATTGCGCTATTTACCAGTGAGTGGTATTATTG
Silene latifolia                  GGAGATTCACGAAGAAGTCTTGCGCTATTTACCAGTGAGTGGTATTATTG
Oryza sativa Japonica             GGAGATTCACGAAGAAGTATTGCGCTATTTACCAGTGAGTGGTATTATTG
RRIC110_nad6                      GGAGATTCACGAAGAAGTCTTGCGCTATTTACCAGTGAGTGGTATTATTG
Millettia pinnata                 GGAGATTCACGAAGAAGTATTGCGCTATTTACCAGTGAGTGGTATTATTG
BPM24_nad6                        GGAGATTCACGAAGAAGTCTTGCGCTATTTACCAGTGAGTGGTATTATTG
Lotus japonicus                   GGAGATTCACGAAGAAGTATTGCGCTATTTACCAGTCAGTGGTATTATTG
PB235_nad6                        GGAGATTCACGAAGAAGTCTTGCGCTATTTACCAGTGAGTGGTATTATTG
RRIT251_nad6                      GGAGATTCACGAAGAAGTCTTGCGCTATTTACCAGTGAGTGGTATTATTG
RRIM600_nad6                      GGAGATTCACGAAGAAGTCTTGCGCTATTTACCAGTGAGTGGTATTATTG
Cycas taitungensis                AGAGATTCACGAAAAAGTATCGCGCTATTCACCAGTGAGTGGTCTTATTG
Zea mays mays                     GGAGATTCACGAAGAAGTATTGCGCTATTTACCAGTGAGTGGTATTATTG
Megaceros aenigmaticus            AGAAATTCACGAGAATGTATTGCGCTATTTACCTGTAGGTGGTATTACTG
Phaeoceros laevis                 AGAAATTCACGAGAATGTATTGCGCTATTTACCTGTGGGTGGTATTATTG
Pleurozia purpurea                AGAAATTCACGAGAATGTGTTGCGCTATTTACCTGTAGGTGGTATTATTG
                                   ** ********  * ** * ******** *** **  ****  *** **

Arabidopsis thaliana              GACTGATCTTTTGGTGGGAAATGTTTTTCATTTTAGATAATGAAAGCATT
Brassica napus                    GACTGATCTTTTGGTGGGAAATGTTTTTCATTTTAGATAATGAAAGCATT
RRII105_nad6                      GACTGATCCTTTGGTGGGAAATGTTCTTCATTTTAGATAATGAAACCATT
Beta macrocarpa                   GACTGATCTTTTGGTGGGAAATGTTCTTCATTTTAGATAATGAAACCATT
Beta vulgaris maritima            GACTGATCTTTTGGTGGGAAATGTTCTTCATTTTAGATAATGAAACCATT
Beta vulgaris vulgaris            GACTGATCTTTTGGTGGGAAATGTTCTTCATTTTAGATAATGAAACCATT
Silene latifolia                  GACTGATCTTTTGGTGGGAAATGTTCTTCATTTTAGATAATGAAACCATT
Oryza sativa Japonica             GACTGATCTTTTGGTGGGAAATGTTCTTCATTTTAGATAATGAAACCATT
```

```
RRIC110_nad6                  GACTGATCCTTTGGTGGGAAATGTTCTTCATTTTAGATAATGAAACCATT
Millettia pinnata             GACTGATCUTTTGGTGGGAAATGTTCTTCATTTTAGATAATGAAACCATT
BPM24_nad6                    GACTGATCCTTTGGTGGGAAATGTTCTTCATTTTAGATAATGAAACCATT
Lotus japonicus               GACTGATCUTTTGGTGGGAAATGTTCTTCATTTTAGATAATGAAACCATT
PB235_nad6                    GACTGATCCTTTGGTGGGAAATGTTCTTCATTTTAGATAATGAAACCATT
RRIT251_nad6                  GACTGATCCTTTGGTGGGAAATGTTCTTCATTTTAGATAATGAAACCATT
RRIM600_nad6                  GACTGATCCTTTGGTGGGAAATGTTCTTCATTTTAGATAATGAAACCATT
Cycas taitungensis            GACCGATCCTTCGGTGGGAAATGTTCCTCATCCTAGATAATGATCACATT
Zea mays mays                 GACTGATCTTTTGGTGGGAAATGTTCTTCATTTTAGATAATGAAACCATT
Megaceros aenigmaticus        GACTTATTCTTTTGTTGGAAATATTTTTCATCGTAGATAATGATTACATT
Phaeoceros laevis             GACTTATTCTTTTGCTGGAAATCTTTTTTATTGTAGATAATGATTACATT
Pleurozia purpurea            GACTTATTTTTTTGTTGGAAATCTTTCTAATGGTAGATAATGATTACATC
                              ***  **  **  *  ****** **  * **  **********   ***

Arabidopsis thaliana          CCATTACTACCAACCCAAAGAAATACGACCTCTCTTAGATATACGGTTTA
Brassica napus                CCATTACTACCAACCCAAAGAAATACGACCTCTCTTAGATATACGGTTTA
RRII105_nad6                  CCATTACTACCAACCCAAAGAAATACGACCTCTCTGAGATATACAGTTTA
Beta macrocarpa               CCATTACTACCAACCCAAAGAAATACGACCTCTCTGAGATATACGGTTTA
Beta vulgaris maritima        CCATTACTACCAACCCAAAGAAATACGACCTCTCTGAGATATACGGTTTA
Beta vulgaris vulgaris        CCATTACTACCAACCCAAAGAAATACGACCTCTCTGAGATATACGGTTTA
Silene latifolia              CCATTACTACCAACCCAAAGAAATACGACCTCTCTGAGATATACGGTTTA
Oryza sativa Japonica         CCATTACTACCAACCCACAGAAATACGACCTCTCTGAGATATACGGTTTA
RRIC110_nad6                  CCATTACTACCAACCCAAAGAAATACGACCTCTCTGAGATATACAGTTTA
Millettia pinnata             CCATTACTACCAACCCAAAGAAATACGACCTCTCTGAGATATACGGTTTA
BPM24_nad6                    CCATTACTACCAACCCAAAGAAATACGACCTCTCTGAGATATACAGTTTA
Lotus japonicus               CCATTACTACCAACCCAAAGAAATACGACCTCTCTGAGATATACGGTTTA
PB235_nad6                    CCATTACTACCAACCCAAAGAAATACGACCTCTCTGAGATATACAGTTTA
RRIT251_nad6                  CCATTACTACCAACCCAAAGAAATACGACCTCTCTGAGATATACAGTTTA
RRIM600_nad6                  CCATTACTACCAACCCAAAGAAATACGACCTCTCTGAGATATACAGTTTA
Cycas taitungensis            CCATCATTACCGACCTACATAGGTACAACCTCTCTGAGATATACAGTTCA
Zea mays mays                 CCATTACTACCAACCCACAGAAATACGACCTCTCTGAAATATACGGTTTA
Megaceros aenigmaticus        CCAATATTACCAACGAAACTGAGTACAACCTATCTAACATATACAGTTTA
Phaeoceros laevis             CCAATATTACCAACGAAACTGAGTACAGCCTATCTAACATATACAGTTTA
Pleurozia purpurea            CCAATATTATCAACGAAATCGGGTGCAACCTATCTAACATATACAGTTTA
                              ***  * ** * **  *      * *  *** *** * ****** *** *

Arabidopsis thaliana          TGCCGGAAAGGTACGAAGTTGGACTAATTTGGAAACATTGGGCAATTTAC
Brassica napus                TGCCGGAAAGGTACGAAGTTGGACTAATTTGGAAACATTGGGAAATTTAC
RRII105_nad6                  TGCCGGAAAGGTACGAAGTTGGACTAATTTGGAAACATTGGGCAATTTAC
Beta macrocarpa               TGCCGGAAAGGTACGAAGTTGGACTAATTTGGAAACATTGGGCAATTTAC
Beta vulgaris maritima        TGCCGGAAAGGTACGAAGTTGGACTAATTTGGAAACATTGGGCAATTTAC
Beta vulgaris vulgaris        TGCCGGAAAGGTACGAAGTTGGACTAATTTGGAAACATTGGGCAATTTAC
Silene latifolia              TGCCGGAAAGGTACGAAGTTGGACTAATTTGGAAACATTGGGCAATTTAC
Oryza sativa Japonica         TGCCGGAAAGGTACGAAATTGGACTAATTTGGACACATTGGGCAATTTAC
RRIC110_nad6                  TGCCGGAAAGGTACGAAGTTGGACTAATTTGGAAACATTGGGCAATTTAC
Millettia pinnata             TGCCGGAAAGGTACGAAGTTGGACTAATTTGGAAACATTGGGAAATTTAC
BPM24_nad6                    TGCCGGAAAGGTACGAAGTTGGACTAATTTGGAAACATTGGGCAATTTAC
Lotus japonicus               TGCCGGAAAGGTACGAAGTTGGACTAATTTGGAAACATTGGGCAATTTAC
PB235_nad6                    TGCCGGAAAGGTACGAAGTTGGACTAATTTGGAAACATTGGGCAATTTAC
RRIT251_nad6                  TGCCGGAAAGGTACGAAGTTGGACTAATTTGGAAACATTGGGCAATTTAC
RRIM600_nad6                  TGCCGGAAAGGTACGAAGTTGGACTAATTTGGAAACATTGGGCAATTTAC
Cycas taitungensis            TGCTGGAGAGATACAAAGTTGGACTAATTCGGAAACATTGGGCAATTTAC
Zea mays mays                 TGCCGGAAAGGTACGAAGTTGGACTAATTTGGAAACATTGGGCAATTTAC
Megaceros aenigmaticus        TGCTGGAAAGATACAAAGTTGGACTAATTTGGAAACGTTAGGCAATTTAC
Phaeoceros laevis             TGCTGGAAAGATACAAAGTTGGACTAATTTGGAAACCTTAGGCAATTTAC
Pleurozia purpurea            TGCTGAAAAGATACATAGTTGGACTAATTCGGAGACATTAGGCAATTTAC
                              *** * * ** ***  * *********** *** ** ** ** *******

Arabidopsis thaliana          TTTATACCTACTATTUTGTCTGGTTTTTGGTTCCTAGTCTTATTTTATTA
Brassica napus                TTTATACCTACTATTUTGTCTGGTTTTTGGTTUCTAGTCTTATTTTATTA
RRII105_nad6                  TTTATACCTACTATTTCGTCTGGTTTTTGGTTUCTAGTCTTATTTTATTA
Beta macrocarpa               TTTATACTTACTATTTTGTCTGGTTTTTGGTTTCTAGTCTTATTTTATTA
```

```
Beta vulgaris maritima        TTTATACTTACTATTTTGTCTGGTTTTTGGTTTCTAGTCTTATTTTATTA
Beta vulgaris vulgaris        TTTATACTTACTATTTTGTCTGGTTTTTGGTTTCTAGTCTTATTTTATTA
Silene latifolia              TTTATACTTACTATTTTGTCTGGTTTTTGGTTTCTAGTCTTATTTTATTA
Oryza sativa Japonica         TTTATACCTACTATTUCGTCTGGTTTTTGGTTTCTACTCTGATUTUATTA
RRIC110_nad6                  TTTATACCTACTATTTCGTCTGGTTTTTGGTTUCTAGTCTTATTTTATTA
Millettia pinnata             TTTATACCTACTATTTCGTCTGGTTTTTGGTTUCTAGTCTAATTTTATTA
BPM24_nad6                    TTTATACCTACTATTTCGTCTGGTTTTTGGTTUCTAGTCTTATTTTATTA
Lotus japonicus               TTTATACCTACTATTTCGTCTGGTTTTTGGTTUCTAGTCTAATTTTATTA
PB235_nad6                    TTTATACCTACTATTTCGTCTGGTTTTTGGTTUCTAGTCTTATTTTATTA
RRIT251_nad6                  TTTATACCTACTATTTCGTCTGGTTTTTGGTTUCTAGTCTTATTTTATTA
RRIM600_nad6                  TTTATACCTACTATTTCGTCTGGTTTTTGGTTNCTAGTCTTATTTTATTA
Cycas taitungensis            TTCATACCTACTATTCCGTTCGGTTCCTGGTTTCTAGTCCTGTTCCATTA
Zea mays mays                 TTTATACCTACTATTUCGTCTGGTTTTTGGTTTCTAGTCTGATTTTATTA
Megaceros aenigmaticus        TTTATACCACCTATTTTACTTCGTTCTTGTTTTCTAGTTTCATTTCATTG
Phaeoceros laevis             TTTATACCACCTATTTTACTTTGTTCTTGTTTTCTAGTTTTATTTTATTG
Pleurozia purpurea            TTTATACAACCTATTTTTTCTTGTTTCTGGTTTCTAGTCTAATTTCATTA
                              ** ****   *****       ***  ** ** *** *    *   ***

Arabidopsis thaliana          GTAGCCATGATTGGGGCTATAGTACTGACTATGCATAGGACTACTAAGGT
Brassica napus                GTAGCCATGATTGGGGCTATAGTACTGACTATGCATAGGACTACTAAGGT
RRII105_nad6                  GTAGCCATGATTGGGGCTATAGTACTGACTATGCATAGGACTACTAAGGT
Beta macrocarpa               GTAGCCATGATTGGGGCTATAGTACTGACTATGCATAGGACTACTAAGGT
Beta vulgaris maritima        GTAGCCATGATTGGGGCTATAGTACTGACTATGCATAGGACTACTAAGGT
Beta vulgaris vulgaris        GTAGCCATGATTGGGGCTATAGTACTGACTATGCATAGGACTACTAAGGT
Silene latifolia              GTAGCCATGATTGGGGCTATAGTACTGACTATGCATAGGACTACTAAGGT
Oryza sativa Japonica         GTAGCTATGATTGGAGCTATAGTACTGACTATGCATAGGACTACAAAGGT
RRIC110_nad6                  GTAGCCATGATTGGGGCTATAGTACTGACTATGCATAGGACTACTAAGGT
Millettia pinnata             GTTGCCATGATTGGGGCTATAGTACTTACTATGCATAGGACTACTCAGGT
BPM24_nad6                    GTAGCCATGATTGGGGCTATAGTACTGACTATGCATAGGACTACTAAGGT
Lotus japonicus               GTTGCCATGATTGGGGCTATAGTACTGACTATGCATAGGACTACTCAGGT
PB235_nad6                    GTAGCCATGATTGGGGCTATAGTACTGACTATGCATAGGACTACTAAGGT
RRIT251_nad6                  GTAGCCATGATTGGGGCTATAGTACTGACTATGCATAGGACTACTAAGGT
RRIM600_nad6                  GTAGCCATGATTGGGGCTATAGTACTGACTATGCATAGGACTACTAAGGT
Cycas taitungensis            GTAGCCATGATTGGGGCTATAGTACTGACTATGCATAGGACTACTAAGGT
Zea mays mays                 GTAGCTATGATTGGGGCTATAGTACTTACTATGCATAGGACTACAAAGGT
Megaceros aenigmaticus        GTAGCCATGATTGGGGCTACAGTACTTACTATGCATAGAACTACTCGGGT
Phaeoceros laevis             GTAGCCATGATTGGGGCTACAGTACTTACTATGCATAAAACTACTCGGGT
Pleurozia purpurea            GTAGCACTTATTGGGGCAATAGTACTTACAATGCATAAAACGACTAGGGT
                              ** **  * ***** ** * ****** ** *******  ** **   ***

Arabidopsis thaliana          GAAAAGACAGGATGTATTCCGACGAAATGCTATTGATTTTAGGAGGACTA
Brassica napus                GAAAAGACAGGATGTATTCCGACGAAATGCTATTGATTTTAGGAGGACTA
RRII105_nad6                  GAAAAGACAGGATATATTTCGACGAAATGCTATTGATTTTAGGAGGACTA
Beta macrocarpa               GAAAAGACAGGATGTATTCCGACGAAATGCTATTGATTUTAGAAGGACTA
Beta vulgaris maritima        GAAAAGACAGGATGTATTCCGACGAAATGCTATTGATTUTAGAAGGACTA
Beta vulgaris vulgaris        GAAAAGACAGGATGTATTCCGACGAAATGCTATTGATTUTAGAAGGACTA
Silene latifolia              GAAAAGACAGGATGTATTCCGACGAAATGCTATTGATTUTAGAAGGACTA
Oryza sativa Japonica         TCAAAGACAGGATGTATTCCGACGAAATGCCUTGGAUTUTAGGAGGACTA
RRIC110_nad6                  GAAAAGACAGGATATATTTCGACGAAATGCTATTGATTTTAGGAGGACTA
Millettia pinnata             AAAAAGACAGGATGTTTTCCGACGAAATGCTATTGATTTTACGAGGACTA
BPM24_nad6                    GAAAAGACAGGATATATTTCGACGAAATGCTATTGATTTTAGGAGGACTA
Lotus japonicus               AAAAAGACAGGATGTTTTCCGACGAAATGCTATTGAATTTAGGAGGACTA
PB235_nad6                    GAAAAGACAGGATATATTTCGACGAAATGCTATTGATTTTAGGAGGACTA
RRIT251_nad6                  GAAAAGACAGGATATATTTCGACGAAATGCTATTGATTTTAGGAGGACTA
RRIM600_nad6                  GAAAAGACAGGATATATTTCGACGAAATGCTATTGATTTTAGGAGGACTA
Cycas taitungensis            GAAGAGACAGGATGTATTCCGACGAAATGCTATTGATTCTAGGAGGACCA
Zea mays mays                 GAAAAGACAGGATGTATTCCGACGAAATGCCTTGGATTUTAGGAGGACTA
Megaceros aenigmaticus        CAAAAGACAAGATGTGTTCCGACAAAATGCTAGAGATTTTCGGAATACTA
Phaeoceros laevis             CAAAAGACAAGATGTGTTCCGACAAAATGCTAGAGATTTTCAGAATACTA
Pleurozia purpurea            CAAACGTCAGGATGTTTTCATACAAAATGCTATAGATTTTCGGAATACTA
                                *  * ** *** * **   ** ******    ** * *   *  ** *
```

```
Arabidopsis thaliana          ----TAATGAGGAGGACGACTGACCCACTCACGATCTACTAAAGGGCAAG
Brassica napus                ----TAATGAGGAGGACGACTGACCCACTCACGATCTACTAAAGGGCAAG
RRII105_nad6                  ----TAATGAGGAGGACGACAGACCCACTCACGATCTACTAA--------
Beta macrocarpa               ----TAATGAAGAGGACGACAGACCCACTCACGATCTACTAAAGGTATCG
Beta vulgaris maritima        ----TAATGAAGAGGACGACAGACCCACTCACGATCTACTAAAGGTATCG
Beta vulgaris vulgaris        ----TAATGAAGAGGACGACAGACCCACTCACGATCTACTAAAGGTATCG
Silene latifolia              ----TAATGAGGAGGACGACAGACCCACTCACGATCTACTAAAGGTATCG
Oryza sativa Japonica         ----TAATGAGGAGGACGACAGACCAAATUTCGATUTACTAAAAG----G
RRIC110_nad6                  ----TAATGAGGAGGACGACAGACCCACTCACGATCTACTAA--------
Millettia pinnata             ----TAATGAGGCGGACGACAGACCCACTCACGATCGACTAAAAGGAAAG
BPM24_nad6                    ----TAATGAGGAGGACGACAGACCCACTCACGATCTACTAA--------
Lotus japonicus               ----TAATGAGGCGGACGACAGACCCACTCACGATCGACTAAAAGGAAAG
PB235_nad6                    ----TAATGAGGAGGACGACAGACCCACTCACGATCTACTAA--------
RRIT251_nad6                  ----TAATGAGGAGGACGACAGACCCACTCACGATCTACTAA--------
RRIM600_nad6                  ----TAATGAGGAGGACGACAGACCCACTCACGATCTACTAA--------
Cycas taitungensis            ----TAATGAGGAGGACGACGGACCAAU---AAGTTGATCGCGGGCGGAG
Zea mays mays                 CTATTCATATTCAGAACAGAAGGTTCTTCTCCCACAAAGGGGATGACGCA
Megaceros aenigmaticus        ----TAA-AAAAAATCAGAAAGATCTGA----------------------
Phaeoceros laevis             ----TAA-AAAAAATCAGAAAGATCTGA----------------------
Pleurozia purpurea            ----TCA-GAAAAGTTCGTTGAAAATGCTGTCAATTCGTTTTTTGGTAAA
                                  * *

Arabidopsis thaliana          TAGCTTG-----ATTGGTTCG----------AATCCAATTCGTGAGCAGA
Brassica napus                TAGCTTG-----ATTGGTTCG----------AATCCAATTCGCGAGCAGA
RRII105_nad6                  --------------------------------------------------
Beta macrocarpa               GAGATTG-----ATTGGTTCG----------AATCCAATTCGTGAGATGG
Beta vulgaris maritima        GAGATTG-----ATTGGTTCG----------AATCCAATTCGTGAGATGG
Beta vulgaris vulgaris        GAGATTG-----ATTGGTTCG----------AATCCAATTCGTGAGATGG
Silene latifolia              GAGATTG-----ATTGGTTCA----------AATCCAATTCGTGAGATGG
Oryza sativa Japonica         AGAATTG-------TGGTTCG----------AGTCCACGTCGTGAGATGG
RRIC110_nad6                  --------------------------------------------------
Millettia pinnata             TCGCCCGGAGATATTGGTTCA----------AATCCAATTCGTGAGAAGA
BPM24_nad6                    --------------------------------------------------
Lotus japonicus               TCGCCCGGAGATATTGGTTCA----------AATCCAATTCGTGAGAAGA
PB235_nad6                    --------------------------------------------------
RRIT251_nad6                  --------------------------------------------------
RRIM600_nad6                  --------------------------------------------------
Cycas taitungensis            ACGGGGGA----ACCA--CCG----------GGCCCAG--CAACGGCTGC
Zea mays mays                 CCTGTCCCCCCCGCTGATCCGGAATCCATTTAATCTGATTTGGCAGAGAG
Megaceros aenigmaticus        --------------------------------------------------
Phaeoceros laevis             --------------------------------------------------
Pleurozia purpurea            ACATAATGG--TATCGATTAGAATTTCGAATGAGGTTGTCTGGAACTCGG

Arabidopsis thaliana          TGAGAAATTGTTGCAC----CGTGGACTCTGATTCAGTAGCGGCAGCAAA
Brassica napus                TGAGAAATTGTTGCAC----CGTGGACTCTGATTCAGTAGCGGCAGCAAA
RRII105_nad6                  --------------------------------------------------
Beta macrocarpa               C-AGTGATCTTTAGCT-GGTCATGGCCATTAAAC----CTTTTTAAGGGC
Beta vulgaris maritima        C-AGTGATCTTTAGCT-GGTCATGGCCATTAAAC----CTTTTTAAGGGC
Beta vulgaris vulgaris        C-AGTGATCTTTAGCT-GGTCATGGCCATTAAAC----CTTTTTAAGGGC
Silene latifolia              C-AGTGATCTTTAGCT-GGTCATGGCCATTAAAC----CTTTTTAAGGGC
Oryza sativa Japonica         CCAGTGATCTTTAGCTTGGTCATGGCCATAATGTGCTTTTTTTCCTCGGA
RRIC110_nad6                  --------------------------------------------------
Millettia pinnata             A-TCCAAGTGAGATACTAATAAAGTAAAGTATAAAGGGCTTTTGC-----
BPM24_nad6                    --------------------------------------------------
Lotus japonicus               A-TCCAAGTGAGATACTAATAAAGTAAAGTATAAAGGGCTTTTGCAA---
PB235_nad6                    --------------------------------------------------
RRIT251_nad6                  --------------------------------------------------
RRIM600_nad6                  --------------------------------------------------
Cycas taitungensis            GCTACCACTACTATATAAAGAGAGGGGGCCACTACTGCTGCGC-------
Zea mays mays                 AATAGAAAGGATTTAT-AGGCGGGCCGGGGAGGAGCAATCTTTAGTAGAA
Megaceros aenigmaticus        --------------------------------------------------
```

```
Phaeoceros laevis                  --------------------------------------------------
Pleurozia purpurea                 GTCTATCTTTCTC-------------------------------------


Arabidopsis thaliana               TCCTT---------------------------
Brassica napus                     TCC-----------------------------
RRII105_nad6                       --------------------------------
Beta macrocarpa                    TTTAA---------------------------
Beta vulgaris maritima             TTTAA---------------------------
Beta vulgaris vulgaris             TTTAA---------------------------
Silene latifolia                   GCTAG---------------------------
Oryza sativa Japonica              TTCATTG-------------------------
RRIC110_nad6                       --------------------------------
Millettia pinnata                  --------------------------------
BPM24_nad6                         --------------------------------
Lotus japonicus                    --------------------------------
PB235_nad6                         --------------------------------
RRIT251_nad6                       --------------------------------
RRIM600_nad6                       --------------------------------
Cycas taitungensis                 --------------------------------
Zea mays mays                      GCCGGGACTACGCCAAGGGAACTTGTAGATAA
Megaceros aenigmaticus             --------------------------------
Phaeoceros laevis                  --------------------------------
Pleurozia purpurea                 --------------------------------
```

###################

nad7 multiple sequence alignment

```
Citrullus lanatus                  ATGACGACTAGGAACAGGCAAATCAAAAATTTCACTTUGAATTUUGGACC
Cucurbita pepo                     ATGACGACTAGTAAGAGGCAAATAACAAATTTCACTTUGAATTUUGGACC
Arabidopsis thaliana               ATGACGACTAGGAAAAGGCAAATUAAAAATTTTACTTUGAATTTTGGACC
Brassica napus                     ATGACGACTAGGAAAAGGCAAATUAAAAATTTTACTTUGAATTTTGGACC
RRII105_nad7                       ATGACGACTAGGAACGGGCAAATCAAAAATTTCACTTUGAATTTCGGACC
PB235_nad7                         ATGACGACTAGGAACGGGCAAATCAAAAATTTCACTTUGAATTTCGGACC
BPM24_nad7                         ATGACGACTAGGAACGGGCAAATCAAAAATTTCACTTUGAATTTCGGACC
RRIC110_nad7                       ATGACGACTAGGAACGGGCAAATCAAAAATTTCACTTUGAATTTCGGACC
RRIM600_nad7                       ATGACGACTAGGAACGGGCAAATCAAAAATTTCACTTUGAATTTCGGACC
RRIT251_nad7                       ATGACGACTAGGAACGGGCAAATCAAAAATTTCACTTNGAATTTCGGACC
Beta macrocarpa                    ---------ATGAACAGACAAATCAAAAATTTCACTTTGAATTTCGGACC
Beta vulgaris vulgaris             ---------ATGAACAGACAAATCAAAAATTTCACTTTGAATTTCGGACC
Beta vulgaris maritima             ---------ATGAACAGACAAATCAAAAATTTCACTTTGAATTTCGGACC
Silene latifolia                   ATGACTACCAGAAAGAAACAAATAAAAAATTTCACTTUGAATTTCGGACC
Lotus japonicus                    ATGACGACTAGGAACGGGCAAATCCAAAATTTCACTTUGAATTTUGGACC
Millettia pinnata                  ATGACGACTAGGAACGGGCAAATCAAAAATTTCACTTUGAATTTUGGACC
Oryza sativa Japonica              ATGACGACTAGGAACGGGCAAATCAAGAATTTCACTTUGAATTUUGGACC
                                            *  **    *****    ***** **** *****  *****

Citrullus lanatus                  TCAACATCCTGCTGCTCATGGTGTTTUACGATUAGTATTGGAAATGAACG
Cucurbita pepo                     TCAACATCCTGCTGCTCATGGTGTTTUACGATUAGTATTGGAAATGAACG
Arabidopsis thaliana               TCAACATCCTGCTGCTCATGGTGTTTUACGATTAGTATTGGAAATGAACG
Brassica napus                     TCAACATCCTGCTGCTCATGGTGTTTUACGATTAGTATTGGAAATGAACG
RRII105_nad7                       TCAACATCCTGCTGCTCATGGTGTTTCACGATCAGTATTGGAAATGAACG
PB235_nad7                         TCAACATCCTGCTGCTCATGGTGTTTCACGATCAGTATTGGAAATGAACG
BPM24_nad7                         TCAACATCCTGCTGCTCATGGTGTTTCACGATCAGTATTGGAAATGAACG
RRIC110_nad7                       TCAACATCCTGCTGCTCATGGTGTTTCACGATCAGTATTGGAAATGAACG
RRIM600_nad7                       TCAACATCCTGCTGCTCATGGTGTTTCACGATCAGTATTGGAAATGAACG
RRIT251_nad7                       TCAACATCCTGCTGCTCATGGTGTTTCACGATCAGTATTGGAAATGAACG
Beta macrocarpa                    TCAACATCCTGCTGCTCATGGTGTTTUACGATTAGTATTGGAAATGAACG
Beta vulgaris vulgaris             TCAACATCCTGCTGCTCATGGTGTTTUACGATTAGTATTGGAAATGAACG
Beta vulgaris maritima             TCAACATCCTGCTGCTCATGGTGTTTUACGATTAGTATTGGAAATGAACG
Silene latifolia                   TCAACATCCTGCTGCTCATGGTGTTTUACGATTAGTATTGGAAATGAACG
Lotus japonicus                    TCAACATCCTGCTGCTCATGGTGTTTUACGATUAGTATTGGAAATGAACG
```

```
Millettia pinnata                  TCAACATCCTGCTGCTCATGGTGTTTUACGATUAGTATTGGAAATGAACG
Oryza sativa Japonica              TCAACATCCTGCTGCTCATGGTGTTTUACGATUAGTATTGGAAATGAAUG
                                   ************************** ***** *************** *

Citrullus lanatus                  GAGAAGTGGTGGAACGTGCGGAACCACATATTGGATTACTCCA--TAGAG
Cucurbita pepo                     GAGAAGTGGTGGAACGTGCGGAACCACATATTGGATTACTCCA--TAGAG
Arabidopsis thaliana               GAGAAGTGGTGGAACGTGCGGAACCACATATTGGATUACTCCA--TAGAG
Brassica napus                     GAGAAGTGGTGGAACGTGCGGAACCACATATTGGATUACTCCA--TAGAG
RRII105_nad7                       GAGAAGTGGTGGAACGTGCGGAACCACATATTGGATTACTCCA--TAGAG
PB235_nad7                         GAGAAGTGGTGGAACGTGCGGAACCACATATTGGATTACTCCA--TAGAG
BPM24_nad7                         GAGAAGTGGTGGAACGTGCGGAACCACATATTGGATTACTCCA--TAGAG
RRIC110_nad7                       GAGAAGTGGTGGAACGTGCGGAACCACATATTGGATTACTCCA--TAGAG
RRIM600_nad7                       GAGAAGTGGTGGAACGTGCGGAACCACATATTGGATTACTCCA--TAGAG
RRIT251_nad7                       GAGAAGTGGTGGAACGTGCGGAACCACATATTGGATTACTCCA--TAGAG
Beta macrocarpa                    GAGAAGTGGTGGAACGTGCAGAACCACATATTGGATTACTCCA--TAGAG
Beta vulgaris vulgaris             GAGAAGTGGTGGAACGTGCAGAACCACATATTGGATTACTCCA--TAGAG
Beta vulgaris maritima             GAGAAGTGGTGGAACGTGCAGAACCACATATTGGATTACTCCA--TAGAG
Silene latifolia                   GAGAAGTGGTGGAACGTGCGGAACCACATATTGGATTACTCCA--TAGAG
Lotus japonicus                    GAGAAGTGGTGGAACGTGCGGAACCACATATTGGATUACTCCA--TAGAG
Millettia pinnata                  GAGAAGTGGTGGAACGTGCGGAACCACATATTGGATUACTCCA--TAGAG
Oryza sativa Japonica              GAGAAGTGGTGGAACGTGCGGAACCACATATTGGATUACTCCAACTAGAG
                                   ******************* **************** ******  *****

Citrullus lanatus                  GGACTGAGAAATTAATAGAGTACAAAACTTATCTTCAAGCTTTACCTTAT
Cucurbita pepo                     GGACTGAGAAATTAATAGAGTACAAAACTTATCTTCAAGCTTTACCTTAT
Arabidopsis thaliana               GGACTGAGAAATTAATAGAGTACAAAACTTATCTTCAAGCTTTACCTTAT
Brassica napus                     GGACTGAGAAATTAATAGAGTACAAAACTTATCTTCAAGCTTTACCTTAT
RRII105_nad7                       GGACTGAGAAATTAATAGAGTACAAAACTTATCTTCAAGCTTTACCTTAT
PB235_nad7                         GGACTGAGAAATTAATAGAGTACAAAACTTATCTTCAAGCTTTACCTTAT
BPM24_nad7                         GGACTGAGAAATTAATAGAGTACAAAACTTATCTTCAAGCTTTACCTTAT
RRIC110_nad7                       GGACTGAGAAATTAATAGAGTACAAAACTTATCTTCAAGCTTTACCTTAT
RRIM600_nad7                       GGACTGAGAAATTAATAGAGTACAAAACTTATCTTCAAGCTTTACCTTAT
RRIT251_nad7                       GGACTGAGAAATTAATAGAGTACAAAACTTATCTTCAAGCTTTACCTTAT
Beta macrocarpa                    GGACTGAGAAATTAATAGAGTACAAAACTTATCTTCAAGCTTTACCTTAT
Beta vulgaris vulgaris             GGACTGAGAAATTAATAGAGTACAAAACTTATCTTCAAGCTTTACCTTAT
Beta vulgaris maritima             GGACTGAGAAATTAATAGAGTACAAAACTTATCTTCAAGCTTTACCTTAT
Silene latifolia                   GGACTGAGAAATTAATAGAGTACAAAACTTATCTTCAAGCTTTACCTTAT
Lotus japonicus                    GGACTGAGAAATTAATAGAGTACAAAACTTATCTTCAAGCTTTACCTTAT
Millettia pinnata                  GGACTGAGAAATTAATAGAGTACAAAACTTATCTTCAAGCTTTACCTTAT
Oryza sativa Japonica              GGACTGAGAAATTAATCGAGTACAAAACTTATCTTCAAGCTTTACCTTAT
                                   **************** *********************************

Citrullus lanatus                  TUTGATCGTTUAGACTATGTTTCTAUGATGGCCCAAGAACACGCTUATTC
Cucurbita pepo                     TUTGATCGTTUAGACTATGTTTCTAUGATGGCCCAAGAACACGCTUATTC
Arabidopsis thaliana               TUTGATCGTTCAGAUTATGTTTCTATGATGGCCCAAGAACACGCTUATTC
Brassica napus                     TUTGATCGTTUAGAUTATGTTTCTATGATGGCCCAAGAACACGCTUATTC
RRII105_nad7                       TCTGATCGTTCAGACTATGTTTCTAUGATGGCCCAAGAACACGCTTATTC
PB235_nad7                         TCTGATCGTTCAGACTATGTTTCTAUGATGGCCCAAGAACACGCTTATTC
BPM24_nad7                         TCTGATCGTTCAGACTATGTTTCTANGATGGCCCAAGAACACGCTTATTC
RRIC110_nad7                       TCTGATCGTTCAGACTATGTTTCTAUGATGGCCCAAGAACACGCTTATTC
RRIM600_nad7                       TCTGATCGTTCAGACTATGTTTCTAUGATGGCCCAAGAACACGCTTATTC
RRIT251_nad7                       TCTGATCGTTCAGACTATGTTTCTACGATGGCCCAAGAACACGCTTATTC
Beta macrocarpa                    TTTGATCGTTTAGACTATGTTTCTATGATGGCCCAAGAACACGCTUATTC
Beta vulgaris vulgaris             TTTGATCGTTTAGACTATGTTTCTATGATGGCCCAAGAACACGCTUATTC
Beta vulgaris maritima             TTTGATCGTTTAGACTATGTTTCTATGATGGCCCAAGAACACGCTUATTC
Silene latifolia                   TTTGATCGTTTAGACTATGTTTCTATGATGGCCCAAGAACACGCTUATTC
Lotus japonicus                    TUTGATCGTTTAGACTATGTTTCTAUGATGGCCCAAGAACACGCTUATTC
Millettia pinnata                  TUTGATCGTTTAGACTATGTTTCTAUGATGGCCCAAGAACACGCTUATTC
Oryza sativa Japonica              TTTGATCGTTUAGACTATGTTTCTAUGATGGCCCAAGAACACGCTUATTC
                                   * ******** *** ********** ******************* ****

Citrullus lanatus                  TTUAGCTGTAGAGAGACTTTTGAATTGCGAGGTACCATTACGAGCTCAAT
```

```
Cucurbita pepo                TTUAGCTGTAGAGAGACTTTTGAATTGCGAGGTACCATTACGAGCTCAAT
Arabidopsis thaliana          TTUAGCTGTAGAGAAACTTTTGAATTGCGAGGTACCATTACGAGCTCAAT
Brassica napus                TTUAGCTGTAGAGAAACTTTTGAATTGCGAGGTACCATTACGAGCTCAAT
RRII105_nad7                  TTCAGCCGTAGAGAGACTTTTGAATTGCGAGGTACCATTACGAGCTCAAT
PB235_nad7                    TTCAGCCGTAGAGAGACTTTTGAATTGCGAGGTACCATTACGAGCTCAAT
BPM24_nad7                    TTCAGCCGTAGAGAGACTTTTGAATTGCGAGGTACCATTACGAGCTCAAT
RRIC110_nad7                  TTCAGCCGTAGAGAGACTTTTGAATTGCGAGGTACCATTACGAGCTCAAT
RRIM600_nad7                  TTCAGCCGTAGAGAGACTTTTGAATTGCGAGGTACCATTACGAGCTCAAT
RRIT251_nad7                  TTCAGCCGTAGAGAGACTTTTGAATTGCGAGGTACCATTACGAGCTCAAT
Beta macrocarpa               TTUAGCCGTAGAGAGACTTTTGAATTGCGAGGTACCATTACGAGCTCAAT
Beta vulgaris vulgaris        TTUAGCCGTAGAGAGACTTTTGAATTGCGAGGTACCATTACGAGCTCAAT
Beta vulgaris maritima        TTUAGCCGTAGAGAGACTTTTGAATTGCGAGGTACCATTACGAGCTCAAT
Silene latifolia              TTUAGCCGTAGAGAGACTTTTGAATTGCGAGGTACCATTACGAGCTCAAT
Lotus japonicus               TTUAGCCGTAGAGAGACTTTTGAATTGCGAGGTACCATTACGAGCTCAAT
Millettia pinnata             TTUAGCCGTAGAGAGACTTTTGAATTGCGAGGTACCATTACGAGCTCAAT
Oryza sativa Japonica         TTUAGCCGTAGAGAGACTTTTGAATTGTGAGGTACCATTACGAGCTCAAT
                              ** *** ******* ************ **********************

Citrullus lanatus             ATATACGAGTGTTATTCUGTGAAATAACTCGAATTTUAAATCATTUACTT
Cucurbita pepo                ATATACGAGTGTTATTCUGTGAAATCACTCGAATTTUAAATCATTUACTT
Arabidopsis thaliana          ATATACGAGTGTTATTCUGTGAAATAACTCGAATTTUAAATCATTUACTT
Brassica napus                ATATACGAGTGTTATTCUGTGAAATAACTCGAATTTUAAATCATTUACTT
RRII105_nad7                  ATATACGAGTGTTATTCCGTGAAATAACTCGAATTTCAAATCATTCACTT
PB235_nad7                    ATATACGAGTGTTATTCCGTGAAATAACTCGAATTTCAAATCATTCACTT
BPM24_nad7                    ATATACGAGTGTTATTCCGTGAAATAACTCGAATTTCAAATCATTCACTT
RRIC110_nad7                  ATATACGAGTGTTATTCCGTGAAATAACTCGAATTTCAAATCATTCACTT
RRIM600_nad7                  ATATACGAGTGTTATTCCGTGAAATAACTCGAATTTCAAATCATTCACTT
RRIT251_nad7                  ATATACGAGTGTTATTCCGTGAAATAACTCGAATTTCAAATCATTCACTT
Beta macrocarpa               ATATACGAGTGTTATTCUGTGAAATAACTCGAATTTUAAATCATTUACTT
Beta vulgaris vulgaris        ATATACGAGTGTTATTCUGTGAAATAACTCGAATTTUAAATCATTUACTT
Beta vulgaris maritima        ATATACGAGTGTTATTCUGTGAAATAACTCGAATTTUAAATCATTUACTT
Silene latifolia              ATATACGAGTGCTATTCUGTGAAATAACTCGAATTTTAAATCATTUACTT
Lotus japonicus               ATATACGAGTGTTATTCUGTGAAATAACTCGAATTTUAAATCATTUACTT
Millettia pinnata             ATATACGAGTGTTATTCUGTGAAATAACTCGAATTTUAAATCATTTACTT
Oryza sativa Japonica         ATATACGAGTGTTATTUTGTGAAATAACTCGAATTTUAAATCATTUACTT
                              *********** ****  ******* ********** ******** ****

Citrullus lanatus             GCTTTAACTACTCATGCTATGGATGTGGGAGCATUAACTCCGTTCCTGTG
Cucurbita pepo                GCTTTAACTACTCATGCTATGGATGTGGGAGCATUAACTCCGTTCCTGTG
Arabidopsis thaliana          GCTTTAACTACTCATGCTATGGATGTGGGAGCATTAACTCCGTTCCTGTG
Brassica napus                GCTTTAACTACTCATGCTATGGATGTGGGAGCATTAACTCCGTTCCTGTG
RRII105_nad7                  GCTTTAACTACTCATGCTATGGATGTGGGAGCATUAACTCCGTTCCTGTG
PB235_nad7                    GCTTTAACTACTCATGCTATGGATGTGGGAGCATUAACTCCGTTCCTGTG
BPM24_nad7                    GCTTTAACTACTCATGCTATGGATGTGGGAGCATUAACTCCGTTCCTGTG
RRIC110_nad7                  GCTTTAACTACTCATGCTATGGATGTGGGAGCATUAACTCCGTTCCTGTG
RRIM600_nad7                  GCTTTAACTACTCATGCTATGGATGTGGGAGCATNAACTCCGTTCCTGTG
RRIT251_nad7                  GCTTTAACTACTCATGCTATGGATGTGGGAGCATUAACTCCGTTCCTGTG
Beta macrocarpa               GCTTTAACTACTCATGCTATGGATGTGGGAGCATTAACTCCGTTCCTGTG
Beta vulgaris vulgaris        GCTTTAACTACTCATGCTATGGATGTGGGAGCATTAACTCCGTTCCTGTG
Beta vulgaris maritima        GCTTTAACTACTCATGCTATGGATGTGGGAGCATTAACTCCGTTCCTGTG
Silene latifolia              GCTTTAACTACTCATGCTATGGATGTGGGAGCATTAACTCCGTTCCTGTG
Lotus japonicus               GCTTTAACTACTCATGCTATGGATGTGGGAGCATUAACTCCGTCCCTGTG
Millettia pinnata             GCTTTAACTAGTCATGCTATGGATGTGGGAGCATUAACTCCGTCCCTGTG
Oryza sativa Japonica         GCTTUAACTACTCATGCTATGGATGTGGGAGCATUAACTCCGTTCCTTTG
                              **** ***** *********************** ******** *** **

Citrullus lanatus             GGCTTTTGAGGAGCGGGAGAAATTATTGGAATTCTATGAAAGAGTCTCGG
Cucurbita pepo                GGCTTTTGAGGAGCGGGAGAAATTATTGGAATTCTATGAAAGAGTCTCGG
Arabidopsis thaliana          GGCTTTTGAGGAGCGGGAGAAATTGTTGGAATTCTATGAAAGAGTCTCGG
Brassica napus                GGCTTTTGAGGAGCGGGAGAAATTGTTGGAATTCTATGAAAGAGTCTCGG
RRII105_nad7                  GGCTTTTGAGGAGCGGGAGAAATTGTTGGAATTCTATGAAAGAGTCTCGG
PB235_nad7                    GGCTTTTGAGGAGCGGGAGAAATTGTTGGAATTCTATGAAAGAGTCTCGG
```

```
BPM24_nad7                        GGCTTTTGAGGAGCGGGAGAAATTGTTGGAATTCTATGAAAGAGTCTCGG
RRIC110_nad7                      GGCTTTTGAGGAGCGGGAGAAATTGTTGGAATTCTATGAAAGAGTCTCGG
RRIM600_nad7                      GGCTTTTGAGGAGCGGGAGAAATTGTTGGAATTCTATGAAAGAGTCTCGG
RRIT251_nad7                      GGCTTTTGAGGAGCGGGAGAAATTGTTGGAATTCTATGAAAGAGTCTCGG
Beta macrocarpa                   GGCTTTTGAGGAGCGAGAGAAATTGTTGGAATTCTATGAAAGAGTCUCGG
Beta vulgaris vulgaris            GGCTTTTGAGGAGCGAGAGAAATTGTTGGAATTCTATGAAAGAGTCUCGG
Beta vulgaris maritima            GGCTTTTGAGGAGCGAGAGAAATTGTTGGAATTCTATGAAAGAGTCUCGG
Silene latifolia                  GGCTTTTGAGGAGCGAGAGAAATTGTTGGAATTCTATGAAAGAGTCTCGG
Lotus japonicus                   GGCTTTTGAGGAGCGGGAGAAATTGTTGGAATTCTATGAAAGAGTCTCGG
Millettia pinnata                 GGCTTTTGAGGAGCGGGAGAAATTGTTGGAATTCTATGAAAGAGTCTCGG
Oryza sativa Japonica             GGCTTTTGAGGAGCGGGAGAAATTGTTGGAATTCTATGAAAGAGTCCUGG
                                  *************** ******** *********************  **

Citrullus lanatus                 GAGCCAGGATGCATGCCAGTTTCATACGACCAGGTGGAGTGGCACAAGAT
Cucurbita pepo                    GAGCCAGGATGCATGCCAGTTTCATACGACCAGGTGGAGTGGCACAAGAT
Arabidopsis thaliana              GAGCCAGGATGCATGCCAGTTTCATACGACCAGGTGGAGTGGCACAAGAT
Brassica napus                    GAGCCAGGATGCATGCCAGTTTCATACGACCAGGTGGAGTGGCACAAGAT
RRII105_nad7                      GAGCCAGGATGCATGCUAGTTTCATACGACCAGGTGGAGTGGCACAAGAT
PB235_nad7                        GAGCCAGGATGCATGCUAGTTTCATACGACCAGGTGGAGTGGCACAAGAT
BPM24_nad7                        GAGCCAGGATGCATGCUAGTTTCATACGACCAGGTGGAGTGGCACAAGAT
RRIC110_nad7                      GAGCCAGGATGCATGCCAGTTTCATACGACCAGGTGGAGTGGCACAAGAT
RRIM600_nad7                      GAGCCAGGATGCATGCNAGTTTCATACGACCAGGTGGAGTGGCACAAGAT
RRIT251_nad7                      GAGCCAGGATGCATGCCAGTTTCATACGACCAGGTGGAGTGGCACAAGAT
Beta macrocarpa                   GAGCCAGGATGCATGCCAGTTTCATACGACCAGGTGGAGTGGCACAAGAT
Beta vulgaris vulgaris            GAGCCAGGATGCATGCCAGTTTCATACGACCAGGTGGAGTGGCACAAGAT
Beta vulgaris maritima            GAGCCAGGATGCATGCCAGTTTCATACGACCAGGTGGAGTGGCACAAGAT
Silene latifolia                  GAGCCAGGATGCATGCCAGTTTCATACGACCAGGTGGAGTGGCACAAGAT
Lotus japonicus                   GAGCCAGGATGCATGCCAGTTTCATACGACCGGGTGGAGTGGCACAAGAT
Millettia pinnata                 GAGCCAGGATGCATGCCAGTTTCATACGACCAGGTGGAGTGGCACAAGAT
Oryza sativa Japonica             GAGCCAGGATGCATGCCAGTTTCATACGACCTGGTGGAGTGGCACAAGAT
                                  **************** ************** ******************

Citrullus lanatus                 CTGCCTCTTGGCTTATGTCGAGATATTGATTCCTTCACACAACAATTTGC
Cucurbita pepo                    CTTCCTCTTGGCTTATGTCGAGATATTGATTCCTTCACACAACAATTTGC
Arabidopsis thaliana              CTGCCTCTTGGCTTATGTCGAGATATTGATTCCTTCACACAACAATTTGC
Brassica napus                    CTGCCTCTTGGCTTATGTCGAGATATTGATTCCTTCACACAACAATTTGC
RRII105_nad7                      CTGCCTCTTGGCTTATGTCGAGATATTGATTCCTTCACACAACAATTTGC
PB235_nad7                        CTGCCTCTTGGCTTATGTCGAGATATTGATTCCTTCACACAACAATTTGC
BPM24_nad7                        CTGCCTCTTGGCTTATGTCGAGATATTGATTCCTTCACACAACAATTTGC
RRIC110_nad7                      CTGCCTCTTGGCTTATGTCGAGATATTGATTCCTTCACACAACAATTTGC
RRIM600_nad7                      CTGCCTCTTGGCTTATGTCGAGATATTGATTCCTTCACACAACAATTTGC
RRIT251_nad7                      CTGCCTCTTGGCTTATGTCGAGATATTGATTCCTTCACACAACAATTTGC
Beta macrocarpa                   CTGCCTCTTGGCTTATGTCGAGATATTGATTCCTUCACACAACAATTTGC
Beta vulgaris vulgaris            CTGCCTCTTGGCTTATGTCGAGATATTGATTCCTUCACACAACAATTTGC
Beta vulgaris maritima            CTGCCTCTTGGCTTATGTCGAGATATTGATTCCTUCACACAACAATTTGC
Silene latifolia                  CTGCCTCTTGGCTTATGTCGAGATATTGATTCCTUCACACAACAATTTGC
Lotus japonicus                   CTGCCTCTTGGCTTATGTCGAGATATTGATTCCTTCACACAACAATTTGC
Millettia pinnata                 CTGCCTCTTGGCTTATGTCGAGATATTGATTCCTTCACACAACAATTTGC
Oryza sativa Japonica             CTGCCTCTTGGCTTATGTCGAGATATTGATTCCTUCACACAACAATTTGC
                                  ** ******************************* ***************

Citrullus lanatus                 TTCTCGTATCGACGAATTAGAAGAGATGTUAACCGGCAACCGTATCTGGA
Cucurbita pepo                    TTCTCGTATCGACGAATTAGAAGAGATGTUAACCGGCAACCGTATCTGGA
Arabidopsis thaliana              TTCTCGTATCGATGAATTAGAAGAGATGTUAACCGGCAACCGTATCTGGA
Brassica napus                    TTCTCGTATCGATGAATTAGAAGAGATGTUAACCGGCAACCGTATCTGGA
RRII105_nad7                      TTCTCGTATCGACGAATTAGAAGAGATGTUAACCGGCAACCGTATCTGGA
PB235_nad7                        TTCTCGTATCGACGAATTAGAAGAGATGTUAACCGGCAACCGTATCTGGA
BPM24_nad7                        TTCTCGTATCGACGAATTAGAAGAGATGTUAACCGGCAACCGTATCTGGA
RRIC110_nad7                      TTCTCGTATCGACGAATTAGAAGAGATGTUAACCGGCAACCGTATCTGGA
RRIM600_nad7                      TTCTCGTATCGACGAATTAGAAGAGATGTUAACCGGCAACCGTATCTGGA
RRIT251_nad7                      TTCTCGTATCGACGAATTAGAAGAGATGTUAACCGGCAACCGTATCTGGA
Beta macrocarpa                   TTCTCGTATCGACGAATTAGAAGAGATGTUAACCGGCAACCGTATCTGGA
```

```
Beta vulgaris vulgaris      TTCTCGTATCGACGAATTAGAAGAGATGTUAACCGGCAACCGTATCTGGA
Beta vulgaris maritima      TTCTCGTATCGACGAATTAGAAGAGATGTUAACCGGCAACCGTATCTGGA
Silene latifolia            TTCTCGTATCGACGAATTAGAAGAGATGTUAACCGGCAACCGTATCTGGA
Lotus japonicus             TTCTCGTATCGACGAATTAGAAGAGATGTUAACCGGCAACCGTATCTGGA
Millettia pinnata           TTCTCGTATCGACGAATTAGAAGAGATGTUAACCGGCAACCGTATCTGGA
Oryza sativa Japonica       TTCTCGTATCGACGAATTAGAAGAGATGTUAACCGGCAACCGTATCTGGA
                            ************ *************************************

Citrullus lanatus           AACAACGATTAGTGGATATTGGTACTGTCACTGCACAGCAAGCAAAGGAT
Cucurbita pepo              AACAACGATTAGTGGATATTGGTACTGTCACTGCACAGCAAGCAAAGGAT
Arabidopsis thaliana        AACAACGATTAGTGGATATTGGTACTGTCACTGCACAGCAAGCAAAGGAT
Brassica napus              AACAACGATTAGTGGATATTGGTACTGTCACTGCACAGCAAGCAAAGGAT
RRII105_nad7                AACAACGATTAGTGGATATTGGTACTGTCACTGCACAGCAAGCAAAGGAT
PB235_nad7                  AACAACGATTAGTGGATATTGGTACTGTCACTGCACAGCAAGCAAAGGAT
BPM24_nad7                  AACAACGATTAGTGGATATTGGTACTGTCACTGCACAGCAAGCAAAGGAT
RRIC110_nad7                AACAACGATTAGTGGATATTGGTACTGTCACTGCACAGCAAGCAAAGGAT
RRIM600_nad7                AACAACGATTAGTGGATATTGGTACTGTCACTGCACAGCAAGCAAAGGAT
RRIT251_nad7                AACAACGATTAGTGGATATTGGTACTGTCACTGCACAGCAAGCAAAGGAT
Beta macrocarpa             AACAACGATTAGTGGATATTGGTACTGTCACTGCGCAGCAAGCAAAGGAT
Beta vulgaris vulgaris      AACAACGATTAGTGGATATTGGTACTGTCACTGCGCAGCAAGCAAAGGAT
Beta vulgaris maritima      AACAACGATTAGTGGATATTGGTACTGTCACTGCGCAGCAAGCAAAGGAT
Silene latifolia            AACAACGATTGGTGGATATTGGTACTGTCACTGCACAGCAAGCAAAGGAT
Lotus japonicus             AACAACGATTAGTGGATATTGGTACTGTCACCGCACAGCAAGCAAAGGAT
Millettia pinnata           AACAACGATTAGTGGATATTGGTACTGTCACTGCACAGCAAGCAAAGGAT
Oryza sativa Japonica       AACAACGATTAGTGGATATTGGTACTGTCACTGCACAGCAAGCAAAGGAT
                            ********** ******************** ** ***************

Citrullus lanatus           TGGGGATTCAGTGGTGTAATGTTAAGAGGTUCAGGGGTATGCTGGGATTT
Cucurbita pepo              TGGGGATTCAGTGGTGTAATGTTAAGAGGTUCAGGGGTATGCTGGGATTT
Arabidopsis thaliana        TGGGGATTCAGTGGTGTAATGTTAAGAGGTCCAGGGGTATGCTGGGATTU
Brassica napus              TGGGGATTCAGTGGTGTAATGTTAAGAGGTUCAGGGGTATGCTGGGATTU
RRII105_nad7                TGGGGATTCAGTGGTGTAATGTTAAGAGGTCCAGGGGTATGCTGGGATTT
PB235_nad7                  TGGGGATTCAGTGGTGTAATGTTAAGAGGTCCAGGGGTATGCTGGGATTT
BPM24_nad7                  TGGGGATTCAGTGGTGTAATGTTAAGAGGTCCAGGGGTATGCTGGGATTT
RRIC110_nad7                TGGGGATTCAGTGGTGTAATGTTAAGAGGTCCAGGGGTATGCTGGGATTT
RRIM600_nad7                TGGGGATTCAGTGGTGTAATGTTAAGAGGTCCAGGGGTATGCTGGGATTT
RRIT251_nad7                TGGGGATTCAGTGGTGTAATGTTAAGAGGTCCAGGGGTATGCTGGGATTT
Beta macrocarpa             TGGGGCTTCAGTGGTGTAATGTTAAGAGGTUCAGGGGTATGCTGGGATTT
Beta vulgaris vulgaris      TGGGGCTTCAGTGGTGTAATGTTAAGAGGTUCAGGGGTATGCTGGGATTT
Beta vulgaris maritima      TGGGGCTTCAGTGGTGTAATGTTAAGAGGTUCAGGGGTATGCTGGGATTT
Silene latifolia            TGGGGATTCAGTGGTGTAATGTTAAGAGGTUCAGGGGTATGCTGGGATTT
Lotus japonicus             TGGGGATTCAGTGGTGTAATGTTAAGAGGTUCAGGGGTATGCTGGGATTU
Millettia pinnata           TGGGGATTCAGTGGTGTAATGTTAAGAGGTUCAGGGGTATGCTGGGATTU
Oryza sativa Japonica       TGGGGATTCAGTGGTGTAATGTTAAGAGGTUCTGGGGTATGCTGGGATTU
                            ***** ************************ * **************** 

Citrullus lanatus           GCGAAGAGCTGCACCTTACGATGTTUATGACCAATTGGATUUTGACGTAC
Cucurbita pepo              GCGAAGAGCAGCACCTTACGATGTTUATGACCAATTGGATUUTGACGTAC
Arabidopsis thaliana        GCGAAGAGCAGCACCTTACGATGTTUATGACCAATUGGATUTTGACGTAC
Brassica napus              GCGAAGAGCAGCACCTTACGATGTTUATGACCAATUGGATUUTGACGTAC
RRII105_nad7                GCGAAAAGCAGCACCTTACGATGTTCATGACCAATTGGATCCTGACGTAC
PB235_nad7                  GCGAAAAGCAGCACCTTACGATGTTCATGACCAATTGGATCCTGACGTAC
BPM24_nad7                  GCGAAAAGCAGCACCTTACGATGTTCATGACCAATTGGATCCTGACGTAC
RRIC110_nad7                GCGAAAAGCAGCACCTTACGATGTTCATGACCAATTGGATCCTGACGTAC
RRIM600_nad7                GCGAAAAGCAGCACCTTACGATGTTCATGACCAATTGGATCCTGACGTAC
RRIT251_nad7                GCGAAAAGCAGCACCTTACGATGTTCATGACCAATTGGATCCTGACGTAC
Beta macrocarpa             GCGAAGAGCAGCACCTTACGATGTTTATGACCAATTGGATTTTGACGTAC
Beta vulgaris vulgaris      GCGAAGAGCAGCACCTTACGATGTTTATGACCAATTGGATTTTGACGTAC
Beta vulgaris maritima      GCGAAGAGCAGCACCTTACGATGTTTATGACCAATTGGATTTTGACGTAC
Silene latifolia            GCGAAGAGCAGCACCTTACGATGTTTATCACCAATTGGATTTTGACGTAC
Lotus japonicus             GCGAAGAGCAGCACCTTACGATGTTUATGACCAATUGGATUUTGACGTAC
Millettia pinnata           GCGAAGAGCAGCACCTTACGATGTTUATGACCAATUGGATUUTGACGTAC
```

```
Oryza sativa Japonica          GCGAAGAGCAGCACCTTACGATGTTUATGACCAATUGGATUTTGACGTAC
                               ***** *** *************** ** ****** ****  ********

Citrullus lanatus              CAGTAGGTACCAGAGGAGATUGCTATGATCGTTACTGTATUCGTATUGAA
Cucurbita pepo                 CCGTAGGTACCAGAGGAGATTGCTATGATCGTTACTGTATUCGTATCGAA
Arabidopsis thaliana           CAGTAGGTACCAGAGGAGATUGCTATGATCGTTACTGTATUCGTATUGAA
Brassica napus                 CAGTAGGTACCAGAGGAGATUGCTATGATCGTTACTGTATCCGTATCGAA
RRII105_nad7                   CAGTAGGTACCAGAGGAGATCGCTATGATCGTTACTGTATCCGTATCGAA
PB235_nad7                     CAGTAGGTACCAGAGGAGATCGCTATGATCGTTACTGTATCCGTATCGAA
BPM24_nad7                     CAGTAGGTACCAGAGGAGATCGCTATGATCGTTACTGTATCCGTATCGAA
RRIC110_nad7                   CAGTAGGTACCAGAGGAGATCGCTATGATCGTTACTGTATCCGTATCGAA
RRIM600_nad7                   CAGTAGGTACCAGAGGAGATCGCTATGATCGTTACTGTATCCGTATCGAA
RRIT251_nad7                   CAGTAGGTACCAGAGGAGATCGCTATGATCGTTACTGTATCCGTATCGAA
Beta macrocarpa                CAGTAGGTACCAGAGGAGATUGCTATGATCGTTACTGTATCCGTATCGAA
Beta vulgaris vulgaris         CAGTAGGTACCAGAGGAGATUGCTATGATCGTTACTGTATCCGTATCGAA
Beta vulgaris maritima         CAGTAGGTACCAGAGGAGATUGCTATGATCGTTACTGTATCCGTATCGAA
Silene latifolia               CAGTAGGTACCAGAGGAGATUGCTATGATCGTTACTGTATCCGTATCGAA
Lotus japonicus                CAGTAGGTACCAGAGGAGATUGCTATGATCGTTACTGTATCCGTATGGAA
Millettia pinnata              CAGTAGGTACCAGAGGAGATUGCTATGATCGTTACTGTATCCGTATTGAA
Oryza sativa Japonica          CAGTAGGTACCAGAGGAGATUGCTATGATCGTTACTGTATCCGTATCGAA
                               * ****************** ******************* ***** ***

Citrullus lanatus              GAGATGCGACAAAGTGTTCGGATCATTGTGCAATGTCUTAATCAAATGCC
Cucurbita pepo                 GAGATGCGACAAAGTGTTCGTATCATTGTGCAATGTCUTAATCAAATGCC
Arabidopsis thaliana           GAGATGCGACAAAGTCTTCGGATCATTGTGCAATGTCTTAATCAAATGCC
Brassica napus                 GAGATGCGACAAAGTCTTCGGATCATTGTGCAATGTCTTAATCAAATGCC
RRII105_nad7                   GAGATGCGACAAAGTGTTCGGATCATTGTGCAATGTCUTAATAAAATGCC
PB235_nad7                     GAGATGCGACAAAGTGTTCGGATCATTGTGCAATGTCUTAATAAAATGCC
BPM24_nad7                     GAGATGCGACAAAGTGTTCGGATCATTGTGCAATGTCUTAATAAAATGCC
RRIC110_nad7                   GAGATGCGACAAAGTGTTCGGATCATTGTGCAATGTCUTAATAAAATGCC
RRIM600_nad7                   GAGATGCGACAAAGTGTTCGGATCATTGTGCAATGTCUTAATAAAATGCC
RRIT251_nad7                   GAGATGCGACAAAGTGTTCGGATCATTGTGCAATGTCUTAATAAAATGCC
Beta macrocarpa                GAGATGCGACAAAGTGTTCGGATCATTGTGCAATGTCTTAATCAAATGCC
Beta vulgaris vulgaris         GAGATGCGACAAAGTGTTCGGATCATTGTGCAATGTCTTAATCAAATGCC
Beta vulgaris maritima         GAGATGCGACAAAGTGTTCGGATCATTGTGCAATGTCTTAATCAAATGCC
Silene latifolia               GAGATGCGACAAAGTGTTCGGATCATTGTGCAATGTCTTAATAAAATGCC
Lotus japonicus                GAGATGCGACAAAGTCTGCGGATAATTTTGCAATGTCUTAATAAAATGCC
Millettia pinnata              GAGATGCGGCAAAGTCTGCGGATAATTTTGCAATGTCUTAATAAAATGCC
Oryza sativa Japonica          GAGATGCGACAAAGTGTTCGGATCATTGTGCAATGTCUTAATCAAATGCC
                               ******** ****** * ** ** *** ********* **** *******

Citrullus lanatus              TAGTGGCATGATCAAAGCCGATGATCGTAAGCTATGTCCTCCATCACGAT
Cucurbita pepo                 TAGTGGCATGATCAAAGCCGATGATCGTAAGCTATGTCCTCCATCACGAT
Arabidopsis thaliana           TAGCGGCATGATCAAAGCCGATGATCGTAAGCTATGTCCTCCATCACGAT
Brassica napus                 TAGCGGCATGATCAAAGCCGATGATCGTAAGCTATGTCCTCCATCACGAT
RRII105_nad7                   TAGTGGCATGATCAAAGCCGATGATCGTAAGCTATGTCCTCCATCACGAT
PB235_nad7                     TAGTGGCATGATCAAAGCCGATGATCGTAAGCTATGTCCTCCATCACGAT
BPM24_nad7                     TAGTGGCATGATCAAAGCCGATGATCGTAAGCTATGTCCTCCATCACGAT
RRIC110_nad7                   TAGTGGCATGATCAAAGCCGATGATCGTAAGCTATGTCCTCCATCACGAT
RRIM600_nad7                   TAGTGGCATGATCAAAGCCGATGATCGTAAGCTATGTCCTCCATCACGAT
RRIT251_nad7                   TAGTGGCATGATCAAAGCCGATGATCGTAAGCTATGTCCTCCATCACGAT
Beta macrocarpa                TAGTGGCATGATCAAAGCCGATGATCGTAAGCTATGTCCTCCATCACGAT
Beta vulgaris vulgaris         TAGTGGCATGATCAAAGCCGATGATCGTAAGCTATGTCCTCCATCACGAT
Beta vulgaris maritima         TAGTGGCATGATCAAAGCCGATGATCGTAAGCTATGTCCTCCATCACGAT
Silene latifolia               TAGTGGCATGATCAAAGCCGATGATCGTAAGCTATGTCCTCCATCACGAT
Lotus japonicus                TAGTGGCATGATCAAAGCCGATGATCGTAAGTTATGTCCTCCATCACGAT
Millettia pinnata              TAGTGGCATGATCAAAGCCGATGATCGTAAGTTATGTCCTCCATCACGAT
Oryza sativa Japonica          TAGTGGCATGATCAAAGCCGATGATCGTAAGCTATGTCCTCCATCACGAT
                               *** *************************** ******************

Citrullus lanatus              CTCGAATGAAACTATCCATGGAATCCTUAATTCACCATTTCGAACTTTAT
Cucurbita pepo                 CTCGAATGAAACTATCCATGGAATCCTUAATTCACCATTTCGAACTTTAT
```

```
Arabidopsis thaliana          GTCGAATGAAACTATCCATGGAATCCTCAATTCACCATTTCGAACTTTAT
Brassica napus                GTCGAATGAAACTATCCATGGAATCCTUAATTCACCATTTCGAACTTTAT
RRII105_nad7                  GTCGAATGAAACTATCCATGGAATCCTCAATTCACCATTTCGAACTTTAT
PB235_nad7                    GTCGAATGAAACTATCCATGGAATCCTCAATTCACCATTTCGAACTTTAT
BPM24_nad7                    GTCGAATGAAACTATCCATGGAATCCTCAATTCACCATTTCGAACTTTAT
RRIC110_nad7                  GTCGAATGAAACTATCCATGGAATCCTCAATTCACCATTTCGAACTTTAT
RRIM600_nad7                  GTCGAATGAAACTATCCATGGAATCCTCAATTCACCATTTCGAACTTTAT
RRIT251_nad7                  GTCGAATGAAACTATCCATGGAATCCTCAATTCACCATTTCGAACTTTAT
Beta macrocarpa               ATCGAATGAAACTATCCATGGAATCCTUAATTCACCATTTCGAACUTTAT
Beta vulgaris vulgaris        ATCGAATGAAACTATCCATGGAATCCTUAATTCACCATTTCGAACUTTAT
Beta vulgaris maritima        ATCGAATGAAACTATCCATGGAATCCTUAATTCACCATTTCGAACUTTAT
Silene latifolia              ATCGAATGAAACTATCCATGGAATCCTUAATTCACCATTTCGAACUTTAT
Lotus japonicus               GTCGAATGAAACTATCCATGGAATCCTUAATTCACCATTTCGAACTTTAT
Millettia pinnata             GTCGAATGAAACTATCCATGGAATCCTUAATTCACCATTTCGAACTTTAT
Oryza sativa Japonica         CTCGAATGAAACTATCCATGGAATCCTUAATTCACCATTTCGAACTTTAT
                               ************************** ***************** ****

Citrullus lanatus             ACAGAAGGTTTTTCUGTACCAGCTTCTTCTACCTATACCGCAGTGGAAGC
Cucurbita pepo                ACAGAAGGTTTTTCUGTACCAGCTTCTTCTACCTATACCGCAGTGGAAGC
Arabidopsis thaliana          ACAGAAGGTTTTTCUGTACCAGCTTCTTCTACCTATACCGCAGTTGAAGC
Brassica napus                ACAGAAGGTTTTTCUGTACCAGCTTCTTCTACCTATACCGCAGTTGAAGC
RRII105_nad7                  ACAGAAGGTTTTTCUGTACCAGCTTCTTCTACCTATACCGCAGTTGAAGC
PB235_nad7                    ACAGAAGGTTTTTCUGTACCAGCTTCTTCTACCTATACCGCAGTTGAAGC
BPM24_nad7                    ACAGAAGGTTTTTCUGTACCAGCTTCTTCTACCTATACCGCAGTTGAAGC
RRIC110_nad7                  ACAGAAGGTTTTTCUGTACCAGCTTCTTCTACCTATACCGCAGTTGAAGC
RRIM600_nad7                  ACAGAAGGTTTTTCNGTACCAGCTTCTTCTACCTATACCGCAGTTGAAGC
RRIT251_nad7                  ACAGAAGGTTTTTCCGTACCAGCTTCTTCTACCTATACCGCAGTTGAAGC
Beta macrocarpa               ACAGAAGGGTTTTCCGTACCAGCTUCTTCTACCTATACCGCAGTTGAAGC
Beta vulgaris vulgaris        ACAGAAGGGTTTTCCGTACCAGCTUCTTCTACCTATACCGCAGTTGAAGC
Beta vulgaris maritima        ACAGAAGGGTTTTCCGTACCAGCTUCTTCTACCTATACCGCAGTTGAAGC
Silene latifolia              ACAGAAGGTTTTTCUGTACCAGCTTCTTCTACCTATACCGCAGTTGAAGC
Lotus japonicus               ACAGAAGGTTTTTCCGTACCAGCTUCTTCTACCTATACCGCAGTTGAAGC
Millettia pinnata             ACAGAAGGTTTTTCCGTACCAGCTUCTTCTACCTATACCGCAGTTGAAGC
Oryza sativa Japonica         ACAGAAGGTTTTTCCGTACCAGCTUCTTCTACCTATACCGCAGTTGAAGC
                              ******** ***** ********* ******************* *****

Citrullus lanatus             ACCTAAAGGTGAATTTGGTGTCTTTCTGGTCAGTAATGGAAGCAATCGTC
Cucurbita pepo                ACCTAAAGGGGAATTTGGTGTCTTTCTGGTCAGTAATGGAAGCAATCGTC
Arabidopsis thaliana          ACCTAAAGGAGAATTTGGTGTCTTTCTGGTCAGTAATGGAAGCAATCGTC
Brassica napus                ACCTAAAGGAGAATTTGGTGTCTTTCTGGTCAGTAATGGAAGCAATCGTC
RRII105_nad7                  ACCTAAAGGAGAATTTGGTGTCTTTCTGGTCAGTAATGGAAGCAATCGTC
PB235_nad7                    ACCTAAAGGAGAATTTGGTGTCTTTCTGGTCAGTAATGGAAGCAATCGTC
BPM24_nad7                    ACCTAAAGGAGAATTTGGTGTCTTTCTGGTCAGTAATGGAAGCAATCGTC
RRIC110_nad7                  ACCTAAAGGAGAATTTGGTGTCTTTCTGGTCAGTAATGGAAGCAATCGTC
RRIM600_nad7                  ACCTAAAGGAGAATTTGGTGTCTTTCTGGTCAGTAATGGAAGCAATCGTC
RRIT251_nad7                  ACCTAAAGGAGAATTTGGTGTCTTTCTGGTCAGTAATGGAAGCAATCGTC
Beta macrocarpa               ACCTAAAGGAGAATTTGGTGTCTTTCTGGTCAGTAATGGAAGCAATCGTC
Beta vulgaris vulgaris        ACCTAAAGGAGAATTTGGTGTCTTTCTGGTCAGTAATGGAAGCAATCGTC
Beta vulgaris maritima        ACCTAAAGGAGAATTTGGTGTCTTTCTGGTCAGTAATGGAAGCAATCGTC
Silene latifolia              ACCTAAAGGAGAATTTGGTGTCTTTCTGGTCAGTAATGGAAGCAATCGTC
Lotus japonicus               ACCTAAAGGAGAATTTGGTGTCTTTCTGGTCAGTAATGGAAGCAATCGTC
Millettia pinnata             ACCTAAAGGAGAATTTGGTGTCTTTCTGGTCAGTAATGGAAGCAATCGTC
Oryza sativa Japonica         ACCTAAAGGTGAATTTGGTGTCTTTCTTGTCAGTAATGGGAGTAATCGTC
                              ********* ***************** *********** ** *******

Citrullus lanatus             CTTACCGTUGTAAAATAAGAGCACCTGGCTUTGCCCATTUACAAGGACTC
Cucurbita pepo                CUTACCGTUGTAAAATAAGAGCACCTGGCTUTGCCCATTUACAAGGACTC
Arabidopsis thaliana          CUTACCGTUGTAAAATAAGAGCACCCGGCTUTGCCCATTUACAAGGACTC
Brassica napus                CUTACCGTUGTAAAATAAGAGCACCCGGCTTTGCCCATTUACAAGGACTC
RRII105_nad7                  CUTACCGTUGTAAAATAAGAGCACCTGGCTCTGCCCATTCACAAGGACTC
PB235_nad7                    CUTACCGTUGTAAAATAAGAGCACCTGGCTCTGCCCATTCACAAGGACTC
BPM24_nad7                    CUTACCGTUGTAAAATAAGAGCACCTGGCTCTGCCCATTCACAAGGACTC
```

```
RRIC110_nad7               CUTACCGTNGTAAAATAAGAGCACCTGGCTCTGCCCATTCACAAGGACTC
RRIM600_nad7               CNTACCGTNGTAAAATAAGAGCACCTGGCTCTGCCCATTCACAAGGACTC
RRIT251_nad7               CUTACCGTUGTAAAATAAGAGCACCTGGCTCTGCCCATTCACAAGGACTC
Beta macrocarpa            CTTACCGTUGTAAAATAAGAGCACCTGGCTUTGCCCATTUACAAGGACTC
Beta vulgaris vulgaris     CTTACCGTUGTAAAATAAGAGCACCTGGCTUTGCCCATTUACAAGGACTC
Beta vulgaris maritima     CTTACCGTUGTAAAATAAGAGCACCTGGCTUTGCCCATTUACAAGGACTC
Silene latifolia           CTTACCGTUGTAAAATAAGAGCACCTGGCTUTGCCCATTUACAAGGACTC
Lotus japonicus            CUTACCGTUGTAAAATAAGAGCACCTGGCTUTGCCCATTUACAAGGACTC
Millettia pinnata          CUTACCGTUGTAAAATAAGAGCACCTGGCTUTGCCCATTUACAAGGACTC
Oryza sativa Japonica      CCTACCGTTGTAAAATAAGAGCACCTGGCTTTGCCCATTUACAAGGACTC
                           * ****** **************** **** ******** **********

Citrullus lanatus          GATTUTATGTCCAAACATCACATGCUAGCGGATGTGGTCACCATCATAGG
Cucurbita pepo             GATTUTATGGCCAAACATCACATGCUAGCAGATGTAGTCACCATCATTGG
Arabidopsis thaliana       GATTUTATGTCCAAACATCACATGCUAGCAGATGTGGTUACCATCATAGG
Brassica napus             GATTUTATGTCCAAACATCACATGCUAGCAGATGTGGTUACCATCATAGG
RRII105_nad7               GATTCTATGTCCAAACATCACATGCUAGCAGATGTGGTUACCATCATAGG
PB235_nad7                 GATTCTATGTCCAAACATCACATGCUAGCAGATGTGGTUACCATCATAGG
BPM24_nad7                 GATTCTATGTCCAAACATCACATGCUAGCAGATGTGGTUACCATCATAGG
RRIC110_nad7               GATTCTATGTCCAAACATCACATGCUAGCAGATGTGGTUACCATCATAGG
RRIM600_nad7               GATTCTATGTCCAAACATCACATGCNAGCAGATGTGGTNACCATCATAGG
RRIT251_nad7               GATTCTATGTCCAAACATCACATGCUAGCAGATGTGGTUACCATCATAGG
Beta macrocarpa            GATTUTATGTCCAAACATCACATGCUAGCCGATGTGGTCACCATCATAGG
Beta vulgaris vulgaris     GATTUTATGTCCAAACATCACATGCUAGCCGATGTGGTCACCATCATAGG
Beta vulgaris maritima     GATTUTATGTCCAAACATCACATGCUAGCCGATGTGGTCACCATCATAGG
Silene latifolia           GATTUTATGTCCAAACATCACATGCUAGCCGATGTGGTCACCATCATAGG
Lotus japonicus            GATTUTATGTCCAAACATCACATGCUAGCAGATGTGGTCACCATCATAGG
Millettia pinnata          GATTUTATGTCCAAACATCACATGCUAGCAGATGTGGTCACCATCTTAGG
Oryza sativa Japonica      GATTUTATGTCUAAACATCACATGCUAGCAGATGTAGTCACCATCATAGG
                           **** **** * ************* *** ***** ** ****** * **

Citrullus lanatus          TACTCAAGATATTGTGTCTGGAGAGGTAGATAGATAG
Cucurbita pepo             TACTCAAGATATTGTGTCTGGAGAGGTAGATAGATAG
Arabidopsis thaliana       TACTCAAGATATTGTGTTTGGAGAGGTGGATAGATAG
Brassica napus             TACTCAAGATATTGTGTTTGGAGAGGTGGATAGATAG
RRII105_nad7               TACTCAAGATATTGTGTTTGGAGAGGTGGATAGATAG
PB235_nad7                 TACTCAAGATATTGTGTTTGGAGAGGTGGATAGATAG
BPM24_nad7                 TACTCAAGATATTGTGTTTGGAGAGGTGGATAGATAG
RRIC110_nad7               TACTCAAGATATTGTGTTTGGAGAGGTGGATAGATAG
RRIM600_nad7               TACTCAAGATATTGTGTTTGGAGAGGTGGATAGATAG
RRIT251_nad7               TACTCAAGATATTGTGTTTGGAGAGGTGGATAGATAG
Beta macrocarpa            TACTCAAGATATTGTGTUTGGAGAGGTGGATAGATAG
Beta vulgaris vulgaris     TACTCAAGATATTGTGTUTGGAGAGGTGGATAGATAG
Beta vulgaris maritima     TACTCAAGATATTGTGTUTGGAGAGGTGGATAGATAG
Silene latifolia           TACTCAAGATATTGTGTUTGGAGAGGTGGATAGATAG
Lotus japonicus            TACTCAAGATATTGTGTUTGGAGAGGTGGATAGATAG
Millettia pinnata          TACTCAAGATATTGTGTUTGGAGAGGTGGATAGATAG
Oryza sativa Japonica      TACTCAAGATATTGTGTTTGGAGAGGTAGATAGATAG
                           ***************** ********* *********
```

###################

nad9 multiple sequence alignment

```
Arabidopsis thaliana       ------ACCAACCGGCACCGGCAATTTCCGACGA----GTCTTTCAGAAT
Brassica napus             ------ACCAACCGGCACCGGCAATTTCCGACGA----GTCTTTCAGAAT
Beta macrocarpa            --GGTTGGAAAACCCAACCGGTGATTTCCGACAA----GTCTTTCTTCAT
Beta vulgaris maritima     --GGTTGGAAAACCCAACCGGTGATTTCCGACAA----GTCTTTCTTCAT
Beta vulgaris vulgaris     --------------------------------------------------
Silene latifolia           AGCCCATTTTAATTTAACAACAGATTTACGACAA----GTCTTCCCTTAT
Oryza sativa Japonica      ----CGGACCAACCCAACCGGCGATTTCCGACAA----GTCTTTC----T
PB235_nad9                 --------------------------------------------------
Citrullus lanatus          ------------GCGATTGATTGATTTCCTACAAGTCAGTCTTCCTGAAT
Cucurbita pepo             --------CCAAGCCAACCGGCGATTTCCGACAAACAAGTCTTTCCGCAT
```

```
BPM24_nad9                        --------------------------------------------------
Millettia pinnata                 ---------------AACCGGCGATTTACAACAAAGAAGTCTTTCCTCAT
Millettia pinnata                 ---------------AACCGGCGATTTACAACAAAGAAGTCTTTCCTCAT
Lotus japonicus                   -------------CCAACCGGCGATTTACAACAAACAAGTCTTTCTTCAT
RRIC110_nad9                      --------------------------------------------------
RRIT251_nad9                      --------------------------------------------------
RRII105_nad9                      --------------------------------------------------
RRIM600_nad9                      --------------------------------------------------
Megaceros aenigmaticus            --------------------------------------------------
Phaeoceros laevis                 --------------------------------------------------
Huperzia squarrosa                ------------------TGGGATATTATAACTTTTCGTTGGTTCAGAGG


Arabidopsis thaliana              TTGAA----GAGCAAG-----AAGCGGAACTACAAGAAAGCTTTCTTTAT
Brassica napus                    TTGAA----GAGCAAG-----AAGCGGAACTACAAGAAAGCTTTCTTTAT
Beta macrocarpa                   TTTT-----GAGCAAG-----AAGCGGAACTACAAGAAAGCTTTCTTTCT
Beta vulgaris maritima            TTTT-----GAGCAAG-----AAGCGGAACTACAAGAAAGCTTTCTTTCT
Beta vulgaris vulgaris            ---------------------AAGCGGAACTACAAGAAAGCTTTCTTTCT
Silene latifolia                  TTTA-----GAGCAAG-----AAGCGGAA----AAGAAAGCTTTCTTTAT
Oryza sativa Japonica             GCTTA----GAGCAAG-----AAGCGGAACCAAAATCAAGCTTTCTTTAT
PB235_nad9                        --------------------------------------------------
Citrullus lanatus                 TGGTC----GAGCAAG-----AAGCGGAACGACAAGAAAGCTTTCTTTAT
Cucurbita pepo                    TTTTC----GAGCAAG-----AAGCGGAACTACAATCAAGCTTTCTTTAT
BPM24_nad9                        --------------------------------------------------
Millettia pinnata                 TTTTTTTTAGAGCAAGAAAAGAAGCGGAACTACAAGAAAGCTTTCTTTAT
Millettia pinnata                 TTTTTTTTAGAGCAAGAAAAGAAGCGGAACTACAAGAAAGCTTTCTTTAT
Lotus japonicus                   TTTTTT--AGAGCAAGAAAAGAAGCGGAACTACAAGAAAGCTTTCTTTAT
RRIC110_nad9                      --------------------------------------------------
RRIT251_nad9                      --------------------------------------------------
RRII105_nad9                      --------------------------------------------------
RRIM600_nad9                      --------------------------------------------------
Megaceros aenigmaticus            ------------------------------------------------AT
Phaeoceros laevis                 ------------------------------------------------AT
Huperzia squarrosa                GTAGAACTACATCTTCCTTCATGAAGATATGTTAAAAAAATACAATTAAT


Arabidopsis thaliana              CTT----TATGGATAACCAATTCATTTTCAAATATAGTTGGGAGACTTTA
Brassica napus                    CTT----TATGGATAACCAATUCATTTTCAAATATAGTTGGGAGACTTTA
Beta macrocarpa                   CTT----TATGGATAACCAATTCATTTTCAAATATAGTTGGGAGACTTTA
Beta vulgaris maritima            CTT----TATGGATAACCAATTCATTTTCAAATATAGTTGGGAGACTTTA
Beta vulgaris vulgaris            CTT----TATGGATAACCAATTCATTTTCAAATATAGTTGGGAGACTTTA
Silene latifolia                  CTT----TATGGATAACCAATTCATTTTAAAATATAGTTGGGAGACTTTG
Oryza sativa Japonica             TTTCATTTATGUATAACCAATCCATTTTCCAATATAGTTGGGAGATTTTA
PB235_nad9                        --------ATGGATAACCAATCCATTTTCAAATATAGTTGGGAAACTCTC
Citrullus lanatus                 CCTTCCTTATGGATAACCAATUCTTTTTCAAATATAGTTGGGATACTCTC
Cucurbita pepo                    CTTT----ATGGATAACCAATUCATTTTCAAATATAGTTGGGAGACTCTC
BPM24_nad9                        --------ATGGATAACCAATCCATTTTCAAATATAGTTGGGAAACTCTC
Millettia pinnata                 --------ATGGATAACCAATUCATTTTCAAATATAGTTTGGAGACTTTA
Millettia pinnata                 --------ATGGATAACCAATUCATTTTCAAATATAGTTTGGAGACTTTA
Lotus japonicus                   --------ATGGATAACCAATUCATTTTCAAATATAGTTGGGAGACTTTA
RRIC110_nad9                      --------ATGGATAACCAATCCATTTTCAAATATAGTTGGGAAACTCTC
RRIT251_nad9                      --------ATGGATAACCAATCCATTTTCAAATATAGTTGGGAAACTCTC
RRII105_nad9                      --------ATGGATAACCAATCCATTTTCAAATATAGTTGGGAAACTCTC
RRIM600_nad9                      --------ATGGATAACCAATCCATTTTCAAATATAGTTGGGAAACTCTC
Megaceros aenigmaticus            GG------ATA---AGC-AATTGTTTCTCAAATCTCTGATAGCTACTTTA
Phaeoceros laevis                 GG------ATA---AGC-AATTGTTTCTCAAATCTCTGATAGCTACTTTA
Huperzia squarrosa                TT------AUG---AACCAATTGTTTTTCAAATTTATGAAAGCTACTTTA
                                          *     * * ***   ** *  *** *      *  * * *

Arabidopsis thaliana              CCCAAGAAATGGGTCAAAAAAATGGAAAGATCGGAACATGGGAATAGATU
Brassica napus                    CCCAAGAAATGGGTCAAAAAAATGGAAAGATCGGAACATGGGAATAGATU
Beta macrocarpa                   CCCAAGAAATGGGTCAAAAAAATAGAAAAATCAGAACATGGGAATAGATU
```

```
Beta vulgaris maritima          CCCAAGAAATGGGTCAAAAAAATAGAAAAATCAGAACATGGGAATAGATU
Beta vulgaris vulgaris          CCCAAGAAATGGGTCAAAAAAATAGAAAAATCAGAACATGGGAATAGATU
Silene latifolia                CCCAAGAAATGGGTAAAAAAAATAGAAAAATCAGAACATGGGAATAGATU
Oryza sativa Japonica           CCCAAGAAATGGGTACATAAAATGAAAAGATCGGAACATGGGAATAGATU
PB235_nad9                      CCCAAGAAATGGGTAAAAAAAATGGAAAGATCGGAACATGGGAATAGATU
Citrullus lanatus               CCCAAGAAATGGGTAAAAAAAAGAGAAAGATCGGAACATGGGAATAGATU
Cucurbita pepo                  CCCAAGAAATGGGTCAAAAAAAGAGAAAGATCGGAACATGGGAATAGATU
BPM24_nad9                      CCCAAGAAATGGGTAAAAAAAATGGAAAGATCGGAACATGGGAATAGATU
Millettia pinnata               CCCAAGAAATGGGTAAAAAAAATGGAAAGATCGGAACATGGGAATAGATU
Millettia pinnata               CCCAAGAAATGGGTAAAAAAAATGGAAAGATCGGAACATGGGAATAGATU
Lotus japonicus                 CCAAAGAAATGGGTAAACAAAATGGAAAGATCGGAACATGGGAATAGATU
RRIC110_nad9                    CCCAAGAAATGGGTAAAAAAAATGGAAAGATCGGAACATGGGAATAGATU
RRIT251_nad9                    CCCAAGAAATGGGTAAAAAAAATGGAAAGATCGGAACATGGGAATAGATU
RRII105_nad9                    CCCAAGAAATGGGTAAAAAAAATGGAAAGATCGGAACATGGGAATAGATC
RRIM600_nad9                    CCCAAGAAATGGGTAAAAAAAATGGAAAGATCGGAACATGGGAATAGATN
Megaceros aenigmaticus          CC---GAAACGGATACATCAATTTAAAACATCGAAACATGAAAATATATT
Phaeoceros laevis               CC---GAAACGGATACATCAATTTAAAACATCGAAACATGAAAATATATT
Huperzia squarrosa              CC---TAAACGGATAAATCAATGTCAAACATCAAAACAAGAAAATCTATT
                                **    *** ** *  *  **    *** ***  **** *  ***  **

Arabidopsis thaliana            TGATACCAATACGGACTACCTATTTCAATTGTTGTGCTTTCTCAAATTGC
Brassica napus                  TGATACCAATACGGACTACCTATTTCAATTGTTGTGCTTTCTCAAATTGC
Beta macrocarpa                 TGATACCAATACGGACTACCUATTTCAATTGTTGTGCTTTCTTAAATTGC
Beta vulgaris maritima          TGATACCAATACGGACTACCUATTTCAATTGTTGTGCTTTCTTAAATTGC
Beta vulgaris vulgaris          TGATACCAATACGGACTACCUATTTCAATTGTTGTGCTTTCTTAAATTGC
Silene latifolia                TGATACCAATACGGACTACCUATTTCAATTATTGTGCTTTCTCAAATTGC
Oryza sativa Japonica           TTATACCAATACTGACTACCUATTTCCATTGTTGTGCTTTCTAAAATGGC
PB235_nad9                      TGATACCAATTCGGATTACCTATTTCAATTGTTGTGCTTTCTTAAATTGC
Citrullus lanatus               GGATACCAATACAGACTACCTATTTCAATTGTTGTGCTTTCTCAAATTGC
Cucurbita pepo                  GGATACCAATACGGACTACCTATTTCAATTGTTGTGCTTTCTCAAATTGC
BPM24_nad9                      TGATACCAATTCGGATTACCTATTTCAATTGTTGTGCTTTCTTAAATTGC
Millettia pinnata               TGATACCAAAACGGACTACCTATTTCAATTGTTGTGCTTTCTTAAATTGC
Millettia pinnata               TGATACCAAAACGGACTACCTATTTCAATTGTTGTGCTTTCTTAAATTGC
Lotus japonicus                 TGATACCAAAACGGACTACCTATTTCAATTGTTGTGCTTTCTTAAATTGC
RRIC110_nad9                    TGATACCAATTCGGATTACCTATTTCAATTGTTGTGCTTTCTTAAATTGC
RRIT251_nad9                    TGATACCAATTCGGATTACCTATTTCAATTGTTGTGCTTTCTTAAATTGC
RRII105_nad9                    TGATACCAATTCGGATTACCTATTTCAATTGTTGTGCTTTCTTAAATTGC
RRIM600_nad9                    TGATACCAATTCGGATTACCTATTTCAATTGTTGTGCTTTCTTAAATTGC
Megaceros aenigmaticus          ATATACCAATCCAGATTACCTGTTTGAATTACTATGGTTCCCGAAACATC
Phaeoceros laevis               ATATACCAATCCAGATTACCTGTTTGAATTACTATGGTTTCTGAAACATC
Huperzia squarrosa              ATATACCAATCCAGACTACCCATTCCAATTATTATGGTTTTTGAAATATC
                                  *******  * ** ****  **   ***  * ** **    ***   *

Arabidopsis thaliana            ATACCTATACAAGGGTTCAAGTTTUGATCGATATTTGCGGAGTTGATUAT
Brassica napus                  ATACCTATACAAGGGTTCAAGTTTUGATCGATATTTGCGGAGTTGATUAT
Beta macrocarpa                 ATACCTATACAAGGTTTCAAGTTTTGATCGATATTTGCGGAGTTGATTAT
Beta vulgaris maritima          ATACCTATACAAGGTTTCAAGTTTTGATCGATATTTGCGGAGTTGATTAT
Beta vulgaris vulgaris          ATACCTATACAAGGTTTCAAGTTTTGATCGATATTTGCGGAGTTGATTAT
Silene latifolia                ATACCTATACAAGGTTTCAAGTTTTGATCGATATTTGCGGAGTTGATTAT
Oryza sativa Japonica           ATACCTATACAAGGGTTCAAGTTTUGATCGATATTTGCGGAGTGGATUAT
PB235_nad9                      ATACCTATACAAGGGTTCAAGTTTUGATCGATATTTGCGGAGTTGATUAT
Citrullus lanatus               ATACCTATACAAGGGTTCAAGTTTUGATCGATATTTGCGGAGTTGATTAT
Cucurbita pepo                  ATACCTATACAAGGGTTCAAGTTTUGATCGATATTTGCGGAGTTGATTAT
BPM24_nad9                      ATACCTATACAAGGGTTCAAGTTTUGATCGATATTTGCGGAGTTGATUAT
Millettia pinnata               ATACCTATACAAGGGTTCAAGTTTUGATCGATATTTGCGGAGTTGATTAT
Millettia pinnata               ATACCTATACAAGGGTTCAAGTTTUGATCGATATTTGCGGAGTTGATTAT
Lotus japonicus                 ATACCTATACAAGGGTTCAAGTTTUGATCGATATTTGCGGAGTTGATTAT
RRIC110_nad9                    ATACCTATACAAGGGTTCAAGTTTUGATCGATATTTGCGGAGTTGATUAT
RRIT251_nad9                    ATACCTATACAAGGGTTCAAGTTTUGATCGATATTTGCGGAGTTGATUAT
RRII105_nad9                    ATACCTATACAAGGGTTCAAGTTTUGATCGATATTTGCGGAGTTGATUAT
RRIM600_nad9                    ATACCTATACAAGGGTTCAAGTTTNGATCGATATTTGCGGAGTTGATNAT
Megaceros aenigmaticus          ATACCAATACACGTTTTCAAGTTTTGATTGATATTCGCGGAGTTGATTAT
```

```
Phaeoceros laevis           ATATCAATACACGTTTTCAAGTTTCGATTGATATTCGCGGAGTTGATTAT
Huperzia squarrosa          ATACCAATACACGTTTTCAAGTTTCGATCGAGATTTGCGGAGTTGATTAT
                            *** * ***** *  ********* *** ** *** ******* *** **

Arabidopsis thaliana        CCCTCTCGAAAACGAAGATTTGAAGTGGTCTATAATTTACTGAGTACTCG
Brassica napus              CCCTCCCGAAAACGAAGATTTGAAGTGGTCTATAATTTACTGAGTACTCG
Beta macrocarpa             CCTTCTCGAAAACGAAGATTTGAAGTGGTCTATAATTTACTGAGTACTCG
Beta vulgaris maritima      CCTTCTCGAAAACGAAGATTTGAAGTGGTCTATAATTTACTGAGTACTCG
Beta vulgaris vulgaris      CCTTCTCGAAAACGAAGATTTGAAGTGGTCTATAATTTACTGAGTACTCG
Silene latifolia            CCTTCTCGAAAACTAAGATTTGAAGTGGTCTATAATTTACTGAGTACTCG
Oryza sativa Japonica       CCCTCTCGAAAACGAAGATTTGAAGTTGTCUATAATTTACTGAGTACTCG
PB235_nad9                  CCCTCTCGAAAACGAAGATTTGAAGTGGTCTATAATTTACTGAGTACTCG
Citrullus lanatus           CCCTCTCGAAAACGAAGATTTGAAGTGGTCTATAATTTACTGAGTACTCG
Cucurbita pepo              CCCTCTCGAAAACGCAGATTTGCAGTGGTCTATAATTTACTGAGTACTCG
BPM24_nad9                  CCCTCTCGAAAACGAAGATTTGAAGTGGTCTATAATTTACTGAGTACTCG
Millettia pinnata           CCCTCTCGAAAACGAAGATTTGAAGTGGTCTATAATTTACTGAGTACTCG
Millettia pinnata           CCCTCTCGAAAACGAAGATTTGAAGTGGTCTATAATTTACTGAGTACTCG
Lotus japonicus             CCCTCTCGAAAACGAAGATTTGAAGTGGTCTATAATTTACTGAGCACTCG
RRIC110_nad9                CCCTCTCGAAAACGAAGATTTGAAGTGGTCTATAATTTACTGAGTACTCG
RRIT251_nad9                CCCTCTCGAAAACGAAGATTTGAAGTGGTCTATAATTTACTGAGTACTCG
RRII105_nad9                CCCTCTCGAAAACGAAGATTTGAAGTGGTCTATAATTTACTGAGTACTCG
RRIM600_nad9                CCCTCTCGAAAACGAAGATTTGAAGTGGTCTATAATTTACTGAGTACTCG
Megaceros aenigmaticus      CCCTCTCGAAAACGAAGATTCGAAGTAGTTCATAATTCACCGAGTATTCG
Phaeoceros laevis           TCCTCTCGAAAACGAAGATTCGAAGTAGTTCATAATTCACTGAGTATTCG
Huperzia squarrosa          CCTTCCCGGAAACAAAGATTTGAAGTAGTTTATAATTCACTTAGTGTTGA
                             * ** ** ****  ***** * *** **  ****** **  **   *

Arabidopsis thaliana        GTATAACTCACGCATTCGTGTACAAACCAGTGCAGACGAAGTAACACGAA
Brassica napus              GTATAACTCACGCATTCGTGTACAAACCAGTGCAGACGAAGTAACACGAA
Beta macrocarpa             GTATAATTCGCGCATTCGCGTACAAACCTGTGCAGACGAAGTAACACGAA
Beta vulgaris maritima      GTATAATTCGCGCATTCGCGTACAAACCTGTGCAGACGAAGTAACACGAA
Beta vulgaris vulgaris      GTATAATTCGCGCATTCGGCTACAAACCTGTGCAGACGAAGTAACACGAA
Silene latifolia            GTATAATTCGCGCATTCGCGTACAAACCAGTGCAGACGAAGTAACACGAA
Oryza sativa Japonica       GTATAACTCACGCATTCGTGTACAAACAAGTGCAGACGAAGTAACACGAA
PB235_nad9                  GTATAACTCACGCATTCGTGTACAAACCAGTGCAGACGAAGTAACACGAA
Citrullus lanatus           GTATAACTCACGCATTCGTGTACAAACCAGTGCAGACGAAGTAACACGAA
Cucurbita pepo              GTATAACTCACGCATTCGTGTACAAACCCGTGCAGACGAAGTAACACGAA
BPM24_nad9                  GTATAACTCACGCATTCGTGTACAAACCAGTGCAGACGAAGTAACACGAA
Millettia pinnata           GTATAACTCACGCATTCGTGTACAAACCAGTGCAGACGAAGTAACACGAA
Millettia pinnata           GTATAACTCACGCATTCGTGTACAAACCAGTGCAGACGAAGTAACACGAA
Lotus japonicus             GTATAACTCACGCATTCGTGTACAAACCAGTGCAGACGAAGTAACACGAA
RRIC110_nad9                GTATAACTCACGCATTCGTGTACAAACCAGTGCAGACGAAGTAACACGAA
RRIT251_nad9                GTATAACTCACGCATTCGTGTACAAACCAGTGCAGACGAAGTAACACGAA
RRII105_nad9                GTATAACTCACGCATTCGTGTACAAACCAGTGCAGACGAAGTAACACGAA
RRIM600_nad9                GTATAACTCACGCATTCGTGTACAAACCAGTGCAGACGAAGTAACACGAA
Megaceros aenigmaticus      GTATAATTCACGTATTCGTGTAGAAAGCAGTGTAAATGAAATAACTCCAA
Phaeoceros laevis           GTATAATTCATGTATTCGTGTAGAAAGCAGTGTAAGTGAAATAACTCCAA
Huperzia squarrosa          CTATAATACACGCATACGTATATTAACAAGTTACGATGAAATAACTCCAA
                             *****  *  * ** **  **  **   **      *** **** * **

Arabidopsis thaliana        TATCTUCGGTAGTAAGTCTATTTCCATCAGCCGGCUGGTGGGAGCGAGAA
Brassica napus              TATCTUCGGTAGTAAGTCTATTTCCATCAGCCGGCUGGTGGGAGCGAGAA
Beta macrocarpa             TATCTCUGGTAGTCAGTCTATTTCCATCAGCTGGCUGGTGGGAGCGAGAA
Beta vulgaris maritima      TATCTCUGGTAGTCAGTCTATTTCCATCAGCTGGCUGGTGGGAGCGAGAA
Beta vulgaris vulgaris      TATCTCUGGTAGTCAGTCTATTTCCATCAGCTGGCUGGTGGGAGCGAGAA
Silene latifolia            TATCTUCGGTAGTCAGTCTATTTCCATCAGCCGGCUGGTGGGAGCGAGAA
Oryza sativa Japonica       TATCTUCGGTAGTCAGTCUATTTCCATCAGCCGGCUGGTGGGAGCGAGAA
PB235_nad9                  TATCTUCGGTAGTCAGTCTATTTCCATCAGCCGGCUGGTGGGAGCGAGAA
Citrullus lanatus           TATCTUCGGTAGTCAGTCTATTTCCATCAGCCGGCUGGTGGGAGCGAGAA
Cucurbita pepo              TATCTUCGGTAGTCAGTCTATTTCCATCAGCCGGCUGGTGGGAGCGAGAA
BPM24_nad9                  TATCTUCGGTAGTCAGTCTATTTCCATCAGCCGGCUGGTGGGAGCGAGAA
Millettia pinnata           TATCTUCGGTAGTCAGTCTATTTCCATCAGCCGGCUGGTGGGAGCGAGAA
```

```
Millettia pinnata                 TATCTUCGGTAGTCAGTCTATTTCCATCAGCCGGCUGGTGGGAGCGAGAA
Lotus japonicus                   TATCTUCGGTAGTCAGTCTATTTCCATCAGCCGGCUGGTGGGAGCGAGAA
RRIC110_nad9                      TATCTUCGGTAGTCAGTCTATTTCCATCAGCCGGCUGGTGGGAGCGAGAA
RRIT251_nad9                      TATCTUCGGTAGTCAGTCTATTTCCATCAGCCGGCUGGTGGGAGCGAGAA
RRII105_nad9                      TATCTUCGGTAGTCAGTCTATTTCCATCAGCCGGCUGGTGGGAGCGAGAA
RRIM600_nad9                      TATCTNCGGTAGTCAGTCTATTTCCATCAGCCGGCNGGTGGGAGCGAGAA
Megaceros aenigmaticus            TTTGTTCGGTAGTCGGTATATTTCCATCAGCCGGCTGGTGGGAGCGAGAG
Phaeoceros laevis                 TTTGTTTGGTAGTCAATATATTTCCATCAGCCGGCTGGTGGGAGCGAGAG
Huperzia squarrosa                TTTGTTCGGTAGTTGGTATATTTCCATCCGCCGGCTGGTGGGAGCGAGAA
                                  * * *  ******   *  ********* ** *** *************

Arabidopsis thaliana              GTTTGGGATATGTTTGGTGTTTCTTTCATCAATCATCCGGATCTACGCCG
Brassica napus                    GTTTGGGATATGTTTGGTGTTTCTTTCATCAATCATCCGGATCTACGCCG
Beta macrocarpa                   GTTTGGGATATGTTTGGTGTTTCTTUCATCAATCATCCGGATCTACGCCG
Beta vulgaris maritima            GTTTGGGATATGTTTGGTGTTTCTTUCATCAATCATCCGGATCTACGCCG
Beta vulgaris vulgaris            GTTTGGGATATGTTTGGTGTTTCTTUCATCAATCATCCGGATCTACGCCG
Silene latifolia                  GTTTGGGATATGTTTGGTGTTTCTTUCATCAATCATCCGGATCTACGCCG
Oryza sativa Japonica             GTATGGGATATGTUTGGTGTTTCTTUCATCAATCATCCGGATTTACGCCG
PB235_nad9                        GTTTGGGATATGTTTGGTGTTTCTTCCATCAATCATCCGGATCTACGCCG
Citrullus lanatus                 GTTTGGGATATGTTTGGTGTTTCTTUCATCAATCATCCGGATCTACGCCG
Cucurbita pepo                    GTTTGGGATATGTTTGGTGTTTCTTUCATCAATCATCCGGATCTACGCCG
BPM24_nad9                        GTTTGGGATATGTTTGGTGTTTCTTCCATCAATCATCCGGATCTACGCCG
Millettia pinnata                 GTTTGGGATATGTTTGGTCTTTCTTUCATCAATCATCCGGATCTACGCCG
Millettia pinnata                 GTTTGGGATATGTTTGGTCTTTCTTUCATCAATCATCCGGATCTACGCCG
Lotus japonicus                   GTTTGGGATATGTTTGGTGTTTCTTUCATCAATCATCCGGATCTACGCCG
RRIC110_nad9                      GTTTGGGATATGTTTGGTGTTTCTTCCATCAATCATCCGGATCTACGCCG
RRIT251_nad9                      GTTTGGGATATGTTTGGTGTTTCTTCCATCAATCATCCGGATCTACGCCG
RRII105_nad9                      GTTTGGGATATGTTTGGTGTTTCTTCCATCAATCATCCGGATCTACGCCG
RRIM600_nad9                      GTTTGGGATATGTTTGGTGTTTCTTCCATCAATCATCCGGATCTACGCCG
Megaceros aenigmaticus            GTATGGGATATGTTTGGTGCGTATTCTTCTAATCATCCTGATTCACGCCG
Phaeoceros laevis                 GTATGGGATATGTTTGGTGCGTATTCTTCTAATCATCCTGATTCACGCCG
Huperzia squarrosa                GTATGGGATATGTTTGGTGTGTTTTTTTCCAATCATCCTGATTTACGTCG
                                  ** ********** ****   * **     ******** ***  *** **

Arabidopsis thaliana              TATATUAACTGATTATGGTTTCGAGGGTCATCCATTACGAAAAGACUTTC
Brassica napus                    TATATUAACAGATTATGGTTTCGAGGGTCATCCATTACGAAAAGACUTTC
Beta macrocarpa                   TATATTAACAGATTATGGTTTCGAGGGTCATCCATTACGAAAAGACTTTC
Beta vulgaris maritima            TATATTAACAGATTATGGTTTCGAGGGTCATCCATTACGAAAAGACTTTC
Beta vulgaris vulgaris            TATATTAACAGATTATGGTTTCGAGGGTCATCCATTACGAAAAGACTTTC
Silene latifolia                  TATATTAACAGATTATGGTTTCGAGGGTCATCCATTACGAAAAGACTTTC
Oryza sativa Japonica             TATATUAACAGATTATGGTTTCGAGGGTCATCCATTACGAAAAGACTTTC
PB235_nad9                        TATATCAACAGATTATGGTTTCGAGGGTCATCCATTACGAAAAGACCTTC
Citrullus lanatus                 TATATUAACAGATTATGGTTTCGAGGGTCATCCATTACGAAAAGACUTTC
Cucurbita pepo                    TATATUAACAGATTATGGTTTCGAGGGGCATCCATTACGAAAAGACUTTC
BPM24_nad9                        TATATCAACAGATTATGGTTTCGAGGGTCATCCATTACGAAAAGACCTTC
Millettia pinnata                 TATATUAACAGATTATGGTTTCGAGGGTCATCCATTACGAAAAGACUTTC
Millettia pinnata                 TATATUAACAGATTATGGTTTCGAGGGTCATCCATTACGAAAAGACUTTC
Lotus japonicus                   TATATUAACAGATTATGGTTTCGAGGGTCATCCATTACGAAAAGACUTTC
RRIC110_nad9                      TATATCAACAGATTATGGTTTCGAGGGTCATCCATTACGAAAAGACCTTC
RRIT251_nad9                      TATATCAACAGATTATGGTTTCGAGGGTCATCCATTACGAAAAGACCTTC
RRII105_nad9                      TATATCAACAGATTATGGTTTCGAGGGTCATCCATTACGAAAAGACCTTC
RRIM600_nad9                      TATATCAACAGATTATGGTTTCGAGGGTCATCCATTACGAAAAGACCTTC
Megaceros aenigmaticus            TATATCAATAGATTATGGTTCTGAGGGTCATCTGTTACGAAAAGACCTTC
Phaeoceros laevis                 TATATCAATAGATTATGGTTTTGAGGGTCATCCGTTACGAAAAGACCTTT
Huperzia squarrosa                TATATTAACAGATTATGGTTTTGAGGGTCATCCATTACGAAAAGACTTTC
                                  ***** **  **********  ***** ****  ************ **

Arabidopsis thaliana              CTCTGAGTGGATATGTCCAAGTACGCTATGATGATCCAGAGAAACGTGTG
Brassica napus                    CTCTGAGTGGATATGTGGAAGTACGCTATGATGATCCAGAGAAACGTGTG
Beta macrocarpa                   CTCTGAGTGGATATGTAGAAGTACGCTATGATGATCCAGAGAAACGTGTG
Beta vulgaris maritima            CTCTGAGTGGATATGTAGAAGTACGCTATGATGATCCAGAGAAACGTGTG
Beta vulgaris vulgaris            CTCTGAGTGGATATGTAGAAGTACGCTATGATGATCCAGAGAAACGTGTG
```

```
Silene latifolia                  CTCTGAGTGGATATGTAGAAGTACGCTATGATGATCCAGAGAAACGTGTG
Oryza sativa Japonica             CTCTGAGTGGATATGTGGAAGTACGCTATGATGATCCAGAGAAACGTGTG
PB235_nad9                        CTCTGAGTGGATATGTGGAAGTACGCTATGATGATCCAGAGAAACGTGTA
Citrullus lanatus                 CTCTGAGTGGATATGTGGAAGTACGCTATGATGATCCAGAGAAACGTGTG
Cucurbita pepo                    CTCTGAGTGGATATGTGGAAGTACGCTATGATGATCCAGAGAAACGTGTG
BPM24_nad9                        CTCTGAGTGGATATGTGGAAGTACGCTATGATGATCCAGAGAAACGTGTA
Millettia pinnata                 CTCTGAGCGGATATGTAGAAGTACGCTATGATGATCCGGAGAAACGTGTG
Millettia pinnata                 CTCTGAGCGGATATGTAGAAGTACGCTATGATGATCCGGAGAAACGTGTG
Lotus japonicus                   CTCTGAGTGGATATGTAGAAGTACGCTATGATGATCCAGAGAAACGTGTG
RRIC110_nad9                      CTCTGAGTGGATATGTGGAAGTACGCTATGATGATCCAGAGAAACGTGTA
RRIT251_nad9                      CTCTGAGTGGATATGTGGAAGTACGCTATGATGATCCAGAGAAACGTGTA
RRII105_nad9                      CTCTGAGTGGATATGTGGAAGTACGCTATGATGATCCAGAGAAACGTGTA
RRIM600_nad9                      CTCTGAGTGGATATGTGGAAGTACGCTATGATGATCCAGAGAAACGTGTA
Megaceros aenigmaticus            CTTTAAGTGGATACTTCGAGGTACGCTATGATGATTCGGAAAAACGCGTG
Phaeoceros laevis                 CTTTAAGTGGATATTTCGAGGTACGTTATGATGATTCGGAGAAACGTGTG
Huperzia squarrosa                CTTTGAGTGGATATGTGGAAGTACGTTATGATGATTCAGAGAAACGTGTG
                                  ** * ** *****  *  * ***** ********* * ** ***** **

Arabidopsis thaliana              GTTTCTGAACCCATTGAGATGACCCAAGAATTTCGCTATTTCGATTTTGC
Brassica napus                    GTTTCTGAACCCATTGAGATGACCCAAGAATTTCGCTATTTCGATTTTGC
Beta macrocarpa                   GTTTCTGAGCCCATTGAGATGACCCAAGAATTTCGCTATTTCGATTTTGC
Beta vulgaris maritima            GTTTCTGAGCCCATTGAGATGACCCAAGAATTTCGCTATTTCGATTTTGC
Beta vulgaris vulgaris            GTTTCTGAGCCCATTGAGATGACCCAAGAATTTCGCTATTTCGATTTTGC
Silene latifolia                  GTTTCTGAGCCCATTGAGATGACCCAAGAATTTCGCTATTTCGATTTTGC
Oryza sativa Japonica             GTTTCTGAACCCATTGAGATGACCCAAGAATTTCGCTATTTCGATTTTGC
PB235_nad9                        GTTTCTGAACCCATTGAGATGACCCAAGAATTTCGCTATTTCGATTTTGC
Citrullus lanatus                 GTTTCTGAACCCATTGAGATGACCCAAGAATTTCGCTATTTCGATTTTGC
Cucurbita pepo                    GTTTCTGAACCCATTGAGATGACCCAAGAATTTCGCTATTTCGATTTTGC
BPM24_nad9                        GTTTCTGAACCCATTGAGATGACCCAAGAATTTCGCTATTTCGATTTTGC
Millettia pinnata                 GTTTCTGAACCCATTGAGATGACCCAAGAATTTCGCTATTTCGATTTTGC
Millettia pinnata                 GTTTCTGAACCCATTGAGATGACCCAAGAATTTCGCTATTTCGATTTTGC
Lotus japonicus                   GTTTCTGAACCCATTGAGATGACCCAAGAATTTCGCTATTTCGATTTTGC
RRIC110_nad9                      GTTTCTGAACCCATTGAGATGACCCAAGAATTTCGCTATTTCGATTTTGC
RRIT251_nad9                      GTTTCTGAACCCATTGAGATGACCCAAGAATTTCGCTATTTCGATTTTGC
RRII105_nad9                      GTTTCTGAACCCATTGAGATGACCCAAGAATTTCGCTATTTCGATTTTGC
RRIM600_nad9                      GTTTCTGAACCCATTGAGATGACCCAAGAATTTCGCTATTTCGATTTTGC
Megaceros aenigmaticus            GTTTCTGAACCTATTGAGATGACTCAAGAATCTCGCTATTTTGATTTTGC
Phaeoceros laevis                 GTTTCTGAACCTATTGAGATGACTCAAGAATCTTGCTATTTTGATTTTGC
Huperzia squarrosa                GTTTCTGAACCAATTGAGATGACTCAAGAATTTCGCTATTTTGATTTCGC
                                  ******** ** *********** ******* * ******* ***** **

Arabidopsis thaliana              TAGTCCTTGGGAACAGCG------TAGCGACGGATAATTCAGAATCAGAA
Brassica napus                    TAGTCCTTGGGAACAGCG------TAGCGACGGATAATTCAGAATCAGAA
Beta macrocarpa                   TAGTCCTTGGGAACAACGTAACGGTAACGAAGGATAATTCGGAATCAGAA
Beta vulgaris maritima            TAGTCCTTGGGAACAACGTAACGGTAACGAAGGATAATTCGGAATCAGAA
Beta vulgaris vulgaris            TAGTCCTTGGGAACAACGTAACGGTAACGAAGGATAATTCGGAATCAGAA
Silene latifolia                  TAGTCCTTGGGAACAACGTAACGGTAACGACGGATAATTTGGAATCATAA
Oryza sativa Japonica             TAGTCCTTGGGAACAGCG------TAGCGACGGATAATTCCGAATCTACA
PB235_nad9                        TAGTCCTTGGGAACAGCG------TAGCGACAGATAA-------------
Citrullus lanatus                 TAGTCCTTGGGAACAGCG------TAGCGACAGATAATTAAGAAGAAGAA
Cucurbita pepo                    TAGTCCTTGGGAACAGCG------TAGCGACAGATAATTCAGAATCAGAA
BPM24_nad9                        TAGTCCTTGGGAACAGCG------TAGCGACAGATAA-------------
Millettia pinnata                 TAGTCCTTGGGAACAGCG------TAGCGACGGATAATTCAGAATCAGAA
Millettia pinnata                 TAGTCCTTGGGAACAGCG------TAGCGACGGATAATTCAGAATCAGAA
Lotus japonicus                   TAGTCCTTGGGAACAGCG------TAGCGACGGATAATTCAGAATCTGAA
RRIC110_nad9                      TAGTCCTTGGGAACAGCG------TAGCGACAGATAA-------------
RRIT251_nad9                      TAGTCCTTGGGAACAGCG------TAGCGACAGATAA-------------
RRII105_nad9                      TAGTCCTTGGGAACAGCG------TAGCGACAGATAA-------------
RRIM600_nad9                      TAGTCCTTGGGAACAGCG------TAGCGACAGATAA-------------
Megaceros aenigmaticus            CAGTCCTTGGGAA-----GTCGCGTCGTTACGAATCA--AATGA------
Phaeoceros laevis                 CAGTCCTTGGGAA-----ATCGCGTCGTTACGAATCA--AATGA------
Huperzia squarrosa                TAGTCCTTGGGAAUAAATGTTGCTATGTGACGAATCA--AAAAACAAGTA
```

```
                                   ************                *   ** *

Arabidopsis thaliana              TAGGTCCACC-----AGTCCAGGGGACAAATCAATAGGAAATGCTATAGG
Brassica napus                    TAGGTCCACC-----AGTCCAGGGGACAAATCAATAGGAAATGCTATTTG
Beta macrocarpa                   TAAGTCCA--------GTCCAGGGGACAAATCAATAGGAAATGCTATTTG
Beta vulgaris maritima            TAAGTCCA--------GTCCAGGGGACAAATCAATAGGAAATGCTATTTG
Beta vulgaris vulgaris            TAAGTCCA--------GTCCAGGGGACAAATCAATAGGAAATGCTATTTG
Silene latifolia                  TAAGTCCA--------GTCCAGGGGACAAAGAAATAGGAAATGCTATTTG
Oryza sativa Japonica             TAACTAGTCT-----AGTCCAGGAGACAAATCAATAGGAAATGCTATTTG
PB235_nad9                        --------------------------------------------------
Citrullus lanatus                 TAGGTCCAGTCC----AG----GGGACAAATCAATAGGAAATGCTATTTG
Cucurbita pepo                    TAGGTCCAGTCC----AGCCAGGGGACAAATCAATAGGAAATGCTATTTG
BPM24_nad9                        --------------------------------------------------
Millettia pinnata                 TAGGTCC---------GGATAGGGGACAAATCAATAGGAAATGCTATTTG
Millettia pinnata                 TAGGTCC---------GGATAGGGGACAAATCAATAGGAAATGCTATTTG
Lotus japonicus                   TAGGTCCAGCTCTATAGGATAGGGGACAAATCAATAGGAAATGCTATTTG
RRIC110_nad9                      --------------------------------------------------
RRIT251_nad9                      --------------------------------------------------
RRII105_nad9                      --------------------------------------------------
RRIM600_nad9                      --------------------------------------------------
Megaceros aenigmaticus            --------------------------------------------------
Phaeoceros laevis                 --------------------------------------------------
Huperzia squarrosa                TAAATAAAATTAT---AGAGGAAGGATTACTACATTGGATAAACAATCCC


Arabidopsis thaliana              AA---ATGCTATTTGC----TTTGTAAGAAGACTTC----
Brassica napus                    CT---TTGTAAGAAGA----CTTCTATGAAAGTCTT----
Beta macrocarpa                   CT---TCGTAAGAAGA----CTTCTATGAAATGAAAGA--
Beta vulgaris maritima            CT---TCGTAAGAAGA----CTTCTATGAAATGAAAGA--
Beta vulgaris vulgaris            CT---TCGTAAGAAGA----CTTCTATGAAATGAAAGA--
Silene latifolia                  CT---TCGTAAGAAGA----CTTCTATGAAATGAAAAAGT
Oryza sativa Japonica             CT---TCTTAAGAAGAAGAACTTTTTTGAA----------
PB235_nad9                        ----------------------------------------
Citrullus lanatus                 CT---TTGTCAGAATA----AAAAGAAGAAACTTGTA---
Cucurbita pepo                    CTGCTTTGTCAGAATA----AAAAGAAGAA----------
BPM24_nad9                        ----------------------------------------
Millettia pinnata                 CT---TTGTAAGAAGA----AGAATAAACTTCTATGAAAT
Millettia pinnata                 CT---TTGTAAGAAGA----AGAATAAACTTCTATGAAAT
Lotus japonicus                   CT---TTGTAAGAAGA----AGAATAAACTT---------
RRIC110_nad9                      ----------------------------------------
RRIT251_nad9                      ----------------------------------------
RRII105_nad9                      ----------------------------------------
RRIM600_nad9                      ----------------------------------------
Megaceros aenigmaticus            ----------------------------------------
Phaeoceros laevis                 ----------------------------------------
Huperzia squarrosa                AGA-------------------------------------
```

###################

rpl16 multiple sequence alignment

```
Arabidopsis thaliana             --------------------------TATATGTTTTTCUGGTCGATTAAA
BPM24_rpl16                      --------------------------------------------------
RRII105_rpl16                    --------------------------------------------------
RRIC110_rpl16                    --------------------------------------------------
PB235_rpl16                      --------------------------------------------------
RRIT251_rpl16                    --------------------------------------------------
RRIM600_rpl16                    --------------------------------------------------
Oryza sativa Japonica            ATGCCAAAAGGGGTGGAGGGGATACGTATTTGTTGTTCTGGTCGATTAGG
Lotus japonicus                  --GAAAAAAGGGGTTGAGGGGATCCGTATATGTTGTTCAGGTAGATUAGA
Millettia pinnata                ATGAAAAAAGGGGTTGAGGGGATCCGTATATGTTGTTCAGGTCGATUAAA
Citrullus lanatus                --------------------------------------------------
Cucurbita pepo                   --------------------------------------------------
```

```
Arabidopsis thaliana            AGACGCAGCAGAAAAAGCTCAAACTAAATGCTATAAGCATAGAAAAACTT
BPM24_rpl16                     --------------------------------------------------
RRII105_rpl16                   --------------------------------------------------
RRIC110_rpl16                   --------------------------------------------------
PB235_rpl16                     --------------------------------------------------
RRIT251_rpl16                   --------------------------------------------------
RRIM600_rpl16                   --------------------------------------------------
Oryza sativa Japonica           GGGTGCGG---AAATAGCTAGAACTGAATGCGGAAAGTATGGAAAAACAT
Lotus japonicus                 AGGCGCAG---AAATAGCTAGAACTGAATGCGGAAAGTATGGAAAAACAT
Millettia pinnata               AGGCGCAG---AAATAGCTAGAACTGAATGCGGAAAGTATGGAAAAACAT
Citrullus lanatus               --------------------------------------------------
Cucurbita pepo                  --------------------------------------------------

Arabidopsis thaliana            CTUGTAATGTATTTAACCATAAAATCGATTATGCTCCTGUGGAAGTATUT
BPM24_rpl16                     --------------------------------------------------
RRII105_rpl16                   --------------------------------------------------
RRIC110_rpl16                   --------------------------------------------------
PB235_rpl16                     --------------------------------------------------
RRIT251_rpl16                   --------------------------------------------------
RRIM600_rpl16                   --------------------------------------------------
Oryza sativa Japonica           CTTGTAATGTATTTAACCAGAAAATCGATTATGCTUTTGCGGAAGTATCC
Lotus japonicus                 CTUGTAATGTATTTAACCAGAAAATCGATTATGCTTCTGCGGAAGTATCT
Millettia pinnata               CTUGTAATGTATTTAACCAGAAAATCGATTATGCTTCTGCGGAAGTATCT
Citrullus lanatus               ------------------------------------------------CT
Cucurbita pepo                  ---------------------------------------------TATCT

Arabidopsis thaliana            ACTCGTTACGGAATCTUAGGTGTCAAAGTGTGGATTTCATATAGTCAAAA
BPM24_rpl16                     --------------------------------------------------
RRII105_rpl16                   --------------------------------------------------
RRIC110_rpl16                   --------------------------------------------------
PB235_rpl16                     --------------------------------------------------
RRIT251_rpl16                   --------------------------------------------------
RRIM600_rpl16                   --------------------------------------------------
Oryza sativa Japonica           ACTCGTAACGGAATTTUAGGTGTCAAAGTGUGGATCTCATATAGTCAAAA
Lotus japonicus                 ACTCGTTACGGAATCTUAGGTGTCAAAGTGTGGATTTCATATAGTCAAAA
Millettia pinnata               ACTCGTTACGGAATCTUAGGTGTCAAAGTGTGGATTTCATATAGTAAAAA
Citrullus lanatus               ACTCGTTATGGAATCTCAGGTGTCAAAGTGTGGATTTCATATAAAAAAAA
Cucurbita pepo                  ACTCGTTATGGAATCTTAGGTGTCAAAGTGTGGATTTCATATAAAAAACA

Arabidopsis thaliana            AAAAGGGAGACGTGCTATATCCGAAACGTACGAAATATAGTAAATATCGT
BPM24_rpl16                     -----------ATGCTATATCCGAAACGTACGAAATATAGTAAATATCGT
RRII105_rpl16                   -----------ATGCTATATCCGAAACGTACGAAATATAGTAAATATCGT
RRIC110_rpl16                   -----------ATGCTATATCCGAAACGTACGAAATATAGTAAATATCGT
PB235_rpl16                     -----------ATGCTATATCCGAAACGTACGAAATATAGTAAATATCGT
RRIT251_rpl16                   -----------ATGCTATATCCGAAACGTACGAAATATAGTAAATATCGT
RRIM600_rpl16                   -----------ATGCTATATCCGAAACGTACGAAATATAGTAAATATCGT
Oryza sativa Japonica           TAAGAAGGGACGTGCTATATCCGAAACGTACGAAATATAGTAAATATAGT
Lotus japonicus                 AAAAAGGGGACGTGCTATATCCGAAACGTACGAAATTTAGTAAATATCGT
Millettia pinnata               AAAAAAGGGACGTGCTATATCCGAAACGTACGAAATTTAGTAAATATCGT
Citrullus lanatus               AAAAAAGGAACGTGTTATATCCGAAACGTACGAAATATAGTAAATATCGT
Cucurbita pepo                  AAAAAAGGAACGTGTTATATCCGAAACGTACGAAATATAGTAAATCTCGT
                                            ** ********************* ******** * **

Arabidopsis thaliana            AAAGGCAGATGTAGTAGGGGTTGCAAACCGGATGGTACAAAACTGGGTTT
BPM24_rpl16                     AAAGGCAGATGTAGTAGGGGTTGCAAACTGGACGGTACACAACTTGGTTT
RRII105_rpl16                   AAAGGCAGATGTAGTAGGGGTTGCAAACTGGACGGTACACAACTTGGTTT
RRIC110_rpl16                   AAAGGCAGATGTAGTAGGGGTTGCAAACTGGACGGTACACAACTTGGTTT
```

```
PB235_rpl16                     AAAGGCAGATGTAGTAGGGGTTGCAAACTGGACGGTACACAACTTGGTTT
RRIT251_rpl16                   AAAGGCAGATGTAGTAGGGGTTGCAAACTGGACGGTACACAACTTGGTTT
RRIM600_rpl16                   AAAGGCAGATGTAGTAGGGGTTGCAAACTGGACGGTACACAACTTGGTTT
Oryza sativa Japonica           AAATGCAGATGTAGTAGGGGTTGCGAACCGGATGGTACACAACTTGGTTT
Lotus japonicus                 AAAGGCAGATGTAGTAGGGGTTGCAAACCAGACGGAACAAAACTTGGTTT
Millettia pinnata               AAAGGCAGATGTAGTAGGGGTTGCAAACCAGACGGAACAAAACTTGGTTT
Citrullus lanatus               AAAGGCAGATGTAGTAGGGGTTGCAAACCGGACGGTACACAACTTGGTTT
Cucurbita pepo                  AAAGGCAGATGTAGTAGGGGTTGCAAACCAGACGGTACACAACTTGGTTT
                                *** ******************** ***  ** ** *** **** *****

Arabidopsis thaliana            TGGAAGATATGGCAUTAAAAGTTGTAAAGCTGGTUGTCTTTCATATCGAG
BPM24_rpl16                     TGGAAGATATGGCAUTAAAAGTTGTAAAGCTGGTCGTCTTTCATATCGAG
RRII105_rpl16                   TGGAAGATATGGCAUTAAAAGTTGTAAAGCTGGTCGTCTTTCATATCGAG
RRIC110_rpl16                   TGGAAGATATGGCAUTAAAAGTTGTAAAGCTGGTCGTCTTTCATATCGAG
PB235_rpl16                     TGGAAGATATGGCAUTAAAAGTTGTAAAGCTGGTCGTCTTTCATATCGAG
RRIT251_rpl16                   TGGAAGATATGGCAUTAAAAGTTGTAAAGCTGGTCGTCTTTCATATCGAG
RRIM600_rpl16                   TGGAAGATATGGCAUTAAAAGTTGTAAAGCTGGTCGTCTTTCATATCGAG
Oryza sativa Japonica           TGGAAGATATGGUAUUAAAAGTTGTAGAGCTGGTCGTCTTTCATATCGAG
Lotus japonicus                 TGGAAGATATGGCAUTCAAAGTTGTAGAGCTGGTCGTCTTTCATATCGAG
Millettia pinnata               TGGAAGATATGGCAUTCAAAGTTGTAGAGCTGGTCGTCTTTCATATCGAG
Citrullus lanatus               TGGAAGATATGGCAUTCAAAGTTGTAGAGCTGGTCGTCTTTCATATCGAG
Cucurbita pepo                  TGGAAGATATGGCAUTCAAAGTTGTAGAGCTGGTCGTCTTTCATATCGAG
                                ************ **  ********* ******* ***************

Arabidopsis thaliana            CCATTGAAGCAGCGCGTCGGGCTATAATCGGACACTTTCATCGTGCTATG
BPM24_rpl16                     CCATTGAAGCAGCGCGTCGGGCTATAATAGGACAATTCCATCGTGCTATG
RRII105_rpl16                   CCATTGAAGCAGCGCGTCGGGCTATAATAGGACAATTCCATCGTGCTATG
RRIC110_rpl16                   CCATTGAAGCAGCGCGTCGGGCTATAATAGGACAATTCCATCGTGCTATG
PB235_rpl16                     CCATTGAAGCAGCGCGTCGGGCTATAATAGGACAATTCCATCGTGCTATG
RRIT251_rpl16                   CCATTGAAGCAGCGCGTCGGGCTATAATAGGACAATTCCATCGTGCTATG
RRIM600_rpl16                   CCATTGAAGCAGCGCGTCGGGCTATAATAGGACAATTCCATCGTGCTATG
Oryza sativa Japonica           CCATTGAAGCAGCGCGTCGGGCTAUAATCGGGCAATTCCATCGTGCTATG
Lotus japonicus                 CCATTGAAGCAGCGCGTCGGGCTATAATCGGACACTTCCATCGTGCTATG
Millettia pinnata               CCATTGAAGCAGCGCGTCGGGCTATAATCGGACACTTCCATCGTGCTATG
Citrullus lanatus               CCATTGAAGCAGCGCGTCGGGCTATAATCGGACAATTCC-----------
Cucurbita pepo                  CCATTGAAGCAGCGCGTAGGGCTATAATCGGACAATTCCATCGTGCTATG
                                ***************** ****** *** ** ** ** *

Arabidopsis thaliana            AGCGGACAATTCCGAAGAAATGGTAAGATATGGGTAAGAGTTTTCGCGGA
BPM24_rpl16                     AGCGGACAATTCCGAAGAAATGGTAAGATATGGGTAAGAGTTUTCGCAGA
RRII105_rpl16                   AGCGGACAATTCCGAAGAAATGGTAAGATATGGGTAAGAGTTUTCGCAGA
RRIC110_rpl16                   AGCGGACAATTCCGAAGAAATGGTAAGATATGGGTAAGAGTTUTCGCAGA
PB235_rpl16                     AGCGGACAATTCCGAAGAAATGGTAAGATATGGGTAAGAGTTUTCGCAGA
RRIT251_rpl16                   AGCGGACAATTCCGAAGAAATGGTAAGATATGGGTAAGAGTTUTCGCAGA
RRIM600_rpl16                   AGCGGACAATTCCGAAGAAATGGTAAGATATGGGTAAGAGTTUTCGCAGA
Oryza sativa Japonica           AGCGGACAATTCCGAAGAAATTGTAAGATATGGGTAAGAGTTUTCGCAGA
Lotus japonicus                 AGCGGACAATTCCGAAAAAATGGTAAGATATGGGTCAGAGTTTTCGCAGA
Millettia pinnata               AGCGGACAATTCCGAAAAAATGGTAAGATATGGGTAAGAGTTTTCGCAGA
Citrullus lanatus               -------------GAAGAAATGGTAAAATATGGGTAAGAGTTUTCGCAGA
Cucurbita pepo                  AGCGGACAATCCCGAAGAAATGGTCAAATATGGGTCAGAGTTUTAGCAGA
                                             *** **** ** * ******** ****** * ** **

Arabidopsis thaliana            TCTCCCTATTACCGGGAAACCCACAGAAGTAAGAATGGGAAGAGGAAAAG
BPM24_rpl16                     TCTCCCTATTACCGGGAAACCTACAGAAGTAAGAATGGGAAGAGGAAAAG
RRII105_rpl16                   TCTCCCTATTACCGGGAAACCTACAGAAGTAAGAATGGGAAGAGGAAAAG
RRIC110_rpl16                   TCTCCCTATTACCGGGAAACCTACAGAAGTAAGAATGGGAAGAGGAAAAG
PB235_rpl16                     TCTCCCTATTACCGGGAAACCTACAGAAGTAAGAATGGGAAGAGGAAAAG
RRIT251_rpl16                   TCTCCCTATTACCGGGAAACCTACAGAAGTAAGAATGGGAAGAGGAAAAG
RRIM600_rpl16                   TCTCCCTATTACCGGGAAACCTACAGAAGTAAGAATGGGAAGAGGAAAAG
Oryza sativa Japonica           TCTTCCTATTACGGGGAAACCCGCAGAAGTTCGAATGGGAAGAGGAAAAG
Lotus japonicus                 TATCCCTATTACCGGGAAACCTACAGAAGTAAGAATGGGAAGAGGAAAAG
Millettia pinnata               TATCCCTATTACCGGGAAACCTACAGAAGTAAGAATGGGAAGAGGAAAAG
```

```
Citrullus lanatus           TCTCCCTATTACCGGGAAACCTACAGAAGTCAGAATGGGAAGAGGAAAAG
Cucurbita pepo              TCTCCCCATTACCGGGAAACCTACAGAAGTCAGAATGGGAAGAGGAAAAG
                            * * ** ***** ********  *******  ******************

Arabidopsis thaliana        GAAATCCTACGGGTTGGATTGCTCGTGTGTCCACGGGACAAATCCUATTT
BPM24_rpl16                 GAAATCCTACGGGTTGGATTGCTCGTGTGTCTACGGGACAAATCCUATTT
RRII105_rpl16               GAAATCCTACGGGTTGGATTGCTCGTGTGTCTACGGGACAAATCCUATTT
RRIC110_rpl16               GAAATCCTACGGGTTGGATTGCTCGTGTGTCTACGGGACAAATCCUATTT
PB235_rpl16                 GAAATCCTACGGGTTGGATTGCTCGTGTGTCTACGGGACAAATCCUATTT
RRIT251_rpl16               GAAATCCTACGGGTTGGATTGCTCGTGTGTCTACGGGACAAATCCUATTT
RRIM600_rpl16               GAAATCCTACGGGTTGGATTGCTCGTGTGTCTACGGGACAAATCCUATTT
Oryza sativa Japonica       GAAATCCTACGGGTTGGATTGCTCGTGTGTCUACGGGACAAATCCUATTT
Lotus japonicus             GAAATCCTACGGGTTGGATTGCTCGTGTGTCCACGGGACAAGTCCTATTT
Millettia pinnata           GAAATCCTACGGGTTGGATTGCTCGTGTGTCCACGGGACAAGTCCTATTT
Citrullus lanatus           GAAATCCTACGGGTTGGATTGCTCGTGTGTCCACGGGACAAATCCUATTT
Cucurbita pepo              GAAATCCTACGGGTTGGGTTGCTCGTGTGTCCACGGGACAAATCCUATTT
                            ***************** ************* ********* *** ****

Arabidopsis thaliana        GAAATGGATGGTGTGAGTTTGGCAAATGCTCGACAAGCCGCTACATTAGC
BPM24_rpl16                 GAAATGGATGGTGTGAGTTTGTCAAATGCTCGACAAGCCGCTACATTAGC
RRII105_rpl16               GAAATGGATGGTGTGAGTTTGTCAAATGCTCGACAAGCCGCTACATTAGC
RRIC110_rpl16               GAAATGGATGGTGTGAGTTTGTCAAATGCTCGACAAGCCGCTACATTAGC
PB235_rpl16                 GAAATGGATGGTGTGAGTTTGTCAAATGCTCGACAAGCCGCTACATTAGC
RRIT251_rpl16               GAAATGGATGGTGTGAGTTTGTCAAATGCTCGACAAGCCGCTACATTAGC
RRIM600_rpl16               GAAATGGATGGTGTGAGTTTGTCAAATGCTCGACAAGCCGCTACATTAGC
Oryza sativa Japonica       GAAATGGATGGTGTGAGTTTGTCAAATGCTCGACAAGCCGCTAGATTAGC
Lotus japonicus             GAAATGGATGGTGTGAGTTTGTCAAATGCTCGACAAGCCGCTACATTAGC
Millettia pinnata           GAAATGGATGGTGTGAGTTTGTCAAATGCTCGACAAGCCGCTACATTAGC
Citrullus lanatus           GAAATGGATGGTGTGAGTTTGTCAAATGCTCGACAAGCCGCTACATTAGC
Cucurbita pepo              GAAATAGATGGTGTGAGTTTGTCAAATGCTCGACAAGCCACTACATTAGC
                            ***** *************** ***************** *** ******

Arabidopsis thaliana        GGCTCATAAACUATGTTUGTCAACCAAGTTTGTTCAGTGGTCGTAAUGTA
BPM24_rpl16                 GGCGCATAAACUATGTTUGTCAACCAAGTTTGTTCAATGGTCGTAA----
RRII105_rpl16               GGCGCATAAACUATGTTUGTCAACCAAGTTTGTTCAATGGTCGTAA----
RRIC110_rpl16               GGCGCATAAACUATGTTUGTCAACCAAGTTTGTTCAATGGTCGTAA----
PB235_rpl16                 GGCGCATAAACUATGTTUGTCAACCAAGTTTGTTCAATGGTCGTAA----
RRIT251_rpl16               GGCGCATAAACUATGTTUGTCAACCAAGTTTGTTCAATGGTCGTAA----
RRIM600_rpl16               GGCGCATAAACUATGTTCGTCAACCAAGTTTGTTCAATGGTCGTAA----
Oryza sativa Japonica       GGCGCATAAACUATGTTUGTCAACCAAGTTTGTTCAGTGGTCGTAACGTA
Lotus japonicus             GGCGCATAAACUATGTTUGTCAACCAAGTTTGTTCAGTGGTCGTAACGTA
Millettia pinnata           GGCGCATAAACUATGTTUGTCAACCAAGTTTGTTCAGTGGTCGTAACGTA
Citrullus lanatus           GGCGCATAAACCATGTTCGTCAACCAAGTTTGTTCAGTGGTCGTAACGTA
Cucurbita pepo              GGCGCATAAACUATGTTUGTCAACCAAGTTTGTTCAGTGGTCGTAACCTC
                            *** ******* ***** ****************** *********

Arabidopsis thaliana        ATT----GGTTAATGGGGATAAAGCGGGCCGAGAATCTTATGTCAAAAGT
BPM24_rpl16                 --------------------------------------------------
RRII105_rpl16               --------------------------------------------------
RRIC110_rpl16               --------------------------------------------------
PB235_rpl16                 --------------------------------------------------
RRIT251_rpl16               --------------------------------------------------
RRIM600_rpl16               --------------------------------------------------
Oryza sativa Japonica       ATT----GGTTAGTGGGGAAAAACCGGGCCGGGACTCA-----AAAGAAT
Lotus japonicus             ATT----GGTTAGTGGGGAAAAACCAGGCCGGGATTCA-----AAATAAT
Millettia pinnata           ATT----GGTTAGTGGGGAAAAACCAGGCCGGGATTCA-----AAAGATT
Citrullus lanatus           ATTAATTGGTTAGTGGGGATAAACCGGGCCGGGATTCA-----AAAGAAT
Cucurbita pepo              ATTTATTGGTTAGTGGGGATAAACCGGGCCGGGATTCA-----AAAGAAT


Arabidopsis thaliana        AAGGACCAAGGATAATCTTTTUGGAAAGGAGG--------
BPM24_rpl16                 ----------------------------------------
```

```
RRII105_rpl16                   ----------------------------------------
RRIC110_rpl16                   ----------------------------------------
PB235_rpl16                     ----------------------------------------
RRIT251_rpl16                   ----------------------------------------
RRIM600_rpl16                   ----------------------------------------
Oryza sativa Japonica           TTGGCGAAGTGTTTGTTCCTGAACGAGGGAAGTGGAAA--
Lotus japonicus                 TAGGCGAAGGGTTTGTTCCTGAACGAGGGAAGTGGAAAGA
Millettia pinnata               TAGGCGAAGGGTTTGTTCCTGAACGAGGGAAGTGGAAA--
Citrullus lanatus               TGGGCGAAGTGT----------------------------
Cucurbita pepo                  TGGGCGAAGTGTTTG-------------------------
```

##################

rpl5 multiple sequence alignment

```
Citrullus lanatus                -------TTTCCGTTTCATTCAACCAACTATCTTTTTGAAGCAGATAGTT
Cucurbita pepo                   -------AATCAGTCTCATTCAACCAACTATCTTTTTGAAGCAGATAGTT
Arabidopsis thaliana             ATGTCAAAAGGACCAAGGATGATCTTTTCGGAA---AGGAGGAGTAGGAG
Brassica napus                   ATGTCAAAAGGACCAAGGATGATCTTTTCGGAA---AGGAGGAGTAGGAG
RRII105_rpl5                     --------------------------------------------------
Lotus japonicus                  ------ATCCCTCTATAGTTCTTCACTACTCCGTGCAGGAAAGACTCTCT
Millettia pinnata                ----ACATCTAACTTTCGTTCTTCACTACTCCGTGCAGGAAAGACTCTCT
RRIM600_rpl5                     --------------------------------------------------
RRIC110_rpl5                     --------------------------------------------------
Beta vulgaris maritima           -----GAAAAAACCAACTATAAATATCCTACAAGTCTTTTTTCATTTCTC
Beta vulgaris vulgaris           -----GAAAAAACCAACTATAAATATCCTACAAGTCTTTTTTCATTTCTC
Beta macrocarpa                  -----GAAAAAACCAACTATAAATATCCTACAAGTCTTTTTTCATTTCTC
Silene latifolia                 ------TAGAGACCTCCCTTGATCTGTGCGGGATCTATCAGCAGCGGCAT
RRIT251_rpl5                     --------------------------------------------------
PB235_rpl5                       --------------------------------------------------
BPM24_rpl5                       --------------------------------------------------
Oryza sativa Japonica            ----ACGTTGGTTTCGGTTCGATCAACTATCCTTTTTGAAGCGGATAGTT


Citrullus lanatus                CACAGTGCTCATTCTATAGATACTGTGTAAAAAAGCCAACTC-ATGTTTT
Cucurbita pepo                   CACAGTGCTCATTCTATAGATACTGTGTAAAAAAGCCAACTC-ATGTTTT
Arabidopsis thaliana             GAGTCAGCTTATTCTATAGATACTG----TAAAAGCCAACTC-ATGTTTC
Brassica napus                   GAGTCAGCTTATTCTATAGATACTG----TAAAAGCCAACTC-ATGTTTC
RRII105_rpl5                     -------------------------------------------ATGTTTC
Lotus japonicus                  TTCTGTCTGTATGGAGGGAATTTTT----CTCAATCAATCACTATGTTTC
Millettia pinnata                TTCTGTATGGAGGGAATTTTGATTT----ATCAATCAATCACTATGTTTC
RRIM600_rpl5                     -------------------------------------------ATGTTTC
RRIC110_rpl5                     -------------------------------------------ATGTTTC
Beta vulgaris maritima           GAGAGAGCAGTCAAAGAATGAATAA----GAAAAGCCAACTC-ATGTTTC
Beta vulgaris vulgaris           GAGAGAGCAGTCAAAGAATGAATAA----GAAAAGCCAACTC-ATGTTTC
Beta macrocarpa                  GAGAGAGCAGTCAAAGAATGAATAA----GAAAAGCCAACTC-ATGTTTC
Silene latifolia                 TCTCCTCTATACTCTTTTTGGAAAC----GAAAAGCCAACTC-ATGTTTC
RRIT251_rpl5                     -------------------------------------------ATGTTTC
PB235_rpl5                       -------------------------------------------ATGTTTC
BPM24_rpl5                       -------------------------------------------ATGTTTC
Oryza sativa Japonica            CACAGTGCTCATTCTCAAAAAAAAGCG--GAAAAGCCA---TGATGTTTC
                                                                            ******

Citrullus lanatus                CACTCGATTTTCATTACGAAGATGTATUACGTCAGGATCUGTTGCTCAAA
Cucurbita pepo                   CACTCGATTTTCATTACGAAGATGTATUACGTCAGGATCUGTTGCTCAAA
Arabidopsis thaliana             CACTCAATTTTCATTACGAAGATGTATUACGTCAAGATCUGTTGCTCAAA
Brassica napus                   CACTCAATTTTCATTACGAAGATGTATUACGTCAAGATCUGTTGCTCAAA
RRII105_rpl5                     CACTCTATTTTCATTACGAAGATGTATCACGTCAGGATCCGTTGCTCAAA
Lotus japonicus                  CACTCAATTTTCATTACGAAGATGTATUACGTCAGGATCUGTTGCTCAAA
Millettia pinnata                CACTCCATTTTCATTACGAAGATGTATUACGTCAGGATCUGTTGCTCAAA
RRIM600_rpl5                     CACTCTATTTTCATTACGAAGATGTATCACGTCAGGATCCGTTGCTCAAA
RRIC110_rpl5                     CACTCTATTTTCATTACGAAGATGTATCACGTCAGGATCCGTTGCTCAAA
Beta vulgaris maritima           CCCTCCATTTTCATTACGAAGATGTTTCACGTCAAGATCUGTTGCTCAAA
```

```
Beta vulgaris vulgaris          CCCTCCATTTTCATTACGAAGATGTTTCACGTCAAGATCUGTTGCTCAAA
Beta macrocarpa                 CCCTCCATTTTCATTACGAAGATGTTTCACGTCAAGATCUGTTGCTCAAA
Silene latifolia                CACTCCATTTTCATTACGAAGATGTTUACGTCAAGATCUGTTGCTCAAA
RRIT251_rpl5                    CACTCTATTTTCATTACGAAGATGTATCACGTCAGGATCCGTTGCTCAAA
PB235_rpl5                      CACTCTATTTTCATTACGAAGATGTATCACGTCAGGATCCGTTGCTCAAA
BPM24_rpl5                      CACTCTATTTTCATTACGAAGATGTATCACGTCAGGATCCGTTGCTCAAA
Oryza sativa Japonica           CACTCCATTTTCATTACGAAGATGTATTACGTCAGGATCTGTTGCTCAAA
                                * *** ******************* * ****** **** **********

Citrullus lanatus               CTGAATCACGCCAACGTTATGGAAGTTCCTGGATUGTGTGAAATAAGAGT
Cucurbita pepo                  CTGAATCACGCCAACGTTATGGAAGTTCCTGGATUGTGTGAAATAAGAGT
Arabidopsis thaliana            UUGAATUACGCCAACGTTATGGAAGTTCCTGGATUGTGTGAAATAAGAGT
Brassica napus                  CUGAATUACGCCAACGTTATGGAAGTTCCTGGATUGTGTGAAATAAGAGT
RRII105_rpl5                    CCGAATCACGCCAACGTTATGGAAGTTCCTGGATTGTGTGAAATAAGAGT
Lotus japonicus                 CTGAATUACGCCAACGTTATGGAAGTTCCTGGATUGTGTGAAATAAGAGT
Millettia pinnata               CTGAATUACGCCAACGTTATGGAAGTTCCTGGATUGTGTGAAATAAGAGT
RRIM600_rpl5                    CCGAATCACGCCAACGTTATGGAAGTTCCTGGATTGTGTGAAATAAGAGT
RRIC110_rpl5                    CCGAATCACGCCAACGTTATGGAAGTTCCTGGATTGTGTGAAATAAGAGT
Beta vulgaris maritima          CUGAATUACGCCAACGTTATGGACGTTCCTGGATTGTGTGAAATAAGAGT
Beta vulgaris vulgaris          CUGAATUACGCCAACGTTATGGACGTTCCTGGATTGTGTGAAATAAGAGT
Beta macrocarpa                 CUGAATUACGCCAACGTTATGGACGTTCCTGGATTGTGTGAAATAAGAGT
Silene latifolia                CTGAATUACGCCAACGTTATGGAAGTTCCTGGATTGTGTGAAATAAGAGT
RRIT251_rpl5                    CCGAATCACGCCAACGTTATGGAAGTTCCTGGATTGTGTGAAATAAGAGT
PB235_rpl5                      CCGAATCACGCCAACGTTATGGAAGTTCCTGGATTGTGTGAAATAAGAGT
BPM24_rpl5                      CCGAATCACGCCAACGTTATGGAAGTTCCTGGATTGTGTGAAATAAGAGT
Oryza sativa Japonica           CTGAATUACGCCAATGTTATGGAAGTTCCTGGATTGTTTGAAATCAGATT
                                  **** ******* ******** ********** ** ****** *** *

Citrullus lanatus               CGTACCAAAGG---CACCCTATGATTTCATAATCAAAAATGGAAAATTG-
Cucurbita pepo                  CGTACCAAAGG---CACCCTATGATTTCATAATCAAAAATGGAAAATTG-
Arabidopsis thaliana            AGTACCAAAGG---CACCCTATAATTTCATAATAAAAAATGGAAAATTG-
Brassica napus                  AGTACCAAAGG---CACCCTATAATTTCATAATAAAAAATGGAAAATTG-
RRII105_rpl5                    AGTACCAAAGG---CACCTTATGATTTCATAATCAAAAATGGAAAATTG-
Lotus japonicus                 AGTACCAAAGG---CACCCTATGATTTCATAATGAAAAATGGAAAATTG-
Millettia pinnata               AGTACCAAAGA---CACCCTATGATTTCATAATAAAAAATGGAAAATTGT
RRIM600_rpl5                    AGTACCAAAGG---CACCTTATGATTTCATAATCAAAAATGGAAAATTG-
RRIC110_rpl5                    AGTACCAAAGG---CACCTTATGATTTCATAATCAAAAATGGAAAATTG-
Beta vulgaris maritima          AGTACCAAAGGCAGCACCTTCTGATTTCATAATCCAAAATGGAAAATTG-
Beta vulgaris vulgaris          AGTACCAAAGGCAGCACCTTCTGATTTCATAATCCAAAATGGAAAATTG-
Beta macrocarpa                 AGTACCAAAGGCAGCACCTTCTGATTTCATAATCCAAAATGGAAAATTG-
Silene latifolia                AGTACCAAAGGCAGCACCTTCTGATTTCATAATAAAAAATGGAAAATTG-
RRIT251_rpl5                    AGTACCAAAGG---CACCTTATGATTTCATAATCAAAAATGGAAAATTG-
PB235_rpl5                      AGTACCAAAGG---CACCTTATGATTTCATAATCAAAAATGGAAAATTG-
BPM24_rpl5                      AGTACCAAAGG---CACCTTATGATTTCATAATCAAAAATGGAAAATTG-
Oryza sativa Japonica           AGTACCAAAAG---CTGCCTCTGATTTTAAAATCCAATTTGGAAAATTG-
                                 ********     *  * * * **** * ***  **  **********

Citrullus lanatus               --GCTATGGAGATTUCGUGCGGTCAGAAATTTATACAGACACAAAGGGGT
Cucurbita pepo                  --GCTATGGAGATTUCGUGCGGTCAGAAATTGATACAGACACAAAGGGGT
Arabidopsis thaliana            --GCTATGGAGATTCCGUGCGGTCAGAAATTCATACAGACACAAAGGGGT
Brassica napus                  --GCTATGGAGATTCCGUGCGGTCAGAAATTCATACAGACACAAAGGGGT
RRII105_rpl5                    --GCTATGGAGATTUCATGCGGTCAGAAATTCATACAGACACAAAGGGGT
Lotus japonicus                 --GCTATGGAGATTCCGUGCGGTCAGAAATTCATACAGACACAAAGGGGT
Millettia pinnata               TGGCTATGGAGATTCCGUGCGGTCAGAAATTCATACAGACACAAAGGGGT
RRIM600_rpl5                    --GCTATGGAGATTNCATGCGGTCAGAAATTCATACAGACACAAAGGGGT
RRIC110_rpl5                    --GCTATGGAGATTUCATGCGGTCAGAAATTCATACAGACACAAAGGGGT
Beta vulgaris maritima          --GCTATGGAGATTUCGTGCGGTCAGAAATTTATACGGACACGCAGGGGT
Beta vulgaris vulgaris          --GCTATGGAGATTUCGTGCGGTCAGAAATTTATACGGACACGCAGGGGT
Beta macrocarpa                 --GCTATGGAGATTUCGTGCGGTCAGAAATTTATACGGACACGCAGGGGT
Silene latifolia                --GCTATGGAGATTUCGTGCGGTCAGAAATTAATAGAGACACGCAGGGGT
RRIT251_rpl5                    --GCTATGGAGATTUCATGCGGTCAGAAATTCATACAGACACAAAGGGGT
PB235_rpl5                      --GCTATGGAGATTUCATGCGGTCAGAAATTCATACAGACACAAAGGGGT
```

```
BPM24_rpl5                       --GCTATGGAGATTUCATGCGGTCAGAAATTCATACAGACACAAAGGGGT
Oryza sativa Japonica            --GCTATGGAGATTTGTGCGGTCAGAGATTCATACAGACACAAAGGGGC
                                   ************    ********* *** ***  *****  *****

Citrullus lanatus                TC---------GACAGGAAAGTCGTTTCGATCCAATCCATTCTTGGAGTC
Cucurbita pepo                   TC---------GACAGGAAAGTCCTTTCGATCCAATCCATTCTTGGAGTC
Arabidopsis thaliana             TC---------GACAGGAAAGTCCTTTCGATCTAATCCATTCTTGGGGTC
Brassica napus                   TC---------GACAGGAAAGTCCTTTCGATCTAATCCATTCTTGGGGTC
RRII105_rpl5                     TC---------GACAGGAAAGTCGTTTCGATCCAATCCATTCTTGGGGTC
Lotus japonicus                  TC---------GACAGGAAAGTCGTTTCGATCCAATCCATTCTTGGGGTC
Millettia pinnata                TC---------GACAGGAAAGTCGTTTCGATCCAATCCATTCTTGGGGTC
RRIM600_rpl5                     TC---------GACAGGAAAGTCGTTTCGATCCAATCCATTCTTGGGGTC
RRIC110_rpl5                     TC---------GACAGGAAAGTCGTTTCGATCCAATCCATTCTTGGGGTC
Beta vulgaris maritima           TC---------GATAGGAAAGTCGTTTCAATCCAATCCATTCTTGGGGTC
Beta vulgaris vulgaris           TC---------GATAGGAAAGTCGTTTCAATCCAATCCATTCTTGGGGTC
Beta macrocarpa                  TC---------GATAGGAAAGTCGTTTCAATCCAATCCATTCTTGGGGTC
Silene latifolia                 TC---------GATAGTAAAGTCGTTTCGATCCAATCCATTCTTGGGGTC
RRIT251_rpl5                     TC---------GACAGGAAAGTCGTTTCGATCCAATCCATTCTTGGGGTC
PB235_rpl5                       TC---------GACAGGAAAGTCGTTTCGATCCAATCCATTCTTGGGGTC
BPM24_rpl5                       TC---------GACAGGAAAGTCGTTTCGATCCAATCCATTCTTGGGGTC
Oryza sativa Japonica            CCTTATTTTAAAGCAGGAAAGTCGTTTCGATCCAATCCATTCTTGGGGTC
                                  *            ** ****** **** *** ************* ***

Citrullus lanatus                AAATAAAGACAAA---GGATATGTCAGTGACCTAGCACGACAAAGCACTC
Cucurbita pepo                   AAATAAAGACAAA---GGATATGTCAGTGACCTAGCCCGACAAAGCACTC
Arabidopsis thaliana             AAATAAAGACAAA---GGATATGTAAGTGACCTAGCACGACAAAGCACTC
Brassica napus                   AAATAAAGACAAA---GGATATGTAAGTGACCTAGCACGACAAAGCACTC
RRII105_rpl5                     AAATAAAGACAAAAAAGGATATGTUAGTGACCTAGCACGACAAAGCACTC
Lotus japonicus                  AAATAAAGACAAA---GGATATGTCAGTGACCTAGCACGACAAAGCACTC
Millettia pinnata                AAATAAAGACAAA---GGATATGTCAGTGACCTAGCACGACAAAGCACTC
RRIM600_rpl5                     AAATAAAGACAAAAAAGGATATGTNAGTGACCTAGCACGACAAAGCACTC
RRIC110_rpl5                     AAATAAAGACAAAAAAGGATATGTUAGTGACCTAGCACGACAAAGCACTC
Beta vulgaris maritima           AAATAAAGACAAA---GGATATGTCAATGACCTAGCACGACAAAGCACTC
Beta vulgaris vulgaris           AAATAAAGACAAA---GGATATGTCAATGACCTAGCACGACAAAGCACTC
Beta macrocarpa                  AAATAAAGACAAA---GGATATGTCAATGACCTAGCACGACAAAGCACTC
Silene latifolia                 AAATAAAGACAAC---GGATATGTCAATGACCTAGCACGACAAAGCACTC
RRIT251_rpl5                     AAATAAAGACAAAAAAGGATATGTUAGTGACCTAGCACGACAAAGCACTC
PB235_rpl5                       AAATAAAGACAAAAAAGGATATGTUAGTGACCTAGCACGACAAAGCACTC
BPM24_rpl5                       AAATAAAGACAAAAAAGGATATGTUAGTGACCTAGCACGACAAAGCACTC
Oryza sativa Japonica            CGAAAAAGACACT---GGATATGTCAGTGACTTTGCACGACAAAGCGTTC
                                   * *******     ******** * **** * ** *********  **

Citrullus lanatus                TCCGAGGGCATGGAATGTCGAATTTTTUGGTCAGAATCTUGACAGTAATG
Cucurbita pepo                   TCCGAGGGCATGGAATGTCGAATTTTGUGGTCAGAATCTCCACAGTAATG
Arabidopsis thaliana             TCCGAGGGCATGGAATGTCTAATTTTTUGGTCAGAATCTUGACAGTAATG
Brassica napus                   TCCGAGGGCATGGAATGTCTAATTTTTUGGTCAGAATCTUGACAGTAATG
RRII105_rpl5                     TCCGAGGGCATGGAATGTCTAATTTTTCGGTCAGAATCTUGACAGTAATG
Lotus japonicus                  TCCGAGGGCATGGAATGTCTAATTTTTUGGTCAGAATCTCGACAGTAATG
Millettia pinnata                TCCGAGGGCATGGAATGTCTAATTTTTUGGTCAGAATCTCGACAGTAATG
RRIM600_rpl5                     TCCGAGGGCATGGAATGTCTAATTTTTCGGTCAGAATCTNGACAGTAATG
RRIC110_rpl5                     TCCGAGGGCATGGAATGTCTAATTTTTCGGTCAGAATCTUGACAGTAATG
Beta vulgaris maritima           TCCGAGGGCATGGAATGTTTCATTTTTTGGTCAGAATCTCGACAGTAATG
Beta vulgaris vulgaris           TCCGAGGGCATGGAATGTTTCATTTTTTGGTCAGAATCTCGACAGTAATG
Beta macrocarpa                  TCCGAGGGCATGGAATGTTTCATTTTTTGGTCAGAATCTCGACAGTAATG
Silene latifolia                 TCCGAGGGCATGGAATGTCTCATTTTTTGGTAAGAATCTUGACAGTAATG
RRIT251_rpl5                     TCCGAGGGCATGGAATGTCTAATTTTTCGGTCAGAATCTUGACAGTAATG
PB235_rpl5                       TCCGAGGGCATGGAATGTCTAATTTTTCGGTCAGAATCTUGACAGTAATG
BPM24_rpl5                       TCCGAGGGCATGGAATGTCTAATTTTTCGGTCAGAATCTUGACAGTAATG
Oryza sativa Japonica            TCCGAGGGCATGGAATGTACCATTTTTTGGTCAGAATCTTGACAGTAATG
                                 *****************    *****  *** *******  *********

Citrullus lanatus                TCTCTCTTAGATTCTCCGGTCGAAATACGGGAAAACTCCATTCAATTCTC
```

```
Cucurbita pepo              TCTCTCTTAGATTCTCCGGTCGAAATACGGGAAAACTCCATGCAATTCTC
Arabidopsis thaliana        TCTCTATTAGATTTTCCGGTCGAAATACGGAAAAACTCCATTCAATTCTC
Brassica napus              TCTCTGTTAGATTTTCCGGTCGAAATACGGAAAAACTCCATTCAATTCTC
RRII105_rpl5                TCTCTCTTAGATTCTCCGGTCGAAATACGGGAAAACTCCATTCAATTCTC
Lotus japonicus             TCTCTGTTAGATTCTCTGGTCGAAATACGGGAAAACTCCATTCAATTCTC
Millettia pinnata           TCTCTGTTAGATTCTCCGGTCGAAATACGGGAAAACTCCATTCAATTCTC
RRIM600_rpl5                TCTCTCTTAGATTCTCCGGTCGAAATACGGGAAAACTCCATTCAATTCTC
RRIC110_rpl5                TCTCTCTTAGATTCTCCGGTCGAAATACGGGAAAACTCCATTCAATTCTC
Beta vulgaris maritima      TCTCTCTTAGATTCTCTGGTGGAAATACGGGAAAACTCCATTCAATTCTC
Beta vulgaris vulgaris      TCTCTCTTAGATTCTCTGGTGGAAATACGGGAAAACTCCATTCAATTCTC
Beta macrocarpa             TCTCTCTTAGATTCTCTGGTGGAAATACGGGAAAACTCCATTCAATTCTC
Silene latifolia            TCTCTCTTAGATTCTCTGGTCGAAATACGGGAAAACTCCATTCAATTCTC
RRIT251_rpl5                TCTCTCTTAGATTCTCCGGTCGAAATACGGGAAAACTCCATTCAATTCTC
PB235_rpl5                  TCTCTCTTAGATTCTCCGGTCGAAATACGGGAAAACTCCATTCAATTCTC
BPM24_rpl5                  TCTCTCTTAGATTCTCCGGTCGAAATACGGGAAAACTCCATTCAATTCTC
Oryza sativa Japonica       TCTATGTTAGATTCTCCGGTCGAAATATGGGAAAACTCCATCAAATTCTT
                            *** * ******* ** *** ****** ** **********  ****** 

Citrullus lanatus           GATGGAAACGGAGTTTTGCGAATTCTCCCCGGAACTGGAAGATCATTTCG
Cucurbita pepo              GATGGAAACAGAGTTTTGCTCATTCTCCCCGGAACTAGAAGATCATTTCG
Arabidopsis thaliana        GATGGAAACGGAGTTTTGCGAATTCTCCCCGGAACTGGAAGATCATTTCG
Brassica napus              GATGGAAACGGAGTTTTGCGAATTCTCCCCGGAACTGGAAGATCATTTCG
RRII105_rpl5                GATGGAAACGGAGTTTTGCGAATTCTCCCCAGAACTGGAAGATCATTTCG
Lotus japonicus             GATGGAAACGGAGTTTTGCGAATTCTCCCCGGAACTGGAAGATCATTTCG
Millettia pinnata           GATGGAAACGGAGTTTTGCGAATTCTCCCCGGAACTGGAAGATCATTTCG
RRIM600_rpl5                GATGGAAACGGAGTTTTGCGAATTCTCCCCAGAACTGGAAGATCATTTCG
RRIC110_rpl5                GATGGAAACGGAGTTTTGCGAATTCTCCCCAGAACTGGAAGATCATTTCG
Beta vulgaris maritima      GATGGAAACGGAGTTTTGCGAATTCTCCCCGGAACTAGAAGATCATTTTG
Beta vulgaris vulgaris      GATGGAAACGGAGTTTTGCGAATTCTCCCCGGAACTAGAAGATCATTTTG
Beta macrocarpa             GATGGAAACGGAGTTTTGCGAATTCTCCCCGGAACTAGAAGATCATTTTG
Silene latifolia            GATGGAAACGGAGTTTTGCGAATTCTCCCCGGAACTAGAAGATCATTTTG
RRIT251_rpl5                GATGGAAACGGAGTTTTGCGAATTCTCCCCAGAACTGGAAGATCATTTCG
PB235_rpl5                  GATGGAAACGGAGTTTTGCGAATTCTCCCCAGAACTGGAAGATCATTTCG
BPM24_rpl5                  GATGGAAACGGAGTTTTGCGAATTCTCCCCAGAACTGGAAGATCATTTCG
Oryza sativa Japonica       TATGGAAACGGAGTTTTGCGAATTCTCCCCGGAACTGGAAGATCATTTCG
                             ******** *********  ********* ***** *********** *

Citrullus lanatus           AGATCTTCGAACATATTCTAGGGTTCAATGTGACTATTGTCACTTCGGCC
Cucurbita pepo              AGATCTTCGAACATATTATAGGGTTCAATGTGACTATTGTCACTTCGGCC
Arabidopsis thaliana        AGATCTTCGAACATATTCGGGGGTTCAATGTGACTATTATCACTTCGGCC
Brassica napus              AGATCTTCGAACATATTAGAGGGTTCAATGTGACTATTGTCACTTCGGCC
RRII105_rpl5                AGATCCTCGAACATATTCGAGGGTTCAATGTGACTATTGTCACTTCGGCC
Lotus japonicus             AGATCTTCGAACATATTCGAGGGTTCAATGTCACTATTGTCACTTCGGCC
Millettia pinnata           AGATCTTCGAACATATTCGAGGGTTCAATGTGACTATTGTCACTTCGGCC
RRIM600_rpl5                AGATCCTCGAACATATTCGAGGGTTCAATGTGACTATTGTCACTTCGGCC
RRIC110_rpl5                AGATCCTCGAACATATTCGAGGGTTCAATGTGACTATTGTCACTTCGGCC
Beta vulgaris maritima      AGATCTTCGAACATATTCGAGGGTTCAATGTAACTATTGTCACTTCAGCC
Beta vulgaris vulgaris      AGATCTTCGAACATATTCGAGGGTTCAATGTAACTATTGTCACTTCAGCC
Beta macrocarpa             AGATCTTCGAACATATTCGAGGGTTCAATGTAACTATTGTCACTTCAGCC
Silene latifolia            AAATCTTCGAACATATTCGAGGGTTCAATGTTACTATTGTCACTTCAGCC
RRIT251_rpl5                AGATCCTCGAACATATTCGAGGGTTCAATGTGACTATTGTCACTTCGGCC
PB235_rpl5                  AGATCCTCGAACATATTCGAGGGTTCAATGTGACTATTGTCACTTCGGCC
BPM24_rpl5                  AGATCCTCGAACATATTCGAGGGTTCAATGTGACTATTGTCACTTCGGCC
Oryza sativa Japonica       AGATCTTCGAGCATATTCGAGGGTTCAATGTGACTATTGTCACTTCGGCC
                            * *** **** ******   *********** ****** ******* ***

Citrullus lanatus           AACACACAAGATGAGACTTTACUACUGTGGAGCGGCTTTTTGCAAAAAGA
Cucurbita pepo              AACACACAAGATGAGACTTTACUACUGTGGAGCGGCTTTTTGCAAAAAGA
Arabidopsis thaliana        AACACACAAGATGAGACTTTACUACTGTGGAGCGGTTTTTTGCAAAAAGA
Brassica napus              AACACACAAGATGAGACTTTACUACTGTGGAGCGGCTTTTTGCAAAAAGA
RRII105_rpl5                AACACACAAGATGAGACTTTACUACUGTGGAGCGGCTTTTTTCAAAAAGA
Lotus japonicus             AACACACAAGATGAGACTTTACUACUGTGGAGCGGCTTTTTGCAAAAAGA
```

```
Millettia pinnata             AACACACAAGATGAGACTTTACUACUGTGGAGCGGCTTTTTGCAAAAAGA
RRIM600_rpl5                  AACACACAAGATGAGACTTTACNACNGTGGAGCGGCTTTTTTCAAAAAGA
RRIC110_rpl5                  AACACACAAGATGAGACTTTACUACUGTGGAGCGGCTTTTTTCAAAAAGA
Beta vulgaris maritima        AACACACAAGATGAGACTTTACTACUGTGGAGCGGCTTTTTGCAAAAAGA
Beta vulgaris vulgaris        AACACACAAGATGAGACTTTACTACUGTGGAGCGGCTTTTTGCAAAAAGA
Beta macrocarpa               AACACACAAGATGAGACTTTACTACUGTGGAGCGGCTTTTTGCAAAAAGA
Silene latifolia              AACACACAAGATGAGACTTTACTATUGTGGAGCGGCTTTTTGCAAAAAGA
RRIT251_rpl5                  AACACACAAGATGAGACTTTACNACNGTGGAGCGGCTTTTTTCAAAAAGA
PB235_rpl5                    AACACACAAGATGAGACTTTACUACUGTGGAGCGGCTTTTTTCAAAAAGA
BPM24_rpl5                    AACACACAAGATGAGACTTTACUACUGTGGAGCGGCTTTTTTCAAAAAGA
Oryza sativa Japonica         AATACAAAAGATGAGACTTTACTACTGTGGAGCGGCTTTTTGCTAAAAGA
                              ** *** *************** *  ********* ***** * ******

Citrullus lanatus             TGAGGGGGAAACTCAGTAAGAT--GTTGGAGAAGCGAAATATAC--GAGA
Cucurbita pepo                TGAGGGGGAAACTCAGTAAGA------GGAGAAGCTAAATATACACGAGA
Arabidopsis thaliana          TGAGGGGGAAACTCAGTAAGA--TGTCGTAGAAGCAAAATAGTA--GAGA
Brassica napus                TGAGGGGGAAACTCAGTAAGA--TGTCGGAGAAGCAAAATAGTA--GAGA
RRII105_rpl5                  TGAGGGGGAAAGTCAGTAA-------------------------------
Lotus japonicus               TGAGGGAGAAACTCAGTAAGAGATGTCGGAGAAGCGAAATATAC--GAGA
Millettia pinnata             TGAGGGAGAAACTCAGTAAGAGATGTCGGAGAAGCGAAATATAC--GAGA
RRIM600_rpl5                  TGAGGGGGAAAGTCAGTAA-------------------------------
RRIC110_rpl5                  TGAGGGGGAAAGTCAGTAA-------------------------------
Beta vulgaris maritima        TGAGGGAGAAACTCAGTAAGA--TGTCGGAAAATCGAAATATAC--GAGA
Beta vulgaris vulgaris        TGAGGGAGAAACTCAGTAAGA--TGTCGGAAAATCGAAATATAC--GAGA
Beta macrocarpa               TGAGGGAGAAACTCAGTAAGA--TGTCGGAAAATCGAAATATAC--GAGA
Silene latifolia              TGAGGG------TCAGTAAGA--TGTCGGAAAAGCTAAATATAC--GAGA
RRIT251_rpl5                  TGAGGGGGAAAGTCAGTAA-------------------------------
PB235_rpl5                    TGAGGGGGAAAGTCAGTAA-------------------------------
BPM24_rpl5                    TGAGGGGGAAAGTCAGTAA-------------------------------
Oryza sativa Japonica         TGAGGGGGAAACTAAGTAAGA--TGTCGGAGAAGCGAAATATAC--GAGA
                              ******      * *****

Citrullus lanatus             TCACAAACGTAGATTGCTCGCGGCTAAATATGAATTGAGAC-GAAAGCTT
Cucurbita pepo                TCACAAACGTAGATTGCTCGCGGCTAAATATGAATTGAGAC-GAAAGCTT
Arabidopsis thaliana          TCACAAACGTAGATTGCTCGCGGCTAAATTTGAATTGAGAC-GAAAGCTT
Brassica napus                TCACAAACGTAGATTGCTCGCGGCTAAATTTGAATTGAGAC-GAAAGCTT
RRII105_rpl5                  --------------------------------------------------
Lotus japonicus               TCACAAACGCAGATTGCTCGCGGCTAAATATGAATTGAGAC-GAAAGCTT
Millettia pinnata             TCACAAACGCAGATTGCTCGCGGCTAAATATGAATTGAGAC-GAAAGCTT
RRIM600_rpl5                  --------------------------------------------------
RRIC110_rpl5                  --------------------------------------------------
Beta vulgaris maritima        TCACAAA----CATTGCTCGCAGCTAAAACTAAAAGACGTTAGAAAGCTT
Beta vulgaris vulgaris        TCACAAA----CATTGCTCGCAGCTAAAACTAAAAGACGTTAGAAAGCTT
Beta macrocarpa               TCACAAA----CATTGCTCGCAGCTAAAACTAAAAGACGTTAGAAAGCTT
Silene latifolia              TCACGTA----GATTGCTCGCAGCTAAAAGTAAAAGACGAAAGCTTGCTT
RRIT251_rpl5                  --------------------------------------------------
PB235_rpl5                    --------------------------------------------------
BPM24_rpl5                    --------------------------------------------------
Oryza sativa Japonica         TCACAAACGTAGATTGCTCGCGGCTAAATATGAATTGAGAC-GAAAGCTT


Citrullus lanatus             TATTTAT-----
Cucurbita pepo                GATTTAGAT---
Arabidopsis thaliana          TATAAAG-----
Brassica napus                TATAAAG-----
RRII105_rpl5                  ------------
Lotus japonicus               TATAAAG-----
Millettia pinnata             TATAA-------
RRIM600_rpl5                  ------------
RRIC110_rpl5                  ------------
Beta vulgaris maritima        TTTGTAAAGATC
Beta vulgaris vulgaris        TTTGTAAAGATC
```

```
Beta macrocarpa                   TTTGTAAAGATC
Silene latifolia                  TCTAAAGCCTT-
RRIT251_rpl5                      ------------
PB235_rpl5                        ------------
BPM24_rpl5                        ------------
Oryza sativa Japonica             TAGAATTTTT--


###################
```

rps12 multiple sequence alignment

```
Arabidopsis thaliana              ----ATUGGGAGTAA-----AGTGATAGGGCACAAAATGGGGGGA--AAA
Brassica napus                    ----ATUGGGAGTAA-----AGTGATAGGGCAAAAAATGGGGGGGGGAAA
RRIC110_rps12                     --------------------------------------------------
Oryza sativa Japonica             -CGGATUGGGAGTAACCACTAGTGAAAGGGCAAA----GGGGGGA--AGG
Zea mays mays                     ---GATUGGGAGTAACCACTAGTGAAAGGGCTAA----GGGGGGA--AGG
PB235_rps12                       --------------------------------------------------
Beta macrocarpa                   -----TUGGGAGTAATCACTAGTGATAGGGCAAAAATAGGGGGGA--AGG
Beta vulgaris maritima            -----TUGGGAGTAATCACTAGTGATAGGGCAAAAATAGGGGGGA--AGG
Beta vulgaris vulgaris            -----TUGGGAGTAATCACTAGTGATAGGGCAAAAATAGGGGGGA--AGG
Citrullus lanatus                 ------CGGGAGTAATTACTAGTGAGAGGGCAAAAAGAGGGGGGA--AGG
Cucurbita pepo                    -----GAGTAATCACTAACTAGTGAGAGGGCAAAAAGAGGGGGGA--AGG
Lotus japonicus                   -----TUGGGAGTAATCACTAGTGATAGGGCAAAAATAGGGAGGA--AGG
Millettia pinnata                 -----TUGGGAGTAATCACTAGTGATAGGGCAAAAATAGGGAGGA--AGG
RRII105_rps12                     --------------------------------------------------
RRIT251_rps12                     --------------------------------------------------
BPM24_rps12                       --------------------------------------------------
Cycas taitungensis                TCGGATTGGGAGTAACC-----TGATAGGGCAACAATCGGGGGGG----A
RRIM600_rps12                     --------------------------------------------------
Huperzia squarrosa                ---------AAGCCA--ATTCATGGGCGATTCTATTGGCCAACGGCCAAA


Arabidopsis thaliana              AGAAAGGAAAGAGAATAATGCCCACGTTTAATCAATTGATTCGTCATGGT
Brassica napus                    AGAAAGGAAAGAGAATAATGCCTACGTTTAATCAATTGATTCGTCATGGT
RRIC110_rps12                     -----------------ATGCCTACATTAAATCAATTGATTCGTCATGGT
Oryza sativa Japonica             ACATAGGAAAGAG--GGATGCCTACAAAAAATCAATTGATTCGTCATGGT
Zea mays mays                     ACATAGGAAAGAG--GGATGCCTACAAAAAATCAATTGATTCGTCATGGT
PB235_rps12                       -----------------ATGCCTACATTAAATCAATTGATTCGTCATGGT
Beta macrocarpa                   ACAAAGGAAAGAG--CGATGCCTACAATAAATCAATTGATTCGTCATGGT
Beta vulgaris maritima            ACAAAGGAAAGAG--CGATGCCTACAATAAATCAATTGATTCGTCATGGT
Beta vulgaris vulgaris            ACAAAGGAAAGAG--CGATGCCTACAATAAATCAATTGATTCGTCATGGT
Citrullus lanatus                 ACAAAGGAAAGAG--CGATGCCTACATTAAATCAATTGATTCGTCATGGT
Cucurbita pepo                    ACAAAGGAAAGAG--CGATGCCCACATTAAATCAATTGATTCGTCATGGT
Lotus japonicus                   ACAAAGGAAAGAG--CGATGCCTACATTAAATCAATTGATTCGTCATGGT
Millettia pinnata                 ACAAAGGAAAGAG--CGATGCCTACATTAAATCAATTGATTCGTCATGGT
RRII105_rps12                     -----------------ATGCCTACATTAAATCAATTGATTCGTCATGGT
RRIT251_rps12                     -----------------ATGCCTACATTAAATCAATTGATTCGTCATGGT
BPM24_rps12                       -----------------ATGCCTACATTAAATCAATTGATTCGTCATGGT
Cycas taitungensis                AGGACGGAAAGAG--CGATGCCTACATCGAATCAATCGATTCGTCATGGT
RRIM600_rps12                     -----------------ATGCCTACATTAAATCAATTGATTCGTCATGGT
Huperzia squarrosa                AATAAAGTAAGTGAAATATGCCTACAATTAATCAAATTATACGTAATGGT
                                                   ***** **    ******   ** *** *****

Arabidopsis thaliana              AGAGAAGAAAAACGGCGCACGGACCGTACTCGAGCTTTGGATAAATGTCC
Brassica napus                    AGAGAAGAAAAACGGCGCACGGACCGTACTCGAGCTTTGGATAAATGTCC
RRIC110_rps12                     AGAGAAGAAAAACGGCGCACGGACCGTACTCGAGCTTUGGATCAATGTCC
Oryza sativa Japonica             AGAGAAGAAAAACAGCGCACGGACCGTACTCGAGCTTCGGATCAATGTCC
Zea mays mays                     AGAGAAGAAAAACGGCGCACGGACCGTACTCGAGCTTUGGATCAATGTCC
PB235_rps12                       AGAGAAGAAAAACGGCGCACGGACCGTACTCGAGCTTUGGATCAATGTCC
Beta macrocarpa                   AGAGAAGAAAAACGGCGCACGGACCGTACTCGAGCTTUGGATCAATGTCC
Beta vulgaris maritima            AGAGAAGAAAAACGGCGCACGGACCGTACTCGAGCTTUGGATCAATGTCC
Beta vulgaris vulgaris            AGAGAAGAAAAACGGCGCACGGACCGTACTCGAGCTTUGGATCAATGTCC
Citrullus lanatus                 AGAGAAGAAAAACGACGCACTGACCGTACTCGAGCTTUGGATCAATGTCC
```

```
Cucurbita pepo               AGAGAAGAAAAACGGCGCACGGACCGTACTCGAGCTTUGGATCAATGTCC
Lotus japonicus              AGAGAAGAAAAACGGCGCACGGACCGTACTCGAGCTTUGGATCAATGTCC
Millettia pinnata            AGAGAAGAAAAACGGCGCACGGACCGTACTCGAGCTTUGGATCAATGTCC
RRII105_rps12                AGAGAAGAAAAACGGCGCACGGACCGTACTCGAGCTTUGGATCAATGTCC
RRIT251_rps12                AGAGAAGAAAAACGGCGCACGGACCGTACTCGAGCTTUGGATCAATGTCC
BPM24_rps12                  AGAGAAGAAAAACGGCGCACGGACCGTACTCGAGCTTUGGATCAATGTCC
Cycas taitungensis           AGAGAGAAAAAACGGCGCACAGACCGTACTCGAGCTTCGGATAAATGTCC
RRIM600_rps12                AGAGAAGAAAAACGGCGCACGGACCGTACTCGAGCTTUGGATCAATGTCC
Huperzia squarrosa           AGAGAAAAAAAGAGGCGCACACTACGCACTCGAGCTTTAAAGAAGTGTCC
                             *****  ****    *****    ** **********   *  * *****

Arabidopsis thaliana         UCAGAAAACAGGAGTATGCCUGCGTGTTTCAACGAGAACACCGAAAAAAC
Brassica napus               CCAGAAGCTAGGAGCATGCCUGCGTGTTTCAACGAGAACACCGAAAAAAC
RRIC110_rps12                CCAGAAGCAAGGAGTAUGCCCGCGTGTTTCAACGAGAACACCGAAAAAAC
Oryza sativa Japonica        CCAAAAGCAAGGAGTATGCCTGCGTGTTTCGACGAGAACACCGAAAAAAC
Zea mays mays                CCAGAAGCAAGGAGTATGCCTGCGTGTTTCGACGAGAACACCGAAAAAAC
PB235_rps12                  CCAGAAGCAAGGAGTAUGCCCGCGTGTTTCAACGAGAACACCGAAAAAAC
Beta macrocarpa              CCAGAAGCAAGGAGTAUGCCUGCGTGTTTCAACGAGAACACCGAAAAAAC
Beta vulgaris maritima       CCAGAAGCAAGGAGTAUGCCUGCGTGTTTCAACGAGAACACCGAAAAAAC
Beta vulgaris vulgaris       CCAGAAGCAAGGAGTAUGCCUGCGTGTTTCAACGAGAACACCGAAAAAAC
Citrullus lanatus            CCAGAAGCAAGGAGTAUGCCUGCGTGTTTCAACGAGAACACCGAAAAAAC
Cucurbita pepo               CCAGAAGCAAGGAGTAUGCCUCCGTCTTTCAACGAGAACACCGAAAAAAC
Lotus japonicus              CCAGAAGCAAGGAGTAUGCCUGCGTGTTTCAACGAGAACGCCGAAAAAAC
Millettia pinnata            CCAGAAGCAAGGAGTAUGCCUGCGTGTTTCAACGAGAACACCGAAAAAAC
RRII105_rps12                CCAGAAGCAAGGAGTAUGCCCGCGTGTTTCAACGAGAACACCGAAAAAAC
RRIT251_rps12                CCAGAAGCAAGGAGTAUGCCCGCGTGTTTCAACGAGAACACCGAAAAAAC
BPM24_rps12                  CCAGAAGCAAGGAGTAUGCCCGCGTGTTTCAACGAGAACACCGAAAAAAC
Cycas taitungensis           TCAGAAGCAAGGAGTACGCCCGCGTGTTCCGACGGGAACACCAAAAAAAC
RRIM600_rps12                CCAGAAGCAAGGAGTAUGCCCGCGTGTTTCAACGAGAACACCGAAAAAAC
Huperzia squarrosa           TCAAAAGCAAGGAGTATGCCTGCGTGTTTCGACAAGAACACCAAAAAAAC
                              ** **   ***** * ***  *** ** * **  **** ** *******

Arabidopsis thaliana         CTAATTCCGCTCUACGTAAGATAGCCAAAGTACGGTTGAGCAATCGACAT
Brassica napus               CGAATTCCGCTCUACGTAAGATAGCCAAAGTACGGTTGAGCAATCGACAT
RRIC110_rps12                CTAATTCAGCTCUACGTAAGATAGCCAAAGTACGGTTGAGCAATCGACAT
Oryza sativa Japonica        CTAATTCAGCTCTACGTAAGATAGCAAAAGTACGGTTGAGCAATCGACAT
Zea mays mays                CTAATTCAGCTCTACGTAAGATAGCAAAAGTACGGTTGAGCAATCGACAT
PB235_rps12                  CTAATTCAGCTCUACGTAAGATAGCCAAAGTACGGTTGAGCAATCGACAT
Beta macrocarpa              CTAATTCAGCTCTACGTAAGATAGCCAAAGTACGGTTGAGCAATCGACAT
Beta vulgaris maritima       CTAATTCAGCTCTACGTAAGATAGCCAAAGTACGGTTGAGCAATCGACAT
Beta vulgaris vulgaris       CTAATTCAGCTCTACGTAAGATAGCCAAAGTACGGTTGAGCAATCGACAT
Citrullus lanatus            CTAATTCAGCTCUACGTAAGATAGCCAAAGTACGGTTGAGCAATCGACAT
Cucurbita pepo               CTAATTCAGCTCUACGTAAGATAGCCAAAGTACGGTTGAGCAATCGACAT
Lotus japonicus              CAAATTCAGCTCUACGTAAGATAGCCAAAGTACGATTGAGCAATCGACAT
Millettia pinnata            CAAATTCAGCTCUACGTAAGATAGCCAAAGTACGATTGAGCAATCGACAT
RRII105_rps12                CTAATTCAGCTCUACGTAAGATAGCCAAAGTACGGTTGAGCAATCGACAT
RRIT251_rps12                CTAATTCAGCTCUACGTAAGATAGCCAAAGTACGGTTGAGCAATCGACAT
BPM24_rps12                  CTAATTCAGCTCUACGTAAGATAGCCAAAGTACGGTTGAGCAATCGACAT
Cycas taitungensis           CTAATCCAGCTCCACGTAAGATAGCCAAAGTACGGTTGAGCAATCAACAT
RRIM600_rps12                CTAATTCAGCTCUACGTAAGATAGCCAAAGTACGGTTGAGCAATCGACAT
Huperzia squarrosa           CTAATTCAGCTTTACGCAAGACAGCCAAAGTACGTTTAACCAATCGAAAT
                             * *** * ***  *** **** *** ******** ** * ***** * **

Arabidopsis thaliana         GATATATTTGCTUACATCCCGGGCGAAGGTCATAATTUGCAGGAACATTC
Brassica napus               GATATATTTGCTUACATTCCGGGCGAAGGTCATAATTUGCAGGAACATTC
RRIC110_rps12                GATATATTTGCTUACATTCCAGGCGAAGGTCATAATTTGCAGGAACATTC
Oryza sativa Japonica        GATATATTTGCTCACATTCCAGGCGAAGGTCATAATTCGCAGGAACATTC
Zea mays mays                GATATATTTGCTUACATTCCAGGCGAAGGTCATAATTUGCAGGAACATTC
PB235_rps12                  GATATATTTGCTUACATTCCAGGCGAAGGTCATAATTTGCAGGAACATTC
Beta macrocarpa              GATATATTTGCTUACATTCCGGGCGAAGGTCATAATTTGCAGGAACATTC
Beta vulgaris maritima       GATATATTTGCTUACATTCCGGGCGAAGGTCATAATTTGCAGGAACATTC
Beta vulgaris vulgaris       GATATATTTGCTUACATTCCGGGCGAAGGTCATAATTTGCAGGAACATTC
```

```
Citrullus lanatus                 GATATATTTGCTCACATTCCGGGCGAAGGTCATAATTTGCAGGAACATTC
Cucurbita pepo                    GATATATTTGCTCACATTCCGGGCGAAGGTCATAATTTGCAGGAACATTC
Lotus japonicus                   GATATATTTGCTUACATTCCGGGCGAAGGTCATAATTUGCAGGAACATTC
Millettia pinnata                 GATATATTTGCTUACATTCCGGGCGAAGGTCATAATTUGCAGGAACATTC
RRII105_rps12                     GATATATTTGCTUACATTCCAGGCGAAGGTCATAATTTGCAGGAACATTC
RRIT251_rps12                     GATATATTTGCTUACATTCCAGGCGAAGGTCATAATTTGCAGGAACATTC
BPM24_rps12                       GATATATTTGCTUACATTCCAGGCGAAGGTCATAATTTGCAGGAACATTC
Cycas taitungensis                GATATATTTGCTTACATTCCGGGCGAAGGTCATAATCTGCAGGAACATCC
RRIM600_rps12                     GATATATTTGCTUACATTCCAGGCGAAGGTCATAATTTGCAGGAACATTC
Huperzia squarrosa                GTAATAATTGCTTACATTCCCGGCGAAGGTCATAATTTGCAAGAGCATTC
                                  *  *** ***** **** ** ***************  *** ** *** *

Arabidopsis thaliana              TACGGTGTTAATAAGAGGAGGTAGAGTGAAAGATTUGCCAGGTGTAAAAT
Brassica napus                    GCAGGTGTTAATAAGAGGAGGTAGAGTGAAAGATTUGCCAGGTGTAAAAT
RRIC110_rps12                     TATGGTGTTAATAAGAGGAGGTAGAGTAAAGGATTUGCCAGGTGTGAAAT
Oryza sativa Japonica             TATAGTCTTAGTCAGAGGAGGTAGAGTGAAAGATTCGCCAGGTGTGAAAT
Zea mays mays                     TATAGTCTTAGTCAGAGGGGGTAGAGTGAAAGATTUGCCAGGTGTGAAAT
PB235_rps12                       TATGGTGTTAATAAGAGGAGGTAGAGTAAAGGATTUGCCAGGTGTGAAAT
Beta macrocarpa                   TATGGTCTTAATAAGAGGAGGTAGAGTGAAAGATTUGCCAGGTGTGAAAT
Beta vulgaris maritima            TATGGTCTTAATAAGAGGAGGTAGAGTGAAAGATTUGCCAGGTGTGAAAT
Beta vulgaris vulgaris            TATGGTCTTAATAAGAGGAGGTAGAGTGAAAGATTUGCCAGGTGTGAAAT
Citrullus lanatus                 TATGGTGTTAATAAGAGGAGGTAGAGTGAAAGATTUGCCAGGTGTGAAAT
Cucurbita pepo                    TATGGTCTTAATAAGAGGAGGTAGAGTGAAAGATTUGCCAGGTGTGAAAT
Lotus japonicus                   TATGGTGTTAATAAGAGGAGGTAGAGTGAAAGATTTGCCAGGTGTGAAAT
Millettia pinnata                 TATGGTGTTAATAAGAGGAGGTAGAGTGAAAGATTTGCCAGGTGTGAAAT
RRII105_rps12                     TATGGTGTTAATAAGAGGAGGTAGAGTAAAGGATTUGCCAGGTGTGAAAT
RRIT251_rps12                     TATGGTGTTAATAAGAGGAGGTAGAGTAAAGGATTUGCCAGGTGTGAAAT
BPM24_rps12                       TATGGTGTTAATAAGAGGAGGTAGAGTAAAGGATTUGCCAGGTGTGAAAT
Cycas taitungensis                TATGGTGTCGATCAGAGGAGGTAGAGTGAAAGATTCGCCAGGTGCGAAAT
RRIM600_rps12                     TATGGTGTTAATAAGAGGAGGTAGAGTAAAGGATTNGCCAGGTGTGAAAT
Huperzia squarrosa                TGTGGTTATGGTTAGAGGAGGTAGAGTAAAAGATTTGCCAGGTGTGAAAT
                                      **     * ***** ******** ** **** ********  ****

Arabidopsis thaliana              UUCATTGTATTCGAGGAGTAAAGGATTTGATGGGAATTCCGGGTCGAAGA
Brassica napus                    UUCATTGTATTCGAGGAGTAAAGGATTTGATGGGAATTCCGGGTCGAAGA
RRIC110_rps12                     UCCATTGTATTCGAGGAGTCAAGGATTTGCTGGGAATTCCGGATCGAAGA
Oryza sativa Japonica             CCCATCGTATTCGAGGAGTCAAGGATTTACTGGGAATTCCGGATCGTAGA
Zea mays mays                     UCCATUGTATTCGAGGAGTAAAGGATTTGCTGGGAATTCCGGATCGTAGA
PB235_rps12                       UCCATTGTATTCGAGGAGTCAAGGATTTGCTGGGAATTCCGGATCGAAGA
Beta macrocarpa                   UCCATTGTATTCGAGGAGTCAAGGATTTGCTGGGAATTCCGGATCGAAGA
Beta vulgaris maritima            UCCATTGTATTCGAGGAGTCAAGGATTTGCTGGGAATTCCGGATCGAAGA
Beta vulgaris vulgaris            UCCATTGTATTCGAGGAGTCAAGGATTTGCTGGGAATTCCGGATCGAAGA
Citrullus lanatus                 UCCATTGTATTCGAGGAGTCAAGGATTTGCTGGGAATTCCGGATCGAAGA
Cucurbita pepo                    UCCATTGTATTCGAGGAGTCAAGGATTTGCTGGGAATTCCGGATCGAAGA
Lotus japonicus                   UCCATTGTATTCGAGGAGTCAAGGATTTGTTGGGAATTCCGGATCGAAGA
Millettia pinnata                 UCCATTGTATTCGAGGAGTCAAGGATTTGTTGGGAATTCCGGATCGAAGA
RRII105_rps12                     UCCATTGTATTCGAGGAGTCAAGGATTTGCTGGGAATTCCGGATCGAAGA
RRIT251_rps12                     UCCATTGTATTCGAGGAGTCAAGGATTTGCTGGGAATTCCGGATCGAAGA
BPM24_rps12                       UCCATTGTATTCGAGGAGTCAAGGATTTGCTGGGAATTCCGGATCGAAGA
Cycas taitungensis                TCCATCGTATTCGAGGAGTCAAAGATTTGCTGGGAACTCCGGGTCGAAAA
RRIM600_rps12                     NCCATTGTATTCGAGGAGTCAAGGATTTGCTGGGAATTCCGGATCGAAGA
Huperzia squarrosa                ACCATTGTATTCGAGGAGTCAAAGATTTGCAGGGAATCCCGGGTCGACGT
                                    *** ************* ** *****   *****  **** ***

Arabidopsis thaliana              AGAGGCAGATCAAAATATGGTGCAGAAAAACCCAA--ATCGA-TATGAAT
Brassica napus                    AGAGGCAGATCAAAATATGGTGCAGAAAAACCCAA--ATCGA-TATGAAT
RRIC110_rps12                     AGAGGCAGATCAAAATATGGTGCGGAAAAACCCAA--ATCGA-TATGA--
Oryza sativa Japonica             AAGGGAAGATCTAAATATGGTGCAGAAAGACCAAA--ATCGA-AATGAAT
Zea mays mays                     AAGGGAAGATCTAAATATGGCGCAGAAAGACCAAA--ATCTA-AATGAAT
PB235_rps12                       AGAGGCAGATCAAAATATGGTGCGGAAAAACCCAA--ATCGA-TATGA--
Beta macrocarpa                   AGAGGCAGATCAAAATATGGTGCAGAAAAACCCAA--ATCGA-TATGAAT
Beta vulgaris maritima            AGAGGCAGATCAAAATATGGTGCAGAAAAACCCAA--ATCGA-TATGAAT
```

```
Beta vulgaris vulgaris              AGAGGCAGATCAAAATATGGTGCAGAAAAACCCAA--ATCGA-TATGAAT
Citrullus lanatus                   AGAGGCAGATCTAAATATGGTGCAGAAAAACCAAA--ATAGA-GAGGAAT
Cucurbita pepo                      AGAGGAAGATCTAAATATGGTGCAGAAAAACCTAA--ATCGA-TATGAAT
Lotus japonicus                     AGAGGCAGATCAAAATATGGTGCAGAAAAACCCAA--ATCGA-TATGAAT
Millettia pinnata                   AGAGGCAGATCCAAATATGGTGCAGAAAAACCCAA--ATCGA-TATGAAT
RRII105_rps12                       AGAGGCAGATCAAAATATGGTGCGGAAAAACCCAA--ATCGA-TATGA--
RRIT251_rps12                       AGAGGCAGATCAAAATATGGTGCGGAAAAACCCAA--ATCGA-TATGA--
BPM24_rps12                         AGAGGCAGATCAAAATATGGTGCGGAAAAACCCAA--ATCGA-TATGA--
Cycas taitungensis                  AGGGGCAGATCTAAATATGGTGCGAAGAGACCCAA--ATCGA-TAUGAAT
RRIM600_rps12                       AGAGGCAGATCAAAATATGGTGCGGAAAAACCCAA--ATCGA-TATGA--
Huperzia squarrosa                  CGAGGCAGATCTAAATATGGTACGAAAAAACCCAAGGATCAAATATGAAT
                                       ** ***** ********* *  * * *** **  **  *  * **

Arabidopsis thaliana                GGA-AGATGCCTCTGGAACTAGT---ATCGGTCAGTCGGTGGAACAATCA
Brassica napus                      GGA-AGATGCCTCTGGAACTAGTT--ATCGGTAAGTCTTTT-AGTAAGCG
RRIC110_rps12                       --------------------------------------------------
Oryza sativa Japonica               GGA-AGATGCCTCTGGAACTTTTTGGTTATTTTGGGT---CCAGATGAAC
Zea mays mays                       GGA-AGATGCCTCTGGAACTTATTGGTTATTTTGGGGGGGCGATATGGAA
PB235_rps12                         --------------------------------------------------
Beta macrocarpa                     GGA-AGATGCCTCTT----CTCTT---TGTCTTGGTCGGTCCAGGGAACC
Beta vulgaris maritima              GGA-AGATGCCTCTT----CTCTT---TGTCTTGGTCGGTCCAGGGAACC
Beta vulgaris vulgaris              GGA-AGATGCCTCTT----CTCTT---TGTCTTGGTCGGTCCAGGGAACC
Citrullus lanatus                   GGA-AGATGCCTCTTTCACTTGTT---TTTCTCGGTCAGTCGAGGGAACA
Cucurbita pepo                      GGA-AGATGCCTCTTTCACTTCTT---TTTCTCGGGCAGTCGAGGGAAAG
Lotus japonicus                     GGA-GGATGCCTCTGGAACTTCTT---TTTCTCGGTCAGGAGAGGTACGA
Millettia pinnata                   GGA-GGATGCCTCTGGAACTTCTT---TTTCTCGGTCAGGAGAGGTACGA
RRII105_rps12                       --------------------------------------------------
RRIT251_rps12                       --------------------------------------------------
BPM24_rps12                         --------------------------------------------------
Cycas taitungensis                  GAA-AGACGAAAAACCAAAGATTCTAATAAATACTTGATCTTAATCAATG
RRIM600_rps12                       --------------------------------------------------
Huperzia squarrosa                  TCATGGGTAAAATCATCGAATTTACGCTGTGTTTTTGGTTCATACTTTGA


Arabidopsis thaliana                CAACGTTACG---
Brassica napus                      TA-TATAAA----
RRIC110_rps12                       -------------
Oryza sativa Japonica               GCAAGTCG-----
Zea mays mays                       GCAGCTA------
PB235_rps12                         -------------
Beta macrocarpa                     CGCGTTAGGCCA-
Beta vulgaris maritima              CGCGTTAGGCCA-
Beta vulgaris vulgaris              -GCGTTAGGCCAA
Citrullus lanatus                   A------------
Cucurbita pepo                      GGAACAAC-----
Lotus japonicus                     AGTCACTC-----
Millettia pinnata                   AGTCACTC-----
RRII105_rps12                       -------------
RRIT251_rps12                       -------------
BPM24_rps12                         -------------
Cycas taitungensis                  TGAATAT------
RRIM600_rps12                       -------------
Huperzia squarrosa                  TGGGTT-------
```

###################

rps13 multiple sequence alignment

```
Beta macrocarpa                     ---AGACAGGCCATTTATGATTCCAGCCTAGAAGACTAGTTAAAGGA--A
Beta vulgaris maritima              ---AGACAGGCCATTTATGATTCCAGCCTAGAAGACTAGTTAAAGGA--A
Beta vulgaris vulgaris              -----ACAGGCCATTTATGATTCCAGCCTAGAAGACTAGTTAAAGGA--A
Citrullus lanatus                   ---AGACAACTCATTTATGATTCCAGCCGAGAAGACTAGTAAAAGGA--A
Cucurbita pepo                      -------AACTAATTTCTTATTCCAGCCTAGAAGACTAGTAAAAGGAGAA
```

```
RRII105_rps13                     --------------------------------------------------
RRIT251_rps13                     --------------------------------------------------
Oryza sativa Japonica             ------TAGTCCATTGTAGATTCCAGCCGAGAAGACCAGGAAAAGAG-AA
Zea mays mays                     ----AGACAACCATTGTAGATTCCAGCCGAGAAGACCAGGAAAAGAG-AA
RRIC110_rps13                     --------------------------------------------------
RRIM600_rps13                     --------------------------------------------------
PB235_rps13                       --------------------------------------------------
BPM24_rps13                       --------------------------------------------------
Megaceros aenigmaticus            --TATGCAATATCGAAACGAGATTAGACATAAAAAGCAAGGAAA-AA--A
Phaeoceros laevis                 -GTATACAATATCGAAAGGAGATTACACATAAAAA-CAAGGAAA-AA--A
Huperzia squarrosa                GGTATACAATATCGGAACGAAATTATGCATAAGAAACAAGGAAAGAA--A


Beta macrocarpa                   GGATCAAGAATGTCATATATTTCAGGAGCTAGATTAGTTGACGATAAACA
Beta vulgaris maritima            GGATCAAGAATGTCATATATTTCAGGAGCTAGATTAGTTGACGATAAACA
Beta vulgaris vulgaris            GGATCAAGAATGTCATATATTTCAGGAGCTAGATTAGTTGACGATAAACA
Citrullus lanatus                 GGATCAAGAATGTCATATATTTCAGGAGCAAGATUTGTTGCCGATAAACA
Cucurbita pepo                    GGAGAAAGAATGCCATATATTTCAGGAGCTAGATUAGTTGCCGATAAACA
RRII105_rps13                     ---------ATGTCATATATTTCAGGAGCTCGATTAGTTGCCGATGAACA
RRIT251_rps13                     ---------ATGTCATATATTTCAGGAGCTCGATTAGTTGCCGATGAACA
Oryza sativa Japonica             GGATAAAGAATGTUATATATUTUAGGAGCTAGATUACTTCCCGATGAACA
Zea mays mays                     GGATAAAGAATGTUATATATCTCAGGAGCTAGATUACTTCCCGATGAACA
RRIC110_rps13                     ---------ATGTCATATATTTCAGGAGCTCGATTAGTTGCCGATGAACA
RRIM600_rps13                     ---------ATGTCATATATTTCAGGAGCTCGATTAGTTGCCGATGAACA
PB235_rps13                       ---------ATGTCATATATTTCAGGAGCTCGATTAGTTGCCGATGAACA
BPM24_rps13                       ---------ATGTCATATATTTCAGGAGCTCGATTAGTTGCCGATGAACA
Megaceros aenigmaticus            GAATCAATCATGTCATATATTTTAGGAACTAATTCAATTTCCAATAAACA
Phaeoceros laevis                 GAATAAATCATGTCATATATTTTAGGAACTAATTCAATTTCCAATAAACT
Huperzia squarrosa                AAAUAAATCAUGCCTCATATTTTAGGAGCTAATTTAGTTTCTCATAAACA
                                           * *    **** * **** *    *   **    ** ***

Beta macrocarpa                   AGTAAAAATTGCCTUAACAAAAATTGATGGAATTGGACCTAAAAAAGCCA
Beta vulgaris maritima            AGTAAAAATTGCCTUAACAAAAATTGATGGAATTGGACCTAAAAAAGCCA
Beta vulgaris vulgaris            AGTAAAAATTGCCTUAACAAAAATTGATGGAATTGGACCTAAAAAAGCCA
Citrullus lanatus                 AGTCAGAATTGCCTUAACCAAAATTGATGGAATTGGACCTAAAAAAGCCA
Cucurbita pepo                    AGTCAGAATTGCCTUAACAAAAATTGATGGAATTGGACCTAAAAAAGCCA
RRII105_rps13                     AGTAAGAATTGCCTCAACAAAAATGGATGGAATTGGACCTAAAAAAGCCA
RRIT251_rps13                     AGTAAGAATTGCCTCAACAAAAATGGATGGAATTGGACCTAAAAAAGCCA
Oryza sativa Japonica             AGTCAGAATTGCCTUAACAAAAATGGATGGAATTGGACCGAAAAAAGCCA
Zea mays mays                     AGTAAGAATTGCCTUAACAAAAATGGATGGAATTGGACCGAAAAAAGCCA
RRIC110_rps13                     AGTAAGAATTGCCTCAACAAAAATGGATGGAATTGGACCTAAAAAAGCCA
RRIM600_rps13                     AGTAAGAATTGCCTCAACAAAAATGGATGGAATTGGACCTAAAAAAGCCA
PB235_rps13                       AGTAAGAATTGCCTCAACAAAAATGGATGGAATTGGACCTAAAAAAGCCA
BPM24_rps13                       AGTAAGAATTGCCTCAACAAAAATGGATGGAATTGGACCTAAAAAAGCCA
Megaceros aenigmaticus            AATAAGAATTGCCTTAATTCAAATTTTCGGAATTGGCCCCAAAAAGGCAA
Phaeoceros laevis                 AATAAGAATTGCCTTAATTCAAATTTTCGGAATTGGCCCTAAAAAGGCAA
Huperzia squarrosa                AGTAAGAATTGCCTCAACTCAAATCTTTGGAATTGGACCTCAAAAAGCAA
                                  * * * ******** **   ****    ******** **  **** ** *

Beta macrocarpa                   TTCAGGTTTGTTATCGATTAGGTATCAGTGATAACATTAAGATAAAAGAG
Beta vulgaris maritima            TTCAGGTTTGTTATCGATTAGGTATCAGTGATAACATTAAGATAAAAGAG
Beta vulgaris vulgaris            TTCAGGTTTGTTATCGATTAGGTATCAGTGATAACATTAAGATAAAAGAG
Citrullus lanatus                 TTCAGGTTUGTTATCGATTAGGTATCAGTGGGAACATAAAGATCAAAGAG
Cucurbita pepo                    TTCAGGTTUGTTATCGATTAGGTATCAGTGGGAACATCAAGATCAAAGAG
RRII105_rps13                     TTCAGGTTCGTTATCGATTAGGTATCAGTGGGAACATAAAGATAAAAGAG
RRIT251_rps13                     TTCAGGTTCGTTATCGATTAGGTATCAGTGGGAACATAAAGATAAAAGAG
Oryza sativa Japonica             TTCAGCTTUGTTATCGATTAGGTATCAGTGGGAACATCAAGATGAATGAG
Zea mays mays                     TTCAGCTTUGTTATCGATTAGGTATCAGTGGGAACATCAAGATTCATGAG
RRIC110_rps13                     TTCAGGTTCGTTATCGATTAGGTATCAGTGGGAACATAAAGATAAAAGAG
RRIM600_rps13                     TTCAGGTTCGTTATCGATTAGGTATCAGTGGGAACATAAAGATAAAAGAG
PB235_rps13                       TTCAGGTTCGTTATCGATTAGGTATCAGTGGGAACATAAAGATAAAAGAG
BPM24_rps13                       TTCAGGTTCGTTATCGATTAGGTATCAGTGGGAACATAAAGATAAAAGAG
```

```
Megaceros aenigmaticus            TTCAGCTTTGTTATCGCTTAGGTCTCAGTGATAACATTAGGGTGAATAAG
Phaeoceros laevis                 TTCAGCTTTGTTATCGCTTAGGTTTGAGTGATAACATTAGGGTTAATAAG
Huperzia squarrosa                TTGAGGTTTGTGATCAATTGGGTCCCAGTGGTAACATTAAGGTTAATAAG
                                  ** ** ** ** ***  ** ***   ****  ***** * * *  *  **

Beta macrocarpa                   TTAACTAAATATCAGATCGACCAAATTGAACAAATGATAGGTCAAGATCA
Beta vulgaris maritima            TTAACTAAATATCAGATCGACCAAATTGAACAAATGATAGGTCAAGATCA
Beta vulgaris vulgaris            TTAACTAAATATCAGATCGACCAAATTGAACAAATGATAGGTCAAGATCA
Citrullus lanatus                 TTAACTAAGTATCAGATCGACCAAATTGAACAAATGATAGGTCAAGATCA
Cucurbita pepo                    TTAACTAAGTCTCAGATCGACCAAATTGAACAAATGATAGGTCAAGATCA
RRII105_rps13                     TTAAATAAGTATCAGATCGACCAAATTGAACAAATGATAGGTCAAGATCA
RRIT251_rps13                     TTAAATAAGTATCAGATCGACCAAATTGAACAAATGATAGGTCAAGATCA
Oryza sativa Japonica             TTAACTAAGTATCAGATCGACCAAATTGAACAAATGATAGCTCAAGATCA
Zea mays mays                     TTAACTAAGTATCAGATCGACCAAATTGAACAAATGATAGCTCAAGATCA
RRIC110_rps13                     TTAAATAAGTATCAGATCGACCAAATTGAACAAATGATAGGTCAAGATCA
RRIM600_rps13                     TTAAATAAGTATCAGATCGACCAAATTGAACAAATGATAGGTCAAGATCA
PB235_rps13                       TTAAATAAGTATCAGATCGACCAAATTGAACAAATGATAGGTCAAGATCA
BPM24_rps13                       TTAAATAAGTATCAGATCGACCAAATTGAACAAATGATAGGTCAAGATCA
Megaceros aenigmaticus            TTAACGTCGTATCAGATTGACAAAATTCTGAAAATAATAAGTCAGAATTA
Phaeoceros laevis                 TTAACGTCGTATCAGATTGACAAAATTATAAAAATAATAAGTCAGAATTA
Huperzia squarrosa                TCAACTAACTCTCAATTTGACCAAATCATCAAAATAATGAGTCAAAATCA
                                  * **     * ***  * *** ****     **** **   ***  ** *

Beta macrocarpa                   TGTTGTTCATTGGGAATTGAAGAGGGGAGAACGAGCAGACATUGAACGAT
Beta vulgaris maritima            TGTTGTTCATTGGGAATTGAAGAGGGGAGAACGAGCAGACATUGAACGAT
Beta vulgaris vulgaris            TGTTGTTCATTGGGAATTGAAGAGGGGAGAACGAGCAGACATUGAACGAT
Citrullus lanatus                 TATTGTTCATTGGGAATTGAAGAGAGGAGAACGAGCAGACATCGAACGAT
Cucurbita pepo                    TATTGTTCATTGGGAATTGAAGAGGGGAGAACGAGCAGACATCGAACGAG
RRII105_rps13                     TGTTGTTCATTGGGAATTGAAGAGGGGAGAACGAGCAGACATCGAACGAT
RRIT251_rps13                     TGTTGTTCATTGGGAATTGAAGAGGGGAGAACGAGCAGACATCGAACGAT
Oryza sativa Japonica             TGTTGTTCATTGGGAATTGAAGAGGGGAGAACGAGCAGACATCGAACGAT
Zea mays mays                     TGTTGTTCATTGGGAATTGAAGAGGGGAGAACGAGCAGACATCGAACGAT
RRIC110_rps13                     TGTTGTTCATTGGGAATTGAAGAGGGGAGAACGAGCAGACATCGAACGAT
RRIM600_rps13                     TGTTGTTCATTGGGAATTGAAGAGGGGAGAACGAGCAGACATCGAACGAT
PB235_rps13                       TGTTGTTCATTGGGAATTGAAGAGGGGAGAACGAGCAGACATCGAACGAT
BPM24_rps13                       TGTTGTTCATTGGGAATTGAAGAGGGGAGAACGAGCAGACATCGAACGAT
Megaceros aenigmaticus            TTTAGTTGATTCGGAATTGAAAGCAGTGATTCAAAGAGATATCAAACGAT
Phaeoceros laevis                 TTTAGTTGATTCGGAATTGAAAACAGTGATTCAAAGAGACATCAAACGAT
Huperzia squarrosa                TTTAGTTGATTCGGAATCGAAGAAATCAATTCGGACAGAAATCAAACGAT
                                  * * *** *** ***** ***          *    *** **  *****

Beta macrocarpa                   TAATTTCTATTTCTTGTTATCGTGGAATTCGTCATCAAGATGGATTGCCC
Beta vulgaris maritima            TAATTTCTATTTCTTGTTATCGTGGAATTCGTCATCAAGATGGATTGCCC
Beta vulgaris vulgaris            TAATTTCTATTTCTTGTTATCGTGGAATTCGTCATCAAGATGGATTGCCC
Citrullus lanatus                 TCATTTCGATTTCTTGTTATCGTGGAATTCGTCATCAAGATGGATTGCCC
Cucurbita pepo                    TCATTTCTCTTTCTTGTTATCGTGGAATTCGTCATCAAGATGGATTGCCC
RRII105_rps13                     TAATTTCTATTTCTTGTTATCGTGGAATTCGTCATCAAGATGGATTGCCC
RRIT251_rps13                     TAATTTCTATTTCTTGTTATCGTGGAATTCGTCATCAAGATGGATTGCCC
Oryza sativa Japonica             TAATTTCTATTTCTUGTTATCGTGGAATTCGTCATCAAGATGGATUGCCC
Zea mays mays                     TAATTTCTATTTCTUGTTATCGTGGAATTCGTCATCAAGATGGATUGCCA
RRIC110_rps13                     TAATTTCTATTTCTTGTTATCGTGGAATTCGTCATCAAGATGGATTGCCC
RRIM600_rps13                     TAATTTCTATTTCTTGTTATCGTGGAATTCGTCATCAAGATGGATTGCCC
PB235_rps13                       TAATTTCTATTTCTTGTTATCGTGGAATTCGTCATCAAGATGGATTGCCC
BPM24_rps13                       TAATTTCTATTTCTTGTTATCGTGGAATTCGTCATCAAGATGGATTGCCC
Megaceros aenigmaticus            TAATCAGTATTGGTTGTTATCGTGGATTTCGTCACAACGCAGGATTACCC
Phaeoceros laevis                 TAATCAGTATTGGTTGTTATCGTGGATTTCGTCACAACGCAGGATTACCC
Huperzia squarrosa                TAATTAGTATTAGTTGTTATCGTGGATTTCGTCATAATGCAGGATTGCCC
                                  * **     **  * *********** *******  * *  ****  **

Beta macrocarpa                   TTACGCGGTCAACGAACTCATACTAATGCTAGGACTTGTCGTAAG---AA
Beta vulgaris maritima            TTACGCGGTCAACGAACTCATACTAATGCTAGGACTTGTCGTAAG---AA
Beta vulgaris vulgaris            TTACGCGGTCAACGAACTCATACTAATGCTAGGACTTGTCGTAAG---AA
```

```
Citrullus lanatus                  TTACGCGGTCAACGAACTCATACTAATGCTAGGACTTGTCGCAAG---CG
Cucurbita pepo                     TTACGCGGTCAACGAACTCATACTAATGCTAGGACTTGTCGCAAG---CG
RRII105_rps13                      TTACGCGGTCAACGAACTCATACTAATGCTAGAACTTGTCGCAAG---CT
RRIT251_rps13                      TTACGCGGTCAACGAACTCATACTAATGCTAGAACTTGTCGCAAG---CT
Oryza sativa Japonica              TTACGCGGTCAACGAACTCATACTAATGCAAGGACTGCTCGCAAG---CT
Zea mays mays                      TTACGCGGTCAACGAACTCATACTAATGCAAGGACTGCTCGCAAG---CA
RRIC110_rps13                      TTACGCGGTCAACGAACTCATACTAATGCTAGAACTTGTCGCAAG---CT
RRIM600_rps13                      TTACGCGGTCAACGAACTCATACTAATGCTAGAACTTGTCGCAAG---CT
PB235_rps13                        TTACGCGGTCAACGAACTCATACTAATGCTAGAACTTGTCGCAAG---CT
BPM24_rps13                        TTACGCGGTCAACGAACTCATACTAATGCTAGAACTTGTCGCAAG---CT
Megaceros aenigmaticus             TTACGTGGTCAACGAACTCATACTAATGCTAAGACTTGTCGCAGATTCCA
Phaeoceros laevis                  TTACGTGGTCAACGAACTCATACTAATGCTAAGACTTGTCGCAGATTCCA
Huperzia squarrosa                 TTACGCGGTCAACGAACTCATACTAATGCTAAGACTTGTCGCAAATTGAG
                                   ***** *********************** *  ***  *** *

Beta macrocarpa                    AA----TTCGGAAATGAAAGAAGTCTACCGGAA--GCCCTTGGTACTTGT
Beta vulgaris maritima             AA----TTCGGAAATGAAAGAAGTCTACCGGAA--GCCCTTGGTACTTGT
Beta vulgaris vulgaris             AA----TTCGGAAATGAAAGAAGTCTACCGGAA--GCCCTTGGTACTTGT
Citrullus lanatus                  AA----TTCGGAAATGAAATCAGTCTACCGAAA--GCCCTTGGTACTTGT
Cucurbita pepo                     AA----TTCGGAAATGAAATCAGTCTACCGAAAAAGCCCTTGGTACTTGT
RRII105_rps13                      AA----TTCGGAAATGA---------------------------------
RRIT251_rps13                      AA----TTCGGAAATGA---------------------------------
Oryza sativa Japonica              AA----TTCGGAAATGAAAGAAGGCTACCGAAAGAAC---AAGCAACGGA
Zea mays mays                      AA----TTCGGAAATGAAAGAAGGCTACCGAAAGAAC---AAGCAACGGA
RRIC110_rps13                      AA----TTCGGAAATGA---------------------------------
RRIM600_rps13                      AA----TTCGGAAATGA---------------------------------
PB235_rps13                        AA----TTCGGAAATGA---------------------------------
BPM24_rps13                        AA----TTCGGAAATGA---------------------------------
Megaceros aenigmaticus             AAACGTTTCGATCAATCAAC---------GAAGTTGATTGATGGTTCACA
Phaeoceros laevis                  AAACGTTTCGATCAATCAAC---------GAAGTTGATTGATGGTTCGCA
Huperzia squarrosa                 AAGGGTTTCGATCAAAAAACTAAAAAAAUGAGGTTGATTGATGATTTGCA
                                   **    ****   *

Beta macrocarpa                    CTGATCAATCATACTGATAGC---------------------
Beta vulgaris maritima             CTGATCAATCATACTGATAGC---------------------
Beta vulgaris vulgaris             CTGATCAATCATACTGATAGCTT-------------------
Citrullus lanatus                  CGGATCAATCACACTGATAGC---------------------
Cucurbita pepo                     CGGATCAATCACACAGATAGC---------------------
RRII105_rps13                      ------------------------------------------
RRIT251_rps13                      ------------------------------------------
Oryza sativa Japonica              TTTCGCGCTCATCCCTTGCGCCTG------------------
Zea mays mays                      TTGAGCGCTCATCCCTTGCGCC--------------------
RRIC110_rps13                      ------------------------------------------
RRIM600_rps13                      ------------------------------------------
PB235_rps13                        ------------------------------------------
BPM24_rps13                        ------------------------------------------
Megaceros aenigmaticus             AGAATTCATCATCCACAATAATCGAAGCGCAAAGCAATGTGTT
Phaeoceros laevis                  ACAATTCATCATCCATCATAATCGAAGCGCGAAGCAATGTGTT
Huperzia squarrosa                 ACGATTCATCATTCGCAATAATGAAGG---AAAGCGAAG----
```

###################

rps1 multiple sequence alignment

```
RRIC110_rps1                       --------------------------------------------------
PB235_rps1                         --------------------------------------------------
RRII105_rps1                       --------------------------------------------------
BPM24_rps1                         --------------------------------------------------
RRIM600_rps1                       --------------------------------------------------
RRIT251_rps1                       --------------------------------------------------
Citrullus lanatus                  CAGCAGCTTAGCTTAGTAGCTCAACTCTT--TCGACTGGCGTGACGCTGC
Cucurbita pepo                     CAACA-CTTAGCTTAGTAGCTCAACTCTT--TCGACTGGCGTGACGCTGC
Millettia pinnata                  -------CTGGATGAGAACTCCACTACTTAATCTACTGGCGTGGTGCTAC
```

```
Oryza sativa Japonica          --------------------------------------------------


RRIC110_rps1                   ------------------------------------------------AT
PB235_rps1                     ------------------------------------------------AT
RRII105_rps1                   ------------------------------------------------AT
BPM24_rps1                     ------------------------------------------------AT
RRIM600_rps1                   ------------------------------------------------AT
RRIT251_rps1                   ------------------------------------------------AT
Citrullus lanatus              TGGATGCTTCTGATCAATAG-----GAAGAGGAGGAAAAAAAAAGCTTAT
Cucurbita pepo                 TGGATGCTTCTGATCAATAG-----GAAGAGGAGGCAAAACAA-GCTTAT
Millettia pinnata              TGGATGCTTCTGATCAATAGAACAGGAAGAGGAGGAAAAAAAA-GCTTAT
Oryza sativa Japonica          --------------------------------------------------


RRIC110_rps1                   GACGAGCATCTATTTGAATCGATCATTTCCAAGAGCTAATTCCAGTTTTT
PB235_rps1                     GACGAGCATCTATTTGAATCGATCATTTCCAAGAGCTAATTCCAGTTTTT
RRII105_rps1                   GACGAGCATCTATTTGAATCGATCATTTCCAAGAGCTAATTCCAGTTTTT
BPM24_rps1                     GACGAGCATCTATTTGAATCGATCATTTCCAAGAGCTAATTCCAGTTTTT
RRIM600_rps1                   GACGAGCATCTATTTGAATCGATCATTTCCAAGAGCTAATTCCAGTTTTT
RRIT251_rps1                   GACGAGCATCTATTTGAATCGATCATTTCCAAGAGCTAATTCCAGTTTTT
Citrullus lanatus              GATGAGCATCTATTTGAGTCGATCATTTCCAAGATCTAATTCGAGTTTTT
Cucurbita pepo                 GATGAGCATCTATTTGAGTCGATCATTTCCAAGATCTAATTCTAGTTTTT
Millettia pinnata              GATGAGCATCTATTTGAGTCGATCATTTCCAAGATCTAATTCCAGTTTCT
Oryza sativa Japonica          --------------------------------GATCTAATTCTAGTTTCT
                                                               ** ******* ***** *

RRIC110_rps1                   CCTTATTCCGAGGTAACGCCTTACAATCTTCAGTCTTACGCTTA------
PB235_rps1                     CCTTATTCCGAGGTAACGCCTTACAATCTTCAGTCTTACGCTTA------
RRII105_rps1                   CCTTATTCCGAGGTAACGCCTTACAATCTTCAGTCTTACGCTTA------
BPM24_rps1                     CCTTATTCCGAGGTAACGCCTTACAATCTTCAGTCTTACGCTTA------
RRIM600_rps1                   CCTTATTCCGAGGTAACGCCTTACAATCTTCAGTCTTACGCTTA------
RRIT251_rps1                   CCTTATTCCGAGGTAACGCCTTACAATCTTCAGTCTTACGCTTA------
Citrullus lanatus              TCTTATGTAGTGGAAACGCCTTACAATCTGAAGTTTTACGCTTA------
Cucurbita pepo                 TCTTACGTAGTGGAAACGCCTTACAATCTTCAGTTTTACGCTTA------
Millettia pinnata              TCTTATGTAGTGGAAAGGCCTTACAATCTGAAGTTTTACGCTTA------
Oryza sativa Japonica          TATTATGTAGTGGAAACGCCTCACAATCTTCAGTTTTACGCTTACGCTTA
                                 ***    * ** ** **** *******  *** *********

RRIC110_rps1                   AGGGGTAAAATGTTCTTGTTGGATGCAGGACTTGGGACCCCCATCATTTG
PB235_rps1                     AGGGGTAAAATGTTCTTGTTGGATGCAGGACTTGGGACCCCCATCATTTG
RRII105_rps1                   AGGGGTAAAATGTTCTTGTTGGATGCAGGACTTGGGACCCCCATCATTTG
BPM24_rps1                     AGGGGTAAAATGTTCTTGTTGGATGCAGGACTTGGGACCCCCATCATTTG
RRIM600_rps1                   AGGGGTAAAATGTTCTTGTTGGATGCAGGACTTGGGACCCCCATCATTTG
RRIT251_rps1                   AGGGGTAAAATGTTCTTGTTGGATGCAGGACTTGGGACCCCCATCATTTG
Citrullus lanatus              AGGGAAGAAATGTTCTTGGTGGATGCAGGACCTGGGACCCCCAGAATTTG
Cucurbita pepo                 AGGGAAGAAATGTTCTTGGTGGATGCAGGACCTGGGACCCCCAGAATTTG
Millettia pinnata              GGGGAAGAAATGTTCTTGGTGGATGCAGGACUTGGGACCCCCAGAATTTG
Oryza sativa Japonica          AGGGAAGAAATGTTCTTGGTGGATGCAGGCCTTGGTACCCCCAAAATTTG
                                ***   *********** ********** * *** *******  *****

RRIC110_rps1                   TATGCAAGATGAGCTTACAGGAGTGCCAAAAAACCGAGCCACCAGGTTTG
PB235_rps1                     TATGCAAGATGAGCTTACAGGAGTGCCAAAAAACCGAGCCACCAGGTTTG
RRII105_rps1                   TATGCAAGATGAGCTTACAGGAGTGCCAAAAAACCGAGCCACCAGGTTTG
BPM24_rps1                     TATGCAAGATGAGCTTACAGGAGTGCCAAAAAACCGAGCCACCAGGTTTG
RRIM600_rps1                   TATGCAAGATGAGCTTACAGGAGTGCCAAAAAACCGAGCCACCAGGTTTG
RRIT251_rps1                   TATGCAAGATGAGCTTACAGGAGTGCCAAAAAACCGAGCCACCAGGTTTG
Citrullus lanatus              TATGCAAGATGAGCCTACAGGAGTGCCAATCAACCGAGCCACCAGGTTTG
Cucurbita pepo                 TATGCAAGATGAGCCTACAGGAGTGCCAATCAACCGAGCCGCCAGGTTTG
Millettia pinnata              TATGCAAGATGAGCCTACAGGAGTGCCAATCAACCGAGCCACCAGGTTTG
Oryza sativa Japonica          TATGCAAGATGAGCTTACAGGACTGCCAATCAAGCGAGCCACCAGGTTTG
                               ************** ******* ******  ** ****** *********
```

```
RRIC110_rps1                      AGAATAAAGTGGGTTUCCTGGGTAAATCAATGATCAAAGAGCGAGCAGCC
PB235_rps1                        AGAATAAAGTGGGTTUCCTGGGTAAATCAATGATCAAAGAGCGAGCAGCC
RRII105_rps1                      AGAATAAAGTGGGTTUCCTGGGTAAATCAATGATCAAAGAGCGAGCAGCC
BPM24_rps1                        AGAATAAAGTGGGTTUCCTGGGTAAATCAATGATCAAAGAGCGAGCAGCC
RRIM600_rps1                      AGAATAAAGTGGGTTNCCTGGGTAAATCAATGATCAAAGAGCGAGCAGCC
RRIT251_rps1                      AGAATAAAGTGGGTTUCCTGGGTAAATCAATGATCAAAGAGCGAGCAGCC
Citrullus lanatus                 AGAATAAGGTGGGATUCCTGG-----------------------------
Cucurbita pepo                    AGAATAAGGTGGGATUCCTGG-----------------------------
Millettia pinnata                 AGAATAAGGTGGGATCCCTGG-----------------------------
Oryza sativa Japonica             AGAATAAGGTGGGATCCAAGA-----------------------------
                                  ******* ***** * *  *

RRIC110_rps1                      GCCAGGTTGAAGAAAAAGGATGTCGCCTGGTATTTTAAGAATAAAGTGAC
PB235_rps1                        GCCAGGTTGAAGAAAAAGGATGTCGCCTGGTATTTTAAGAATAAAGTGAC
RRII105_rps1                      GCCAGGTTGAAGAAAAAGGATGTCGCCTGGTATTTTAAGAATAAAGTGAC
BPM24_rps1                        GCCAGGTTGAAGAAAAAGGATGTCGCCTGGTATTTTAAGAATAAAGTGAC
RRIM600_rps1                      GCCAGGTTGAAGAAAAAGGATGTCGCCTGGTATTTTAAGAATAAAGTGAC
RRIT251_rps1                      GCCAGGTTGAAGAAAAAGGATGTCGCCTGGTATTTTAAGAATAAAGTGAC
Citrullus lanatus                 --------------------------------------------------
Cucurbita pepo                    --------------------------------------------------
Millettia pinnata                 --------------------------------------------------
Oryza sativa Japonica             --------------------------------------------------


RRIC110_rps1                      CGCCTGCTTTTTTAAGAATAAAGTGGCCGGTGAATCAATGATCAAAGAGC
PB235_rps1                        CGCCTGCTTTTTTAAGAATAAAGTGGCCGGTGAATCAATGATCAAAGAGC
RRII105_rps1                      CGCCTGCTTTTTTAAGAATAAAGTGGCCGGTGAATCAATGATCAAAGAGC
BPM24_rps1                        CGCCTGCTTTTTTAAGAATAAAGTGGCCGGTGAATCAATGATCAAAGAGC
RRIM600_rps1                      CGCCTGCTTTTTTAAGAATAAAGTGGCCGGTGAATCAATGATCAAAGAGC
RRIT251_rps1                      CGCCTGCTTTTTTAAGAATAAAGTGGCCGGTGAATCAATGATCAAAGAGC
Citrullus lanatus                 -----------------ATCTAGTGGCCGGTGAATCACTGATCAAAGATC
Cucurbita pepo                    -----------------ATCTAGTGGCCGGTGAATCACTGATCAAAGAGC
Millettia pinnata                 -----------------ATCTAGTGGCCGGTGAATCACTGATCAAAAAGA
Oryza sativa Japonica             -----------------ATGTAGTGGCTGGTGAATCACTGATCAAAAAGC
                                                   **  ****** ********* ******** *

RRIC110_rps1                      ATATTTTGGAGAGATTCTTCATCGATCTAGTGGCCGGTGAATCAATGATC
PB235_rps1                        ATATTTTGGAGAGATTCTTCATCGATCTAGTGGCCGGTGAATCAATGATC
RRII105_rps1                      ATATTTTGGAGAGATTCTTCATCGATCTAGTGGCCGGTGAATCAATGATC
BPM24_rps1                        ATATTTTGGAGAGATTCTTCATCGATCTAGTGGCCGGTGAATCAATGATC
RRIM600_rps1                      ATATTTTGGAGAGATTCTTCATCGATCTAGTGGCCGGTGAATCAATGATC
RRIT251_rps1                      ATATTTTGGAGAGATTCTTCATCGATCTAGTGGCCGGTGAATCAATGATC
Citrullus lanatus                 AAATTTTGGAGAGATTCTTCATCGATCTAGTGGCCGGCGAATCACTGATC
Cucurbita pepo                    CTATTTTGGAGAGATTCTTCATCGATCTAGTGGCCGGCGAATCACTGATC
Millettia pinnata                 AGATTTTGGAGAGATTATTCATCGATCTAGTGGCCGGCGAATCGCTGATC
Oryza sativa Japonica             GGATTGAAGAGAGATTCTTCATCGATCTAGTGGCCGGTGAATCACTGATC
                                    ***   ******** ******************** *****  *****

RRIC110_rps1                      AAAGAGCGAGCAGCCGCCAGGTTTAATGATTTGGTGGGATCCACAGATGT
PB235_rps1                        AAAGAGCGAGCAGCCGCCAGGTTTAATGATTTGGTGGGATCCACAGATGT
RRII105_rps1                      AAAGAGCGAGCAGCCGCCAGGTTTAATGATTTGGTGGGATCCACAGATGT
BPM24_rps1                        AAAGAGCGAGCAGCCGCCAGGTTTAATGATTTGGTGGGATCCACAGATGT
RRIM600_rps1                      AAAGAGCGAGCAGCCGCCAGGTTTAATGATTTGGTGGGATCCACAGATGT
RRIT251_rps1                      AAAGAGCGAGCAGCCGCCAGGTTTAATGATTTGGTGGGATCCACAGATGT
Citrullus lanatus                 AAAGAGCGAGCTGCCGCCAGGTTTAATGATTTGGTGGGATCCACAGATGT
Cucurbita pepo                    AAAGAGCGAGCAGCCGCCGGGTTTAATGATTTGGTGGGATCCACAGATGT
Millettia pinnata                 AAAGAGCGAGCAGCCGCCAGGTTTAATGATTTGGTGGGATCCACAGATGT
Oryza sativa Japonica             AAAGAGCGAGCAGCCGCCAGGTTTAATGATTTTGTGGGATUCCTGGATGT
                                  *********** ****** ************* ******* *   *****

RRIC110_rps1                      AGTGGCTGGTGAACCGC---TTCTTCTTCCACGAAGATTCATACAAATCC
```

```
PB235_rps1                      AGTGGCTGGTGAACCGC---TTCTTCTTCCACGAAGATTCATACAAATCC
RRII105_rps1                    AGTGGCTGGTGAACCGC---TTCTTCTTCCACGAAGATTCATACAAATCC
BPM24_rps1                      AGTGGCTGGTGAACCGC---TTCTTCTTCCACGAAGATTCATACAAATCC
RRIM600_rps1                    AGTGGCTGGTGAACCGC---TTCTTCTTCCACGAAGATTCATACAAATCC
RRIT251_rps1                    AGTGGCTGGTGAACCGC---TTCTTCTTCCACGAAGATTCATACAAATCC
Citrullus lanatus               AGTGGCTGGTGAACCGCTTTTTCTTCTTCCACGAAGATTCAGACAAAACC
Cucurbita pepo                  AGTGGCTGGTGAACCGCTTTTATTCTTCCACGAAGATTCAGACAAAACC
Millettia pinnata               AGTGGCTGGTGAACCGCTTCTTCTTCTTCCACGAAGATTCAGACAAAACC
Oryza sativa Japonica           AGCGGCTGGCGAACCGC---TTCTTCTTCCACAAAGATTCAGACAAAACC
                                ** ****** *******   ** ********* ******** ***** **

RRIC110_rps1                    GAGCTTGGATGGAACTGATAAAGATTTGGCGAACGAAAAAAAAGGTCAAA
PB235_rps1                      GAGCTTGGATGGAACTGATAAAGATTTGGCGAACGAAAAAAAAGGTCAAA
RRII105_rps1                    GAGCTTGGATGGAACTGATAAAGATTTGGCGAACGAAAAAAAAGGTCAAA
BPM24_rps1                      GAGCTTGGATGGAACTGATAAAGATTTGGCGAACGAAAAAAAAGGTCAAA
RRIM600_rps1                    GAGCTTGGATGGAACTGATAAAGATTTGGCGAACGAAAAAAAAGGTCAAA
RRIT251_rps1                    GAGCTTGGATGGAACTGATAAAGATTTGGCGAACGAAAAAAAAGGTCAAA
Citrullus lanatus               GAGCTTGGATGGAACTGAACAAGATTTGGCAAACGAATACAAAAGTCAAA
Cucurbita pepo                  GAGCTTGGATGGAACTGAACAAGATTTGGCAAACGAATACAAAAGTCAAA
Millettia pinnata               GAGCTTGGATGGAACTGAACAAGATTTGGCGAACGAATACAAAGGTAAAA
Oryza sativa Japonica           GAGCTTGGATAGAACTGAAGAAGATTTGGCAAACGAAGAAAAAGGTCAAA
                                ********** *******  ********** ****** * *** ** ***

RRIC110_rps1                    GGCTTTTATATTAAGAAAATCAAGGGAGGTTATTCAGTAGCCATCGCAGG
PB235_rps1                      GGCTTTTATATTAAGAAAATCAAGGGAGGTTATTCAGTAGCCATCGCAGG
RRII105_rps1                    GGCTTTTATATTAAGAAAATCAAGGGAGGTTATTCAGTAGCCATCGCAGG
BPM24_rps1                      GGCTTTTATATTAAGAAAATCAAGGGAGGTTATTCAGTAGCCATCGCAGG
RRIM600_rps1                    GGCTTTTATATTAAGAAAATCAAGGGAGGTTATTCAGTAGCCATCGCAGG
RRIT251_rps1                    GGCTTTTATATTAAGAAAATCAAGGGAGGTTATTCAGTAGCCATCGCAGG
Citrullus lanatus               GGCTTTATTATTGAGAAAGTCAAAGGAGGTTATTCAGTAGCCATCGCGGG
Cucurbita pepo                  GGCTTTATTATTGAGAAAGTCAAAGGAGGTTATTCAGTAGCCATCGCGGG
Millettia pinnata               GGCTTTATTCTTAATAAAGTAAAAGGAGGTTATTUAGTAGCCATCGCGGG
Oryza sativa Japonica           GGGTTTCGTATTAAAAAAATCAAAGGAGGTTATTUAGTAGCCATCGCAGG
                                ** ***  * ** * *** * ** ********** ************ **

RRIC110_rps1                    TTTCATTACTTTTCTTCCATTCCGTCCTCTCAA---------AAGGAAAG
PB235_rps1                      TTTCATTACTTTTCTTCCATTCCGTCCTCTCAA---------AAGGAAAG
RRII105_rps1                    TTTCATTACTTTTCTTCCATTCCGTCCTCTCAA---------AAGGAAAG
BPM24_rps1                      TTTCATTACTTTTCTTCCATTCCGTCCTCTCAA---------AAGGAAAG
RRIM600_rps1                    TTTCATTACTTTTCTTCCATTCCGTCCTCTCAA---------AAGGAAAG
RRIT251_rps1                    TTTCATTACTTTTCTTCCATTCCGTCCTCTCAA---------AAGGAAAG
Citrullus lanatus               TTTCATTACTTTTATTCCATTCCGTCCTCTCAT---------AAGGAAAA
Cucurbita pepo                  TTTCATTACTTTTCTTCCATTCCGGCCTCTCAT---------AAGTCAAA
Millettia pinnata               TTTCATTACTTTTCTTCCATTCCGTTCTCACAAGAAAAGAAGAAGGAAAA
Oryza sativa Japonica           TTTCATTACTTTTCTTCCATTCG---CTAAAAA---------AAAAAAAA
                                ************* ********    **   *          **   **

RRIC110_rps1                    GAACATGGAAAAAG-GAATCGCGAAGGCGATGGCGAAAGATAATGGAAAG
PB235_rps1                      GAACATGGAAAAAG-GAATCGCGAAGGCGATGGCGAAAGATAATGGAAAG
RRII105_rps1                    GAACATGGAAAAAG-GAATCGCGAAGGCGATGGCGAAAGATAATGGAAAG
BPM24_rps1                      GAACATGGAAAAAG-GAATCGCGAAGGCGATGGCGAAAGATAATGGAAAG
RRIM600_rps1                    GAACATGGAAAAAG-GAATCGCGAAGGCGATGGCGAAAGATAATGGAAAG
RRIT251_rps1                    GAACATGGAAAAAG-GAATCGCGAAGGCGATGGCGAAAGATAATGGAAAG
Citrullus lanatus               GGATATCGAATGATCGATTCACCATTGAGAGGGGAGAGAGCATTAACCCC
Cucurbita pepo                  GGATATCGAATGAGAGATTCACCATTGAGAGCATTAAC----CCAAAAAC
Millettia pinnata               AGATATCGAATGATCGATTCACCATTGAGAGCATTAAC----CCCAAAAG
Oryza sativa Japonica           GGATAGCGAATGATCGATTCACCATTGATAGCTTTAAC----CCTAAAAG
                                  * *  ***  *  ** ** * *  *  *      *

RRIC110_rps1                    GCGAAGGCAAATGGAAATGCAAAGGCGAGC-GC-TATTGAATGGTCGATT
PB235_rps1                      GCGAAGGCAAATGGAAATGCAAAGGCGAGC-GC-TATTGAATGGTCGATT
RRII105_rps1                    GCGAAGGCAAATGGAAATGCAAAGGCGAGC-GC-TATTGAATGGTCGATT
```

```
BPM24_rps1                        GCGAAGGCAAATGGAAATGCAAAGGCGAGC-GC-TATTGAATGGTCGATT
RRIM600_rps1                      GCGAAGGCAAATGGAAATGCAAAGGCGAGC-GC-TATTGAATGGTCGATT
RRIT251_rps1                      GCGAAGGCAAATGGAAATGCAAAGGCGAGC-GC-TATTGAATGGTCGATT
Citrullus lanatus                 AACGAATATTGTGGTGTTCTAAGAGTG----GCAGATCAAACAAGAACTT
Cucurbita pepo                    AACGAATATTGTGGTGTTCTAAGAGTGAGTGGCGGATCAAACAAGAACTT
Millettia pinnata                 GACGAATATTGTGGTGTTCTAATCTTTTGT-GCATCTTTCTCCCATGCTT
Oryza sativa Japonica             GAGGGATATTGTGAT-----AATAGCG----GCAGATCAAACAAGAACTT
                                             **      **         **   *           **

RRIC110_rps1                      CAACATCGAGCGCTATGATC--CCAAATGGATGAATATTGTGGTGTCGCT
PB235_rps1                        CAACATCGAGCGCTATGATC--CCAAATGGATGAATATTGTGGTGTCGCT
RRII105_rps1                      CAACATCGAGCGCTATGATC--CCAAATGGATGAATATTGTGGTGTCGCT
BPM24_rps1                        CAACATCGAGCGCTATGATC--CCAAATGGATGAATATTGTGGTGTCGCT
RRIM600_rps1                      CAACATCGAGCGCTATGATC--CCAAATGGATGAATATTGTGGTGTCGCT
RRIT251_rps1                      CAACATCGAGCGCTATGATC--CCAAATGGATGAATATTGTGGTGTCGCT
Citrullus lanatus                 GATGAGCGAAATCCGCTGACGAAAAAAAAAAGAGCTTTTTTTTAAACAGC
Cucurbita pepo                    GTCGGTCGAG--CTGCTGA--GCTCCGGAACCCATTTCTTCTTTCACTGT
Millettia pinnata                 TTCTTTGGTCAACAACCAA------GCACAACTATTTATAATGAATTCAT
Oryza sativa Japonica             GATGAAAAAAA-TAATAAATTATTAAAAATCCGAAGCCCACCTTAATCCA


RRIC110_rps1                      AUCAGCGGAAGATCAAAGAAGAACTTGA--------------------
PB235_rps1                        AUCAGCGGAAGATCAAAGAAGAACTTGA--------------------
RRII105_rps1                      AUCAGCGGAAGATCAAAGAAGAACTTGA--------------------
BPM24_rps1                        AUCAGCGGAAGATCAAAGAAGAACTTGA--------------------
RRIM600_rps1                      AUCAGCGGAAGATCAAAGAAGAACTTGA--------------------
RRIT251_rps1                      ANCAGCGGAAGATCAAAGAAGAACTTGA--------------------
Citrullus lanatus                 TCCTCTCCCTATCGGTCGAGCTGCTTTGTTCCTCTCCCTATCGGTCGA
Cucurbita pepo                    AACTGTAAACGAAGAA--------------------------------
Millettia pinnata                 CACTATAAAGGAGAAGTGAA----------------------------
Oryza sativa Japonica             TTTTGTTGAGGATCTTGCACTTATTCCGGCCC----------------
```

###################

rps4 multiple sequence alignment

```
Arabidopsis thaliana              ------------GGACTTAGAAAAGTCCTAAAATATCTTTCTGACCATGG
Brassica napus                    -----------GGGACTTAGAAAAGTCCTCAAATCTCTTTCTGACCATGG
Oryza sativa Japonica             -----------------------------------------ACGATATGT
RRIM600_rps4                      --------------------------------------------------
Citrullus lanatus                 -------------------------------CTTTGGCATAAAGATACCT
Cucurbita pepo                    ---------------------AAGGAAGGATCTTTGGCATAAAGATACCA
PB235_rps4                        --------------------------------------------------
RRIC110_rps4                      --------------------------------------------------
Lotus japonicus                   -------TCTGTCCTCCAAGACATCTCTCATGCCGTGC--CAAGGCAGCG
Millettia pinnata                 CTAAAGCGCTAACGCGCCTTATATTTTTAATTTTTTTTAGTAAAGCAAAG
BPM24_rps4                        --------------------------------------------------
RRIT251_rps4                      --------------------------------------------------
RRII105_rps4                      --------------------------------------------------
Beta macrocarpa                   --------------------------------------------------
Beta vulgaris vulgaris            --------------------------------------------------
Beta vulgaris maritima            --------------------------------------------------


Arabidopsis thaliana              TGTCAATAGCCCATACTTTCAGTCTTTTTTTAAAAAGACAACGTGAAGAG
Brassica napus                    TGTCAATAGCCCATACTTTCAGTCTTTTTT-AAAAAGACAACGTGAAGAG
Oryza sativa Japonica             AGTAGCTCATCCTAATTGAAGTATTTCATTCTGGCGAGCGTTCCTTTCTT
RRIM600_rps4                      --------------------------------------------------
Citrullus lanatus                 TCTTATTATTATTAGAGAAAGGGGCAAGCCAAAACCAACTCCTAAAAAGT
Cucurbita pepo                    GATTATTA--GAGAGAGAAAGGGGCAAGGCAAAACCAACTCCTAAAAAGT
PB235_rps4                        --------------------------------------------------
RRIC110_rps4                      --------------------------------------------------
Lotus japonicus                   AAACCTTTCGCCCGGCGAAAGCGGTAATCTCTTTCCCGTTCCCGGCTTTT
```

```
Millettia pinnata                 CAACCTTTCGCCGGGCGAAAGCGGGAATCTCTTTCCCGTTCCCGGCTTTT
BPM24_rps4                        --------------------------------------------------
RRIT251_rps4                      --------------------------------------------------
RRII105_rps4                      --------------------------------------------------
Beta macrocarpa                   ----------TCAATATCTGATCAGTCTCTGTTAGCAGCTTCAGTAGGAT
Beta vulgaris vulgaris            ----------TCAATATCTGATCAGTCTCTGTTAGCAGCTTCAGTAGGAT
Beta vulgaris maritima            ----------TCAATATCTGATCAGTCTCTGTTAGCAGCTTCAGTAGGAT


Arabidopsis thaliana              GAATAGATCAACAAAACAAAGAAAGGACAGGAACAGGGGTT----GTTGC
Brassica napus                    GAATAGATCAACAAAACAAAGAAAGGACAGGAACAGGGGTT----GTTGC
Oryza sativa Japonica             T---------------TCGGCGGCATCCTTCCGCATTGGCGGCGAGTGGA
RRIM600_rps4                      --------------------------------------------------
Citrullus lanatus                 GGCTGGATCGAAGTATCTCGAACATTCCCCATCCGCCCGCCGCCAGCTGC
Cucurbita pepo                    GG---AAT--AAGTATCTCGAAGATTCTCCATACGCCCGC---CAGCTGC
PB235_rps4                        --------------------------------------------------
RRIC110_rps4                      --------------------------------------------------
Lotus japonicus                   TAAGCTGTTAAACCAGCTTAGGAAAGATGAGAACTC---------GCAGC
Millettia pinnata                 T-----TTTAA----GCTTAGGAAAGATGAGAACTC---------GCAGC
BPM24_rps4                        --------------------------------------------------
RRIT251_rps4                      --------------------------------------------------
RRII105_rps4                      --------------------------------------------------
Beta macrocarpa                   TCAGGTCTTTTCTTTCTTTAAATAGAGGGGTCGCGG------------AA
Beta vulgaris vulgaris            TCAGGTCTTTTCTTTCTTTAAATAGAGGGGTCGCGG------------AA
Beta vulgaris maritima            TCAGGTCTTTTCTTTCTTTAAATAGAGGGGTCGCGG------------AA


Arabidopsis thaliana              AGAATTGGGTTCGAGTCCCAG-GGACGCAATGTGGCTGCTTAAAAAACTG
Brassica napus                    AGAATTGGGTTCGAGTCCCAG-GGACGCAATGTGGCTGCTTAAAAAACTG
Oryza sativa Japonica             AGAGTTGGGTTCGATTCCCTTTATACGCGATGTGGCGA---AAAAGACTG
RRIM600_rps4                      --------------------------------------------------
Citrullus lanatus                 AGAGG-GGGTTCGATTCCCGTTATACGCGATGTGGCGA---AAAAGACGG
Cucurbita pepo                    AGAGG-GGGTTCGATTCCCGTTATACGCGATGTGGC-A---AAAAAACGG
PB235_rps4                        --------------------------------------------------
RRIC110_rps4                      --------------------------------------------------
Lotus japonicus                   AGAGG-GGGTTCGATTCCCTTTATACGCGATGTGGCGA---ATCAGACTG
Millettia pinnata                 AGAGG-GGCTTCGATTCCCGTTATACGCGATGTGGCGA---ATCAGACTG
BPM24_rps4                        ----------------------------------------------ACGA
RRIT251_rps4                      ----------------------------------------------ACGA
RRII105_rps4                      --------------------------------------------------
Beta macrocarpa                   AGAATTGGGTTCGAGTCCCAT-AGCCCCGATGTGGCGA---AAAAGACTG
Beta vulgaris vulgaris            AGAATTGGGTTCGAGTCCCAT-AGCCCCGATGTGGCGA---AAAAGACTG
Beta vulgaris maritima            AGAATTGGGTTCGAGTCCCAT-AGCCCCGATGTGGCGA---AAAAGACTG


Arabidopsis thaliana              ATTCAA-CGA-GATATAGATTTGTCCCCATTAAGATTTCAAACTTGTCGT
Brassica napus                    ATTUAA-CGA-GATATAGATATGTCCCCATTAAGATTTCAAACTTGTCGT
Oryza sativa Japonica             ATTCAA-CGA-AATAT-GCCATGCCTGCATTAAGATTTAAAACGTGTCGT
RRIM600_rps4                      -----A-CGA-GATAT-GCCTTGCCTGCATTAAGATTTAAAACTTGTCGT
Citrullus lanatus                 ATTCAA-CAA-GATAT-GCCTTGCCTGCATTAAGATTTCAAACTTGTCGT
Cucurbita pepo                    ATTCAA-CAA-GATAT-GCCTTGCCTGCATTAAGATTTAAAACTTGTCGT
PB235_rps4                        -----A-CGA-GATAT-GCCTTGCCTGCATTAAGATTTAAAACTTGTCGT
RRIC110_rps4                      -----A-CGA-GATAT-GCCTTGCCTGCATTAAGATTTAAAACTTGTCGT
Lotus japonicus                   ATTCAA-CGACGACGA-GATATGC---------GATTTAAAACTTGTCGT
Millettia pinnata                 ATTCAA-CGACGACGA-GATATGC---------GATTTAAAACTTGTCGT
BPM24_rps4                        GATATG-CCTTGCCTG-CAT-TAA---------GATTTAAAACTTGTCGT
RRIT251_rps4                      GATATG-CCTTGCCTG-CAT-TAA---------GATTTAAAACTTGTCGT
RRII105_rps4                      -----A-CGA-GATAT-GCCTTGCCTGCATTAAGATTTAAAACTTGTCGT
Beta macrocarpa                   ACTGATTCAACAATAG-ATAATGACTGCATTAAGATTTAAAACTTGTCGT
Beta vulgaris vulgaris            ACTGATTCAACAATAG-ATAATGACTGCATTAAGATTTAAAACTTGTCGT
Beta vulgaris maritima            ACTGATTCAACAATAG-ATAATGACTGCATTAAGATTTAAAACTTGTCGT
                                         *             *           ***** **** ******
```

```
Arabidopsis thaliana          CTACTTTCAGGAAATGTTUGGAACAGAGAACTGACAATAATACAACGCCG
Brassica napus                CTACTTTUAGGAAATGTTUGGAACAGAGAACTGACAATAATACAACGTCG
Oryza sativa Japonica         CTACTTCUAGGAAATGTTUGGAACAGAGAACTTTCTCTAATCCAACGCCG
RRIM600_rps4                  CTACTTTCAGGAAATGTTCGGAAGAGAGAACTTACAATAATACAACGCCG
Citrullus lanatus             CTACTTTTAGGAAATGTTUGGAACAGAGAACTTACAATAATACAACGCCG
Cucurbita pepo                CTACTTTTAGGAAATGTTUGGAACAGAGAACTTACAATAATACAACGCCG
PB235_rps4                    CTACTTTCAGGAAATGTTUGGAAGAGAGAACTTACAATAATACAACGCCG
RRIC110_rps4                  CTACTTTCAGGAAATGTTUGGAAGAGAGAACTTACAATAATACAACGCCG
Lotus japonicus               CTACTTTCAGGAAATGTTUGGAACAGAGAACTTACAATAATACAACGCCG
Millettia pinnata             CTACTTTCAGGAAATGTTUGGAACAGAGAACTTACAATAATACAACGCCG
BPM24_rps4                    CTACTTTCAGGAAATGTTUGGAAGAGAGAACTTACAATAATACAACGCCG
RRIT251_rps4                  CTACTTTCAGGAAATGTTUGGAAGAGAGAACTTACAATAATACAACGCCG
RRII105_rps4                  CTACTTTCAGGAAATGTTUGGAAGAGAGAACTTACAATAATACAACGCCG
Beta macrocarpa               CTAGTTTCAGGAAATGTTUGGAATAAAGAACTTACACTATCGCAACGCAT
Beta vulgaris vulgaris        CTAGTTTCAGGAAATGTTUGGAATAAAGAACTTACACTATCGCAACGCAT
Beta vulgaris maritima        CTAGTTTCAGGAAATGTTUGGAATAAAGAACTTACACTATCGCAACGCAT
                              *** **  ********** **** * ******  *  **   *****

Arabidopsis thaliana          CATTCTCCGAAGATTGAGGAACAGGAAGAGATCTATTAAGAAGAGAAAGA
Brassica napus                CATTCTCCGAAGATTGAGGAACAGGAAGAGATCTATTAAGAAGAGAAAGA
Oryza sativa Japonica         CATTCTCCGAAGATTGAGGAACAAGAGGAGATCCATTAAAA---GAAATC
RRIM600_rps4                  CATTCTCCGAAGATTGAGGAACAAGAAGAAAGATATTAAGA---GAAAGA
Citrullus lanatus             CATTCTCCGAAAATTGAGAAACAAGAAGAGATCTATTAAGA---GAAAGA
Cucurbita pepo                CATTCTCCGAAAATTGAGAAACAAGAGGAGATCTATTAAGA---GAAAGA
PB235_rps4                    CATTCTCCGAAGATTGAGGAACAAGAAGAAAGATATTAAGA---GAAAGA
RRIC110_rps4                  CATTCTCCGAAGATTGAGGAACAAGAAGAAAGATATTAAGA---GAAAGA
Lotus japonicus               CATTCTCCGAAGATTGAGGAAGAAGAAGAGATCTATTAAGA---GAAAGA
Millettia pinnata             CATTCTCCGAAGATTGAGGAACAAGAAGAGATCTATTAAGA---GAAAGA
BPM24_rps4                    CATTCTCCGAAGATTGAGGAACAAGAAGAAAGATATTAAGA---GAAAGA
RRIT251_rps4                  CATTCTCCGAAGATTGAGGAACAAGAAGAAAGATATTAAGA---GAAAGA
RRII105_rps4                  CATTCTCCGAAGATTGAGGAACAAGAAGAAAGATATTAAGA---GAAAGA
Beta macrocarpa               CGCTCTCCGAAAATGGAGAATGAAGAAAAAATCTATTAAGA---AAAAGA
Beta vulgaris vulgaris        CGCTCTCCGAAAATGGAGAATGAAGAAAAAATCTATTAAGA---AAAAGA
Beta vulgaris maritima        CGCTCTCCGAAAATGGAGAATGAAGAAAAAATCTATTAAGA---AAAAGA
                              *  ******** ** *** *  * **  * *   ***** *    ***

Arabidopsis thaliana          TTTATCCGAAAAAATATCTTACCAGTTATATACAATTACAAACTACACGA
Brassica napus                TTTATUCGAAAAAATATCTTACCAGTTACATACAATTACAAACTACACGA
Oryza sativa Japonica         TTTCTCAGAGAGAAAATCTAAACAGTAACATCAAATUACAAACTACACGA
RRIM600_rps4                  TTTCTNCGAGAGAAAATCTTAACAGTTACATCCAATNACAAACTACACGA
Citrullus lanatus             TTTATUCGAGAGAAAATCTTAACAGTTACATCCAATUACAAACTACACGA
Cucurbita pepo                TTTATUCGAGAGAAAATCTTAACAGTTACATACAATUACAAACTACACGA
PB235_rps4                    TTTCTUCGAGAGAAAATCTTAACAGTTACATCCAATUACAAACTACACGA
RRIC110_rps4                  TTTCTUCGAGAGAAAATCTTAACAGTTACATCCAATUACAAACTACACGA
Lotus japonicus               TTTATCCGAGAGAAAATCTTAACAGTTACATCCAATUACAAACTACACGA
Millettia pinnata             TTTCTCCGAGAAAAAAATATGAACAGTTACATCCAATUACAAACTACACGA
BPM24_rps4                    TTTCTUCGAGAGAAAATCTTAACAGTTACATCCAATUACAAACTACACGA
RRIT251_rps4                  TTTCTUCGAGAGAAAATCTTAACAGTTACATCCAATCACAAACTACACGA
RRII105_rps4                  TTTCTNCGAGAGAAAATCTTAACAGTTACATCCAATNACAAACTACACGA
Beta macrocarpa               TGTATUCGAGAAAAAAATCGTAACAGTTACATACAATTACGAACTACACGA
Beta vulgaris vulgaris        TGTATUCGAGAAAAAAATCGTAACAGTTACATACAATTACGAACTACACGA
Beta vulgaris maritima        TGTATUCGAGAAAAAAATCGTAACAGTTACATACAATTACGAACTACACGA
                              * * *  ** * ** **   * **** * **  *** ** **********

Arabidopsis thaliana          AAGTTGCCCUTTTTTUATGGGGATTTACCCATCACAGAGATGCACAGAGG
Brassica napus                AAGTTGUCCCTTTTTUATGGGGATTTACCCATCACAGAGATGCACAGAGG
Oryza sativa Japonica         AAGTTGTCCCTTTATTATGGGGATTTACCCATAAGGGAGATGCACAGAGG
RRIM600_rps4                  AAGTTGCCCCTTTTTCATGGGGATTTACCCATCACAGAGATGCACAGAGG
Citrullus lanatus             AAGTTGUCCCTAAATUATGGGGATTTACCCATCACAGAGATGCACAGAGG
Cucurbita pepo                AAGTTGUCCCTTTTTUATGGGGATTTACCCATCACAGAGATGCACAGAGG
PB235_rps4                    AAGTTGCCCCTTTTTCATGGGGATTTACCCATCACAGAGATGCACAGAGG
```

```
RRIC110_rps4                     AAGTTGCCCCTTTTTCATGGGGATTTACCCATCACAGAGATGCACAGAGG
Lotus japonicus                  AAGTTGUCCCTTTTTUATGGGGATTTACCCATCACAGAGATGCACAGAGG
Millettia pinnata                AAGTTGUCCCTTTTTUATGGGGATTTACCCATCACAGAGATGCACAGAGG
BPM24_rps4                       AAGTTGCCCCTTTTTCATGGGGATTTACCCATCACAGAGATGCACAGAGG
RRIT251_rps4                     AAGTTGCCCCTTTTTCATGGGGATTTACCCATCACAGAGATGCACAGAGG
RRII105_rps4                     AAGTTGCCCCTTTTTCATGGGGATTTACCCATCACAGAGATGCACAGAGG
Beta macrocarpa                  AAGTTGTCCCTTTTTUATGGGAATTTACCCATCACAAAGATGCACAGAAG
Beta vulgaris vulgaris           AAGTTGTCCCTTTTTUATGGGAATTTACCCATCACAAAGATGCACAGAAG
Beta vulgaris maritima           AAGTTGTCCCTTTTTUATGGGAATTTACCCATCACAAAGATGCACAGAAG
                                 ****** ** *   * ***** ********** *   *********** *

Arabidopsis thaliana             AACAAAACGAACTTCATATATCCCTTTTCUACTCAATCUAGAAACAAGAT
Brassica napus                   AACAAAACGAACTTCATATATCCCTTTTCUACTCAATCUAGAAACAAGAT
Oryza sativa Japonica            AAGAGAACGAACTTCATATATCCCTTTTTTACTCAATCAAGAAACAAGAT
RRIM600_rps4                     AACAGAACGAACTTCATATATCCCTTTTCTACTTAATCCAGAAACAAGAT
Citrullus lanatus                AACAGAACGAACTTCTTATATCCCTTTTCUACTCAATCUAGAAACAAGAT
Cucurbita pepo                   AAGAGAACGAACTAATTATATCCCTTTTCUACTCAATCUAGAAACAAGAT
PB235_rps4                       AACAGAACGAACTTCATATATCCCTTTTCTACTTAATCCAGAAACAAGAT
RRIC110_rps4                     AACAGAACGAACTTCATATATCCCTTTTCTACTTAATCCAGAAACAAGAT
Lotus japonicus                  AACAGAACGAACTTCATATATCCCTTTTCTACTCAATCUAGAAACAAGAT
Millettia pinnata                AACAGAACGAACTTCATATATCCCTTTTCTACTCAATCUAGAAACAAGAT
BPM24_rps4                       AACAGAACGAACTTCATATATCCCTTTTCTACTTAATCCAGAAACAAGAT
RRIT251_rps4                     AACAGAACGAACTTCATATATCCCTTTTCTACTTAATCCAGAAACAAGAT
RRII105_rps4                     AACAGAACGAACTTCATATATCCCTTTTCTACTTAATCCAGAAACAAGAT
Beta macrocarpa                  AACAAAACGAACTTCATATATCCCTTTTCTACTCAATCUAGAAACAAGAT
Beta vulgaris vulgaris           AACAAAACGAACTTCATATATCCCTTTTCTACTCAATCUAGAAACAAGAT
Beta vulgaris maritima           AACAAAACGAACTTCATATATCCCTTTTCTACTCAATCUAGAAACAAGAT
                                 ** * ********   ************  *** **** ***********

Arabidopsis thaliana             TTGACGTTATTCCGCTTCGTCTCUATTTCTTGAAACTATTCCTCAAGCA
Brassica napus                   TTGACGTTATTCUGCTTCGTCTCCATTTCTTGAAACTATTCCTCAAGCA
Oryza sativa Japonica            UGGACGTGATTCUGGTTCGTCTCCATTTTTGTGACACTCTTCCTCAAGCA
RRIM600_rps4                     CGGACGTTATTCCGGTTCGTCTCCATTTTTGTGAAACTATTCCTCAAGCA
Citrullus lanatus                UGGACGTTATTCUGGTTCGTCTCCATTTTAGTGAAACTATTCCTCAAGCA
Cucurbita pepo                   UGGACGTTATTCUGGTTCGTCTCCATTTTAGTTCAACTCTTCCTCAAGCA
PB235_rps4                       CGGACGTTATTCCGGTTCGTCTCCATTTTTGTGAAACTATTCCTCAAGCA
RRIC110_rps4                     CGGACGTTATTCCGGTTCGTCTCCATTTTTGTGAAACTATTCCTCAAGCA
Lotus japonicus                  UGGACGTTATTCUGGTTCGTCTCCATTTTUGTGAAACTATTCCTCAAGCA
Millettia pinnata                UGGACGTTATTCUGGTTCGTCTCCATTTTUGTGAAACTATTCCTCAAGCA
BPM24_rps4                       CGGACGTTATTCCGGTTCGTCTCCATTTTTGTGAAACTATTCCTCAAGCA
RRIT251_rps4                     CGGACGTTATTCCGGTTCGTCTCCATTTTTGTGAAACTATTCCTCAAGCA
RRII105_rps4                     CGGACGTTATTCCGGTTCGTCTCCATTTTTGTGAAACTATTCCTCAAGCA
Beta macrocarpa                  TGGACGTTATTCTGGTTCGTCTACATTTTTGTGAAACTATTCCTCAAGCA
Beta vulgaris vulgaris           TGGACGTTATTCTGGTTCGTCTACATTTTTGTGAAACTATTCCTCAAGCA
Beta vulgaris maritima           TGGACGTTATTCTGGTTCGTCTACATTTTTGTGAAACTATTCCTCAAGCA
                                   ***** **** * *******  *****  *   *** ***********

Arabidopsis thaliana             AGGCAGCUGATAAGTCATCGAAGGGTTTGTGTGAATAAAGGAATGGTAAG
Brassica napus                   AGGCAGCUGATAAGTCATCGAAGGGTTTGTGTGAATAAAGGAATGGTAAG
Oryza sativa Japonica            AGGCAGCUGATAAGTCATCGAAGGGTTTGTTTGAATAATGGACTGGTAAC
RRIM600_rps4                     AGGCAGCCGATAAGTCATCGAAGGGTTTGTGTGAATAATGGAATGGTAAG
Citrullus lanatus                AGGCAGCUGATAAGTCATCGAAGGGTTTGTGTGAATAAAGGAATGGTAAG
Cucurbita pepo                   AGGCAGCUGATAAGTCATCGAAGGGTTTGTGTGAATAAAGGAATGGTCCC
PB235_rps4                       AGGCAGCCGATAAGTCATCGAAGGGTTTGTGTGAATAATGGAATGGTAAG
RRIC110_rps4                     AGGCAGCCGATAAGTCATCGAAGGGTTTGTGTGAATAATGGAATGGTAAG
Lotus japonicus                  AGGCAGCUGATAAGTCATCGAAGGGTTTGTGTGAATAATCGAATGGTAAG
Millettia pinnata                AGGCAGCUGATAAGTCATCGAAGGGTTTGTGTTAATAATCGAATGGTAAG
BPM24_rps4                       AGGCAGCCGATAAGTCATCGAAGGGTTTGTGTGAATAATGGAATGGTAAG
RRIT251_rps4                     AGGCAGCCGATAAGTCATCGAAGGGTTTGTGTGAATAATGGAATGGTAAG
RRII105_rps4                     AGGCAGCCGATAAGTCATCGAAGGGTTTGTGTGAATAATGGAATGGTAAG
Beta macrocarpa                  AGGCAGCUGATAAGTCATCGAAAAGTGTGTGTGAATAATGGAATAGTAAA
Beta vulgaris vulgaris           AGGCAGCUGATAAGTCATCGAAAAGTGTGTGTGAATAATGGAATAGTAAA
```

```
Beta vulgaris maritima           AGGCAGCUGATAAGTCATCGAAAAGTGTGTGTGAATAATGGAATAGTAAA
                                 ******* **************  ** *** * *****  ** * **

Arabidopsis thaliana             CATTACTCATTTTAAACTTTCCCACGGTGATATAATATCTTTTCAAGAAA
Brassica napus                   CATTACTCATTTGAAACTTTCCCACGGTGATATAATATCTTTTCAAGAAA
Oryza sativa Japonica            CATTACTCATTTGAAAGTTTCCCACGGTGATCTAATATCTTTTCAAGAAA
RRIM600_rps4                     CATTACTCATTTGAAAGTGTCCCACGGTGATCTAATATCTTTTCAAGAAA
Citrullus lanatus                CATTACTCATTTTAAAGTTTCCCACGGTGATCTAATATCTTTTCAAGAAA
Cucurbita pepo                   GATTACTCATTTTAAAGTGTCCCACGGTGATCTAATATCTTTGAAAGAAA
PB235_rps4                       CATTACTCATTTGAAAGTGTCCCACGGTGATCTAATATCTTTTCAAGAAA
RRIC110_rps4                     CATTACTCATTTGAAAGTGTCCCACGGTGATCTAATATCTTTTCAAGAAA
Lotus japonicus                  CATTACTCGTTTGAAAGTTTCCCACGGTGATCTAATCTCTTTTCAAGAAA
Millettia pinnata                CATTACTCGTTTGAAAGTTTCCCACGGTGATCTAATATCTTTTCAAGAAA
BPM24_rps4                       CATTACTCATTTGAAAGTGTCCCACGGTGATCTAATATCTTTTCAAGAAA
RRIT251_rps4                     CATTACTCATTTGAAAGTGTCCCACGGTGATCTAATATCTTTTCAAGAAA
RRII105_rps4                     CATTACTCATTTGAAAGTGTCCCACGGTGATCTAATATCTTTTCAAGAAA
Beta macrocarpa                  CATTAGTCATTTTAAAGTTTCCCACGGTGATATAATATCTTTTCAAGAAA
Beta vulgaris vulgaris           CATTAGTCATTTTAAAGTTTCCCACGGTGATATAATATCTTTTCAAGAAA
Beta vulgaris maritima           CATTAGTCATTTTAAAGTTTCCCACGGTGATATAATATCTTTTCAAGAAA
                                  **** ** *** *** * ************ **** *****  ******

Arabidopsis thaliana             ATAACGCGATAATACGCGGTGAAGAAATAAGGAGATCTTTCTATAAAGAA
Brassica napus                   ATAACGCGATAATACGCGGTGAAGAAATAAGGAGATCTTTCTATAAAGAA
Oryza sativa Japonica            ATGACGCGAGAACCCGCGGTGAAGAAATAAGGAGATCTTTCTATATCGAC
RRIM600_rps4                     ATGACGCAAGAATCCGCGGTGAAGAAATAAGGAGATCTTTCTATATCGAA
Citrullus lanatus                ATGACGCGAGAATCCGCGGTGAAGAAATAAGGAGATUTTTCTATATCGAA
Cucurbita pepo                   ATGACGCGAGAATCCGCGGTGAAGAAATAAGGAGATCTTTCTATATCGAA
PB235_rps4                       ATGACGCAAGAATCCGCGGTGAAGAAATAAGGAGATCTTTCTATATCGAA
RRIC110_rps4                     ATGACGCAAGAATCCGCGGTGAAGAAATAAGGAGATCTTTCTATATCGAA
Lotus japonicus                  ATGACGCGAGAATCCGCGGTGAAGAAATAAGGAGATCTTTCTATATCGAA
Millettia pinnata                ATGACGCGAGAATCCGCGGTGAAGAAATAAGGAGAGCTTTCTATATCGAA
BPM24_rps4                       ATGACGCAAGAATCCGCGGTGAAGAAATAAGGAGATCTTTCTATATCGAA
RRIT251_rps4                     ATGACGCAAGAATCCGCGGTGAAGAAATAAGGAGATCTTTCTATATCGAA
RRII105_rps4                     ATGACGCAAGAATCCGCGGTGAAGAAATAAGGAGATCTTTCTATATCGAA
Beta macrocarpa                  ATTACGCGAGAACCCGCGGTGAAGAAATAAGGAAATTTTTUTATATCAAA
Beta vulgaris vulgaris           ATTACGCGAGAACCCGCGGTGAAGAAATAAGGAAATTTTTUTATATCAAA
Beta vulgaris maritima           ATTACGCGAGAACCCGCGGTGAAGAAATAAGGAAATTTTTUTATATCAAA
                                 ** **** * **  ******************* *   *** ****   *

Arabidopsis thaliana             ATTTUAGTTGAAAAAATCATAGGCAAATTACTGCATCAACCGC-------
Brassica napus                   ATTTUAGTTGAAAAAATCATAGGCAAATTACTGCATCAACCGC-------
Oryza sativa Japonica            ATATUAGTTGGAAAAATCATAGGCAAATTACTA------CCGG-------
RRIM600_rps4                     ATATNAGTTGAAAAAATCATAGGCAAATTCCTGGATCACCCGG-------
Citrullus lanatus                ATATUAGTTGAAAAAATCATAGGAAAATTCCTGGATCACCCGG-------
Cucurbita pepo                   ATCTCAGTTGAAAAAATCATAGGCAAATTCCTAGATCACCCGG-------
PB235_rps4                       ATATUAGTTGAAAAAATCATAGGCAAATTCCTGGATCACCCGG-------
RRIC110_rps4                     ATATNAGTTGAAAAAATCATAGGCAAATTCCTGGATCACCCGG-------
Lotus japonicus                  ATATUAGTTGAAAAAATAATAGGCAAATTCCTGGATCACCCGG-------
Millettia pinnata                ATATUAGTTGATAAAATCATAGGCAAATTCCTGGATCACCCGG-------
BPM24_rps4                       ATATUAGTTGAAAAAATCATAGGCAAATTCCTGGATCACCCGG-------
RRIT251_rps4                     ATATUAGTTGAAAAAATCATAGGCAAATTCCTGGATCACCCGG-------
RRII105_rps4                     ATATNAGTTGAAAAAATCATAGGCAAATTCCTGGATCACCCGG-------
Beta macrocarpa                  ATATUAGTTGAAAAAATCATAGGCAAATTAAGGGGTCACCGGGGAAGACT
Beta vulgaris vulgaris           ATATUAGTTGAAAAAATCATAGGCAAATTAAGGGGTCACCGGGGAAGACT
Beta vulgaris maritima           ATATUAGTTGAAAAAATCATAGGCAAATTAAGGGGTCACCGGGGAAGACT
                                 ** * *****  ***** ***** *****          * *

Arabidopsis thaliana             -----------------------------------TAAGAATGTGGAGAA
Brassica napus                   -----------------------------------TAAGAATGTGGAGAA
Oryza sativa Japonica            -----------------------------------TCAGAATCTGGAGAA
RRIM600_rps4                     -----------------------------------TAAGAATGTGGAGAA
Citrullus lanatus                -----------------------------------TAAGAATGTGGAGAA
```

```
Cucurbita pepo                  -----------------------------------TAAGAATGTGGAGAA
PB235_rps4                      -----------------------------------TAAGAATGTGGAGAA
RRIC110_rps4                    -----------------------------------TAAGAATGTGGAGAA
Lotus japonicus                 -----------------------------------TAAGAATGTGGAGAA
Millettia pinnata               -----------------------------------TAAGAATGTGGAGAA
BPM24_rps4                      -----------------------------------TAAGAATGTGGAGAA
RRIT251_rps4                    -----------------------------------TAAGAATGTGGAGAA
RRII105_rps4                    -----------------------------------TAAGAATGTGGAGAA
Beta macrocarpa                 GCGGAGCAGAAGCAAAACAGAATGGTTCCGCCGAGTAAGAATGCGGAGCA
Beta vulgaris vulgaris          GCGGAGCAGAAGCAAAACAGAATGGTTCCGCCGAGTAAGAATGCGGAGCA
Beta vulgaris maritima          GCGGAGCAGAAGCAAAACAGAATGGTTCCGCCGAGTAAGAATGCGGAGCA
                                                                   * *****  **** *

Arabidopsis thaliana            GAAGCAAAACTGAATGGTTCCACCTACTCAAAACTAAGAGGGGATGCCGC
Brassica napus                  GAAGCAAAACTGAATGGTTCCACCTACTCAAAACTAAGAGGGGATGCCGC
Oryza sativa Japonica           AAACAAAAACAGAATGGTTCCGCTTACTCACAACTCAGAGGGTATGCCGC
RRIM600_rps4                    GAACCAAAACAGAATGGTTCCACCTACTCAAAACTAAGAGGGGATGCCGC
Citrullus lanatus               GAACTAAAACAGAATGGTTCCACCTACTCAAAACTAAGAGGGGATGCCGC
Cucurbita pepo                  GAACTAAAACAGAATGGTTCCACCTACTCAAAACTAAGAGGGGATGCCGC
PB235_rps4                      GAACCAAAACAGAATGGTTCCACCTACTCAAAACTAAGAGGGGATGCCGC
RRIC110_rps4                    GAACCAAAACAGAATGGTTCCACCTACTCAAAACTAAGAGGGGATGCCGC
Lotus japonicus                 GAACCAAAACAGAATGGTTCCACCTACTAAAAACTAAGCGGGGATGCCGC
Millettia pinnata               GAACCAAAACAGAATGGTTCCACCTACTCAAAACTAAGCGGGGATGCCGC
BPM24_rps4                      GAACCAAAACAGAATGGTTCCACCTACTCAAAACTAAGAGGGGATGCCGC
RRIT251_rps4                    GAACCAAAACAGAATGGTTCCACCTACTCAAAACTAAGAGGGGATGCCGC
RRII105_rps4                    GAACCAAAACAGAATGGTTCCACCTACTCAAAACTAAGAGGGGATGCCGC
Beta macrocarpa                 GAAGCAAAACAGAATGGTTCCGCCGACTCAAAACTAAGAAGGGATGCCGC
Beta vulgaris vulgaris          GAAGCAAAACAGAATGGTTCCGCCGACTCAAAACTAAGAAGGGATGCCGC
Beta vulgaris maritima          GAAGCAAAACAGAATGGTTCCGCCGACTCAAAACTAAGAAGGGATGCCGC
                                 **  ***** ********** *  *** * **** **  ** *******

Arabidopsis thaliana            CTACTACTAAAATCCCGGTTTTTGCAA---CAGTTGCGTTCTTCTATGCA
Brassica napus                  CTACTACTAAAATCCCGGTTTTTGCAA---CAGTTGCGTTCTTCTATGCA
Oryza sativa Japonica           TTACTACTCAAATCCTGGTTTTTGCAA---GAGTTGCGTTCTTATATGCA
RRIM600_rps4                    CTACTACTAAAATCCCGGTTTTTGCAA---CAGTTGCGTTCTTCTATGCA
Citrullus lanatus               CTACTACTAAAATCCCGGTTTTTGCAA---CAGTTGCGTTCTTCTATGCA
Cucurbita pepo                  CTACTACTAAAATCCAGGTTTTTGCAA---CAGTTGCGTTCTTCTATGCA
PB235_rps4                      CTACTACTAAAATCCCGGTTTTTGCAA---CAGTTGCGTTCTTCTATGCA
RRIC110_rps4                    CTACTACTAAAATCCCGGTTTTTGCAA---CAGTTGCGTTCTTCTATGCA
Lotus japonicus                 CTACTACTAAAATCCCGGTTTTTGCAACAACAGTTGCGTTATTCTATGCA
Millettia pinnata               CTACTACTAAAATCCCGGTTTTTGCAAAAACAGTTGCGTTATTCTATGCA
BPM24_rps4                      CTACTACTAAAATCCCGGTTTTTGCAA---CAGTTGCGTTCTTCTATGCA
RRIT251_rps4                    CTACTACTAAAATCCCGGTTTTTGCAA---CAGTTGCGTTCTTCTATGCA
RRII105_rps4                    CTACTACTAAAATCCCGGTTTTTGCAA---CAGTTGCGTTCTTCTATGCA
Beta macrocarpa                 CTACTACTAAAAGACCGGTTTTTGCAA---AAGTTACATTCTTCTATGCA
Beta vulgaris vulgaris          CTACTACTAAAAGACCGGTTTTTGCAA---AAGTTACATTCTTCTATGCA
Beta vulgaris maritima          CTACTACTAAAAGACCGGTTTTTGCAA---AAGTTACATTCTTCTATGCA
                                 ******* ***  * ***********    **** * ** ** ******

Arabidopsis thaliana            AGAAGAAGACTTAGAAAGAACAAAGAAGTT---TGGATCCGAAAAAGTAT
Brassica napus                  AGAAGAAGACTTAGAAAGAACAAAGAAGTT---TGGATCCGAAAAAGTAT
Oryza sativa Japonica           AGAAGAAGACTTAGAAAGAACAAAGAAGTT---TGGATCCGCAAAAGTAT
RRIM600_rps4                    AGAAGAAGACTTAGAAAGAACAAAGAAGTT---TGGATCCGAAAAAGTAT
Citrullus lanatus               AGAAGAAGACTTAGAAAGAACAAAGAAGTT---TGGATUGGAAAAAGTAT
Cucurbita pepo                  AGAATCAGACTTAGAAAGAAGAAAGAAGTTGTTTGGATCGGATAAAGTAT
PB235_rps4                      AGAAGAAGACTTAGAAAGAACAAAGAAGTT---TGGATCCGAAAAAGTAT
RRIC110_rps4                    AGAAGAAGACTTAGAAAGAACAAAGAAGTT---TGGATCCGAAAAAGTAT
Lotus japonicus                 AGAAGAAGACTTTGAAAGAACAAAGAAGTT---TGGATCCGAAAAAGTAT
Millettia pinnata               AAAAGAATACTTAGAAAGAACAAAGAAGTT---TGGATCCGAAAAAGTAT
BPM24_rps4                      AGAAGAAGACTTAGAAAGAACAAAGAAGTT---TGGATCCGAAAAAGTAT
RRIT251_rps4                    AGAAGAAGACTTAGAAAGAACAAAGAAGTT---TGGATCCGAAAAAGTAT
RRII105_rps4                    AGAAGAAGACTTAGAAAGAACAAAGAAGTT---TGGATCCGAAAAAGTAT
```

```
Beta macrocarpa                  AGAACAAGACTTAGAAAGAACAAAGAAGTT---TGGATCCGA--------
Beta vulgaris vulgaris           AGAACAAGACTTAGAAAGAACAAAGAAGTT---TGGATCCGA--------
Beta vulgaris maritima           AGAACAAGACTTAGAAAGAACAAAGAAGTT---TGGATCCGA--------
                                 * **  * **** ******* *********   *****  *

Arabidopsis thaliana             GCTTAGGAAGTTCCTTCGCTGAGCACAAGAGAATGAAGAGGAATTTGT--
Brassica napus                   GCTTAGGAAGTTCCTTCGCTGAGCACAAGAGAATGAAGAGGAATTTGT--
Oryza sativa Japonica            GCTTAGGCAGTTCCTTCGCTGAGCACAACAGAATGAAGAGGAATTTGTTT
RRIM600_rps4                     GCTTAGGCAGTTCCTTCGCTGAGCACAACAGAATGAAGAGAAATTTGTAT
Citrullus lanatus                GCTTAGGCAGTTCCTTCGCTGAGCACAACAGAATGAAGAGGAATTTGTAT
Cucurbita pepo                   GCTTAGGCTGTTCCTTCGCTGAGCACAACAGAATGAAGAGGAATTTGTAT
PB235_rps4                       GCTTAGGCAGTTCCTTCGCTGAGCACAACAGAATGAAGAGAAATTTGTAT
RRIC110_rps4                     GCTTAGGCAGTTCCTTCGCTGAGCACAACAGAATGAAGAGAAATTTGTAT
Lotus japonicus                  GCTTAGGCAGTTCCTTCGCTGAGCACAACAGAATGAAGAGGAATTTGTAT
Millettia pinnata                GCTTAGGCAGTTCCTTCGCTGAGCACAACAGAATGAAGAGGAATTTGTAT
BPM24_rps4                       GCTTAGGCAGTTCCTTCGCTGAGCACAACAGAATGAAGAGAAATTTGTAT
RRIT251_rps4                     GCTTAGGCAGTTCCTTCGCTGAGCACAACAGAATGAAGAGAAATTTGTAT
RRII105_rps4                     GCTTAGGCAGTTCCTTCGCTGAGCACAACAGAATGAAGAGAAATTTGTAT
Beta macrocarpa                  ----------------CGCTGAGCACAACAGAATGAAGAGGAATTTTTAT
Beta vulgaris vulgaris           ----------------CGCTGAGCACAACAGAATGAAGAGGAATTTTTAT
Beta vulgaris maritima           ----------------CGCTGAGCACAACAGAATGAAGAGGAATTTTTCT
                                                 ************ *********** ***** *

Arabidopsis thaliana             ----TAAAATCCCTATTCTTATCGAAGAGAAGGAAGGATAAA----AACC
Brassica napus                   ----TAAAATCCCTATTCTTATCGAAGAGAAGGAAGGAGAAA----AACC
Oryza sativa Japonica            CATTTCAAATACTTCTTCTTATTGAAAAGAAGGAAGGAGGAAGAGGAATT
RRIM600_rps4                     CATTTCAAATCCCTATTCTTATCGAAGAAAAGGAACGAGAAA----AACC
Citrullus lanatus                CATTTGAAATCCCTATTCTTATCGAATAGAAGGAACGAGAAA----AACC
Cucurbita pepo                   CATTTTCAATCCCTATTCTTATCGAATAGAAGGAAGGAGAAA----AACC
PB235_rps4                       CATTTCAAATCCCTATTCTTATCGAAGAAAAGGAACGAGAAA----AACC
RRIC110_rps4                     CATTTCAAATCCCTATTCTTATCGAAGAAAAGGAACGAGAAA----AACC
Lotus japonicus                  CATTTCAAATCCCTATTCTTATCGAAGAGAAGAAACGAGAAA----AACC
Millettia pinnata                CATTTCAAATCCCTATTCTTATCGAAGAGAAGAAACGAGAAA----AACC
BPM24_rps4                       CATTTCAAATCCCTATTCTTATCGAAGAAAAGGAACGAGAAA----AACC
RRIT251_rps4                     CATTTCAAATCCCTATTCTTATCGAAGAAAAGGAACGAGAAA----AACC
RRII105_rps4                     CATTTCAAATCCCTATTCTTATCGAAGAAAAGGAACGAGAAA----AACC
Beta macrocarpa                  CACTCCGTGACCTTATTCTTATCGAA------------------------
Beta vulgaris vulgaris           CACTCCGTGACCTTATTCTTATCGAA------------------------
Beta vulgaris maritima           CACTCCGTGACCTTATTCTTATCGAA------------------------
                                            * * ******* ***

Arabidopsis thaliana             TAA--------------------ATCTTCCTACTCGAACAATCAGTCCTA
Brassica napus                   TAA--------------------ATCTTCCTACTCGAACAATCAGTCCTA
Oryza sativa Japonica            GATAAGAACAATAGGGGAAGCGGAAAACCGAAAAAGAGCAATAAGTCCTT
RRIM600_rps4                     GA--------------------AATCTTCCTACTCGAATAAGAAGTCCTA
Citrullus lanatus                GAA--------------------ATCTTCCTACTCGAACAAGAAGTCCTA
Cucurbita pepo                   GAA--------------------ATCTTCCTACTCGAAGAATCAGTCCTA
PB235_rps4                       GAA--------------------ATCTTCCTACTCGAATAAGAAGTCCTA
RRIC110_rps4                     GAA--------------------ATCTTCCTACTCGAATAAGAAGTCCTA
Lotus japonicus                  GAT--------------------ATCTTCCTACTCGAACAAGAAGTCCTA
Millettia pinnata                GAT--------------------ATCTTCCTACTCGAACAAGAAGTCCTA
BPM24_rps4                       GAA--------------------ATCTTCCTACTCGAATAAGAAGTCCTA
RRIT251_rps4                     GAA--------------------ATCTTCCTACTCGAATAAGAAGTCCTA
RRII105_rps4                     GAA--------------------ATCTTCCTACTCGAATAAGAAGTCCTA
Beta macrocarpa                  --------------------------------------------------
Beta vulgaris vulgaris           --------------------------------------------------
Beta vulgaris maritima           --------------------------------------------------


Arabidopsis thaliana             TAGTTTACAACTCTTCTTTATCTTTATATAGTAATTCGACCTATTGCTTC
Brassica napus                   TAGTTTACAACTCTTCTTTATCTTTATATAGTAATTCGACCTATTGCTTC
Oryza sativa Japonica            TTGTTTACAAGTCTTCTTTAT------ATAGAAATTCGACCTATTGCTCU
```

```
RRIM600_rps4                      TAGTTTACAACTCTTCTTTAT------ATAGTAATTCGATCTATTGCTCC
Citrullus lanatus                 TAGTTTACAACTCTTCTTTAT------ATAGTAATTCGACCTATTGCTCC
Cucurbita pepo                    TAGTTTACAACTCTTCCTTAT------ATAGTAATTATACCTATTGCTCC
PB235_rps4                        TAGTTTACAACTCTTCTTTAT------ATAGTAATTCGATCTATTGCTCC
RRIC110_rps4                      TAGTTTACAACTCTTCTTTAT------ATAGTAATTCGATCTATTGCTCC
Lotus japonicus                   TAGTTTACAACTCTTCTTTAT------ATAGTAATTCGACCTATTGCTCC
Millettia pinnata                 TAGTTGACTACTCTTCTTTAT------ATAGTAATTCGACCTATTGCTCC
BPM24_rps4                        TAGTTTACAACTCTTCTTTAT------ATAGTAATTCGATCTATTGCTCC
RRIT251_rps4                      TAGTTTACAACTCTTCTTTAT------ATAGTAATTCGATCTATTGCTCC
RRII105_rps4                      TAGTTTACAACTCTTCTTTAT------ATAGTAATTCGATCTATTGCTCC
Beta macrocarpa                   --------------------------------------------------
Beta vulgaris vulgaris            --------------------------------------------------
Beta vulgaris maritima            --------------------------------------------------


Arabidopsis thaliana              GCATCCCCCCATAAGTTGACTATGAAGAGAAGAATCAAAAGGATCGAACT
Brassica napus                    GCATCCCCCCATAAGTTGACTATGAAGAGAAGAATCAAAAGGATUGAACT
Oryza sativa Japonica             GGATCCCC------GTTTACTAGGAAGATAAGAATCAAAAGGATUGAACT
RRIM600_rps4                      GCATCCCCCTATCAGTTTACTATGAAGAGAAGAATCAAAAGGATCGAACT
Citrullus lanatus                 GCATCCCCCCATCAGTTTACTATGAAGAGCAAAATCAAAAGGATCGAACT
Cucurbita pepo                    GCATCCCCCCATCAGTTTACTATGAAGAGCAAAATCAAAAGGATCGAACT
PB235_rps4                        GCATCCCCCTATCAGTTTACTATGAAGAGAAGAATCAAAAGGATCGAACT
RRIC110_rps4                      GCATCCCCCTATCAGTTTACTATGAAGAGAAGAATCAAAAGGATCGAACT
Lotus japonicus                   TCATCCCCCCATCAGTTTACTATGAAGAGAAGAATUAAAAGGATCGAACT
Millettia pinnata                 TCATCCCCCCATCAGTTTACTATGAAGAGAAGAATUAAAAGGATCGAACT
BPM24_rps4                        GCATCCCCCTATCAGTTTACTATGAAGAGAAGAATCAAAAGGATCGAACT
RRIT251_rps4                      GCATCCCCCTATCAGTTTACTATGAAGAGAAGAATCAAAAGGATCGAACT
RRII105_rps4                      GCATCCCCCTATCAGTTTACTATGAAGAGAAGAATCAAAAGGATCGAACT
Beta macrocarpa                   -----------------------GAAGAGAAGAATCAAAAGGATAGAACT
Beta vulgaris vulgaris            -----------------------GAAGAGAAGAATCAAAAGGATAGAACT
Beta vulgaris maritima            -----------------------GAAGAGAAGAATCAAAAGGATAGAACT
                                                         *****  * *** ******** *****

Arabidopsis thaliana              ACCTACTCATTATTUGGAGGTTAATUATAGAACACCAAAAGCTGTGGTAT
Brassica napus                    ACCTACTCATTATTUGGAGGTTAATUATAGAACACCAAAAGCTGTGGTAT
Oryza sativa Japonica             ACCTACTCATTATTUGGAGGTGAATUATAGAACACTAAAAGCTGTGGTAT
RRIM600_rps4                      ACCTACTCATTATTNGGAGGTGAATCATAGAACACTAAAAGCTGTGGTAT
Citrullus lanatus                 ACCTACTCATTATTUGGAGGTGAATUATAGAACACTAAAAGCTGTGGTAT
Cucurbita pepo                    ACCTACTCATTATTUGGAGGTGAATUATAGAACACTAAAAGCTGTGGTAT
PB235_rps4                        ACCTACTCATTATTUGGAGGTGAATCATAGAACACTAAAAGCTGTGGTAT
RRIC110_rps4                      ACCTACTCATTATTNGGAGGTGAATCATAGAACACTAAAAGCTGTGGTAT
Lotus japonicus                   ACCTACCCATTATTUGGAGGTGAATUATAGAACACUAAAAGCGGTGGTAT
Millettia pinnata                 ACCTACTCATTATTUGGAGGTGAATUATAGAACACUAAAAGCGGTGGTAT
BPM24_rps4                        ACCTACTCATTATTUGGAGGTGAATCATAGAACACTAAAAGCTGTGGTAT
RRIT251_rps4                      ACCTACTCATTATTUGGAGGTGAATCATAGAACACTAAAAGCTGTGGTAT
RRII105_rps4                      ACCTACTCATTATTNGGAGGTGAATCATAGAACACTAAAAGCTGTGGTAT
Beta macrocarpa                   ACCTACTCATTATTCGGAGGTCAATCATAGAACACTAAAAGCTGTGGTAT
Beta vulgaris vulgaris            ACCTACTCATTATTCGGAGGTCAATCATAGAACACTAAAAGCTGTGGTAT
Beta vulgaris maritima            ACCTACTCATTATTCGGAGGTCAATCATAGAACACTAAAAGCTGTGGTAT
                                  ****** ******* ****** *** ********* ****** *******

Arabidopsis thaliana              UTTATGGACCTAACATAGGTCATATCCCTCACGACATAAGATTAAAAGAT
Brassica napus                    UTTATGGACCTAACATAGGTCATATCCCTCACGACATAAGATTAAAAGAT
Oryza sativa Japonica             UTTATGGACCTAACATAGGTCACATCCCTCACGACATAAGATTGAAAGAT
RRIM600_rps4                      CTTATGGACCGAACATAGGTCATATCCCTCACGACATAAGATTGAAAGAT
Citrullus lanatus                 UTTATGGACCTAACATAGGTCACATCCCTCACGACATAAGATTGAAAGAT
Cucurbita pepo                    CTTATGGACCTAAGATAGGGCACATCCCTCACGACATAAGATTGAAAGAT
PB235_rps4                        CTTATGGACCGAACATAGGTCATATCCCTCACGACATAAGATTGAAAGAT
RRIC110_rps4                      CTTATGGACCGAACATAGGTCATATCCCTCACGACATAAGATTGAAAGAT
Lotus japonicus                   TTTATGGACCTAACATAGGTCACATCCCTCACGACATAAGATTGAAAGAT
Millettia pinnata                 TTTATGGACCTAACATAGGTCACATCCCTCACGACATAAGATTGAAAGAT
BPM24_rps4                        CTTATGGACCGAACATAGGTCATATCCCTCACGACATAAGATTGAAAGAT
```

```
RRIT251_rps4                      CTTATGGACCGAACATAGGTCATATCCCTCACGACATAAGATTGAAAGAT
RRII105_rps4                      CTTATGGACCGAACATAGGTCATATCCCTCACGACATAAGATTGAAAGAT
Beta macrocarpa                   UTTATGGACCTAACATAGATCACATCCCTCACGACATAAGATTGAAAGAT
Beta vulgaris vulgaris            UTTATGGACCTAACATAGATCACATCCCTCACGACATAAGATTGAAAGAT
Beta vulgaris maritima            UTTATGGACCTAACATAGATCACATCCCTCACGACATAAGATTGAAAGAT
                                   ********* ** ****  ** *******************  ******

Arabidopsis thaliana              UUAAACCTTCUTCTTUGGAGCAGAAACGGACGTGGCCAAAACATATAAA-
Brassica napus                    UUAAACCTTCUTCTTUGGAGCAGAAACGGACGTGGCCAAAACATATAAA-
Oryza sativa Japonica             CUAAACCTTCUTCTTUGGAGCGGAAACGGACGTGGCCAAAACATATAAAA
RRIM600_rps4                      TTAAACCTTCUTCTTUGGAGCGGAAACGGACGTGGCCAAAACATATAA--
Citrullus lanatus                 CUAAACCTTCCTCTTCGGAGCGGAAACGGACGTGGCCAAAACATATAAA-
Cucurbita pepo                    CCAAACCTTCCTCTTCGGAGCGCAAACGGCCGTGGCCAAAACATATAAA-
PB235_rps4                        TTAAACCTTCNTCTTNGGAGCGGAAACGGACGTGGCCAAAACATATAA--
RRIC110_rps4                      TTAAACCTTCNTCTTNGGAGCGGAAACGGACGTGGCCAAAACATATAA--
Lotus japonicus                   CUAAACCTTCUTCTTUGGAGCGGAAACGGACGTGGCCAAAACATATAAA-
Millettia pinnata                 CUAAACCTTCUTCTTUGGAGCGGAAACGGACGTGGCCAAAACATATAAA-
BPM24_rps4                        TTAAACCTTCUTCTTUGGAGCGGAAACGGACGTGGCCAAAACATATAA--
RRIT251_rps4                      TTAAACCTTCUTCTTUGGAGCGGAAACGGACGTGGCCAAAACATATAA--
RRII105_rps4                      TTAAACCTTCNTCTTNGGAGCGGAAACGGACGTGGCCAAAACATATAA--
Beta macrocarpa                   CUAAACCTTCUTCTTUGGAGCGGAAAGGGACGTGGCCAAAACACATAAA-
Beta vulgaris vulgaris            CUAAACCTTCUTCTTUGGAGCGGAAAGGGACGTGGCCAAAACACATAAA-
Beta vulgaris maritima            CUAAACCTTCUTCTTUGGAGCGGAAAGGGACGTGGCCAAAACACATAAA-
                                    ******** **** *****  *** ** ************* ****

Arabidopsis thaliana              ---GATCGGCGTAGTCACTCATAGGGACATCTCTATCCGGATAGAGGATA
Brassica napus                    ---GATCGGCGTAGTCACTCGTAGTAGGA-----GTCAAGATATTGCGTA
Oryza sativa Japonica             AAAGATCGGCCTAGTCGCTCATAGGGACATATCTATCCGGATAGAGGATA
RRIM600_rps4                      --------------------------------------------------
Citrullus lanatus                 ---GATCGGCGTAGTCGCTCATAGGGACATATCTATCCGTCTCGAGTCTC
Cucurbita pepo                    ---GATCGGC----TCGCTC----GGACATATCTATCCGTCTCGAGTCTC
PB235_rps4                        --------------------------------------------------
RRIC110_rps4                      --------------------------------------------------
Lotus japonicus                   ---GATCGGCGTAGTCGCTCATAGGGACATATCTATCCGGATAGAGGATA
Millettia pinnata                 ---GATCGGCGTAGTCGCTCATAGGGACATATCTATCCGGATAGAGGATA
BPM24_rps4                        --------------------------------------------------
RRIT251_rps4                      --------------------------------------------------
RRII105_rps4                      --------------------------------------------------
Beta macrocarpa                   ---GATCGGCGTAGTCGCTCATAGGGACATATCTATCCGGATAGAGATAA
Beta vulgaris vulgaris            ---GATCGGCGTAGTCGCTCATAGGGACATATCTATCCGGATAGAGATAA
Beta vulgaris maritima            ---GATCGGCGTAGTCGCTCATAGGGACATATCTATCCGGATAGAGATAA


Arabidopsis thaliana              GTCTAGATCGATTCATAGATGGATTTT--TTCATCTAGATAGGTATC-CC
Brassica napus                    ---TAAATATATATAAAGAGAGAGTCT--CTTGAGCGGCCGAGAATC-TT
Oryza sativa Japonica             GTCTAGATCAATTTATTGATAAATCTCTCTCTATATAGATAGGTATCTCT
RRIM600_rps4                      --------------------------------------------------
Citrullus lanatus                 GTCTAGATCGATTCATAGATCGATTTCTATCCATATGTATCGGTATCTCC
Cucurbita pepo                    GTGACGATCGATTCATAGATCGATTTCTATCCATATGTAGCGGTATCGCC
PB235_rps4                        --------------------------------------------------
RRIC110_rps4                      --------------------------------------------------
Lotus japonicus                   GTCTAGATCGATTCATAGATAGATTTCTATCCATATAGATAGGTATCTCC
Millettia pinnata                 GTCTAGATCGATTCATAGATAG--------CCATATAGATAGGTATCTCC
BPM24_rps4                        --------------------------------------------------
RRIT251_rps4                      --------------------------------------------------
RRII105_rps4                      --------------------------------------------------
Beta macrocarpa                   GGACAGGGATCCATCTAGAAAAAAT---ATTGAACCGGTTTCATTTTTTT
Beta vulgaris vulgaris            GGACAGGGATCCATCTAGAAAAAAT---ATTGAACCGGTTTCATTTTTTT
Beta vulgaris maritima            GGACAGGGATCCATCTAGAAAAAAT---ATTGAACCGGTTTCATTTTTTT


Arabidopsis thaliana              GTGGATAGAGAGAAAGATAGGGATCCATCTAGATCTT--GATTCACTATT
```

```
Brassica napus               ATGT-CAAAAGGACCAAGGACGATCTTTTCGGAAAGGAGGAGTCAGTCTT
Oryza sativa Japonica        GTGGATAGAGATAAAGATAGGGATCCCTATAAATC--GAAAAGTGCACTT
RRIM600_rps4                 --------------------------------------------------
Citrullus lanatus            GTGGATAGAGATAAAGATAGGGATCCATCTAGATCTTGATTCACTATTT-
Cucurbita pepo               GTGGATAGAGATAAAGATAGGGATCCATCTAGATCTTGATTCACTATTTG
PB235_rps4                   --------------------------------------------------
RRIC110_rps4                 --------------------------------------------------
Lotus japonicus              GTGGATAGAGATAAAGATAGGGATCCATCTAGATCTTGCTTGATTCACTA
Millettia pinnata            GTGGATAGAGATAAAGATAGGGATCCATCTAGATCTTGCTTGATTCACTA
BPM24_rps4                   --------------------------------------------------
RRIT251_rps4                 --------------------------------------------------
RRII105_rps4                 --------------------------------------------------
Beta macrocarpa              TTTTCTTGATTCTGATTGATTGCCTTTTTTTGACGACAAGTTTTAA----
Beta vulgaris vulgaris       TTTTCTTGATTCTGATTGATTGCCTTTTTTTGACGACAAGTTTTAA----
Beta vulgaris maritima       TTTTCTTGATTCTGATTGATTGCCTTTTTTTGACGACAAGTTTTAA----


Arabidopsis thaliana         TCC---ATTTTTTTTTACTTAGTC----
Brassica napus               CTTTGAAGATCGAGGCGCGTAGCGGACA
Oryza sativa Japonica        TTTGACTACT------------------
RRIM600_rps4                 ----------------------------
Citrullus lanatus            -----CCATTTTTTTGA-----------
Cucurbita pepo               ATTGATTGCCTTTTTCAACAAG------
PB235_rps4                   ----------------------------
RRIC110_rps4                 ----------------------------
Lotus japonicus              TTT--CCATTTTTTTC------------
Millettia pinnata            TTT--CCATTTTTTTTTCTTGATT----
BPM24_rps4                   ----------------------------
RRIT251_rps4                 ----------------------------
RRII105_rps4                 ----------------------------
Beta macrocarpa              ----------------------------
Beta vulgaris vulgaris       ----------------------------
Beta vulgaris maritima       ----------------------------
```
